# The South American Fruit Fly: An Important Pest Insect With RNAi-Sensitive Larval Stages

**DOI:** 10.3389/fphys.2019.00794

**Published:** 2019-06-27

**Authors:** Naymã Dias, Deise Cagliari, Frederico Schmitt Kremer, Leticia Neutzling Rickes, Dori Edson Nava, Guy Smagghe, Moisés Zotti

**Affiliations:** ^1^Molecular Entomology and Applied Bioinformatics Laboratory, Faculty of Agronomy, Department of Crop Protection, Federal University of Pelotas, Pelotas, Brazil; ^2^Bioinformatics and Proteomics Laboratory, Technological Development Center, Federal University of Pelotas, Pelotas, Brazil; ^3^Entomology Laboratory, Embrapa Clima Temperado, Pelotas, Brazil; ^4^Faculty of Bioscience Engineering, Department of Plants and Crops, Ghent University, Ghent, Belgium

**Keywords:** RNA interference, transcriptome, gene silencing, Diptera, *Anastrepha fraterculus*

## Abstract

RNA interference (RNAi) technology has been used in the development of approaches for pest control. The presence of some essential genes, the so-called “core genes,” in the RNAi machinery is crucial for its efficiency and robust response in gene silencing. Thus, our study was designed to examine whether the RNAi machinery is functional in the South American (SA) fruit fly *Anastrepha fraterculus* (Diptera: Tephritidae) and whether the sensitivity to the uptake of double-stranded RNA (dsRNA) could generate an RNAi response in this fruit fly species. To prepare a transcriptome database of the SA fruit fly, total RNA was extracted from all the life stages for later cDNA synthesis and Illumina sequencing. After the *de novo* transcriptome assembly and gene annotation, the transcriptome was screened for RNAi pathway genes, as well as the duplication or loss of genes and novel target genes to dsRNA delivery bioassays. The dsRNA delivery assay by soaking was performed in larvae to evaluate the gene-silencing of *V-ATPase*, and the upregulation of *Dicer-2* and *Argonaute-2* after dsRNA delivery was analyzed to verify the activation of siRNAi machinery. We tested the stability of dsRNA using dsGFP with an *in vitro* incubation of larvae body fluid (hemolymph). We identified 55 genes related to the RNAi machinery with duplication and loss for some genes and selected 143 different target genes related to biological processes involved in post-embryonic growth/development and reproduction of *A. fraterculus*. Larvae soaked in dsRNA (dsV-ATPase) solution showed a strong knockdown of V-ATPase after 48 h, and the expression of *Dicer-2* and *Argonaute-2* responded with an increase upon the exposure to dsRNA. Our data demonstrated the existence of a functional RNAi machinery in the SA fruit fly, and we present an easy and robust physiological bioassay with the larval stages that can further be used for screening of target genes at *in vivo* organisms’ level for RNAi-based control of fruit fly pests. This is the first study that provides evidence of a functional siRNA machinery in the SA fruit fly.

## Introduction

The South American fruit fly (SA fruit fly), *Anastrepha fraterculus*, is a major polyphagous pest of fruit crops. This fruit fly species occurs from the Southern United States (Texas) and Mexico to Argentina and is associated with 116 plant species in Brazil alone (Zucchi, 2008). Oviposition and larval feeding of *A. fraterculus* cause the damage that leads to accelerated ripening and premature fruit dropping (Aluja, 1994). Importantly, its presence limits access to markets because of quarantine limitations imposed by fruit-fly free countries (Lanzavecchia et al., 2014; Dias et al., 2018). The global losses caused by fruit flies can range 2 billion USD annually, and in Brazil, the economic losses are between $120 and 200 million USD per year (Macedo et al., 2017).

Currently, the control tactic available for *A. fraterculus* is the application of bait sprays (Dias et al., 2018). However, the control of SA fruit fly by chemical tactics is becoming increasingly restricted, because the effective but broad-spectrum neurotoxic and systemic-acting insecticides have been banned for commercialization (Böckmann et al., 2014). Fruit growers are also seeking new economic fruit fly control options, especially environmentally sustainable tactics (Sarles et al., 2015). Thus, RNA interference (RNAi) is a promising strategy for pest control used to suppress the expression of key genes (Katoch et al., 2013; Andrade and Hunter, 2017). Molecules of double-stranded RNA (dsRNA) are RNAi triggers that promote the post-transcriptional down-regulation of a target-gene (Elbashir et al., 2001). Some features, such as high target gene specificity and lack of environmental persistence, make RNAi technique desirable for crop protection (Huvenne and Smagghe, 2010; Zotti et al., 2018).

Efficient gene silencing by RNAi in insects requires some essential elements, such as dsRNA processing by RNAi enzymes, dsRNA uptake into cells, and expression of the RNAi core genes (Huvenne and Smagghe, 2010; Wang et al., 2016; Christiaens et al., 2018; Niu et al., 2018). *Drosophila* species have been used as a model for RNAi studies in Diptera. However, this species shows low sensibility to dsRNA uptake by cells, and consequently it is necessary to use transfection agents for delivery of dsRNA molecules (Taning et al., 2016). Soaking of *Drosophila melanogaster* larvae for a period of 1 h with naked dsRNA resulted in only 5–8% of knockdown for *b-glucuronidase* (*gus*) (Whyard et al., 2009). In *Drosophila suzukii* larvae, the RNAi efficiency varied between 20 and 40% in a study using dsRNA formulated with transfection reagent (Taning et al., 2016). For *Bactrocera dorsalis*, Shi et al. (2017) found knockdown around 50% in larval stages. This fact raises the question about the variability in the uptake routes and uptake mechanisms between different Diptera species (Whyard et al., 2009).

Thus, the understanding of the RNAi pathway in the target organism can provide information about use of this tool for pest control (Vélez et al., 2016). However, for the SA fruit fly, we first need the molecular information on RNAi core genes, in addition to insights into the gene-silencing mechanism by RNAi.

This paper is the first report of RNAi bioassays in the SA fruit fly together with a transcriptome analysis over the life stages of eggs, larvae, pupae, and female and male adults. We aimed to provide a genetic database to better understand this important pest insect and to screen for genes related to the RNAi machinery. We also aimed to identify possible gene duplication, gene loss, and novel target genes for dsRNA bioassays. Hence, we focus also on genes related to insect-specific biological processes involved in post-embryonic growth/development and reproduction as potential future insecticidal target genes.

In addition, we developed a straightforward experimental RNAi setup by soaking the SA fruit fly larvae. If successful, it is an easy and robust bioassay for the larval stages that can be used to screen target genes *in vivo* at organism level. In order to validate the RNAi response, we first investigated the expression of *Dicer-2* and *Argonaute-2*. Next, we investigated the silencing of *V-ATPase* and the insect mortality. Finally, we measured the stability of dsRNA with an *in vitro* incubation together with hemolymph. Overall, this study will be the first to provide evidence of a functional siRNA machinery in the SA fruit fly.

## Materials and Methods

### SA Fruit Fly Colony and Maintenance

A colony of *A. fraterculus* was originally field-collected in 2015 from an orchard of strawberry guava (*Psidium cattleianum*) in Pelotas, Rio Grande do Sul, Brazil (31°40′47″ S e 52°26′24″ W) and was reared for thirteen generations before use in the experiments. SA fruit fly stages were maintained under standard conditions (temperature: 25 ± 1°C; RH: 70 ± 10% and 14L:10D photoperiod). The rearing methods were the same as described by Gonçalves et al. (2013).

### RNA Extraction, cDNA Library, and RNA-Seq

Total RNA was extracted from eggs, larvae (first-, second-, and third-instar), pupae, and adults (female and male) of SA fruit fly using the RNAzol (GeneCopoeia, Rockville, MD) and treated with DNase I (Invitrogen, Carlsbad, CA), following the manufacturer’s instructions. The RNA samples were pooled prior to cDNA synthesis. The RNA quality and concentration were examined on the Agilent 2100 Bioanalyzer and cDNA library was performed using the TruSeq RNA Sample Prep kit (Illumina, San Diego, CA) protocol. The library was paired-end (2x125bp) sequenced (RNA-Seq) using the Illumina HiSeq2500 platform. Raw sequence data were submitted to the Short Read Archive (SRA) of the NCBI database (accession number SRP157027).

### Reads Quality Control and *de novo* Assembly

All reads were trimmed for quality and length using the software Trimmomatic^1^ and the quality was checked using the software FastQC^2^. Phred score over 30 across more than 70% of the bases was used as a high-quality threshold. The high-quality reads were *de novo* assembled using Trinity software^3^ since there is no reference genome sequence for *A. fraterculus*. This software uses a De Bruijn graph algorithm and was executed using default settings, a k-mer length of 25.

### Transcriptome Analysis and Target-Genes Database

The contigs generated by Trinity were aligned to the UniProt database using Diamond algorithm (Buchfink et al., 2015) and only those with hits on insects (E-value of 1e-10) were selected for analysis. For functional categorization by gene ontology (GO), a similarity search was performed to predict the contigs generated by searching the UniProt database with the Diamond. The predicted genes were used as query in QuickGo from EBI^4^ and to calculate GO terms. A database was generated for novel target genes related to post-embryonic growth and development of the SA fruit fly larvae and the reproduction events in adults. The target genes for SA fruit fly database were searched in QuickGo using the GO terms related to biological processes: larval development (GO:0002164), imaginal disk morphogenesis (GO:0007560), post-embryonic development (GO:0009791), female sex differentiation (GO:0046660), sexual reproduction (GO:0019953), genital disk anterior/posterior pattern formation (GO:0035224) and oviposition (GO:0018991). The *D. melanogaster* sequences corresponding to the genes found were recovered in UniProt database and were used as an input to search the transcriptome from *A. fraterculus* using the tblastn tool with a threshold bit score ≥ 150 and E-value ≤ 1e-5 (Supplementary Material S1).

### Identification of RNAi Machinery Genes

A list of RNAi-related genes, as employed by Swevers et al. (2013), Prentice et al. (2015), and Yoon et al. (2016), was selected covering the RNAi core machinery, auxiliary factors (from RISC), dsRNA uptake, nucleases, antiviral RNAi, intracellular transport, and lipid metabolism. Homologous sequences from *D. melanogaster* corresponding to RNAi-related genes were obtained in UniProt database and were used as an input to search the transcriptome from SA fruit fly (Supplementary Material S2). Alternatively, sequences of *Drosophila* and Tephritidae species were used in the absence of sequences of *D. melanogaster* (Supplementary Material S2). The ORF Finder tool from NCBI was used to detect open reading frames. The protein domains were predicted by NCBI Conserved Domains using the Conserved Domain Database (CDD) (Supplementary Material S2). A similarity search was performed using the BLASTp against the NCBI database to confirm the identity of the RNAi-related genes (Supplementary Material S4).

### Potential Loss and Duplication of RNAi-Related Genes

We screened the SA fruit fly transcriptome for the copy number of the 10 RNAi pathway genes using tblastn tool. The number of copies was based in the number of genes obtained by Trinity assembly. The distribution of these genes was compared to related insects, following the results showed by Dowling et al. (2016). We also searched for genes for a systemic RNAi response, as *SID-1* found in cells of *Caenorhabditis elegans* (Winston et al., 2002).

### Phylogenetic Analysis

A phylogenetic analysis was constructed to provide additional confirmation of the main siRNA machinery genes (*Dicer-2* and *Argonaute-2*) and the candidate gene for silencing (*Vacuolar-proton-ATPase*) from the *A. fraterculus* transcriptome. Phylogenetic trees were constructed through the Neighbor-Joining method in the MEGA X software using the bootstrapping reconstructions (1000 replicates). The selected species and accession numbers of the sequences used for analysis are shown in Supplementary Table S4.

### dsRNA Synthesis

The *A. fraterculus* transcriptome was searched for the *Vacuolar-proton-ATPase V0-domain* (*V-ATPase V0*) using *D. melanogaster* sequence as a query. Primers were designed from the *A. fraterculus* transcriptome sequences using Primer3^5^. The *V-ATPase V0* fragment (483 pb) was amplified by PCR using cDNA from second-instar larvae of *A. fraterculus* as a template, synthesized with SuperScript First-Strand Synthesis System for RT-PCR (Invitrogen, Carlsbad, CA). For dsRNA synthesis of *green fluorescent protein* (*GFP*), a 560 bp *GFP* fragment was amplified by PCR using plasmid pIG1783f. The GFP amplicon was confirmed by Sanger sequencing. The primers used for the PCR are listed in Supplementary Table S1.

The dsRNA templates were generated by PCR using primers with a T7 promoter region at the 5′ end of each primer (Supplementary Table S1). The PCR products were used for *in vitro* transcription and purification using MEGAscript kit (Ambion, Austin, TX) according to the manufacturer’s instructions. Synthesized dsRNA products were quantitated by a Nanovue spectrophotometer (GE Healthcare, Little Chalfont, United Kingdom) at 260 nm and the integrity was confirmed by electrophoresis on 1% agarose gel.

### RNAi by Soaking of Larval Stages

The soaking treatment was performed using second-instar larvae of *A. fraterculus*. The dsRNA of *V-ATPase V0* (ds*VTP*) was diluted with RNase-free water to yield 500 ng/μl, considering the data reported by Whyard et al. (2009). The ds*GFP* in the same concentration was used as control for the soaking assays. The insects were starved for 1 h and each larva was soaked in a 200 μl-tube with 25 μl of dsRNA solution for 30 min. After soaking, the treated larvae were transferred to artificial diet (Nunes et al., 2013). The mortality of the insects was monitored over a 7-day period.

Larvae of *A. fraterculus* were stored at -80°C at 24, 48, and 72 h after soaking with dsRNA for the RNAi silencing efficiency assay. The RNA was extracted with three biological replicates at each time point, using RNAzol (GeneCopoeia, Rockville, MD) following the manufacturer’s instructions. After, the RNA samples were incubated with 10 U DNase I (Invitrogen, Carlsbad, CA) at 37°C for 30 min. The RNA was quantified using a Nanovue spectrophotometer (GE Healthcare, Little Chalfont, United Kingdom) and verified by 2% agarose gel electrophoresis. The cDNA was produced from 2 μg RNA using the SuperScript First-Strand Synthesis System for RT-PCR (Invitrogen, Carlsbad, CA).

### Measurement of RNAi Efficacy

Real-time Quantitative PCR analysis (qPCR) was performed to evaluated RNAi efficacy using a LightCycler 480 (Roche Life Science, Switzerland). A standard curve based on a serial dilution (1:1, 1:5, 1:25, and 1:125) of cDNA was performed to validate the primers used in the analysis (Supplementary Table S1). The reactions included 5 μl of EvaGreen 2X qPCR MasterMix (ABM, Canada), 0.3 μl (10 μM) of forward primer, 0.3 μl (10 μM) of reverse primer, 3.4 μl of nuclease-free water and 1 μl of cDNA, in a total volume of 10 μl. The qPCR conditions included 10 min at 95°C followed by 40 cycles of 30 s at 95°C, 45 s at 59°C, and 30 s at 77°C, interrupted by the dissociation curve with denaturation at 95°C (5 s), cooling at 70°C (1 min) and gradually heating at 0.11°C steps up to 95°C and cooling at 40°C (30 s), as described by Benemann et al. (2017). The reactions were set-up in 96-well microtiter plates (Roche Life Science, Indianapolis, IN), using the cDNA dilution of 1:25, with three technical replicates and no-template controls. Relative mRNA expression of the *V-ATPase* gene was normalized to the housekeeping genes *α-tubulin* and *actin* by the equation ratio 2-ΔΔCt (Livak and Schmittgen, 2001). The data were analyzed using analysis of variance (one-way ANOVA) and *t*-test (*p* ≤ 0.05).

### Expression of siRNA Genes *Dcr-2* and *Ago-2* Upon Exposure to dsRNA

The regulation of siRNA pathway genes during the SA fruit fly RNAi bioassay was determined by the expression of *Dicer-2* (*Dcr-2*) and *Argonaute-2* (*Ago-2*) in response to soaking with ds*GFP.* The *Dcr-2* and *Ago-2* sequences found in the *A. fraterculus* transcriptome were used for primers design using the Primer3. The primers used for the qPCR are listed in Supplementary Table S1. The qPCR analysis was performed as described above, and the expression responses were evaluated at 24, 48, and 72 h after larvae soaking with ds*GPF*.

### dsRNA Degradation Assay

Body fluid (hemolymph) was collected in pre-chilled 1.5 ml-tubes by centrifugation at 13,000 rpm for 10 min at 4°C from 5 second-instar larvae. For the degradation assay, 20 μl of ds*GFP* solution (500 ng/μl dsRNA) was mixed with 2 μl of body fluid and incubated at 25°C. We collected aliquots of 5 μl at 0, 1, 2, and 4 h after incubation and the same volume of EDTA (10 mM) was added to stop the enzymatic reaction. The samples were stocked at -80°C until the analysis. The results were verified by 1.5% agarose gel electrophoresis and the bands were analyzed using the Gel Analyzer software.

## Results

### SA Fruit Fly Transcriptome Analysis

The RNA sequencing generated a total of 103,808,135 reads of 125 bp length. The assembled transcriptome consisted of 163,359 transcripts, which accounted for 84,105 contigs (Supplementary Table S2). Of all contigs, 72,388 are eukaryotic. The length distribution of Eukaryote contigs in *A. fraterculus* transcriptome is shown in Supplementary Figure S1.

The Diamond analysis produced 73,193 hits, representing 45% of the total contigs (Supplementary Figure S2). For the sequences with significant hits, 72% of the contigs were similar to sequences from fruit fly species: 17% to *Ceratitis capitata*, 16% to *Zeugodacus cucurbitae*, 15% to *B. dorsalis* and *Bactrocera latifrons*, 9% to *Bactrocera tryoni*, and 28% to other organisms. The species distribution of the top 30 hits is shown in Supplementary Table S3. Considering the insect genera, the contigs were similar to sequences from Diptera, which featured for 55% to *Bactrocera*, 16% to *Ceratitis*, 3% to *Drosophila*, 1% to *Tabanus*, 0.9% to *Glossina*, 0.8% to *Lucilia*, and 20% to other insect genera.

**FIGURE 1:**
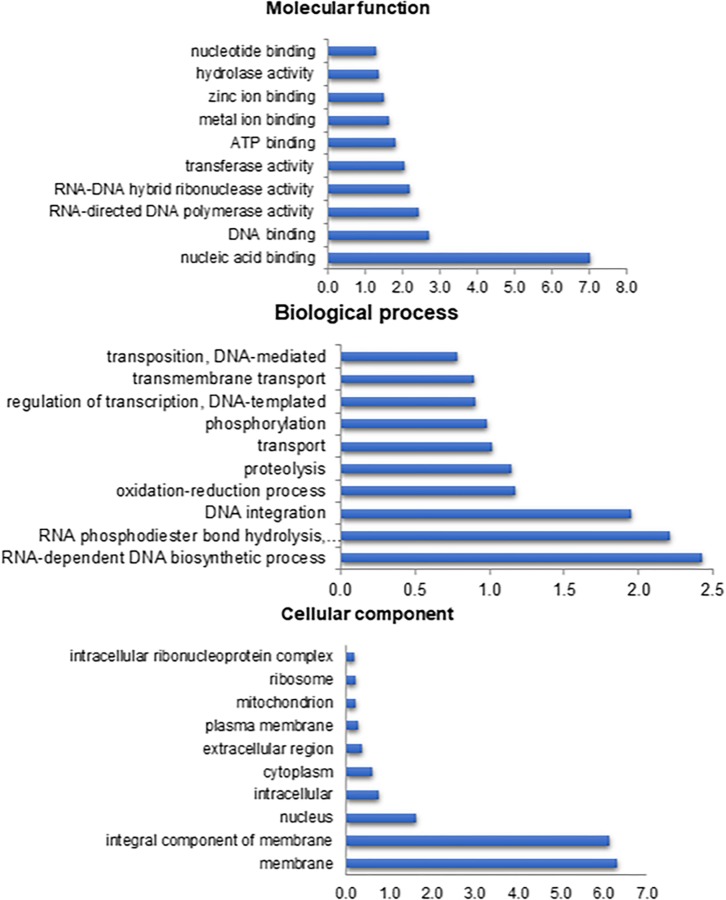
Percentage of *Anastrepha fraterculus* contigs assigned to a certain gene ontology term as predicted by QuickGO from EBI. Top 10 terms are shown.

The GO terms calculated starting the Diamond similarity search were grouped into three main categories: molecular function (48%), biological process (31%) and cellular component (20%). A total of 167,729 predicted GO terms was obtained. The major GO terms within the molecular function category were nucleic acid binding (11,734; 7%), for the biological processes it was RNA-dependent DNA biosynthetic process (4,070; 2%), and for the cellular component, it was the membrane (10,584; 6%) (Figure 1).

### Target Genes Related to Post-embryonic Growth/Development and Reproduction Events

We selected 143 different target genes related to biological processes involved in post-embryonic growth/development and reproduction of *A. fraterculus*, with preference for sequences with annotations reviewed by Swiss-Prot and with experimental evidence. The target genes selected are involved in five biological processes: larval development (54 genes), imaginal disk morphogenesis (22 genes), post-embryonic development (12 genes), sexual reproduction (44 genes), female sex differentiation (2), genital disk anterior/posterior pattern formation (2) and oviposition (7). The results are shown in Supplementary Material S1.

### RNAi Machinery Genes Are Present in SA Fruit Fly

**FIGURE 2:**
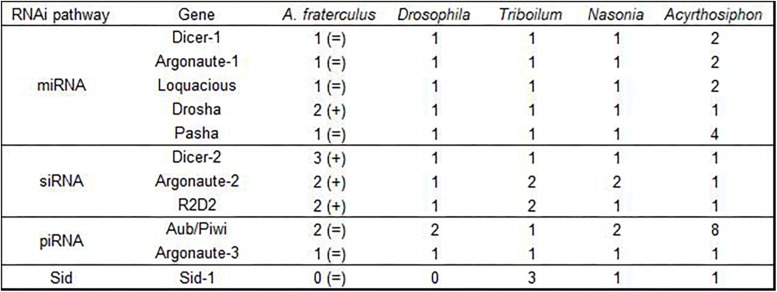
Copy number of the ten RNAi-related genes and *SID-1* found in *Anastrepha fraterculus* transcriptome by Trinity and in other insect species (showed by Dowling et al., 2016). The number of copies showed in *A. fraterculus* is compared to *Drosophila*. (=) Same and (+) duplication.

We identified 55 genes related to the RNAi machinery in the *A. fraterculus* transcriptome (Table 1). The sequences of the genes of the miRNA, siRNA, and piRNA pathways, auxiliary factors (from RISC), dsRNA uptake, intracellular transport, antiviral RNAi, nucleases, and lipid metabolism showed most structural and functional units conserved (protein domains) (Supplementary Material S2). The number of the copies at which these genes were found in *A. fraterculus* is shown in Figure 2.

**TABLE 1:** Overview of the presence of genes related to the RNAi pathways in the *Anastrepha fraterculus* transcriptome.

	**Contig**	**First hit tblastn**	**ID taxon homologue**	**Comparison to homologue**	**Identity (%)**
**miRNA**					
Dicer-1	TRINITY_DN33861_c2_g1_i1	Endoribonuclease 9 (*Drosophila melanogaster*)	Q9VCU9	*E* = 0.0; bits = 2728	62
Argonaute-1	TRINITY_DN32900_c0_g1_i7	Argonaute-1, isoform A (*Drosophila melanogaster*)	Q32KD4	*E* = 0.0; bits = 1823	94
Loquacious	TRINITY_DN27977_c3_g1_i4	Loquacious (*Drosophila melanogaster*)	Q4TZM6	*E* = 6e-106; bits = 332	72
Drosha	TRINITY_DN30547_c4_g2_i1	Drosha (*Drosophila melanogaster*)	Q7KNF1	*E* = 0.0; bits = 1719	73
Pasha	TRINITY_DN28163_c0_g1_i6	Partner of drosha, isoform B (*Drosophila melanogaster*)	A0A0B4KI70	*E* = 0.0; bits = 809	70
Exportin-5	TRINITY_DN23399_c0_g1_i2	exportin-5 isoform X1 (*Drosophila ficusphila*)	A0A1W4VG06	*E* = 0.0; bits = 1634	67
**siRNA**					
*Dicer-2*	TRINITY_DN32516_c1_g2_i1	Dicer-2, isoform A (*Drosophila melanogaster*)	A1ZAW0	*E* = 0.0; bits = 1582	48
*Argonaute-2*	TRINITY_DN30039_c4_g1_i5	Protein argonaute-2 (*Drosophila melanogaster*)	Q9VUQ5	*E* = 0.0; bits = 834	53
R2D2	TRINITY_DN28410_c0_g2_i4	R2D2 (*Drosophila melanogaster*)	Q2Q0K7	*E* = 9e-085; bits = 277	47
**piRNA**					
Argonaute-3	TRINITY_DN27717_c4_g1_i3	Protein argonaute-3 (*Drosophila melanogaster*)	Q7PLK0	*E* = 0.0; bits = 1056	57
Piwi	TRINITY_DN30302_c0_g2_i1	Protein piwi (*Drosophila melanogaster*)	Q9VKM1	*E* = 0.0; bits = 1046	63
Aubergine	TRINITY_DN30302_c0_g1_i1	Protein aubergine (*Drosophila melanogaster*)	O76922	*E* = 0.0; bits = 1081	64
Zucchini	TRINITY_DN31164_c0_g2_i2	Zucchini (*Drosophila melanogaster*)	L0CR90	*E* = 3e-053; bits = 183	42
**Auxiliary factors (from RISC)**					
Tudor-SN	TRINITY_DN30816_c0_g1_i2	LD20211p (*Drosophila melanogaster*)	Q9W0S7	*E* = 0.0; bits = 1503	82
Vasa intronic (VIG)	TRINITY_DN23682_c0_g1_i2	LD07162 (*Drosophila melanogaster*)	Q9V426	*E* = 1e-066; bits = 233	49
FMR	TRINITY_DN33674_c0_g2_i3	Synaptic functional regulator FMR1 (*Drosophila melanogaster*)	Q9NFU0	*E* = 0.0; bits = 750	74
Rm62	TRINITY_DN31247_c0_g1_i3	ATP-dependent RNA helicase p62 (*Drosophila melanogaster*)	P19109	*E* = 0.0; bits = 716	91
Translin	TRINITY_DN31480_c3_g3_i11	GM27569p (*Drosophila melanogaster*)	Q7JVK6	*E* = 2e-122; bits = 372	74
Translin associate factor X	TRINITY_DN24775_c0_g1_i2	translin-associated protein X (*Drosophila ficusphila*)	A0A1W4VFE4	*E* = 4e-124; bits = 367	61
Armitage	TRINITY_DN31912_c0_g1_i3	Probable RNA helicase armi (*Drosophila melanogaster*)	Q6J5K9	*E* = 0.0; bits = 1164	50
Homeless (spindle-E)	TRINITY_DN31966_c0_g1_i1	ATP-dependent RNA helicase spindle-E (*Drosophila melanogaster*)	Q9VF26	*E* = 0.0; bits = 1281	48
Maelstrom	TRINITY_DN28061_c2_g2_i5	Protein maelstrom (*Drosophila yakuba*)	B4PIP5	*E* = 6e-085; bits = 279	38
HEN1	TRINITY_DN27986_c1_g1_i3	Small RNA 2′-*O*-methyltransferase (*Drosophila melanogaster*)	Q7K175	*E* = 3e-103; bits = 319	47
RNA helicase Belle	TRINITY_DN28586_c1_g3_i2	ATP-dependent RNA helicase bel (*Drosophila melanogaster*)	Q9VHP0	*E* = 0.0; bits = 892	86
PRP16	TRINITY_DN32795_c0_g2_i1	pre-mRNA-splicing factor ATP-dependent RNA (*Drosophila ficusphila*)	A0A1W4VUB2	*E* = 0.0; bits = 737	93
Gemin3	TRINITY_DN30190_c0_g1_i1	BcDNA.LD05563 (*Drosophila melanogaster*)	Q9V3C4	*E* = 3e-131 bits = 430	49
Gawky	TRINITY_DN27487_c0_g4_i19	Protein Gawky (*Drosophila melanogaster*)	Q8SY33	*E* = 0.0; bits = 803	55
Staufen	TRINITY_DN33993_c3_g1_i10	Maternal effect protein staufen (*Drosophila melanogaster*)	P25159	*E* = 2e-159; bits = 523	51
Clip 1	TRINITY_DN32205_c1_g4_i1	CLIP-associating protein (*Drosophila melanogaster*)	Q9NBD7	*E* = 0.0; bits = 1765	64
Elp-1	TRINITY_DN33357_c0_g1_i4	Putative elongator complex protein 1 (*Drosophila melanogaster*)	Q9VGK7	*E* = 0.0; bits = 1102	48
GLD-1	TRINITY_DN24535_c0_g1_i2	Protein held out wings (*Drosophila melanogaster*)	O01367	*E* = 0.0; bits = 527	86
ACO-1	TRINITY_DN30096_c0_g1_i6	1-aminocyclopropane-1-carboxylate oxidase (*Bactrocera dorsalis*)	A0A034VX75	*E* = 0.0; bits = 753	92
**dsRNA uptake**					
Scavenger receptor	TRINITY_DN31545_c2_g1_i7	Scavenger receptor isoform A (*Drosophila melanogaster*)	Q9VM10	*E* = 0.0; bits = 717	66
Eater	TRINITY_DN33643_c4_g2_i2	Eater (*Drosophila melanogaster*)	Q9VB78	*E* = 6e-107; bits = 370	41
Clathrin Heavy chain	TRINITY_DN29160_c0_g1_i4	Clathrin heavy chain (*Drosophila melanogaster*)	P29742	*E* = 0.0; bits = 3150	94
FBX011	TRINITY_DN32848_c4_g1_i12	GM01353p (*Drosophila melanogaster*)	Q6NQY0	*E* = 0.0; bits = 1540	86
HPS4 = CG4966	TRINITY_DN31238_c0_g1_i2	Hermansky-Pudlak syndrome 4 ortholog (*Drosophila melanogaster*)	A1ZAX6	*E* = 0.0; bits = 604	61
Adaptor protein 50 (Ap50)	TRINITY_DN29475_c0_g1_i1	AP-50 (*Drosophila simulans*)	B4R022	*E* = 0.0; bits = 899	99
TRF3	TRINITY_DN30474_c2_g1_i5	Similar to Drosophila transferrin (Fragment) (*Drosophila yakuba*)	Q6XHM9	*E* = 5e-098; bits = 294	77
Sortilin Like Receptor	TRINITY_DN26733_c0_g2_i34	Sortilin-related receptor (Fragment) (*Bactrocera dorsalis*)	A0A034V651	*E* = 0.0; bits = 856	79
Innexin2 (Gap Junction)	TRINITY_DN33133_c1_g1_i6	Innexin inx2 (*Drosophila melanogaster*)	Q9V427	*E* = 0.0; bits = 644	93
Low density lipoprotein	TRINITY_DN19392_c0_g3_i1	Low-density lipoprotein receptor-related (*Drosophila melanogaster*)	A1Z9D7	*E* = 0.0; bits = 1407	83
TRF2	TRINITY_DN32249_c1_g1_i3	LD22449p (*Drosophila melanogaster*)	Q9VTZ5	*E* = 0.0; bits = 1307	76
**Intracellular transport**					
Vha16	TRINITY_DN29956_c2_g1_i7	V-type proton ATPase 16 kDa subunit (*Drosophila melanogaster*)	P23380	*E* = 2e-088; bits = 284	95
VhaSFD	TRINITY_DN26174_c1_g1_i6	V-type proton ATPase subunit H (*Drosophila melanogaster*)	Q9V3J1	*E* = 0.0; bits = 675	90
Small Rab GTPases (Rab7)	TRINITY_DN30000_c1_g3_i9	CG5915 protein (*Drosophila melanogaster*)	O76742	*E* = 9e-125; bits = 371	87
Light	TRINITY_DN31345_c1_g2_i1	LD33620p (*Drosophila melanogaster*)	Q7PL76	*E* = 0.0; bits = 1113	67
Idlcp (Exocytocis)	TRINITY_DN46925_c0_g1_i1	Inner dynein arm light chain, axonemal (*Drosophila melanogaster*)	Q9VGG6	*E* = 1e-164; bits = 463	90
**Antiviral RNAi**					
SRRT = Ars2	TRINITY_DN31881_c2_g1_i5	Serrate RNA effector molecule homolog (*Drosophila melanogaster*)	Q9V9K7	*E* = 0.0; bits = 1823	94
CG4572	TRINITY_DN33767_c1_g1_i2	Carboxypeptidase (*Drosophila melanogaster*)	Q9VDT5	*E* = 0.0; bits = 749	73
Egghead	TRINITY_DN32129_c1_g1_i5	Beta-1,4-mannosyltransferase egh (*Drosophila melanogaster*)	O01346	*E* = 0.0; bits = 863	94
ninaC	TRINITY_DN26176_c0_g1_i5	Neither inactivation nor after potential protein C (*Drosophila melanogaster*)	P10676	*E* = 0.0; bits = 1894	83
**Nucleases**					
Snipper	TRINITY_DN31391_c0_g1_i1	LD16074p (*Drosophila melanogaster*)	Q95RQ4	*E* = 7e-128; bits = 388	65
Nibbler	TRINITY_DN29782_c2_g2_i1	Exonuclease mut-7 homolog (*Drosophila melanogaster*)	Q9VIF1	*E* = 2e-152; bits = 475	44
**Lipid metabolism**					
Saposin receptor	TRINITY_DN32577_c3_g2_i1	Saposin-related, isoform B (*Drosophila melanogaster*)	Q8IMH4	*E* = 0.0; bits = 1021	58

**FIGURE 3:**
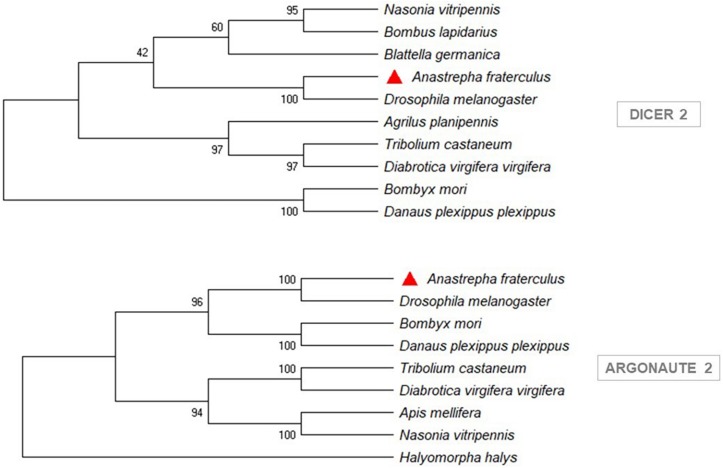
Phylogenetic trees of siRNA pathway genes, *Dicer 2* (*Dcr-2*) and *Argonaute 2* (*Ago-2*). MEGA X was used to construct the phylogenetic trees with Neighbor-Joining method. *Anastrepha fraterculus* sequence from transcriptome was marked with a red triangle. All accession numbers are shown in Supplementary Table S4.

**FIGURE 4:**
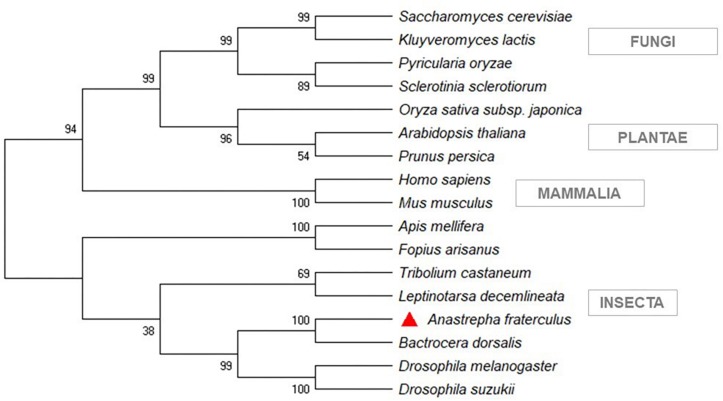
Phylogenetic tree of target gene of silencing, *V-ATPase*. MEGA X was used to construct the phylogenetic tree with Neighbor-Joining method. *Anastrepha fraterculus* sequence from transcriptome was marked with a red triangle. All accession numbers are shown in Supplementary Table S4.

The sequences of *Rhagoletis zephyria*, *B. dorsalis*, and *C. capitata*, from a BLASTp similarity search, showed the closest similarity to *A. fraterculus* (Supplementary Material S4). The phylogenetic analysis showed that the siRNA pathway gene sequences (*Dcr-2* and *Ago-2*) from *A. fraterculus* transcriptome were classified in the same clade of *D. melanogaster* (Figure 3) and the *V-ATPase* sequence in the same of *B. dorsalis* clade (Figure 4). The *V-ATPase* sequence was grouped only with insect sequences, indicating the dsRNA sequence specificity.

### Gene Silencing and Mortality in Larval Stages Induced by dsRNA Soaking

**FIGURE 5:**
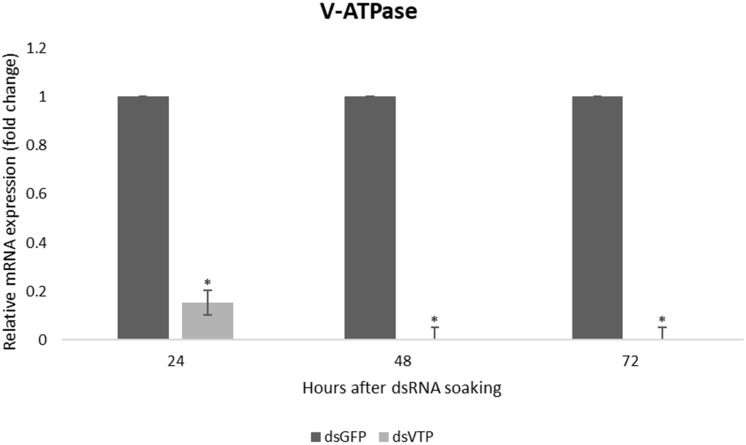
Relative mRNA expression of *V-ATPase* in *Anastrepha fraterculus* larvae after 24, 48, and 72 h of soaking in dsRNA (500 ng/μl). The mRNA levels were normalized using *α-tubulin* and *actin* as reference genes. The columns represent the mean ± SE (*n* = 3). ^*^p ≤ 0.05 (*t*-test).

**FIGURE 6:**
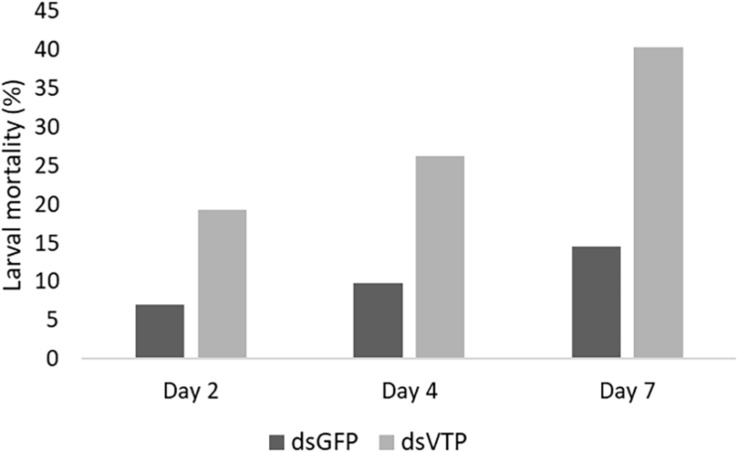
Mortality cumulative of *Anastrepha fraterculus* larvae (*n* = 57) after soaking in dsRNA solution (500 ng/μl) from *V-ATPase* (ds*VTP*) and *GFP* control (ds*GFP*) at 2, 4, and 7 days.

Larvae of *A. fraterculus* soaked in a concentration of 500 ng/μl of dsVTP showed a robust gene silencing as early as 24 h after exposure to dsRNA. The dsVTP soaking resulted in an 85% knockdown relative to ds*GFP* control and this increased to 100% after 48 h (Figure 5). The silencing effect persisted up to 72 h (*p* ≤ 0.05). The mortality of *A. fraterculus* was evaluated for a period of 7 days when larvae reached the pupal stage. Larval mortality started at one day post-soaking (dps), with 5% mortality in larvae soaked with dsVTP. The mortality induced by dsVTP became evident at 2 dps (19%) and rose further to 40% at 7 dps (Figure 6). While the mortality in larvae soaked with ds*GFP* (control) was 14% at 7 dps.

### Expression of siRNA Pathway Genes *Dcr-2* and *Ago-2* dsRNA

**FIGURE 7:**
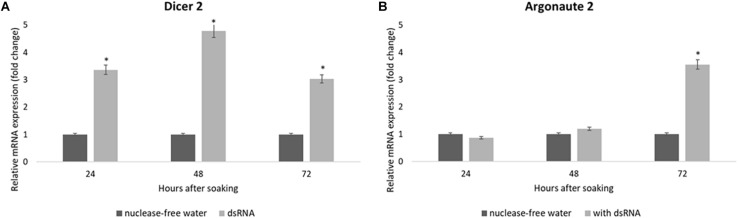
Relative mRNA expression of *Dicer-2* **(A)** and *Argonaute-2* **(B)** in *Anastrepha fraterculus* larvae in response to dsGFP soaking after 24, 48, and 72 h (500 ng/μl). Nuclease-free water was used as control. The mRNA levels were normalized using *α-tubulin* and *actin* as reference genes. The columns represent the mean ± SE (*n* = 3). ^*^p ≤ 0.05 (*t*-test).

The expression of the siRNA genes after the dsRNA soaking in the SA fruit fly larvae confirmed the robust response of the *V-ATPase* gene. The *Dcr-2* mRNA levels were upregulated in the first 24 h after the dsRNA soaking and increased after 48 h; at that moment the *V-ATPase* mRNA levels were completely downregulated (Figure 7A). The *Ago-2* mRNA levels needed a long time to show upregulation: the *Ago-2* upregulation was significant at 72 h after soaking (Figure 7B).

### dsRNA Degradation in *A. fraterculus* Larvae

We checked the degradation of ds*GFP* by the dsRNases present in the body fluids (hemolymph) from *A. fraterculus* larvae. After 1 and 2 h of incubation, no significant degradation of dsRNA was observed (Supplementary Material S3). However, after a longer incubation of 4 h, approximately 40% of the body fluid band intensity was reduced when compared with the initial incubation (0 h).

## Discussion

Although *A. fraterculus* is one of the main pests of fruit crops in the American continent, the lack of genetic information is still an obstacle to unraveling molecular mechanisms of this pest insect. The analysis of the insect transcriptome allows the identification of genes that can be used for pest control through different molecular approaches (Sagri et al., 2014; Garcia et al., 2017). Several studies in the context to develop RNAi for the control of fruit flies species were conducted so far, but only for *Anastrepha suspensa* (Schetelig et al., 2012), *B. dorsalis* (Chen et al., 2008, 2011; Suganya et al., 2010, 2011; Li et al., 2011, 2016; Zheng et al., 2012, 2015; Shen et al., 2013; Liu et al., 2015; Peng et al., 2015; Xie et al., 2017), *Bactrocera minax* (Xiong et al., 2016), and *C. capitata* (Gabrieli et al., 2016). With this project, more than 84,000 new entries related to *A. fraterculus* are now available. We also provide a database of 143 novel potential target genes.

The Diamond search analysis showed the greatest quantity of non-significant hits, which indicates that the *A. fraterculus* transcriptome contains unknown sequences that are not described in the protein sequences databases. Thus, the *A. fraterculus* transcriptome was screened for the presence of genes related to the RNAi machinery and for further exploration of essential genes to be silenced as target gene by RNAi technology. Similarity searches were performed using as reference the *D. melanogaster* sequences because it is the species with the complete genome sequenced and fully annotated (Adams et al., 2000) and phylogenetically more closely related to *A. fraterculus*. This is the first study showing evidence of a functional RNAi mechanism in the SA fruit fly.

### Novel Target Genes Found in *A. fraterculus* Transcriptome

The target genes selected are involved in post-embryonic growth/development and reproduction. Fruit fly pests cause direct damage to production by the puncture for oviposition and the larval development inside the fruit (Aluja, 1994). Thus, the use of RNAi techniques in insect post-embryonic development is crucial for crops protection. Genes involved in the formation of posterior organs during the larval stage, for instance, the ovipositor, are very interesting for RNAi studies. Examples of genes involved in the formation of the posterior organs found in the SA fruit fly transcriptome are *hedgehog* (*hh*), *homeobox protein abdominal-A* (*abd-A*) and *homeobox protein abdominal-B* (*abd-B*). These genes are part of a developmental regulatory system and provide cells with specific positional identities on the anterior-posterior axis (Celniker et al., 1990).

Genes involved in reproductive events such as oviposition regulation can also be screened in the *A. fraterculus* database. The *sex peptide receptor* (*spr*), for example, is a gene involved in the suppression of mating receptivity and induces the egg laying (Yapici et al., 2008). These genes can be studied for dsRNA delivery sequentially or dsRNA-concatemerized.

### Three Pathways of the RNAi in SA Fruit Fly

In insects, three RNAi pathways can be distinguished: miRNA, siRNA, and piRNA, based on the Dicers (Dcr) or Argonautes (Ago) enzymes and related small RNAs. Thus, the miRNA pathway includes nuclear Dicer (Drosha/Pasha), cytoplasmic Dicer (Dcr-1/Loquacious), and Ago-1 as core enzymes. The siRNA pathway is activated by exogenous dsRNA and involves Dcr-2/R2D2 and Ago-2. The piRNA is another pathway involved in the defense against transposable elements and includes Ago proteins (Aubergine/Ago-3), which is independent of Dcr (Taning et al., 2016). Sequences representing all core RNAi genes were identified in the *A. fraterculus* transcriptome with a bitscore ≥ 150 and E-value ≤ 1e-5. The domains of the Drosha and Dcr proteins were conserved in *A. fraterculus* (Supplementary Material S2). We found some *Dcr* domains as following: amino-terminal DExH-box helicase domains, PAZ domain, two RNaseIII domains, and carboxy-terminal dsRNA-binding domain (dsRBD) (Carmell and Hannon, 2004). However, some components of the *Dcr* family differ from this general arrangement; for instance, some of these lack a functional helicase domain or a PAZ domain, or the number of dsRBD is ranging from zero to two (Macrae et al., 2006). The sequence of Dcr-2 in *A. fraterculus* does not show a dsRBD domain.

Unlike Dcr, the PAZ and amino-terminal DExH-box helicase domains are not present in Drosha. Two cofactors with the conserved domains DSRM, Pasha, and Loquacious, were also identified in *A. fraterculus*. These proteins are needed to interact with the RNase III, Drosha, and Dcr-1, respectively (Carmell and Hannon, 2004). For R2D2, we found sequences inside the defined threshold, but without conserved domains. R2D2 can form the Dcr-2/R2D2 complex and bind to siRNA to enhance sequence-specific messenger RNA degradation mediated by the RNA-induced silencing complex (RISC).

The Ago superfamily is divided into Ago and Piwi clades. *Drosophila* species have two Ago members (Ago-1 and Ago-2) and three Piwi members (Piwi, Aubergine, and Ago-3) (Cerutti et al., 2000; Cox et al., 2000). In these insects, Ago-2 is involved in the siRNA-directed mRNA cleavage, and Ago-1 mainly mediates miRNA-directed translational inhibition. Some Argonaute proteins can cleave the target mRNA, while others affect their nucleic acid targets using alternative mechanisms (Ketting, 2011). Two domains characterize ago proteins: PAZ and C-terminal Piwi (Cerutti et al., 2000). In the *A. fraterculus* transcriptome, we have identified the five members of the Ago protein superfamily, with the PAZ and Piwi conserved domains.

The third pathway of RNAi, the piRNA, involves the proteins Aubergine, Ago-3, Piwi, and Zucchini (Hartig et al., 2007). Zucchini is an endoribonuclease that has a role in piRNA maturation. With the absence of this protein, the transposons are no longer repressed and no piRNAs can be detectable (Pane et al., 2007). In *A. fraterculus* we found sequences of Zucchini protein with the presence of conserved domains superfamily PLD (Phospholipase D).

### Duplication and Loss of the RNAi-Related Genes in *A. fraterculus*

The biogenesis of the RNAi pathways and related-proteins is similar among eukaryotes, however, throughout evolution duplications and losses of genes have occurred in several insects. Duplications or loss of RNAi-related genes can lead to higher or lower functionalization of the RNAi mechanisms and could explain differences in the efficacy of RNAi in different insect groups (Dowling et al., 2016).

Our transcriptome analysis indicated gene duplication and gene loss events in *A. fraterculus*. Possible duplicates of *Drosha*, *Ago-2* and *R2D2* were found in the SA fruit fly transcriptome compared to *D. melanogaster*. Dowling et al. (2016) also found possible duplicates of *Ago-2* in transcriptomes of other order insects, as *Peruphasma schultei* (Phasmatodea), *Prorhinotermes simplex* (Isoptera) and *Pseudomallada prasinus* (Neuroptera). These authors suggested that *Ago-2* was present in two copies in the last common ancestor of insects. Is it possible that SA fruit fly has three copies to *Dcr-2*, while *D. melanogaster* has only one copy for *Dcr-2*? It is known that insects inherited a complete RNAi system from their common ancestor and diversified and expanded this original system (Dowling et al., 2016). One example of this is the piRNA pathway (*Piwi*/*Aub*) in insects that acts as a defense against transposons in the germ line. In the *A. fraterculus* transcriptome of this study, this gene is present with two copies, while Hemiptera species as *Acyrthosiphon pisum* has eight copies for this piRNA gene. Dowling et al. (2016) considered that homologs of both *Piwi/Aub* and *Ago-3* could be present in the last common ancestor of insects in multiple copies. Although we have used a mix of all developmental stages of SA fruit fly to generate a comprehensive transcriptome, it must be remarked that the final conclusion that a gene is lost from a species cannot be made since the gene in question may not have been expressed or very lowly expressed (Dowling et al., 2016).

### SA Fruit Fly Has Auxiliary Factors (From RISC)

We found 19 intracellular factors that are associated with the activity of the RISC. In the RISC assembly for exogenous dsRNA in *D. melanogaster*, the siRNA duplex is transferred from complex B to the RISC-loading complex (RLC), including *Dcr-2* and *R2D2*. Next, *C3PO* (*translin* and *TRAX*) binds to the RLC and the RISC [consisting of the *Dcr-1*, *Tudor-Staphylococcal nuclease* (*Tudor-SN*), *vasa intronic gene* (*VIG*), *FMR*, and *Ago-2* subunits] to produce the holoRISC (Jaendling and McFarlane, 2010). These sequences were found in our *A. fraterculus* transcriptome, holding conserved main domains and with an identity between 49 and 82% compared to *D. melanogaster* (Supplementary Material S2).

The nucleases involved in piRNA biogenesis, Armitage and Homeless (spindle-E), showed long sequences (>4,000 nc) in *A. fraterculus*, while small fragments represented *Maelstrom*. Genes that encode *Gawky*, an RNAi effector, *Staufen*, an RNA-binding protein, *Elp-1*, a component of the core elongator complex involved in the RNAi, and *Clp-1*, a kinase that can phosphorylate siRNAs, as well the *RNA helicases Rm62* and *Belle* also showed long sequences (Findley, 2003; Vagin et al., 2006). The DEAD-box RNA helicase *Belle* has a function in the endo-siRNA pathway, interacting with *Ago-2* and endo-siRNA-producing loci and is localized in condensing chromosomes in a *Dcr-2*- and *Ago-2*-dependent way (Cauchi et al., 2008). Another, the DEAD-box RNA helicase *PRP16* has a key role in the pre-mRNA splicing and was found in the *A. fraterculus* transcriptome with an identity of 93% as compared to *Drosophila* sequences (Ansari and Schwer, 1995).

### dsRNA Uptake Genes

With the exception of *SID-1*, all dsRNA uptake components were found in the *A. fraterculus* transcriptome. This confirms the idea that this gene is absent in Diptera. Unlike the typical model organism of *C. elegans*, which uses SID-1 to transport dsRNA into the cells, *Drosophila* species do not have SID-1 orthologues (Huvenne and Smagghe, 2010), therefore two scavenger receptors, namely SR-CI and Eater, were proven to undertake the transport function in *Drosophila* (Ulvila et al., 2006). Scavenger receptors play a role in phagocytosis and act for large molecules and microbes (Prentice et al., 2015). In *A. fraterculus*, scavenger receptors were found only for Eater and *SR-CI* sequences; this last one with conserved domains (Supplementary Material S2). Other genes coding for proteins involved in endocytosis were found in *A. fraterculus*, including *HPS4* (*Hermansky-Pudlak Syndrome 4*), a factor involved in the regulation of the combination of late endosomes and RNA-processing GW bodies, *FBX011* (F-box motif, Beta-helix motif), a regulator of endosome trafficking and the *clathrin heavy chain* (*chc*), which is needed for clathrin-mediated endocytosis (Swevers et al., 2013).

### Nucleases in SA Fruit Fly Development Transcriptome

Nucleases sequences were identified only for Snipper, a histone involved in mRNA metabolism, siRNA degradation, and apoptosis, and for the Nibbler, a nuclease described in *Drosophila* and involved in the processing of 3′ends of miRNAs (Swevers et al., 2013). We identified the conserved domains ERI-1 3′ exoribonuclease for Snipper sequences in our *A. fraterculus* transcriptome (Supplementary Material S2).

### Presence of Genes Involved in RNAi Efficacy

We found five intracellular transport components as classified by Yoon et al. (2016). The components *Vha16* (*Vacuolar H+ ATPase 16kD subunit 1*) and *VhaSFD* (*Vacuolar [+] ATPase SFD subunit*) related to proton transport, *Rab7* (*Small Rab GTPases*) involved in endocytosis process, *Light* involved in lysosomal transport, and *Idlcp* involved in exocytosis process.

Four antiviral RNAi were found in the *A. fraterculus* transcriptome, *Ars2*, a regulator involved in innate immunity via the siRNAs processing machinery by restricting the viral RNA production, *CG4572*, a protease implicated in systemic silencing and antiviral RNAi, *Egghead* (e*gh*), a seven-transmembrane-domain glycosyltransferase with innate immunity against RNA virus, and *ninaC*, a protein involved in vesicle transport. All antiviral RNAi components were identified with conserved main domains (Supplementary Material S2).

The *Saposin* receptor, which is involved in lipid metabolism, was identified with Saposin A and Saposin B conserved domains in *A. fraterculus* (Supplementary Material S2). Saposin is a small lysosomal protein that serves as the activator of various lysosomal lipid-degrading enzymes (Darmoise et al., 2010).

### Evidence for the Sensitivity of Larval Stages of *A. fraterculus* to RNAi

The functionality of the RNAi mechanism in *A. fraterculus* was demonstrated using a dsRNA targeting *V-ATPase*, evaluated by an *in-house* soaking bioassay. *V-ATPases* are ubiquitous holoenzymes among eukaryotes (Finbow and Harrison, 1997). These enzymes are composed of two subcomplexes, the cytosolic V1-domain, where ATP binding and hydrolysis take place, and a transmembranous V0-domain, through which protons are translocated (Vitavska et al., 2003). The *V-ATPase* sequence analyzed in *A. fraterculus* belongs to the V0-domain (Supplementary Material S2). The *V-ATPases* uses the energy produced from ATP hydrolysis to transport protons across intracellular and plasma membranes of eukaryotic cells (Nelson et al., 2000). Although the V0 complex plays a key role in protons translocation, just a few studies aiming V0-domain as the target were published with insects (Ahmed, 2016). Therefore, we synthesized a dsRNA fragment with 483 bp length targeting *V-ATPase V0*-domain gene.

The results presented here indicate that *A. fraterculus* has a sensitivity to RNAi. We demonstrated that a small dose of dsRNA (500 ng) administered by soaking for 30 min could produce significant RNAi responses (target-gene knockdown and mortality). We attributed the high knockdown efficiency in *A. fraterculus* to some factors confirmed in this study by transcriptome analysis and dsRNA delivery assay. These factors confirm the presence of RNAi machinery genes, the activation of siRNA pathway genes (especially *Dcr-2* upregulation at 48 h after dsRNA delivery), few nucleases, and factors related to uptake, that need to be clarified.

The effective response of gene silencing as showed by *A. fraterculus* at 48 h after dsRNA soaking resulted in mortality of these larvae. The *V-ATPase* sequence from the *A. fraterculus* transcriptome contains the VMA21, a short domain that has two transmembrane helices (Supplementary Material S2). The product of the VMA21 is characterized by an 8.5 kDa integral membrane and contains a C-terminal di-lysine motif that is needed for retention in the endoplasmic reticulum, and disruption of the gene causes failure to assemble a stable V0, rapid turnover of *Vph1p* subunit (that contains charged residues that are essential for proton translocation) and consequent loss of *V-ATPase* function (Hill and Stevens, 1994). In other dipterans species, the *V-ATPase* knockdown responses were variable. In *B. dorsalis*, the ingestion of 2000 ng *V-ATPase D* (V1-domain) dsRNA through diet caused only 35% of gene silencing after 4 days (Li et al., 2011). The neonate larvae of *D. melanogaster* when soaked in 500 ng of *V-ATPase E* (V1-domain) dsRNA caused a decrease of 49% in gene expression and feeding larvae caused 56% knockdown and 70% mortality (Whyard et al., 2009). These studies suggest that the silencing of *V-ATPase* subunits demonstrate variable results related to subunit and target species.

### *Dcr-2* and *Ago-2* Respond to dsRNA Exposure

The upregulation of the Dcr-2 at 24 h after the dsRNA soaking demonstrated that the RNAi response in *A. fraterculus* is active. The Dcr-2 is a specific ribonuclease that initiates RNAi by cleaving dsRNA substrates into small fragments (Macrae et al., 2006). The PAZ and RNase III domains from Dcr-2 found in our *A. fraterculus* transcriptome are shown in the Supplementary Material S2.

### dsRNA Is Degraded in *A. fraterculus* Body Fluid

Degradation of ds*GFP* (0.5 mg/ml) was observed only after 4 h of incubation. Liu et al. (2012) verified ds*GFP* degradation only after 3 h of incubation using hemolymph of *Bombyx mori* larvae. On the other hand, the authors verified that ds*GFP* degradation in gut juice occurred at less than 10 min. Christiaens et al. (2014) demonstrated an intense dsRNA degradation shortly after 1 h in aphid hemolymph (*A. pisum*).

According to Singh et al. (2017), usually, a high concentration of body fluid from dipteran insects is required to degrade dsRNA. For *A. suspensa*, for example, Singh et al. (2017) showed that 4.44 mg/ml of body fluid was needed to degrade 50% of dsRNA, while for *Spodoptera frugiperda* a very low concentration of hemolymph (0.11 mg/ml) was enough to degrade dsRNA within an hour. Singh et al. (2017) also suggested that the expression of dsRNases could be lower in Diptera species when compared to other orders. This was noted in the present work in which only a nuclease (Snipper) involved in the siRNA degradation could be identified based on the lists previously reported (Swevers et al., 2013; Prentice et al., 2015; Yoon et al., 2016). Recently, Prentice et al. (2019) confirmed the impact of one specific ribonuclease in the gut of the African sweet potato weevil (SPW), *Cylas puncticollis.* Two nucleases were identified by transcriptome analysis and they were demonstrated to affect the dsRNA stability in the gut when dsRNA was delivered by oral feeding.

## Conclusion

Our project made available more than 84,000 new entries related to the developmental of *A. fraterculus* and generated a database of 143 novel and different target genes to dsRNA bioassays. This transcriptome database is a handy tool for research on the SA fruit fly, especially in studies with a focus on RNAi. The identification of the RNAi machinery genes combined with dsRNA soaking, siRNA genes expression and dsRNA degradation bioassays demonstrated that an RNAi response is active in *A. fraterculus*. The presence of RNAi machinery and efficient genes for silencing confirms the sensitivity *A. fraterculus* to produce a robust RNAi response.

Interestingly, we demonstrated that soaking of the larval stages in ds*V-ATPase* lead to a strong gene-silencing and this concurred with strong mortality of 40%. This assay by soaking demonstrates that dsRNA delivery can also be effected via the cuticle of the insect (environmental RNAi) (Niu et al., 2018). Our data demonstrated the existence of a functional RNAi machinery in *A. fraterculus* and an easy and robust physiological bioassay with the larval stages that can be used for *in vivo* selection of target genes for RNAi-based control of fruit fly pests.

## Data Availability

All datasets for this study are included in the manuscript and the Supplementary Files.

## Author Contributions

ND, DC, GS, and MZ contributed the conception and design of the study. ND and FK organized the database. ND performed the statistical analysis and wrote the first draft of the manuscript. ND, LR, DN, GS, and MZ wrote the sections of the manuscript. All authors contributed to the manuscript revision, read and approved the final version of the manuscript.

## Conflict of Interest Statement

The authors declare that the research was conducted in the absence of any commercial or financial relationships that could be construed as a potential conflict of interest.

## Footnotes

1 http://www.usadellab.org/cms/?page=trimmomatic

2 http://www.bioinformatics.babraham.ac.uk/projects/fastqc

3 http://trinityrnaseq.sourceforge.net/

4 http://www.ebi.ac.uk/QuickGO/GAnnotation

5 http://primer3.ut.ee/
