# Supplementary material for: The South American Fruit Fly: An Important Pest Insect With RNAi-Sensitive Larval Stages
Source: Front Physiol. 2019 Jun 27;10:794. doi: 10.3389/fphys.2019.00794 (PMC6610499; doi:10.3389/fphys.2019.00794)

Supplementary Material

**RNAi machinery and efficacy genes**

**Sequences of *Anastrepha fraterculus*: Comparasion with *Drosophila* or Tephritidae species**

**Dicer-1**

**>TRINITY_DN33861_c2_g1_i1 len=8068**

TCACGAGCCAGTGCGGATCCGAGAAGATATGGTTGTTGTTTTTGTTGCAGCAGCGAAAAT

ATTCCCCATATATGTACGGGCGGTGCTCCTGGAGTGACAGTCCGACCAATATAGTAAATC

GTTCCGTTAATGTAGAACCGACTGTCGGGGGAACTTTTTGGTCGCTTGTCATGCCTATGG

ATTATTCACGACGGCAAACGGTCTACGACAGCAAATATCGAACTATCGGGATTTTTGAAC

AATCGAGAAAATCGACTAAAACATTTAGGACTCTGCCAACACTAGCAGACACTTGTCAAA

AGGGAAATCTTTTTTTCTGCCTACGCATACAAAGATGGTTCTGCTGGATAATTCTTCGTT

CATAATTCGATTAGAAAAGTTCGCAAATTCTGCGAAGAAGGATTCTTCATTCACGGTGAC

GTTTAAACGCTACAATGGCCACGAGCGACCAAAGCCGCGCGAAGGGCGACCAGCGCTACC

CGAACCAGAAACGTACTTGTGCCTTATGCGTGCACAGATGAAATCAAAGAAGATATCCAC

AATTGTGCGTCAGGAAGATGTACCCAAAATGATGAGCATGTACGCGCAATTCATGAAGAG

CAACATGGATGGTCTAAAGCGTGTCAAAAAAGTGAAGAGCAAAGCGAAGGCGGCAAAGGG

ATAAACGCTGTCAACTCATCTGTTTAGGACATAATTTAGCTTTTAAAGAACCTTCTTGTT

TGCCTATAAATTGCAAGTCAAAGTAAAACAGGACAATATTTGTCAATGTCCGCTATACCC

CACTGCAAGTGAAGGACAATATAAACAATAAACATAGTTCTTTTAAATTGTGTAAAAATG

CATATTGAAAAACCTATATTTGCACAGTAACGTTAGTCGCATGCACATACACGTATTCTT

CTTAATTAAAATATTGAAAAATTTCTCATTACAAAATCTGCTTACAAATTAAAATATTGC

CTTAAATAGGTAATAAGTAGATATATTTAATAGGGGCCGAAATCACAACACGGCTTAGTC

CTTCTTTATCAAACCTTGCTTCTTTAATTGACGTAATGCGCATTTAGCTGCCGTGCATTT

CGCTATACGATAGTTACGGCCAATACCACGGAATGTGCCTTTGCAGAAGACGTCAACGGT

GACGCGCACCCGACGCCCATCAGCCAGTTTCTCCGGTTTTCCAAATTTGGCGGTCTCCGG

TTCCAGTTCCAGCAATTCTCTAATAGGGGATTTTGGCACTGAGTTGCTAAATTGTTCTAT

CTCTGGCTTCATCATATTACTATACACCTGCCATACAACGTCCAAAGACATATTGGAGTC

CAGGAAGATGGCGCCTGCAATTGACTCGAACACATCACCCAAAGCTTTTGGCACTTCAAC

ATCTTCAGCATCGTCGCACTCTTCCTCCGAGAGTAGATAATATTCTTCGCTTATGCTGTG

ACCGTTTTCGTTCTGTATGCGTACAAAACGATCGATGACTTCATTTAAACCCGGCGATAG

ATGTCGAAAATATTTGTGGAAACCATGTCGCACAGCCAGTGACGCAAATATAGTATTATT

TACTAACGCCGAGCGCAAGTCGGTCAACGCACCCGGCGAATGTTGCCTGGGATCTTCGTA

AAGATGACGAGTAATCAGATAATCCAACACAGCATCACCCAGGAACTCTAACCGTTGGTA

GCAATCTGTCAAACGATTTGGCGAGTAGCTGGCATGGGTCATGGCTTGCAGTAGATAGGA

ACGATCACGAAATTTATAACCCAATGTATTCTCAAACTCATCGAAACCCTCCAACATAGA

CTCCAGCTCCTGTGGGGCGTTGGGCGAATAGTGGAGCAGTGGACTTTTTGGCGCTGTCCA

CTCGCCATATACAGTTATTTCACCATTCACATCCGGATTAGAGCTGCCCGGTATACGGGG

CTCTTGTGGATTATATGGTTTGCGTACGAACGGAAGCACACGTATGCCAAGCCACGCCAT

AAACAACAGAGCGCCACGCGGACCGCATTCGATGAGATATGCGCCGATTAACGCCTCAAC

ACAATCGGCAACCGATTTATCTGGAATGCTGTGTTGAGTCACAAGATTGTAAGGAATAAA

GCAGGAAAAGTCATTGCCTTCAGCGATTTCATCGACAGCGCTAGCATCCTCGCTGCAATT

GTATTCATCACCGAAGAGTCCTAATTTGTCGGCCTTTTCACGCACCAATGCGCATATCTC

GGCGCTGTTCAACTTTTTAATGTTAGCCAAATCGACCAGCTTCCAGTGATGCGGCGGTAT

CTTTGCCTCGATCAGTTCCTTCTCAAGCTCTTTGGGCACGTAGTAGCAGGGCGGCAACCA

ATTGTCGTGTGGCTCGAATTTCGTCGCTATCATATATTCGCCCAGCTTTTTCCGGCGCCC

CAGTCGATATAGATTCAAGTTGGCCACTTGTTTCGATCTCAAATGACTTAGCTTGCCTTC

ATGCACGTTTTCGTACGTTATGTACAAATAAGTGGTAATCGCATATTTCAGAAATGAGTC

GCCAATCGTCTCCAGACGTTCTAAATTGATGCCATCATTGGCATTCGACATTGTTAAACC

TTGCAGAATGATGCTTGGGCTAGGTCCTGGGTGGCCAGCTAAATCCGGTTGGCGGTCGAA

GCTAAACGTATTGCCTTGCATGTTTTTACTCTCCTGTTTTACATCTATTAAGCTAACGCA

GCCTGTTGCACCTGCTTTTCTCTCAAAATTCAACTTAATCAGCTTCTTATAACCTTCACC

CCAACCCTCCAACTTCAACATGTCCTTATCATTGAAATTATCAAAAATATCACCACAACC

TTGAATTTCGAATAGCTCCAATGGATGCGATTTCAAATAGTCGGCATTTAAACGCGCAAT

ATCGGCAATGGTCAGCACATTTGCCTGCTTGCTCAAAATAGACATTAGCACATCTTTATC

AGCATAGGGCAAAAGTTTCAAAAACGCTTCAGTAGTTGCCGCCTCGCCGTGTGCAGCCGA

CTCGTTTGTGGTGCGCTCTTTACGTGCCAACATCACTGGCTGGTTGTATTTCACAAGCAT

ACCGGACTGTTCAATGTTCGCTTTCAGTAGGCGTGTAGATGCAGTGAACTTGGCAATGGA

GTCGCACAATTCTGCTTCTATATTCGGTTTTAAGTCCTCAAAGTTATAGCCCACAAGCAG

GTTTTTGGTTTTCTGATAATTGCGCTCATTTGCATTGGTCGCTTGAATGATGGACAGTTT

TTTATTGCGCTTTTCGATCTCCTGATCGGTTTCGATGGTTTCAGCCTCATTTCTAGAGGT

GAAGGCTATACGCAAAGGACCACACTCATCATCTTCATCCTCACTATAGTTGGTTGGCTC

ATTACCATCGTAACTGGATGCGAATGAGTCGTCGGAGTCGTAATAGGCAAATGAATTTTT

GCGAAAATTGTTCGTTTCGGTGGTGTTTTTGCTTTGCGCACAATCGTCCGGTTTTTTATT

TAGTTGGGATTCCTTAACATCCCAGAACGTGGGCGAGCCATAACGCATCTGCTGGTCGCA

AAAACTGACATTCGATGGAAAATATTTCAAACATTCCTCCTCATCGTCGTCTAATGAGTC

CGAGTATTTATTAAAATTGATATCATTCGCCATGTCATTGGACCAGGTGCCTATTTCTAT

AAAATTACTATCCTTCAATTTCTTTTCGCCTTCTATAATAATTTCGTTTGCGCTCTTTGC

TTTAGTTTGATTATCTTCTGCGCAAAATTCACCATTGACGCCAATATCTTTCGCAAAACC

GTTACTTTGTTTGTTATTTGCCTCCTTTTCCTTTTGTTCTTCCGTTTTCTTGTCCAACGA

CGCATTATTTTCTTTTGATCGCTTTAGCACCTCCGACAAACTCCAGCCAAAGTCCAGCAT

TGGCCACTCAAAGTCGTCGGGAATTTCTTGCTGTCCCAGCCCTATATCCAACGACACCTT

CTTGCGTATGTCATCCGCTAACAGCAATCCGTTTATGCGGTACAGAATGCAGGGCAAGCA

AACAGCAGTGCGCCACAGTGAGGCCGGAAATGGATGTACGGTGCACAGTTCAGGCACAAG

AATTTGCTTTTGTTCGAGGTTCTCACGTTTGGCACGTTTCGTTTCTTCCGAGCTGGTGGG

CAAGGCAACTCCTTTTCGATTTACATAACGTGGCGTTAGGAAATTCAAACGCGCACTCGT

GTGATCCACATCCAACAGTGGCTGTTTCACATTCTGTATGGTCAGTCCGTATTTGAGGAA

ATAGTAATGCTTAAATGTGCGGTAGTTTTCGCCAGGAAAACAACTTTGCGGTGATAACTG

CGGACAAATCTCTGCCACGTAAAAGTATTGCGGCTGATCTTGATTGCGATACCAAGGCAT

AACGACGGCATCCTTGAACTTGTTAGCATCAAAATCTATGGATTTGCGCATGTCTTCTGG

CACTGGAGTTGGCATCATACTTGCATTTCGTTCAATTAACTCAAGGAAGTCCCAGTCGAT

CACCTTGCCGCCATCTCGCTTCACTGTCGGCACTATGAATACACAATTTTCCGTGGAGTC

GGGATCAAAGAGCATAAGGAACTTCTGCAGACGCAATACGTTAGTGAATGTATAATTGAG

GAATGTATTGATGCAGCGTATTTGTGTTTCGGTGAGCACCACCCGCTCTTTCGCGAGCAC

AATGGAAACTTTTACCTCGCCGGAACGTGTAAATATAGGAAATGAACTGACTTTTGGTAT

ACGCTTTAAGGTAAGTATACCAAAACCTTGTTGCGCATCTTCAGGCGGGTATATTTTACG

GCCACGTGTATTTTGCTCCTCTGGAATAGGGCACTGGAGAGTCAAGTCGATTAAATACAG

ATAGCAAGGAACACCGGCTGTCGGACGACAATCACTAAACTCCGAGGCAATTCGCTTATA

GTAATACTGTCTGCGTTTTGTAGTGCCTGGACGCGGCTCAGAGTTTTCAAGTACGATCTT

GTCATCTTCCTCCTCCAAGTCAAAGTTCTCCCAATCGGGTTCCATAGCACGGAATCCCTC

TTTTCCTATTGGCAGCAGGTTGTCGTCCAGCTCGCCAGTTTTATGCAGCTCTATGCATGC

CTGTAATGCGGCCATTCTACGTGCCAATATATTCGAAGACATTGGAAGCCCAACTATATC

ATATTTTAATGGCGAATTTATCGGAAGACGTATTGTGTATTGGTACATTTCGACACCACA

TCGAATGGTTTTTGTGCAACGCCAGAGCGCAGTGAGTTTTGTAAATGTGTCGCTTGGCAA

CTTGGAGCAGTATTTGTTGACTAACGCGATGGATGAAGTGAGATCCACAGAAGCGCCTGT

CAGCAGATGTTCGCAGGGTTTGTAGCGCTTCACGCATTTGTTGAAATAGTCCGCCAGTTG

GTGTTCTGCTTCCGATGGCTCAGTATTAGCACATTTTTGTAGAAGCATCTTCTCTATTTC

CCGATACTCTGCCAATTGCTCCACTATTTTATTAGTTGTGAATTCTATTTGGTTGATAGT

AGCATCGAGGCCGGTTTCCAAACCGTGTTGTGCACAGTTTTCATTTTCACAGATACAGCG

AAACCGACGCTTCTCCTTTTTTTGCTTTGGTGTAACCGATGCTGAAGCCGGTGTTGTTGC

TGTTTCTTCCAATAAAATTCTAGAAGAGTTATTACTGCTTATGTCTGAAATCATGTCGCT

AAGATCGAGCACATCGAAACAATCATGTAGCGACTCGTTGGCCACTGACTTCGTTTTACT

AACTTCCTTTATTGAAGATGTTTCATTTTGCAGTTCCGTAATAGGAGCTAATTCGCTTTC

AATTTCTACTAAATCAATGGTATTGGTATCTTCATTTTCTATGCCTTTAATGGCCGGCAA

TTCGTCCTTGATTTCCTTGAGCGTAATCTCGCATTTTGTTGGTGGCTTACTGGTAATCAC

TTCGGGATTTAATATTTTTACGATTCCCTTTGAAGAACCGATTGTAAATTTGTTACTCTT

GCCATTGCATTCCTCCTCATCGTCGCTACATGAATCCGTATCGATAACGTCGTCCTGACC

AACCTCTAATTTACATAAATACCTATGGCTCTGGTCACTTAACTGTTCATTTTCACAAAG

AATTGCATTATAAGCTGGCGATACTAGTATCACATGGTAAGCAGGTGCTGCTCGCGCACG

CCCTTTACATTGCACATAGCTGCGATATGTTGTAGGTGCATCCCAGCGCACTACTAAATT

ACACTTTGGCAAATCAATGCCCTCCTCTAGCACTGAGGTGCCAATTAAGACATTGCAGTC

GTGCATACGAAACCGTTTCAATACCTCTTCCTGACGCCTATGCTCGACCTCAGCTTCCTT

GGGTTCGGTTATAGGATCAGCGACACGGTCAGCTGTGAACTGGCATTTAAGGAATTTCAG

TTCCGGATCGTGGCGGCTCATCTCGCTCAGTAAATCAAAAAGCACACGTGCCGTGTAATT

GGAGTTACAGAAAATAATAGCGCAGAGCGTATCCAACGCATCACCGTTGTCACGCATTTG

TCGGCGGTTAAAAGGTCGCCGCTTGAAGCGGCCATGTGAACCACCGACACCATGGCGTCG

ATTTGTACTGGCGGAACTTTCCGTTTTGTGCGTTGTGTTCGCTTGCAATGTCTGCTGTTT

TGCCACATCAGTTGTGGGCGATTTTACAATTGGATCTAAATTATCCATAATCGAGCGTGA

TTCGAGTTGTAGTTTTGTAGTCACATTTTCCACATTCTGGCAACTATATTCGATACTTCG

GCTGAGTTTTTGAAAATCCATCTGATCCAAATCCTGGCTCATTTTCTGAATTGTTTCGTT

TTTGGTCAATAATTCTTCCGGCTTGAATAAGCGTAAAATTTCCAGCAAACGAAAAACTTT

AGGGCTCGAATAGCGTTCCACTAATTCTTTATGATTATTAAATTTTTTGAATGTTTGTTC

GCAAATGGCATGAAATTCTAGAAGCGCTGTATTCACAACGCAAAGCAAAATGTAATGGCG

TTCATGTGGCGTTTTTATTTTCTGTTTCTCTATGCGGTGAAACATGTTGACAGCAACACG

ATCTGCACACCACGGGCCCATTTCGTAGAGCACAAAGAGCATGATTTCCAAAAATTCCAT

GGGTTCTTCTTTCGGATCGGGAATATCCTTTAATTCCTCCATGTATTCTTCTGTACAGTA

TATTTCGAAAGGGTCGTAACGGTGATCTATTAAAAACGCTTTTCTCGTGCAAATTACCTC

CTCTAGCACAGCGGTCAACTCATCTCGCGGATATGGCGTGCATTGCACTATATACTCACT

AGGTTTGGCGCAATACCTTAATACTGTTACGATATCACTGGCAGTCTCTGCTTTGCAGTG

CACACTATCCTCCAAAGTATTCAGCATTGCACTGAGCTCTTCTAGCGAGCATTCAGCGCT

GTGAAGTGGTCCAGCTAAGCCCAACACTTTCGGCATGTCTTTGCCGGAAACATTGAAATG

GTCTTGAAAGATTTGACGCAAGTCTCTATATACCTCCGCTTCATGACAGTCTTCCAGTAT

TAACAAATGCACATTCGTCATACTTAGCTTTCCACATCTCAAGGCTGCCAGACAAGTACG

TGGATGAAGTACGTAAAGTTGAAAGTCTTTTGGTATATGAATTTCCTGCTTATCCGCCGC

CCCCGTTCTCTCCTCTTTGTACACTTTCAAGTCAGTCAAGTGCTCTATCATGGTTGTTAT

AGATGGAGCGTTTTGTTCACAGGTCAGGTATAAATTAATTTTTTCCGCGATTCGTGCTGG

TCGTGCCAGCTCCTGAAGGAGCTTTAAGGCAATAAACTCCTTGGATGATTTGTGACCGAG

GCATATCATTACATTACGCTCGAACGCGGCAGATAGCAATTCGATTTGATAATCACGAGG

GGTAAAAATTGTGGTGTGAATGTTATCACACCAGTGGAACGACATTTTGGTGGCATAAAA

CACAACACAATTTGAATTTAAAAACACTAAATTAGTTAAAATCGAGAAAGTCAAACCAGA

GCACAAGTTGGGAAATCACTAAATTTGT

**tBLASTn(First hit)**

Score = 2728 bits (7071), Expect = 0.0, Method: Compositional matrix adjust.

Identities = 1467/2381 (62%), Positives = 1748/2381 (73%), Gaps = 200/2381 (8%)

Frame = -2

Query 1 MAFHWCDNNLHTTVFTPRDFQVELLATAYERNTIICLGHRSSKEFIALKLLQELSRRARR 60

M+FHWCDN +HTT+FTPRD+Q+ELL+ A+ERN +ICLGH+SSKEFIALKLLQEL+R AR

Sbjct 7965 MSFHWCDN-IHTTIFTPRDYQIELLSAAFERNVMICLGHKSSKEFIALKLLQELARPARI 7789

Query 61 HGRVSVYLSCEVGTSTEPCSIYTMLTHLTDLRVWQEQ-------PDMQIPFDHCWTDYHV 113

++++YL+CE SI TM+ HLTDL+V++E+ ++ IP D+ +

Sbjct 7788 AEKINLYLTCEQNAP----SITTMIEHLTDLKVYKEERTGAADKQEIHIP-----KDFQL 7636

Query 114 SILRPEGFlylletrelllssvelivleDCHDSAVYQRIRPLFENHIMPAPPADRPRILG 173

+L P L L +L +++V L++LEDCH++ VY+ +R +F++H D P++LG

Sbjct 7635 YVLHPRTCLAALRCGKLSMTNVHLLILEDCHEAEVYRDLRQIFQDH-FNVSGKDMPKVLG 7459

Query 174 LAGPLHSAGCELQQLSAMLATLEQSVLCQIETASDIVTVLRYCSRPHEYIVQCAPFEMDE 233

LAGPLHSA C L++LSAML TLE SV C+ ETASDIVTVLRYC++P EYIVQC P+ DE

Sbjct 7458 LAGPLHSAECSLEELSAMLNTLEDSVHCKAETASDIVTVLRYCAKPSEYIVQCTPYPRDE 7279

Query 234 LSLVLADVLNTHKSFLLDHRYDPYEIYGTDQFMDELKDIPDPKVDPLNVINSLLVVLHEM 293

L+ VL +V+ T K+FL+DHRYDP+EIY T+++M+ELKDIPDPK +P+ + +L VL+EM

Sbjct 7278 LTAVLEEVICTRKAFLIDHRYDPFEIYCTEEYMEELKDIPDPKEEPMEFLEIMLFVLYEM 7099

Query 294 GPWCTQRAAHHFYQCNEKLKVKTPHERHYLLYCLVSTALIQLYSLCEHAFHRHLGSGSDS 353

GPWC R A + + EK K+KTPHERHY+L C+V+TAL++ +++CE F + ++

Sbjct 7098 GPWCADRVAVNMFHRIEKQKIKTPHERHYILLCVVNTALLEFHAICEQTFKKF----NNH 6931

Query 354 RQTIERYSSPKVRRLLQTLRCFKPEEVHTQADGLRRMRHQVDQADFNRLSHTLESKCRMV 413

++ +ERYSSPKV RLL+ LR FKPEE+ T+ + +++M +DQ DF +LS ++E C+ V

Sbjct 6930 KELVERYSSPKVFRLLEILRLFKPEELLTKNETIQKMSQDLDQMDFQKLSRSIEYSCQNV 6751

Query 414 DQMD-QPPTETRALVATLEQILHT-TEDRQTNRSAARVtptptpahakpkpSSGANTAQP 471

+ + + E+R+++ L+ I+ + T D ++ T T + A G +

Sbjct 6750 ENVTTKLQLESRSIMDNLDPIVKSPTTDVAKQQTLQANTTHKTESSASTNRRHGVGGSHG 6571

Query 472 rtrrrvytrrhhrDHNDGSDTLCALIYCNQNHTARVLFELLAEISRRDPDLKFLRCQYTT 531

R +RR + RR RD+ D DTLCA+I+CN N+TARVLF+LL+E+SR DP+LKFL+CQ+T

Sbjct 6570 RFKRRPFNRRQMRDNGDALDTLCAIIFCNSNYTARVLFDLLSEMSRHDPELKFLKCQFTA 6391

Query 532 DRVADPTTEPKEAELEHRRQEEVLKRFRMHDCNVLIGTSVLEEGIDVPKCNLVVRWDPPT 591

DRVADP TEPKEAE+EHRRQEEVLKRFRMHDCNVLIGTSVLEEGID+PKCNLVVRWD PT

Sbjct 6390 DRVADPITEPKEAEVEHRRQEEVLKRFRMHDCNVLIGTSVLEEGIDLPKCNLVVRWDAPT 6211

Query 592 TYRSYVQCKGRARAAPAYHVILVAPSYKSPTVGSVQLTDRSHRYICatgdtteadsdsdd 651

TYRSYVQCKGRARAAPAYHVILV+P+Y + + QL+D+SHRY+C + D+D

Sbjct 6210 TYRSYVQCKGRARAAPAYHVILVSPAYNAILCENEQLSDQSHRYLCKLEVGQDDVIDTDS 6031

Query 652 saMPN---SSGSDPYTFGTARGTVKILNPEVFSKQPPTACDIKLQ--------------- 693

+ + S+ +T G+++G VKILNPEV + +PPT C+I L+

Sbjct 6030 CSDDEEECNGKSNKFTIGSSKGIVKILNPEVITSKPPTKCEITLKEIKDELPAIKGIENE 5851

Query 694 --------EIQDELPAAAQLDTSNSS---------------------------------- 711

EI+ EL +L SS

Sbjct 5850 DTNTIDLVEIESELAPITELQNETSSIKEVSKTKSVANESLHDCFDVLDLSDMISDISSN 5671

Query 712 -------DEAVsmsntspsessteQKSRRFQCELSSLTEPEDTSDTTAEID---TAHSLA 761

+E + ++ +++ RRF+C + E E+ + E T + +

Sbjct 5670 NSSRILLEETATTPASASVTPKQKKEKRRFRC----ICENENCAQHGLETGLDATINQIE 5503

Query 762 STTKDLVHQMAQYREIEQMLLSKCANTEPPEQEQSEAERFSACLAAYRPKPHLLTGASVD 821

TT +V Q+A+YREIE+MLL KCANTEP E E A+ F+ C+ Y+P HLLTGASVD

Sbjct 5502 FTTNKIVEQLAEYREIEKMLLQKCANTEPSEAEHQLADYFNKCVKRYKPCEHLLTGASVD 5323

Query 822 LGSAIALVNKYCARLPSDTFTKLTALWRCTRNERAGVTLFQYTLRLPINSPLKHDIVGLP 881

L S+IALVNKYC++LPSDTFTKLTALWRCT+ R GV ++QYT+RLPINSPLK+DIVGLP

Sbjct 5322 LTSSIALVNKYCSKLPSDTFTKLTALWRCTKTIRCGVEMYQYTIRLPINSPLKYDIVGLP 5143

Query 882 MPtqtlarrlaalqaCVELHRIGELDDQLQPIGKEGFRALEPDWECFELEPEDEQIVQLS 941

M + LARR+AALQAC+ELH+ GELDD L PIGKEGFRA+EPDWE F+LE ED++IV +

Sbjct 5142 MSSNILARRMAALQACIELHKTGELDDNLLPIGKEGFRAMEPDWENFDLEEEDDKIVLEN 4963

Query 942 DEPRPGTTKRRQYYYKRIASEFCDCRPVAGAPCYLYFIQLTLQCPIPEEQNTRGRKIYPP 1001

EPRPGTTKRRQYYYKRIASEF DCRP AG PCYLY I LTLQCPIPEEQNTRGRKIYPP

Sbjct 4962 SEPRPGTTKRRQYYYKRIASEFSDCRPTAGVPCYLYLIDLTLQCPIPEEQNTRGRKIYPP 4783

Query 1002 EDAQQGFGILTTKRIPKLSAFSIFTRSGEVKVSLELAKERVILTSEQIVCINGFLNYTFT 1061

EDAQQGFGILT KRIPK+S+F IFTRSGEVKVS+ LAKERV+LT QI CIN FLNYTFT

Sbjct 4782 EDAQQGFGILTLKRIPKVSSFPIFTRSGEVKVSIVLAKERVVLTETQIRCINTFLNYTFT 4603

Query 1062 NVLRLQKFLMLFDPDSTENCVFIVPTVKAPAGGKHIDWQFLELIQANGNTMPRAVPDEER 1121

NVLRLQKFLMLFDPDSTENCVFIVPTVK GGK IDW FLELI+ N + MP VP++ R

Sbjct 4602 NVLRLQKFLMLFDPDSTENCVFIVPTVKRD-GGKVIDWDFLELIERNASMMPTPVPEDMR 4426

Query 1122 QAQPFDPQRFQDAVVMPWYRNQDQPQYFYVAEICPHLSPLSCFPGDNYRTFKHYYLVKYG 1181

++ FD +F+DAVVMPWYRNQDQPQYFYVAEICP LSP SCFPG+NYRTFKHYY +KYG

Sbjct 4425 KSIDFDANKFKDAVVMPWYRNQDQPQYFYVAEICPQLSPQSCFPGENYRTFKHYYFLKYG 4246

Query 1182 LTIQNTSQPLLDVDHTSARLNFLTPRYVNRKGVALPTSSEETKRAKRENLEQKQILVPEL 1241

LTIQN QPLLDVDHTSARLNFLTPRYVNRKGVALPTSSEETKRAKRENLEQKQILVPEL

Sbjct 4245 LTIQNVKQPLLDVDHTSARLNFLTPRYVNRKGVALPTSSEETKRAKRENLEQKQILVPEL 4066

Query 1242 CTVHPFPASLWRTAVCLPCILYRINGLLLADDIRKQVSADLGLGRQQIEDEDFEWPMLDF 1301

CTVHPFPASLWRTAVCLPCILYRINGLLLADDIRK+VS D+GLG+Q+I D DFEWPMLDF

Sbjct 4065 CTVHPFPASLWRTAVCLPCILYRINGLLLADDIRKKVSLDIGLGQQEIPD-DFEWPMLDF 3889

Query 1302 GWSLSEVLKKSRE------------------SKQKESL-KDDTINGKDLA-DVEKKPTSE 1341

GWSLSEVLK+S+E +KQ KD +NG+ A D + K S

Sbjct 3888 GWSLSEVLKRSKENNASLDKKTEEQKEKEANNKQSNGFAKDIGVNGEFCAEDNQTKAKS- 3712

Query 1342 ETQLdkdskddkveksAIELIIEGEEKLQEADDFIEIGTWSNDMADDIASFNQEDD--DE 1399

A E+IIEGE+KL+++ +FIEIGTWSNDMA+DI +FN+ D D+

Sbjct 3711 ----------------ANEIIIEGEKKLKDS-NFIEIGTWSNDMANDI-NFNKYSDSLDD 3586

Query 1400 DDAFHLPVLPANVKFCDQQTRYGSPTFWDVSNGESGFkgpkssqnkqggkgkakgpakPT 1459

D+ L P+NV FCDQQ RYGSPTFWDV ES + + K +

Sbjct 3585 DEEECLKYFPSNVSFCDQQMRYGSPTFWDVK--ESQLNKKPDDCAQSKNTTETNNFRKNS 3412

Query 1460 FNYYDSDNSLGSSY----------DDDDNAGPLNYMHHNYSSddddvaddidaGRIAFTS 1509

F YYDSD+S SSY D+DD GPL RIAFTS

Sbjct 3411 FAYYDSDDSFASSYDGNEPTNYSEDEDDECGPL---------------------RIAFTS 3295

Query 1510 KNEAETIETAQEVEKRQKQLSIIQATNANERQYQQTKNLLIGFNFKHEDQKEPATIRYEE 1569

+NEAETIET QE+EKR K+LSIIQATNANER YQ+TKNLL+G+NF ED K +

Sbjct 3294 RNEAETIETDQEIEKRNKKLSIIQATNANERNYQKTKNLLVGYNF--EDLKPNIEAELCD 3121

Query 1570 SIAK-------LKTEIESGGMLVPHDQQLVLKRSDAAEAQVA---KVSMMELLKQLLPYV 1619

SIAK LK IE GMLV ++Q ++L R + + A + + E +LLPY

Sbjct 3120 SIAKFTASTRLLKANIEQSGMLVKYNQPVMLARKERTTNESAAHGEAATTEAFLKLLPYA 2941

Query 1620 NEDVLAKKLGDRRELL-LSDLVELNADWVARHEQETYNVMGCGDSFDNYNDHHRLNLD-- 1676

++DVL L + +L ++D+ LNAD++ H E + + GCGD FDN+ND L L+

Sbjct 2940 DKDVLMSILSKQANVLTIADIARLNADYLKSHPLELFEIQGCGDIFDNFNDKDMLKLEGW 2761

Query 1677 ----EKQLKLQYER----IEIEPPTSTKAITSAILPAGFSFDRQPDLVGHPGPSPSIILQ 1728

+K +KL +ER K + + FSFDRQPDL GHPGPSPSIILQ

Sbjct 2760 GEGYKKLIKLNFERKAGATGCVSLIDVKQESKNMQGNTFSFDRQPDLAGHPGPSPSIILQ 2581

Query 1729 ALTMSNANDGINLERLETIGDSFLKYAITTYLYITYENVHEGKLSHLRSKQVANLNLYRL 1788

LTMSNANDGINLERLETIGDSFLKYAITTYLYITYENVHEGKLSHLRSKQVANLNLYRL

Sbjct 2580 GLTMSNANDGINLERLETIGDSFLKYAITTYLYITYENVHEGKLSHLRSKQVANLNLYRL 2401

Query 1789 GRRKRLGEYMIATKFEPHDNWLPPCYYVPKELEKALIEAKIPTHHWKLADLLDIKNLSSV 1848

GRRK+LGEYMIATKFEPHDNWLPPCYYVPKELEK LIEAKIP HHWKL DL +IK L+S

Sbjct 2400 GRRKKLGEYMIATKFEPHDNWLPPCYYVPKELEKELIEAKIPPHHWKLVDLANIKKLNSA 2221

Query 1849 QICEMVREKADALGL--EQNGGAQNGQLddsndscndfscfIPYNLVSQHSIPDKSIADC 1906

+IC +VREKAD LGL ++ +++ D NDFSCFIPYNLV+QHSIPDKS+ADC

Sbjct 2220 EICALVREKADKLGLFGDEYNCSEDASAVDEIAEGNDFSCFIPYNLVTQHSIPDKSVADC 2041

Query 1907 VEALIGAYLIECGPRGALLFMAWLGVRVLPITRQLDGGNQEQRIPGSTKPNAENVVTVYG 1966

VEALIGAYLIECGPRGALLFMAWLG+RVLP R+ QE RIPGS+ P+ +TVYG

Sbjct 2040 VEALIGAYLIECGPRGALLFMAWLGIRVLPFVRK-PYNPQEPRIPGSSNPDVNGEITVYG 1864

Query 1967 AWPTPRSPLLHFAPNATEELDQllsgfeefeeslgYKFRDRSYLLQAMTHASYTPNRLTD 2026

W P+SPLLH++PNA +EL+ +L GF+EFE +LGYKFRDRSYLLQAMTHASY+PNRLTD

Sbjct 1863 EWTAPKSPLLHYSPNAPQELESMLEGFDEFENTLGYKFRDRSYLLQAMTHASYSPNRLTD 1684

Query 2027 CYQRLEFLGDAVLDYLITRHLYEDPRQHSPGALTDLRSALVNNTIFASLAVRHGFHKFFR 2086

CYQRLEFLGDAVLDYLITRHLYEDPRQHSPGALTDLRSALVNNTIFASLAVRHGFHK+FR

Sbjct 1683 CYQRLEFLGDAVLDYLITRHLYEDPRQHSPGALTDLRSALVNNTIFASLAVRHGFHKYFR 1504

Query 2087 HLSPGLNDVIDRFVRIQQENGHCIseeyyllseeecddaedvevPKALGDVFESIAGAIF 2146

HLSPGLN+VIDRFVRIQ ENGH ISEEYYLLSEEECDDAEDVEVPKALGDVFESIAGAIF

Sbjct 1503 HLSPGLNEVIDRFVRIQNENGHSISEEYYLLSEEECDDAEDVEVPKALGDVFESIAGAIF 1324

Query 2147 LDSNMSLDVVWHVYSNMMSPEIEQFSNSVPKSPIRELLELEPETAKFGKPEKLADGRRVR 2206

LDSNMSLDVVW VYSNMM PEIEQFSNSVPKSPIRELLELEPETAKFGKPEKLADGRRVR

Sbjct 1323 LDSNMSLDVVWQVYSNMMKPEIEQFSNSVPKSPIRELLELEPETAKFGKPEKLADGRRVR 1144

Query 2207 VTVDVFCKGTFRGIGRNYRIAKCTAAKCALRQLKKQGLIAK 2247

VTVDVFCKGTFRGIGRNYRIAKCTAAKCALRQLKKQGLI K

Sbjct 1143 VTVDVFCKGTFRGIGRNYRIAKCTAAKCALRQLKKQGLIKK 1021

**Conserved domains**


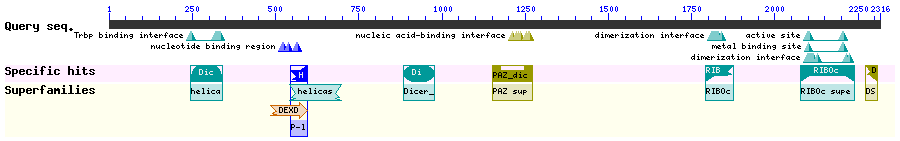


**Argonaute-1**

**>TRINITY_DN32900_c0_g1_i7 len=6281**

CAGCTCTCGTTCACAACATCGTCGCATCGTCGCCGTTTAGTGTTTTGCATCTGTGTCTGT

TTTCGCAGCCAATTTATGGCTGCACAGTTTTCAACTCGGCGCAATTTGTTTTTAACCATT

GGTAATCATTGACGCAAATTCTGGCGGCAGAATTAAAGAAGGGAGTGCAGATTGCAATCA

GATTAGTAAATTATTATAACAATAACAAGTGGTGCATTTGTATGCAAAAGTCTGTATGTA

TATTGTTTTTGTGCGCACCGCGAAATGCTCTTATCAGAAACAGCATAGCAAACAAATGAC

AAAGCTAGTGTACATATTGGTATAGTATGTACATATGTATGGAAATGAAAACCGGAGTAC

TGTAAAACAATAGCAACCAATTGTGCGGTCGAGAGTGTGGTAATTTTTTATATTATTTGT

GCATATACTTACATATGTATGTATGCAAGTATTAGATTTGGTAGCGGTTACTTATATGTG

TTGTTTATTATTGGTTAATGGCTTTACATTTGCAAAGCAGATTATTTATGAAAGTCTAAA

TCTGCAACTACTTGGATTGATTTGAATATAAATAAGAGGAAAAAGGAGAGGAATTGGACT

GAACGAGAGAGCTTGTGTTTATATTGTACATACTTGTAGAAGCGGCTGCTCAAAAGCGAA

AACACCAATAAAATATCCGAAGTGCAAGGCCTCAAAAAGATATTTGTAAATGAGCATTAA

AAGTGAGTGCAAAAGGCACAAATAATATTAAAAAATACGCATTTACTCCTACAGATGTTT

TAATATCCCCATATAATAGAAGATGTAAATTATTGGAGAAACTTGAAGTATCAGCTAAAA

CAGCAATACCAGCAAAAGTGAAAAGCCGAGACAGGAGCGAAGAAAGTCATCCCTCCACTC

GTCTTCCTGCCCACCTGGCATTCAAAACACCCGCATGCCCTTTATGCTTATGTAGTTCGT

TAGTCGCAAAGCTGATTGCTCGGTTCTCGCCCACCCCCTCCCCAGGTTGGATAGCCGGCT

CGGCTACCTCTCTGGTACATTGCGTTGTATGTTGTTGGAGCAAATAAGCAGAAAAAGCAA

CTGAGTGCAGGAGGGGAGCTGCAAAGTCGTCTTTGCTGTCGCAGCTGCTGCCACCGCCAT

TCGCTCTGCTACCAGTGGGAATAAACGAGAAATTGCGTTTTATCGCTTGTGCTGATGAAA

TGTGCTTGTTTTCGGAAATTTCCAAATGTCGAAAATTGTCAAAAAGGTGTTTTAACAATT

GGAATGAAACTATGAGAAATTATGATAAAAAATGCAGTTACATATGTTCATGTTTGTAAT

TAAAAATCACAAATTAAATCTGGAATGTACCTGTTATATACATATGTATATTGATAAAGA

AATTTGAGAAAATGTCGCGCGTCTTTAATGTACCATACATAGTTTATGTACATGTTTAAT

GTTATGAAAATGTTGGCACACTTAAGAAACTTATGAAAATGCTTTAATAAAAACGCAACG

CGAAGATTTAACAATAATAGTGAAAGCGATCATCACAAAGTTACAATGCCTCCTTCAAAA

GAAAGAAAATAAAAAATAAAGTTTGTAAAATGTTTTGTGAAAAACTCTAAAATTGTACCA

AACAAATAAGTGAATTAACTTTTAACGGTGTAAATGAAAAACTGACAACAATTAAACTTC

AAAATTGGCTCGACATTCTGTGTAGGATTATCCCTCAATCACAGAAACAATCGATAAGAT

TATACGGATTAACTCTGCTATTGGAAAAATAAAGGCCTCAAGCTGACGCCGACACCGACA

AAGATGTCCACGGAGCGTGAGCTTGCTCCAGGAGGACCAGCACAGCTCCATACACTACCC

GTGGCTTACTCGGATCTCGGCGCTACACTGCAACTGAACACAGCTGTTCGAATAATTGGC

AAAGTGTATGAGTCACAGTGGACACCTTCGCCACCGAGGCCTCAAAGCCCTTCTCAATCG

CAAACCAACTACGATCCACACACATCACCACCAGCTACCGGTTCATCAGTGAATCCCACC

GCCGTGACCAGTCCGAGTGCGCAAAATGTGGCCGTTGGGGGGGCAACTGTGGGGGGCGCT

GGCGCTACAGGTCACTCCACACAAGCGGCTGCAGTCGCCTCACCCTTGGGAGCGGCAGCG

GCTACTTCGCCTACCACCCAACCTGAACTGCCAGTATTCACGTGCCCCCGTCGGCCAAAT

TTGGGTCGAGAAGGTCGACCGATTGTTTTGCGCGCCAACCATTTTCAGGTGTCTATGCCG

CGCGGCTATGTACATCACTATGATATCAACATTCAGCCGGATAAGTGTCCGCGAAAAGTG

AATCGTGAAATTATCGAGACCATGGTGCATGCGTATAGCAAGATATTTGGAGTTTTGAAG

CCAGTATTTGACGGGCGCAATAATTTGTATACACGTGACCCGCTTCCCATAGGCAACGAT

CGACTGGAATTGGAGGTAACACTTCCAGGCGAGGGAAAAGATCGTATTTTCCGCGTAACC

ATCAAATGGATGGCGCAAGTATCGCTTTTCAATCTAGAGGAAGCCTTGGAAGGGCGCACG

CGCCAAATACCGTACGATGCAATACTTGCTTTAGATGTGGTTATGCGGCATTTACCGAGC

ATGACATACACGCCTGTGGGTCGCAGTTTCTTCAGCTCACCCGATGGTTATTACCATCCA

CTGGGTGGCGGCCGTGAAGTTTGGTTTGGTTTCCATCAAAGCGTACGTCCCTCGCAATGG

AAAATGATGCTAAACATTGATGTTTCGGCCACGGCTTTTTATAAAGCGCAGCCAGTTATT

GATTTTATGTGTGAAGTACTTGATATCCGAGATATTAATGAGCAAAGGAAACCACTCACC

GATTCGCAGCGTGTAAAATTCACCAAAGAAATTAAAGGATTGAAAATAGAAATTACCCAC

TGTGGAGCTATGCGTCGCAAATACCGTGTCTGCAATGTAACACGTCGTCCAGCACAAATG

CAATCATTTCCACTTCAACTTGAAAATGGCCAGACTGTGGAGTGTACCGTGGCCAAATAC

TTCTTGGATAAATACCGCATGAAACTGCGATATCCCCATTTGCCCTGTCTACAAGTTGGG

CAGGAACATAAGCACACTTACTTGCCTCTAGAAGTGTGTAATATAGTCGCCGGTCAACGT

TGTATCAAAAAACTGACTGACATGCAGACATCGACGATGATTAAAGCTACGGCTCGTTCA

GCACCCGATCGTGAACGTGAAATCAATAACTTGGTTAAGCGTGCCGATTTCAACAACGAT

TCTTATGTTCAAGAATTCGGTTTAACTATTTCTAACTCTATGATGGAAGTGCGAGGACGT

GTGTTACCACCCCCCAAACTGCAATATGGTGGACGGGTATCAAGTATGACCGGCCAACAA

CTTTTCCCACCCCAGAATAAGGTCAGTTTGGCTTCCCCAAATCAAGGTGTCTGGGACATG

CGTGGAAAGCAGTTCTTCACTGGTGTGGAAATTCGCGTATGGGCAATTGCTTGTTTTGCG

CCTCAAAGAACCGTGCGTGAAGATGCTTTGCGTAATTTTACGCAGCAATTACAAAAAATT

TCGAATGATGCCGGCATGCCCATAATTGGACAACCTTGTTTTTGTAAATACGCGACTGGC

CCAGATCAGGTAGAACCTATGTTCAGATATCTGAAGAATTCATTTAACGCACTGCAATTG

GTGGTGGTTGTGCTACCCGGAAAAACACCAGTCTATGCTGAGGTAAAACGTGTTGGCGAC

ACCGTGTTGGGTATGGCCACACAATGCGTACAGGCAAAAAATGTTAATAAGACATCGCCG

CAGACCTTGTCGAACCTTTGTCTGAAAATTAATGTGAAATTAGGAGGAATAAATTCCATA

TTGGTACCTTCAATTCGTCCGAAGGTCTTCAACGAACCCGTTATTTTCTTAGGCGCAGAC

GTGACTCATCCTCCAGCCGGTGATAATAAGAAACCCTCTATTGCAGCGGTTGTTGGCTCA

ATGGATGCTCATCCGTCACGCTATGCGGCCACAGTACGAGTCCAGCAGCATCGCCAAGAA

ATTATACAGGAATTAAGCAGTATGGTCCGCGAACTATTGATTATGTTTTATAAATCTACT

GGCGGTTATAAGCCTCATCGTATCATTTTGTATCGCGACGGCGTCTCAGAAGGGCAATTC

CCTCACGTATTGCAACATGAGTTGACTGCTATACGTGAGGCATGTATCAAACTGGAAGCA

GAATACCGCCCTGGTATAACATTTATTGTTGTACAAAAAAGGCATCATACTCGCCTCTTC

TGTGCTGAGAAAAAGGAACAGAGTGGAAAATCTGGCAATATACCAGCTGGTACCACAGTC

GATGTTGGTATTACACATCCAACCGAGTTTGATTTCTATCTATGTAGCCACCAAGGAATA

CAAGGTACAAGTCGGCCTTCACATTATCACGTTTTATGGGATGACAATCACTTTGATTCC

GATGAACTACAGTGCTTAACATATCAACTGTGTCACACCTATGTGAGATGTACACGCTCA

GTGTCGATACCAGCACCGGCTTACTACGCTCATCTTGTGGCATTCAGAGCCAGATACCAT

TTAGTTGAAAAGGAACATGATTCAGGCGAAGGATCTCATCAAAGCGGTTGCTCAGAAGAT

CGCACACCTGGTGCGATGGCGCGCGCAATAACGGTACATGCGGACACAAAGAAAGTCATG

TACTTTGCTTAAATCCATTTTGCAAACATTCTTATGTTATATCATTAACACAAAAGTTTA

GATTTTCCAGCCAGTAAAATATTGCTGGATCTCATTACGCATTTCCAATTCAATTTGTAC

TTTCTTCGAAAATTTTGGTCTGTGTTGTTTGGTACATTTGAATCAGAGATCATATTATAT

TATAAAGAATAACATCAATTCTAAGCAACTAAAAAAACCACTAAAACGAAATGGAGAATG

CGGATAGCGCTCAAAATACCTAAATGAATCCAATCAACATTTTACTGGTTTAAGTATCTA

GAATTGCATCAAGGCAGTCTTAAGTAATGTGAATCTTCAGAAAAAAGGTAAAAATTAACC

AAAAATGACTTTTAAAATGGAACTCAAGCCATGCCTAAGCTATTAAATTACATCTTAAAT

TGTAATAAGCTTTTAGTAAGTTTAAGCTTCTTCCATATTTGTTATATTTGCTACAAAGTA

ATTCCTACTAATTTATTTTTAAATCTCCAATAGAAAAAAAAGATAAGTATAAAAGTGTTA

AAAAATCATTTCGGATTTATGTGAAATATATTAATATTAATATATGAGACTTCGTATGAG

TTTCCTAATACGATCCGCAAATATGAAAAATTCAATTGCCACTTATGGTATGCTTACTAT

GTACTCGTATGTGTAGCACAAATACGAACTGATTGCTTACATACCAGGATTTGATTTTCG

AATTGTGTTATACAAAATTTATATAAATATTTTCATAGATTGATCAAGGTTTTAATTTCA

CTCTTGTTATCTTATTTTTTGAAAACGTGCATCCCCATAAAAAGAGTCCATTTATATCTG

AGGAATGTGCAGATGTACATTTTTTATTAAACACGGAGAATATCGCTAATTGACTCAACT

AAGAATCAACTACAAAATCATATTGCGCTCTACACAAAATAAACATGGTGGGATGGAAAA

AACTGTACACAAATTTATACATAAATGTCAGCGTCATTACTTAATTTTGTATTGCCAAAC

CGCATATTTTATTACTACAATGATGTAAACAACGTGAAATATGTCAAATGCTAATGTTAA

AAATTAGATTTGACTTAAGATATTCGTACGCGCCTACTTGTGGACTAGTTAATATGTAAA

GACCCCCCAATCTGAAGGGAGTAAATTCTTAATTTATTAACTATTTAAAGTACCAAATAT

AGTTGTCCGACTTTGGTCTATCTGCCCTAGTCGGTTTGTACTCTCAAGCGCATCAGTCGC

ATTTCTCTTGCTTTCAACTTTGACAGACATACACACTATGTACTTCTTATAAATATATTA

ATAAGACAATAGCGCTGATGGATTAAGCAATTTTTATTATAAGGAGACTCTCCCAAAAAA

CCAGATATCTTAAACTTTCTCCAACAATTCAATTTCATCGGTATATAATTTAAACAATGT

ACTCAAATGTATTTATATTAAAAAATAGTACAATTATTGAAAAAAAAAAATTAATTATGT

TGATGCAGCAGCATTTAACTTCCGTTTTAAGTTTGCCACAA

**tBLASTn(First hit)**

Score = 1823 bits (4723), Expect = 0.0, Method: Compositional matrix adjust.

Identities = 927/988 (94%), Positives = 945/988 (96%), Gaps = 10/988 (1%)

Frame = +1

Query 1 MSTERELAPGGPAQLHPHTLPLTFPDL----QMTSTVGIIGKVYEsqwtpsptrpqspsq 56

MSTERELAPGGPAQLH TLP+ + DL Q+ + V IIGKVYESQWTPSP RPQSPSQ

Sbjct 1804 MSTERELAPGGPAQLH--TLPVAYSDLGATLQLNTAVRIIGKVYESQWTPSPPRPQSPSQ 1977

Query 57 aqtsFDTLTSPPAPGSSVNPTAVTSPSAQNvaaggatvagaaataaqvasalgattGSVt 116

+QT++D TSPPA GSSVNPTAVTSPSAQNVA GGATV GA AT +A S

Sbjct 1978 SQTNYDPHTSPPATGSSVNPTAVTSPSAQNVAVGGATVGGAGATGHSTQAAAV---ASPL 2148

Query 117 paiatatpatQPDMPVFTCPRRPNLGREGRPIVLRANHFQVTMPRGYVHHYDINIQPDKC 176

A A +P TQP++PVFTCPRRPNLGREGRPIVLRANHFQV+MPRGYVHHYDINIQPDKC

Sbjct 2149 GAAAATSPTTQPELPVFTCPRRPNLGREGRPIVLRANHFQVSMPRGYVHHYDINIQPDKC 2328

Query 177 PRKVNREIIETMVHAYSKIFGVLKPVFDGRNNLYTRDPLPIGNERLELEVTLPGEGKDRI 236

PRKVNREIIETMVHAYSKIFGVLKPVFDGRNNLYTRDPLPIGN+RLELEVTLPGEGKDRI

Sbjct 2329 PRKVNREIIETMVHAYSKIFGVLKPVFDGRNNLYTRDPLPIGNDRLELEVTLPGEGKDRI 2508

Query 237 FRVTIKWQAQVSLFNLEEALEGRTRQIPYDAILALDVVMRHLPSMTYTPVGRSFFSSPEG 296

FRVTIKW AQVSLFNLEEALEGRTRQIPYDAILALDVVMRHLPSMTYTPVGRSFFSSP+G

Sbjct 2509 FRVTIKWMAQVSLFNLEEALEGRTRQIPYDAILALDVVMRHLPSMTYTPVGRSFFSSPDG 2688

Query 297 YYHPLGGGREVWFGFHQSVRPSQWKMMLNIDVSATAFYKAQPVIDFMCEVLDIRDINEQR 356

YYHPLGGGREVWFGFHQSVRPSQWKMMLNIDVSATAFYKAQPVIDFMCEVLDIRDINEQR

Sbjct 2689 YYHPLGGGREVWFGFHQSVRPSQWKMMLNIDVSATAFYKAQPVIDFMCEVLDIRDINEQR 2868

Query 357 KPLTDSQRVKFTKEIKGLKIEITHCGQMRRKYRVCNVTRRPAQMQSFPLQLENGQTVECT 416

KPLTDSQRVKFTKEIKGLKIEITHCG MRRKYRVCNVTRRPAQMQSFPLQLENGQTVECT

Sbjct 2869 KPLTDSQRVKFTKEIKGLKIEITHCGAMRRKYRVCNVTRRPAQMQSFPLQLENGQTVECT 3048

Query 417 VAKYFLDKYRMKLRYPHLPCLQVGQEHKHTYLPLEVCNIVAGQRCIKKLTDMQTSTMIKA 476

VAKYFLDKYRMKLRYPHLPCLQVGQEHKHTYLPLEVCNIVAGQRCIKKLTDMQTSTMIKA

Sbjct 3049 VAKYFLDKYRMKLRYPHLPCLQVGQEHKHTYLPLEVCNIVAGQRCIKKLTDMQTSTMIKA 3228

Query 477 TARSAPDREREINNLVKRADFNNDSYVQEFGLTISNSMMEVRGRVLPPPKLQYGGRVSTG 536

TARSAPDREREINNLVKRADFNNDSYVQEFGLTISNSMMEVRGRVLPPPKLQYGGRVS+

Sbjct 3229 TARSAPDREREINNLVKRADFNNDSYVQEFGLTISNSMMEVRGRVLPPPKLQYGGRVSS- 3405

Query 537 LTGQQLFPPQNKVSLASPNQGVWDMRGKQFFTGVEIRIWAIACFAPQRTVREDALRNFTQ 596

+TGQQLFPPQNKVSLASPNQGVWDMRGKQFFTGVEIR+WAIACFAPQRTVREDALRNFTQ

Sbjct 3406 MTGQQLFPPQNKVSLASPNQGVWDMRGKQFFTGVEIRVWAIACFAPQRTVREDALRNFTQ 3585

Query 597 QLQKISNDAGMPIIGQPCFCKYATGPDQVEPMFRYLKITFPGlqlvvvvlPGKTPVYAEV 656

QLQKISNDAGMPIIGQPCFCKYATGPDQVEPMFRYLK +F LQLVVVVLPGKTPVYAEV

Sbjct 3586 QLQKISNDAGMPIIGQPCFCKYATGPDQVEPMFRYLKNSFNALQLVVVVLPGKTPVYAEV 3765

Query 657 KRVGDTVLGMATQCVQAKNVNKTSPQTLSNLCLKINVKLGGINSILVPSIRPKVFNEPVI 716

KRVGDTVLGMATQCVQAKNVNKTSPQTLSNLCLKINVKLGGINSILVPSIRPKVFNEPVI

Sbjct 3766 KRVGDTVLGMATQCVQAKNVNKTSPQTLSNLCLKINVKLGGINSILVPSIRPKVFNEPVI 3945

Query 717 FLGADVTHPPAGDNKKPSIAAVVGSMDAHPSRYAATVRVQQHRQEIIQELSSMVRELLIM 776

FLGADVTHPPAGDNKKPSIAAVVGSMDAHPSRYAATVRVQQHRQEIIQELSSMVRELLIM

Sbjct 3946 FLGADVTHPPAGDNKKPSIAAVVGSMDAHPSRYAATVRVQQHRQEIIQELSSMVRELLIM 4125

Query 777 FYKSTGGYKPHRIILYRDGVSEGQFPHVLQHELTAIREACIKLEPEYRPGITFIVVQKRH 836

FYKSTGGYKPHRIILYRDGVSEGQFPHVLQHELTAIREACIKLE EYRPGITFIVVQKRH

Sbjct 4126 FYKSTGGYKPHRIILYRDGVSEGQFPHVLQHELTAIREACIKLEAEYRPGITFIVVQKRH 4305

Query 837 HTRLFCAEKKEQSGKSGNIPAGTTVDVGITHPTEFDFYLCSHQGIQGTSRPSHYHVLWDD 896

HTRLFCAEKKEQSGKSGNIPAGTTVDVGITHPTEFDFYLCSHQGIQGTSRPSHYHVLWDD

Sbjct 4306 HTRLFCAEKKEQSGKSGNIPAGTTVDVGITHPTEFDFYLCSHQGIQGTSRPSHYHVLWDD 4485

Query 897 NHFDSDELQCLTYQLCHTYVRCTRSVSIPAPAYYAHLVAFRARYHLVEKEHDSGEGSHQS 956

NHFDSDELQCLTYQLCHTYVRCTRSVSIPAPAYYAHLVAFRARYHLVEKEHDSGEGSHQS

Sbjct 4486 NHFDSDELQCLTYQLCHTYVRCTRSVSIPAPAYYAHLVAFRARYHLVEKEHDSGEGSHQS 4665

Query 957 GCSEDRTPGAMARAITVHADTKKVMYFA 984

GCSEDRTPGAMARAITVHADTKKVMYFA

Sbjct 4666 GCSEDRTPGAMARAITVHADTKKVMYFA 4749

**Conserved domains**

**protein containing domains ArgoN, ArgoL1, PAZ_argonaute_like, and Piwi_ago-like**

**
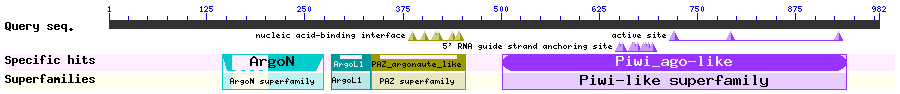
**

**Loquacious**

**>TRINITY_DN27977_c3_g1_i4 len=2162** CTGTACATGTAATTATCAGAAAAGGTTATTTATTCGAATTCTTTGCAATAGTTTATAATA

TAACTCATTCGTATCATGTCTAGAGATTTCATTTTTATGTGAATGTATGTACTATATATA

GTTATGTATGTATGCGTCATAGATTTATGATTTCAGCAGTTATGGCCGAAACAAATGATT

AAAATGACGAATAATTATGCACTTAATTCAAGAAAATATATATTGTGTGTCAATGAAGAC

CCTGGCGGCGTAGAATGACCTAGTAGACCAGTCTCCATCGTATATTGTATAAATAAATAT

GTGTGCCAATTTAAAAGTGCAAAATTATATTTAAACTATCTTACTTCCATTCAGAATTGC

CCAAAATACACTGTTAATGCATTAATCGTGTCTTGACGTTTGCGAACGTGTTCGCTTTCC

CACACTTACTGCATTTTTACGAAAAACATAATAATAAAAATAAAATAAAGCAAGTTTAAC

ATAGTTAATTTTTTTGTATTAATGTTTTTAACATATTTCATGTAATTGTGGTTAATTTTT

CAGAAAAACATTGTGAACTCATAAGTTTCTGCTCTCTATCACAAAACTGCCATAGGTATA

TTCAAAAAGACATATTGTAAATGTAATCGTTGCACAAATACATATGGTCTTTAAAATAAA

AATTTTTCCAAATTTCTTTATCGCCGCTTTTTCATATTTTGCTTCCATTATAATCGTCTT

AAGCAGAAATACACATAGAAAATTTCACTGCTTCACTGGACTTCATTTGTTTGCAGTACC

AAACAATTTTGAATTTCTTTCGTGTTACTTATTAAATTGCATATTATTTCTTTTTTTTTA

ATATCTTATTTTAATTCTAATTCTTTTGTTTTTTCTTCACTTGTTTCGCTGCTGTATCAA

GCGATTTACTTCTTTGTCATAATTTTCAAATATTCCAAAGCGTTTTGAGCAGCCTGTTTC

TGTGCGTCAGCTGCTGTTGGTCCAGAGCCATGACATACGCCGACTGGCAATGTTGAAAGC

TGCACTAGACATTGGCATTGGTCAGTGAAAGTTTTCTCTTCAATGTCAACGTATGTGACT

TCAAACTGATTTTCATTGGCAATCTCCGACAACAATTTCACATAATCAATTTTGGGTCCT

TTTAGACAAGTTTTCTGTAACTTGAGTAATTTCTTTCCAGTAGCATTTTTAAGTGTTTTG

TGGAACTGTGAGACCTTATTACTATGCTGGCTGGTTAAGGTGGGCACAGTGATATCTTTC

AAATCACCATAATAATTTGCACAGTTACGATTGTCGCCATCGAGATCGACACGCATTGAT

TCACATATTTGATTGTTGTCCAATGGGTTTTCTTGTAGACGTGTCCACATCTTGTGTGCA

GCCAGACGTTTAGCGATCTTCTTGCTTTTCCCTTTTCCCACTTCACGATAATTAAGGATT

GAACAGGCAATGGTAAATAAACGCTCATGCGGCAAGCCAACCTCTGTTTCAGTTTCGTAA

GTTGGTGGTGGCCAGCGACGTGACATGCACATCTCCTGCAGCCAGCCAATCGGATTTCCA

ACTATTTTATCGGCGCCATCGCCGCTTCCAACTGCGTTTACGTTGGCGTCACTGCCGGCG

CTTGTAGCAGTAATGCCGCTAGAGGCTGTACTAACACCTGCACCGGCGCATGCTTGTGCA

GTATCGGGTAACTGTACACCAGTTAATTTATCAATTAGTGCACGTGCTGCTGAATGCTTA

GCCTCTTTTTTGGAGCGTCCAGCGCCCATAGCCGTGAAGGGCACATCCTTGTCATTAAAC

GCGACACGATAACGAAATGTTGGCTCATGTATGGCACCCTCAATTTGCACCAACTCATAA

TTGGGTGTAATTCCCCGACGACTCAAGAGCTCTTGTAGAATGGATACTGGTGTCTTCATA

GCCAACCCATTACTATCGGTCTGAAAAGAAAAAAATTCAAATTTATCATTTTTGGAGTTT

ACTCTTAAAAAAAGCAATTCTAATTGACCCTTAATTCCGTCTAATAGAACCGAATATTTG

TTCAAAACATTAATATTATATTTAATAACATCAATATTTGAACATTCCCAATTGTCAGTA

AAAAGCTTAGATATAACTCCTTTGTTAATAATATTATTAGCAGGTGCTAACTTAATCCTA

AG

**tBLASTn(First hit)**

Score = 332 bits (851), Expect = 6e-106, Method: Compositional matrix adjust.

Identities = 165/228 (72%), Positives = 191/228 (84%), Gaps = 4/228 (2%)

Frame = -1

Query 122 FVSETDANGLAMKTPVSILQELLSRRGITPGYELVQIEGAIHEPTFRFRVSFKDKDTPFT 181

F +TD+NGLAMKTPVSILQELLSRRGITP YELVQIEGAIHEPTFR+RV+F DKD PFT

Sbjct 1952 FSFQTDSNGLAMKTPVSILQELLSRRGITPNYELVQIEGAIHEPTFRYRVAFNDKDVPFT 1773

Query 182 AMGAGRSKKEAKHAAARALIDKLIGAQLPESPSSSAGPSVT----GLTVagsggdgnana 237

AMGAGRSKKEAKH+AARALIDKL G QLP++ + AG V+ G+T +G D N NA

Sbjct 1772 AMGAGRSKKEAKHSAARALIDKLTGVQLPDTAQACAGAGVSTASSGITATSAGSDANVNA 1593

Query 238 tgggdasdKTVGNPIGWLQEMCMQRRWPPPSYETETEVGLPHERLFTIACSILNYREMGK 297

G GD +DK VGNPIGWLQEMCM RRWPPP+YETETEVGLPHERLFTIACSILNYRE+GK

Sbjct 1592 VGSGDGADKIVGNPIGWLQEMCMSRRWPPPTYETETEVGLPHERLFTIACSILNYREVGK 1413

Query 298 GKSKKIAKRLAAHRMWMRLQETPIDSGKISDSICGELEGENESVKHLF 345

GKSKKIAKRLAAH+MW RLQE P+D+ +I +S+ +L+G+N + + +

Sbjct 1412 GKSKKIAKRLAAHKMWTRLQENPLDNNQICESMRVDLDGDNRNCANYY 1269

**Conserved domains**

**DSRM domain-containing protein**

**
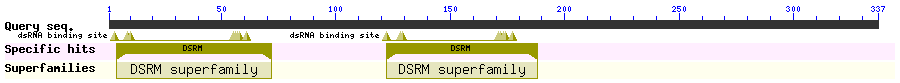
**

**Drosha**

**>TRINITY_DN30547_c4_g2_i1 len=4616** TTTACCAATGACAGCATTAGGAATCGTTAATCACTAACACGTAGTTTTTTTATTATAATT

TAGTAAGTACACAAAATAATTGTTTCCTTTTATTGGAAGTGCTTAAATATGAATTTTATT

TAACACTTTTTAGATTTAATGTAGATAAATAAGATGGAAATTAGCTACTAAGTTCCCCTG

ACTCTATATCAGAGTTTGACGAAACATCTTCCACCAAATCTTTTAAGTCATGAGGCATTG

TATTTCCGACTGATCTAACATTTTTACATGCGGAGCTAGGCATAGGCTTATTCGGTGATA

ATTTATGATTTTCGGCTGTAAAAGAAACTGTGGAAATTGTATTTTCTCCAAAGCAATCGT

CACTACTGACATCTTCGCAATTCCTTTTGTTGGTGTTCTGATGATCCTCCTCTTCCACTT

GGTGGCACATTCTTTTAGATGAAAATAAATTTGACCTCGATCCGTGCCCAATAAACTCTA

GTTTTTTAGTGCGGCTTCTTTTTTTCAGCTTTGGTACGCGGCCCTTTTCCAATATTTCAT

CGTCTGAACTTGAGCTGCTTGAACTGGTATGACCTGAATGACCTTTGTTGGTTATATTTC

CAGCATCGTTGATGTCATCTTCTGGTAGCTCATCACTTGAAATGTCTTCGCGAACTCGGT

ATTGCTTAGGAAGATGTGATTCATCCTCCATTGAGTTCGTAATTTTAGGTTTAGTTTGTT

CAGCCAATATCTCTTCGAGTTCTGGTTTTAGTTCGGTACCCTTTTGTTTTTTGATACTTT

TAGCAATTACACGCTTTTGGTGATCCAATTGAGGGAATAAATCACGAGAATTCTCCAATG

CTTGTTTCGCTGCATTCATTTCAGCCTGCTGAATAGAAGAACCGGTTGCGGTCGCTAAAC

GTTTAGACCGAAAATAAACTGCAACTGTATACACTCTCGTATTAGTTGGTCCAATAGACT

CGATCACTTTATAAATCGGAATGTCAGGTTCCCCTCCTTCCATCGTTCGCAAGGTAAGGC

AGCATTGTTGCAGCTTTGATTTGGGATCATTCCAATCCTGATTCATAATAAATATTTGCA

AACGTGGAAATAAGCACACATGGCAAAATTGCTCGCAATAGAGGAGGCCTTTGTCAACAT

ATAGTGCACCTAAAAATGCTTCTAACAAATCTGCACGATCCTTTGTCTTCAACTCTACCT

TTGGGTTCGAATAAACTGCATATTTAGTCATACCAAGATCATCACAGACTACTGCTTGAG

TGCGGTTATTCACGAGAGATGATCGCAAGAGCGACAAATGACCCTCATGATGTTCAGGAA

AATGACGATAAAGATATTCCGAACAAATTAATTGAAGAACAGTATCACCGAGAAATTCCA

AACGTTGATTAGATCCCAATGTAAGATGAGTAAATCCAATGGAGCGATCCGTAAAAGCAC

GAGCTAGTAGACGTATATGCTTGAATTGAATGCCAATGGATTCCTCAAATTTAGTTAGTT

CTTGAAGCACAGGATAGGAAGCAATACAAGCCCGATCTCCTAATGGTTCCTGTTCTTGCA

GCGGATGTTCCGGATAATTTTTCCAGATTTCTCTTAATTGCTCGTCTTCCATGTATAAGG

CGTTCATAAATACTTCATCAGCTACTGATATACCACCATCAAGTAGGAGCGCACCCATTA

ACGCCTCAAAACAGTTAGCCATAGCATGTCGCAATTCCAATTCATGACATAAATCAGAGC

CGTGTGCATAAAGCATGTATTCTTCCAAGTGCAACTTTTTAGCTAGCAATGCCAAATGTT

GATTCTGCACAATAGCCGCGCGATATGTGGCTAATCCACCTTCCTCCAAATCGGGAAACA

TGAAAAACAAATGGATGGAACTTAAGAATTCAACAACGGCATCACCCAAAAATTCTAATC

GCTCATTATGTGTTATATTTGACATCGTTTCGTATTCCTTGCCGAATCTTGACATTATAT

TTATAAGGGTATTTATGCCACGTTTTCGTGTATTCAAGTAATGTATTTTGCGGTCACCAT

ATTCGGGTTGCCGAATGCCGCAATTAGTAAGTGAGTTTCGGGCATGATCAGGATTCGTTC

CATAATTTTCTTTGTATGATGGATGCGTCAGAGCTAATTGCAATAAATATCGGTTTTTAA

AATTATAGCCGATATTTTGCTCTAAAAGATCAAGGGATCTATGGAATCGTAAATGGCCAG

TAAGTACCGGGATGAGCATTGCATGTTGGACAATGTCACACATTATTCCAGTTCTGTAAT

AACTCTTCGAGCTTACAGCTACTGTTATGTTACGTTTCATGCGACCTTGTGTTCGCATTT

CCTGCAATCGTGCCTCCTTTTCTTCCAGCTTACGCTTGTCAACAAATGATGGTTTGGACA

TATTAGCCATCAAGTGGCGATATTTCACATATTCGCGCCATGCTTTTTGGTACTCGGGAT

TACCAGCATAGCTTAATTGTGGTGGGCGTATACCAAAATGAACGATTGCTGGATAGGTAA

CGTTTTCTTCGACATCAAAACATTCAGGCAAATCAGAAACATTTCGATCTAACTGATCAA

CCCTCACAGAACACGGCTTGAATCCAGGCTTTGTCACAAGCATGCCTTTTATAAAGTCAA

CGTAATCCTGCCAATCATGCTGGCTGATATCCTTTATGCAGCGCAGTTGTTCTCTATCAA

CAAGAGGTGCTGCACTATCTAAAAGGTATCGAAGAACTTCACTCATAGCCAATACTTCTT

TTCCGTTATCTGGCAAGTCTCTTACAAAACGTGGAAAGAAATGAAAGGCGGGGCATGTTT

CTGAACTTGACATGCCGCTAGGGTACAATGAGAAATCCACCAGCTCCAGAAGTTCGTGGA

ATAGGTATTTATTGAACAACTCTAGTTCTCTTATAGTAAAGTTTTCCGGCATCTTTTCTT

CAACATATAGGATAGTATACTCGATATTAAAACGAATAACTTTACATGTTGGTAAGTCCG

CAAGAGGAGAATGTGTCAACATAGAAAACCCTTCAAATAAGAACTCATGTTCATCATGCT

TAATTATTGTTGGCGTTTTAGTAAGGAAGTTCGTCGGAGGTGATATAGTAATACGATAAT

GATAAAGTCGCGATGCATTATTTGAATTCATCACACATTTTGAATACCCTGTTTCACCAG

GATAGATACCGTGGCGTATACCAATGCGGCGCGCCTTTGCAGAACAACGACAGAGGGGAC

CGTCATTCATTTCACCTGAATCATTATGCCATAAATCAGCATGAACCCGATATGGATGCT

GTATTTTCCGAGTGAGTTCTTCCATACAACAATCTTGCTCGATTTTAAGATCATCGTCAG

ATGAATCTTCAGAGGAAGATGAGCAAGCTTCCGATTTATGTTTACACAATCGACGACGTG

GTTTACGTGTTGGTGCCTCATACGGAGGTAATGTGTCGCTTATACGTTCAGCACGTTGCA

CTAACTTTTCTTCAAACAATTTGCAGAGGGCTTCTAACCTGCTCGTGGAAACAACCTCAT

TTGAGACATTTGTACGTCGATAAAAGAGGTCCGCTGGAGACGATCGTATCCATACGCTTC

GAGAATTTTCATCCTGTTCACCTAATTCTGCTAATTTCTCGGCAATATCATCTGATGTTT

CGCAATAATTTGATCTCCAACTTTTCAATAATCTATCTCTTTCAGTTTCATACTTTTGTT

CTTTGCTGTCATTCCGGGACTCATTTTTCCTCTCTCTTTGGTTGTAGAATCTACTAGATT

CTGAATATTTGGCATAACCGTAACCTCTTTGGGTTGATGTAGGCGTAAAAGGAAGACGAT

GAGTGTAGGTGGAATTAGATGTATTGCGAAAAGCTGTTCTGGGTACACCATGGCCAGGAT

TCGATGTTGAATTGTTGTACTCATATGAAAATGCTCTTGCGCCATCAGGTGGCTTATAAT

GTACTTTATTCATCACCATGTCCCTGCCACTCCTGTAATTGCGATGAATAGAGTCTTCGT

ACTTTTGTTTGTGATAGTTGTATTCGTTATATGTGGGTTCATAAGGAATATATTCAGTTT

TTCTGGGGGCAGGATGTGGCCCTTTATATCCTTCTTCTTTTAAATACCCATCTGCAAAGA

CAGTCGCATCCCGAAAATGAGGATAAATGGGTGTTCCCCCTTCTATACGTGAATTTTGAT

AAAAAGTTGATGGGAAAGAGTGAAAGCTATTAGTTGCAGGAGTTTGAGGCATTGGTGGTG

CCACGGAGTAATTTGAAAAATTCGCGCAATCTACATCCTTTGAAGTCGATAAATGAAAAG

TTTCAGGTGCTGAAGGAGGCGGAGGTGGCATGCCTACAGGCAGCGGAGGCTGTGTTTTCG

TCGGAACATTCTCCGTTGGCAACGGTGGCATTGTGGACAAATGTTGAAAATTCATTTTTT

TCAGGCGATTTCAAATATCAGTACTTTACTTATATCTGGACAAAGAAAACACAACTTGTT

TAATTAAAACTTCTAATTCATTAACAGTTGAAATTAAGTAAAATTTCCATCACAGTTGAT

AAATTCTAACACTTTTGACTATCGATCAGTACATTCCCATAAAACACAGAGTGTCG

**tBLASTn(First hit)**

Score = 1719 bits (4452), Expect = 0.0, Method: Compositional matrix adjust.

Identities = 892/1216 (73%), Positives = 992/1216 (82%), Gaps = 59/1216 (5%)

Frame = -2

Query 59 PAPYASSVPSYDPYQQPPAYGYEGYAYNEQAQKYGGQESHYQYQYPASGSSFLYES--YK 116

PAP + Y PY+ P Y YN QKY H Y+ SG + YK

Sbjct 4093 PAPRKTE---YIPYE--PTYN----EYNYHKQKYE-DSIHRNYR---SGRDMVMNKVHYK 3953

Query 117 YPDRYPAYSSNYRPPSERQRYTSNSSSQGYHHYPGYSSGRRYEQRHDQEHRQIQDSRYAH 176

PD A+S Y +NS+S PG+ R R +S Y H

Sbjct 3952 PPDGARAFSYEY----------NNSTSN-----PGHGVPR-------TAFRNTSNSTYTH 3839

Query 177 EPRHGHYAHRQAKGSQHGYYGSAARNQVSDDYSPRGHHERERNETLEKTRAKPKVETERD 236

+Q GY G A ++ S Y+ R RN++ E+ K ETERD

Sbjct 3838 R------LPFTPTSTQRGY-GYAKYSESSRFYNQRERKNESRNDSKEQ-----KYETERD 3695

Query 237 RLLRQWCSNFCEKPEDYVKKMNALSEADAPVES-WVRSSPAELYYERTKSENEVRGRARL 295

RLL+ W SN+CE +D +K+ L E D S W+RSSPA+L+Y RT NEV +RL

Sbjct 3694 RLLKSWRSNYCETSDDIAEKLAELGEQDENSRSVWIRSSPADLFYRRTNVSNEVVSTSRL 3515

Query 296 QKLCTLFDEELLQRAKRVREKLPVYVPPPRKARRRVCKHKHKSEACssssssdddsdEDA 355

+ LC LF+E+L+QRA+R+ + LP Y P RK RRR+CKHK SEACSSSS D

Sbjct 3514 EALCKLFEEKLVQRAERISDTLPPYEAPTRKPRRRLCKHK--SEACSSSSEDSSDD---D 3350

Query 356 FKIEQDCCMEELSRKVQHPQRVHADLWHNDAGEMNDGPLCRCSAKSRRIGIRHGIYPGET 415

KIEQDCCMEEL+RK+QHP RVHADLWHND+GEMNDGPLCRCSAK+RRIGIRHGIYPGET

Sbjct 3349 LKIEQDCCMEELTRKIQHPYRVHADLWHNDSGEMNDGPLCRCSAKARRIGIRHGIYPGET 3170

Query 416 GYKLCDPNSNNAGKLFHYRISISPPTNFLTKTPTIIKHDEHEFLFEGFSLLSHVRLSDLP 475

GY C NSNNA +L+HYRI+ISPPTNFLTKTPTIIKHDEHEFLFEGFS+L+H L+DLP

Sbjct 3169 GYSKCVMNSNNASRLYHYRITISPPTNFLTKTPTIIKHDEHEFLFEGFSMLTHSPLADLP 2990

Query 476 VCKVIRFNIEYTIEYEEEKMPENFTIHELDIFFKYLFHELLELVDFNLMPNLPSGNVEES 535

CKVIRFNIEYTI Y EEKMPENFTI EL++F KYLFHELLELVDF+L P+ S + E+

Sbjct 2989 TCKVIRFNIEYTILYVEEKMPENFTIRELELFNKYLFHELLELVDFSLYPSGMSSS--ET 2816

Query 536 CPAFHFFPRFVRDLPDNGKEVLAMVEVLRYLLDNSAQLVERQQLLHLNQISQSEWQNYVD 595

CPAFHFFPRFVRDLPDNGKEVLAM EVLRYLLD++A LV+R+QL + ISQ +WQ+YVD

Sbjct 2815 CPAFHFFPRFVRDLPDNGKEVLAMSEVLRYLLDSAAPLVDREQLRCIKDISQHDWQDYVD 2636

Query 596 FIKGMLVTKPGYKPCSLRVDQLDRNNSDLPECVDRETGISHPAIVHFGICHPQLSYAGNP 655

FIKGMLVTKPG+KPCS+RVDQLDRN SDLPEC D E +++PAIVHFGI PQLSYAGNP

Sbjct 2635 FIKGMLVTKPGFKPCSVRVDQLDRNVSDLPECFDVEENVTYPAIVHFGIRPPQLSYAGNP 2456

Query 656 EYQKAWREYVKYRHLMANMSKPSFKDKRKLEEKEQRLQEMRTQGRMKRNITVAISSEGFY 715

EYQKAWREYVKYRHLMANMSKPSF DKRKLEEKE RLQEMRTQGRMKRNITVA+SS+ +Y

Sbjct 2455 EYQKAWREYVKYRHLMANMSKPSFVDKRKLEEKEARLQEMRTQGRMKRNITVAVSSKSYY 2276

Query 716 RTGIMCDVVQHAMLIPVLTGHLRFHKSLDLLEESIGYRFKNRYLLQLALTHPSYKENYGT 775

RTGIMCD+VQHAMLIPVLTGHLRFH+SLDLLE++IGY FKNRYLLQLALTHPSYKENYGT

Sbjct 2275 RTGIMCDIVQHAMLIPVLTGHLRFHRSLDLLEQNIGYNFKNRYLLQLALTHPSYKENYGT 2096

Query 776 NPDHARNSLTNCGIRQPEYGDRKIHYMNTRKRGINTLVSIMSRFGKEHETVSNITHNERL 835

NPDHARNSLTNCGIRQPEYGDRKIHY+NTRKRGINTL++IMSRFGKE+ET+SNITHNERL

Sbjct 2095 NPDHARNSLTNCGIRQPEYGDRKIHYLNTRKRGINTLINIMSRFGKEYETMSNITHNERL 1916

Query 836 EFLGDAVVEFLSSIHLFFMFPELEEGGLATYRAAIVQNqhlallakklqleeFMLYAHGS 895

EFLGDAVVEFLSSIHLFFMFP+LEEGGLATYRAAIVQNQHLALLAKKL LEE+MLYAHGS

Sbjct 1915 EFLGDAVVEFLSSIHLFFMFPDLEEGGLATYRAAIVQNQHLALLAKKLHLEEYMLYAHGS 1736

Query 896 DLCHELELRHAMANCFEALMGALLLDGGIKVADEVFTDALFRQDEKLLSIWKNLPEHPLQ 955

DLCHELELRHAMANCFEALMGALLLDGGI VADEVF +AL+ +DE+L IWKN PEHPLQ

Sbjct 1735 DLCHELELRHAMANCFEALMGALLLDGGISVADEVFMNALYMEDEQLREIWKNYPEHPLQ 1556

Query 956 EQEPLGDRSCIDSYRVLKELTKFEDSIGIKFKHIRLLARAFTDRSIGFTHLTLGSNQRLE 1015

EQEPLGDR+CI SY VL+ELTKFE+SIGI+FKHIRLLARAFTDRSIGFTHLTLGSNQRLE

Sbjct 1555 EQEPLGDRACIASYPVLQELTKFEESIGIQFKHIRLLARAFTDRSIGFTHLTLGSNQRLE 1376

Query 1016 FLGDTVLQLICSEYLYRHFPEHHEGHlsllrsslVNNRTQAVVCDDLGMPKYAVYANPKA 1075

FLGDTVLQLICSEYLYRHFPEHHEGHLSLLRSSLVNNRTQAVVCDDLGM KYAVY+NPK

Sbjct 1375 FLGDTVLQLICSEYLYRHFPEHHEGHLSLLRSSLVNNRTQAVVCDDLGMTKYAVYSNPKV 1196

Query 1076 DLKTKDRADLLEAFLGALYVDKGLLYCEQFCHVCLFPRLQLFIMNQDWNDPKSKLQQCCL 1135

+LKTKDRADLLEAFLGALYVDKGLLYCEQFCHVCLFPRLQ+FIMNQDWNDPKSKLQQCCL

Sbjct 1195 ELKTKDRADLLEAFLGALYVDKGLLYCEQFCHVCLFPRLQIFIMNQDWNDPKSKLQQCCL 1016

Query 1136 TLRTMDGGEPDIPYYKVVEASGPTNTRVYKVAVYFRSKRLATSSGSSIQQAEMNAAKQAL 1195

TLRTM+GGEPDIP YKV+E+ GPTNTRVY VAVYFRSKRLAT++GSSIQQAEMNAAKQAL

Sbjct 1015 TLRTMEGGEPDIPIYKVIESIGPTNTRVYTVAVYFRSKRLATATGSSIQQAEMNAAKQAL 836

Query 1196 ENSRDLFPQLDHQKRVIAKSIKKQTGNELDNDSDRQHQEEKIKRPKYATPLQDESHLPKQ 1255

ENSRDLFPQLDHQKRVIAKSIKKQ G EL + + E+ +PK ++DESHLPKQ

Sbjct 835 ENSRDLFPQLDHQKRVIAKSIKKQKGTELKPELEEILAEQ--TKPKITNSMEDESHLPKQ 662

Query 1256 YRMHENISSDELPEDE 1271

YR+ E+ISSDELPED+

Sbjct 661 YRVREDISSDELPEDD 614

**Conserved domains**

**RIBOc domain-containing protein**

**
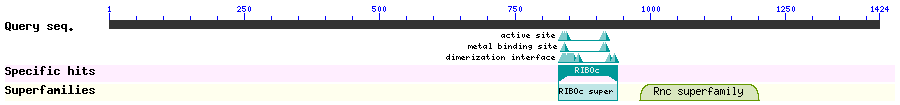
**

**Pasha**

**>TRINITY_DN28163_c0_g1_i6 len=2921** GAAAAGTTAACTTCATTTTATTTTAGTCATATAAAAAGCCGGACTTTCAATAAAAACTGA

AAAATAAATTAAAATATCAAAGCTTCAAAGAAGTACTTTTGCACTGGTTTCGTGTATTTT

ACACTAAATACAAATTTTAAAGCAGAATCCATTGCGAAAGTTGCTTCAGTCGAATTTTAA

CACAACGCTGAAAATAAAATCCGATAAAGAAAGTCAGGTGCATTAATCTGGGCTAGTTCT

GAAATAACCAATGTGACCAATCCAATTTGTATGAAAAATCAATTGCAAACGTTGGATTCC

ATCAGGTTGGGTGTTTTAAAGAGCAGCCTTTAATCGACAAAACTTCTAAGCTCCATCAAG

CTTGCATACTCGTACACAAGAAATAAAATTGCCTTCTCAAAGAATTTTATAAAATATAAA

AGCGAGAAACAACAATGTCCAGCGAAAGTGATAACACGCAACGGTCGCCCTCGTCGCCAC

CATCGCTCCCATCGTCACCACAGCCCCACAAAAGAATGCGTCTTGATGAGCAAACGCTGC

ACGAGGAAGCCACCGCCATGAAGGAGAAGGGTTTCTCGTTGAGTGCCATCGAGGAAATGA

AAACACGTGTGGAGGAGAGCAATCAAAGCGGAGTGCTCGATCAACAGTTGCGACAATTTC

AAGTGTTAGACGAAGTACAGGGTTCCGGCTCTGAAGATTCGGACATTGCCAGCGAAAATG

GCGATCAAAATCATGAGCACGGTGATGGTTATGAATCATCTGATTTGGATATAGACGATA

ATGAGATTGAGAATTTGCTCAACGAGAATTTACCTGATGATTTGAAAGAACCAAAGAAGC

CAAAATATGAAGAACGCTTTAAAACGGTTTTGGAGGAGAAAGGTCATAACCATTTTGAAG

TGCTGCCGGAGGGTTGGGTTCAGGTGACACATAACAGTGGCATGCCACTTTTTCTGCATA

AACAATCACGTGTCGTTTCAGCATCGAGACCATATTTCCTTGGTCATGGCAGTGTGCGAA

AACATGCAATACCTTTGGGTGCTATACCATGCCTTAATTATAGGCGAGCATTGGAGGAGG

AGGAGGCGGAGATGAAGCGTTTGAATGAAGCATCAACAAAAGAGGTTTCGGTAGATGCAA

CCCGGGATGCTGACGAGGCTATAGTGGATAATATTCCCAAATGTCCATACACTGGCGCTA

GTAGCAGCGCGATTACCCCTACAGAGTCAAGTCAGTCTACACAAGTTTCATCTTCGACAA

ATGGCATCGCGGTAAATAACGAAAATATGGACACGAGCGACCTGACAATTTCGACACAGT

CTGCTGCTACACTTACGACTCCAGCATCAACCTCTACAACTGCTACACCCACAGCGACAG

CGACAACGACACCGGGCGACTTGGCCGCTAATATTGCGGCATTGAAGAGCCTAGTGCCAC

CTGCAAAAATTGTCACCGTCACCGAGAATACACAAAACGAGTCGTTGACGCCAGAAAAAT

TGAATCAGTATTGCACCAAATTGTTTAAATTCAAAGTGATACGTGTTTTGCGTTTTCGTT

CGTGGAATGCGCGACGCAAATTCACGAAAAATCGCAAGCACATTAAAAACTTGCAACGTC

CGACGCTGCCCGATGGCACTAAACTGATAAAATTTCCAATATTGGCACCCAGTGGCGATG

CAAGTGCCAATACACGCGGACGTAAAGAATGGATTATGAATCCGAATGGTAAGAGTTATG

TGTGCATATTGCATGAGTATGTGCAGCATGCACTCAAAAAGCAGCCAACATACGAATTCA

AGGAGTTGGAAAATGCGGCAACTCCCTACTCGGCAACTGTATCGATCAACGAACTGAAAT

ATGGCACGGGTTATGGCACCAGCAAGAAGCAAGCGAAGTCTGATGCTGCACGCGAAACAC

TGGAGATATTGATACCGGAAATGAAGGACAAGATTACGGGCATTAAGCAGGACAAAAATG

CGCCGAAAAGCAATCACAAAGACTTATCAGTTTTCGATGATATTAAAATTCAGGATCCAC

GCGTTGCTGAATTTTGTAACAAAACAACGGAGCCATCACCGCATGCTATATTGTTGACCT

GCTTGCAACGTAATTTCGGTCTTGGCGATGTGCAGATTAATTATGAAATTAATCGTACGA

AAAATAAAAAGAATGAATTCACCATGACTGTGGGCAAACACACGGCTAAAGTGTTATGCA

AGAATAAACGCGAGGGCAAGCAACTGGCCTCGCAAGCAATACTACAGATTTTACATCCAC

ACATTAAGACATGGGGCTCTCTGCTACGCCTATACGGCAATAATTCAATTAAGACTTTCA

AAGAAAAGAAGCTAGAAGAACAAGAAATCACTGTACTGCAAAGCAAAGCTGCCATAAATC

AGCCGAATTATGCTATTCTTGACAAACTCAAGGCAGAAATGCTTAAATTGGCTGAAAAGA

ATAAATCCGTTAAATCTATGGGCACCTTTGTGCCGCCCAGTGATGTTGATTTACCCTCAT

CATCAGGCTCTAATCTCAATAATGTGGAGCTATAAGCGCATTTCTTACATTTTCGTAAAT

GTGTGTAGGTTTCGACTGAACTGAAATATGACTGAACAATGAACAACTAAAATTTCTAAT

GCGATTGTTTTGAATGAAACCGCCGAAAAAATTGAAGCACAAATGAACACTAGAAAAATA

TATTTAAATACTTATCTGTGGTAGCAAATGAAAAAAGCTGGAGAGAACATAAGCAAAACT

AATAATTGAACTACTAAAATGGACAGTTACAAAATAAATATGGGGATAAATTGCCAAAAT

GTGCATCAAGCAGCTTAAGGGGGGAGCCTGGTTTATAAGGTAAAAAAAAATGATTTTTTT

TTTTCGTTTTTAGCGACTCTGAATATATTTCAGAATATTCA

**tBLASTn(First hit)**

Score = 809 bits (2090), Expect = 0.0, Method: Compositional matrix adjust.

Identities = 413/589 (70%), Positives = 454/589 (77%), Gaps = 66/589 (11%)

Frame = +3

Query 56 NLLDEKLPEELRESKQPKYEQRFKTVLEEKRLNHFEVLPEGWVQVTHNSGMPLFLHRKTR 115

NLL+E LP++L+E K+PKYE+RFKTVLEEK NHFEVLPEGWVQVTHNSGMPLFLH+++R

Sbjct 792 NLLNENLPDDLKEPKKPKYEERFKTVLEEKGHNHFEVLPEGWVQVTHNSGMPLFLHKQSR 971

Query 116 VCCASRPYFLGTGSARKHAVPLGAIPCLNYRRALEEES------------EVEQQKTDGA 163

V ASRPYFLG GS RKHA+PLGAIPCLNYRRALEEE EV T A

Sbjct 972 VVSASRPYFLGHGSVRKHAIPLGAIPCLNYRRALEEEEAEMKRLNEASTKEVSVDATRDA 1151

Query 164 PPVAV-----CPMAGAS----------------------ALPAEAMDVEGKENNEGST-- 194

V CP GAS A+ E MD + S

Sbjct 1152 DEAIVDNIPKCPYTGASSSAITPTESSQSTQVSSSTNGIAVNNENMDTSDLTISTQSAAT 1331

Query 195 ----------------------DSKTQPDGITALMPAAKIVTVNENTQKESITPEQLNTY 232

D + +L+P AKIVTV ENTQ ES+TPE+LN Y

Sbjct 1332 LTTPASTSTTATPTATATTTPGDLAANIAALKSLVPPAKIVTVTENTQNESLTPEKLNQY 1511

Query 233 CQKLFKFKVIRVLRFRSWNARRKFTKNRKHIKNIQRPTLPDGTKLIKFPILANAGEGSTN 292

C KLFKFKVIRVLRFRSWNARRKFTKNRKHIKN+QRPTLPDGTKLIKFPILA +G+ S N

Sbjct 1512 CTKLFKFKVIRVLRFRSWNARRKFTKNRKHIKNLQRPTLPDGTKLIKFPILAPSGDASAN 1691

Query 293 PRGRKEWIMNPNGKSFVCILHEYVQHALKTQPTYEFKELQNAATPYSATVSVNNLKYGTG 352

RGRKEWIMNPNGKS+VCILHEYVQHALK QPTYEFKEL+NAATPYSATVS+N LKYGTG

Sbjct 1692 TRGRKEWIMNPNGKSYVCILHEYVQHALKKQPTYEFKELENAATPYSATVSINELKYGTG 1871

Query 353 YGTSKKQAKSEAARETLEILIPDVKDKITGNNKDQKSGTAKKAQSDLSVFDDIRIEDPRV 412

YGTSKKQAKS+AARETLEILIP++KDKITG +D+ + K DLSVFDDI+I+DPRV

Sbjct 1872 YGTSKKQAKSDAARETLEILIPEMKDKITGIKQDKNA--PKSNHKDLSVFDDIKIQDPRV 2045

Query 413 TEFCNKTTEPSPNAILLTCLQRNYG-SDVQISQEINRTANNKNEFTMTVGKHTAKVVCKN 471

EFCNKTTEPSP+AILLTCLQRN+G DVQI+ EINRT N KNEFTMTVGKHTAKV+CKN

Sbjct 2046 AEFCNKTTEPSPHAILLTCLQRNFGLGDVQINYEINRTKNKKNEFTMTVGKHTAKVLCKN 2225

Query 472 KREGKQLASQAILQILHPHIQTWGSLLRLYGNNSIKTFKEKKLEEQEITVLQSKAAVNQP 531

KREGKQLASQAILQILHPHI+TWGSLLRLYGNNSIKTFKEKKLEEQEITVLQSKAA+NQP

Sbjct 2226 KREGKQLASQAILQILHPHIKTWGSLLRLYGNNSIKTFKEKKLEEQEITVLQSKAAINQP 2405

Query 532 NYAILDKLKSEMLKLCAKQESVLTMGTFVPPSDVDLPTSSGSNLNNVEL 580

NYAILDKLK+EMLKL K +SV +MGTFVPPSDVDLP+SSGSNLNNVEL

Sbjct 2406 NYAILDKLKAEMLKLAEKNKSVKSMGTFVPPSDVDLPSSSGSNLNNVEL 2552

**Conserved domains**

**WW and DSRM domain-containing protein**


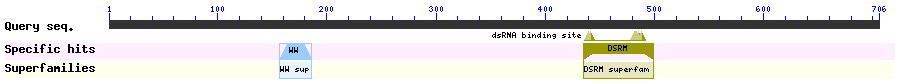


**Exportin-5**

**>TRINITY_DN23399_c0_g1_i2 len=4735**

AACCCTGTATTGCACAAGGTGAGACGGGTTTGCTGTGTGCTGTGTGTTGTGTGCTTTGCT

GTGTAAAATCGCTACAAATTAACTTTAGCACATTTTTTGAAGTCTGATTTATAAAAAATT

TACTTAAGATAATTTTTGCATAAAAGAAGACATACAACTAAATCGCCCGTTGAAACATAA

ACCCCCAACACATGAAAAAACAATATCGGCCGGCGAAGAAGAAAAAAAAAACGAAAAGCA

AAGAAATTAGAATTCTGTGTGAAGGTGGTAAAGAAGGAGGATACGAAAGTTGGACATCGG

AAACCGCATACACCAGGCATTTCTACAGAGTTAAAAAGAAAAAATAAAATTTTTCAAACT

TTTTGTGTGTGGGCTCATAGGATACGTGTCATATAGGTCGGTCGAAGACAAAAAATTGAT

AATTGAAAGTGGCGGACCACCGGCATTAAGATGACACAAAGTGGAAATGTAGCCGCGCTC

GGAGAAGAGCTTGCACAGGCAATTGAATTAATTATGCGTCCAGATACAGCCCAGCAGTCG

CGCATGGAAGCCTACATGGCTTGCGAACGATTTAAGGAAGAATCACCGTTATGTGCACAA

GTTGGCCTTTACTTAGCCAGCGGTCAACAATTCGGCCAAAATGTCAAGCATTTTGGCTTA

CAACTAATGGAATACACAATTAAATTTAAATGGAATAGTATTTCACATGAGGAAAAGCTT

TTCATAAAAGAAAACGCTATGAAACTTATTCATTTTGGTGTTGGTCCGGCCGAAGATGCA

AGCTTGGCACACCTAAAGGACGCTTTATCGCGCATAATTGTGGAAATGATCAAACGCGAA

TGGCCACAGCAGTGGACGACATTGCTATCTGAGTTATCTGAAGCTTGCAATAAAGGCGAA

CCACAGACGGAGCTGGTGCTTTTGGTTTTTCTACGCCTCGTCGAGGATGTGGCATTGCTT

CAGACCATAGAATCAAACCAGCGACGCAAGGACATGTATCAGGCGCTTAACAACAATATG

AACGACATTTTTGAATTTTTCCAACGACTTATCGAGCTGCATGTTACATCCTTTCGTGAG

GCCACTGCGCACGGAAACTTCCAGAAAGCGAATGCACACGGCCGCGTTGTAGAAGTGGTG

TTATTAACACTAACTGGATTCGTGGAATGGGTTTCAATGAACCACATCACCTCCAATAAC

TGCAAGTTGCTACAAATTCTTTGCATTTTGTTGAACGACAAAGCATTCCAATGCAACGCA

GCTGAGTGTCTCTCACAGATTACTAATCGAAAGGGCCAGGTGAAGGAGCGTAAACCGTTG

CTGATGTTGTTCGGCGAAGATCCGATGCGTTATATCTTTACGGCCAGCCAAATGTTGCCA

GATGCAGCCGTCGCTGGCTCTTTAGAGCAAAACCATAATTTCCTTAAGAAGCTTCTGAAT

ATGCTGAGCGGTTTGGGTCAGCAAATCGTAATACTGTGGGGTAAAGAAGATGGCAGCATA

CAGCGGCCACAGCACTTTGAAATATTTCTAGAATGTTTACTTCTGCTCACACGCCACCCC

TCGTTGACGGTGGCGCATGATGCATCGCTAATCTGGAATATGCTACTCAAACATGATGGC

ATTTCCAAGGATGCAACTGTGGTCCCATACATTCCAAAACTGATACACGTTATCGCGCCG

CGCATCATTAAAACCCAGTATCCCAGCACGCGCTCATTACCCACCTCAGTGTCGACGGCC

GCATATATCTGCTTAGAATACGACAGTGAGGAGGAATTTGCGGTGTTCTACTACCGCTGC

CGCACCGATTTTTTGGAGGTATTTCGCCAGTCAACCCTCGTTCAACCTGTCGTTACGTTC

ACGTATTGCGAACAGTGGCTCAACGCTCGTCTGGCCAAAGCACATACCGAACGCGATAAT

GTTAATTGTTCTGTACATGATCCCGTTTATATGGAATGGGAAGCTTTGGTTTGCGTTATC

GATGGAGTTCTTAGTCGTATACTCTTGGTGTCAGAACGGCCATCGGTGCAATCAGGACTG

CGTTTGCTGGAAGAGTGCTTGAAAGTTGAGACTAGTAATCCGCTCTTACTCTCCATATTG

TTGTCCTGCATTTCGGCTCTTTTTGTGTTTCTCAGTATGTCTTCATGTCAGATAACACCG

AATAATTGCGTAGCTATGAGCGGCGTAGCATTGCTACCACGAGTGTTGGAGCGTATTTTC

GAAGCACTTGTATTTCGGAATCCCACCGAGCCAAGCACTTTAACAACGCGCGCACAAGCC

ACAAAAAATTTGCGTCGTCACGCCGCTTCCCTCATGGTTAAACTGGGTCATAAGTATCCA

CTGTTACTGCTACCAGTTTTCGATCAGATTGACACACATGTTAAGGTCCTGCTCGATGAT

CCGCGGCATGCACTAGGCAAAATGGAACGCACTACACTACAGGAGGCTCTAATGTTAATA

TCGAATCATTTTTGCGATTACGAACGGCAGACTGCTTTCATTGCAAACATTATGAAGAGC

ACGCTTGGGCATTGGTCTACTTTTGCGGAGGTGTTCAAATCGGCATATACCTTTATACAG

TTCGTGGGGCTCGATAAACCGGCGGTAACTGCATTTCAATCAGATCCACTCTGCATAAAT

CGTGGTATTTTGTTGGACTCACTAAACGTGGTGCTAGCTGTTATTAAACGTTGCACATGG

CCTGATGATCCGGATCGTGCATCCCGCGGCGGATTCGTTGTAGGCTTCACCGAGCTCGGC

AACCCCATTTGCCGCAATCCAGCCACACCACATATAATTCCCCTGCTGCCACATATACTT

GCATTAATGCGCGTGCTCAACGAGCTCTACCGCCCTCAAGCGAAGGCTTTGCTCTCAGAG

GATTTTCGTAATGTATACTCCATGTTAGAGCACGAGAAAAAGACTTTGCTGGGCGTGTGC

ACACCACCAGCTGACCCACTTGACCCAACTGTAAAAACGGTGACAAGTACTGTGGACCGC

ATGCAGCAGTTCATGTCATTGTTATACGAAGGCTGCTATCACATGATGGGCTCTGCGGGA

CCAACTCTAGGACGCGATCTCTATCAGTTACAAGGTATTTCTGACGCGTTAATCAATACC

GTTTTCGCTTCACTAGAAGATGTACCAGATTATCGACTGCGCCCCATCGTGCGTGTTTTC

TTCAAACCGTTTGTATATTCTTGCCCGCCCGCCTTCTACGATACGGTACTGGTGCCGATA

TTTGCACACTTTGCTCCTTTCATGTGCTATCATCTGGTTCAACGTTGGACATATATATCG

TCGTTATATGAATCGGGTCAGTTGAACGAGGAGTCGAATGATACGCAAGAGGTACTGGAG

GATATGCTGAACCGTTCTTTAACGCGAGAATATCTGGACGTGTTAAAAATAGCGCTAGTC

GGTTTTGGCACTGACAATGTGCATGCTGCTGCAAATGTAACCGACGTTGCTATGGAGCCA

GAAGAGCACTCCATGGATGGCACAGCACATTCTCGTGCAGCGCAATCGGCACTGCTATCA

GATATCATCAGTGACTTGGGTGCGAAACTGTTACGCAATGATGCGACCGGAAATCATATA

CTAATGACACTGATGGCTGCGCTCTCTTGGCAAGATAGCGCCTGCAGCATGAAGGCGGTT

AATGTAGTGGCGCCGGTGATGCGTTTCCTGGCCACAAACGAGATACAGTTAATGGACCAA

CACAAAGCAATGACCGCGTTCCATGCGGTTTTGCAAGGCCTCCAGGTGCACGGTATGCAT

GAAGCCAATCAGGCTGGACTCATAACGCTCGGTGTGCAATTCTACGAGCTGCTGCGTCCC

AAATTTCCTATACTTAGCGATGTGCTACGAAATATACCAAATGTCAGTGGAGCTGATGTA

CATAAATTTGATGAGAAAGTGAGCGTGGCGCCCTTGAAGGGCAATAAAGTGGACAAAGCT

AAGAAAGATATATTTAAGAAGATGACCGCACGCTTGGTGGGACGCAGTGTAAATCAGATG

TTCTGCCGCCAAATCGAAATTCTCAATTTGCCGCCAATGCAGGCGCACGCACCTAAACCC

CATACGGATATTGTTGATATTACAGAAAATGCGGGATTGACGCAGCTATTCCGCACGGAA

AAGTGACCAGTAAAAAAAGCAGGGCATGCAACTGTAGAAAAATTTTGAATTTTATTAAAC

ACTTCTCCCGCTCTGTTACTGCGCCACAAATAGAGGTTTCGTTGATGAGTTCGTATTTGC

AGCTTGCATATAAGGGAAAGGAACATTATGCAGTTCAAACTACTACATACATATTTACTT

ATTATTGGAGGAAACGGCCGTTCGGCAATATTTATGCGCACTTTTCCATCAACTGTACTA

CGAAAAGGAGAACGAAATTATTTAGCTACATCCATATGAAATAATTTATTAATTATAAAA

ATATTATATATGCGTATATAAATCCTATGTTAATTTTGAGCTTTCAAATAATTTCTATTT

ACGAAAATTGGCATTGTTTTTATGAATATTTTAAAATATTATGTAAGACTTTAGAAATTT

TTATTAAGATATTATAAAAGAAGTGAAGTACGAACGAAATAAACAAATTGAGTTTAAGCC

GAGTGAAACTTGAATAGTACATACAAAAATATGTGGATGTTAAATATGGCAGAGGTGGTA

AATTTGTCACTTTGCATTGGAAAACAAGTGGCATAATTCAGAAGTAAAACAATGG

**tBLASTn(First hit)**

Score = 1634 bits (4232), Expect = 0.0, Method: Compositional matrix adjust.

Identities = 850/1265 (67%), Positives = 1021/1265 (81%), Gaps = 30/1265 (2%)

Frame = +1

Query 5 GRRQQL--------AMAMAQHggtealaaelataVDLIMHPMTQPQARLEAYMACERFKE 56

GRRQ++ + M Q G AL ELA A++LIM P T Q+R+EAYMACERFKE

Sbjct 400 GRRQKIDN*KWRTTGIKMTQSGNVAALGEELAQAIELIMRPDTAQQSRMEAYMACERFKE 579

Query 57 ESPLCAQVGLFLASSPQSNQQVRHFGLQLIEYTIKFRWNCITHEEKVYIKDNAIKMLNVG 116

ESPLCAQVGL+LAS Q Q V+HFGLQL+EYTIKF+WN I+HEEK++IK+NA+K+++ G

Sbjct 580 ESPLCAQVGLYLASGQQFGQNVKHFGLQLMEYTIKFKWNSISHEEKLFIKENAMKLIHFG 759

Query 117 VGPAEDRTLLPTKDALSRIIVEMIKREWPQQWSDLLPELSQACTKGEAQTElvllvflrl 176

VGPAED +L KDALSRIIVEMIKREWPQQW+ LL ELS+AC KGE QTELVLLVFLRL

Sbjct 760 VGPAEDASLAHLKDALSRIIVEMIKREWPQQWTTLLSELSEACNKGEPQTELVLLVFLRL 939

Query 177 vEDVALLQTIESNQRRKDMYQALNNNMNDIFEFFLRLVEQHVTAFRETTRQCNFTKANAH 236

VEDVALLQTIESNQRRKDMYQALNNNMNDIFEFF RL+E HVT+FRE T NF KANAH

Sbjct 940 VEDVALLQTIESNQRRKDMYQALNNNMNDIFEFFQRLIELHVTSFREATAHGNFQKANAH 1119

Query 237 SRVVEMVLLTLSGFVEWVSIQHIMSSNGKLMHFLCILLNDKAFQCNAAECLAQITNRKGQ 296

RVVE+VLLTL+GFVEWVS+ HI S+N KL+ LCILLNDKAFQCNAAECL+QITNRKGQ

Sbjct 1120 GRVVEVVLLTLTGFVEWVSMNHITSNNCKLLQILCILLNDKAFQCNAAECLSQITNRKGQ 1299

Query 297 AKERKPLLQLFNEEPLRYIYQASQIPPDSSSALAIEQHHNFLKKLLQVLNGMAQQLVALW 356

KERKPLL LF E+P+RYI+ ASQ+ PD++ A ++EQ+HNFLKKLL +L+G+ QQ+V LW

Sbjct 1300 VKERKPLLMLFGEDPMRYIFTASQMLPDAAVAGSLEQNHNFLKKLLNMLSGLGQQIVILW 1479

Query 357 GKDDAT-QRPVHlevllecllllvQHPSLTVAHGAALIWQLLLKHEPCSKDFATVNYIPK 415

GK+D + QRP H E+ LECLLLL +HPSLTVAH A+LIW +LLKH+ SKD V YIPK

Sbjct 1480 GKEDGSIQRPQHFEIFLECLLLLTRHPSLTVAHDASLIWNMLLKHDGISKDATVVPYIPK 1659

Query 416 LIHTIAPRIVKLpypaststsspaslsteaYIRLEYDSEEEFALYFFRCRTDFLEIFRLA 475

LIH IAPRI+K ++ S P S+ST AYI LEYDSEEEFA++++RCRTDFLE+FR +

Sbjct 1660 LIHVIAPRIIK--TQYPSTRSLPTSVSTAAYICLEYDSEEEFAVFYYRCRTDFLEVFRQS 1833

Query 476 TLVQPLVTYGYCEQWLQQRLRNAVAEADVEVKSGNISCSVLDPVYLEWDALVSVLDGVLS 535

TLVQP+VT+ YCEQWL RL A E D N++CSV DPVY+EW+ALV V+DGVLS

Sbjct 1834 TLVQPVVTFTYCEQWLNARLAKAHTERD------NVNCSVHDPVYMEWEALVCVIDGVLS 1995

Query 536 RILLVAERPSVPAGLRLLEECLKLETINPLTYSILLSCISALFVFLSMSACQITATNCVA 595

RILLV+ERPSV +GLRLLEECLK+ET NPL SILLSCISALFVFLSMS+CQIT NCVA

Sbjct 1996 RILLVSERPSVQSGLRLLEECLKVETSNPLLLSILLSCISALFVFLSMSSCQITPNNCVA 2175

Query 596 MSGVSLLPRVLDKIFRALVLKPPNELEKV--QAKSAVNLRRHAASLLVKLAHKYPLLLLP 653

MSGV+LLPRVL++IF ALV + P E + +A++ NLRRHAASL+VKL HKYPLLLLP

Sbjct 2176 MSGVALLPRVLERIFEALVFRNPTEPSTLTTRAQATKNLRRHAASLMVKLGHKYPLLLLP 2355

Query 654 VFEQINGHVELLLKEPGQHQLCRLERTTLQEALILISNHFCDFERQTLFIEHIVQDKRTE 713

VF+QI+ HV++LL +P +H L ++ERTTLQEAL+LISNHFCD+ERQT FI +I++

Sbjct 2356 VFDQIDTHVKVLLDDP-RHALGKMERTTLQEALMLISNHFCDYERQTAFIANIMKSTLGH 2532

Query 714 WLAFGDALKSPLDFMSFVGLDKPPSYAVEGSPTLQNRSRLLDALHVVLGVVKRCTWPDDP 773

W F + KS F+ FVGLDKP A + P NR LLD+L+VVL V+KRCTWPDDP

Sbjct 2533 WSTFAEVFKSAYTFIQFVGLDKPAVTAFQSDPLCINRGILLDSLNVVLAVIKRCTWPDDP 2712

Query 774 DRAQRGGFVIGCTELGNPICRNPATKhvvpllshvlslMRVLNELFAPEALLALSDGYRG 833

DRA RGGFV+G TELGNPICRNPAT H++PLL H+L+LMRVLNEL+ P+A LS+ +R

Sbjct 2713 DRASRGGFVVGFTELGNPICRNPATPHIIPLLPHILALMRVLNELYRPQAKALLSEDFRN 2892

Query 834 IHGMLEHEKKQLMGICALPTDPLDTTIRSEPTPFEKMQTFMMMVTEGCYHLMGSAGPSLG 893

++ MLEHEKK L+G+C P DPLD T+++ + ++MQ FM ++ EGCYH+MGSAGP+LG

Sbjct 2893 VYSMLEHEKKTLLGVCTPPADPLDPTVKTVTSTVDRMQQFMSLLYEGCYHMMGSAGPTLG 3072

Query 894 RDLYQLLGLADAIVTNVFSRMDVVPDYRLRPIIRVFFKPFVYSCPPSFYDSVLVPLFAHL 953

RDLYQL G++DA++ VF+ ++ VPDYRLRPI+RVFFKPFVYSCPP+FYD+VLVP+FAH

Sbjct 3073 RDLYQLQGISDALINTVFASLEDVPDYRLRPIVRVFFKPFVYSCPPAFYDTVLVPIFAHF 3252

Query 954 APLMCERLTRRWLYIASLYESGQLNGEVNDTQEVLEDQLNRTLTREYLDVLKIALVGGQI 1013

AP MC L +RW YI+SLYESGQLN E NDTQEVLED LNR+LTREYLDVLKIALVG

Sbjct 3253 APFMCYHLVQRWTYISSLYESGQLNEESNDTQEVLEDMLNRSLTREYLDVLKIALVG--F 3426

Query 1014 GADHvaaganansnsvaMENEEHSMDSAPQSRASQSALLSDIISDLGGKLLRNGHIGNYV 1073

G D+ A AN VAME EEHSMD SRA+QSALLSDIISDLG KLLRN GN++

Sbjct 3427 GTDN--VHAAANVTDVAMEPEEHSMDGTAHSRAAQSALLSDIISDLGAKLLRNDATGNHI 3600

Query 1074 LMTLLKAIAWNDGMCSMKAVNIAAPVMRFLAAE--KLMDENKAVTAFTAVLQGMQVHGQH 1131

LMTL+ A++W D CSMKAVN+ APVMRFLA +LMD++KA+TAF AVLQG+QVHG H

Sbjct 3601 LMTLMAALSWQDSACSMKAVNVVAPVMRFLATNEIQLMDQHKAMTAFHAVLQGLQVHGMH 3780

Query 1132 EANQSGLVTLGVQFYELLRPHFPILSEVLQHIPSVNAADIQKFDEKIAVAPVKGNKVDRA 1191

EANQ+GL+TLGVQFYELLRP FPILS+VL++IP+V+ AD+ KFDEK++VAP+KGNKVD+A

Sbjct 3781 EANQAGLITLGVQFYELLRPKFPILSDVLRNIPNVSGADVHKFDEKVSVAPLKGNKVDKA 3960

Query 1192 KKDIFKKLTAQLVGRSVNQLFRHEVQIANLPPMQSHKAKGAMGTTADIMDSNQNASLARL 1251

KKDIFKK+TA+LVGRSVNQ+F +++I NLPPMQ+H K DI+D +NA L +L

Sbjct 3961 KKDIFKKMTARLVGRSVNQMFCRQIEILNLPPMQAHAPK----PHTDIVDITENAGLTQL 4128

Query 1252 FGPEK 1256

F EK

Sbjct 4129 FRTEK 4143

**Conserved domains**

**IBN_N and Xpo1 domain-containing protein**

**
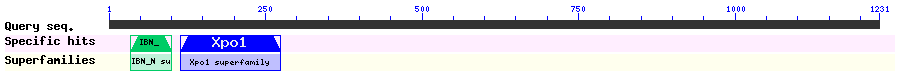
**

**Dicer-2**

**>TRINITY_DN32516_c1_g2_i1 len=5709**

GATGTTTTTCCAAGGCAGATGGTATTCGATGAGTAACTTTTTCATGGCAGAAATACACTC

GGAAGATTGCCATTGAATGGTAGCCAGTCACGTAAAAAACCCATTCGGCTAGGCCAACCA

CAATTTATTGCACTTCAAATAAAACTTTCTAACTTCAAGATAGATGCTCACGCAATGCTA

TAGATTTAAATGTATCTTGCTAAACACTATCCACTTTCAAAACCACTTTTTCTATACCTA

ATTTATCCTATTTGACTTTTTAACACACACAAATTAAGTTTTGCATTTGAGTACAAGCAG

AAATACTTAACTCGAATATTTAGCTAATATCTGTAGTGCATGCTTAGCAGCGGCCTTTTT

TGCCTGTTTACCATTCAAACCAAAACCGTTTACTTCAACACTTTTGTCAAGACATGTAAA

CTGGCATTTCACCATGTATGTTTCATTGTCTGAAATCGGCTTGCTGTACTTGGGATTTGC

ATGTTTATGATCATTCAACTGTCGCACTGCATCGATGGGCACTTGCTGGGCAAACTGATT

GAATTCCTGCTCTAGCAAGCCGTAAATGACATGCCAAGTTGTACGCAGGTCACGACTATC

GAGATAGACTGCGGCAATAAGTGCTTCCAGAATATCACCTAACGCTTTAGGAACATCAAC

ATTATCCGATATATTATAAGTGGCTCCTTCCACCTCGTTTTCAGTCGTAATATTTTCCTC

TGACAATATGTGCACTTGATCGGTAACATAGTAATTATGCTTTTCTTGATATTTGTCGAA

ATTCTTGATAGACTCCGCCAGCGAACTATTTTCAGCCAGTAAAAATAAGTGGAAGCGATG

ACGCACACAGACGCAACCCAAAGTTATGTTATTGACCAGTGCCGATCGCAAATCGGTGAG

CATACCGGGGTCCATGTGACTATAGCGTTCAAAGATATAGCATGAAACCATAAAGTCGAG

TATGGCATCTCCAATAAATTCGAGTTCTTGATAGCAACCAGTGATGCGATTGGTCGGATA

GGACGGATGTGTTAAAGCCTGAAGTAAGTACGCCCGATCACGGAATTTATAGCCCAAATT

CTCTTCCAGTTTAGGATAGTTGATAATGTAGCTATCGACTTGGCGAGAAGATATATTTGT

ATGCAGCAAAGGGCCGCCTAATTTCAGGTCCATTAAACGTGTAAAGGATTGGTCTGCATC

AGGTTTACATATGCCAAAAAACTCCAACATACGGAAGGTATTAATAATACCGTAATTTTT

TACACAAACTCCAAGTATGGCCTCCAAAGTGTCGGCAACGACTTTATCGCGCAACACCTC

CTTGTATGCAAACAAATTCAGTCCGCTGTCAAGTCTCTCTTCTTCCGCCAGTTTATGTGC

TTGCCTGCTACTAGCGTTCAATTTAGTTAGATGCACGGCAGAACAACAACCGCTGAGAAT

TTCATCGTCATCCAGTTCGATACCATACAAATCACTCGGTGTTAAGTTTTCCAATACTTG

TTCATTATTTTTTAAAAGTTCCAGAAGGTTGCGTGGCAAACTGATTAATGGAGGCAACCA

CTCATGTGGGGGCTTGAATAAATATCCACAAATACATTCCGGTATATCGGTCTGAAGCAT

ACAATAAATAAGATTGCGATTTGATACTAACTTTCCTTTAATTTCAGTTAGAAATCCCTC

GTGCCATTTTGGATACTTATGTGCCAAATAAAGAGAGATAGAAAATTTTAGGAATGAATC

GCCTAAAAACTCAAATCGCTCCATATCGAAAACGTCATTTGCGCCGGCCGTTGTAATGGC

GGCCAAAAAGTCTGCTTGCTCAGCGGTCTTCAAAGTCTGCTCACTCAATGTTAGTTCTAA

TATATCGAGGCGCCGTTTATCAGCTAATGGCACGTCGCATAGCGAAATACTACACAAATC

CTTTCCGCGTACTGGTGAACCGGCTGCATTCTGATTTGGCATATTCAACTGCGACTGCGA

CAACGACCATTCACCTTCAGTTAGTTTCAGTGCATCTAATTCTATTGCCAAGCCGCTTAT

AAATTTGTAGTAGTAATCTAATTCAACGGGGAATAACGACTCAGGCTTCCGAGAAAAGTC

TTCGGGCTCCAAATACTCCTTCCATGCCACATCATCTATATGAAGCATCTCAAATTCGGC

CTCTATGGAGCTAGTAGGCATAGCCGGTAACAGTTTTTTCGGTTCATCTGGCTCCTCCAT

GGCATTGCCGTCAAAATCTACTGCACGCTTTAATGACCCATCGACAGCTAATTGCTTTGG

CTGGTATTCCACACTATCCACTATGCCCAGAAATGTATTAACACGACATCGCAAATTTTC

TGCATGCAACAAGAAATGCACCCGATGTAAAATGCTCGGCAGAAACAAAGCTTTCAGCCA

CATATCACCCGGATACATGAAGTTGTGACAAAGCTCAGGTATTAGGCGTATGGCACAATT

ATTTTTATGCACATTTGATGATTTGCCAACAGCATTTATGAAGAAATTACGTCGCGAAGT

TAGAGCGCGCACTTCAAGCATAAATTGATTTGGTTGCACAACGCAATCCACTTCGTTCTT

GTATTTGCAATCAATGAAATCAACATAGCTTGAATAATCGTTATTGTCGAAGGGACTTAA

TGGTGTACGATCGGTTAGTACCTTGGTTACCACAAAACGCTGATTTTCTCTGCCCGAATA

CCATTTGTTAACCACTTTGCCAATATAGTCCTCCGGCTTGTAAATCTTTTGCTTCCTTTC

GCTAACGCTGTATTTACGCTGTGGTAATAGACGTTGGAAGTTTTCTACCAACTCCCAGTC

TATTTTACCCGCATTGATCGGTACTATCAGGTATGAATTCTCTTGGTTACGCTGATCTAG

TACGAAGAAAGTTTTCCAACACTCGAGCAAATCACGGAAGAGCATCAAATGGAATTTACA

TAATTTTTGTAATTGCTGTTCATTGGTTATGGTCAATGTGAGCGGTTGTGGGGCTATTTT

CACCGAAATTTTACCTTGATTCATGAAGAGAGGCATTTCTGCGAGTGGTGGTATGCGTTT

ACGCGACAAAATTGCATAGTTACGATTGCTCTGCAATAGTGTACTTATATGTAGAGAGTA

GTCATTGATTTCAAAGTGGGGGTACGCATGAATCTCGTAGGCATAACAAACTTCATTTAG

ATGCGGGATAGCGTTTTGGAGTTCATCCGGATATTTGCGATTGTACAAGCGCCGCTGTTG

TTTGCCCGCTGTTTTTGAGGTCACATCGTCATTAAATTTTTTCCAATGATCGAAGAGTTC

TTCCGCTACCTTCTGTACACATTCACGCTTCGTCACGGGCAAGAGTCGGTCATTTAGTTC

CCCGTGTTCGTACAATTTTATACACGCCTTAAAAGCAGCAGATATTTTTGCTTCCTTGGT

AGTAGGAAATGTATCGCTAATTATTGTCTCCTTCACTGAAGATTGCAGCGGCATTTTTAG

CTCTACAGCTATTCCTCTCTCAGTACTTGTTTTAGTGAACCATGGTAGAGCGACGCCAAA

GGCGTCTGAGGGCAGCAGTTGAGCATAACGATATAACAGTGATAAGGCACGCGATGGAAA

TAAAATGGCACCGCTCGGCAATGTAAAAGGTTTAATTAAATCCACAAATTGTTCGGCGAT

TTTTTCCTCTTTTGGATCATCTCGATCCAAAATGCGTCCAATCAAATAATTCTGTATGAT

TTGATGTGCCTCTTGATATTTTTTAATTTGTGATGCCACTTGTAATTTGTCCACTTCCGG

CGTGAATATGGCATAAATAGATTCATTACTACGTGCTCGCCCCTTTGATTGTACATACGA

ATTAAACGTCTTTAAAGGGTCGTAAGCGAAGACGTAATTGCAGGCCTGTACATCAATGCC

CTCTTCTAGTACATTCGAACAGACAATCACATTACACTCACCGGAGCGGAAGCTTTCGAT

GGCCGATTTGTTCCATTTCTGATCAAGAACGCTTTCTATACTTGGCATTATCGAATTCCG

CCCCACCATGAACTGCGGCCGTAGCACTTTCGTTAGAGCCGGTTCCAGTTCAGCATACTT

TTTCAGTACGTAGAATACACATTTAGCTGTGTAGCGGCGTTCCACAAATATCAAACAACG

AATATCGGCCGGATCTTTATTAGAAAATAGTTTTTTCGCTAAAAGAAGAAGCGTTCGCAT

TTTGGGCGTAGAAAAATTTAGTATTATATCCAGCGTGTGACAAACAGTATCTGGAAGTGA

GTCCTCTTCTTTTAGCTCCATTACAAGCATATGACGTATTTTTTCACATACGCTGATTGT

ATACCGATAGAGATTGCGTAAAGCCAATGTTTCTGCCTGTCGCTTCTTTATTTCAAATTC

CATTATCGGTGACATAATTGCTATAGCTGATGCATATAAACCGAACTCATTTGTTTGATA

TAAGAAATCTTTTAGTAGATTTGTTATGAATGTCTTTTTATTTTGACGATATCCTGCCAA

ATTTTTTGTCTGCTTTACTGAAATAGTCCCTAAATCCCATTGTTTTATCACATCAAAACT

TTCACTTACGATCCGTTCGATATGCTTAAGCAATTGAAAATCGTGGGTTTGTCTGGTGAA

AGTTATTAGTTGTTCTTTTGGTTTTGTCGAATATAGCATAACATTTTGGTATTTTTCCAT

ACTACTAACTGTGACAATATTACCACGGAAAGTAGACTCTAAACATTCAAGCTCCTCGCG

GATTCTAGTAAGTTTGTTACCCTTCAACAAGACACCAGTTAGGCCAATCACACGCGGTAA

TTTAGTTTTGTCTGCAACTTCTTGGAAGTGACGCATGAACTCATGCATCGGATGGTTTCT

TGTTGCATGGTGGCATTCGTCGATTACAACCACACTTAAATCAGTGATTTTAATGTAATT

TTGTGAAACTATATCCACAAAAATTTGTGCAGTCCCTACTAGAACTTGATTTACACTGAT

TTCTTCATCCCATTTTTTTCTCGACCAACTGTCAACATCGCGCTCGCCTACATAGAAACC

AATTTGCAAATTAGTGCATCGTTTAAGTTCTATAGCCTGTTGGCGTGCTAGCTCGACAGT

ATTACACATGAATATAGCACGTTTTCCACCATTTTCAATTGTTTCTTGCATTTGATGCGA

AAAACGCTTCAATGCCATTATCGCCACGTAAGTTTTGCCGGCGCCGGTAGGAAGATATAT

TATCGCGTTCCGATCCATGACATAGTTTAGGAGGTCCAACTGATAGTGGCGCGGCTCCAT

TGCCTTCTTCGGTATAATATTTGTAACAAGATTCGTTGTCATTGTTTTTTTTTTTTCGTT

TTTGATTTTCTTTCCAGGAAATTTATTTGCTGTATACTCTTTTCCTTACTGCTGATTAAA

ACTCATCCAATTGCTTCAGCAACTGTTGCGGTTGTGAAATTGAACTGATCAATATTTCTT

ACGTGGCACTAGCACACTGGGGGATATGTTATTTGTTTTGCTTTGGGAGTGAGCAAGTAC

TTAATTTATATCAACTACGGTGATTTCCCGACTTAAAGAAAAAAAACAACACCAAACAAA

GCACATTAAGTACGCCTGGATCGTTGTACAGAATCATAAATAAAAACACTCTCAGCTGGT

TGTCAAAAAATGCACAGCTGAGATCACCTGATTCTTTCATAGAAAATCCCATCAAAAAGT

CTATAAAAT

**tBLASTn(First hit)**

Score = 1582 bits (4095), Expect = 0.0, Method: Compositional matrix adjust.

Identities = 828/1719 (48%), Positives = 1150/1719 (67%), Gaps = 70/1719 (4%)

Frame = -1

Query 6 IKPRGYQLRLVDHLTKSNGIVYLPTGSGKTFVAILVLKRFSQDFDKPIESGGKRALFMCN 65

++PR YQL L++++ N I+YLPTG+GKT+VAI+ LKRFS + IE+GGKRA+FMCN

Sbjct 5280 MEPRHYQLDLLNYVMDRNAIIYLPTGAGKTYVAIMALKRFSHQMQETIENGGKRAIFMCN 5101

Query 66 TVELARQQAMAVRRCTNFKVGFYVGEQGVDDWTRGMWSDEIKKNQVLVGTAQVFLDMVTQ 125

TVELARQQA+ ++RCTN ++GFYVGE+ VD W+R W +EI NQVLVGTAQ+F+D+V+Q

Sbjct 5100 TVELARQQAIELKRCTNLQIGFYVGERDVDSWSRKKWDEEISVNQVLVGTAQIFVDIVSQ 4921

Query 126 TYVALSSLSVVIIDECHHGTGHHPFREFMRLFT-IANQTKLPRVVGLTGVLIKGNEITNV 184

Y+ ++ LSVV+IDECHH T +HP EFMR F +A++TKLPRV+GLTGVL+KGN++T +

Sbjct 4920 NYIKITDLSVVVIDECHHATRNHPMHEFMRHFQEVADKTKLPRVIGLTGVLLKGNKLTRI 4741

Query 185 ATKLKELEITYRGNIITVSDTKEMENVMLYATKPTEVMVSFPHQEQVLTVTRLISAEIEK 244

+L+ LE T+RGNI+TVS ++ +NVMLY+TKP E +++F Q + + I + +

Sbjct 4740 REELECLESTFRGNIVTVSSMEKYQNVMLYSTKPKEQLITFTRQTHDFQLLKHIERIVSE 4561

Query 245 FYVSLDLMNIGVQPIRRSKSLQCLRDPSKKSFVKQLFNDFLYQMKEYGIYaasiaiisLI 304

+ + ++G ++++K+L R +KK+F+ L DFLYQ E+G+YA++IAI+S I

Sbjct 4560 SFDVIKQWDLGTISVKQTKNLAGYRQ-NKKTFITNLLKDFLYQTNEFGLYASAIAIMSPI 4384

Query 305 VEFDIKRRQAETLSVKLMHRTALTLCEKIRHLLVQKLQDMTYdddddNVNTEEVIMNFST 364

+EF+IK+RQAETL+++ ++R +++CEKIRH+LV +L++ D +T ++I+NFST

Sbjct 4383 MEFEIKKRQAETLALRNLYRYTISVCEKIRHMLVMELKEEDSLPDTV-CHTLDIILNFST 4207

Query 365 PKVQRFLMSLKVSFADKDPKDICCLVFVERRYTCKCIYGLLLNYIQSTPELRNVLTPQFM 424

PK++ L+ K F++KDP DI CL+FVERRYT KC++ +L Y + P L VL PQFM

Sbjct 4206 PKMRTLLLLAKKLFSNKDPADIRCLIFVERRYTAKCVFYVLKKYAELEPALTKVLRPQFM 4027

Query 425 VGRNNISPDFESVLERKWQKSAIQQFRDGNANLMICSSVLEEGIDVQACNHVFILDPVKT 484

VGRN+I P ESVL++KW KSAI+ FR G N+++CS+VLEEGIDVQACN+VF DP+KT

Sbjct 4026 VGRNSIMPSIESVLDQKWNKSAIESFRSGECNVIVCSNVLEEGIDVQACNYVFAYDPLKT 3847

Query 485 FNMYVQSKGRARTTEAKFVLFTADKEREKTIQQIYQYRKAHNDIAEYLKDRVLEKTEPEL 544

FN YVQSKGRAR+ E+ + +FT + ++ + QI +Y++AH I YL R+L++ +P+

Sbjct 3846 FNSYVQSKGRARSNESIYAIFTPEVDKLQVASQIKKYQEAHQIIQNYLIGRILDRDDPKE 3667

Query 545 YEIKGHFQDDIDPFTNENGAVLLPNNALAILHRYCQTIPTDAFGFVIPWFHVLQEDERDR 604

+I F D I PFT +GA+L P+ AL++L+RY Q +P+DAFG +PWF

Sbjct 3666 EKIAEQFVDLIKPFTLPSGAILFPSRALSLLYRYAQLLPSDAFGVALPWFT--------- 3514

Query 605 IFGVSAKGKHVISINMPVNCMLRDTIYSDPMDNVKTAKISAAFKACKVLYSLGELNERFV 664

S + + + MP+ +++TI SD K AKISAAFKAC LY GELN+R +

Sbjct 3513 --KTSTERGIAVELKMPLQSSVKETIISDTFPTTKEAKISAAFKACIKLYEHGELNDRLL 3340

Query 665 PKTLKERVASIADVHFEHWNKYGDSVTATVNKADKSKDRTYKTECPLEFYDALPRVGEIC 724

P T +E V +A+ F+HW K+ D VT+ A K + R Y + P E +A+P + E+C

Sbjct 3339 PVTKRECVQKVAEELFDHWKKFNDDVTSKT--AGKQQRRLYNRKYPDELQNAIPHLNEVC 3166

Query 725 YAYEIFLEPQFESCEYTEHMYLNLQTPRNYAILLRNKLPRLAEMPLFSNQGKLHVRVANA 784

YAYEI P FE +Y+ H+ LQ+ RNYAIL R ++P LAEMPLF NQGK+ V++A

Sbjct 3165 YAYEIHAYPHFEINDYSLHISTLLQSNRNYAILSRKRIPPLAEMPLFMNQGKISVKIAPQ 2986

Query 785 PLEVIIQNSEQLELLHQFHGMVFRDILKIWHPFFVLDRRSKENSYLVVPLILGAGEQKCF 844

PL + I N +QL+ L +FH M+FRD+L+ W FFVLD+R++ENSYL+VP+ G

Sbjct 2985 PLTLTITNEQQLQKLCKFHLMLFRDLLECWKTFFVLDQRNQENSYLIVPINAGK-----I 2821

Query 845 DWELMTNFRR-LPQSHGSNVqqreqqpaprpeDFEGKIVTQWYANYD-KPMLVTKVHREL 902

DWEL+ NF+R LPQ S +Q +PED+ GK+V +WY+ + + +VTKV +

Sbjct 2820 DWELVENFQRLLPQRKYS--VSERKQKIYKPEDYIGKVVNKWYSGRENQRFVVTKVLTDR 2647

Query 903 TPLSYMEKNQQDKTYYEFTMSKYGNRIGDVVHKDKFMIEVRDLTEQLTFYVHNRGKFNAK 962

TPLS + N +Y +F KY N + VV ++FM+EVR LT + F+++ GK +

Sbjct 2646 TPLSPFDNNDY-SSYVDFIDCKYKNEVDCVVQPNQFMLEVRALTSRRNFFINAVGKSSNV 2470

Query 963 SKAKMKVILIPELCFNFNFPGDLWLKLIFLPSILNRMYFLLHAEALRKRFNTYLNLHLLP 1022

K + LIPELC NF +PGD+WLK +FLPSIL+R++FLLHAE LR R NT+L +

Sbjct 2469 HKNNCAIRLIPELCHNFMYPGDMWLKALFLPSILHRVHFLLHAENLRCRVNTFLGI---- 2302

Query 1023 FNGTDYMPRPLEIDYSLKRNVDPLGNVIPTEDIEEPKSLLEPMPTKSIEASVANLEITEF 1082

+ +Y P+ L +D SLKR VD GN + E+ +EPK LL MPT SIEA L I +

Sbjct 2301 VDSVEYQPKQLAVDGSLKRAVDFDGNAM--EEPDEPKKLLPAMPTSSIEAEFEMLHIDDV 2128

Query 1083 ENPWQKYMEPVDLSRNLLSTYPVELDYYYHFSVGNVCEMNEMDFEDKEY-WAKNQFHMPT 1141

W++Y+EP D SR S +PVELDYYY F G E++ + + E+ +++Q +MP

Sbjct 2127 --AWKEYLEPEDFSRKPESLFPVELDYYYKFISGLAIELDALKLTEGEWSLSQSQLNMPN 1954

Query 1142 GNIYGNRTPAKTNANVPAL-MPSKPTVRGKVKPLLILQKTVSKEHITPAEQGEFLAAITA 1200

N G+ K ++ +P R L IL+ T+S++ + AEQ +FLAAIT

Sbjct 1953 QNAAGSPVRGKDLCSISLCDVPLADKRR-----LDILELTLSEQTLKTAEQADFLAAITT 1789

Query 1201 SSAADVFDMERLEILGDSFLKLSATLYLASKYSDWNEGTLTEVKSKLVSNRNLLFCLIDA 1260

+ A DVFDMER E LGDSFLK S +LYLA KY W+EG LTE+K KLVSNRNL++C++

Sbjct 1788 AGANDVFDMERFEFLGDSFLKFSISLYLAHKYPKWHEGFLTEIKGKLVSNRNLIYCMLQT 1609

Query 1261 DIPKTLNTIQFTPRYTWLPPGISLPHNVLALWRENPEFAKIIGPHNLRDLALGDEESLVK 1320

DIP+ + F P + WLPP ISLP N+L L + N + + + P +L + L D+E ++

Sbjct 1608 DIPECICGYLFKPPHEWLPPLISLPRNLLELLKNNEQVLENLTPSDLYGIELDDDE-ILS 1432

Query 1321 GNCSDINYNRFVEGCRANGQSFYAGADFSSEVNFCVGLVTIPNKVIADTLEALLGVIVKN 1380

G CS ++ + R S +N + +KV+ADTLEA+LGV VKN

Sbjct 1431 GCCSAVHLTKLNASSR-QAHKLAEEERLDSGLNLFAYKEVLRDKVVADTLEAILGVCVKN 1255

Query 1381 YGLQHAFKMLEYFKICRADIDKPLTQLLNLELGGKKMRANVNTTEIDGFLINHYYLEKNL 1440

YG+ + F+MLE+F IC+ D D+ T+L++L+LGG + N+++ ++D ++IN+ LE+NL

Sbjct 1254 YGIINTFRMLEFFGICKPDADQSFTRLMDLKLGGPLLHTNISSRQVDSYIINYPKLEENL 1075

Query 1441 GYTFKDRRYLLQALTHPSYPTNRITGSYQELEFIGDAILDFLISAYIFENNTKMNPGALT 1500

GY F+DR YLLQALTHPSYPTNRITG YQELEFIGDAILDF++S YIFE + M+PG LT

Sbjct 1074 GYKFRDRAYLLQALTHPSYPTNRITGCYQELEFIGDAILDFMVSCYIFERYSHMDPGMLT 895

Query 1501 DLRSALVNNTTLACICVRHRLHFFILAENAKLSEIISKFVNFQESQGHRVTNYVRILLEE 1560

DLRSALVNN TL C+CVRHR H F+LAEN+ L+E I F +QE + VT+ V IL EE

Sbjct 894 DLRSALVNNITLGCVCVRHRFHLFLLAENSSLAESIKNFDKYQEKHNYYVTDQVHILSEE 715

Query 1561 ADVQptpldlddeldmtelpHANKCISQEAEKGVPPKGEFNMSTNVDVPKALGDVLEALI 1620

N E E +N+S NVDVPKALGD+LEALI

Sbjct 714 ----------------------NITTENEVEGAT-----YNISDNVDVPKALGDILEALI 616

Query 1621 AAVYLDCRDLQRTWEVIFNLFEPELQEFTRKVPINHIRQLVEHKHAKPVFSSPIVEGETV 1680

AAVYLD RDL+ TW VI+ L E E +F ++VPI+ +RQL +HKHA P +S PI + ET

Sbjct 615 AAVYLDSRDLRTTWHVIYGLLEQEFNQFAQQVPIDAVRQLNDHKHANPKYSKPISDNETY 436

Query 1681 MVSCQFTCMEKTIKVYGFGSNKDQAKLSAAKHALQQLSK 1719

MV CQFTC++K+++V GFG N QAK +AAKHALQ L+K

Sbjct 435 MVKCQFTCLDKSVEVNGFGLNGKQAKKAAAKHALQILAK 319

**Conserved domains**

**protein containing domains DEXDc, Dicer_dimer, PAZ, and RIBOc**

**
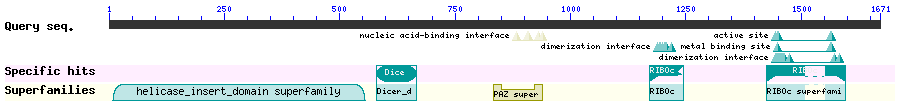
**

**Argonaute-2**

**>TRINITY_DN30039_c4_g1_i5 len=3965** GTTTATTTACAGTATGGGTACTCGTAAATCTATCCATCTATCCATATAAATATATTATAA

TTTTGCTAGCGAGAGACAGAACTTCGCAAAATAAAGTAGAAATTTCAGTGAGCAATTTGT

GTTTTAACAGTTAAAAGCTTAAATATTAATAAAGTATCAATACCAATATCGACAAACAAT

AAAATAAAGTGAGAAATATGGGTAAAAAGAATAAAAATAAAACAGGTGGAGGACAAAGCA

GCGGTGAGCCTGCACCAACTGGACCTACGCCACCACAGGGACAGCAGCGGCAACCACCGC

AGCATGAAATGCCAACTAGTAGTGGATCCGCTTCAGCACAACAATCACAGCAATCGCAAA

GACAACAAGAGCAACGACCAAAGCAGCAGGAGCAACTAAGTGGTCAACAACACCCACAGC

AGCGGAAGCAAGGACAGGGTCAACAACAACCTCAGGGACAAAAACAACAACAGAAGCAAG

CACGGGGTCAACAACAACAGTACGAGCAGCAGGAGCAAACATGGCGTAAACAACAACCAC

AGGGACAGCAACAACAGCAACAACAACAACAACCACAGGGACAGCAACAACAACAACAAC

GGAAGCAAGCACAGGGACAACAACAGCCGCACCAGCAACAACAACCACAGCTGCAGGAGC

AAACATGGCGTCAACAACAACCGCAGCAGGAGAAGCAAGCATGGGGTCAACAGCAACCAC

AACAGCAGGAGCAACCACAGCAACGGAAGCAAGCACGGGGTCAACAACAACAACAACAGA

AGCAACCACAGGAACAACAACAACCACAGCAACGGAAGCAAGCACGGGGACAGCAACAAC

AACAACAGCAGAAGCAACCACAGGAACAACAACAACCACAGCAACGGAAGCAAGCATGGG

GTCAGCAACAACCGCACGAGCAGCAACAACCACAGCTGCAGGAGCAAACATGGCGTCAAC

AACAACCACAGCAGCAGAAGCAAGCATGGGGTCAACAACAACCACAACATCAGAAGCAAG

CATGGGGTCAACAACAACCACAACAACAAGAATTTAAACAACAGCCCAGTAGCTCATCCT

ATACACAATCTTCTGGAGCTGTCCCGAAACAGGCAGGAGAACAACCTTCACCGCAGAGAC

AGACTCCTATTCAAGCAGCATCGCAAACTGCATCATCACGATCATCGCGTACAAGCATTT

CCCAGTCTTCTTCATCAACTTCTATTACATCATCTACGTCCCAAAGAAGCATACAACCCG

GCACTTTGGGCATAAAAGGTGAAGTTGAAGCTAATTATTTGGTTTTGAATTTAGGCAAAA

TGCCTGATATAGCATACCATTATGACGTTACTATTACACCGGATCGCCCTAAGAAATTTT

TCCGAAGTGCTTTCAAACAGTTCATAAACACTCATCTGCCTGGTCAAACCGTCGCTTTTG

ATGGCGTTAAGAGTTGTTACATGGTAGAAAGGTTACCAAATCCAGTTTATGAAGGCGATG

TTAAGATAGCAGATTCCGGTTCGAGGCAAATACAATTTAGAGTTTCTATTAAACTCACAG

ACAATCCCGAAGTTGAGCTGCGATCGTTAAAAACTTACCATAATGAGCGTGTGTTTGACA

AACCAATGCGTGCCCTACAATGTATTGAGGTTGTTTTAGCAAATGATTGCCACAACAAAG

GTATACGTGCGGGTCGCTCCTTCTTTACACAACCTGATAAAACAATGGACCTCGATGAGG

GCTACGAGTTATACACAGGACTTTACCAAGCTGCTATATTGGGTGAACAGCCTTATTTAA

ATGTAGATATTTCACATAAGTCATTTCCTATGCCGTATGATTTGATTACTTATTTGGAGA

GTGTATTGAATTGTAATAGACAATCCAATTTAGACCCAAGGAACTTGCAAAGGCTATCTA

AGCACTTGAAAAATTTGAAAGTGGTTTATAACCCACCTCCTAGTTTTGGTGCTGGTCCAC

GCTCTTATAAAGTAAACGATATCAGTAGAGAACCCGCTGCCACTTTGTCATTTACCACTG

ACAATGGAGAAAAGTTTACCGTTCAAAAATACTTCCAAAGCCGTGGTTATAATTTGAGAT

ACCCTAATTTAAACTGCGTAGTGGCGGGATCAACAATAAGGCCGAATTATTTTCCGATGG

AATTATGTAGCATTGAAGCAGGACAAGCCATCAGGAGAAAAGATGGAAGCAGACAAGTTC

AAAAGATGATTCGATTCGCAGCAACATCGACAGATGAACGTAAACGAAAAATTATGCAAA

AGCTCGCGTATTTTAATATTAATGCGGATCGCCTCGTACAAGCCTTCGGTATTAGCGTCG

GCGAACAATTTATAAAGGTACCAATGAGATTGCTGAAAGCACCAGCAATTGAATATCACT

CGAGCAAATACGTAGAGCCTCGTAATGGCTCGTGGCGCAATTTACCGTTTTTAGAAACCG

GAGCAGCTTTCAAAAAAAGTGGTCATAAATGGGCTATCATATATACTCCATCCAGATTTT

TGAAATATCCAACTTTAATGGATTTGGCAAACATGTTATATAATGGAGCCAAACGAATGG

GAATAAACTTGGATGCCCAAAAGGACATAAAAGAAACAGGAAATATTGTTGCAACTTTAG

AAGAATATAAAAGAAATGATTACGATTTGGTTATAGTTGTAATTCCCGGCTTTGGCACCT

CGTATGCTGATATTAAACAGAAAGCCGAACTAGTTTGTGGTCTTCTAACTCAATGTATCA

AAGAGCAGACTTTAAATCGTGGTGTGAACGATATGTTGATCTCGAATTTGCTGCTAAAAG

TAAATTCAAAACTAAATGGATCCAATCACAAAATATCGGCTAATCATCACGTTGTACTTG

ACCACGTAATGTTTATGGGCGCTGATGTCACTCATCCATCACCCGACCAAAATAACATAC

CAAGTGTCGTCGGTGTGGCAGCCTCGCACGATCTGAATGGCTCTGTTTACAACATGCAAT

ACCGTTTACAGGAGCCAGCAAAAGAAGAGATCGTTGACATGCGAACCATTGCTTCTCACC

ATCTAAGAGTATATTTTCAAAAACAGAAATGTTATCCTAACAATATTATATATTATCGCG

ACGGTGTATCAGACGGACAGTTCCAAAAGGTGGAGATGCTAGAACTTGGTGCTATACGGG

CCGTATGCAAAGAGTTGCGTATTACACCTAAAATAACTTGCATAATTGTGGTGAAGCGCC

ATCATACTCGCTTTTTCCCCACAAAGCCGACTGGGGATAAATGGAATAATGTTCTGCCTG

GGACGGTAGTTGATCAGAAAATCGTACACCCAAACGAGACGCAGTTCTTTATGGTAAGCC

ACCAATCCATTCAAGGTACAGCAAAACCTACACGTTACAATGTGATTGTGGATGATGCCA

AAATGTCTATGGACGATTTGCAAAAGATGACCAACAACTTGTGTTACATGTTTCCTCGTT

GTAATCGCGCAGTTTCTTATCCGGCACCAGCATACCTAGCCCATTTAGTTGCAGCACGTG

GTCGTGTTTATATTGACGGACCACCACTACGGCGAGCACTACCTGAGGAATACAAAAAGC

GTTTGATCAATGAGAGGTTCATGAATACCACGCCGATGTTCTTTGTTTAAATGAGAAATG

AAATTACGGATTCTCATTCAGATACATATATTCATCCACATAAAATAATCCGATATACAT

AGCTTATGATTATTGTTTATATATATATAATGGAAATTCTACAAAAGAGTGTTTGAGCAC

CAAATTTTCATTTAGTCCAATAGTTTTCCCCAAATAACCGATGCTTTTCTGAAAAAAAAA

ATTTATTTTATAGTCCAACAAACTTGGTTTCAGACGGGCTTATTTTTTCGATAGAAATAA

TATTCAACTCAAAATAGCGCTTGCTACCAATTTGGCCTTTTTACCTTCCATCTAATTGGG

CAGTA

**tBLASTn(First hit)**

Score = 834 bits (2155), Expect = 0.0, Method: Compositional matrix adjust.

Identities = 431/819 (53%), Positives = 554/819 (68%), Gaps = 29/819 (4%)

Frame = +3

Query 405 IKRGTIGKPGQVGINYLDLDLSKMPSVAYHYDVKIMPERPKKFYRQAFEQFRVDQLGGAV 464

I+ GT+G G+V NYL L+L KMP +AYHYDV I P+RPKKF+R AF+QF L G

Sbjct 1251 IQPGTLGIKGEVEANYLVLNLGKMPDIAYHYDVTITPDRPKKFFRSAFKQFINTHLPGQT 1430

Query 465 LAYDGKASCYSVDKLPLNSQNPEVTVTDRNGRTLRYTIEIKETGDSTIDLKSLTTYMNDR 524

+A+DG SCY V++LP +V + D R +++ + IK T + ++L+SL TY N+R

Sbjct 1431 VAFDGVKSCYMVERLPNPVYEGDVKIADSGSRQIQFRVSIKLTDNPEVELRSLKTYHNER 1610

Query 525 IFDKPMRAMQCVEVVLASPCHNKAIRVGRSFFKMSDPNNRHELDDGYEALVGLYQAFMLG 584

+FDKPMRA+QC+EVVLA+ CHNK IR GRSFF + P+ +LD+GYE GLYQA +LG

Sbjct 1611 VFDKPMRALQCIEVVLANDCHNKGIRAGRSFF--TQPDKTMDLDEGYELYTGLYQAAILG 1784

Query 585 DRPFLNVDISHKSFPISMPMIEYLERFSLKAKINNTTNLD-YSRRFLEPFLRGINVVYTP 643

++P+LNVDISHKSFP+ +I YLE N +NLD + + L L+ + VVY P

Sbjct 1785 EQPYLNVDISHKSFPMPYDLITYLESV---LNCNRQSNLDPRNLQRLSKHLKNLKVVYNP 1955

Query 644 PQSFQSAPRVYRVNGLSRAPASSETFEHD-GKKVTIASYFHSRNYPLKFPQLHCLNVGSS 702

P SF + PR Y+VN +SR PA++ +F D G+K T+ YF SR Y L++P L+C+ GS+

Sbjct 1956 PPSFGAGPRSYKVNDISREPAATLSFTTDNGEKFTVQKYFQSRGYNLRYPNLNCVVAGST 2135

Query 703 IKSILLPIELCSIEEGQALNRKDGATQVANMIKYAATSTNVRKRKIMNLLQYFQHNLDPT 762

I+ P+ELCSIE GQA+ RKDG+ QV MI++AATST+ RKRKIM L YF N D

Sbjct 2136 IRPNYFPMELCSIEAGQAIRRKDGSRQVQKMIRFAATSTDERKRKIMQKLAYFNINADRL 2315

Query 763 ISRFGIRIANDFIVVSTRVLSPPQVEYHSKRFTMVKNGSWRMDGMKFLEP----KPKAHK 818

+ FGI + FI V R+L P +EYHS ++ +NGSWR + FLE K HK

Sbjct 2316 VQAFGISVGEQFIKVPMRLLKAPAIEYHSSKYVEPRNGSWR--NLPFLETGAAFKKSGHK 2489

Query 819 CAVLYCDPRSGRKMNYTQLNDFGNLIISQGKAVNISLDSDVTYRPFTDDERSLDTIFADL 878

A++Y R + Y L D N++ + K + I+LD+ D + I A L

Sbjct 2490 WAIIYT---PSRFLKYPTLMDLANMLYNGAKRMGINLDAQ-------KDIKETGNIVATL 2639

Query 879 ---KRSQHDLAIVIIPQFRISYDTIKQKAELQHGILTQCIKQFTVERKCNNQTIGNILLK 935

KR+ +DL IV+IP F SY IKQKAEL G+LTQCIK+ T+ R N+ I N+LLK

Sbjct 2640 EEYKRNDYDLVIVVIPGFGTSYADIKQKAELVCGLLTQCIKEQTLNRGVNDMLISNLLLK 2819

Query 936 INSKLNGINHKIKDDPRLPMMKNTMYIGADVTHPSPDQREIPSVVGVAASHDPYGASYNM 995

+NSKLNG NHKI + + ++ + M++GADVTHPSPDQ IPSVVGVAASHD G+ YNM

Sbjct 2820 VNSKLNGSNHKISANHHV-VLDHVMFMGADVTHPSPDQNNIPSVVGVAASHDLNGSVYNM 2996

Query 996 QYRLQRGALEEIEDMFSITLEHLRVYKEYRNAYPDHIIYYRDGVSDGQFPKIKNEELRCI 1055

QYRLQ A EEI DM +I HLRVY + + YP++IIYYRDGVSDGQF K++ EL I

Sbjct 2997 QYRLQEPAKEEIVDMRTIASHHLRVYFQKQKCYPNNIIYYRDGVSDGQFQKVEMLELGAI 3176

Query 1056 KQACDKVGCKPKICCVIVVKRHHTRFFPSGDVTTSNKFNNVDPGTVVDRTIVHPNEMQFF 1115

+ C ++ PKI C+IVVKRHHTRFFP+ T +K+NNV PGTVVD+ IVHPNE QFF

Sbjct 3177 RAVCKELRITPKITCIIVVKRHHTRFFPTK--PTGDKWNNVLPGTVVDQKIVHPNETQFF 3350

Query 1116 MVSHQAIQGTAKPTRYNVIENTGNLDIDLLQQLTYNLCHMFPRCNRSVSYPAPAYLAHLV 1175

MVSHQ+IQGTAKPTRYNVI + + +D LQ++T NLC+MFPRCNR+VSYPAPAYLAHLV

Sbjct 3351 MVSHQSIQGTAKPTRYNVIVDDAKMSMDDLQKMTNNLCYMFPRCNRAVSYPAPAYLAHLV 3530

Query 1176 AARGRVYLTGTNRFLDLKKEYAKRTIVPEFMKKNPMYFV 1214

AARGRVY+ G L +EY KR I FM PM+FV

Sbjct 3531 AARGRVYIDGPPLRRALPEEYKKRLINERFMNTTPMFFV 3647

**Conserved domains**

**protein containing domains ArgoN, ArgoL1, PAZ_argonaute_like, and Piwi_ago-like**


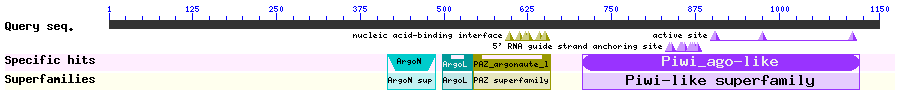


**R2D2**

**>TRINITY_DN28410_c0_g2_i4 len=2773**

CGGGTAGATTAATTTTGTGGATTTAAGGGTAATTCATGCATATGTGAACGCACTTTTACC

GTCAGACATTAGAAATATAATTGTGAAGTGAATTAGGAATGGGAGGGCAAATTATGTGCT

ATGGAAACAGAGCATTATTTTGATCGAGTTTCTAGTATGGCATCGCTGACCACACGTGTT

GGTTAACACCGACAAGTTGTTTTGTTTACCAAATAGAAAAACGTATTTCCACAAAATCTT

GTTGAGATAACCGGATTTTATTTACAAAATGTTTAAATTTGCTTTTAATGTTGATATTGA

TAATGACAATCCTTTCAGTAGAAAAAGTAATGAAAAGGAATTGAACTTAATACCTACTAC

GAGTCAAAATGAGTTGAACAATGAACCTGAAGAATGGTACGAAGCAGAAAATATACCTAT

AAGCATTGAAACCCTGGAAAACTTAGATGTATATAAATTAAATGCGAGAATATTAAATAT

TGGCGATATTTCCATAAAACACATTATGACAAAATTTCTATTAGATCACATAACTGGCAA

TAGTGAAGACAATAAAGGTATTAGCAAAGCCGAGCAAAAGCACTCCGACCTCATTCCAGG

CATTTATGAAGGTGGTGCGAAAATCTGGGAATGCACAGAAGACCTTCTGCAATACTTAGT

AAAAACTTTTAAGCCACAAGAGTGGCGCGGAAAACGGGTGTTGGATCTAGGCTGTGGCGC

TGGTTTGCTTGGTATTTATGCGTACAAGTGTGGTTCAACAGTGCACTTTCAAGATTATAA

TAAAGATGTTTTGACACAAATTACGATACCAAATGTTCTGTTAAATGTAGCCAGGGTAAA

ATCTGATGTTGACAAATCAGGCATATCGAACGCAGAACAAATTCAACGGAATGAAATTGA

CGCATTGAGTAAAAGTTTGCAATTTTACTCTGGTGATTGGCGAAAATACTCGGACCTAAC

TTCGCAAGGCACAACGGATAAGTTCGATTTTATACTAACATCAGAGACAATTTATAATCC

TAAAAATCAGCAAAAGCTGTTGGAGACATTATACAGGAAACTAAGTCCAGGCGGTGTAGT

GCTAGTTGCCGCAAAAACATACTATTTTGGTGTTGGTGGAGGACTAAGGCAATTTGAGGA

CTTGATTACAGCAGATAAACGATTCCAATGCAAAATAGTCTGGACTAGTACGGATGGCGT

AGGTCGTGAGATATTAGAATTAAGTTTAAACAAACAATAACTTATTTAATTTTTTATGGT

CTACGAAGTACACGAGCATGTAATTAAAGAAAAAAATTTTAAATTATAATCGTTTGTTGG

TACATTTTAATAAGCACATGAAACAATTCCCTTTAATTAATTCATTCACTAAGCATCCGT

TCAGCTTTTTTTTTTTTTAATTCATATATTGTGATAATAGTTACTCCTCTCATTCTCGTA

AAATATTTTTCATAGCATCACTTTGAAATAATCAATAATATCTCTGTACACTTTGCTCGG

CGATCCAGCGAATACAACATCAAAGTCACAGTTCATCTCGACAAACAACATGGCTTTTCC

TTCCGATGAGTGCATTTCCGAAATTTTTGGTTTGATTTTTAGCGTATCCAGTAAATCCAT

TACCTGGTCCTCCTCATTCTCGTATGTAGTACTAGAAAGTATTTTATACGCTGCGTTTTT

TAATTCGGTATAAAAATTCTTGAAATAATTGTGTCGATCACAGAGTAAGACCCCTGGTAT

TTCGCCGGTAGTGGATTCAGTCATTTCACGATAACTTTTAAATTTTTTATAACGTTCTGC

TTCAATATCTTCTTGTGCATCTTGTAACGTAGCTACTTGCATTTGATGCTCCAATCTAGA

CACATCATCAACAATTATACGTAGCATTTCATAAGCGGCTTTCTGTCGTGCATCCTTTTT

CTTTTCTGAAACTCCATAACGACGTATTGAAGCAACTGTGCATAACGCTGTGAATTCGGG

CGCATCTGGTGTGCCGGCCTGTTGTACAATTTCGATTGTGGGTAGCGGCATTTGGTGTTG

TACGCAATAATCCCGCAACTGGACTATGACGTCAGTAGTTGGTATGTCAGCATCGAAATC

CTCGACGAGTTGTGGCATGTTGCTGAGCCGCAAACGTTTTATGACTGAAGCGGCGGCCTG

GTGTTTTGCATCTCGTTTAGACCGACCTCTACCCTCCGCGACGGTGTCCATAAAGGAAAT

CTGGCAGGTAAAACTACCGTCTTCCTCATCAACGTACTCGTAGGTTGGTGGCTGTACCTT

GTTTTTAGCACAATATTCCTGTAGTGCCGAAACGGAGGACTTACCAACCATTTTGGTATA

AATTGTACACTTTCGAAATTGCGGTGATTCCCCTAGTAAAGATGCAGGGATAAGAAATAT

GTGCCCTTCCGTGTTAACTTAACGTTTCGACATTAAATTTTTGATTATTTTCAATGGTCA

ATTTCAATGAAATTGCCAATCGCGACAGCAAATTGAATGAAGTTGGCATTCATGTTGTGA

AATATTTTAAGTCATATATATGGCCAACATCTGTGAAACAATTAGCTGCCATTTTGCATA

TTTGATGGCCATTTGATATTTTTTTGTGGCTCTTCCATTGCCAACATCTTTGATCATTTT

AGTTTTTCAGACTGTGCATTTCCCTTCTTTGCAAAAAAGGATTCATCCAAAATCTCGGCG

TCTTTCTTTCCTCTTCATTAGGGCAGGTCGATTTAAAAATCGCTCATTGCTCTGTGAAAA

TCGTATTCTAGGG

**tBLASTn(First hit)**

Score = 277 bits (709), Expect = 9e-085, Method: Compositional matrix adjust.

Identities = 147/312 (47%), Positives = 211/312 (68%), Gaps = 21/312 (7%)

Frame = -2

Query 1 MDNKSAVSALQEFCARTQINLPTYSFIPGEDGGYVCKVELLEIEALGNGRSKRDAKHLAA 60

M KS+VSALQE+CA+ ++ PTY ++ EDG + C++ ++ A G GRSKRDAKH AA

Sbjct 2331 MVGKSSVSALQEYCAKNKVQPPTYEYVDEEDGSFTCQISFMDTVAEGRGRSKRDAKHQAA 2152

Query 61 SNILRKIQL--LPGIHGLMKDSTVGDLDEELTNLNRDMVKELRDYCVRREMPLPCIEVVQ 118

++++++++L +P + V D D ++ D++ +LRDYCV+ +MPLP IE+VQ

Sbjct 2151 ASVIKRLRLSNMPQL--------VEDFDADIPTT--DVIVQLRDYCVQHQMPLPTIEIVQ 2002

Query 119 QSGTPSAPEFVACCSVASIVRYGKSDKKKDARQRAAIEMLALISSNSDNLRPDQMQVAST 178

Q+GTP APEF A C+VASI RYG S+KKKDARQ+AA EML +I + L QMQVA+

Sbjct 2001 QAGTPDAPEFTALCTVASIRRYGVSEKKKDARQKAAYEMLRIIVDDVSRLE-HQMQVAT- 1828

Query 179 SKLKVVDMEESMEELEASRRKKFTTYWELKEAGSVDHTGMRLCDRHNYFKNFYPTLKKEA 238

++++ E++EA R KKF +Y E+ E+ + + G+ LCDRHNYFKNFY LK A

Sbjct 1827 -------LQDAQEDIEAERYKKFKSYREMTESTTGEIPGVLLCDRHNYFKNFYTELKNAA 1669

Query 239 IEAINSDEYESSKDKAMDVMSSLKITPKIsevessslvpllsvelNCAFDVVLMAKETDI 298

+ ++S YE+ +D+ MD++ +LKI PKISE+ SS +L VE+NC FDVV + +

Sbjct 1668 YKILSSTTYENEEDQVMDLLDTLKIKPKISEMHSSEGKAMLFVEMNCDFDVVFAGSPSKV 1489

Query 299 YDHIIDYFRTML 310

Y IIDYF+ ML

Sbjct 1488 YRDIIDYFKVML 1453

**Conserved domains (not found)**

**
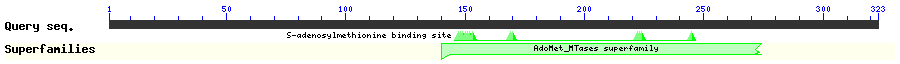
**

**Argonaute-3**

**>TRINITY_DN27717_c4_g1_i3 len=3258**

CCAAGCGAATTTTAGAAAAACGGCGCTAATTATATGTGCGTCCTTCATACGGTGCTTGTA

ACATCCCGTAAAAAGTGTGACGACTCTGCGATAATATTAAAGGTCTAAAAGTCAAATTAA

GAAATTTGTGTAAAATACTGTGGATAGCGTAGCCGGGATTGGACTGACCAATATTTTGTT

TTTAACCTCGAATGAAGATCGTGCCCACAGATAAGCGTTCCCCCTAGAAATACTACGGAT

AGAGGAGTTTAAATAGACTTCAATTTATTGCCCAAGGATCTTGGAACAAAATTATCTATA

TTTTCACGGATTGAATTAAGCGTTTGAAAAAGCAATGTCAGCACGCGGTCGCGGATTTCT

TTTATCCCTTAAAGTGGATAAAGATACCACTGATGGAGAAGGGTCTCTAAAAGACAGTGG

CCTAGGCAGCAGAAGTCTTAATTCGGGTGGAGTAGAGTTTCGTCGTGTCGGTCGTGGCAA

GCTTTTAGATGACCTGGCTTCTTCATGCACCAACATGACACTTGAGGGTCGTTCGTCTGA

TGATACGAATCAAGGGAATACAACTTCTACGACCGATTCTAGTAAACCTCTATCCGGGGG

TCGTGGCCGTGCCAATGTTTTCAAAAATTTATTTCGCGATGAGAAACCGGAACCACAATT

GGATGCTAAGCCTACTCCTGTTCCTGTTGCTGTTCCCGATGTTATTACAAAGGGCAACAC

CGTTACCGTTCCCGTTTCTATGCCAGTGGCAGTGCCAAAAGCGGAAGTTATTCAAGAGGT

AATGCCATCCCATATTTATAATCCAGAGGTGAAACATGGCAGCAAGGGTATGCCAGTCCG

TTTAGCGTGTAATTACATACGATTATCATCTGACCCCGAGAAAGGAGTGTTTGTATACGA

AGTGCGTTTCCATCCCCCAGTAGACTCGCTTAGTTTGCGCATGAAATACCTAAATGAACA

TCGTGATAAATTCGGCGGCACAAAGACATTTGACGGTGTGACACTTTATTTGCCCATTTT

ACTGAAAGATAAATTAACGACATTTATAAGCAAAAATATCGCAGACGATTCTGATATTGA

AATACGAATTTTATTCAAACGCAAAGAAGCGCTCAAAAATTGTATACATCTCTATAATGT

GCTATTCGATAGAGTTATGAAAACTCTGAACTATGTGCGCTTCGACCGAAAACAATTCGA

TCCAACAGCACCAAAAATCATTCCACAGGCAAAATTGGAAGTATGGCCCGGTTATGTAAC

CGCAATTGATGAGTACGAAGGTGGCCTGATGTTGTGTTGTGACGTTTCCCATCGACTTTT

ATGCCAGAAAACCGTTCTCGAAACATTAGTAGAGATCTATCGTTCCAACACCGCGTTATT

TCAAGAAAACGCTAAAAAGTATTTATTGGGTTCCGTTGTTATAACCCGCTACAATAACCG

AACCTATCGTATCGACGATATTTGTTTTGACAAAAATCCTAAATCAACTTTTCAAACTAA

ACAGGCGGAATTATCATACATCGACTATTACAGACAGAGTCATAATATCTTGATTAAAGA

TGAAACTCAACCATTAATCATTAGTATAAAGAAACAAAAAACCGCCGATAAGCAAGCGGC

TGAAGATTTAATTGTTTGCCTGATTCCTGAGCTATGCTATTTGACTGGTTTGCGTGATGA

AATACGATCGGACTATAAACTTATGCGAGAGATTGCCACATTTACTCGAGTATCGCCGAA

CCAAAGGCTGCTCGGATTGGAGAAGTTTTTCAATAACATAAACAAATGTCCAGAAGCTCA

AAATATACTTCAAAGCTGGGGACTAACTCTGAAGAATGCACATGAATGTGTGAATGGCAG

ACAATTTGAGGAAGAGCAAATTTTGTTTGCAAAAAAACAATTTTCAGCCGGTATAAATGC

AGACTTTTCCAAATATGTTGGCAACAATGAAGTTTTGGAGGTGGTTCACCTTACAAACTG

GTTATTGATTCACTGCAAAAATGATACCAGATGTGCCAAAAACTTTTGTGAACATATAGA

GCGAAACTCTCGTGCCTTAGGAATACGCGTAGATAAACCGAAAATTATTACTTTGGATAA

CGATAGGGTGGACACCTTTGTGAGAGCTTTGAGATTAAATATTGATGGGCAAACACAAAT

TGTTGTGTGTATTAGTCCTACGAATCGTGACGACCGCTATGCGGCGATTAAGAAAGTTTG

CTGCGCTGAAATACCGGTACCATCCCAGGTGATAAACGCGCGAACTCTTTTGAATGACGC

TAAAAATCGTTCGATCGTCGTAAAGATCATGTTGCAAATGAACTGCAAACTTGGTGGGTC

ATTGTGGGCCGTTAAAATTCCGTTCAAAAATGTCATGATTTGTGGAATCGATTCATATCA

TGATGCCGCACAAAAAGGCAACTCCGTAGCTGCTTTTGTAGCTTCTTTAAATTCTAATTA

TACCAAATGGTACAGCAAGGCTGTCATACAGGGCAAGAGAGAGGAAATCGTTAACGGGCT

TTGCGCGTCGTTTACAGCTGCCGTGACGCGTTTTCACAGAGAGAATGGCAGGTTTCCCGA

CAACATTATCATATATCGAGATGGTGTTGGTGATGGTCAATTGCCTCTTTGCTCTGGTCA

CGAGATACCGCAATTGGAGATTGCTTGCAAGCGTGCTTTCAAGGATTATACTGTCAAAAT

AACCTTCATCGTTATACAAAAGCGCATAAATACTCGATATTTTGCTATGAATGGAACCAA

CGCCGATAATCCGCCACCAGGTACAGTGGTTGACAATTCTATAACGCGTTCGAAGATGTA

CGACTTTTATTTGGTATCGCAGGCAGTAAGGCAGGGCACCGTCACTCCATCACATTACAT

AGTGCTAAGAGACGATGCCAAGTATAGCCCCGATATTATACAAAGACTTACATATAAACT

CTGCTTTATGTACTACAATTGGCCAGGCACTATTCGGATACCCGCTTGCTGCCAATATGC

GCACAAGATGGCATATCTCATCGGTCAGAGCATACGAAGGGCAACATCGGAAGAGCTTTC

AGACAGACTATTTTACCTTTAATTGTATTTCCGGTTATTCTTTATTAACACACTTTTGTA

TTGATTACCTTAAAAAAATTAGAATCAAAAGGGCTGGGACAATGGGAAAATTGAAATGAA

AATCATTAAAGCAAAGTAAATATGAATTTGTATCGGTCGTAATGTTGAGCATATCTAATG

ACCATGAAAATAAAAAAG

**tBLASTn(First hit)**

Score = 1056 bits (2732), Expect = 0.0, Method: Compositional matrix adjust.

Identities = 512/891 (57%), Positives = 652/891 (73%), Gaps = 47/891 (5%)

Frame = +2

Query 3 GRGNLLSLFNKNAGNMGKSISSKDHEIDSGLDFNNSESSGERLLSSHNIETDLITTLQHV 62

GRG LL + NM S D D N ++ TT

Sbjct 470 GRGKLLDDLASSCTNMTLEGRSSD-------DTNQGNTTS--------------TTDSSK 586

Query 63 NISVGRGRARLIDTLKTDDHTSNQFITSESKENITKK---TKG-------------PESE 106

+S GRGRA + L D+ Q + + TKG P++E

Sbjct 587 PLSGGRGRANVFKNLFRDEKPEPQLDAKPTPVPVAVPDVITKGNTVTVPVSMPVAVPKAE 766

Query 107 AIAS--ENGLFFPDLIYGSKGSSVNIYCNYLKLTTDESKGVFNYEVRFFPPIDSVHLRIK 164

I + ++ P++ +GSKG V + CNY++L++D KGVF YEVRF PP+DS+ LR+K

Sbjct 767 VIQEVMPSHIYNPEVKHGSKGMPVRLACNYIRLSSDPEKGVFVYEVRFHPPVDSLSLRMK 946

Query 165 YLNDHKDKLGGTKTFDGNTLYLPILLPNKMTVFISK--AEDVELQIRILYKKKEEMRNCT 222

YLN+H+DK GGTKTFDG TLYLPILL +K+T FISK A+D +++IRIL+K+KE ++NC

Sbjct 947 YLNEHRDKFGGTKTFDGVTLYLPILLKDKLTTFISKNIADDSDIEIRILFKRKEALKNCI 1126

Query 223 QLYNILFDRVMKVLNYVKFDRKQFDPSRPKIIPLAKLEVWPGYVTAVDEYKGGLMLCCDV 282

LYN+LFDRVMK LNYV+FDRKQFDP+ PKIIP AKLEVWPGYVTA+DEY+GGLMLCCDV

Sbjct 1127 HLYNVLFDRVMKTLNYVRFDRKQFDPTAPKIIPQAKLEVWPGYVTAIDEYEGGLMLCCDV 1306

Query 283 SHRILCQKTVLEMLVDLYQQNVEHYQESARKMLVGNIVLTRYNNRTYKINDICFDQNPTC 342

SHR+LCQKTVLE LV++Y+ N +QE+A+K L+G++V+TRYNNRTY+I+DICFD+NP

Sbjct 1307 SHRLLCQKTVLETLVEIYRSNTALFQENAKKYLLGSVVITRYNNRTYRIDDICFDKNPKS 1486

Query 343 QFEIKTGCTSYVEYYKQYHNINIKDVNQPLIYSIKKSRGIPAER-ENLQFCLIPELCYLT 401

F+ K SY++YY+Q HNI IKD QPLI SIKK + + E+L CLIPELCYLT

Sbjct 1487 TFQTKQAELSYIDYYRQSHNILIKDETQPLIISIKKQKTADKQAAEDLIVCLIPELCYLT 1666

Query 402 GLRDEVRSDNKLMREIATFTRVSPNQRQMALNKFYENVSNTPAAQEILNSWGLSLTNNSN 461

GLRDE+RSD KLMREIATFTRVSPNQR + L KF+ N++ P AQ IL SWGL+L N

Sbjct 1667 GLRDEIRSDYKLMREIATFTRVSPNQRLLGLEKFFNNINKCPEAQNILQSWGLTLKNAHE 1846

Query 462 KISGRQMDIEQIYFSKISVSAGRSAEFSKHAVTNEMLKVVHLSKWIIIHLRNYRQAATSL 521

++GRQ + EQI F+K SAG +A+FSK+ NE+L+VVHL+ W++IH +N + A +

Sbjct 1847 CVNGRQFEEEQILFAKKQFSAGINADFSKYVGNNEVLEVVHLTNWLLIHCKNDTRCAKNF 2026

Query 522 LDNMKQACESLGMNISNPTMISLDHDRIDAYIQALRRNITMNTQMVVCICHNRRDDRYAA 581

+++++ +LG+ + P +I+LD+DR+D +++ALR NI TQ+VVCI RDDRYAA

Sbjct 2027 CEHIERNSRALGIRVDKPKIITLDNDRVDTFVRALRLNIDGQTQIVVCISPTNRDDRYAA 2206

Query 582 IKKICCSEIPIPSQVINAKTLQNDLKIRSVVQKIVLQMNCKLGGSLWTVKIPFKNVMICG 641

IKK+CC+EIP+PSQVINA+TL ND K RS+V KI+LQMNCKLGGSLW VKIPFKNVMICG

Sbjct 2207 IKKVCCAEIPVPSQVINARTLLNDAKNRSIVVKIMLQMNCKLGGSLWAVKIPFKNVMICG 2386

Query 642 IDSYHDPSNRGNSVAAFVASINSSYSQWYSKAVVQTKREEIVNGLSASFEIALKMYRKRN 701

IDSYHD + +GNSVAAFVAS+NS+Y++WYSKAV+Q KREEIVNGL ASF A+ + + N

Sbjct 2387 IDSYHDAAQKGNSVAAFVASLNSNYTKWYSKAVIQGKREEIVNGLCASFTAAVTRFHREN 2566

Query 702 GKLPTNIIIYRDGIGDGQLYTCLNYEIPQFEMVCGNR-----IKISYIVVQKRINTRIFS 756

G+ P NIIIYRDG+GDGQL C +EIPQ E+ C +KI++IV+QKRINTR F+

Sbjct 2567 GRFPDNIIIYRDGVGDGQLPLCSGHEIPQLEIACKRAFKDYTVKITFIVIQKRINTRYFA 2746

Query 757 GSGIHLENPLPGTVVDQHITKSNMYDFFLVSQLVRQGTVTPTHYVVLRDDCNYGPDIIQK 816

+G + +NP PGTVVD IT+S MYDF+LVSQ VRQGTVTP+HY+VLRDD Y PDIIQ+

Sbjct 2747 MNGTNADNPPPGTVVDNSITRSKMYDFYLVSQAVRQGTVTPSHYIVLRDDAKYSPDIIQR 2926

Query 817 LSYKLCFLYYNWAGTVRIPACCMYAHKLAYLIGQSIQRDVAEALSEKLFYL 867

L+YKLCF+YYNW GT+RIPACC YAHK+AYLIGQSI+R +E LS++LFYL

Sbjct 2927 LTYKLCFMYYNWPGTIRIPACCQYAHKMAYLIGQSIRRATSEELSDRLFYL 3079

**Conserved domains**

**PAZ_piwi_like and Piwi_piwi-like_Euk domain-containing protein**

**
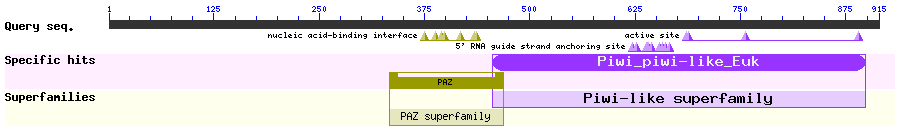
**

**Piwi**

**>TRINITY_DN30302_c0_g2_i1 len=3314** GGGTTTTATTTTAGAACGCAAACTACGATACATTTTAAAAGATGGAAATCGGAGAAAAGT

TGTATTACTTATTCTTTTAATCACTTAATCTCTTATCGAGTATAATACTCGTATCAATAA

ACTCAGCATATTTCTATTGATACAATAAATAACACAAGTAAATCGAATGATCTGCAAGTT

TATTTCTTTAAATTAAAAATTCAGGGTGACATAGTTTCAAAGTCGAGTCTGTTTTTGTTT

ACCCCACATCTATTTTTGCAAAAATTACATATAAATATATATGTATAAAATGACTTCAGT

ATTGATCGTTATTAATGCTATTAATGCTTCTTTAATTCATTTGGATTTTATTTCATTACT

TCGACCTTTTTTGTTACACATATTATATGTATGTTGTTTTGATTTCTTTCGAGTTCACTC

ATCTATATGGAATACATGTGCCTTTGCATTTCAAAAGTTAAAAAAAAACTGTTTTCACAA

GACATTTTGAAATCATGTGAAGAAAAATCGCTTACTAATACAGACTTGTTAATCATTTAC

AAAAAAAAGGAACCTTACAAATAATAGAGTTTTTTCTCAAGTGCGTTCTGAGGCGGTTGG

TAGAGACTTGTGGCCACTAAAGTTGCAAGCTTTTTAGCATATTGGCACACAGCTGGAACA

CGTGTTGTACCAGACCAATTGTAATACAAGTGAGTCATTTTGTATGTTAAAAGCTGCATT

TGATCTGGTGTTAAGCGAATATTGCTGTATACAATATTATAGCTTGTTGGGGACACTGTT

CCTTGTCTCACAGACTGAGATACCAAGAAAAAGTCATAACGTTCTGGACACGTAACTACA

TCATCGACTATTGTACCAGGAGGCGGATTTCGGCCTCGTGCAAAGAGTCGGGTATTAATA

TTTTTCGATACCACAACATATGCAAACATAGGTGGTTTTTCAGAGCCAACACGCTTATAC

TCTTGCTCGAGTTTCTCAACCACGTCCTTAACCTCGTGTTCGTAGACTTGTCGCAAGGAG

CCTTCACCTACCCCATCGCGATAGAATAGAATTCGCGTTGGTAATTTTCCGTCATGTTCC

TTGCGATATTGACGTAAAGCTTTAGTCATCATCGGCCAAAGACTATTCGCTAAAACATCG

TGGGAACTGCATTCTGCAACCGTGCTGTAGAAGGTGGCATTAGTTTTCATATCCATTGAT

GCCACCAATGCCCCAAACGCCTTTGATCGATCACGTGTACTTTTGGCTACATCGTAGCCA

ATTGTCATCAACCCGGAAAGTGGTAGGTCAATCATCCATGGGGTATAACCCAATTTACAA

TTGATTTGTATAGCTACCTTTGTGGCTATGCTCATAAGACCGCGCTGGTTATTTGCCGAC

TTTTGTGTTATTACTTGCGTTGGAATGGCTCGTTCCAAGCACCCTTTTTTCTTTATAGAA

GCGTAGCGCTCAGCATTATTATTCGGCACCAAACAAAGTATAAGTTGGGGGTCGTGACGG

CTGCAGTCCTCCATCGCCTGTATATACGAGTGATTTCTATCATCATATAACATAACTTCT

CGAGGGCGATTGATACGCATCTGCATTCCGCCAGCAGCACGAATTAAAGATTCGACGAAA

TTTCTTAATTCTCTGGAGTTACGATTTGTCGCAATAACAGACCACCTGTCAAGGCCACGA

GATGGAGTTGTAAACAAACCGTTATCGCGAAAGAGGCGAGTCCAGTCTGCTTGTTCACCG

GCAGATGCCTTTTTGTGTTCGCTGAATACGATACGTTGTGGCTCAAGAACACGACCATTA

AGTTCAACCAAATGGCGATCTAGGGTCATGTTCCAATCATTCAGTACTTGTATGCTGGCA

TCAGTCTGCTGAAGTCTATTATTAAATATTCTTAAGCGGTCAATACGACGATCAGGATTC

ATACGTGTATGATCTGCCATAGCACGCATAAGTTGGAAATTATTGCGCATGGTATCCGTT

AGACCGGTAGGCCTACAAAGTTCGGGAATAAGTATGATTATATCACTGCCCCCAGTTCTC

ATTGCCCTTTCTTTAGGCTTAGATATGAGCAAAGGCTGCTTTGGATCGCGTATGCGAATA

TGGTATTTTTGGTAATAATAATCAATGAAAGACACATCTTTCTCTTTACAACTAAATGTT

TTTGATGGATTTTTGTTGAAATCTACGTCATTAATGCGATAAGTTTTGTTATTGTAGTCA

GTTAGAACAGTAAGTCCTAATACATGTCGGCGAAATTCCTCTTGGTAATCTCTAGCAGTT

TCCGTGCATTTCATTAAAATATCATAAACAGTTTCTGTGCGCATAACTTTGTGAGCGATT

TCAGCACAGAGTAAAATGTCACTTTCATGTTGGCGGATTGATGTTTGATAACCCGGCCAG

AGTTGCAAACGGTACTCTCGCAGATCAATTTTTGCAATCGCGTCATAAAAATTTCTGCCA

ACTAACTGTAATTTGAGACCCTCCATAGCGCGACGCAAAATTAAATTCAAAATCTGAAGT

GATTGCCACTCCGTCATGGAAATTACACCAACGTATTTAATTACAATTGTGTAACTGTCA

CCGACTTTAGATTTCGCATGAATAATTGTTTTATCTTCCGGTAGCTTACATGAAGTAAAC

ATTTTTGTACCATCATATAAATATCCACCTAATGTTGAACGATGTTCAGAAAGAATACCA

CCCCGAACACGTCGCAGTTCAACTTTTGGTGTAAAATCAACGTGGTATTGGAATATGCGC

CAGGTTGGTTTGGTCAACAAACGATAGTAGTTAGCTTGCAATATTATTGGGGTGCCACCA

CTACCAATTTTGGATTGAATATCAGAAGGACGTGTATGCACAATTTCATAGCGCTCCTTC

TTCATGCCATGGTCCGCGCTGTCTCCACCATCTCCATCTGCACCAAAACCCCCACCACCT

GCAGCACCTGATGTTGATGGTTGATCTCTGGGCTCTCTGCGTCCATTTCCATTGCGCGAT

GAACTGGAACTTCTATCCGGTGAAACCGGTTCTTTTTTATACACTTTAGGAGGACGCGAT

TCGTCTGGCGATTGTCGCTCTCTGCGTCTTCGTTGAGAGTCACCGCCACCGGTATTGCTG

GAATAGGTATCTAGTGGCGAACGCGACCTAGAATACCGTTGCTCTCGTGATGGTCTACGC

CGACCACGACTATGATCATCAGACATACTTTCCGATATTATTAAAACAAATAAAAACTCT

GAAAATTAGTTTACCAGCGCGAGGTGACGCGAAGCTTAAGAAATTAGTAACGAAACATGG

AGTGGACTTTGTGT

**tBLASTn(First hit)**

Score = 1046 bits (2706), Expect = 0.0, Method: Compositional matrix adjust.

Identities = 486/774 (63%), Positives = 626/774 (81%), Gaps = 5/774 (1%)

Frame = -1

Query 74 DQYDYLNTRPAELVSKKGTDGVPVMLQTNFFRLKTKPEWRIVHYHVEFEPSIENPRVRMG 133

++Y+ ++TRP+++ SK G+ G P++LQ N++RL TKP WRI YHV+F P +E RVR G

Sbjct 2876 ERYEIVHTRPSDIQSKIGSGGTPIILQANYYRLLTKPTWRIFQYHVDFTPKVELRRVRGG 2697

Query 134 VLSNHANLLGSGYLFDGLQLFTTRKFEQEITVLSGKSKLDIEYKISIKFVGFISCAEPRF 193

+LS H + LG GYL+DG ++FT+ K ++ T++ KSK+ Y I IK+VG IS E +

Sbjct 2696 ILSEHRSTLG-GYLYDGTKMFTSCKLPEDKTIIHAKSKVGDSYTIVIKYVGVISMTEWQS 2520

Query 194 LQVLNLILRRSMKGLNLELVGRNLFDPRAKIEIREFKMELWPGYETSIRQHEKDILLGTE 253

LQ+LNLILRR+M+GL L+LVGRN +D AKI++RE++++LWPGY+TSIRQHE DILL E

Sbjct 2519 LQILNLILRRAMEGLKLQLVGRNFYDAIAKIDLREYRLQLWPGYQTSIRQHESDILLCAE 2340

Query 254 ITHKVMRTETIYDIMRRCSHNPARHQDEVRVNVLDLIVLTDYNNRTYRINDVDFGQTPKS 313

I HKVMRTET+YDI+ +C+ +Q+E R +VL L VLTDYNN+TYRINDVDF + P

Sbjct 2339 IAHKVMRTETVYDILMKCTETARDYQEEFRRHVLGLTVLTDYNNKTYRINDVDFNKNPSK 2160

Query 314 TFSCKGRDISFVEYYLTKYNIRIRDHNQPLLISKNRDKALKTNASELVVLIPELCRVTGL 373

TFSCK +D+SF++YY KY+IRIRD QPLLISK +++A++T S++++LIPELCR TGL

Sbjct 2159 TFSCKEKDVSFIDYYYQKYHIRIRDPKQPLLISKPKERAMRTGGSDIIILIPELCRPTGL 1980

Query 374 NAEMRSNFQLMRAMSSYTRMNPKQRTDRLRAFNHRLQNTPESVKVLRDWNMELDKNVTEV 433

MR+NFQLMRAM+ +TRMNP +R DRLR FN+RLQ T S++VL DWNM LD+++ E+

Sbjct 1979 TDTMRNNFQLMRAMADHTRMNPDRRIDRLRIFNNRLQQTDASIQVLNDWNMTLDRHLVEL 1800

Query 434 QGRIIGQQNIVF-HNGKVPAGENADWQRHFRDQRMLTTPSDGLDRWAVIAPQRNSHELRT 492

GR++ Q IVF + K AGE ADW R FRD + TTPS GLDRW+VIA RNS ELR

Sbjct 1799 NGRVLEPQRIVFSEHKKASAGEQADWTRLFRDNGLFTTPSRGLDRWSVIATNRNSRELRN 1620

Query 493 LLDSLYRAASGMGLRIRSPQEFIIYDDRTGTYVRAMDDCVRSDPKLILCLVPNDNAERYS 552

++SL RAA GM +RI P+E ++YDDR +Y++AM+DC R DP+LILCLVPN+NAERY+

Sbjct 1619 FVESLIRAAGGMQMRINRPREVMLYDDRNHSYIQAMEDCSRHDPQLILCLVPNNNAERYA 1440

Query 553 SIKKRGYVDRAVPTQVVTLKTTKN-RSLMSIATKIAIQLNCKLGYTPWMIELPLSGLMTI 611

SIKK+G ++RA+PTQV+T K+ N R LMSIATK+AIQ+NCKLGYTPWMI+LPLSGLMTI

Sbjct 1439 SIKKKGCLERAIPTQVITQKSANNQRGLMSIATKVAIQINCKLGYTPWMIDLPLSGLMTI 1260

Query 612 GFDIAKSTRDRKRAYGALIASMDLQQNSTYFSTVTECSAFDVLANTLWPMIAKALRQYQH 671

G+D+AKSTRDR +A+GAL+ASMD++ N+T++STV ECS+ DVLAN+LWPM+ KALRQY+

Sbjct 1259 GYDVAKSTRDRSKAFGALVASMDMKTNATFYSTVAECSSHDVLANSLWPMMTKALRQYRK 1080

Query 672 EHR-KLPSRIVFYRDGVSSGSLKQLFEFEVKDIIEKLKTEYARV-QLSPPQLAYIVVTRS 729

EH KLP+RI+FYRDGV GSL+Q++E EVKD++EKL+ EY RV PP AY+VV+++

Sbjct 1079 EHDGKLPTRILFYRDGVGEGSLRQVYEHEVKDVVEKLEQEYKRVGSEKPPMFAYVVVSKN 900

Query 730 MNTRFFLNGQNPPPGTIVDDVITLPERYDFYLVSQQVRQGTVSPTSYNVLYSSMGLSPEK 789

+NTR F G+NPPPGTIVDDV+T PERYDF+LVSQ VRQGTVSPTSYN++YS++ L+P++

Sbjct 899 INTRLFARGRNPPPGTIVDDVVTCPERYDFFLVSQSVRQGTVSPTSYNIVYSNIRLTPDQ 720

Query 790 MQKLTYKMCHLYYNWSGTTRVPAVCQYAKKLATLVGTNLHSIPQNALEKKFYYL 843

MQ LTYKM HLYYNWSGTTRVPAVCQYAKKLATLV T+L+ PQNALEKK YYL

Sbjct 719 MQLLTYKMTHLYYNWSGTTRVPAVCQYAKKLATLVATSLYQPPQNALEKKLYYL 558

**Conserved domains**

**protein containing domains ArgoL1, PAZ_piwi_like, and Piwi_piwi-like_Euk**


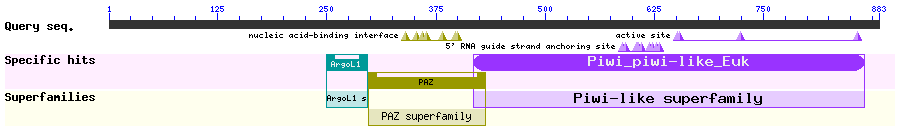


**Aubergine**

**>TRINITY_DN30302_c0_g1_i1 len=3183**

ACATAACATTTATATAGAATATTCTATACGGTTAAAGAAACAAACACATATGTAAAAATG

TGTTACAAAATTTCGGTACACACATTTTCAAGTATTTTTTGGCATAGTATAATAAATCAA

AGTATAATAAATCAAAATATATGGCATCTCTAGGAAGCTTGTGAAGCCTTTTGGCGTGGC

TGGATTCTACGCGATGGATAAGTTAACAAATGCTGGACGCTCAAGAGGTCGTGGTCGAGC

CTCTAATCCTCATCAGCCTCGTGGCGATGCACGAAGCCGTCGAACTGGTCAACCAGGACT

GCAAGCGGAAGCTAGTGGTGGTCCAGGTCCTTCAAGAAATTTGCCACAAGCACCTCCAAC

GTCTGCTTGGGGACAACCCTTACCAGGTGTAGCGCGTGGTTCAACTATTGCACCAAAACC

AAGCTGTAGTACTGCTCCCGTTCAGCAACGTCCGGCTGCACCAGTTATGCAAGGCCGAGC

AACTGCTCACAGAGGAGCTCCAATAGGTGATGCACCAGTTGTTAAGGAAGATTCCCGTGG

AGCAGTGCGTGGGAAGCGTGTGTTACATGAGGTAGTATCTTCGCGCCCAAGCACCTGTAT

TACCAAAACAGGCTGTACTGGAAAGAAAGTTGTGATCCAGACTAACTATTTTCGAGTTTT

AAAAAAACCGCAATGGTCCATTCACCAATACCGGGTGGACTTTGCTCCTGACGTCGACAT

GATACGCTTAAGACGTGCCTATTTAGCTCAACATAAGGAAACATTCGGTGGATACATATT

CGATGGCACTATGTTATTTTGTACAAAGTACTTAGAAAAGCCACAAATGGAGCTTTTAAC

TAAAAATCGTGAGGGTGAAACTATCCAAATAAAAATTAAACACGTTGGGCAATTGGAAGT

GACTGATTCGCAGCAGCTACAAGTGCTAAATCTTATTTTACGTCGTGCTATGTCAGGGCT

GAATTTAGAACTTGTTGGGCGTAGTTTCTTTGATCCGAAGGCAAAGCATTGTCTAAACAG

TTTCTACCTCGAATTATGGCCTGGATATCAAACTTCAATACGCCAACATGAGCAAGACAT

TCTCTTATGTGCTGAAATTGCTCATAAAGTAATGCGCACTGATACGTTATATAAAATTCT

ACAAGATTGCGTCGGTAAACCAGACTTCCATGATGCTTTTAAAAGAGAAGTCGTTGGAAC

AATAATCCTCACCGACTATAATAATAAAACATATCGGATAAATGACGTGGATTTCCAACA

ATCGCCCAAGTCGAAATTTGCAACCAAAGAAGGAGAAATTTCGTATATTGACTATTATAA

AAAACGTTACAATATTAAAATATCAGATGCAAACCAACCACTCTTGATGTCTCGCCCAAC

TGAACGTGATATACGTGGTGGCATAGACGACTTCATTATGTTAATTCCTGAGTTATCACG

CGCAACTGGACTTACTAACGCTATGAGAAGTAACTTCAGCTTGATGAGGGCAATGAGTGA

ATTCACCCGTTTGGCCCCTGAAAGACGTATTGAACGATTGCGAGTATTTAATAGGCGTTT

GAATCAGGCACCTGAAAGCGTTCAGGTCTTAGAGTCTTGGAGTATGCGATTAGATACAAA

TCTCGTTGAAGTACCTGGTCGTATTATACCAGCTAACAGAATTGTTTTTGGAAACAATAA

ACGTTACGATTGCAATGAATTTGCAGATTGGACGCGAGAATTTCGAAATAATTCAATGTA

CAAACACGTTGACATTAAGAGGTGGTACGTGATTACGCCATCACGGAATTTACGTGAAGC

TCAAAATTTCGTACAAATGTGCATAAGAGCAGCTAATGGAATGCGTATGGGCATTGCAGA

GCCTATATATCAACAAATTAGTGACGATCGTAGTGGATCTTATTCACAGGCAATCAATAC

CGTTTCTACTGGAGATCCTCAAATCGTGATGGTAGTTCTGGTATCTGCAAATGAAGAAAA

ATATGGTTGCGTCAAAAAGAAGTGTTGCGTTGACCGTCCAATGCCATCTCAAGTAGTGAC

ACTACGTACTATTGCCCCTCGTGGAGACAAAGCCGCTGGTTTGATGTCTATAGCTACTAA

AGTTGTTATACAAATGAATGCAAAACTCATGGGAGCACCTTGGTTAACCGAAATTCCAGT

AAGTGGTCTAATGACTGTTGGTTTCGATGTTTGTCATTCGCCCAGAGAGAAAACTAAATC

TTATGGCGCATTAGTAGCCACAATGGATCTGAAGTCGAAGCCCATTTACTTCTCTTCAGT

TTCAGAACATGTGAAAGGCCAGGAGCTATCAAACGAAATAGCATTGCGCATGGGCTATGC

TTTGAAAGCATATCAACAAGAGCATGGTATGTTACCAAAGAAAATCCTCTTCTATCGCGA

CGGAGTCGGTGATGGGCAATTGCATCAAGTTTTCAGCACCGAAGTGAAATTTTTGATAGA

AAGGTTAAACAAAATGTATTCTGATTATGAGAAGAAACCTACGATTTGCCCCTTGGCGTT

TATAGTGGTATCGAAGCGAATTAATACTCGTTACTTCATTAATGGCAGGAATCCACCTCC

GGGTACCGTGGTTGATGATGTGATCACTCTACCGGAACGTTATGACTTCTTTTTAGTTTC

CCAATCAGTGCGTCAGGGAACTGTTTCGCCTACTAGTTACAATGTTATTTACGACACCAT

GGGTTTCGATGCCGACAAATTACAAATGTTAACATACAAAATGACCCATCTGTATTATAA

TTGGAGTGGCACTTGCCGTGTACCTGCCGTGTGCCAATATGCGCACAAACTTGCATTCCT

TGTCGCTGAGAGCATTCATCGTTTGCCAAGCAACGCTTTAGAAAAGCAACTTTATTTCCT

TTAAACAGTTTTGAATGTTTATATATTTTATACATAATGAGATAAACTACATAAATTTTA

TCCATTTCCATTTTTCTTATATTGTTTTTTTTATACATAAGTGCAACATCAATATTTTAT

TTTCATTTATTGGCATTACTTGTTATTTGAATTGCATTTGTTTGCGTATGCTCTCATACG

AATAAACGTTTATTGGAGTTCTTCATTTATGTTCCGACTTACTAAAACATAATTATCATA

TGTAAACAAACGTTAAGCTTTTTATATTGAAAAATAAAAATGAATTTTCGTACAATGAAA

AAA

**tBLASTn(First hit)**

Score = 1081 bits (2795), Expect = 0.0, Method: Compositional matrix adjust.

Identities = 506/790 (64%), Positives = 627/790 (79%), Gaps = 7/790 (1%)

Frame = +2

Query 78 DPRGSVRGRRLITDLVYSRPPGMTSKKGVVGTHITVQANYFKVLKRPNWTIYQYRVDFTP 137

D RG+VRG+R++ ++V SRP +K G G + +Q NYF+VLK+P W+I+QYRVDF P

Sbjct 530 DSRGAVRGKRVLHEVVSSRPSTCITKTGCTGKKVVIQTNYFRVLKKPQWSIHQYRVDFAP 709

Query 138 DVEATRLRRSFLYEHKGILGGYIFDGTNMFCINQFKAVQDSPYVLELVTKSRAGENIEIK 197

DV+ RLRR++L +HK GGYIFDGT +FC + Q +EL+TK+R GE I+IK

Sbjct 710 DVDMIRLRRAYLAQHKETFGGYIFDGTMLFCTKYLEKPQ-----MELLTKNREGETIQIK 874

Query 198 IKAVGSVQSTDAEQFQVLNLILRRAMEGLDLKLVSRYYYDPQAKINLENFRMQLWPGYQT 257

IK VG ++ TD++Q QVLNLILRRAM GL+L+LV R ++DP+AK L +F ++LWPGYQT

Sbjct 875 IKHVGQLEVTDSQQLQVLNLILRRAMSGLNLELVGRSFFDPKAKHCLNSFYLELWPGYQT 1054

Query 258 SIRQHENDILLCSEICHKVMRTETLYNILSDAIRDSDDYQSTFKRAVMGMVILTDYNNKT 317

SIRQHE DILLC+EI HKVMRT+TLY IL D + D+ FKR V+G +ILTDYNNKT

Sbjct 1055 SIRQHEQDILLCAEIAHKVMRTDTLYKILQDCV-GKPDFHDAFKREVVGTIILTDYNNKT 1231

Query 318 YRIDDVDFQSTPLCKFKTNDGEISYVDYYKKRYNIIIRDLKQPLVMSRPTDKNIRGGNDQ 377

YRI+DVDFQ +P KF T +GEISY+DYYKKRYNI I D QPL+MSRPT+++IRGG D

Sbjct 1232 YRINDVDFQQSPKSKFATKEGEISYIDYYKKRYNIKISDANQPLLMSRPTERDIRGGIDD 1411

Query 378 AIMIIPELARATGMTDAMRADFRTLRAMSEHTRLNPDRRIERLRMFNKRLKSCKQSVETL 437

IM+IPEL+RATG+T+AMR++F +RAMSE TRL P+RRIERLR+FN+RL +SV+ L

Sbjct 1412 FIMLIPELSRATGLTNAMRSNFSLMRAMSEFTRLAPERRIERLRVFNRRLNQAPESVQVL 1591

Query 438 KSWNIELDSALVEIPARVLPPEKILFGNQKIFVCDARADWTNEFRTCSMFKNVHINRWYV 497

+SW++ LD+ LVE+P R++P +I+FGN K + C+ ADWT EFR SM+K+V I RWYV

Sbjct 1592 ESWSMRLDTNLVEVPGRIIPANRIVFGNNKRYDCNEFADWTREFRNNSMYKHVDIKRWYV 1771

Query 498 ITPSRNLRETQEFVQMCIRTASSMKMNICNPIYEEIPDDRNGTYSQAIDNAAANDPQIVM 557

ITPSRNLRE Q FVQMCIR A+ M+M I PIY++I DDR+G+YSQAI+ + DPQIVM

Sbjct 1772 ITPSRNLREAQNFVQMCIRAANGMRMGIAEPIYQQISDDRSGSYSQAINTVSTGDPQIVM 1951

Query 558 VVMRSPNEEKYSCIKKRTCVDRPVPSQVVTLKVIAPRQQKPTGLMSIATKVVIQMNAKLM 617

VV+ S NEEKY C+KK+ CVDRP+PSQVVTL+ IAPR K GLMSIATKVVIQMNAKLM

Sbjct 1952 VVLVSANEEKYGCVKKKCCVDRPMPSQVVTLRTIAPRGDKAAGLMSIATKVVIQMNAKLM 2131

Query 618 GAPWQVVIPLHGLMTVGFDVCHSPKNKNKSYGAFVATMDQKESFRYFSTVNEHIKGQELS 677

GAPW IP+ GLMTVGFDVCHSP+ K KSYGA VATMD K YFS+V+EH+KGQELS

Sbjct 2132 GAPWLTEIPVSGLMTVGFDVCHSPREKTKSYGALVATMDLKSKPIYFSSVSEHVKGQELS 2311

Query 678 EQMSVNMACALRSYQEQHRSLPERILFFRDGVGDGQLYQVVNSEVNTLKDRLDEIYKSAG 737

++++ M AL++YQ++H LP++ILF+RDGVGDGQL+QV ++EV L +RL+++Y

Sbjct 2312 NEIALRMGYALKAYQQEHGMLPKKILFYRDGVGDGQLHQVFSTEVKFLIERLNKMYSDYE 2491

Query 738 KQEG-CRMTFIIVSKRINSRYFTGHRNPVPGTVVDDVITLPERYDFFLVSQAVRIGTVSP 796

K+ C + FI+VSKRIN+RYF RNP PGTVVDDVITLPERYDFFLVSQ+VR GTVSP

Sbjct 2492 KKPTICPLAFIVVSKRINTRYFINGRNPPPGTVVDDVITLPERYDFFLVSQSVRQGTVSP 2671

Query 797 TSYNVISDNMGLNADKLQMLSYKMTHMYYNYSGTIRVPAVCHYAHKLAFLVAESINRAPS 856

TSYNVI D MG +ADKLQML+YKMTH+YYN+SGT RVPAVC YAHKLAFLVAESI+R PS

Sbjct 2672 TSYNVIYDTMGFDADKLQMLTYKMTHLYYNWSGTCRVPAVCQYAHKLAFLVAESIHRLPS 2851

Query 857 AGLQNQLYFL 866

L+ QLYFL

Sbjct 2852 NALEKQLYFL 2881

**Conserved domains**

**
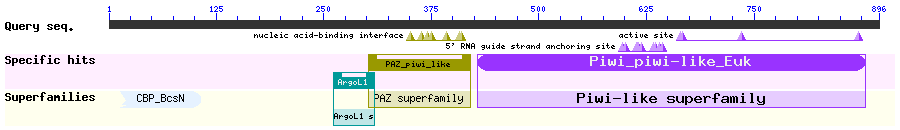
**

**Zucchini**

**>TRINITY_DN31164_c0_g2_i2 len=1520**

TGAGGGGCTGGCAGCACTGCTCAATCGTACTACAGCTGATTCGGCTGTCAAACGAACCAA

ATTTACTTGCCTTGAACGAATAACACGTGCATTTTTGACCGCTGCATCTCGCGAATCTAT

TTTTTGCAACTCGTAGCATTTCCGTTTGGCTGCAATACGCAACAAACTCCAATAAAAGCA

GAGTATTTATTATCCAAATTAATTGATTGTGTGTGCGACAAAAAGTGAGTTGTGAATAAA

AATATTGAAATTAATGTGATTTTTAAGCAATAAATTGATCAAATTTGCATGTGAAAAGAA

ATCCAGTCAAAGTTTATTTAAAAGAAAATGATGGAGAATCGGGTGTTGCGCTACACTTTT

TATAGCCTGACAACGGGCATATCGGTAGTGTTGTTGTCGGAAATTTTATATCGTGGCTAT

TTGTACGTGCGCGAACGAATGTGGCGACGCGATTCATCTGACGATGAAGTGAACGAAGTC

ATCTGGACGAATGAGTTGACACAAAGTTGTGCTGCTGGACACATATTAAAGTCCCCGTCC

GAACGCAAGAGTCCTACGCAGGAGAATCCATCGAAAGGCATGGTCGAGAATGGACATATT

ACAAAACTACCAGTCCCCAGTACGAGTCCTACATGTCAAAATCGGTTTTGTGCTTCTAAA

AATGTCGGCCGTTTAATATCGTTAATTGATAAATCAAAACATACAATAGATCTAGCCATG

TATACGTTCACATCCTACGATCTGTCGCAGGCCTTCATGCGAGCCATTCGTAGAGGAGTG

TCTGTGCGTATAATAAGTGACAAAGAAATGGTATACTCCAGTGGATCACAAATTATAACA

TTGACAAAAGCAGGCGTTGCGGTTCGTTGTCCCATGACTACAATGCTAATGCACCATAAA

TTTTGTTTGTTGGATGGTCCAAGGCGCGCGCGTACGGTTTTGGCCAATGTTGGAAAAACG

TACGATTCAAAAACTGCCAAGGGTATCGTCATGTCTGGCTCACTAAACTGGACGATGCAG

GGCTTTGGCGGTAACTGGGAAAATATTGTAATCAGCTCGAATAAAGTGTTATTGGAACAA

TTTGAGGATGAATTTGAACGTATGTGGCTAACGTTCGCACCAAAAATGAGTACTTAAACA

TGTAAAACGAAAGATCTCCAATCAACTCTGAAGTTCAATTGCAGTGTGTATTACTGAAAT

TCCCTTAGCTTTACGTAAATCTTAGCATAGTGTTTAAAGAGAAAATGCTCGAAATCCAAT

ACTATGAATTTTTTAATTTTAAAATTATATTTGATAAGCCCAAATCGCTCAGGTCCTATC

TATGAAATGTGAGTACAAATGTGTAAAAATAAAAGTCTATTAAATTAAGTGCCACGATAA

GTGTCTTACTAAACACCCACCGCCACATCTCGTCGAAAAGCAGCAACAGTTATCATATTA

AGTAATTACACCAAAGCTTAAGCATTAGCACTTACATCCCCGTTATCAAGTTTTCTACTG

CAAAAGTGTATTCGCAGCAA

**tBLASTn(First hit)**

Score = 183 bits (465), Expect = 3e-053, Method: Compositional matrix adjust.

Identities = 105/252 (42%), Positives = 150/252 (60%), Gaps = 27/252 (11%)

Frame = +1

Query 19 TISIAVSTVLASEVIWK--LVQCSRSKREKAS--RVHEVIIFNELGEICAAVHM------ 68

+++ +S VL SE++++ L R R +S V+EVI NEL + CAA H+

Sbjct 364 SLTTGISVVLLSEILYRGYLYVRERMWRRDSSDDEVNEVIWTNELTQSCAAGHILKSPSE 543

Query 69 ------RNSSMGSXK---------PQVSPCC-NTHCSLRNVAKIVEQIDRAVYSIDLAIY 112

N S G + P SP C N C+ +NV +++ ID++ ++IDLA+Y

Sbjct 544 RKSPTQENPSKGMVENGHITKLPVPSTSPTCQNRFCASKNVGRLISLIDKSKHTIDLAMY 723

Query 113 TFTSLFLADSIKRALQRGVIIRIISDGEMVYSKGSQISMLAQLGVPVRVPITTNLMHNKF 172

TFTS L+ + RA++RGV +RIISD EMVYS GSQI L + GV VR P+TT LMH+KF

Sbjct 724 TFTSYDLSQAFMRAIRRGVSVRIISDKEMVYSSGSQIITLTKAGVAVRCPMTTMLMHHKF 903

Query 173 CIIDGFERVEEIRL-LRKLKFMRPCYSIVISGSVNWTALGLGGNWENCIITADEKLTATF 231

C++DG R + + K + IV+SGS+NWT G GGNWEN +I++++ L F

Sbjct 904 CLLDGPRRARTVLANVGKTYDSKTAKGIVMSGSLNWTMQGFGGNWENIVISSNKVLLEQF 1083

Query 232 QAEFQRMWRAFA 243

+ EF+RMW FA

Sbjct 1084 EDEFERMWLTFA 1119

**Conserved domains**


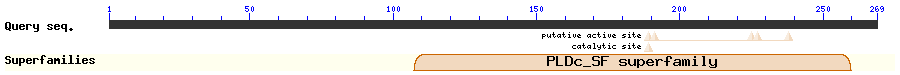


**Tudor-SN**

**>TRINITY_DN30816_c0_g1_i2 len=4675**

CACCGAGATTCGAACCTACGTTCTCTCTGAACTCCAAATGGTGGTCACGCACCAACTCAT

TCGGCTACGGCGGCCACCGTCGATTAATTTATTGAAGTTTTAAATATTATGGTTATAAAG

TACAAGGTATTTCTCTTGTGACAACACTTTTGAAGATCTGTTTACAGTTTTCACTGAGAC

CTGACTTTATCGACCGGCGATAGGCGTAAAATTAAAAATAAACAATTCTCGACGAATTTC

ATCGTCGCTTTCGTTGTGTGCAATCTCATATACTTCTGTGTGTTTTAATTTTTACAATGA

AATTTCGTCCGAGAAAATTCGCTCTGCGGTTTGATTTTTTTGTACATTTAGGAAGAATTA

TTAAATGTAATAGCTGTTAGGATAATTGAGTCAATGAAAAAAGATGCGATCATGCTTAAA

GTGCTCTTTGGAGTTGGTCGTTTGAAGGGGTATTTAGCCTCAACGAATCGATTTATTTAT

AGACTAGAAAATGAAGTTATATTGCAGAAATCTAGGTTCCCTAACGGAAGGCTTGGGAAG

CATTTCTAGCAGGAATATCTATTACAAGTTGATTTGTAAGTGGAACAGAACTTTAGGGCG

TGTGCAAGCTGCAATAATTGAGGGAGCGTTGCAATTTATTGGCTCGGCCAAACACATCCC

TATTTCCCACACTAATCATTTCACAATGCTGCATAACTTCTAATTCCTTATCAATCGATG

GGTAATGTTGCAATATATGATTTATTATACGAATGTGAATCCAGAATGTAAAGCTACTAT

TCCTCGACCTAATGTGTTATTTATTTAATATTTTTCCTTTTATAATTTAATATAAGGAAT

GGCTGTATACAAATGGCAGGTTCTCATTTTATAGATTATTTAGAAAAACAATTAATTGAA

TTCATAAAGAGTATGTTCTCAATTTTTTAATAAAAAATTATAGTAAAGAAAAATATATAA

TCATAAATGTTGGATTTTTGTTTATAAAGGTTTTGTAGTTGAATTTGAAAATGTTTAAAG

TTTTTTTTTTTGTAGATTTAACAGCATACATAAGCAATAATAGCCGCGAGTGCGCGCCTT

GAAATATTTCAATTTTCTGATTTCCGTTCTTACAGGGAAGCTTTAGGATATTAGGCATAC

TATGTATAATAGAACAATTCATGTCTTAAAAAATTTTTTTGATTTGTTTTATTTATTTGT

TTTACAATTTTAGTGGCCATGGAAGTATGTATTTATATGCGAAGCTTGTGTGGACACTTC

TGCATTGGCATGATAAGACATCGATCTTTAAGCCCAGCTGTAATGCATTTATGCATTGCC

GACGTTGTTGTTGTGTACAGATTATTTTGTTTTATTTTTAATGTACAACTACGTCGCTCC

TGGTGTGTGCATGTAATATTGTATACATGTTCGTTCTGGGGTTTTTTTTCACTTCATATT

ACTTTATGAATATCTGTTCGAACTTTACTCTACTTGAGTATGTTGTTTTGAATCAATTCT

TGTCAAGTTACTGTAATTTTTCTTACTATACCTCATTTTCATTTATTTTTCGTTATTTTT

TCATTTATGTATGTAGCTATGAATGTTGTTGTTTTTGTAGGGCGGGTATGTGACAGACAA

ACATTTAAAACGCTATCGCGCACGTCCGTTTTAGCGGCTAAATTCAGGCGCATCGTCCTG

AGTGATGTCACCATATTTCCAAATGCCCAAATGTGCAGTAAGCGCGGCTTGCTGGGCAGC

CCTATACTGCTCCACCAAATCTTTAAGCTTACGTTCGCGACGTTTTTCGGCTAACACATA

GCCATCAGCTACCAATTGCTTACCAATATCGGCATTGGTAGCTGGGTCATGCACCGTTGC

CAATGCCGGCCCAGTGGCAGGTTTTAGTTCGACATTCAATTTTACAGTGCGATTGAGCAC

ATCCTCGGCAAACAATCGTAGTGCTTCTTCCTTGTCTTCATTATCGGAGGGAAGTTGAAC

CAAAGCAAGCGCATACTCGGTGGCATACGGCCTGTCGCTGGTGAATGCAGAAGGCAGGGC

TGCCAAACGGTTTGTAGGCACAGTCTCTTTGTTGCCATAGTCGATATAGAGCACAGTAGC

ATTATTGCCTTGAACACGTTCCACCTTGGCACGATACCACTGATTGTCGGCAGAAAATTG

AGCTGCGCATACATCTCCGCGCTTAGTAGTGTAAGCGCCAACTATTGGCGGGTTGACTTG

GAAGTCAGCATGCAACTTGGCCATCATTGCCTCCAACTTCGCTCCATTCTCAACGGCTTG

CGCAAAGAATGTCAAGTCAGCAGTAATCTCGGTTACAATAACATCCTCGTAGTTCACTTT

ACGTTCTACGGGTGCCTTTTCATCTTTCTCCTCTTCAACCACAACTGGTTTCTCTTCAAC

GACTTGTTCTACGTAATTTGCCCATATATTCTTCTTGGCAGCCTTAGCGCGATCTTCGGC

ATTCTTTAGTTGACGGTAATATTCAGATTTTTCAGCGCTAAAGTGCACTTCGGCGAGCCC

TTCTTCGACTAATGCCACTGACAAATTCACATTGTTATCCGTCCAAAGCCAACCGATAAC

TGAAGATCCGGCCTTGTCTGTGGTATCAATATGCACTGAGACATCCCGCTGTAAAACTCG

GTCACGTGTGAAAGCTAAGGCCTCCTCACCGTAGGGTTCACCTTCTTGTGCTGGACTGCC

ATTCAGTGCAGGACGTGAGGAACGCGGACATGAAATGCCAGCCAGTAGGAATGTCACCAG

GCAGCTATCCTTCGGCACGTACAAACGTAGACGTGAACCACTTGCAACGAACTCAACTAT

ACCCTCGGTACGCAGAGCCCGCTGCCAAGATGGTAGATACTGTACTTTGATGCGGGAATG

ATCAACAGTCAAGTCATTAATACGTAATGGCACGTTATCCTTTTTGCCAAACATACCCTT

TTGACCCTTGATAGCCTGGCTTTCTGCAGCTAGAAGCTGATCGTATACTGAAGAACGCTG

ATCATCATCTTGACGATGACGTACGCAGTTGGCTAGACCCTTCGCGACCATAGCTTCAGC

GACATTCTGTCCGCCCACCAAAACGGTGTAGCAATACTTCTCGGGGAAATTATCGCGAGC

TGGCGAAATGTAATCCAAATTACATTGTACTTTTTTATTGATCAGTTTTTTGCGCAAAAA

TTCACGTGCTTCAAACATAAAGGGAATCTCATATAGTGGGCGGTAGTTTTTACCGCGTGG

AGGTAACACAACCTCACCATCAGCACCGACACCGGAGCGAGTGTCACGTGGAGGACGAAT

CGAAGAGAAGAATATCTTCTTGACTAGTCCATTTGACAGGCGGACGCTTATGGCATCGCC

ATTAAACACTTCTACTACAGTGCCAGTGAAATCTTTTTCCTTAGCATTCACAGTAGGCGC

CTTGGATTGGTAATCTTGCCACAAACGCAGTCGTTTGTCTTTAGCAGTTCGTTCGGCAGC

ACGTAACTTATCGGCACCACTCTTCATGACGGCCATCGACCAGTCGACACACTTAGCCAA

ACCCTCGCGCAGCAACGATTCGGCAATGTTGCCTTTTGGGAATATGATTGTGCCAATAAA

ATTTGAATTGTTGACGGACTCAAGACGTATTTCAACTTCACGCTGAAGTAAACGCGATTC

CACGAAAAAACGCGCCTCATCGGCAAATGGAATTTTCACACTCAGATCCGGTTTGCCATC

CGCATCAAGTTTCACTCCTGGACAACGGATACCCGAAATCATTAAAGTAATGTATTGGAA

TTCAGGCAGCAAGAAGGCACGAACCGTCGACCCATCTCGAACATGTTCAATAATAGCTTT

AACGGGTTTACCACCATAATATTCAACAAGATGAGCAGGATTTTCTTGTGTCCATTTAAT

ATAGCGCACCTTGTCAACAGTTGGAACATGTGACCATCTGCCGCGATTCGCCGACTTTGC

TTGATCTTCTAGCTCGATAAGACGTTGCAGTTCGCTAGATGGTCTACCCTCCCGGCGGAC

TGTCACAAAACCTTCGCGTACCATAGTCTCGACAACATTCTCGCCGCTTTCAGCATCCTT

ACCGAGCCAAACAAAACCATACTCCCGATTGGAATTGGTAGGTTTCTCAAAGGAGAATAA

AACTTCTTCGCCGATGAGTTTCTTCCGTAGAAATTCGCGGGAATCCCAAGCCCAGGGTTC

GTCTTTAGTTTCATCGCCTCCGGCGCCTGGGCGACGTGCTAGCTTGGGTGCAAGAACATA

CGAGAAAGTGATTTGTTTTTCCGGTGGTGGCGCACCTTTTTGGGCACGAATAACAACGGT

GTCACCAGAAAGAACCTGTTTCACGACACCACGCCGCTTTGGTACAACCGCTGCTGAATC

GCCATCCTTATTTGTCGCCGTGGCTGGAGCTGTCGCTGATGGTCCACTCATTTCGCTTCA

ATATAACTTGCTTGCTATAGCGGGTATTTATGCACTGAAAATTCGTGACGATTGCACTAT

TTAATTAAGGTTTTTCTTGCTTTCTGCAATTAAAATATAATTGAAGACGCGGAAAAAATT

ATTCTGCTTGAACTCAATGTTCACCTTTCTGCTTCTGCTTCGTCCGTTCCACGTTTGCAC

GAGTATTAACACACCACCGCCCGTGAAATTTTGCTTCGGTTTGACAGCAGTCAGG

**tBLASTn(First hit)**

Score = 1503 bits (3892), Expect = 0.0, Method: Compositional matrix adjust.

Identities = 736/900 (82%), Positives = 815/900 (91%), Gaps = 4/900 (0%)

Frame = -2

Query 27 GIVKQVLSGDTVVIRATKGAPPPEKQITFSHVLAPKLARRPGAGGDETKDEPWAWESREF 86

G+VKQVLSGDTVVIRA KGAPPPEKQITFS+VLAPKLARRPGAGGDETKDEPWAW+SREF

Sbjct 4350 GVVKQVLSGDTVVIRAQKGAPPPEKQITFSYVLAPKLARRPGAGGDETKDEPWAWDSREF 4171

Query 87 LRKKLIGVEVTFTFDKPANSNREYGFVWIGKDKETGENVVESIVREGLVSVRREGRPTAE 146

LRKKLIG EV F+F+KP NSNREYGFVW+GKD E+GENVVE++VREG V+VRREGRP++E

Sbjct 4170 LRKKLIGEEVLFSFEKPTNSNREYGFVWLGKDAESGENVVETMVREGFVTVRREGRPSSE 3991

Query 147 QQTLIELEDQARAAGRGKWSPTASAADKVRNIKWSHENPAHLVDIYGGNPVKAIIEHVRD 206

Q LIELEDQA++A RG+WS + DKVR IKW+ ENPAHLV+ YGG PVKAIIEHVRD

Sbjct 3990 LQRLIELEDQAKSANRGRWSHVPTV-DKVRYIKWTQENPAHLVEYYGGKPVKAIIEHVRD 3814

Query 207 GSTVRAFLLPDFHYITLMISGIRCPGVKLDADGKPDLSVKVPFADEARYYVETRLLQRDV 266

GSTVRAFLLP+F YITLMISGIRCPGVKLDADGKPDLSVK+PFADEAR++VE+RLLQR+V

Sbjct 3813 GSTVRAFLLPEFQYITLMISGIRCPGVKLDADGKPDLSVKIPFADEARFFVESRLLQREV 3634

Query 267 EIRLESVNNSNFIGTILYPKGNIAESLLREGLAKCVDWSMAVMKTGTDKLRAAERFAKEK 326

EIRLESVNNSNFIGTI++PKGNIAESLLREGLAKCVDWSMAVMK+G DKLRAAER AK+K

Sbjct 3633 EIRLESVNNSNFIGTIIFPKGNIAESLLREGLAKCVDWSMAVMKSGADKLRAAERTAKDK 3454

Query 327 RLRQWQDYQAKTPAFNSKEKDFSGTVVEVFNGDAINVRLSNGQVKKVFFSSIRPPRDQRA 386

RLR WQDYQ+K P N+KEKDF+GTVVEVFNGDAI+VRLSNG VKK+FFSSIRPPRD R+

Sbjct 3453 RLRLWQDYQSKAPTVNAKEKDFTGTVVEVFNGDAISVRLSNGLVKKIFFSSIRPPRDTRS 3274

Query 387 VVGTDGEEIVKAPPRGKNYRPLYEIPHMFDAREFLRKKLINKKVQCNLDYISPPRENFPE 446

VG DGE ++ PPRGKNYRPLYEIP MF+AREFLRKKLINKKVQCNLDYISP R+NFPE

Sbjct 3273 GVGADGEVVL--PPRGKNYRPLYEIPFMFEAREFLRKKLINKKVQCNLDYISPARDNFPE 3100

Query 447 KYCYTVSIGGQNVAEAMVAKGLATCVRYRQDDDQRSSAYDQLIAAEQQAIKGLKGLHAKK 506

KYCYTV +GGQNVAEAMVAKGLA CVR+RQDDDQRSS YDQL+AAE QAIKG KG+ KK

Sbjct 3099 KYCYTVLVGGQNVAEAMVAKGLANCVRHRQDDDQRSSVYDQLLAAESQAIKGQKGMFGKK 2920

Query 507 DNATLRVNDLTVDHSRIKVQYLPSWQRALRTEAIVEFVASGSRLRIFVPKDSCLVTFLLA 566

DN LR+NDLTVDHSRIKVQYLPSWQRALRTE IVEFVASGSRLR++VPKDSCLVTFLLA

Sbjct 2919 DNVPLRINDLTVDHSRIKVQYLPSWQRALRTEGIVEFVASGSRLRLYVPKDSCLVTFLLA 2740

Query 567 GISCPRSSRPALNGVPAQEGEPFGDEALTFTRERVLQRDVSVHIDTTDKAGSSVIGWLWT 626

GISCPRSSRPALNG PAQEGEP+G+EAL FTR+RVLQRDVSVHIDTTDKAGSSVIGWLWT

Sbjct 2739 GISCPRSSRPALNGSPAQEGEPYGEEALAFTRDRVLQRDVSVHIDTTDKAGSSVIGWLWT 2560

Query 627 DSGANLSVALVEEGLAEVHFSAEKSEYYRQLKIAEDRAKAAKKNIWTNYveevpkektvt 686

D+ NLSVALVEEGLAEVHFSAEKSEYYRQLK AEDRAKAAKKNIW NYVE+V +EK V

Sbjct 2559 DNNVNLSVALVEEGLAEVHFSAEKSEYYRQLKNAEDRAKAAKKNIWANYVEQVVEEKPVV 2380

Query 687 eeekedkvv-aerkvNYENVIVTEITETLTFFAQSVESGSKLESLMSKLHADFQSNPPIA 745

EE++D+ ERKVNYE+VIVTEIT LTFFAQ+VE+G+KLE++M+KLHADFQ NPPI

Sbjct 2379 VEEEKDEKAPVERKVNYEDVIVTEITADLTFFAQAVENGAKLEAMMAKLHADFQVNPPIV 2200

Query 746 GSYTPKRGDLVAAQFTLDNQWYRAKVERVQGSNATVLYIDYGNKETLPTNRLAALPPAFS 805

G+YT KRGD+ AAQF+ DNQWYRAKVERVQG+NATVLYIDYGNKET+PTNRLAALP AF+

Sbjct 2199 GAYTTKRGDVCAAQFSADNQWYRAKVERVQGNNATVLYIDYGNKETVPTNRLAALPSAFT 2020

Query 806 SEKPYATEYALALVALPTDNEDKEEALRAFSEDVLNHKVQLNVELKVTGSPNLATLRDPT 865

S++PYATEYALALV LP+DNEDKEEALR F+EDVLN V+LNVELK P LAT+ DP

Sbjct 2019 SDRPYATEYALALVQLPSDNEDKEEALRLFAEDVLNRTVKLNVELKPATGPALATVHDPA 1840

Query 866 TKVDFGKQLVAEGLVLAEQRGERKLKELVDQYKAAQEAARVAHLAIWKYGDITQDDAPEF 925

T D GKQLVA+G VLAE+R ERKLK+LV+QY+AAQ+AA AHL IWKYGDITQDDAPEF

Sbjct 1839 TNADIGKQLVADGYVLAEKRRERKLKDLVEQYRAAQQAALTAHLGIWKYGDITQDDAPEF 1660

**Conserved domains**

**protein containing domains SNc, and TUDOR**


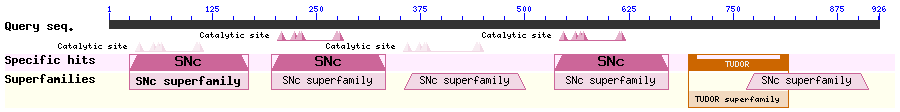


**Vasa intronic**

**>TRINITY_DN23682_c0_g1_i2 len=2645**

GTTCTATTAGTCCTGAATAAAAGAATTACATAATTTAATTTTATCAAATAAGTTTTTATT

TAAACGAGTTCTTGAACTTTCATGGGTCAAAATACATTTCAAAAATTTTCTCATAATATG

TATTTCCATACGAGTTCTAATCGTAAGTAGTTTTCACTTGGGTGATTTTTCTTTTTTTTA

GTTACAGAGTAAATACAATTAAAACCGGTGTGTTTCAAATCTAAAATTGTGTACTATGCT

CCTGCTACATAGAAATAATGGTATTTTGTGTTGTAGTTTTTCAACTTTTTGGTTGCACAG

AAACGCAATTACACATTGTTCAACATGACATACCTCTACCCTTTTTGTTAATTAATTTGG

CTTGTTTTTCTTTAATAGTAGTGTGAACTTTTATTTGCCTTTAAATTATTTTGTTAATAC

AATTAAGAAAGTTATAATAAAATGATAATTGCTTTGCATGAGGGTGATGATGATGATGGT

TTTATTTGCTTCACTTCATGGATCTCATTCATGTGATGACATCAAATAATTTTTGTATCT

TTTTTCTAACCGTTTAATACGAACTGCTTAACGGAGTCCTTGTATTGTTAGTTTAATCAT

ATTATTTAAATTTTAAGTGCTAAAATTTTGTAAAACTCGAAATAGTTTTGAAAATAATGG

AAAGTGACTGAACTCTCACTTTCGATATCATTTTGTAATAAACAAAATTTTAAATTTAAT

CAAACAACAAAAATATACACCTTACTTTAGCAGTGGAACCTACTAGACTTTATATATACG

ACAAGTGCTGTTGCATTGGATATGCTAGTGTGGCTCTTGTAGTTATTGGTTAAACGCTTT

GACTTGAAATCACTTGCTTCTTATTTTCCTTGTTCTCACGCTTTGACGCTGTAATTGGGC

GGTTTTTAATCTGTATTTTACTGTTATAGGATAATATATATTTTAATGCATCAATTCAAT

ATAATGGTATTGAAAACAGAAGTAGTATATCTTGTGATAATTCATTAAATTGGATTTTAA

TATTTTTCAGCAGTATCTGTTACTTTTCTTTACCATATGGTCTTTAAATTTAGTGAAGCT

TCAAACTTATCTTAATTCTCGATTCCTTTTATTATTTGCTTCTGTATTTCCATTTGTTTA

AAAACGTTCTTTTTTGTTTTTGATATTGCGTCTGGTTCAAATGTGCAGAAATTTTAAGCC

AATGTAGGGAATTGACGTTCATCGTTGACTTTTGGTGCAACGTTTTGTTTATAACCGAAA

TTGCGTTTTCCGCCTCGGCCGCCTGTATTAACGCCGCGTCCACCGACTACTGGCCGTTCA

GTAACGCTATTATTATAGTTGGTGTTTGTGTTGGGAACGGCACCTCCTGGACGAGGACCA

CGTGCACGTCCTCCACGGCCACCAAAACCGCCACCGCGACGTCCGTCATTGAAATTGAAT

TGTATGTCAAGTACGCGTTGTTGGCGTCCAACTCTTTGTGGGTACATGGCAGGATCGTAC

TCAAGTTCCTCTTCGCTTTCATTGTCCTTTTTCTTATTGTTGTTTAAAACTATCATTTTC

TTCCATTGAGTAGTGTCTTCACCTTCTCCTGCTTTACGGATATTGAATGTTGGCTTGACT

CTTTGTTGTTTTTGCGCTTTCCATTCATCCAGCGTTAATTCTTTGGTTTCTTCTTCCACC

GGCGTCTGATCAACCTGTTCATTACCAGATTCTTCAGCCTTATCAGTGACATTGCCTGCT

TCAACATCGCTTTTATTAACATCATCGATAGCCTCCTTAACTGATCCCCAGTTATGTGCC

CCGGCGCCATCTCGTTTGTCAATCGCTTTCACACCAGTTTTATCAGAACCCGATTGGCGG

TCGAATTCACGTTTTCCGCCTCGATTACCACCTTCGAAATTGCGATTACGGGGTGGACCG

CGATTTTCTCGGTCCCGAAATTGACGTGGTTGCTGTTGCGATGATCCACCTTCAATTTGC

GCACCAAAATTACCAGCAAATCCATTTTCTCTAGCATTGCGTCGATTATTGCGTTGTTCA

CGAGTTTCGCCGTTTTGTTGTCTAAAATTTAAGTTCCTTGTGCCCTGTTGCTGTTGTCCA

CCTTGACCGCCATCACTAGCGGGACCAGTGCGTTTGCGATTTGCATTGGCAGCAGACCCA

CCTTGCTTGTTAGTTCTATCTACGGCAGTACCAGCCACCGATTTCTTATTCAATTCATTT

TTGTTCAGGGCCGATGGCTTATTCTCTTTCTCGGCTTGGTTGGGTTTCTTGTTTGATTGT

GCCCTTGTAGCACCGTCGACATTTTGTTTATTCGAAGTCTTTGCGGAGGGTGCGCTACCC

CCACTGCCACCAGCAGTCTTAACCTGTTTTTTCGACTTGCTCGCAACATTATCTAATGGA

TCACTAATATCATCGTCCATGAACAAAAGTTCATAACGATTATTTCCAGAGCCATCCATA

ATTGGCGTTTTTGTGAGTTATTTGTATTATTGTTTCTTACTTTTCTTCGGCCAATTCCTT

ATAAGTTTAAACTAACCTTAAATTCTGCTTTCTGCTGCACGATAGTCAAGACTAAGAACA

CATGCCCATCATTGAAAAAGCATGGTATACATATAAGAAGAAGAAGAGTGATGAAACTTT

TGACA

**tBLASTn(First hit)**

Score = 233 bits (595), Expect = 1e-066, Method: Compositional matrix adjust.

Identities = 184/376 (49%), Positives = 223/376 (59%), Gaps = 35/376 (9%)

Frame = -1

Query 1 MDSAGKNRYELLFMDDDVSDPLDNLvaptaaaaaaaagkkkqpsaaaaaatkttankvan 60

MD +G NRYELLFMDDD+SDPLDN+ + + A G +A + +

Sbjct 2459 MDGSGNNRYELLFMDDDISDPLDNVASKSKKQVKTAGGSGGSAPSAKTSNKQNVDGATRA 2280

Query 61 snnkanagsnIGGPNAKKPNQAEKENKPNNALNKTDGKKFTPSADNKQQQFNNNASSNYK 120

+N KKPNQAEKENKP+ ALNK + NK+ K

Sbjct 2279 QSN-------------KKPNQAEKENKPS-ALNKNE--------LNKKSVAGTAVDRTNK 2166

Query 121 QQGAPRQGGGANRTREFgsgqgqgqgqggqqqRSVNFRQQNGnaetreqrnnrrnvrenV 180

Q G+ ANR R + G GQ Q R++NFRQQNG +

Sbjct 2165 QGGS---AANANRKRTGPASDGGQGGQQQQGTRNLNFRQQNGETREQRNNRRNARENGFA 1995

Query 181 GAPDgqqsrpyrgpgggp-gaggdrpqrqNRNYDGQNR--KREFDRQSGSDRTGVKSIDK 237

G Q +R +NRN++G NR KREFDRQSGSD+TGVK+IDK

Sbjct 1994 GNFGAQIEGGSSQQQPRQFRDRENRGPPRNRNFEGGNRGGKREFDRQSGSDKTGVKAIDK 1815

Query 238 RDGAGSHNWGSVKEAIDDVNKNESET-NVTNAEGGAKADESGtepqneqataeeeakelt 296

RDGAG+HNWGSVKEAIDDVNK++ E NVT+ KA+ESG E ++ EE +

Sbjct 1814 RDGAGAHNWGSVKEAIDDVNKSDVEAGNVTD-----KAEESGNEQVDQTPVEEETKELTL 1650

Query 297 ldeWKAQQGQRIKPTFNIRKAGEGEDTTQWKKMVVLTsnkkkendseeeleYDPALYPQR 356

+ WKAQ+ QR+KPTFNIRKAGEGEDTTQWKKM+VL +NKKK+N+SEEELEYDPA+YPQR

Sbjct 1649 DE-WKAQKQQRVKPTFNIRKAGEGEDTTQWKKMIVLNNNKKKDNESEEELEYDPAMYPQR 1473

Query 357 VGRQQRVLDIQFNFND 372

VGRQQRVLDIQFNFND

Sbjct 1472 VGRQQRVLDIQFNFND 1425

**Conserved domains**

**Rho and HABP4_PAI-RBP1 domain-containing protein**


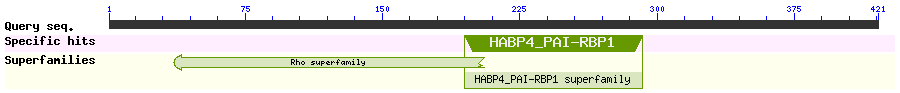


**FXMR1**

**>TRINITY_DN33674_c0_g2_i3 len=2535** GCTGTGGCTGTTGTTGTTGTTGTTGCTGCTGTTGCTGCGGTGGTAACTGCTGCTGCTGCT

GCTGTTGTTGTAATTGCTGCTGCTGCTGCTGCTGTGGTTTCTCATTGTTACCTTTAATTG

CCACATTACTGCCATCTTTGCTGTTCACGCTCTGCTGTTTCGATCGCTCCGACGAGCTGC

CGGCACCACCACCGCCACCCCCACTGTTGTTTTTGTTGTTTTCTACATTTTGTGGTTGTT

TGATTTGTTGGGATTTGTTATTGGTAACCCCACCATTCGTATTATGAGCAGCGTTGCTGT

TCTTTTGCTTTCTGCGCCTTCTTGAACTGCCTTCGTATGACGAGTTGCTTTCAGCTCTCT

CGACACTACTCAATTCACGTGTGTCCTCTCGCATATCGTCGTTATGATTGTGATAGTGGT

CTCGGCCGAGATCCCTGCCGTTTTGCTCTCTGCGTCCATTTTGCTTACCCCCACCACCTC

GTCGATCGTTGCCGCCACGATAACCGCCGCCACCACCACCGCCGCTGCGGTCATTGTAAC

GATGATCACCGCGGGAATTGTAGTCATCATCGTCCCGACGACCTTGATGATAACGCTGAT

TGCCCCCATTACCGCCGCCTCGTCCTCTACCACGCCCGCGCGGTCCACCACGACCCGAAC

GCACCGACTCGATATCACTACTGTAACCTCGTTCCGATCGCCGTGTAACAGGAAAATTCT

GCATAGAACCCATTGTGGACTCTTGTATTGCGCGCAGTTGTTGATCGATTTCCAATTTTT

CCTGACGCAGCAGCTCCACTTCCTTCAAGTGCCCTAAGTGATATTCCAGCAACACTTTGG

CATTCGAAATACTTTCAACGGTACCAATGAACACAAATGGTACATGACCATGCTCTCTGG

GTATGTTTTGATCCTGTTCGTCATCGCCTGCGATCTTAATTCGAAAGACTCCGCTCTTGT

CTACGATTTCCTGTATAATTCGACCGTTCTTGCCAATGACCTTACCCACCAACTCTCTAG

GAACCTGAAAGAATTCCTCGGCATACTCGAGCATGGCGCGTGCTCGCTGCACAGAATCTT

CAGATTCGCCGGTAATTTTGAAGGTGCACGATTTCTCCTCTAACTCAATATTTGTTACAC

CCTCTAATAGGCGAGCTGCTTGTATATTGGCACCGTGTGAACCTATGGCCAAGCCCATCA

AATCTTCGCGCACTTGGAATTCATCGGTATAGCCACGGTTAAGCAGTTTAGTCGACTCCA

GCTGGCGCGCAGCTTCCTCGGTTCGTTTCAGCAGCATAACCTTCTGTGCTAAATTGCGAA

AATGCATATCCTTCAACATGCTGGCTCGTTTCTGAGTGTATTCCCATTTCGAAATCACAA

TCAGACTATCCAAGTCACGATTGTATACGCACACTCCGGCACCTATTGTACGCTGAAATT

CCTTATGAATACCATCCTTTTGCGCTTCCTGTCGCAATTCCTCGGGTACGGGTATTGAGA

ACTGATAGAATGACTTGGACGTCAATGGCGGATTTTGATTTTTAAGGCGTAAACGCTTAA

GTTCGCAGATTTCGGTGTACGGTGTTTCGAAGCCAATATACGCAACAGCGCAGATCTCAG

CCTTTATCATTTTTATGGAAGCAATCCACCAGCCACAGGACTCGCGCTCCGTCGAACGGG

TAAACACTTCCACTTCCATGCCTTCCTCAAAGGTGGGCGGATTCTCGTTCTCTTCTGGCG

GGAAGCGCACATTTGAGAAAGGATATTTTTGTACCTCGGGACAGCCATCAACTTCTACAA

ACACGCCATCGTCGCACACTGCAGTCACCATACCCTTATAATAGGCGCCATTGTCGAGCC

TCACTTCGACCATTAAATCGTCCATTTTGCTACCGTATTCCTTGCCTTTCAAGTTTTAAT

GTAATGCAATTTTGTGAATTGCGACCAAAAATTAAATTGATGAATGAAGTTTCAAGGATG

TACGTTATGTGAATGATGTACGAGATGAACAGTCCAAATTAGGCAAATAGAAGTTTTAAG

CGTAGCTCCTTCCCTGATTATATCCTCCTACTATTGTGGTGGAGCGCTATTAATTTGCGC

ACCTCCTCCTGGCTTGGCTTATTTCAACTTGCTTCAGTCTTTCGATTTTCTGCTTTTATA

CTCTTCTTCTCTTCCACTTAATTATAAACACTCAGCTCCTTGTTTTCCGTATAGTTTTAT

CCTTCTTCCTGTAATTATTTTCAATCTTTTCGTTGCTTGCCGCTCCTTTACCGTTTTCCG

ACGTTAATTAATCAATTTCTTCTATTTTTACTATTTTTTGTTTTTAATTTATTGAATTTT

ACTGCAAGTTCATAAATTTTCGTTAGATCTTTTCTATACGTCGAGCGACGACCAACGGCG

AATGAACAGACAAAGGGGAGACGACAAAAAAATGTGCCTTTTTAATCCGCGGCGAATAGA

TGCACTATGAAACCCTGTGACAGCGAACTCAAGAAAGCAGTAGAACATGCAATGAATTTT

GCTAATCTCTTTTCA

**tBLASTn(First hit)**

Score = 750 bits (1936), Expect = 0.0, Method: Compositional matrix adjust.

Identities = 400/541 (74%), Positives = 444/541 (82%), Gaps = 32/541 (6%)

Frame = -3

Query 1 MEDLLVEVRLDNGAYYKGQVTAVADDGIFVDVDGVPESMKYPFVNVRLPPEETVEVAAPI 60

M+DL+VEVRLDNGAYYKG VTAV DDG+FV+VDG PE KYPF NVR PPEE P

Sbjct 1885 MDDLMVEVRLDNGAYYKGMVTAVCDDGVFVEVDGCPEVQKYPFSNVRFPPEENEN--PPT 1712

Query 61 FEEGMEVEVFTRTNDRETCGWWVGIIKMRKAEIYAVAYIGFETSYTEICELGRLRAKNSN 120

FEEGMEVEVFTR+ +RE+CGWW+ IKM KAEI AVAYIGFET YTEICEL RLR KN N

Sbjct 1711 FEEGMEVEVFTRSTERESCGWWIASIKMIKAEICAVAYIGFETPYTEICELKRLRLKNQN 1532

Query 121 PPITAKTFYQFTLPVPEELREEAQKDGIHKEFQRTIDAGVCNYSRDLDALIVISKFEHTQ 180

PP+T+K+FYQF++PVPEELR+EAQKDGIHKEFQRTI AGVC Y+RDLD+LIVISK+E+TQ

Sbjct 1531 PPLTSKSFYQFSIPVPEELRQEAQKDGIHKEFQRTIGAGVCVYNRDLDSLIVISKWEYTQ 1352

Query 181 KRASMLKDMHFRNLSQKVMLLKRTEEAARQLETTKLMSRGNYVEEFRVRDDLMGLAIGSH 240

KRASMLKDMHFRNL+QKVMLLKRTEEAARQLE+TKL++RG Y +EF+VR+DLMGLAIGSH

Sbjct 1351 KRASMLKDMHFRNLAQKVMLLKRTEEAARQLESTKLLNRG-YTDEFQVREDLMGLAIGSH 1175

Query 241 GSNIQAARTVDGVTNIELEEKSCTFKISGETEESVQRARAMLEYAEEFFQVPRELVGKVI 300

G+NIQAAR ++GVTNIELEEKSCTFKI+GE+E+SVQRARAMLEYAEEFFQVPRELVGKVI

Sbjct 1174 GANIQAARLLEGVTNIELEEKSCTFKITGESEDSVQRARAMLEYAEEFFQVPRELVGKVI 995

Query 301 GKNGRIIQEIVDKSGVFRIKVSAIAGDDEQDQNIPRELAHVPFVFIGTVESIANAKVLLE 360

GKNGRIIQEIVDKSGVFRIK IAGDDEQDQNIPRE HVPFVFIGTVESI+NAKVLLE

Sbjct 994 GKNGRIIQEIVDKSGVFRIK---IAGDDEQDQNIPREHGHVPFVFIGTVESISNAKVLLE 824

Query 361 YHLSHLKEVEQLRQEKMEIDQQLRAIQESSMGSTQSFPVTRRSERGYSSDIESVRSMrgg 420

YHL HLKEVE LRQEK+EIDQQLRAIQES+MGS Q+FPVTRRSERGYSSDIESVRS RGG

Sbjct 823 YHLGHLKEVELLRQEKLEIDQQLRAIQESTMGSMQNFPVTRRSERGYSSDIESVRSGRGG 644

Query 421 gggqrgrvrgrggggpgggngLNQRYHNNRRDEDDYNSRGDHQRDQQRGYNDrgggdntg 480

G+ G GG NQRYH RRD+DDYNSRGDH+ YNDR GG G

Sbjct 643 PRGRGRGRGGGNGG--------NQRYHQGRRDDDDYNSRGDHR------YNDRSGGGGGG 506

Query 481 syrgggggaggpgnnrrgginrrpprnDQQNGRDY---QHHNHTTEEVRETREMSSVERA 537

YRGG GG G +QNGRD +HNH + +TRE+SSVERA

Sbjct 505 GYRGGNDRRGGGGKQNGRR---------EQNGRDLGRDHYHNHNDDMREDTRELSSVERA 353

Query 538 D 538

+

Sbjct 352 E 350

**Conserved domains**

**protein containing domains Agenet, KH-I, and FXMRP1_C_core**


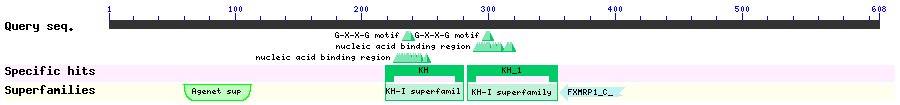


**Rm62 = p68 RNA helicase**

**>TRINITY_DN31247_c0_g1_i3 len=5751**

CACCAAATAGTTTTTTTTTTTTATTTATCAGATATTTTTCTTCAATGCATTGTATACATA

TGTATTGTTGTATTTGATTTCATTTAAAACTCACTTAACTCCTTCATAATGGAATTTTGT

ACATTAATTATTTTTAGTTTATTGTAAATGTTATTTGAAGTCTAGACGTACACATTATCC

AAAATGTGCTGATATTATGGTATTAAAGGGATATACTGTAACTTTGTGCCAGTTGCTTAC

TGGGGGTGCGCTAACTTAACACTAGACATATGACGATATTATTACGGCTACGAATGAATG

CAAATACGCGTTTTGTTTAAAGTAAAGTTAATAAATAAATCATTTTTCCCAAATGTGTCC

AATATCACAAATTACATGTGTAAAGCACATAACCCGCGAGAAATAATTGGAATTTGCCTT

CGCAGAAATACATACAGCAACTGATTAATTTGTTTCAATTTAATCTCGAAAATATTGGCA

TCAGTAGACACTTTGTTACAATTCGCTTTTGATATTGGGATGATGTGCTTTTATTTGCAA

AGTACTCTAAATATTTTTTTTGTTTGAAAAGAAATGCAAATTCTTTAAATATTTGAACTT

AAATTGGTTTGGTTCTTCATTTTAATTATGTATTAGCTATGCTTTATAGAAGTTTCTGTT

AAAAACCAGAAACTTAAAAATCAAATTCACTTCAAAATATTATTGAAATGAGTTTGTTTT

GTTTAATTTTAGTTATCTTTTAAGTAAAATCGTAATATTATATTTAAATGAAAAAATAAT

ATTAATAATAGCCTATGAATTAGTATAATTTAATTTAATCAAAACGCGTATGCTGTCTGC

CTCCTCCACTACTACTGTTGGCAGCATTACGATTGCCATAACCATTTTCGCCGCTGTTGG

TATTGCTGTAGCCGTTACTGCGACCACCGCCATAACCGCCGCCGCTGTTACCGCCATAAC

CGCCACCGTAACCGCGTCCACCATTGCCACCGCCGCCTATCGCACCCTTTCTGAAGGTGC

CATTGAAGCGAGAGTTGCCGCCGCCACCACCGTAGCGGGAACGGCCACCATCGAAACCTC

GGTTACTACGCGCCATACCCTCTAGTGCAGGATTGATTTCCTGATTAGCCTCTCGTAAAA

CATCAACCAGCGCTCTCGACTGTTTGGCATTGTTTTTGGTAAAGAATGCATATGAAGTGC

CCTTTGTATTGGAACGTCCAGTGCGTCCTATTCTGTGAATGTAGTCCTCCGACGATTGTG

GGTAATCGAAATTAATCACATATTTGATGCCATCAACGTCTGTAGCATTTGAAGTTAAAA

TATTTTTAAGAGGGAAAGGGGAAAATAAAAAATTAATATAATAATATATTTACAGCAATA

ATTAGGGTAAGTGGCAAATAAGGTATCGTTGGTAATATAATTAAATGGACTTTCGACTTT

TGCAACGTAGAGCACTGTTTTTTTAGTACATTTTTAATTCACTTGTAAAAATTTAAAGTC

TGTTCAGAACACTTTTGGTATGCTGGCTTTGATCTTTTTTTTTCATTTTTCTTTGGTTTT

TTTTTTTCATACTACTTTTGAGCGTTAAAGTAAATTTAAACAAAAAGAGATAAAGTGGAC

TTAGTTAAATATGTACTTTACCTTCAAATAGTCGATTTATATCCACGCTCTTCTCTCGTC

ACGTTCAGCGAAACGAGGAAAAGAGCATGTCGAAAAACTTTAAATATATGTAGGGTATTT

TTCCCTCTAATCTGTCTCTCCCTCTCCCTCACTCAATCAGCATTGACTTGACAGAAGATG

GACGAACGATGCAGCTCGATTCCGCACGACTTTTGAATTAATGCGTCTCGATCGGAGTTC

AGCGCACATCCAACTAATTTTATGGTTCGAGTTTGTGCGCCGCCCGCCGACGTCGTGCAT

CCATCTACCAATGTTCAGGTAAACCACTACCGCAGGCAAGTTGATAAAGCATGTTTTCAT

CTTTTCAGTTCAACGGCCAGTAAATGCCCGCCGCTTTTCTCCAAATAACTGTGTTTATTT

TTACATTTCAAGCTTTTGATATGCATATCCTCCAAATTATAGTATTAATAAAATAATCGA

TGTTGCAACTGTGTCCTTTGTAGGCGGCTTTGCGATGCATATGTGGCACTTTTTCTAAGA

GAACGTTTTGCCTTGCACGAACTTTTCGCATTTTATGGAATGTGAATGTGTTGCACCAGT

ATCCGCAGACGACGACGCTTATTTCTGGACGTGCAGCAAATGGTGAACTGGTGTTCTGTG

ATGTCCATGGTAGAGTTAGAACAACAGCAACAGCTTCTCAACATTTGTTTAGTGTCCAAA

AGAAGGTCGCCACCAACACGTTCGATGCAACCGTTTACGTCTATCGAATGAGTAAGGCAA

AACATTTTTTACACCGTGCCACGTTACACCAACCAACCTAGCTTCAAATCCGCGGATACG

CGATGAACAGGATCACAGCGTTTGCAAACAAATAAAAATTAAGTTCACTTATAATTCAAT

TCCAACACTACAACAACTATTAAAATATTGCAATAACCGTAGTGACCCTGTTCCTTGCAA

ATCAATTATCTTTTTTACGATGACGTTACGATCCAGCCAAAGAACACATGCGCTGTTGCG

ATTCTAGGACGGACATGCATTTAGTTTTATGGAGTTTCTCTAAGTGCCTGCGAGTGATTT

AAACGCTGTTCCCTTATGGAGTACATGCCAGAGAGATACACAAAATAACTAACTTAATCT

CTCCTTTATGTGCTCCACAGTAAGTCATTTGAGTAACGCTGTCTGTACGGTTAGAAATCC

TATTTCTTTTTTTATAGTTTTCTCTCTTTGGATTCGATATTGTTTAGTTTTACAAATACT

ATCACTTGAATAAAAGATAGCAGAGGCCACATTTGCTGCATGATGATAGGGATATTGTGT

ACCGCAGAACTCTATGTGCACATTGCGAATTTGGGAAGAAGTAGAATTTACTTTTGTATT

TGGAAGAGCAAATATTTTACACGACGCGACATATATCTCGCGTTTTGTAAATGAATGTTG

CCGCTTTTTGTTTTCTTGTTGCTTATCAATTGAGTATCAGCTAGAGTACGCTGGAACGCT

TTTTCGTAAAGGTTGCACGTATCAGGCAAGATTATCTATCGGCTGTTGATTAATATATCT

TCTCCTCCAATGTGCAGTGACGTTATGAGCAAACGTATGGCAGCAGATGGTTCCACATCC

GTTTTACCTTGGAATGTTTAGACAAAGTACTGCCACCACACTCGGTTTTATTGGAAAAGA

TCCTTTCAAAGAGCTTTTCACCGGCTTCTTTTTATGATAAAATTTCAGAGTTCACTTATA

AACGCAATTGTATCGAATTGCGTAGTTTCAGAAGTTGTTCATACTATCCCCACCATACTA

CAATAATCGGCCTCTGTAAACGAAAAGAATCAGGAGAATAGTTTTGGGGGGAAAAGAAGG

TAAAATTCCAAGTTGATTAATATGTCTTTTAACAGATATGATGACTTATTTGTCGCTAAA

GCTAAAGTGAATTTTTGGCAATTGATTCGAGCAGTTTTAAGGTCTAACATATAGAGTATT

TTGATGTGCCTACGGGCTTAAAGGTGCCATGGCGTAGTTGCTTACAATCGTCAAATGCTG

GGAGTATGTGATTGCAAAATGATTGCAGATCTGGTGAATATTATCAATCCTTATGTTATG

AAGTCAGCTAACACACTTTGCATTCACCATACCTCAGTTCATTTGAAAATATAATCAAAC

CAGTACAATAATATATGTGAATGATAATGGTGTAATGTTCTCAATAACGAGCGTAGAGAG

TGAAACTTACCCAAACCGCGTGCAGCCACGTCGGTTGCTACCAAAATGTTTGACTTGCCC

GAACGGAATTCGCGCAAAACGTAGTCACGTTCCGACTGTGACTTGTCACCGTGAATGGCG

CCACAACGAACACCAAAACTGCGAATGAAACGCACCAGATTGTCAACACGGCGTTTAGTC

TCCACAAATATGATGATCTTGCCGGGGTTTTCGCTGGTATCGTAAATTTCGGAAAGCAAT

GATTTCAGCTTGTCTTCCTTGTCATGTTCTTCACAAACATCGATCACTTGACGGATGTTA

TGGTTCGCCGACAACTCCAAAGAGCCAATATTGATTTGTATGTAGTTGCCAAGGAAATCT

TCTGCCAATTGCTTAACCTCTTTCGGCCAAGTGGCGGACCACATGAGAGTTTGGCGATCA

GGGCGAATCTGTGACAATATTTTGCGGATCTGTGGCTCAAAACCCATGTCTAACATACGG

TCAGCTTCATCTAATACTAAATATGTACAACGCTTCAAGTTTGTAGCATTCATTGAGAGA

AAATCAATTAGACGACCGGGTGTGGCAATGACGATTTCGCATCCACGCTGCAAGTCGCGC

ATTTGGCCTCCTTTTGGAGCACCACCGAATACACAAGTATTTCGAACATAAGAAGATGAC

CCGAATTCTGTCGCAACTTGTTGAATCTGCTGTGCCAATTCGCGCGTAGGTGCCAATACT

AATGCAATTGGTCCATCACCACGCTCCAATGGTTTTTGGTTGTTAATGTGCACAATTGCT

GGTAAGATATAACCGAGTGTTTTTCCTGATCCAGTCTGAGCAATACCAACAAAATTAGAA

CCGCTCATAGCGATTGGCCAACCTTGAGCTTGGATAGCTGTAGGTGCCTTGTATCCTTGT

CGGCGTACCTCCTTCATAACATAATCCGGAAAATACGCTTCACTGAAGTCCTGAATTGGG

TTTGGTGCATTTCCACGAATGGTAATCTCATGTTCATCACGGAAGCGCTGTACTTCATAG

GGCGAACGATTGGCTACAATTGGGTGTTCCTGATAGAAATTCTTCTTGAAGGGTGCCAAA

TTTGAGAAATCGACAGAGCCAAGTGTGATGTCCTGTGTGCCACCGCGGCTATTGCCACCA

CCGTATCCACCAGCTCCGCCGCCGAAGCCACTGCCGCCACCAAAACTACCGCCACGGCCG

CCACCACGGTTCTTATCAATACGTCCATTGCGTACGCCATGGAAGTCACCACCACCAACA

CCACCACCGCCGCCGCCATGACGCATGCCACCAGCACTACCGCCATGTCTAGCCTGACGG

TCATCACCGCCACGTCCACCACGAGTACCAAAATTCCTATCGTGAGGTGCCATTTCGTCT

GGATCTATATATATTGATGTTTTTGTAAATGAGTCCTGATGTCTTTTTGTTGCCGTAAAT

TTTTGCTTTTCCACAGGCGTCGCTTCTGCTTCCAACAAATTTTCGCTTGTTTTGTGAAAA

TGCCTATAGAAGCTATCAGAGCTACTTGTTATTCCACCGAGATTACTTGATTTCTTAAAC

TCTGCTTGAGCGATAGTTTCTTTGCCTGTAAATAGCTGGTACTTGGAAAAAGCAAAATTC

ACCGGTCCTCTGTTGGTTGAAGAAAAAAGAAAATGGCGTCGTGATATAGTACTCGTGGCT

GGGTTAATAACGGCCGATGGAAAAATCAATAAATTTTTTACACACGCCACTGCAGCCACA

TGTGACGGTGTTCGGTTTTCACACGAGACTTTCGCTAAACTTTGAACCAATATTTTAAAC

ATTTTTACTTGTATAGTAACAGTTAGCACTTTTAATAAATTTTTCACTATCACATTTATT

GGTTACAAAGCAGCAAAACTCGTGCAAATGAACCGATTATAAAATGCGTTC

**tBLASTn(First hit)**

Score = 716 bits (1847), Expect = 0.0, Method: Compositional matrix adjust.

Identities = 336/369 (91%), Positives = 356/369 (96%), Gaps = 0/369 (0%)

Frame = -2

Query 226 SQDLPMRPVDFSNLAPFKKNFYQEHPNVANRSPYEVQRYREEQEITVRGQVPNPIQDFSE 285

+QD+ + VDFSNLAPFKKNFYQEHP VANRSPYEVQR+R+E EIT+RG PNPIQDFSE

Sbjct 4958 TQDITLGSVDFSNLAPFKKNFYQEHPIVANRSPYEVQRFRDEHEITIRGNAPNPIQDFSE 4779

Query 286 VHLPDYVMKEIRRQGYKAPTAIQAQGWPIAMSGSNFVGIAKTGSGKTLGYILPAIVHINN 345

+ PDYVMKE+RRQGYKAPTAIQAQGWPIAMSGSNFVGIA+TGSGKTLGYILPAIVHINN

Sbjct 4778 AYFPDYVMKEVRRQGYKAPTAIQAQGWPIAMSGSNFVGIAQTGSGKTLGYILPAIVHINN 4599

Query 346 QQPLQRGDGPIALVLAPTRELAQQIQQVATEFGSSSYVRNTCVFGGAPKGGQMRDLQRGC 405

Q+PL+RGDGPIALVLAPTRELAQQIQQVATEFGSSSYVRNTCVFGGAPKGGQMRDLQRGC

Sbjct 4598 QKPLERGDGPIALVLAPTRELAQQIQQVATEFGSSSYVRNTCVFGGAPKGGQMRDLQRGC 4419

Query 406 EIVIATPGRLIDFLSAGSTNLKRCTYLVLDEADRMLDMGFEPQIRKIVSQIRPDRQTLMW 465

EIVIATPGRLIDFLS +TNLKRCTYLVLDEADRMLDMGFEPQIRKI+SQIRPDRQTLMW

Sbjct 4418 EIVIATPGRLIDFLSMNATNLKRCTYLVLDEADRMLDMGFEPQIRKILSQIRPDRQTLMW 4239

Query 466 SATWPKEVKQLAEDFLGNYIQINIGSLELSANHNIRQVVDVCDEFSKEEKLKTLLSDIYD 525

SATWPKEVKQLAEDFLGNYIQINIGSLELSANHNIRQV+DVC+E KE+KLK+LLS+IYD

Sbjct 4238 SATWPKEVKQLAEDFLGNYIQINIGSLELSANHNIRQVIDVCEEHDKEDKLKSLLSEIYD 4059

Query 526 TSESPGKIIIFVETKRRVDNLVRFIRSFGVRCGAIHGDKSQSERDFVLREFRSGKSNILV 585

TSE+PGKIIIFVETKRRVDNLVRFIRSFGVRCGAIHGDKSQSERD+VLREFRSGKSNILV

Sbjct 4058 TSENPGKIIIFVETKRRVDNLVRFIRSFGVRCGAIHGDKSQSERDYVLREFRSGKSNILV 3879

Query 586 ATDVAARGL 594

ATDVAARGL

Sbjct 3878 ATDVAARGL 3852

**Conserved domains**


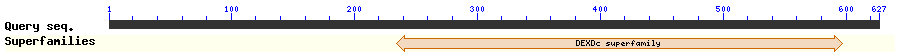


**Translin**

**>TRINITY_DN31480_c3_g3_i11 len=2472**

ATTTGAATTCATTTTACTTTTAATTTAATCGTCAAAAATACTCATTATCCGACGTGCTAA

ACCTCAAGTGGTATTTTATTACCCCTTATATTATTTACTACTTCTCCTTGCATTTTACAT

TTTATTCTGTGGTCAAATTGAAACATAATTTCCAAATTAACTTCGCGGGTATTCGATTTG

GAAATAGTAATTTTTCTATAGTTCTCCCGTTTGTTTCCTCACACAGTTTCATTTAAGGGG

TAAGACCACCCTGACGGCATGACAATTAGATTAGAAATATTTTTTTTAAGAAACTTTTAG

GTGTAGAACTTTTTCACATTTAAGTAGTTAAACGTAAAGTAGTGAACTTCAAAACACATA

TGCAGATTTATTAGAAAAAAAGCAACAATTTAATTGGCGAAAATTAAATTGGCTCTGCAT

GAGACAGTTTTTCCAGGACACTCTTTTGTGGTGGGCGTAAAAGGTACCCCAATTTTTATC

TTTAACAAACATGTGAAGGCGTTTTTAAGTTTGGCAAATTATCTGCAGAGTAACGTAGCG

GTTTTTGAAAACAAACAAAAAAACACTCGCCTTAAAATTAAAAGTTAAGGGGGTCTTCTA

CCGTCACGGTTTGAAAAAATCGATTTTTTTTTTTTTAGATTTTTTAAAAGCTACTAATGT

TAAGAGTATGCTATAAAGAGGGTTTTATGAAAAACATGTTCCTTCATGAGCATTTCGGGG

AGAATACATGCATGCGCGTGGCATTTTCGAGCGTTTCTGTGGCGTGACTTTATCTTTAAA

CGCCTTTTCCCGAAAGTTGACTTTTTTCAACTTTCTGGTCACACATCTACTATAAATATT

TGTCGAATTCATTCCATCTTTTTTCCAGTTATTCAGTGAACATCTCGCTATGTTTTCCCA

GACCACTTTTTTCAATTTTTGATTAGTTTAATTTTGCGGGCCTCTAAAGTGTAAAAAAAA

GAGGAAATTTTCAAACTTTTTTTTTAAAATCACGCATTTTTTACAAATTTCAAAATATTT

AAAATCGCCACTGGGAAAACATAGCCAACGCTACACTTTATGATAAAAAAAAAAATTTTT

TTGATTTCAGATAAGTAAAATGGTCGATCGCGTGCCACAAAGTCGCCTAGGACATTTTTA

CAAGGGCAACCTCGAGGACGGTTCAAGGACACAGGGCCAAAGAATTCGATTTGAAAAATA

TATTTTTAAATAGTGCAAACTTGTGCAAATTAAATAAAAAAAATCTGATTTCATTATCTC

AAGTGGTTGCTTTGTAATTAATTCCCAAAAATAGGCCCGAGATTAAGGCGCGACGGTAGA

AGACCCCCTTAAGTTATATGTTCTATACTGAACTGCTTGCTAAATTTTAATTTTTACAAG

ACATGAAGCATAACATAATTTTCTTAATTATTTGACACTTCCCCGCTGCGCACTTACTTT

GAGCTTGACTCTTCTCCCCCATCTTGTGTTGCATTGTTGCGCAGTCCGCGTATGGTAATA

TCATAAACCACTTCTTCAATTTTTTTCACATCGTATTTCAAGGCATCAAATCTCTTTCGC

AAACCATCATTTTTTAAATTAAGTAGACGAAAGCCAGAATTTAAATTCGCCATGAAGTGA

GAAATGTTAAGAGGCCGGTCATAGTCACCCATAGTTACCGAGTTGGTTGCGAAACGAGAT

AGTTCAGATGCCATTTGTAAAATTCCCATTAAATAATCCTCTATATCCAGGTGAAAGCCA

TGCGCCTGATTAGTTTTCAAGCCCAACATTTCCGCTACAGTTTCACGATTTACTAAAAAA

CCCGCTTCTAAATAAACAACCATTGCAATCAGAAAAACTAGACGTTGTGTTATAAATGTC

CAATGATCCGAGTATCTATAATACTGTCCCATGGGAACTAGTTCAGCTAATTGCTTATAG

ATGTTAGAACAAGCAGATATCTGTTTACGGGCTTCCTTACATGCATTGTCAATATTTGAT

AAGTCAGAATGAATGACTTGAAGTTGAATTGTTGATTCCTTTGCCAATTGCTCAATTTCT

CGCACTTTTATTTTGATATTCTCACGAATTTCCTGTTCATTTTCCATATACTTCTGGTAT

TTGGCAAAAATTTCTAGATCGACAAAATTAGCCATGCTTTAAACAATAACAACTTTAAAC

ACGAAATTACTTTCCCTCGCAAGCACTTTTCTTATTTACGCTTCGGTGCGGCTTTTCGTT

ATGGATGTACAATCGATTCCGATACTTAGAGTTACTGTGCGTTTACACATTACATATTTA

GAAACGTTTTCTTGAGAGGTTCCAGTCACAGCAAGAACAAGAATCATAGAATAACCGATT

GCGCCTTCCGAATTCCCCGTGTCGTAAAAGCCCATGAATAAACAAACAAATTATTTGTTT

ATGAAGTGGAAGAGTAGGCGAAAGTTATGGAAACAACCAAACACAAGAAATTATACGCAC

ACACACACTCAG

**tBLASTn(First hit)**

Score = 372 bits (954), Expect = 2e-122, Method: Compositional matrix adjust.

Identities = 172/231 (74%), Positives = 207/231 (90%), Gaps = 1/231 (0%)

Frame = -2

Query 1 MSNFVNLDIFSNYQKYIDNEQEVRENIRIVVREIEHLSKEAQIKLQIIHSDLSQISAACG 60

M+NFV+L+IF+ YQKY++NEQE+RENI+I VREIE L+KE+ I+LQ+IHSDLS I AC

Sbjct 2135 MANFVDLEIFAKYQKYMENEQEIRENIKIKVREIEQLAKESTIQLQVIHSDLSNIDNACK 1956

Query 61 LARKQVELCAQKYQKLAELVPAGQYYRYSDHWTFITQRLIFIIALVIYLEAGFLVTRETV 120

ARKQ+ C+ Y++LAELVP GQYYRYSDHWTFITQRL+F+IA+V+YLEAGFLV RETV

Sbjct 1955 EARKQISACSNIYKQLAELVPMGQYYRYSDHWTFITQRLVFLIAMVVYLEAGFLVNRETV 1776

Query 121 AEMLGLKISQSEGFHLDVEDYLLGILQLASELSRFATNSVTMGDYERPLNISHFIGDLNT 180

AEMLGLK +Q+ GFHLD+EDYL+GILQ+ASELSRFATNSVTMGDY+RPLNISHF+ +LN+

Sbjct 1775 AEMLGLKTNQAHGFHLDIEDYLMGILQMASELSRFATNSVTMGDYDRPLNISHFMANLNS 1596

Query 181 GFRLLNLKNDGLRKRFDALKYDVKKIEEVVYDVSIRGL-SSKEKDQQEEPA 230

GFRLLNLKNDGLRKRFDALKYDVKKIEEVVYD++IRGL ++ +D EE +

Sbjct 1595 GFRLLNLKNDGLRKRFDALKYDVKKIEEVVYDITIRGLRNNATQDGGEESS 1443

**Conserved domains**

**Translin domain-containing protein**


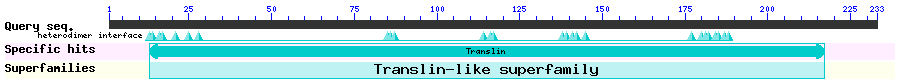


**Translin associate fator X**

**>TRINITY_DN24775_c0_g1_i2 len=1300** CTCTTTCCCTACACGACGCTCTTCCGATCTCGTGACCTCAATGAACTGACAAAGCTGCAT

TCAACGCCATTTACAACTTTTAGATTAGTTTATATCTAAACTAAATTAACACGACATATT

GGCATACAAAAATATATTTTTAAGGTCATTTAATTACTTTTTATTTAATTTTATTATAAA

ACACTCCATATGATAACTATTGTTATATACCTTTTGATGCAATTCTTTACAAAGCCCACC

ATAGACCATTTAAAAGAACCCTTCATCTAGGTCTTCAGCTGCTTTAGTTTCGAAAACAGC

ACCCCATTTCGCTGCTTCCCCACCTCTTACTTTAACATTGTAACAAACGTTTTCAGCTTT

TAATACACTTTGACGCATTGTATAAATTTTACGCGAAAGTTCACGACACCTTTGTACGTT

GAGACTTATATAGCCTGTGAAAAGCTGTTTTAAAACTTTGCATGATTCCAAACATACGTC

CGTTTCCCCACTGCCCAAAGAATTGATGCAGCGCCGCATCAATTCACCGGTTAGGTCTGC

TATTCCCAAAATATACTCGGTGGGATCGATGTTGAATTCAACATTTTGAGGTTCTATTTT

TTCTACAGCTTTATCTGTATCCTGTTGCTTCTGATCTGGTGCAACTACCGATTCCTCAGA

ATCGTCATCTGTTTGACTAACTTTTTCATGTTCTTGTTTATATTTCATTTTTTCTTGCAA

AATTATCCAGTCTGACATGGTATCCAACGGTTTATCTCCCCCGCATTTGAAATACTCCAT

AAAACTGTACGCTTCGATGAATTCCTGTAATCCGGGTGAATATGCCCAACGGTATTGATA

GGGATCATATCCATTTAGCTCCAGGGCTATAGCTTTGAAATTTGTATCAATGATCTTATT

CAGGCGTTCTTCAGCCTCTTCAAGTACTCGGAAACGGTTATTTTTTCGTGCATCTATTGT

ATGTAATAAAAAAATTATACGCTTTGCTTCGATTGTAATATCACGGCTGAGTTTCACAAG

CCTTTCATGGCGGTCATGTTTGTCATCCAGTTCGTTTGCGTATTCCTGGAACGCTTTCAG

TACTGGATTCTGTTCATCTGTGGCCGAAAGACTCTGAACATTTTTTTGTTTGGGTTGGTT

TCGACGGTTGTTATTCCGAAAACGAAAATTTTGAGACATTTTTATTTATTTCAAACTTTA

TCCAGAGTGCAAAATTAACTATTTTTCTATTAGACGCCGACTAGGGATGTGCCGATGCAA

ATTGGTGCTGAGTGATATTTCAGTATCGGTAATTTTTTAA

**tBLASTn(First hit)**

Score = 367 bits (941), Expect = 4e-124, Method: Compositional matrix adjust.

Identities = 181/297 (61%), Positives = 228/297 (77%), Gaps = 13/297 (4%)

Frame = -2

Query 14 PRKRQT-PVAQLDEENPIVQAFRNYSNELTMKHDRHERIVKLSRDITIESKRIIFLLHSI 72

P+++ ++ DE+NP+++AF+ Y+NEL KHDRHER+VKLSRDITIE+KRIIFLLH+I

Sbjct 1134 PKQKNVQSLSATDEQNPVLKAFQEYANELDDKHDRHERLVKLSRDITIEAKRIIFLLHTI 955

Query 73 DSRKQNKEKVLEEARQRLTKLIEVNFKAVALELRDQDVYQFRAAYSPGLQEFIEAYTYME 132

D+RK N+ +VLEEA +RL K+I+ NFKA+ALEL D YQ+R AYSPGLQEFIEAY++ME

Sbjct 954 DARKNNRFRVLEEAEERLNKIIDTNFKAIALELNGYDPYQYRWAYSPGLQEFIEAYSFME 775

Query 133 YLCYEDGEGEKEVKSVSDWQAIQAVMQYVEETSKLKGASTEGEEVEVAA---------LT 183

Y ++ G G+K + ++SDW +Q M+Y +E K+ + EE VA

Sbjct 774 Y--FKCG-GDKPLDTMSDWIILQEKMKYKQEHEKVSQTDDDSEESVVAPDQKQQDTDKAV 604

Query 184 EDEIPKKFQFFVDPTEYILGLSDLTGELMRRCINSLGSGDTDTCLETCKTLQHFYTGYIS 243

E P+ +F +DPTEYILG++DLTGELMRRCINSLGSG+TD CLE+CK L+ +TGYIS

Sbjct 603 EKIEPQNVEFNIDPTEYILGIADLTGELMRRCINSLGSGETDVCLESCKVLKQLFTGYIS 424

Query 244 LNCQRARELWRKITTMRQSVLKAENVCYNVKVRGGEAAKWGATFDQKPAEDVDEGFY 300

LN QR REL RKI TMRQSVLKAENVCYNVKVRGGEAAKWGA F+ K AED+DEGF+

Sbjct 423 LNVQRCRELSRKIYTMRQSVLKAENVCYNVKVRGGEAAKWGAVFETKAAEDLDEGFF 253

**Conserved domains**

**TRAX domain-containing protein**


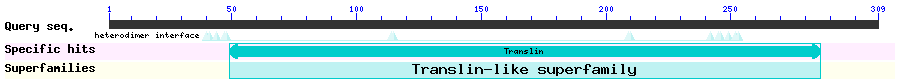


**Armitage**

**>TRINITY_DN31912_c0_g1_i3 len=4570**

ATAGAAATACACTCGGAGGTTTGCCATTATCTTCCGAACGGCGACCGCTATTAGAAAAAA

CTTTTTTTATCATTTGGTGTTTCATGCATGGAGTTCCGAACCTGTGAACTTCCACACAAC

CAACCTATTCGGCTTACGGCGGCCGAAGCTTACACGCCTCTGAAAAATCACCCTTTAATA

CCTAATAAAAACTTGAAATCATATCATATCTATATACGTAAATTTTAAGTTTTTGAAAGA

TTCCGTACTGGTAAAGGTTGAACCTTTGTTTTACAAAGCAACTCCTTGGTATGACACACT

TAAATAAAAACTTATTCGTACAATGTAAATAATGTAAAAAGAAAAACATCGATAATTTTA

AATTACTAACTGAAGTTAAAGTTATAGGGCAGTTGCTGCAATTAACTTTCTAGTGAATTT

CGTTGAAATTTTTTTAGCAATTGCATAGGGTGTTTAATTTCACTTTTTACGATAGGGTTT

TTAATTTCACTTTTTACTTGCAAACATTAAGCCGGTAGCGCCGATTTCGATGTGATTTCA

AATGTATCTTCCTGCTCATCTTGTTCCGGTCTAGAGTGAAAGTCATCTGGCAGATCACAG

CCAAGATACGCTTCATTGTCTACACAATATTTGATGCACGTACGCCAACAGTGATCTAAG

TAAAGCAAGTGAGGATTTCCAAATATAAACATTAGATAGCGTGCCCGTGAAATGGCTACA

TTCATGCGTTTGGAGCTTTGAACAAAGCCTAGTGCGTGACGCAAGTCCCGACTAATTAAT

TTCTGCGAAGAACGCACTGTGGAGATAAGGATAATGTCGCGCTCCTGTCCCTGAAATTCT

TCTACGGAGCCAATTTTCGGCATGGCGATATCCGCTTCGATAAAGAGTGTACGCAAATGT

TTCACTTGTTTCATATAAGGCGTTATTATGCCGATATTTTCCGGTTGAATGCCTTGACGA

TATAACTTTATGGTCATTAAAAACACATTTCTGGCTTCGAGTGGATTGAACCATGAGGGT

GAATCGGCTTCCTGTTTATTTTCGCTGCGGGTGCCGTAGAAGAAGACGCCATGCGATTTT

GGTCGTTTCTCTGATGCGGGTAACAGCGCATCAAGATTTTTCAACATATCAGCCTCACGC

GAATCATTCTCATTTATCATAGCACGCAACTCAGAGTCGTAGAACAACTCACTGTACACG

TTGAGTATGGACGGCAAGGCACGATAATTATAGAGCAACTTTGTTACCAAGCGCGGATCG

AAACCCGACGTGTCGGGGAAACGCATCAGGTCGCGCAGATATGGTGTTCGTCCTAGTATG

CGTTCCAGCAATGATAGTGATAAGCCACGTTCAGAGGCAAATCGGTTAATTACAATTGCT

TGCAGTTGATGTGGATCTCCAGCCAGTATTACTTGACTGCGCTTCTGAGACAGCAAGGAC

ATTGGCACTATAATTTCGGGTTCAGTACATTGACCGGACTCATCGATGAGCAGATGTGTA

AAATGACCAGGTGGAAATCCCATTTGCAGAAAATTACCTAGTGTGGTGCATGTGCTGATA

GTGAGTCGATGACGTCCCAAATACTTCATTTGACAACGTAGTTTCATGCCTGATTCTGTG

ACAATCATCGAGTCGTCACAAGTGCCATCGGCAGCCATATCAACAGTGGCACAATACGGC

ATTAGATGATCTGGTATTAGTTCCTTTTCGATTTGATTTTGAGAAACTAAGCGCACAAAA

TCGCCCGGCTGCAATTGTTTGCATGCTATTAAACGCGTTGTTATCAAATCCGCTGATCCA

TTTGAGGGTGTGCCAATGAGTAAGCGAGAACTAGGAATGAGTTTAACCAGCTGTAATATA

GTTTCGACTAGTGTCATTGTTTTACCGGTGCCTGGAGGACCAAATATTATGTAGGGCATA

TTTTGTGCTTCGCCACGTAAAATATTCCGTATGGCGCGCTTTTGTATGGGATTTAATAGT

GGATTAAACCAGGGACAACGTGTCCCCTGCAGCATGAGATTATTATCGCTGTCTAACTCA

ATTTGTAATTGGGGATTATCACGTGTATAAACACGAGAGGGAAAGAGAAATTGTTCGCCA

ACGTTTTTTATTATACGACTGACAGCGTAGTGTTGTTTACGAAAACAAAAACGGGAATAG

TAAAACTCTAGTCGAAAGTCTTCGCCATTATATTTTGACTGGAATCCTTCGTTAAATTTT

AGTAAAATGCGATTGAAAAGCACTTTGTGGATAACGCCATCATAACTGCGTTTATCGTCT

TCATTGCACCAAGGATTAATGGCACGCACAGTGTCTCCAATAACGAGTGATGGTCGGCGT

TCAGAAAGATTCTCAATAGTGAGTGACAAAAACTCGCCGTCACGTGTGAAATGTGCCCTC

TCACGATCATAGTTGCGAAAATTCACAAAATATTCTACTTCCTCCAGATACACTAATGTT

TGAAATCTTATGGCGTAGTTCTTTATATTTAGTTCCTCTTTCAGGCAGGGGAACATACCC

TCTAAGTTGTCTACCATTTCTTGACGATTGGCTGCCGTCAAGTAAGTATAGCGTAAGCGT

TCGGGTACCTCGTAAAAGGCAATGCGATGTGACAAAAAGCGGCGCTTTGTTGCCAAACCA

ACGCCTGGTATAACTTCACCTTTATTGGACCACACTTGATTGGCGTAATGACGTGAACGC

TGTCGCATAACATGCATACCTGGCGATGTATTTGCAATACTATTAGATGTTTGAGCACGT

GCGGCCTCGGCCTCGGCTTCCGTTTCGCACACGACTATCGAAATAGAACGCTTTACACGG

AACGTTTCAAATTTAATGAGGAAAACTTCGCGGGATTCACCATAGAACTTAGATTCTACT

TCAAAGTGTAGCGTACGACTACCATGACCGGGCAGTTCAAAGTTGGTTTCATCGAGTTCG

GGATCTACAAGTTTAACTTGTGAATCACGTCGGCGCCCTAAAAATTCGATGGAAAGTACT

CGCATTTTCCGTTCTATATTATTTTTGATTATAATTTCAACCTTTTCGGTCTTAAAAGTG

CTAGAAAATACGCAACGTTGATCTTCGGAAACCGTAATTGCATGATTACTTTCGTTCCGT

GCTTTCGGCGTTGAGCGTCTTTCTCCGTTTATTGGTTTCTTAGTTCCGGCGGGCGTCGTT

GGCTGTGGCACTATGCGTTCCAGTAGCGATAATTTAATGCAGCGCCAAATGTACGGATCT

CTTTCACATTCGATCAGATCTGCTATTACAATATCGCCTACATTGAGTTTACATGTATTG

GACACAATATCAAATGTAAAATAGCAATCATCGTTTAATACACCCCAGTCTGAATTTAAA

CGCCGCACTTCACATTTTTCTTGTTTGAGTAAGCGAGCTGGGTGTACGCTAACCTCCTGC

AGTACTTCACCCTGGCGATTAACGAAATGCTCATCACTTTGTACGTTGCAGCAGATAAAA

ACACGATCACCAGCTTGTGGTATGAAATCGATTTCTACTGTATCTAAATTAATGTTCATC

AACTTATGCTCGGTATCGACGACAATAGTTCCCGGCTTCCGCTCCATTATAAGTCCGAGT

ATATTACGTTGATGGGTATTGAAAAATTTCGGTTTTTCATTGCGAAGCTCTGTTATTTGT

TGTTCGATCTTTTCCGGGGTGGGTAAAGATTCATCCCAATATAATTCGGATACTTCCTTG

ATTTGTACCACTTTAATGTGATCACTATGTACTTGTTGAAATGCCAGGTACTTAACAGGA

CAACCGACTTTCAAGTCACTCGCCAAACCGCCAGCAACGTTAATATCAAAAAATATTGAA

TGGTCAATGGTGCCATGGTTACTTTTTAATGTGGTTATTACGCCAAGCCGTTCGAAACAC

GTGCCCTCTTCCTTACTTGTGGTAGTCACTTCGTCATTTTCTGATCCGTTAAGAGACCTC

AATATAGTTTGCATTTTTGAAGTATCGCCATATTCAATCTTTTTATTCTCTTCCTTTTCA

ATTTCTGCATCCAGGTGGCGTTGTTCTTCTTCTAGTTTTTCGGCGTACATCCGCTTCTGC

TTCTCGGCATCCATAGAATCCCATAGATTCTTCAAAAACGATAACATGTTGCTCACAAAT

TTTAAAACTTAACTAGTATTAGACTTAATATTAGTTATTTCAGGATAATGAATTATTTTT

ATAGCGTCTTTCTCTCAACGGATTCATACATCCAGTCTGTCTCACTTATGTAATTCAACT

CCTTGCGCAGGTTCTGCAATCGCTTCTGATAAGTATCCGAAATTCTTAACTTGTAGTCGT

CTTCCGTCAGTACAATTGAATGACAACGTGAAAATATTTTATTCGTTTGGGTCTCCGGTT

TTTTAAGTTCACCGTCGACTGCTGTTTCTGATGTGCTCGGCAATTCCTCAGCTATGCTTC

CGGGCTGGTGCATGATACAAATGATCCTCAAATCAATGTGCTTTCTGTTTTATAAAGGAA

TCCAATACAATTTTTAAACGCGCGTCCGCAAACAAAAAGCCGGATAATAAAGCACCGCAA

CCGCATCAGA

**tBLASTn(First hit)**

Score = 1164 bits (3010), Expect = 0.0, Method: Compositional matrix adjust.

Identities = 593/1189 (50%), Positives = 831/1189 (70%), Gaps = 35/1189 (3%)

Frame = -3

Query 5 VSKFFTNPDRNREDILESLDRENS--FLDQKLMEEK--MDQQLKANPNE-INGVLSNKIA 59

V KF +N +++ +S+D E +KL EE+ +D +++ N+ I ++K+

Sbjct 4148 VLKFVSNMLSFLKNLWDSMDAEKQKRMYAEKLEEEQRHLDAEIEKEENKKIEYGDTSKMQ 3969

Query 60 ELTHGL--SEMD-----VSKESSCTARKGVITSLDGDRGVIDKDVLFETKVAEDIILDLH 112

+ L SE D +E +C R GVIT+L + G ID + F+ VA + DL

Sbjct 3968 TILRSLNGSENDEVTTTSKEEGTCFERLGVITTLKSNHGTIDHSIFFDINVAGGLASDLK 3789

Query 113 VGCVVEYLTF--TTGEAMRVVKVKSILEHSWEDT--SQKEIEKAVDNLKNEKPTFFNTET 168

VGC V+YL F + ++VV++K + E W+++ + ++IE+ + L+NEKP FFNT

Sbjct 3788 VGCPVKYLAFQQVHSDHIKVVQIKEVSELYWDESLPTPEKIEQQITELRNEKPKFFNTHQ 3609

Query 169 RSVLGLISQRLASSIDVETEYGQLTVELDNIEMNFIPTNGDRVRLECNIQLDDGFVDKQG 228

R++LGLI +R +I V+TE+ + + LD +E++FIP GDRV + CN+Q D+ FV++QG

Sbjct 3608 RNILGLIMERKPGTIVVDTEHKLMNINLDTVEIDFIPQAGDRVFICCNVQSDEHFVNRQG 3429

Query 229 EILEVTKLFPTRIQEGEKCIVERVYVHMVVLGPETYILKTDLPTGTDLHLGDIVLADLIE 288

E+L+ + P R+ + EKC V R+ VL + Y + L++GDIV+ADLIE

Sbjct 3428 EVLQEVSVHPARLLKQEKCEVRRLNSDWGVLNDDCYFTFDIVSNTCKLNVGDIVIADLIE 3249

Query 289 CQYSKFTRRAIKITPLEK------NFGATKLT-----QQssmgssgsgKAVTVTGVNRFI 337

C+ + R IK++ LE+ TK + + + S A+TV+ R +

Sbjct 3248 CERDPYIWRCIKLSLLERIVPQPTTPAGTKKPINGERRSTPKARNESNHAITVSEDQRCV 3069

Query 338 TAELWQKESVSLKltnnlnrtlrlESIT-VCNDSESQLSVVSPLESK---EISSGSEITV 393

+ ++ E V + + NN+ R +R+ SI + +SQ+ +V P + E+ T+

Sbjct 3068 FSSTFKTEKVEIIIKNNIERKMRVLSIEFLGRRRDSQVKLVDPELDETNFELPGHGSRTL 2889

Query 394 TFEIHTQFLGEAIEKYVLNFDLLKVRRVFTVIVCKTKEEVAEAEKRMIAAEALMAPGRN- 452

FE+ ++F GE+ E +++ F+ +V+R +++VC+T+ E A + + A +PG +

Sbjct 2888 HFEVESKFYGESREVFLIKFETFRVKRSISIVVCETEAEAEAARAQTSNSIANTSPGMHV 2709

Query 453 SQERSRFYANQVWCNKVDVIPGQQIVTKRRFVALRLGCFEVPKELRQICLTSERRQEMIK 512

++RSR YANQVW NK +VIPG + TKRRF++ R+ +EVP+ LR LT+ RQEM+

Sbjct 2708 MRQRSRHYANQVWSNKGEVIPGVGLATKRRFLSHRIAFYEVPERLRYTYLTAANRQEMVD 2529

Query 513 AIEQHYSFLTEPLSIKTYMHRFRLLLHLEEIECFVNFRNYDRDRAHFLRDGEFLTLQIEN 572

+E + L E L+IK Y RF+ L++LEE+E FVNFRNYDR+RAHF RDGEFL+L IEN

Sbjct 2528 NLEGMFPCLKEELNIKNYAIRFQTLVYLEEVEYFVNFRNYDRERAHFTRDGEFLSLTIEN 2349

Query 573 LAERRPSLVIGDTLRVINPWSDPDSQTTKSYEGIIHKVLFDRILLKFHSSFQEKYNGEDY 632

L+ERRPSLVIGDT+R INPW + D + +SY+G+IHKVLF+RILLKF+ FQ KYNGED+

Sbjct 2348 LSERRPSLVIGDTVRAINPWCNEDDK--RSYDGVIHKVLFNRILLKFNEGFQSKYNGEDF 2175

Query 633 RLEFYFSRYSFRKQHHAISKIVGVMGEDFLFPSKVTKRENPQLDVYM-KDDDMYLYDSKL 691

RLEFY+SR+ FRKQH+A+S+I+ +GE FLFPS+V R+NPQL + + D+++ L ++

Sbjct 2174 RLEFYYSRFCFRKQHYAVSRIIKNVGEQFLFPSRVYTRDNPQLQIELDSDNNLMLQGTRC 1995

Query 692 EWYNQSLNSIQKRAVFNILRGEAENIPYVLFGPPGSGKTMTLIETLLQLVRNLPGARILV 751

W+N LN IQKRA+ NILRGEA+N+PY++FGPPG+GKTMTL+ET+LQLV+ +P +R+L+

Sbjct 1994 PWFNPLLNPIQKRAIRNILRGEAQNMPYIIFGPPGTGKTMTLVETILQLVKLIPSSRLLI 1815

Query 752 GTPSNSSADLVTKRLIDSKALLQGDFIRLVSYNQVEKDLIPPEIMSYCATSDVGAVGSCE 811

GTPSN SADL+T RLI K L GDF+RLVS NQ+EK+LIP +M YCAT D+ A G+C+

Sbjct 1814 GTPSNGSADLITTRLIACKQLQPGDFVRLVSQNQIEKELIPDHLMPYCATVDMAADGTCD 1635

Query 812 DKMMVTESGLKLRCQAKFIGTHRITISTCTTLGNFLQLGFPAGHFTHVLFDEAGQCTEPE 871

D M+VTESG+KLRCQ K++G HR+TISTCTTLGNFLQ+GFP GHFTH+L DE+GQCTEPE

Sbjct 1634 DSMIVTESGMKLRCQMKYLGRHRLTISTCTTLGNFLQMGFPPGHFTHLLIDESGQCTEPE 1455

Query 872 TMVPIVMLTKKRSQVVLSGDPRQLQSIVTSRIASKMGFSISFLERLLERSPYRKDLQRFP 931

+VP+ +L++KRSQV+L+GDP QLQ+IV +R AS+ G S+S LER+L R+PY +DL RFP

Sbjct 1454 IIVPMSLLSQKRSQVILAGDPHQLQAIVINRFASERGLSLSLLERILGRTPYLRDLMRFP 1275

Query 932 ESSGYNPLVLTKLLYNYRALPSIMSIYSRLFYDDELIPVLSEKDSRESRLLSKLRCVFES 991

++SG++P ++TKLLYNYRALPSI+++YS LFYD EL +++E DSRE+ +L L + +

Sbjct 1274 DTSGFDPRLVTKLLYNYRALPSILNVYSELFYDSELRAMINENDSREADMLKNLDALLPA 1095

Query 992 EKDIPQAHGTFFYGIIGENRQNNDSPSWFNPQEVREVFLMTIALYRANVTADQIGIITPY 1051

+ P++HG FFYG EN+Q DSPSWFNP E R VFLMTI LYR + + IGIITPY

Sbjct 1094 SEKRPKSHGVFFYGTRSENKQEADSPSWFNPLEARNVFLMTIKLYRQGIQPENIGIITPY 915

Query 1052 QKQVKMLRSMFIGTDVVMPKIGSVEEFQGQERDIILISTVRSSEEILRMDARFSLGFVRC 1111

KQVK LR++FI D+ MPKIGSVEEFQGQERDIILISTVRSS++++ D R +LGFV+

Sbjct 914 MKQVKHLRTLFIEADIAMPKIGSVEEFQGQERDIILISTVRSSQKLISRDLRHALGFVQS 735

Query 1112 SKRLNVAVSRARAMMIIFGNPHLLAVDECWRQLILFCVKNNAYFGCDLP 1160

SKR+NVA+SRAR +M IFGNPHLL +D CWR I +CV N AY GCDLP

Sbjct 734 SKRMNVAISRARYLMFIFGNPHLLYLDHCWRTCIKYCVDNEAYLGCDLP 588

**Conserved domains**


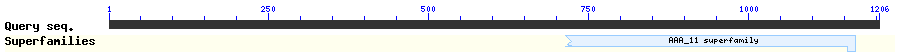


**Homeless (spindle-E)**

**>TRINITY_DN31966_c0_g1_i1 len=6268** CGCTTTTAATAAAFTGATTTTTGAATACAAATATCTTATTGGACATTGTTCAATTACAAAT

TAATTATATTTGCTGAGCACTTATATATTTCGTAGATTATGCTGCAATCGCGTTATGAGT

TGGCTGGCTGGACGTTGAGTACACGTCCTTCGATGAAATGGGTATTTTTATTAGAGGCAC

TATTGAAACCATCGCGGCTGCTAAAGGACACAAATCCATAGCCTTTAGAGAAACCAAGCT

GCTTATCAAATACAACAGCGGCATTTGCAACATGACCAAAATTTGAAAAGTAGGACTGCA

GCTCTTTTTGGCCAATTGTCCAAGGCAGATTACCAACAAATAGTTTGTATGAATTACGTA

CTGCTCCAGACATTGTAAGTTTGTTTCTGCTTAAAGAAAAATAATTGCTTAAAAATAAAC

CACTCTCAGCGCTCTTTTGTTATTTCAATCAAGTGTATGGTATGCCCGCCCATTAATTCC

ACCAGATCAATACCAGAGCTTTTGCCAACGTTATCAAGAGTTATCGGCACAGCATGCAAT

TTCCACTTCTTTAGCAATGCTTCAATAGACCAATCTGCACTGCGCTCTTGATACGTACAA

AGAAATTTTGCACCCGTATTACGTTCCAGTAGAAATGAAATGCTTACTAGTATATCTTCG

AATACGCTTGGGTCGTAAAAGCAATCAGCACCAATTATCAAATCAAGTGGTCCTATATTG

AAAACGCTATTCAATAGTAGGCCCCAACTGAGGCCAATTACTTCTATGTCCTTCCCTGGG

ACGAGATTATTAGTCAAACAAGACTTTCGTATGTGCGCTAATGATTTGGGTAGTATGCAA

TTGTCGCTAAGTATAACTCGTGCACCACACTTTGCAGCCAAAATGCCAGGAAGCGCTGTG

CCCGCACCCAATTCAAGTACACGTTTGCCGACAAGATTCCGCCGCCTCTCCCATAAAAAC

CAAGCCAACACGGGGGCACAAGGCCAAGTGTAGAAAGAGTAACCGCTTTGTAGAAGCTCA

GGTATTTTAATCTCCAATTTTTCTAAAGGTTCCGCTGTTGCACCTGATTCAGAAACATTT

GCGCTTTGATTGGTAAAAATAAACTTTCTTATATGCTCCGCAATATCTCCACTGCTTCCA

CTGCACGCACTGTCGATTTGCGTATTGGCCGAACTTGTTGTTGCTGTCACTGTTGTTGTC

GAAGCTGTTGTTTGTTCATTATCAGTTCCTTCATTCTGTTGATAACCGCTAGTCATTTTT

GGAAAAACTCAAAATTAGACAATGCTATGTATTAACGTAAAATGAAAATTTCTTATGTTT

CGACAGAAATTAAAACCCAACGGGATTAAAACTAGCGGGGCTTTAAAACTTCAAGCCGAT

ACCAAAGATAATCCGATAGCGAAACTGAAATTTCCCAATTGGTACAAACAATGCAAACAA

GTAGTGAACAATAATTGGCAGCACTTTTCAGGGAAATAACGGAGAGAAATATTTACAGAA

GAATCTTCTTCTGCGATAATTGTGTAAATTGTAAAATTTTTAGAGGCTTGTTGGCAAACG

TAAAGTATTTTTTTTTTATAAACTTGATTGTTTATTGCAACGAAAATGAATGAAATAGAA

GAATTCTTCAATTTCACGAAAGACTTCAAACGTGAGCCTGCACTTCGTGGTTACATTTCA

GGAGACCTCGCCGCAACTGCGGGAAAATTTGATAATGGAAAACCCATTAAACGCGAATAT

TTTGGAGATCAGTATTCAAAGCCTGTTGCTGAAAAGGAACGGAGGAGAATGCTGGATGAA

GATGTTTCGATGGAAGAAATTCATTCTTCACGGCAAGGTGCGACTTCGTCTACTTGTATG

GATGAACTTGAAGATTTGAGTGAAGAAGAAGATGCTAAGCCAAAAATACTCCGAATCGAC

GATGGTGTATACTCCAAATATAATTTCAATCTGAAGCGCGATGAATCGTTGCCTATACAT

GACAACAAAGATCAAATATTAAATGCCATACGTAAGAATCCTGTTGTAGTGCTTGAGGGG

GATACTGGCTGTGGCAAAACAACGCAGGTGCCTCAATATATACTTGACGAGGCCTATGAG

AATCGAGACTACTGCAAAATTGTCTGCACACAACCACGCCGTATTGCGGCAATATCAATT

GCTAGACGTGTCTGCCAAGAAAGAAAATGGGAAGAAGGTTCTGTGGTCGGTTTTCAGGTT

GGCCTGCATGCCAATATTAGCGAAGATACACGCTTGATTTACTGCACGACTGGTGTGCTG

CTACAGAAACTGATTAAAGAAAAATCACTCAAACAATTCACACATATAATTCTTGATGAG

GTGCACGAACGTGATCAGGAAATGGACTTTCTACTTATAGTCATAAGAAAGTTGCTAACT

ACAAATTCACGTGATGTAAAAATCGTACTTATGTCAGCCACCATTAATGCAGGAGAGTTC

TCCGACTACTTTACTATACGTCGCAAACCAGCGCCAGTGCTGCGTGTGGACTCGCGTCGA

CTTTTTCAAGTACGCGAATTCTACTTATCCGATCTGGGGCGTATAAATAGTGCAAACATC

GACGTGGACATTTCTGATCCTGGCATCTCTAAAGAAATGTTTAATATCGCACTTAAATTG

ATTATTGTTATAGACAATATAGAAAAACAGGAAGCCGCTGTGGCAATAGATAATGGCCCT

TTACAACAAACTTCGATACTCATCTTTTTGCCGGGTATCAACGAAATTGAACAAATGTCC

AGCAAGTTAAGATTACTAAGCGAATCTGATGACAACAATGTAAAACTCTATCCCATACGT

TTACATTCTTTAATCTCGCCGGATGAACAGAACAAAGTATTTAACAACCCACCTGGGGGA

TTTCGAAAAGTAATATTGTCTACAAATATTGCAGAGAGTTCTATCACCGTGCCCGATGTT

AAATATGTCATTGATTTTTGTTTGACGAAATCGCTAATTACTGATACTGCAACAAATTTC

TCTTCACTGCAATTGCATTGGGCGTCGCGCGCAAATTGCCGTCAACGTGCCGGCCGTGCA

GGTCGTGTCATGAATGGCCGCGTCTATCGTATGGTCTCAAAGAATTTTTATGATCATTAC

ATGGAAGAGTTTAGCACCGCGGAAATGCTGCGTTGCCCGCTGGAGAATGCTGTGCTGAAA

GCCAAACTGCTCGATATGGGTCCACCGCCTGATATACTCGGCTTGGCAATGACACCGCCA

AATCTTTCCGATATACACAACACCATATTGACGCTGAAGGAGGTGGGCGCACTTTTCACA

ACTGTCAATGGCGTTTACTCAATACAAGATGGTGATTTGACCTTTATGGGGCGTGTGATG

GCCGGCATGCCATTGGATATACGTTTAACACGTCTCATATTGCTGGGCTATATCTTTAGT

GCTCTGGATGAGACGATTATAATGGCTGCCGGCTTAAGCGTGCGTTCAATTTTCAAGTCG

AGCGTCGATCGTCGTGGTCAGGGTGAAGCAGATGCATATATACAAAAACTTGTCTGGGCA

GATGGTTCTGGTTCTGATTTATTTGCTATTTTGAGTGCTTATCGCGTTTGGAGTTCAATG

CGCGAACAACAAAACATCCACGAGGAAGGGCCCGAATACAATTGGGCCAAACGTTTCTTC

ATTAATTTACGTTCAATGAAGGAGATGCATTTGTTGGTTACAGAGTTGCGTAGCCGTCTA

AAATCCTATGGCATACGTGAACAACAGGCTTATCAACGCGTTTGTTGGATTGATCGGGAG

ATGACAATTATATTGAAAATAATCATAGCTGGCGCATTTTATCCCAATTATTTCACACGT

TCCAATTTGAATGACACGGAACGTGAACGTGGCATTTATCACACACTTTGTGGCAATGAT

CCTTGCAACACAGTCTATTTTACTGGTTTCAATACACGTCACATTGGTCAGCTGTATGCG

GGCTCAATCAAAGATCTGTTTCGTGCTGTGCGCATACATCCTAAAAATATTGAGGTACGT

TTTCAACCGGGCGCCGAGCGTGTGTTTGTCACATTCAAAAATGATCGGGATGACGATTCT

GAAGGTGGAGCCTACAGGCTGGTTGTGCCAGGACGTGTATGTCCTGAGGTATACAAGGCC

GTGCGCATGCGTATGCTCGGCATGCGCACCACCATGCGTGTTATGGACCCACGCAACGAG

GTGAAATATGCGGAGGAGCGCCATATTGGCAGGATGGTTGAAGGCATTTGGCAGCCGACT

AAGAAACAAATAAAAAATCCCGAGTTAATTGTATTGCCATCGGTTTTCCAGAAAATGATA

CGTGGCTACATTACACATATTGAAAGTTGCAGGAAATTTTATTTCCAACCGCTATCTGAA

ATGGAGCGCCTACGCGAAATTAATGCCTTGTTAAATAACCCAGAAGACCTGGAGGGTGGT

CGTTTCAGAAACCCTGCGGCCATATCTAAAGGCATGATGGTGGCGGCACCGTTTGAAAAT

AAATATCATCGCGCTACAGTCTTGAAAGTGTTGACTGCGGCTCGGCAGCATTGTCAGTTT

AAGGTATTCTTCGTCGACTATGGCAACACTGATGTTATAGATTTCGAACAATTGCGTCGT

TTGCCCTATCGCTGTGAGTCCCTCGTTGACATACCACCACGTATGTTTGAATGCCGCTTA

GTTATGGTGGAGCCATCATCGGTGAAGTCGCCAAGCGGAAAATGGCCCGAGGAGGCTATG

GAATTTATGCAGCAGACAGCCGATGCTGGTGTAGTCGAAATAGAAGTTTACTCCGTGGTT

GCCGGTGTTTCGAATGTCATTATAAAAACCGCCACAGGCACGCTCAATGACATACTGGTG

GAGAAGGGACTTGCCCACAAGTCAGATGAGAATTACATGTCCAAGGCCGATCATGATTTT

CGTTTGCGTAAACAATCAGTTGCTACACGTTTCCTAGATGAAGACCATTCCAAACAAAAT

GAAGAGTATATGCGTTCAATACAACAAGAAACCGACTTGGAAGTCGATCCACCACCACGC

GAGTATTGCACCAAAGCTATTAATTTGCGCGGTCCATTTAGTGCGCTAGAAACAAAGATT

TGTTCAGCTGTCCGCATAGGTACCTGGAAGAGTGTGAATGTCGAACGCGACTCTGTGAAT

TCGGTGTTGATCGATACAGACCCGCAAGATGTGCATGAGCGTCTCATTGTCGCGGCCAGT

ATAACGGAAGCTCAAAGTGCGGAAACGCTTACTGCTCGCTCTACTACGCTAATGCCAAAT

ATACACGGTTTTGGTGCGTTAATGACGCTGCTCTTCTGTCCTACTATGCAGATAAAACGG

AATAGTAATAAGACCAAATATGTTACGGTACTTGCGGGATTGGGTTACAACGAAGAAACA

TATAAGCCACTCTATGAGGAACATGATATCGTGCTAAATTTGGATGCAGAAATTTTGAAG

GATGATATAGAATTGACCAATCAGCTACGATATTGCATGGACACAATGTTGTATACCGAT

CCAGGTTGTGAGACTTTACCGATATTGCCAAATACACGCGCAGATTTGTCAGCCAAAATT

AAAAACTTAATTATAAGACTCTTAAATAAAAATCGCAAATATATCGAGACCCACGTAGAT

AGCCTCGACAATGTCTGGCAACGTTACGATCCGGAAGAAGTGATCGAGACAGAACCCATC

TATGGTAAACGTTCAATATTTCCCTTACACGCTGCACTCAAGTTGTACGATGAGAAATTC

GATCGCATACACGCTTTGGGTGTACACTGTCAGGAGCTGCATCGTTTGCGTCAATTTGAT

GGCTCCATTCAACCGATCACTTGTCAGCTATGTAATCAATCACTGGAGAATATCGTACAA

CTACGCATACATTTGCTCTCACAGTTGCATCGTGATCGCGAATATCAGATACGTTTCAAA

ATGCCACGTTAAATGGCATAGCTTTTAATTTAGAATTTGCAAAGAATTTAATTTGCAATT

ACTGTAACTTTTATCGAAATGTTTTCGTTTATGATCTTTAAAAATTGAATTGTATGTTTT

AGTTTCTTTTTCTATGAAGAAAATTTCCAGAAATTTTCTAGATGTACGACCTTTCTTTAT

GTAAATTAAATGGACAAAGAATTCACAAATTTTCTAGATTTATGAAACTTTTTTACGTAA

ATTAAATGGAGAAAAAATTCACAAATTTTCAAGATTTATGACACTTTTTTATGTAAATTG

TACACCCCTCGAATTAATTGAAAAGGCA

**tBLASTn(First hit)**

Score = 1281 bits (3315), Expect = 0.0, Method: Compositional matrix adjust.

Identities = 701/1448 (48%), Positives = 953/1448 (66%), Gaps = 26/1448 (2%)

Frame = +1

Query 4 EVMDFFDFSKELKRVAAAPQGYISSDPRLMATKFKSSEVPNRELIGTDYVSKIVAKEKC- 62

E+ +FF+F+K+ KR A +GYIS D A KF + + RE G Y + KE+

Sbjct 1612 EIEEFFNFTKDFKR-EPALRGYISGDLAATAGKFDNGKPIKREYFGDQYSKPVAEKERRR 1788

Query 63 LLNGTLLNEQ----PQGKrirtlddldtddegeeteirrdde----YYKKFRFNLNRDKN 114

+L+ + E+ QG T D D EE + Y K+ FNL RD++

Sbjct 1789 MLDEDVSMEEIHSSRQGATSSTCMDELEDLSEEEDAKPKILRIDDGVYSKYNFNLKRDES 1968

Query 115 LSIYAKREEILAAINAHPVVIIKGETGCGKTTQVPQYILDEAYKSGKYCNIVVTQPRRIA 174

L I+ +++IL AI +PVV+++G+TGCGKTTQVPQYILDEAY++ YC IV TQPRRIA

Sbjct 1969 LPIHDNKDQILNAIRKNPVVVLEGDTGCGKTTQVPQYILDEAYENRDYCKIVCTQPRRIA 2148

Query 175 AISIANRVCQEREWQQNTVCSFQVGLHRPNSLEDTRLLYCTTGVllnnlinnktltHYTH 234

AISIA RVCQER+W++ +V FQVGLH N EDTRL+YCTTGVLL LI K+L +TH

Sbjct 2149 AISIARRVCQERKWEEGSVVGFQVGLH-ANISEDTRLIYCTTGVLLQKLIKEKSLKQFTH 2325

Query 235 IVLDEVHERDQNMDFLLIVVRRLLATNSRHVKIILMSATIDAKELSDYFTTTNSIPPVIT 294

I+LDEVHERDQ MDFLLIV+R+LL TNSR VKI+LMSATI+A E SDYFT PV+

Sbjct 2326 IILDEVHERDQEMDFLLIVIRKLLTTNSRDVKIVLMSATINAGEFSDYFTIRRKPAPVLR 2505

Query 295 TNHRRKHSIEKFYRDQLGSIIWNEEDVGHQQVPEINKHGYRAAVKIIVIIDNMERKAAIQ 354

+ RR + +FY LG I DV P I+K + A+K+I++IDN+E++ A

Sbjct 2506 VDSRRLFQVREFYLSDLGRINSANIDVDISD-PGISKEMFNIALKLIIVIDNIEKQEAAV 2682

Query 355 SRQSYDEALRYGAVLIFLPGIYEIDTMAENLTCMLENDPN-IKVSIVRCFSLMTPENQRD 413

+ + L+ ++LIFLPGI EI+ M+ L + E+D N +K+ +R SL++P+ Q

Sbjct 2683 AID--NGPLQQTSILIFLPGINEIEQMSSKLRLLSESDDNNVKLYPIRLHSLISPDEQNK 2856

Query 414 VFNPPPPGFRKIILTTNIAESSITVPDVSYVIDFCLAKVKVTDTASSFSSLRLTWASKAN 473

VFN PP GFRK+IL+TNIAESSITVPDV YVIDFCL K +TDTA++FSSL+L WAS+AN

Sbjct 2857 VFNNPPGGFRKVILSTNIAESSITVPDVKYVIDFCLTKSLITDTATNFSSLQLHWASRAN 3036

Query 474 CRQRAGRVGRLRSGRVYRMVNKHFYQREMPEFGIPEMLRLPLQNSVLKAKVLNMGSPVEI 533

CRQRAGR GR+ +GRVYRMV+K+FY M EF EMLR PL+N+VLKAK+L+MG P +I

Sbjct 3037 CRQRAGRAGRVMNGRVYRMVSKNFYDHYMEEFSTAEMLRCPLENAVLKAKLLDMGPPPDI 3216

Query 534 LALALSPPNLSDIHNTILLLKEVGALYLTVDGIYDPLDGDLTYWGTIMSRLPLDTRQSRL 593

L LA++PPNLSDIHNTIL LKEVGAL+ TV+G+Y DGDLT+ G +M+ +PLD R +RL

Sbjct 3217 LGLAMTPPNLSDIHNTILTLKEVGALFTTVNGVYSIQDGDLTFMGRVMAGMPLDIRLTRL 3396

Query 594 IILGYIFNMLEEAIIIAAGLSTPGLFAHEGGRSQLG--DSFWMHYIFSDGSGSDLVAIWR 651

I+LGYIF+ L+E II+AAGLS +F R G D++ +++DGSGSDL AI

Sbjct 3397 ILLGYIFSALDETIIMAAGLSVRSIFKSSVDRRGQGEADAYIQKLVWADGSGSDLFAILS 3576

Query 652 VYLTYLNIVE--NGHDQESAIRWAKRFHVSLRSLKEIHLLVQELRVRCTHLGLIPFPVNP 709

Y + ++ E N H++ WAKRF ++LRS+KE+HLLV ELR R G+

Sbjct 3577 AYRVWSSMREQQNIHEEGPEYNWAKRFFINLRSMKEMHLLVTELRSRLKSYGIREQQAYQ 3756

Query 710 NQMMDDREKAIMLKVIIAGAFYPNYFTRSKESCADTDRNIYQTISGHDPCRTVYFTNFKP 769

DRE I+LK+IIAGAFYPNYFTRS + + +R IY T+ G+DPC TVYFT F

Sbjct 3757 RVCWIDREMTIILKIIIAGAFYPNYFTRSNLNDTERERGIYHTLCGNDPCNTVYFTGFNT 3936

Query 770 AYMGELYTRRIKELFQEVRIPPENMDVTFQEGSQKVFVTFK--QDDWIEGSSKYVPVSGR 827

++G+LY IK+LF+ VRI P+N++V FQ G+++VFVTFK +DD EG + + V GR

Sbjct 3937 RHIGQLYAGSIKDLFRAVRIHPKNIEVRFQPGAERVFVTFKNDRDDDSEGGAYRLVVPGR 4116

Query 828 VQSEVYKAVMMRQNRVERPIHIMNPSAFMSYVQQRGIGDVIEGRWIPPTKPL-NVELLAL 886

V EVYKAV MR + + +M+P + Y ++R IG ++EG W P K + N EL+ L

Sbjct 4117 VCPEVYKAVRMRMLGMRTTMRVMDPRNEVKYAEERHIGRMVEGIWQPTKKQIKNPELIVL 4296

Query 887 PSVFDKTISGSITCIVNCGKFFFQPQSFEECIRNMSEIFNAPQQLR-NYVTNASAIAKGM 945

PSVF K I G IT I +C KF+FQP S E +R ++ + N P+ L N +AI+KGM

Sbjct 4297 PSVFQKMIRGYITHIESCRKFYFQPLSEMERLREINALLNNPEDLEGGRFRNPAAISKGM 4476

Query 946 MVLAKRDSYFQRATVIRPENQSNRQPMFYVRFIDYGNCTllpmqlmrlmprelTEQYGDL 1005

MV A ++ + RATV++ + + F V F+DYGN T + E D+

Sbjct 4477 MVAAPFENKYHRATVLKVLTAARQHCQFKVFFVDYGN-TDVIDFEQLRRLPYRCESLVDI 4653

Query 1006 PPRVFECRLAMVQPSSVVSGNNRWSTAANDMLKTVAQCGLIDIEVYSLFNNVAAVLIHMR 1065

PPR+FECRL MV+PSSV S + +W A + ++ A G+++IEVYS+ V+ V+I

Sbjct 4654 PPRMFECRLVMVEPSSVKSPSGKWPEEAMEFMQQTADAGVVEIEVYSVVAGVSNVIIKTA 4833

Query 1066 DGIINDKLVELMLCRRSDEDYMSRKDHDFRLRRQESARNLSTAQRQQINEEYLRSCqlpq 1125

G +ND LVE L +SDE+YMS+ DHDFRLR+Q A + NEEY+RS Q

Sbjct 4834 TGTLNDILVEKGLAHKSDENYMSKADHDFRLRKQSVATRFLDEDHSKQNEEYMRSIQQET 5013

Query 1126 dhdlpppplEKCKTVVMLKGPNSPLECTMRSITRVGLSKRVNIDHLSVNALLLDADPQDH 1185

D ++ PPP E C + L+GP S LE + S R+G K VN++ SVN++L+D DPQD

Sbjct 5014 DLEVDPPPREYCTKAINLRGPFSALETKICSAVRIGTWKSVNVERDSVNSVLIDTDPQDV 5193

Query 1186 HDHLIVAHEIAESRNGQTLTARGTTLMPNVQGFGALMVMLFSPTMQLKCNKEGTSYVSVL 1245

H+ LIVA I E+++ +TLTAR TTLMPN+ GFGALM +LF PTMQ+K N T YV+VL

Sbjct 5194 HERLIVAASITEAQSAETLTARSTTLMPNIHGFGALMTLLFCPTMQIKRNSNKTKYVTVL 5373

Query 1246 GGLGCDPDTNEPYFAEHDVLINLDVNILEDDVILINQIRYYIDSVFFNFKEENNPAVSVN 1305

GLG + +T +P + EHD+++NLD IL+DD+ L NQ+RY +D++ + + N

Sbjct 5374 AGLGYNEETYKPLYEEHDIVLNLDAEILKDDIELTNQLRYCMDTMLYTDPGCETLPILPN 5553

Query 1306 ERVSIYTQLRSLINRLLCKDRRYIERNMSNADFEWETn--pelplpnepFGKRAIFPMHS 1363

R + ++++LI RLL K+R+YIE ++ + D W+ E+ +GKR+IFP+H+

Sbjct 5554 TRADLSAKIKNLIIRLLNKNRKYIETHVDSLDNVWQRYDPEEVIETEPIYGKRSIFPLHA 5733

Query 1364 LTELQEEDTGRLVQLRENCSMLHKWRNFEGTLPHMTCKLCNQLLESVPQLRLHLLTILHR 1423

+L +E R+ L +C LH+ R F+G++ +TC+LCNQ LE++ QLR+HLL+ LHR

Sbjct 5734 ALKLYDEKFDRIHALGVHCQELHRLRQFDGSIQPITCQLCNQSLENIVQLRIHLLSQLHR 5913

Query 1424 DREKQIDY 1431

DRE QI +

Sbjct 5914 DREYQIRF 5937

**Conserved domains**

**DEXDc and TUDOR domain-containing protein**


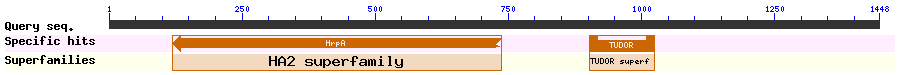


**Maelstrom**

**>TRINITY_DN28061_c2_g2_i5 len=2033**

ATTGACATTAAAATGCATGCTCATTGCGGCAAAAATCAATTCCAGTTAGTGCGTAAAATT

TATTTGCTTGTCAACAATTTGTTAATTGTTTCACAAATTGATAAACCAAAGAAAAGTTTT

TGGGAAGCTTTATTTTCAGCTCAAAAAAGAAAACTTATTAATTTAGTGCAGCATTAAAAC

GATATATTCAAAAATGAGGAAAAATAAAAAGCCGAATGGTTTTCTTACGTTCACTATTGA

ATGGAAGTCAAAGTATGGTAAAAGGATGACTTTGAGCCAAGCTACGGAAGAAGCAGGCAA

AATTTGGATTTCGATGAGTATGGAAGAACGCAGCCCTTATAATGAGCGTGCTAAACAAGA

GAGATCTAAATTAAAATCTGCACCACCGAAGCTAACCTGCACAGGGAAACCCTTAGATCA

AGTGGAAAAAGATTTACAAGTGGCAGATCAATGGAATGTTCAGATGAAAAGAAATATAGA

AATGGTGGTGCGCAATAGTGTCAATAATGACCAACTTAAAACGCAATCATATTTTTTTGT

AATGGTTAATTATTTTGTGAAATCGCTCAGGGGTGATATCTATGTACCAGCCGAAATATC

TGTTGCTGAATATTCATTGCAGGAGGGCGTTTGCCGGAAATATCATACGTTAATAAATCC

TGGTCGCGACTTGTACGGTCTGCAGTTTGATGCGCAAGATCATGCGGACCGCACACACAA

GCTTCCGTTGCCACCAAATGCTTTGGGTGAAGAGAATTTGGGTCTGATTTATAATCATGT

ATTGGACTTTGTACGCGACCCAGAAACTGGGGATTATCCACCCATTTATACACATCGTGA

TTCAATACCAATTGTTAAGTCTGTATTGGACTTCCTCAAAAATTCCAGTTCAACTAATGT

TGAATTGAAGGTATATTCCATACAATACCTTTTCTTTATACTCAAGGAAGCTACAGCAAA

AGAGGGTGACGTACGAAGCCCAAAATGTCACTACATAACTGATGCTTGCTTTGACCGTGA

TTTTTACGAATATCAAACTGATATTGCCTGCAATAAACATGAAGAGATGGACAAAAGTAA

ATATTGTACACAATCGTGTGTAACCCGCTGGGGATACATTTTCTCGGATTTTATGTGTGG

TGACGTCGCTATCTCTGTGGTAGAAGGACGACACAAACCGATTCGTACGGATGTAGACCA

CAGTGACAATAACAATCCGGCACCACCGAGTACTTGCTTCGATACTGAATCACAAATTTC

TGGAAATAGCGAAAGCACATACGCCACCAGGCCATTTTCAGAGAACAGAGTTTGTTATGA

TGACCACAAAACATTTATGTCGGCACATTCAAGTGCTATCGGTACCTCTACGAACAATAA

CCAGACCAATGACTTTCCTTATTTGGGAGCTAGAAAGAAAACTTCAAAAATTACTTCACC

TTCGCCACCAAGAAGCAGTCGCTGGAACACGGAAGGGTTTGATGGCAGAACACTTGATCT

TGATTTGTGCGAAGGTGGGAATCCGTGGTCAATACGTTCGCGTGATGTGCCACGCGAACC

AGATACATCGCACTTCGATATCAATTACACTCGCGACGAAACAACCGACAATGATAGGCC

TGGAACTTCCAGTGGTTATGGACGTGGAAGAATGCGTATGAATCATTCTGCCACTAGCAA

TACAACTGTAGGCTCGGGACGTGGCAGGCTATGTCGAAATCCCGACTACCGCAATTAATG

AAATAAAGTGTTTAATTCTAAAACTATGTTTATACAATTAATGTATATCCAAATAAGGCG

TAGAGTATTAGCAAAAAGTGCGCGTATTTCAATATTTAATTGTAAGTAAAAAAAATTACA

ACATATCGTTACAAAAACATGCACAAAGAGTCCCTAACCAACATGTTTGAAATTTAACAG

AAGCAAAATCCATTAAGGGCAATCACAACCCGTGCATATGACCTTGATTGTGCATAATTC

GCTGCATTTGCAAAATTTCTGTTGCCATGAAATATGAATATGCAGGCATATAT

**tBLASTn(First hit)**

Score = 279 bits (713), Expect = 6e-085, Method: Compositional matrix adjust.

Identities = 163/433 (38%), Positives = 240/433 (55%), Gaps = 31/433 (7%)

Frame = +2

Query 4 KKHSGFMMFVNEWRNHNAEGRRMTLAQAVSHCGTIWEKMTTQQRGPYNSGAKDADVADRG 63

KK +GF+ F EW++ G+RMTL+QA G IW M+ ++R PYN AK +

Sbjct 206 KKPNGFLTFTIEWKS--KYGKRMTLSQATEEAGKIWISMSMEERSPYNERAKQERSKLKS 379

Query 64 KRERLNCYGQGIAQVDLAHKEAAESLMHMKRTTERLVINAKKSYDLENAKFVFATFNYFT 123

+L C G+ + QV+ + A + + MKR E +V N+ + L+ + F NYF

Sbjct 380 APPKLTCTGKPLDQVEKDLQVADQWNVQMKRNIEMVVRNSVNNDQLKTQSYFFVMVNYFV 559

Query 124 KALTTDVYVPAEFAACEYSLKEGVRSIYSTMIDPGQIIFGQGSDALHHSSTTHDLPLPPN 183

K+L D+YVPAE + EYSL+EGV Y T+I+PG+ ++G DA H+ TH LPLPPN

Sbjct 560 KSLRGDIYVPAEISVAEYSLQEGVCRKYHTLINPGRDLYGLQFDAQDHADRTHKLPLPPN 739

Query 184 ALGEKNMAKLYRNIVCYLTKCQGADKPLIVFTPTENIAMVNSCFRYLECEDDSGDGGRKI 243

ALGE+N+ +Y +++ ++ + D P I +T ++I +V S +L+ +S ++

Sbjct 740 ALGEENLGLIYNHVLDFVRDPETGDYPPI-YTHRDSIPIVKSVLDFLK---NSSSTNVEL 907

Query 244 QVFDIQYLLFILKKAVMDVAGLNDEKINKFVTDAFFKKDFFEFTSGIACQYHEDNDRTKY 303

+V+ IQYL FILK+A + K + ++TDA F +DF+E+ + IAC HE+ D++KY

Sbjct 908 KVYSIQYLFFILKEATAKEGDVRSPKCH-YITDACFDRDFYEYQTDIACNKHEEMDKSKY 1084

Query 304 CTQSMVTRWAYTFSDFMCGDLAITVQPGKHIPAET--------KPNYRIICSDASSLAHE 355

CTQS VTRW Y FSDFMCGD+AI+V G+H P T P C D S

Sbjct 1085 CTQSCVTRWGYIFSDFMCGDVAISVVEGRHKPIRTDVDHSDNNNPAPPSTCFDTESQISG 1264

Query 356 SSFESFYSCPGS--RVKKETQSEDFSLSSSQISVASRSYTPTDHTSFTTDLTKVCEFPSL 413

+S ++ + P S RV + S SS I + T+ + +FP L

Sbjct 1265 NSESTYATRPFSENRVCYDDHKTFMSAHSSAIGTS-------------TNNNQTNDFPYL 1405

Query 414 GMR-KSSKHTGPS 425

G R K+SK T PS

Sbjct 1406 GARKKTSKITSPS 1444

**Conserved domains**


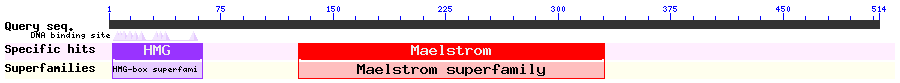


**HEN1**

**>TRINITY_DN27986_c1_g1_i3 len=1479**

GCCAGACCTTAACAACAATCCACTTTATTTCAGCTATCGCGTATGTACATACATACATAC

GTAGTTAGAAACAAGTATACATGCTTAGTGTATGTACTTATGTAAATATGTATATGAATT

CACGATTTCCAAATTAAAAAATGCTGTACTGTAAGACTACTTGCTTATATTTAATCCCAA

TTTTCCTCAGATTCGTAAGTGACATCATCATTGGCGAGTGAATGTCCACCGCATGCGCCC

AATTCGTCGTCATTATCACATCCAGTATCGTTCCACAAGCCGTATTCATCATCATAACAA

TCATCATAATGACGATAGCTCAAATCGTCTTCATCATATTCAGGCAATATGATATAATCG

CCCTTTACCTCTATATCATTGTCCTTTAAGACATCCAGTAACTCTTCCGTTGAGGCCCCA

AGAGTTTCAATATATGTGTCCATAACTGTAAGTGGCACTTGATATACCCCTAAATCTTGA

TTGAAGTATCTATCTATGCGACGGCATCGGTCGATTTGATAACGCGCCTCGTCCAAAATT

TTCTTTTCCTTACTGCGTTCGTCGACATTAAATGGGAAATCGACAGCAAATATTTCCTTA

TACTGCACATCTGCATCAGTATTACAATCGACTGGTACTTCGGAGCGCAACGGTTCAACT

AGGCGACGACCAAGCAGATCGTGACGTGCAAAAATTGCAATTTGAGACACGTTACCAATA

CTCTTGTTATCCGGTGGTGCATCGCCAACACCCAAAAAGGCAACACTATAATTTGGATAT

TGCTGGCAAATTGTTCGCGCCCAATCGCGGAATTCGGCGCGCGTCCATTCGAATTTGTGA

TCATCGTGGCGGAAACCATTGGCCAATAATGGTTGAAAAAGTACATTGAATTCAGAGTTG

GGTGTTGAAAATATTGCGATTTTAGGTTGCATAAATCCGAAAATGTTCTTCGGGACATTC

TCCAATGTTTTGGGGTACAAATGTTCAATTATTTCCAAGGCGACAACTGCATCTACATTA

AGCAACTGTTCGACCGATGCATCGATACTTCCTTTTAGTAATTCCACACGCAAAGGATTT

TCACGCCTCATTAAATAATCCGATACGAGTGGTTCTGCTCTCAATTTATTGCTTCGCAAA

ATATTTTCGTCAACATCAACCTCCAGGATATGCTCAATGCCTTTAACACGTCTCAGTAAT

GGCAACAAACGCATATCCGCACATCCAAAATCCACCACTTTTTTAAATTTCTGTCCCCAA

CGTGAGTCTTCCAGTATGCGAACTGCTGTAGTGTAACGCTGTTCATACACAGGAGGATCG

AACTTGATACCACCTTCGGCATTTATATAACAACAAGTACGCAGACCTCCATCCGGCAAT

TTATAGGAAAACATTTTTGATAACTTTACCGGTGAAAATCAAAAGTTCGCCGCTTTGTTT

ATAAATGATTGTATGTTTACGATGCCGCTAACTACATAT

**tBLASTn(First hit)**

Score = 319 bits (817), Expect = 3e-103, Method: Compositional matrix adjust.

Identities = 158/339 (47%), Positives = 227/339 (67%), Gaps = 14/339 (4%)

Frame = -2

Query 1 MFSHKFICGSLTKM----TETGITFDPPVYEQRYCATIQILEDARWKDQIKKVVEFGCAE 56

MFS+K G L E GI FDPPVYEQRY ++ILED+RW + KKVV+FGCA+

Sbjct 1394 MFSYKLPDGGLRTCCYINAEGGIKFDPPVYEQRYTTAVRILEDSRWGQKFKKVVDFGCAD 1215

Query 57 MRFFQLMRRIETIEHIGLVDIDKSLLMRNLTSVNPLVSDYIRSRASPLKVQILQGNVADS 116

MR L+RR++ IEHI VD+D+++L N PLVSDY+ R +PL+V++L+G++ S

Sbjct 1214 MRLLPLLRRVKGIEHILEVDVDENILRSNKLRAEPLVSDYLMRRENPLRVELLKGSIDAS 1035

Query 117 SEELRDTDAVIAIELIEHVYDDVLAKIPVNIFGFMQPKLVVFSTPNSDFNVIFTRFNPLL 176

E+L + DAV+A+E+IEH+Y L +P NIFGFMQPK+ +FSTPNS+FNV+ F PLL

Sbjct 1034 VEQLLNVDAVVALEIIEHLYPKTLENVPKNIFGFMQPKIAIFSTPNSEFNVL---FQPLL 864

Query 177 PNGFRHEDHKFEWSRDEFKNWCLGIVEKYPNYMFSLTGVGNPPKEYESVGPVSQIAIFVR 236

NGFRH+DHKFEW+R EF++W I ++YPNY + GVG+ P + +S+G VSQIAIF R

Sbjct 863 ANGFRHDDHKFEWTRAEFRDWARTICQQYPNYSVAFLGVGDAPPDNKSIGNVSQIAIFAR 684

Query 237 KDMLEMQLVNPLVSKPNIDKES---IPYKLIHTVEYPFYVDTRTEKEKLWTEVQIELQRF 293

D+L +LV PL S+ +D + + YK I V++PF VD R++++K+ E + ++ R

Sbjct 683 HDLLGRRLVEPLRSEVPVDCNTDADVQYKEIFAVDFPFNVDERSKEKKILDEARYQIDRC 504

Query 294 KRQFESSEIEEGTYQDTCNMPIAFLLDRLEHVGATKERI 332

+R + G YQ +P+ + +E +GA+ E +

Sbjct 503 RRIDRYFNQDLGVYQ----VPLTVMDTYIETLGASTEEL 399

**Conserved domains**


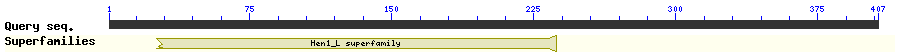


**RNA helicase Belle**

**>TRINITY_DN28586_c1_g3_i2 len=4358**

CTATAGTTTTAGTATTCACTTTGAAATCGTGCTTTCTTGATTTCTGGAATAAATAAATAA

AATATACTCTAAAACTTAGGTGCGCAAATTTCTCTTCTACACAATCAACAAATTTTTAAA

ATCACTACGGGAAGTAGCAACGTATTTGAAATACTTTTACAATGAGGAAATAACTACAGT

AAATTCGCATTTTTTTGTTTTTGCAAGTGAGAAAACCCGTTTATTTCATTTCAGTAAGTA

AGATTCTTGGTTAAGGAAAAACCACCTTAAGGAAGATAGAGTTGCGCCATACTCAGCTCT

TGCTTATCCAAGCTTGACACTATAAATTTACTAAGTCGCGCTAGCATGAATGAAACATCT

TTCTTTTTTCATTTGCTAAAAAGTACATTTATTTAGCAAACTTGTTTAATATATATTTTT

TTATTGTATTTGTTGTAGGTTTTGTTGTAATTTACTATTTGAAATTGTTGTTGCTGACGA

TTCTCGTTTTTTTTTTTATATATATCCGCTGTATACTGATTTTAATTATTACATTTCTTA

TTGCAAGTACGTTTTTTCCAACGCTTTTAGTTGCTTTTAATAAGAGTTTATTTTTCAACT

TGTTTCAGATTCGTCATTTTTTCAAGTGCTTTCCAATGCAATTAGATTACATTATTACAT

TTATTAATAATAAAAATGAAATTCAAAAATGTCAATTGCATCTGTTCTCTTGTGTTTCTT

CTTGTTATACGTTTTTTCTCTAATAATCATAACAATTGAGTAAGTGCATTCGAATGTATT

TCACCATTACGATTCGGCAAACGAACCGAATCGATCGACCCTTCAACCACACCTTATTCA

ATACTTTTCCAGCTAAACTGTTGCTTAACTCAACGCGCGCATATGCATTTGCATGCCCTC

TGCAATCATTCACTCACACATTCAATTCAAAGTGTTACTGTATTCCCATTCGTGTTCCTT

TTTAAAAATTTCTTCGTATTTCAATTGCTTACGCCTTTTCGTATTTTATAATATAATTTA

AAATTTTCTTTTATTATTGTTGCAGGGGTTTGCCATTGTTTATTATTTCAATAAATATGA

GCCATTTATGTAATTTTCCACAGTGTGTGTGTGTCTTTCTTTATATAGGTATTCTTATAA

AAGAAAATACATAGGAGCAAATTTGAGAAATTCATTAGGATCCGTTTGGATGTTTAGTTT

TGTATGTGTGAAATCCTTTTGGACGAATGTGTACCTTTTGGTGTGGGAAGGGATAATATT

TCCTTATTTGTATAAATGTGTATATGATTTGTTTGAATGTTTACTGTTTTATAAAATTAG

TAATCTATACATAAATAATTGTAATATATAATTTCTATTTTTTTTTTCGCAGTTCCGCTT

CCATTATGTTTCCTTTTTATAATGTCTTCCGTATGCATTGTTAATCTTGCTTTGCTTTGC

ACTCTCTCAACCCTTATATTTTGCAGTTGCTGATTTCATCTTTTTGTTTACTTTTTCATA

CATTTCATTTATTTGATTTTTCGCTTTCAGCGGATATTCGCAATCAGTTCACTTTATTTA

ACTTCCCCACCAATCGGGACCGGAGTTGGAGTTAGCATGAGATGCCGAGTAACTGCCTCC

GTAAGAGCCACCACCTCCACCTCCACCTCCACTACCGCCATAGTAGCCACCACCAGAACT

GCCACCATTGCTGCGGTACGATCCACCGCCTCCACCTCCGCCGCTTCGCGGAGGACCACC

GCCACCACCGCTACGGGGGCCACCTCCACCACCGCCTCCGCCAGAAGTTTGACGGTAATC

ACGAGAGCCAAAACCGCCGCCGTAGCGTCCAACACCACCGCCTCCACGTCGCCGATTGCC

ACTATGCGTGCGGTCTGATGAGAGCATCTCTTCAAGAAAACCAGGAACTTCCTGCTTTGT

CTCGACGAGCAACTCCAGCAAATCACCACAAATATTGCGATTCTTCTCATTGAAAAACGA

TGTAGCAACACCGAGATTGCCCATACGTCCTGTACGACCAATGCGATGTACGTACTCCTC

CACATCTGAGGGCAGGTCAAAGTTAATAACGTGCTTCACATGGGGAATGTCCAAGCCACG

TGCTGCCACCGCAGTGGCAACCAAAATGGGACAATCGCCGGAACGGAAGCAACGTAAAGC

CTCCTCACGTTCTTTTTGTGTACGATCACCGTGAATACTTGTCACCGGATGATTGCATTG

GTAGAGGAACTCTTCCAGTGCGTCGGCGCCTTTTTTCGTTTCTACGAAGATAAGCGTTAA

ACTATCCTTTGAGTACTCGGCACCGGCGCGTATCGACGACAATAGATCAAGCAAATACGA

ACGCTTATCTTGTTCGTATACCCATAAAATAGTTTGGGTTATATTCTCCGACGTGGAACC

AACACGTCCCACAGCCAAGAATATATAATTGCTAAGAAAATCAGATGCCAGCTCTTGTAT

TTGTTTGGGGAAAGTAGCCGAGAACATCAAAGTTTGCCTCTGCCCAGTTGGTGGCATATT

AGATTGCTCTACAATGCGTCGGATTTGAGGTTCGAAACCCATGTCCAACATACGATCGGC

CTCATCCAATACCAAAAATCGTATGTTGTCCAAACCCACCTTTCCGCGTGTAATCATATC

CTCCAGACGACCCGGCGTAGCCACAATCAGATGGCAGCCACGATCCAATTCACGCATTTG

CTCACTTGTATTATTACCGCCGTAGAGTACAGCGGGACGCATACGAGAGCGATACGCAAA

CTTCTTGGCCTCCTCGAAAATCTGTGTGGCGAGCTCGCGCGTCGGTGCCAGTACTAGCCC

AAGCGGATACTGCTTACGGCGACTGTACTGGCGATTATTTTGCGGTGGCGGCGTCATGCC

ATGCTCATACATTTGATTGAGAATAGGCAGCAAAAAAGCTGCTGTCTTGCCCGACCCGGT

TTGTGCGCAGGCCATTAAATCGCGCCCATTTATTATAATTGGGATCGCATATTTCTGCAC

CGGTGTCGGTTTATCATATCTAGCCAACATCACGTTATTGCGTACGATCTCGGTCAGCTG

CACGTCATCAAACGATACGATGTTAGGCGGTACATTCTGTCCCGTCGCTTCCACGGGTAT

ATCCTCGTATTTGTCAAAGTTAATGCCGGTGTTGCCGACACCGAACAGTTCCGTCTCCAC

GCGTTCATCACGTGGTCCCAATTTAGTGTAGTCGATGTCGCCGCGGCGATCTTCCTTCCA

TCGTCCGCCATAGTTACGTTCTCCGCCGTTGCCACCACCACCTCCGCCTCTTTCGTTGCG

TTGCTGTGCACCGTCTTCTGGGCGACGCTCTGGCTCCTGCCAACGATCGTTGCGAGGTTG

TTGTGGTTGTGCTTGATCGTCGAAGGTTTCACTGTTACGATTACTGGTATTTCCAGTACC

GGCACCACCGCCCTGGCCTTGATTGCGTCCGCCACGATAGCCTCCGTTTTCCCGCCTATC

AAATGTTCGCGAATTTTGCGGGCCACGACCTCCACGATTCCAATCTTCTCCACCACGTCG

GGTTTCACCTTCAACTTCGAAACCGCCGCTGTTCTCCTCGTATCTGCCTCCACCACCACG

ACGCCCACCGCCGCCGTAACCACCACCAGAATTGTTATAACCACGACCACCGCCGCCTCT

GCGATACTCTCCACCGCCGCCGCCGCGCTGTTCTCTGCCTTCGAACTTTGACGATGGAGT

TCTCTCCTCGCGATTATCACTATTAGCGTTAGGTGCGCTGTTGCCACCTCCGCGTAGATG

TGGAGGGATATAAACGCCGGAATTAGATGAACTCTTCGTTGTTATGGGGCTGCTATTATT

AGCTTTGCCGCCATTCAAGTCCAGACCAGCAACCTGCTGCTCTAGACCTGTGCCATTTTG

GTTAATAGCATTACTCATATTACCCTCCAATCAGTTGCTGGTTTTTTCTTTCTTTCCGTT

TAGGAAAATCTCGAATGCTTGCTCACTTGCTTACACTGCTTCTATTGTATATAGAAATCG

AGAAGAAATAAAACCAAAATTGTCTTTTCTCTATCTATTAAGAGTTTCAGTTCAGTTAAG

GGACTTTTTTTTTTTTTTGGTGTATACGGCAGTATATTCTTTTTTTGCTATCTCATCAGT

TTTTTCACATGGGCTACAAGTTCTTGCAAATTTTCAGCACACGAACGAAAAACGCGTCGA

ACGTCTACTTCACTCGTTCAGTAAAAGAAAAAGAAAACTGGCCGAACTCAGCCGACTACC

GAAACGAAGGAAAGTACAATTGCAGAGATGCTTTTGTGGAATGTAATCTACAAACTTGCA

AAAAACAAAAGTCGAAAGTGCTAAACAGCTATAAACCG

**tBLASTn(First hit)**

Score = 892 bits (2305), Expect = 0.0, Method: Compositional matrix adjust.

Identities = 469/545 (86%), Positives = 486/545 (89%), Gaps = 8/545 (1%)

Frame = -3

Query 180 NLNEQTAEDGqaqqqqqPRNDRWQEPERPAGFDGSEGGQSAggnrsynnrgerggggyns 239

N N +T +D Q QQPRNDRWQEPER E G R G G Y

Sbjct 3402 NRNSETFDD--QAQPQQPRNDRWQEPERRP-----EDGAQQRNERGGGGGGNGGERNYGG 3244

Query 240 rWKEGGGSNVDYTKLGARDERLEVELFGVGNTGINFDKYEDIPVEATGQNVPPNITSFDD 299

RWKE ++DYTKLG RDER+E ELFGVGNTGINFDKYEDIPVEATGQNVPPNI SFDD

Sbjct 3243 RWKEDRRGDIDYTKLGPRDERVETELFGVGNTGINFDKYEDIPVEATGQNVPPNIVSFDD 3064

Query 300 VQLTEIIRNNVALARYDKPTPVQKHAIPIIINGRDLMACAQTGSGKTAAFLVPILNQMYE 359

VQLTEI+RNNV LARYDKPTPVQK+AIPIIINGRDLMACAQTGSGKTAAFL+PILNQMYE

Sbjct 3063 VQLTEIVRNNVMLARYDKPTPVQKYAIPIIINGRDLMACAQTGSGKTAAFLLPILNQMYE 2884

Query 360 LGHVPPPQSTRQYSRRKQYPLGLVLAPTRELATQIFEEAKKFAYRSRMRPAVLYGGNNTS 419

G PPPQ+ RQYSRRKQYPLGLVLAPTRELATQIFEEAKKFAYRSRMRPAVLYGGNNTS

Sbjct 2883 HGMTPPPQNNRQYSRRKQYPLGLVLAPTRELATQIFEEAKKFAYRSRMRPAVLYGGNNTS 2704

Query 420 EQMRELDRGCHLIVATPGRLEDMITRGKVGLENIRFLVLDEADRMLDMGFEPQIRRIVEQ 479

EQMRELDRGCHLIVATPGRLEDMITRGKVGL+NIRFLVLDEADRMLDMGFEPQIRRIVEQ

Sbjct 2703 EQMRELDRGCHLIVATPGRLEDMITRGKVGLDNIRFLVLDEADRMLDMGFEPQIRRIVEQ 2524

Query 480 LNMPPTGQRQTLMFSATFPKQIQELASDFLSNYIFLAVGRVGSTSENITQTILWVYEPDK 539

NMPPTGQRQTLMFSATFPKQIQELASDFLSNYIFLAVGRVGSTSENITQTILWVYE DK

Sbjct 2523 SNMPPTGQRQTLMFSATFPKQIQELASDFLSNYIFLAVGRVGSTSENITQTILWVYEQDK 2344

Query 540 RSYLLDLLSSIRDGPEYTKDSLTLIFVETKKGADSLEEFLYQCNHPVTSIHGDRTQKERE 599

RSYLLDLLSSIR G EY+KDSLTLIFVETKKGAD+LEEFLYQCNHPVTSIHGDRTQKERE

Sbjct 2343 RSYLLDLLSSIRAGAEYSKDSLTLIFVETKKGADALEEFLYQCNHPVTSIHGDRTQKERE 2164

Query 600 EALRCFRSGDCPILVATAVAARGLDIPHVKHVINFDLPSDVEEYVHRIGRTGRMGNLGVA 659

EALRCFRSGDCPILVATAVAARGLDIPHVKHVINFDLPSDVEEYVHRIGRTGRMGNLGVA

Sbjct 2163 EALRCFRSGDCPILVATAVAARGLDIPHVKHVINFDLPSDVEEYVHRIGRTGRMGNLGVA 1984

Query 660 TSFFNEKNRNICSDLLELLIETKQEIPSFMEDM-SSDrghggakragrggggrygggfgs 718

TSFFNEKNRNIC DLLELL+ETKQE+P F+E+M SSDR H G +R G GG GRYGGGFGS

Sbjct 1983 TSFFNEKNRNICGDLLELLVETKQEVPGFLEEMLSSDRTHSGNRRRGGGGVGRYGGGFGS 1804

Query 719 rDYRQ 723

RDYRQ

Sbjct 1803 RDYRQ 1789

**Conserved domains**

**ATP-dependent RNA or DNA helicase similar to DbpA, a DEAD-box RNA helicase involved in the assembly of the 50S ribosomal subunit**


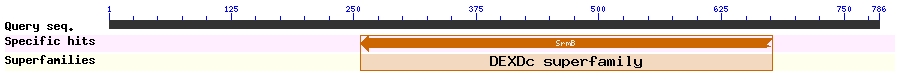


**PRP16**

**>TRINITY_DN32795_c0_g2_i1 len=1364**

TTATATACGTCATCGATTCGGGATACTGTAAACTGAAAGTCTACAATCCACGCATCGGTA

TGGATGCTCTACAAATCTATCCTATCTCGCAGGCGAACGCCAATCAACGTTCGGGTCGTG

CAGGGCGTACTGGTCCCGGACAGGCGTTTCGCCTGTATACACAACGACAATACAAAGAGG

ATCTACTGGCGCTGACTGTGCCCGAGATACAGCGTACTAACTTGGCGAATACGGTGTTGC

TGCTGAAGTCGCTGGGAGTGGTGGACCTGCTGCAATTTCACTTTATGGATCCGCCACCTC

AGGATAATATACTTAATTCCTTGTATCAGCTATGGATACTTGGCGCATTGGATCACACTG

GTGCGCTGACACCACTCGGTCGGCAAATGGCCGAATTTCCACTCGATCCGCCGCAATGTC

AAATGCTGATTGTCTCCTGCCAAATGGAGTGCAGCGCCGAAGTGCTCATCATAGTATCTA

TGCTTTCCGTTCCCTCCATTTTCTATCGACCCAAAGGACGCGAAGAAGAGGCAGATGGTG

TACGTGAGAAATTCCAAGTGCCGGAATCCGATCATCTAACCTACCTCAATGTATACCTGC

AATGGAAGCAAAATAATTACAATTCCAGTTGGTGCAATGAACATTTCATACACGTCAAGG

CAATGCGTAAAGTACGTGAAGTGCGTCAACAGCTCAAGGATATTATGGTACAACAAAAAC

TCAATGTAAAATCTTGCGGCACTGATTGGGATATTGTGCGCAAATGTATTTGCTCGGCGT

ATTTCTATCAGGCTGCACGTCTCAAAGGTATCGGCGAGTATGTGAACTTGCGTACGGGCA

TGCCTTGCCATCTGCATCCCACATCGGCGCTCTATGGCCTTGGCACAACACCCGACTACG

TTGTGTATCATGAACTTGTGATGACTGCCAAGGAGTATATGCAATGCGCAACCGCTGTCG

ATGGCTATTGGCTGGCAGAATTGGGTCCTATGTTCTTCTCTGTGAAAGAAACTGGGCGCA

GTGGGCGCGAGAAGAAGAAGCAAGCGGCTGAACATTTAAAGGAAATGGAGACACAAATGC

AGTTGGCGCAAGAACAGATGGAGGAACGCAAATTGCAGGCAGCACAACGCGAAGAGCAGA

TGACACCGAAGCAGGAAATTATAACGCCCGGCGGTGCTACACCCAGGCGTACGCCAGCGC

GCATTGGCTTGTAATTGCGGCAAGATCATTTCGATTTACATTCGTATGTACATACTTAAG

TGTGCTTGTAGCCTAATTCATGACTCTGTATTTTTACCAGCAACTAATGGAAATAAAGTG

GTTAACAGAAAAATGTTAAGCATTTTAGTTTTAATATAATATTT

**tBLASTn(First hit)**

Score = 737 bits (1903), Expect = 0.0, Method: Compositional matrix adjust.

Identities = 374/403 (93%), Positives = 388/403 (96%), Gaps = 0/403 (0%)

Frame = +3

Query 825 IYVIDSGYCKLKVYNPRIGMDALQIYPISQANANQRSGRAGRTGPGQAYRLYTQRQYKDE 884

IYVIDSGYCKLKVYNPRIGMDALQIYPISQANANQRSGRAGRTGPGQA+RLYTQRQYK++

Sbjct 3 IYVIDSGYCKLKVYNPRIGMDALQIYPISQANANQRSGRAGRTGPGQAFRLYTQRQYKED 182

Query 885 LLALTVPEIQRTNLANTvlllkslgvvdllQFHFMDPPPQDNILNSLYQLWILGALDHTG 944

LLALTVPEIQRTNLANTVLLLKSLGVVDLLQFHFMDPPPQDNILNSLYQLWILGALDHTG

Sbjct 183 LLALTVPEIQRTNLANTVLLLKSLGVVDLLQFHFMDPPPQDNILNSLYQLWILGALDHTG 362

Query 945 ALTTLGRQMAEFPLDPPQCQMLIVACGIGCSAEVLIIVSMLSVPSIFYRPKGREEEADGV 1004

ALT LGRQMAEFPLDPPQCQMLIV+C + CSAEVLIIVSMLSVPSIFYRPKGREEEADGV

Sbjct 363 ALTPLGRQMAEFPLDPPQCQMLIVSCQMECSAEVLIIVSMLSVPSIFYRPKGREEEADGV 542

Query 1005 REKFQVPESDHLTYLNVYQQWRQNNYSSSWCNEHFIHIKAMRKVREVRQQLKDIMTQQNL 1064

REKFQVPESDHLTYLNVY QW+QNNY+SSWCNEHFIH+KAMRKVREVRQQLKDIM QQ L

Sbjct 543 REKFQVPESDHLTYLNVYLQWKQNNYNSSWCNEHFIHVKAMRKVREVRQQLKDIMVQQKL 722

Query 1065 SVKSCGTDWDIVRKCICSAYFYQAARLKGIGEYVNLRTGMPCHLHPTSALYGLGTTPDYV 1124

+VKSCGTDWDIVRKCICSAYFYQAARLKGIGEYVNLRTGMPCHLHPTSALYGLGTTPDYV

Sbjct 723 NVKSCGTDWDIVRKCICSAYFYQAARLKGIGEYVNLRTGMPCHLHPTSALYGLGTTPDYV 902

Query 1125 VYHELIMTAKEYMQCATAVDGYWLAELGPMFFSVKESGRSGREKKKQAAEHLKEMEEQML 1184

VYHEL+MTAKEYMQCATAVDGYWLAELGPMFFSVKE+GRSGREKKKQAAEHLKEME QM

Sbjct 903 VYHELVMTAKEYMQCATAVDGYWLAELGPMFFSVKETGRSGREKKKQAAEHLKEMETQMQ 1082

Query 1185 KAQHEMeerkqqaaereeqlaakqeIATPGNATPRRTPARIGL 1227

AQ +MEERK QAA+REEQ+ KQEI TPG ATPRRTPARIGL

Sbjct 1083 LAQEQMEERKLQAAQREEQMTPKQEIITPGGATPRRTPARIGL 1211

**Conserved domains**
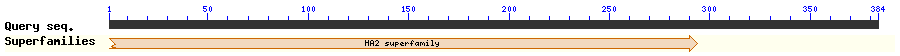


**Gemin3**

**>TRINITY_DN30190_c0_g1_i1 len=3368** CGATTTTTTAATTCATTTCAATTGCAAGAAAACAAAAATTTAATTCATTTAATGAAATGC

TTGAATAAAATAAACATTAAATTTAAGAGCTAATCTCATATTGTACTTATATGTAGCTAC

TAGAAAAAGAAAAGCTTGCATACATATAAAGCAATTACTTTTCATCTTTCAACTTTCCTG

CTTCAAGTGTCATTTGTACATAATCATTTATTTGTTTTAACTGTGTCCGGTACATTTCCA

TCCACAAAGCATATGCTGCTTCGTAGTTGCTGCCATAGGCATTACACTGCGACGAATGCG

TATCGTTTTGTGCTTCTCTGGCTTTTTGTGAATGCGTTTTTTTGCCATGACCCCTAGAAT

GCGTTGGCTTCATGCTTGCTTTCGCACTATTCTTTTCTTGTGATCTATTTTCCTCATTAT

CTTTGTTTGTTGCAGTTCCACTTTCCGTACTAACTGCGTTTGGTGTTTGTTCATTACAAT

GTTCGCTGTCATTTCGTTCTTTGTCGTCGCCATCATCTAATTCGTCATTTTCATCTCCAG

CTTCATCATTTTCTACTTCATCTTCCACTTTAACCTCATTATCGTCCTCTTCATCTTCCT

CTTCTTCTCCAGATTCCTCATATGTTTCCGCATATGGAAATAACAATTCTGGATCAACTT

CGTAACTTGAAGCTGTTTCAGTGTCTGGGGAAGAATAATACCCATTTTCAACATAGCGTT

CTGGTTCCGTATCGGATGTCACAATTCCAGAAGAAACGCTAGTAGTATTGTGCTCATCAA

AGCCACTACTACCTCCACGTTCTGAATTTGATCGCGAATTTAAGGAATCACTGCCACTAA

TAGAGGGAGAATGATCTTCGTCTTGGGAATGTACAATGGAATCGTTTAGAGCCGGAGAAT

TCGCAGGCGATGGTTGATGAGGCGGAATTTCAATGTGTGCACCAATGTTGACTGATAAAT

TATGATATTGTTGAGAATTATACGGCATACCCATAAGCCGTTGACAAGCGGGGATAACGT

CAGGTATTGCATTGTTCGTTTGATTATGAGCTGGTGAGGTAAAATGCTTTACTTTAGAAA

CTTCTAGGCTGTCAGATATAGATCGTGAATTTTTCAAATCACTAGTTGTCGTACTATTGA

CGAATTCTTCAATAGAATCCTGTACTTTTGAAGGAGTTGCAACCGATAATATAAAAGTTC

CTTCATCAATTTTGTTGGAACCAGTTTCTTGACTCAAACTACTAGAGTTTTCCTCGGTAT

TAAAATTAGCGTAGTCGGAGTATATATCATGTTTATTTTCGACTAACTTAGTTGGAACTG

GCTGGTCGATAAGCATAGATTTGTAAGTAGCTATTTTGTGGGATATGACCGGTATTTCCA

TATTTTTCTCTTCAGTCTCTTTCAAGGAACTATTTGAATTGGTAAGAACTGTTCTCTTGT

AGTCGTCAAACAAATCTAATTGGGGCTTCTTTTTAGTTTCACCTTCCTTAGCGTCCATTT

TGCTTTCGCTGCTATTCGGATCGACAAGGAATTCAAATAACGTAGGAATTGCGGCTTTAT

GCATTAGCACAGATTGGTATCGAGATATATCTTGCTGGCTACTACGAAGACTGTCTGCGG

CAATACTACTAGCATCATCAATAGTATTTGAAGACGGCAGTTGCTGTGAAGAGCCGTGTC

GTGAATCTATGGTTAACTTATGTGAAGGAGAGAGTGCTTGCTGATTATATTCCGGACGTT

CTAATAAGTTTTCCTGTATATTTAGTGCACTATCTTTAGAGTCTACGCGAAAGGTATTTG

TATCGATTGATGAGCTCGAGTTCAAAAAATTATCAGTTTGATTCTCTATATACTCTTGTG

TATCGGAAAATGAACTACGATCCTGCGAAGCTAATGGTGCTAATGGCTCGCATCCAAATT

GACCAAAGTATTCTTTATCTTTTTCTAAATTTGTAAAGTCCCAAAAGTCCACTTCGCCCT

CCTGAAATATTTGCTTCTGGGGGAATTTTAATACACTCATACCATTGCCAATTCGGGAAA

TAAGACGGGAAAGTATGCAACGTTCTTTCTCAGTAGCAACGAAATTAATAGCAATGCCAT

GTGAGCCAAAACGTCCGGCACGTCCAATACGATGCAAATAAGTAACCATATCTGTTGGTA

ATTCCAGATTGATAACCAAGTTCACATGCTCCGAGTCTACGCCGCGTGACATAAGATCAG

TCGTAATTATAACTCTTGATTTGAATTCTCGAAATTTGTGAAACATTTCCAGCCGCGTTT

TCTGGTCCTGTGCTCCAGAAATTAATTCACAAGGCCAACCCTCTTTTTCCAAATAATTGC

AATAAGAATTAGCTCGTGACTGTGAGCCAGCAAATATCAAACATTGTTTGAATGCTATGC

GTCCAAATATAAATCGAAGACCTTCTAACTTTGACTGCATTTCAAGTATACTCGTTTTTT

GATCAGGAAGTTCATAGACAAATTGTTTAATTCCAATCAGCAGAGTTGCTCTTTCCTCAG

TGGAAATCAGTAGGGGATTGCGCATTATCTTGGCCAACTCGACGTCTAAACCATCACAGA

ATGTAGCGCTGCAAGCTACGGTCTGTCGCTTTGCCGGCAGAGCATTTTGAATACGTCGCA

GATCTTGCCTAAAAGATTGAGTATACATTTTATCAGCTTCATCCAGCACCAGCAAACGCA

TCTTTGAGGTATTAAGAACATTATTTTGGATCAAATGTAGCAACCGGCCTGGAGTGCCCA

CAACTGCTTTAGCACCTTGCAACCGCTTTCTATCCTCAGCTACATCTAAACCACCGATTA

CACTGACAGCTCGGAACCCATTACAGCAACTGCCAATTTGATTTAGTACCATTTCGATTT

GAACGGCTATTTCACGTGTTGGTGCCACAATCAAAGATTGTGGTTCTGACAAATCCGCTC

GATAAGCTTCCAGTATTATCACGCAAAATATAAGCGTTTTCCCTGTTCCGGACTTCGACT

GTATAAGTAAATCTTGTCCAGATTTTCCCATCGGAATTGACATAGCTTGAATAGCTGTGG

GATATATAAATCCCGTTTTCTGCAAGCCCTTGCGTATCGATTCTGATAAAAGCATTTTAC

TAAAGGGTGATAATTCTTTAAGTTTTATGTCCTCTGTTCGTTCTTTGCCGTCCAGGCTGT

GCGCAACACATTCTCCTTCCATATTTTGCACCTCACACAAGTTTTCACTTTGTTTTAGAA

CGCTGACAAATAAAACCCGGCTACTAATTTATTCGTCAAACCAACATTTAGATGTGCCTT

GCGCCAAACATTGCAAATAAAATCAGCTGTTCACTGCATTGCATTTTGATACTATAGTTT

ACATTGGC

**tBLASTn(First hit)**

Score = 430 bits (1105), Expect = 3e-131, Method: Compositional matrix adjust.

Identities = 203/418 (49%), Positives = 291/418 (70%), Gaps = 5/418 (1%)

Frame = -2

Query 1 MERE-IAHSLAGGEERSSDVAPGQVKTFEELRLYRNLLNGLKRNNFVTPTKIQAAAIPMA 59

ME E +AHSL G+ER+ D+ ++ F ++ L ++ GL++ F+ PT IQA +IPM

Sbjct 3202 MEGECVAHSL-DGKERTEDIKLKELSPFSKMLLSESIRKGLQKTGFIYPTAIQAMSIPMG 3026

Query 60 LAKMDLIIQSKSGTGKTLIYVIAVVQSFNPNINQPHAMIVVPTRELAIQVQDTFFHLCKS 119

+ DL+IQSKSGTGKTLI+ + +++++ ++++P ++IV PTRE+A+Q++ +

Sbjct 3025 KSGQDLLIQSKSGTGKTLIFCVIILEAYRADLSEPQSLIVAPTREIAVQIEMVLNQIGSC 2846

Query 120 FRDFKCSAFIGGTDVAKDRKRMNESRVIIGTPGRLLHLYENRVFDVSKLRLLVLDEADQL 179

F+ + IGG DVA+DRKR+ ++ ++GTPGRLLHL +N V + SK+RLLVLDEAD++

Sbjct 2845 CNGFRAVSVIGGLDVAEDRKRLQGAKAVVGTPGRLLHLIQNNVLNTSKMRLLVLDEADKM 2666

Query 180 YQTKSLQHTVSKLIEAMPKNRQIIACSATYDQNLDERLAKVMDKPMLISNSERATVLLGI 239

Y T+S + + ++ A+P RQ +ACSAT+ LD LAK+M P+LIS ERAT+L+GI

Sbjct 2665 Y-TQSFRQDLRRIQNALPAKRQTVACSATFCDGLDVELAKIMRNPLLISTEERATLLIGI 2489

Query 240 RQFVYELPQQNNSVEEMRLKLQILGQIFNQLPYEQAIIFASSQMRADSYKNYLTASGIDC 299

+QFVYELP Q S+ EM+ KL+ L IF ++ ++Q +IFA SQ RA+SY NYL G C

Sbjct 2488 KQFVYELPDQKTSILEMQSKLEGLRFIFGRIAFKQCLIFAGSQSRANSYCNYLEKEGWPC 2309

Query 300 HLISGAMEQSERLHVFEGYRNFTMRILVATDLMARGVDSPHANLVINIDPPQDHVTYLHR 359

LISGA +Q RL +F +R F R+++ TDLM+RGVDS H NLVIN++ P D VTYLHR

Sbjct 2308 ELISGAQDQKTRLEMFHKFREFKSRVIITTDLMSRGVDSEHVNLVINLELPTDMVTYLHR 2129

Query 360 IGRAGRFGSKGIAITFIASKKESQRFREMSKKIATAWSVLEFPKEPM--PNEFNFWDF 415

IGRAGRFGS GIAI F+A++KE + +I SVL+FP++ + E +FWDF

Sbjct 2128 IGRAGRFGSHGIAINFVATEKERCILSRLISRIGNGMSVLKFPQKQIFQEGEVDFWDF 1955

**Conserved domains**

**ATP-dependent RNA or DNA helicase similar to DbpA, a DEAD-box RNA helicase involved in the assembly of the 50S ribosomal subunit**

**
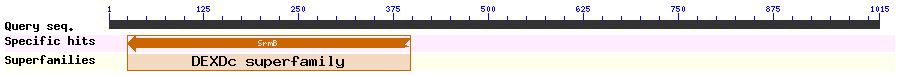
**

**Gawky**

**>TRINITY_DN27487_c0_g4_i19 len=9192**

TTAAAAAATAATAAATAAAATTAATTTTATTTTAACCATTGCTCGAATGGTGTTTTTTTT

TGTTTTTTTTTTTTGCATGGCCAACCAGTTTGCTTTTGGACGATTAAAATTTTATGTGCA

TCTTAAAACATAACTTGCTGACTTCCTAAAGAGAAAATTTGAACCACATTCTTAATACCT

TTCTCATTATCCTGAGTGATGTTGTACGTCACTATATAAGAAAAGTTTATCAAAAACTTG

CTGATCAAATCAATAAGTTGTATTAATGCAAAGCCATAGTAGGCATATTAAAGTTTCGGG

CAGCGAAAATCCGAAACTGTCCCATATGTGAAGAGTCCCCAAGATAGGAAAGAGCTCCTC

GTTTGCCCTTCTTTATTTGCCAATCCGATTTCGAATTTAAGCGATCACACAGTTGTACAG

GTAAATATAAAGAATAAATAATAAAAATAAAATATTGCACTTATAACAGTGTCAGTAATA

ACATTTGCAAAATTATAGCACGTGCAAATAATGACGAATAAATAACATTAAATTATTTTT

TTTAACAACTTCATCATAAACTTCTTGATCTCTTCCTGCTGTTAATAATGTTTCTATGTT

TTGGATTCCACTCCAATGCTTAATGTTTCTTAGCCAGGATAACTGTTTTCTGCCCATTTC

ACGTTTTCCTTATATCTTTCCTTAAAGTATTAATTGCAGCACACCACATTTGTCATTCCT

CATAATATGTCATAAGTATGCAGCTTTTCGTCTCTTAGTAGTAGTGAGCTGTTCTCGGTC

TTGCCGAATTCTTCGCATTATTTCCGCATTTGTCACTCTGTCTGTCCATCTTAGTTTTAG

GATTCTGCGAAAAATCCACATCTCAAATGCTTTCAGTTTGTTTAAATCAATGGTTTTTAT

TGTCTATATCTCCATAGCATAAAGGACATTTGCCAATACGGATTTTAAGTTTAATATTAA

AGTCATGATTACTTAATACATTTCAGTACTTATAAAATATTTCTCTCGAATGCTCTATTC

TAGCTCTTATTTCTTTAGAAGGATACCAGTTTTCAGTAACCCAGCAGCCTAAATATTTGA

AATTTTGCACTCGTTGTACATTTAAGTTGTCAACGGTTAAGTTTGCATTTACGTACATAC

ATATGTAAGTGTTTCTTCTGCTAACTATGATGTACTTCATTTTGTTAGCATTAATGTTCT

AGCCATAATTCTTGCTGCAGAACGCAATATTGCTGACCAGTGATTGTAGTCCACTCAGAC

TGTCTGCTAGAAGGAAAATGTTGTTAGCATATCTGATATTATTAATATTAATACCGTTTA

ATTTAATACCGACATCCGAATTTTGTAATGCTTAGTGGAAAATTCTTTCAGAGTAGATGT

TTAACAGTAAAGGTGAGAGAATTCACGCTTGTCTGACTCCTCTTCGAATAGAAATTGCGT

CAGTGCTCTCATTGCTTACGCTTACTTCTGCGGTTTGCTGCCAGTAGAGGTTCTTTGAAT

ATCTTTGGTATCAATATCCATTGTTTAATTGATAAGTTTATCGTGCTGCACAGTGTCGAA

TGCCTTTTCATAATCAATTAAACACATAAATACATCTTTTTGAACATCTCCGTAATTTTC

CACCAGAACGCATGTCTGCTTTGAAGCCGAATTGATTTTCTCCCATTGCTAATATACATT

TTTTGTATATTCTGTTATGAATAACTTTTAAAAGTTATTTTTAATGAGCGATTTTCCAAT

CTTGGGATCCTGTTGCTTTGTTGTCCTTAGACAATTTGATGGCATTTCTGATTTCTACCC

TTGCTGGCCCCGTCAATATTCATACACCGATCTATTGTTGTCCGAAAAAAGAAAATAGAT

GTTTATTCCAGATTTTTTCCTTATCGCTGGGCTCGAACACAATTTCATTCTCGTCATTTA

TTAGAATGGTTGGCATTGTTTTGTGGTATACAACGGCCGCCTCTTTAGGCTTCTTGTGAA

GGTTGGCAACACAGTAGTCAAAACTTAACAATTTTTTTATTCGAGGTCTTTGCAAAGCTG

ACAAAAGTGATTTCCAAACAGTTTTTTTAAACGTTGTAAATAAAATAGTGTTATTTGTTC

TCGAAATTTTTAAACATATTTCACATCTTTTTTTGGTAGTCATGTAAATTCAAATACCAT

AAGTAATAATATTCAGCTTTATCACTGAGTTACGAATACAACTCTCTGTTACGACTACGA

AAAAGTTGCTATCACTGATTCCGGATGTGTTCGCTGCACTTATTTATTACAGGAATATTG

CGGTCCTGTTACTTTTGATGATCCTGTTGCTGATACTCAGCTGATTAGCTGCTGGAAGGT

TGCAAAAGTTATTTCGCGCCGAACGTGTTGTTTCAAAATATGGAAGCTTATGCCAATATC

TACGTTTCATTTGAACTTAAATTTCGCCCCAAAACCAATAAACGAACAATAAAGTTCAAA

GTAGTATTTATTAAATATAAACTTACTTTTCTTCTTCCATTTCTACTCCGCAGTAAAAAC

TTTTATATTGCGCTTGATTAGTTAGTGAGGAGCATCAGATTGTCCAATTTTGCTGACAGA

ACGCGTAACGTTGGAATTGTTGCATAAATTTTGTAACAGATCGTGATGCCGTTTCATACA

AAGTAACACTGCCGCAATTATTACAGCTCGAATGTGAACACGAAAGTCCTGCTGAACTCA

AAAATCAAAATTTTGATGTTTGGACACCACCTGGCTCTCGATGTAAATAAACAATTTTGT

ACTGTTTCTTTAGCTTTATTTTGCAAGCGCTTCCCTTTTCTTTCCAATAAAATAAATTAT

ACTGGCCGCACTAATAATAATTTTTAATGAAAAAATGTAGCAAAACTTAAGAATACTTTA

AATAGTTCAGATTAAGATACGCCTTTAATCCTTTGGAAATAGATCTCACTTTGTATGAAA

AAATCTTGGATGTCAAAATATATAATTTTCGAAGTTTTAAATTAAAGATTTGTTAAAAAT

GTTCGATGTTTTTTTAAATGGGTGTAATAAAATTATTCTTTCTGAATTTTATTTTGAAAC

TTTTCTTCGACAACGGAAACGAAATTCTTTGAAAAATCTACGAAATGGCACTATGAATAA

ACTCAGTGATGGCGCTCTACGAATTTCGAATTTACGACGCGGCAGTTCGAACTCAGTAAT

GCCTTTTTCGTAGTATCTACGAAAACCGTACGACGATGGATTCAGTGATAGAGCAGATCA

AATTAAAAAAATAATAACAATCAATCTTTTATTTAAAAAAACGTCTCTTTTGACCAACGT

TATATAAACGCCAAGCAACAGTCTCTCCAGAAACAATTAACTAAAAAAAATGATGTAAAC

TCATCTTAGTTCGTAAGAACGATAAAATTCTGTAAGTTTAATTTGTGTATGAAAAGTATA

TTGAAAAATTTTAAATATCCAATATAATATACAGGAAAAGCAAGGCTTTTCAATCATTTA

TTATATTTTAAAGTCTTCGTAATGTTGTAGCCTTTAGATACATATGCACATATACATACA

CAAGAATGTTATCCAGAAAGTACTTTACATTGTGAACTAATTCCCCAATATATCACATAT

TATCTAATAGAATTGCATCTGTTCAGTGTGAAATTAGAAATTAATAGGATTATAAGCAAA

ATAATCAATGGTTGTCACTGCTTATAATATATGTATGTATATACACTTATACAATAAAAT

GGATCTAAGATCTGCATGTTTCATTACATATCTGAAGTGAAAATAGATTTCCGTTAGTGC

TAAGCTCGAGGATAAGACGTATTAGTTGCCCTGAAATACAAACTTGAGTGTCATAAAAAA

TTACCACGAATATAGGACTTCACAATTAATATGAGAACAAAATAAAGTAATAAGAATAAA

AACTAAATAATTTTAAGTTTGGAAATCTTCAAACAAACTGTTCTATTAAGGGCTGTAGCG

AGAGCAGAGCAATGTTTTCATTGATATTGCTGCTACGAAGTAGTTAATGCAAACCCTGCT

ATAAGCTGCAAGAACAATATAAACTATAAAAATGTATCAGTGCAATATACGTATATAAGT

AACGCCAGCTGTTCTAGTAGGACGTTACATAAAATAAATAAGTATATACAAAAATAATAT

AAACGACGAATGTATCACGACGACGACAACGGCAACGGCAACGGAATTACTAAATAAGTA

AACGCATTGTATCAACATCAAGCAATTCAATATCAAATTTACCGATAAGTTTATAAACAA

CGTCGCGAATTTTTATCGGGCCCACAATAGAGCAAAATTAAAGTGGGAAAACACAATGCT

AGCAGCTAATTTACCAGTATGCCAATACGCTGGAATGCCTGGACAAAGCAACAGTAATAA

CGGTGGGACCAGTAATAACAATAACAGTAGCACTAATAATACTAAGACAAATAACAGTAG

CAGCTGTAACTCTGGCACAAGTGGTCCGAATTCGAATAATAATAATGGTGGCATTAGCAC

GGGCAGTGTTGGATTAGTTGGTGGTGGTACTTCTGCAGCAGCTGCTAAAAATCAATTAGA

ACAATTGAACACAATGCGAGAGGCGCTTTTTTCTCAGGATGGTTGGGGTTGCCAGCACGT

TAATCAGGACACGAATTGGGAAGTGCCTAGTTCACCTGAGCCAACAAACAAAGATCCATC

TGGTGGACCAACGATGTGGAAACCTACTATAAATAATGGTACTGACCTATGGGAATTAAA

TTTACGCAACGGTGGTCAGCCACCAACTCAACCAGTTCAAAAGACACCATGGGGTCATAC

ACCATCGTCGAATTTAGGTGGAACATGGGGAGAAGACGATGACGGTGCGGACAGTAGCAG

CGTTTGGACTGGATCATCAAATAATCCATCAACTGCAGGTGTTGTGGGTGTAACTGGAGT

TAGTTCGACGGGTGTAGGTGCCAATGGTCCTGGAAATGGGCCGCAATGGGGTCAAAGCTC

TGTAGGTGTCGGCGTCGGTGCAGCTGCTAACGCTACAGGAACGCCATCTGTAAACGTAGG

CAGTGTGTCTGTCGTAGGTGGTCCTGCCGGTGGCCCTGGCGTAAATGTGCCTGGTAGTGT

AAATGCGGCGGCAGCCAATAATTCTGCTGGTAATAATTGGAGTGATTCACGTGAAATGAG

TAGTGGGGCTGTGAGCGGTGCAATAGCTATGCGTGGAGTTGACCCACGTGATCAAATGCG

AGGTACAGCTGCTGAGCCTCGGGAGTTACGCATGTTAGATCCTCGTGACCCCATACGTGG

TGATCCTCGAGGAATTTCGGGGCGTTTAAATGGCACGTCGGAAATGTGGGGACAACACCA

TTCTATAGCACATAATCAAATACCCGCACTAAATAAAATAGTGGGACCCGGGGTTAGCGT

AGGCTCCGGTGTGTCTACTGGTGCTGTAGTTACCGGTGGAGGTGTAGCTGGTTTAGGGGC

AGGTGGGGTCAATGCCATAAATGCGGGCGGTGTAAGTGGTAGTGTTAATGCCCATTGGGG

CGCATCTTCAGCGCTTGTTGGACCAAAAGATATTTCTTCAATGACAGGTAAAGTCACTGG

GTGGGAGGAGCCATCGCCTCCACCGCAGCGACGTAATATACCGAACTATGACGACGGAAC

ATCTTTATGGGGACAGCAGTCTCGAGTACCTAGTGGTTCTCATTGGAAAGATATTTCCGA

TCCAATGAATAGACATTTAACGCGAAATACAGTAGGTAACCAGAATACGCCAAATACGGT

AGCTGGCCTTGGTAGCGGATCTGGAAGTGGATTGGGAAACAACCAAGGGGTACTAAATGC

ACCAGTCGTTGGTGGTAATACGAACAATTCCATAAGCAGTGTTGGTCCTCCAGGACGGCT

TGGTTCTGGTGTAGGACCTGGTATTGCTGTCAATCAGCATAAACCTGATAATACCATGTG

GGTACATGCAAGCAATCCAAATGTGAATGCCCGTAATGCGGCATCATGGGGTGATGAAGC

AAACCACAATATGGGCCCGAACTCTGGTGTTAATTGGATGGATGATAAAACTAGTGCAGG

TATAGGACAATTGGGTGTCAGCGTTGGTGGCACTAACTCTTGGAATGATCCATCTGCATC

TTGGAACAAAAATCAAAATAAAATGGCAGGTGGTGCGACGGGATGGGGTACGGGTGGAGG

TAATGATGGTGCCGATCTGTCATCGGATTGGAGTGCTCACGGCGGCATTGTAGGAAAAAC

TCAACAAAAAATAGGAGGCGTTAATGTTAACATTACTAACTTGAATACAGATGTGATCAA

ACAGAGTAAACAATATCGTGTACTTGTGGAAAATGGCTTTAAAAAGGAGGATGTGGAACG

AGCTCTAATTAGCGCCAATATGAACATCGAAGAAGCCGCGGATTTACTCCGGGCTAATGC

CACAATGTCGATGGATTGGAGAAGACACGAAGACCCCCTTGGCTCATATGCTGATCATCC

TAATCCTGGTGGTAGCTTCCCAGGACGCTACCCACCAACGGCCACTCAGCCCACTATGCC

ATTCCCTCCGAATATCTTAAACAATATGAGCGGTAACACTGTTAGTGCCAGCGGCAATAA

TTCAAATCTAGCAGCGCTCAATTCGATACAGCCACTTCCAGTGCAAAAGTATCTAAATTC

AGCTCCACACAATGTTGCAAGTGGACCGCAGACCATGAATCCTGCTGTGGCTTTCGGACA

GGGAGTTGCTAATGTTACTGCGGCTGTGAATGCAGCTTCAAATAGTACCAGCCAGCCATC

CACTCAACAGTTACGCGTTCTTGTACAGCAAATCCAGTTAGCTGTTCATAGTGGTTACCT

ATCCAGCCAAATTTTATCAGAGCCTTTGCCATCCTCAATGCTTGTACTTTTATATCAATT

ATTAACAAATATTAAACACCTTCAAGCGGCACAGCAGTCTTTGACACGTGGTGGCAACAA

TGCCAATAATTCACAAATAAGTTATGCTATTGCCAAATATAAACAGCAAATCCAAAATTT

ACAAAATCAAATAAACGCACAACAGGCGTTCATACTGAAACAGAAGCAGCAGTCCATGCC

GCAAAATGCGCAGCATTCTGCAGCACATGTCAACAGTTCGAACCTAGAATATATTAGAGG

GCAGCATGATGCCATAAATGCTTTACAGAGCAACTTTTCAGAAATGAACTTGGCTAAGCA

TAGTGGCTATCCAACTGGTCCAAATTCGCAGTCAAAACTTATAAATCAGTGGAAACTTCC

GGAAAAGGATATAACGTCTGAAAGTACAGATTTTTCTCGGGCACCGGGAGCATCCAAACA

AAACTTAAACGCTTCTGGGAATACTATGGGCCCCCTAGGTCTACAAAATGATGGCACTTG

GTCGACTGGGCGAAGTATTGGCGACGGTTGGCCAGACTCATCTACAGAAACTGAGAATAA

AGACTGGTCCGTTGCACAACCTACGCCTGCTGCGACGTACACTGATTTAGTTCAGGAGTT

TGAACCAGGAAAACCGTGGAAGGGCTCGCAGATTAAAAGTATAGAAGACGATCCTAGTAT

AACTCCTGGCAGTGTAGCTAGATCTCCATTGTCTATTAATTCGACGCCAAAAGAAGCTGA

TATTTTTGCGAACCCTAGCAAAAGTTCACCGACTGATTTAACACCATTAAGCTTATCCTC

ATCGACATGGAGCTTTAATCCATCATCAAGCAATCAAAACTTTCAAAGTTGGTCCGATAG

TCCGCAACAATGCACCCCCTCGGAATTATGGGCCAGTTCAATGAACAAGACATCGCGGGG

TCCACCACCCGGTTTGGGTTCAGGCAAAGGAGCAGGCGTAGCAGGAACTGGCGTACAATC

CGTTACAAATTCCTCCTCCGTAGTTGCTGGCAGTTCAAATGGATGGATCGTTACTGGTGG

CCGTGGAGTTCCCAATAACAATTCTGGTTGGACTACGTCGAATTCAGGGTGGAATTCTAC

ATGGTTACTACTCAAAAATTTAACCGCACAGATTGATGGCTCAACATTACGTACCTTATG

TATGCAGCATGGCCCACTTGCTAATTTTCACTTATATTTAAATCAAGGAATTGCCTTATG

TAAATATGCAACACGTGAGGAAGCAAACAAAGCCCAAATGACATTGAATAACTGTGTTCT

CGGTAGCACTACAATTTGTGCAGAGTCACCGAGTGAAAATGAAGTTCAAAGTATTTTACA

GCATTTGCCTCAGACGTCTAACAGTGTTTCCGGGTCTGGTTTAACGGGAAATGGCGGTAA

TTCGAGCAACCCTACGTCGAATAGTGGTGGGAGTGTTGCAGGAGGAAATGGTTCTCTATC

TTCTAACAGTGGTAATAATAATGGAGGCCCCTGCAATAATGTTGGTGGTGTGAGTGGTGG

CAATGGTAACTGCAGCATGGCTAACCATTCGGTGGTGAGCGGCTCGGGCGGTAATGGATG

CGGAAGTAATGCGATGAATAATGGTAGCACTGGAGGCAATGGCAACAGTGGAAATAATCA

AAGTCAACCCAGTTCTGGTGCTTGCAGTTCGAACGGTGGTAAAAGTGGAAATAGTAATAT

TTCTAGCGGAAATAGCAGTTCAAATGCAACATCAAATAGTGTTGTCGGAAATAATAGCGT

TGTGGCTGCCCCTGCATGGCGTCAAACACAAAGTCAGCCAAGACCATCTGGTAGGGATGA

ATATGATTATATTTCGAAATTTGTTTGTTCTATTGTGGATGACTAAAATTACACTTTGAT

CAATAAGGCGTTCAAAATTTGTATTTCATATAATCTTGACTAATCTTAACTACTCGACAA

AAAATCAAGTTACAACAGAATTACAAGGTATACAGATTGACTGATAGACGAATGGACTTA

GAATTTTATATGTAATTTTTTCTTGTGGTTCATAGGATTGTATTTTCAATCAAATTAAAC

GAAAATTACTTTAAATATAAAATTAGTTATGGTTAAATGCCACACTTATGCTACAAAACA

TTTATTTTAAAAATTAATACGAATTATGTGAAGTGTAGAAATAATTTATCACTTAATTTG

CCGAAATGGTTCATTGAAGACAGCATATTTGTTATTGAGCCTATAAAATCAAGACAAATG

CAAAATAAAAAATAATGAAAAGCAAAGAACTGTAATAAATTGTTATAATTTTAGATTTAA

AATTAAGACTAC

**tBLASTn(First hit)**

Score = 803 bits (2074), Expect = 0.0, Method: Compositional matrix adjust.

Identities = 602/1097 (55%), Positives = 730/1097 (67%), Gaps = 132/1097 (12%)

Frame = +2

Query 174 WGDPREIRPLGVGGSMDIRNVEHRGGNGSGATSSdprdirmidprdpirgdprgiSGRLN 233

W D RE+ V G++ +R V+ R + T+++PR++RM+DPRDPIRGDPRGISGRLN

Sbjct 5138 WSDSREMSSGAVSGAIAMRGVDPR--DQMRGTAAEPRELRMLDPRDPIRGDPRGISGRLN 5311

Query 234 GTSEMWGHHPQMSHNQLQGINKMVG---------Qsvatastsvgtsgsgigpggpgpst 284

GTSEMWG H ++HNQ+ +NK+VG + A + G

Sbjct 5312 GTSEMWGQHHSIAHNQIPALNKIVGPGVSVGSGVSTGAVVTGGGVAGLGAGGVNAINAGG 5491

Query 285 vsgNIPTQWGPAQPVSVGVSGPKD---MSKQISGWEEPSPPPQRRSIPNYDDGTSLWGQQ 341

VSG++ WG S + GPKD M+ +++GWEEPSPPPQRR+IPNYDDGTSLWGQQ

Sbjct 5492 VSGSVNAHWG----ASSALVGPKDISSMTGKVTGWEEPSPPPQRRNIPNYDDGTSLWGQQ 5659

Query 342 TRVPAASGHWKDMTDSIGRSSHLMRGQSQTGGIGIAGVGNSNVP---------------- 385

+RVP+ S HWKD++D + R HL R VGN N P

Sbjct 5660 SRVPSGS-HWKDISDPMNR--HLTRNT----------VGNQNTPNTVAGLGSGSGSGLGN 5800

Query 386 ---------VGANPSNPISSVVGPQARI-----PSVGGVQHKPDGGAMWVHSGN--VGGR 429

VG N +N ISSV GP R+ P + QHKPD MWVH+ N V R

Sbjct 5801 NQGVLNAPVVGGNTNNSISSV-GPPGRLGSGVGPGIAVNQHKPDN-TMWVHASNPNVNAR 5974

Query 430 NNVAAVTTWGDDTHSVNVGAPSSGSVSSNNWVDDKSNSTLAQ--------NSWSDPAPVG 481

N +WGD+ + N+G P+SG NW+DDK+++ + Q NSW+DP+

Sbjct 5975 N----AASWGDEANH-NMG-PNSGV----NWMDDKTSAGIGQLGVSVGGTNSWNDPS--- 6115

Query 482 VSWGNKQSKPPSNSASSGWSTAAGVVDGVDLGSEWNTHGGIIGKSqqqqKLAGLNVGMVN 541

SW Q+K + G DG DL S+W+ HGGI+GK+QQ K+ G+NV + N

Sbjct 6116 ASWNKNQNKMAGGATGW---GTGGGNDGADLSSDWSAHGGIVGKTQQ--KIGGVNVNITN 6280

Query 542 VINAEIIKQSKQYRILVENGFKKEDVERALVIANMNIEEAADMLRANSSLSMDGWRRHDE 601

+N ++IKQSKQYR+LVENGFKKEDVERAL+ ANMNIEEAAD+LRAN+++SMD WRRH++

Sbjct 6281 -LNTDVIKQSKQYRVLVENGFKKEDVERALISANMNIEEAADLLRANATMSMD-WRRHED 6454

Query 602 SLGSYADHNSSTSSGGFAGRYPVNSGQPSMSFPHNNLmnnmggtavtggnnntnmta--- 658

LGSYADH G F GRYP + QP+M FP N L N G T GNN+

Sbjct 6455 PLGSYADH--PNPGGSFPGRYPPTATQPTMPFPPNILNNMSGNTVSASGNNSNLAALNSI 6628

Query 659 --LQVQKYLNQGQHGVAVGPQAVGNSSAVSVGFGQntsnaavagaasvniaantNNQPSG 716

L VQKYLN H VA GPQ + +V FGQ +N A A+ N +QPS

Sbjct 6629 QPLPVQKYLNSAPHNVASGPQTMN----PAVAFGQGVANVTAAVNAASNST----SQPST 6784

Query 717 QQIRMLGQQIQLAIHSGFIssqiltqpltqttlnllnqllsnIKHLQAAQQSLTRGGN-V 775

QQ+R+L QQIQLA+HSG++SSQIL++PL + L LL QLL+NIKHLQAAQQSLTRGGN

Sbjct 6785 QQLRVLVQQIQLAVHSGYLSSQILSEPLPSSMLVLLYQLLTNIKHLQAAQQSLTRGGNNA 6964

Query 776 NPMAVNVAISKYKqqiqnlqnqinaqqaVYVKqqnmqptsqqqqpqqqqlpSVHLSNSGN 835

N ++ AI+KYKQQIQNLQNQINAQQA +KQ+ Q + H+++S

Sbjct 6965 NNSQISYAIAKYKQQIQNLQNQINAQQAFILKQKQQSMPQNAQHS------AAHVNSSNL 7126

Query 836 DYLRG-HDAINNLQSNFSELNINKPSGYQGASNQQSRL-NQWKLPVLDKEINSDSTEFSR 893

+Y+RG HDAIN LQSNFSE+N+ K SGY N QS+L NQWKLP +K+I S+ST+FSR

Sbjct 7127 EYIRGQHDAINALQSNFSEMNLAKHSGYPTGPNSQSKLINQWKLP--EKDITSESTDFSR 7300

Query 894 APGATKQNLTANTSNINSLGLQNDSTWSTGRSIGDGWPDPSSDNENKDWSVAQPTSAATA 953

APGA+KQNL A+ + + LGLQND TWSTGRSIGDGWPD S++ ENKDWSVAQPT AAT

Sbjct 7301 APGASKQNLNASGNTMGPLGLQNDGTWSTGRSIGDGWPDSSTETENKDWSVAQPTPAAT- 7477

Query 954 YTDLVQEFEPGKPWKGSQIKSIEDDPSITPGSVARSPLSINSTPKDADIFANTGKNsptd 1013

YTDLVQEFEPGKPWKGSQIKSIEDDPSITPGSVARSPLSINSTPK+ADIFAN K+SPTD

Sbjct 7478 YTDLVQEFEPGKPWKGSQIKSIEDDPSITPGSVARSPLSINSTPKEADIFANPSKSSPTD 7657

Query 1014 lpplslssstwsFNP---NQNYPSHSWSDNSQQCTATSELWTSPLNKSSSRGPPPGL--- 1067

L PLSLSSSTWSFNP NQN+ SWSD+ QQCT SELW S +NK+S RGPPPGL

Sbjct 7658 LTPLSLSSSTWSFNPSSSNQNF--QSWSDSPQQCTP-SELWASSMNKTS-RGPPPGLGSG 7825

Query 1068 --tansnksansnastpttITGGANGWLQPRSGGVQttntnwtggnttwgsswLLLKNLT 1125

+ S ++ + + G +NGW+ GV N+ WT N+ W S+WLLLKNLT

Sbjct 7826 KGAGVAGTGVQSVTNSSSVVAGSSNGWIVTGGRGVPNNNSGWTTSNSGWNSTWLLLKNLT 8005

Query 1126 AQIDGPTLRTLCMQHGPLVSFHPYLNQGIALCKYTTREEANKAQMALNNCVLANTTIFAE 1185

AQIDG TLRTLCMQHGPL +FH YLNQGIALCKY TREEANKAQM LNNCVL +TTI AE

Sbjct 8006 AQIDGSTLRTLCMQHGPLANFHLYLNQGIALCKYATREEANKAQMTLNNCVLGSTTICAE 8185

Query 1186 SPSENEVQSIMQHLPQT 1202

SPSENEVQSI+QHLPQT

Sbjct 8186 SPSENEVQSILQHLPQT 8236

**Conserved domains**

**protein containing domains Ago_hook, UBA_like_SF, M_domain, and RRM_GW182_like**


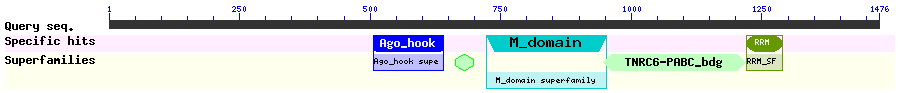


**Staufen**

**>TRINITY_DN33993_c3_g1_i10 len=6660**

ATGGGAAATTTGGAAAAAACATAGCTAATAATTTAAGGAAAAAATTAAACATTTATTTCA

ATTAAATGTATATATTTATGTACGTATATATTGAAAGAAAGTAAACTTTCGACAACAAAT

ACTTAATACTAAGTAAAGAGAAACATCGGACAACCCATAATAATTTCGGCGGATAAGCAA

CAATTTTAAAATTTTCAACATTTTAAATTTTCTAAAGCATTACATAAGGCGCATTGCAAA

CTACCATAGTTTATCTACCGCTGTTGTGAGATATACTAATTAAGTTAACAATTTCGCGAC

ATGGGTTTTTCAATTGACATGTAAAATCACCATTTCACCATTTATCGAAAATGCAAAGAG

GAAAGAAAGAAAGAAAAAGAAAAATAAGAGGTTAACGCAACTTATTATGAATTGTCCGTG

TGTGCAGATGAAGAAATAAATAATATGTATTATGCAGTAGAAATACAACTAAGTACACAC

CTCCAAGTCGTCAGTTTCAAGCAGCGAAAAATCGCAAGTTCGCCTCATTGAAATTGGCAA

CATTGAAGCAGATAGTAGCGCTGTTGTACGAGTACTTAGCGAGTTTTAAATGATCAGCCA

GTGTCATTTAATTTATAAATAACCTATAGCGTATTATTTTCGTTTCAATCGTTGTTGTTG

GTTATCAATCGCTATTGTTTTTTGTTTTTTGCAAATAGTTAAATTAACAGTTATGCAGGC

ATGCAAGTATGTATGTATCTATGGATGTGAATGTGTTGTTACGTCTATAAGCAGTTTATA

AATTAAATTTACGACATTCATGCAGCTCTTTATTTATTTCTTAAAAAGTATTTAATTATA

CTTTTTCATTATTACGACGAGTTCCATCATCGAGAAAAAAGTATGTATGTGTGTGTTTGC

ATTTGTGCAACACTAACAAATAAACATAAGTTTTATATATATATTTTTTTGTTTTTGTTT

CTAGTAGTAATATGCTTTTAGAATCAACACAATCAGCAAATGTGCAGTTATGCCATTCAT

AGCCAAATTGCTGTTGTATTGATTTTAATTTTTTATTTTTATTATTATTATTGTTTTCTT

TTGTTGCAAGTTGTATTTAATGAATTTCGCATTTATCTAAAAATAAATTAAAAATTTCAA

ATTATCACCAATTGCCACGTCCATACTCGTATAATACATCGCACAAGTATGCACATATAT

ACGAGTATATATACACATGTACGTGCAATCTGCTCAACGGTTGCGCATTTGGATACAACA

ACACCCCAATTAATTTCAAATTTTTATATTTATTTTTAATATACAATCAAAATACGACCA

CATGTTACAATTTCTTTTATGATGATTCTTAATGTATGTATGTGTGTGTACTGCATATTT

TCGCATTTTTTATAATCCAATGACGTTGCTTACACTAAATTACTTAATATTTTTGTATTT

GTAATTTATTTTCTTAACCCAAGGAACTTTCGAAATAAAGTAGAAATGTTTTCAAATTAC

AAAATTGCCTGCGAAACCACGATTTGATTTCAAGTCACAATATATATTTTTTTACACTTC

ACTAGTGGTTCAGCAATTAAATTTAAGCTGCATATTTGCAGGCGCACATCATTTGTGTAA

TATTTCAGTTCAATCTATGTAATTTGAAAATAGTTTTTACTATTGCAACATGACAATAAA

TGAGCGATTGAAATCGGTTGAACGGTAATAAATCGAACAGAATTTTCACTGTTACTCCAG

ACACGTTTCTTCCATACTTTCACCGCAACAGAGGCGATCAAAATACAGAGCGCGCTTTTC

AAAACACTGACTGTGCTGGAGGTACATTTACGTCAAGATACGGTCTTAGTTTTTTAGCGT

TCATTCAATGCTTTGCTGCAATACATTTTTTATAATTTTCATATAGGAAACAAAAATATG

TACATACATATTTGTTTAAATCTGTTAACCCAGTGAACACAAATAATACATGCTTTCGCG

TTTCAAACAGTTCAGCAGGGGTAATTTTGAATATTCGAAAATTGGGCAAACGGTAAATGA

ATTCAAAAGTAATAATTTTTATAAACTCATTCCTCTACGTTATAGAAAAAATCGCATACG

AATGTTAAGTCTTTTCATAAGAGACACTAGAGAAAATAGAGACCGAATTTTCTACGGTAT

CGAATCTTTGAGATATCGATATCCGATGAGAGAGTATATCTATCGTGCGAAGAGATGGCA

GATAACCTAAAATTGAATTCTAATCTCTCCAGAAACATGAACTCTGTTAAAGTGGCGTAA

GAATCGAGTTTTCTGCGGTATCAAAGGAATTCCATTAAGGGAAGAGTTGAAAAGTACTTA

AGGGAGGCTTCTGTCGATATTAAATTATTTTTTCTTACTAGGGGGATAAAATCTGAAGAG

ATAGTAGATAAGCAAAAATAAATTTTAATCTCTCTAGAAACGTCATCTCTTCTGAAGTGG

CACTAGGAAGCATGAGAATTGAGTTTTCTACTTTATCAAATCCATATCGATATACATATG

TATCGCTTAAGGGCATGTCTCCTCTGAAGAGATAGTAGCGAATGTAGAAATTGATGCGGT

ATCGAACTTTGATAGATATATATCGGTGATCGAGCATATTAAGTGAAACTATGATGGGTA

AGAATTAATTTTTCTACAGTGTGTACTTTTATCTCTAATAAAGGAATATCTCTTCTGAAG

GGACAATACAGAACAGCGACTCTCTACTACTCACTAATCAAAATTCACAGATTTTCGGTA

AATGTATGCCGCTTGTTGTTATGTCCTTGTATTCTGTTCGGGTTCGGTTCAGAAAAAAAT

TTTGCTTTTCTATTAAATTATAATGCTTTCGTATTAAACATTTTTTTGATCGAACGTAAA

TAAACTGCTTAATTTTTCACTAAAACTGTTTTGTGATCACAAAAAAATAAGTAATTCTTT

GAGACTTGTTATTAGGGTTACAATAAATTTACAAAATATCAAATCATTCTTTTTTTTTAA

CTAATATTTTTTAACAAAACTCACTTATTGCTAACCGACATAAGTTTTAGCTTTTTGCTC

AACTTTATGGAAACAAATTTGGTTCAGATAATGAGCCTAACACATTTAACTAAGCAGATA

TATAACAAAATAATTATATAGTAAGTAAATATTTTTTTCAACGCAACTAGTATATAAAAA

AAATGTATAAATATAGCAGCATTGTTCATGTTTTCAAATTGTTTGTAAAATTTGATATAA

CTTTTTTGTTGCACATTCGTTCGCAATTAATTTTCATTCTTTTTTCTATATTTGGGATTT

TCATGAAATAATAATTTGTTTTGAAATATAATTTTACAGCAACTAACTTTCAATTCACTT

CCTGCTGTTCGTGTAATGACTCCCCATATCTTAGGTATATAGACTTTTTTCGCGCCGAAT

TCAATTTGAATTTATATAGTTCTTATTTTATATACAGTTTTATTCAAATTTCTAGTTTTT

GTATGACAAATTTATTTTGTAGCATTATTCAAACCTAATTCACTAAGAATTTTCAATGCA

TTTTTGGCGGCATCATTTTGAGATTCTTCTGCGCTGGAACCCACGCCATGACAAATTTGT

GGTGGATTTGAAGAGAGCGTTACGATAGTTAAGAATTCACTATGATTTCCTTTTGGATAA

TCGGAAAAGTTCACCTCGAATCCCAACAACTTTGCCAGATAAAGCAGCTGGTCTTTCATG

TGCACACCTGTTTGTTTTGATGCGCTATTGCTAGATGATGTTGTGTCGCTGTTGGTTGTA

CTGCTGGCAGTATTTGTAATATTGCTGCTCTCAGTGTGAGCCGCAGCAGTCGCAGCCATG

GATGGTGGTGTAGCGCTCGCTGCGGTTCCTACATCAATGCCTTTCACAATATCGCTTGCA

TTGCTTTTAACCGCAGTGGGTTGCACAGTTGGCACTGCTGTGCCCACAACTAACTCAGCG

CGCTCAATTTCCCTATCCTCGTCCGGGAGGCAGTTTGCACTGGGTACATATTCCTTTTTT

TTGGTAAAATGTTTTTTATTATGACGCAAAATCAAAATGCCGGGTACTTGCCCGGCTGTT

GTGGACACGAGCGGCAAATCCAAATGTGCCTGCACAGGTGCAGCTGTTGTAGCAGCAGCG

TTTGCTACTTCAGCAATTGGCTGCAATACTGTTGTTGTTGTTGTCTGCTCTTGACTTGCT

GTTACCGTAGCGACTACTGTTGTTGCAGACGCAAATTGCTCTTTAACAGCAGGCTCGGTA

TTAGAGGATGCTAATACTGTGTTGTTCTCAGCGCTAATGCTGCCACTTTCGCCCATAGCT

GTCAGCAAATTTTGTGCTGCATTACGCTTCGCTAGCTTCTTGCTGTTGCCGGTGCCACGT

GCCACCATGCCATGTGCTGTCACCTCCATAATGAATTCGCGCCGACGTGAGTTCTCATTT

CCATTTTTCGCTATCAAATCAAATATGGGCTCTTTTTCCTTGCGAGTTTGTTGCAATTGT

ATGAGTCGCGTAATGGGATTTTCCGCATCGGCATCTACTTCATTTTTCTCTTTAATCGAA

CCACTCCCACGCTTGCGTCGTTCCCCAACGGCGCTACCAGCTTTTCCACTAACACCTCCA

GCCGCGTCAGCACTTGCAGACCCTTTGTTGGGTGTTTTTACTTTGATACGTCTAACGGGC

GACTTGGTAGGCGACATTGGTGGCAGTTTTTTTAGCTCTTCGAGCATTTTTTCGGCGGCA

CGTTTTTTCGACGTTTTCTTTCCATTTCCCTCGCCCTCCGTTACAATGGAGCCCACGACG

CAAGCTGTTACAAACTTTTTCATATGTGCTGGCCCTTCCTCGCGCAGCACCTTAAAATGC

ACCGTCAAGCTTCGTTGTATTCCGATCTCGTGCACCAACGAAATAGGCGATTTACTATCG

CTTTCCTCGATGCTGTTGTTGAGTTCTTCACTTATCTGCGTTTTAGCCTGTGTTTTCAGC

ACCTGCAATGCACGCGCCGCGGCATCGTGCTTGGCTTGTTGTAGTGTACGGCCTAAACCA

ACAAATTTCTGCTTGCCTACGACTAGTGTCACTTTGGTCAATGCAGCAGATATGCCGTAA

CGGTGGGGAAGCGGCCCATGATGGGGATGTGGCATTGGCGGTGGTGGAACCATGCCATTA

TGTGGAGCACCACCCATATGCGGTGGCGCCATCAAGGGATACCGTTGCTGTTGGTTATAT

TTGGGCATGAAGGCGCGCTGAGCTGGCGTAAGGTAGCGTGGCCGCATGCCCGGTCCGCCC

GCAGTATGGCCGTGCATCGGTGGCGGGGGCGGTGCGGCATAGGGGCCATTTCGACGCACA

AATGCAGCCGGATCCATCATACGTGCATGCGGTGGCGGTGGATGTTGCGACAGCATATTG

CCGCCAGCTACGCCGGGCGGGAACGCATGTGGTGGTGGCAGTGGTATGCCATATCGCGGC

GGCATCATAGCGTCGGGTGGCGGTCCGATTCTATCGGGTGAGGTGACTTGACGTGGATTT

AAGAGGTAATAAGTCTGTTCGCCCAACTTCATAGCCAGTGCATTTAACTCAACTGTGGGC

GTGATATTGGAACGCGATGAATGACCCTCACCTTCGGCATTGCGACGAACCACTTTCGGC

ACTGGGTGCTTGTATTTGGTCTTTTCAATAGCCTCTGCAGCTGCTAAATGTTGGGCTTTC

TTTATTTTGAAACCCTCAGCGGAATACTCCTCCTCGCCCAATTTCAAAGTGACAGTGAAC

CGTTTGCAATGCGCCGGACCTTTCTCACTGGTTAGGCGATATTGATGTGTAATCTTGTTG

AATCGCGCCAATTCGTTCACAAGACACATTGGTGTCTTCTCTTTAGAATTTTTTGACCCC

GCGCTCTCCTCGTTAGACAATATATTACTATTGTTGTTATTTGCTGGATTGTTGCTAACC

ACTTCATTGACAGTAACATTTGTACTTTGTGTCCTGCTTACCCCATTTTCCTTATTAGCA

TCGTTGAAGGAAGCATCTGTTTGGCTTGTGCTTTGCACAATTTTCAAACTCTCCTGAGCT

GCCGCGGCCGCAGCAACAACTACACCATCGGTTACAGCGGCGGCTACGTTGCTAGCAACA

TGAGCTTTTGCCGGCGTCTCTGTAACGGCAACTGCATTGCTGCTGCCGATATTGTTATTA

CTACTACTCTTCGATACGTTCTTAGTCTCTTCTATGCTCACTAAAGGACCTACAGTAGGC

ACCTCAATGTTGTTCGCTGCAGCGCTGCTGTGCAGACGCGCTTTTAGCACGCGATTAGGA

TTTTGTAAAATAGCTTTTGGTGGCATTCCACCACCACCACCAGTGCCAGTACTACCACCG

CCAGCGTTATTGCTATACTTCGACTTATGTATATTATTGTTGTTGCTTTGAGCAGTCCCG

GCAGTGTAGTAGTTTAATGCTGTAGGCGGTTGTTGTGGATGTTGAGTCGGTGTCGGCGGC

GGTGGATGGCCGCTGATAACATTATTGGCAATATTCAAAGTTGTTTGTAGTTGTTGCTGC

TGCTGTTGTTGTAGCAGCTTACTAAGTCTAGTCGTACTAGCCGCTAGTATTTAGTAGCAA

TGCTTTATTGCTCGTAAATTTATATGTGTATGTATATGTTTGTAAATGTGTGTGTGTGTG

**tBLASTn(First hit)**

Score = 523 bits (1347), Expect = 2e-159, Method: Compositional matrix adjust.

Identities = 326/636 (51%), Positives = 402/636 (63%), Gaps = 93/636 (15%)

Frame = -2

Query 299 DTSSSGRGGKDKTPMCLVNELARYNKITHQYRLTEERGPAHCKTFTVTLMLGDEEYSADG 358

+ S+ + K+KTPMCLVNELAR+NKITHQYRLT E+GPAHCK FTVTL LG+EEYSA+G

Sbjct 5891 EESAGSKNSKEKTPMCLVNELARFNKITHQYRLTSEKGPAHCKRFTVTLKLGEEEYSAEG 5712

Query 359 FKIKKAQHLAASKAIEETMYKHPPPKIRRSE---EGGPMRTHITPTVELNALAMKLGQRT 415

FKIKKAQHLAA++AIE+T YKHP PK+ R EG R++ITPTVELNALAMKLG++T

Sbjct 5711 FKIKKAQHLAAAEAIEKTKYKHPVPKVVRRNAEGEGHSSRSNITPTVELNALAMKLGEQT 5532

Query 416 FYLLDPTQI-------PPTDSIVPPEF--------------AGGHLLTApgpgmpqpppp 454

+YLL+P Q+ PP D+++PP + AGG++L+ P

Sbjct 5531 YYLLNPRQVTSPDRIGPPPDAMMPPRYGIPLPPPHAFPPGVAGGNMLSQHPPPPHARMMD 5352

Query 455 pAYALRQRLGNGFVPIPSQP-MHPHFFHGPGQRP---------FPPKF--PSRFAL--PP 500

PA +R+ NG P P MH H GPG RP F PK+ R+ L PP

Sbjct 5351 PAAFVRR---NGPYAAPPPPPMHGHTAGGPGMRPRYLTPAQRAFMPKYNQQQRYPLMAPP 5181

Query 501 PLGAHVHHG-------------PNGPFPSVPTPPSKITLFVGKQKFVGIGRTLQQAKHDA 547

+G H+G P + +K+TL VGKQKFVG+GRTLQQAKHDA

Sbjct 5180 HMGGAPHNGMVPPPPMPHPHHGPLPHRYGISAALTKVTLVVGKQKFVGLGRTLQQAKHDA 5001

Query 548 AARALQVLKTQAISASEEALEDSMDEGDKKSPISQVHEIGIKRNMTVHFKVLREEGPAHM 607

AARALQVLKTQA + E L +S++E D KSPIS VHEIGI+R++TVHFKVLREEGPAHM

Sbjct 5000 AARALQVLKTQAKTQISEELNNSIEESDSKSPISLVHEIGIQRSLTVHFKVLREEGPAHM 4821

Query 608 KNFITACIVGSIVTEGEGNGKKVSKKRAAEKMLVElqklppltptkqtplkRIKVKTPGK 667

K F+TAC+VGSIVTEGEGNGKK SKKRAAEKML E K P ++P++RIKVKTP K

Sbjct 4820 KKFVTACVVGSIVTEGEGNGKKTSKKRAAEKML-EELKKLPPMSPTKSPVRRIKVKTPNK 4644

Query 668 SGAAAREGSVVSGTDGPTQTGKPERRKRLNPPKDKLIDMD-DADNPITKLIQLQQTRKEK 726

A+A VSG G + ERRKR + + ++D DA+NPIT+LIQLQQTRKEK

Sbjct 4643 GSASADAAGGVSGKAG---SAVGERRKRGSGSIKEKNEVDADAENPITRLIQLQQTRKEK 4473

Query 727 EPIFELIAKNGNETARRREFVMEVSASGSTARGTGNSKKLAKRNAAQALFELL-EAVQVT 785

EPIF+LIAKNGNE +RRREF+MEV+A G ARGTGNSKKLAKRNAAQ L + E+ ++

Sbjct 4472 EPIFDLIAKNGNENSRRREFIMEVTAHGMVARGTGNSKKLAKRNAAQNLLTAMGESGSIS 4293

Query 786 PTNET--------------------------QSSEECSTSATMSavtapaveataeGK-- 817

N T S E+ +T+ + + A A

Sbjct 4292 AENNTVLASSNTEPAVKEQFASATTVVATVTASQEQTTTTTVLQPIAEVANAAATTAAPV 4113

Query 818 -----VPMVATPVGPMPGILILRQNKKPAKKRDQIV 848

+P+V+T G +PGILILR NKK K+ + V

Sbjct 4112 QAHLDLPLVSTTAGQVPGILILRHNKKHFTKKKEYV 4005

**Conserved domains**


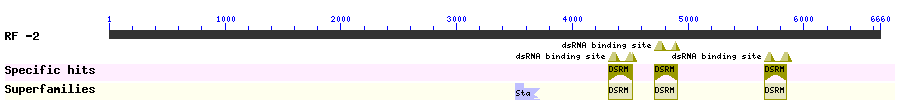


**Clip 1**

**>TRINITY_DN32205_c1_g4_i1 len=7273**

GTTTGTTTATTAAATTTTCAATTATTTTGAAAAACTAAGCTAGTCTAAATATTTGTTTAC

CTCGCATATTAATGTTTTTTTTTTGTTTATTAATGATTTCTCACTTCAAATTTGTGTGTC

TGAATTTATCAATTGTTATAAATAATTTCCAATTACATTAAAATGCGCTTCCTTCGTACA

CATGGCGCGTTAGCGTATAGTTAATTCGGTTATATGTTTGCCATTTCTACTAGAGCTTTT

GTTGTTTTTACATACATATATATTGTATTTTGTTATTATTAACGTGTTGCATACTATTTA

GCTTTAATTCTTGGATCTTTCGAATTACTTTCTCATGTACATACATATATATTAATATCA

AGTGTATAACGAAATTTAAATACGTATTTATATATTTCTAGTTTTAATTTATTTGCATTA

CTTATATTGTGTATATTATCTATTACTGCTTAATTTTTCTCTATTTGTTTATATATACTA

TATGTATATATATTTTTTTGTTTACACTTGTGTTATTAGTATTTTTTATGAAGCACTGCA

TTCAAGCAAAGATCTGATGCTGCACTTATTTCAATATTTCAAGAGTATCAAGATTAAATA

TGCATTGCATGAATGAGAATATAAAAAACGCTACCAAGCCGGCTGAAGTAAATGATAGTA

GATTCTCTTGTTCTATTACTTAGTTGTAACCCTAACTTTTTCGTTTTTCGAACGCTAACA

CAACGCATTTTCACATTTTGGAAAACATTTTGCTAAGCTAAAATTTTGTCAACGGGAGAG

GAAAAAGTTATAGCTGAATATTTTCGGTTAAGTTACTTTTATTTACTTTTTAATAATTTT

TTCTTGCAAAAAATACACGAAAGTTTCCACTTTTACTAAAATATTGTATAAGTTAAAGTA

TGCATATAACTTAGATGTAGGATTAGAGCAACTAAAAACTAAATATATTTTTATTACTTG

TATGCATTTAACATCAATGTATTCGTTTGTGTAAAATGTCGAAATATAGTTTTTAGAAAA

TCATTAAACTTATAATTACATGTAAGACAAGTAATTAAAGATGTTGGTTAAAAAACGCGC

AAAATATGCAAGAAATTACGAAATCATAAAACTAAACATTTAAAACACAGTTAAAAATAG

TGATGTAGGGGAAATTATAAACGAATACATACATATAGGCGTATGAAGACGGGATTTACA

AACAGATACATTTTGAGTTTAGTAGATCAAGTCCGAATGAATAGTCGCTAGGAAGAATTT

GCAAATTAGTTGTTGCTTTGTTGATAGGAAATCTGATGGGCGTAGTAATGTAAAAAGTGT

ATAAGTTCAGGGGAGAAGAATGTGCGAGATAAATAATTTCCAGCTTAGTTGTTTTGTTGT

TTTACCATTCGTATTAATTAAATGTAATTATAACTAACAAATTACAATTAGTTATCAATT

AATTAATAGGTTTTTGTGTGTAAATTTACATTTTTTTTTTTGGTTTTGTAAGCAAAAGTA

TGCAAATACATATTTAAATTTCAAATGCGAACTCGCCAAGGCGTCTTTTATAACATATGC

CGTTACGCTTTAGCCAAAGTGGAGTCGAATATAACTCGCGAATAAATGCAATGGAATTTC

GGAAAAAGACTGGCTCTTAATGTTTCACCAAATAATTTTAGTTGCTGCTTAACAATTTTG

AACAAAATACGCACATTACTCCTCACTTTTTAGTGACTGACGATTTTTAACACCTAAACT

ATATACACATACAGACAAATAAGTAGTACTGTAATTATCAGTTACAGTCACTTGAAATTA

TCCCGTCACATAAACTAACGATTGCGCTAAATACGTATACAACATTTTCAATTATATTTA

TATTTTTTTTGTTTTTTAAGTAAAGGCTCTGAGAAAAAATTAACTTCGCTACCCATTCCA

TTAACTCCACTTTGACACCACTGCAAGTTGCATTTGTTTGTGTTATAGTATTGCATTTAA

TGCGTTTACCTACATTAATAAAATTTCAACAAATACTATACGTACGTACACATACACACC

ACCCACAGTCGACGGCCCTTGGCCAAACCATAACAACGGGCTCACTGCCGTAGTGTTCTA

CCGTCTTCTTTTTAGTGCTGGTATTGTTATTGTTGTTGCAATTGCCGTCCGTCTGTTTCC

ACTTCTTCACGCGTCGTCTGTGCACGACGCAATCATGACGATGATGCTGATGAATTTTTC

GTTGAACTGCCACCGCCACTGTTGCCGCGCTGCTTGTCTATATACACGTTGAGCAAGCGC

ACTTTGCTTGGATTTAACACCGACAACTTCGGCTTCACCTTTTCCTCGCCCATCACTATG

TAGAGTTTGACAATACAAAAAACAGCAGCTTTCCGCACCATTGATTCGCTGTCATCAGTG

AGGCGCGCCAGATTCGGAAATATGCTATCTAAATGAAATTCGGTTAGCTCGGAACCGTGA

CGGTCTGCCAGCTCTGTGAGCAATTTGACGGCACATAAGTTCATCGGATAACAACTAGTC

GCAATGACAGGATTTACAATATTGATTGTGGCGTTCAGTGGCAACGATGATACGATTCGT

GGTATGATCAAATCGATTTCACGCGCTGTCTCCTTACTGTGCTGATAGCAGTTTATTATA

CGCAGCAATATTAGTTCGAGGAAGTTAATCCAAGTTTCCTTCATTTTCGTGCTGCGCACT

ATCTTGCCGAGCACACTTATCACGGCAATCATCACATCAGCTGTTTGCGAATCCAACAGT

CCTAGCAACATCTTCATGATGGCGCGGAAATGTTTGTTCGGTAACTCGCAGTTTCCACCT

TTAATGCAGATCTCCAAATTGGCGAGCCCTTGCTGAAGTTGCTCCACTGGCATATCTACT

TTTAGTGCACATGCCACGCGTATGATTTCACTTTCGGTTAAGCCGCTTTCGAGCACAAGT

TCACCATTCGATGCGACCGTGTAATTGCAACGTGAACCGTGATGTGTCGTTCCACTTACC

ACAGCTGTATTGGAGTTAGTTGTTGCAGAAGAGAGTCCAACGTTTGTAGTCATGCGTTGG

TGCTGTTCTAACAAATTTGCGTCTAAGCGCATTGTAGTGCTTTCGGGTGTGTTCGATTCG

GTTGTGGTGGCTGACTGGGTCTTTGAATTCGAAGAAGCGCACGAGTCTTGCTGGTCGTGC

ACGTAGCCATTGTAGCCGTTGGCATTCGAGAGCGAATGATACTGACGAGTATTATTAGAG

ACCGGCTCAATGCCGACACCTACACCGAAGCAATTTCGTATCTCTTCCGAAGTTTTATGG

ATGTTATGCTGTACATCAGCTTCCGAATAGAGTTCGTGGTCGACTGACGACTGCCGCGAG

CGGGGACTAGTATTAAATTGTGGCTGCAGTGACGAAAATGGACCAAGCGATGGGCTCTGT

AACTTGGGGCTGGCACTTGAATGTGGTGAGGATGGTGAATTTGTGCCGGAAGTGCTATTG

CGTCGCAAATGTGACTGTATAATGGCCTTTGCAGTATCCTGATATCCCTTGGGTAGGCTA

GACAACAACTTAGTCATCTGCGGTGTGCTGCGATTATAGAGCGCGATAAGACATTTACGC

GCTTGATCACGCAGATCCTTGCTCTTAGGATCACCAGCTTGTTGCACAATTTTCAGTACA

GCTTTATCAACCGCCAATGGTCCATCGTCGGAGGGAAAATCGGTGGACTTGCAGTAACTG

GTCACCAAGTCGGTGAGGAATTTCAATATGGCTATCTTGGTTTTCGTGCAAGGAGTTTGC

GCCGTGTCGGCGAGTATGCGGAAGACGTCCCTGAGCTGCTGATCGGTAGGGAAATATTGG

TGTACAATATGTAGCGTCTTCCCGATCTTAATGTGCATTGAGTTGAGCATCTCCGTGCCA

AGTTTGTTGAATAATCGCGTCAATAATATGAACAGCCAATCATGAAGCTCATTGGCATGC

GTTTGTATCAGCTCGGTGACTGCATCAAGAAAGAGTGCATACACTTTAGTGTGTGGATCC

ATGAATAGTTTACGGAACATGTCTAAAACAGCTTGTAGTTGTTGCGCTGTCAACTGATTG

CCATCGTCCAGGTACTGAGTTAGGCTGATAACGCCGTCCTTACGATCGGACCAATGCGTC

GAAGCACAGTACTGTATGATAGTGTCGATGTCGTCGAATGGTGCACGTGTGCAGCTCCAG

TCAAGCCGGTTACGTGAACCTGACAATGAATAGTTCGAATTGTTGCCGCGCGTCATTGAC

GAATCAAACGACCGCTCCGAACACACCGACGATGCCTCGGAGTCGTCGGACTCGTCGCGC

GACAACAACTTTCGACCCATTCTCAAACCAGCCGTGTATCCACGCGAATACTCGCCATAA

TCTATAGTCCGTTCTCCTTCCGGTGAAAGCGCGTCGGCCAGTGCGTTTTCCGCCTCACGA

CTCTGTTGTAAAATTCGTGTCGCAGTTAGTGGACGCGAATTACTGCGCTCAGGTGTCCGT

CGTGCTGAACCCATTGAGCTATTTGTCGAATAAATGCTCCGTTTCATGGCAACGCGTGTG

GGACTGGTTTCCCGCGAGCTGGTCAATGAGCGCGGTATACCGGAGGCCTTTTTAGGTATA

GTACCTGTTATAGGTCTATATCCGTATTGTTCTCGCAGTTTTGAACTGGGTGAGGTGGAT

CGAGATCCAGGCTGCGACTGCGATACTCCAGCGCGCCCACGAGTACGCGGCGAAATACCT

ACATTTGACTGCGCAGTTGTAGCTGCGGCTGCCGGAGTGACACCTCCCATATACCGTGGA

CGCGGTAAAGATCCCGTTGCTGCAGGACCTGTACTGCTGACTGTTCCAGCAGTGTTGCTA

ACAGTGCTTAACTTTTTACGAGGATAAAACGAGTACTGTGCGCGTGCTTTGGCACGTTGG

GCAGCAGCAGTGTCTACTGCAGACACACTACGCATTCCAGCCGCTGGCTTTTGTAGTGAA

CCTGGTGAACGCTGGAATCGTGTTGTCGCAGCTGCAGAGCGACGCTCAGCCTCGACTAGC

ACATTACCACCGTCTCGCTCTCGTTCCAGCGCACGTTGTGACGCAATGTCTAGCGTTGTA

TAGATCTGGTCAGCAAGATCGGGGAAATGCCGCCGAAATTTCCAATAAGCACGACGCGAA

TGACGACGTGCCTCGTTATCTGCATCACTTATTGATCGTTTGAGCACATCACGAAGTTGC

TGAGAACACCGCTCCATAGTTTTCGTCTGCCACTCATCGAACATCAAGCAAAGCATCTCA

CATAAAGCGGCGCGTATATCTTTCGATTTTGCTTGCTGCAGTGTCTCGGTGACGAGTTTA

ATTATTTTCGACGAATGGGTGTACTTGATAATGTACTTTAGAGCGATTGTCGATGATGAG

GCGATTACTTTAGCCGAATTTTGAATCAAATTGATAAGTGCCTCTAAAATAGCCAAACAG

AACGGCTCGAGTTTGTTGCGCAATGTCTTTGACATATATGCGATTGTAATGCACGCCTCG

CGTATTACTTGTGAACGCAACTCCTCCTTCAGTATGTCGATAAATGGAATTGAAAGATCT

TTTAACTGCGCCACGAATTGGGGTTGTGAATGTATATTCAGCATTAAGAGTGAACGCACT

TTCTTTAGCGAATCGATGCGTTTCTCCCAATCGGCATTCTTATCACTAATAACAACGATA

ATATTCCTGTAAATGTCGTCCATGTCTTTTGGATGAAATATAGTTAATTGTGGCACAATT

TCGAAGGAAGATTCAAATATTTCCATTGTGACGGCGCCGGCATCACCACTTGCGCTTGAA

TCCGCGCTACTTGGCTTATTTCGTGTTGACGCAGATATAGTACGTTTAACAATTTTCGTC

GGGCGATCACGAACACTCACATTATCTGACTCATCGTGACCATTATTGACGGATGGATTA

GTTAGAAGAGCTGACTTCAACAACAGACCCTCCGCTTTGATCTGATCAAATTTTTGTTCT

AGCACAGCTAATTTCGAAGCGGGCACATCATCCATTTTGCGGAGATCCACACGCAAGCGA

TCGCCTACATGCTTATAGATTTCTACTAATGTCTGCATAGCCGCATCCCGCACCGTTGCT

GTGGGATCGCCTAGCAAAGCACTGGTAGGCTGTATGTAGGTGCGCACACTCAATTGCGAT

GTGCCGTATTCGTTCAATGTATTCACAATGGTCTGCAAAAACTCTTCACGTACTTTAGCA

TTCTTATGTTTGAAGCAAGCGACCGCTAATTTATCAATGGTGGCTTGTGGTGAGATCACT

TTGTATTCCATCACAATCTGTAATAGCAACTGGGCTTTCTCACGTACTGTATCCTTACTG

TCGCCCAGACGATCTATCACATGTGGCAGCACGGTTGCGCTGTACGCATTAAAATCAGGT

CCAAGCCTTTTGATCAGTTCTGAAAATGCTTCTAATGATTTTTGTGCTATTTTAAAATGA

CTGCCAGTTAACCAAGGCATCAGTCCATCCACAAGCATGCCCATATCCATGCAGACAATC

GAGTTGGCCTCATCACTTAGGAATGTAACCAAGTCTTCGGCGAGCAGAGCTTTAACTCGC

ATATCCGCTTTTGGCATCATTTGTATAAAGCCATCTAAATCGTTAGGTTTGCGATACGCC

ATATTTGAGTTTTTGCAGTGTTGGGTTCTTGTGAGAGAAATAAATAGTGTGCGGCAACGG

CAACGCGCCGCCTATCTCTATCTTTATTTTTGAATTAACAAATTCCAATGTCTTTTTCGT

CTCTCTTTCACTGCTCTTCTCGGCTTCTGCTTTGCGCAGTAATTCCCTGCTCAGCTTTTC

TCGATTTTGTATTTGTTTGATATCTCCTTTCCTTCCTTTTCAAGATCCGCTAATTCTTTT

ACCGTAGTTCAAACAGCTCACAAGCAAAAACAACACCCAACAAGCACTTACATTCACACG

CACTTTATTCACTCTTTTTTTGTTTTATCGCTCAATTCGTTTCTCGCTGCTTCTCTGCTC

GGGCTTTCCAACGAATGACGCAGATGCAGCAGCGACACTAAACTGTTTTGCTATTAATTA

CCAAATTTTACTTTCTAATTCGAAGCATATGATATTTTTTTATTTCTTCACAATTTGCCA

TAACTTTTGCACAAATTTTCAGTTCGTTCGAGTTTTATTTTTAGTTTTAAATTCGCTGTT

TCTTCACCCAAACGTAGAATCGTTGGAAGATTATTCGCTCTTCGTAGAATTATGTCATTT

ACAAAAAAAGAGT

**tBLASTn(First hit)**

Score = 523 bits (1347), Expect = 2e-159, Method: Compositional matrix adjust.

Identities = 326/636 (51%), Positives = 402/636 (63%), Gaps = 93/636 (15%)

Frame = -2

Query 299 DTSSSGRGGKDKTPMCLVNELARYNKITHQYRLTEERGPAHCKTFTVTLMLGDEEYSADG 358

+ S+ + K+KTPMCLVNELAR+NKITHQYRLT E+GPAHCK FTVTL LG+EEYSA+G

Sbjct 5891 EESAGSKNSKEKTPMCLVNELARFNKITHQYRLTSEKGPAHCKRFTVTLKLGEEEYSAEG 5712

Query 359 FKIKKAQHLAASKAIEETMYKHPPPKIRRSE---EGGPMRTHITPTVELNALAMKLGQRT 415

FKIKKAQHLAA++AIE+T YKHP PK+ R EG R++ITPTVELNALAMKLG++T

Sbjct 5711 FKIKKAQHLAAAEAIEKTKYKHPVPKVVRRNAEGEGHSSRSNITPTVELNALAMKLGEQT 5532

Query 416 FYLLDPTQI-------PPTDSIVPPEF--------------AGGHLLTApgpgmpqpppp 454

+YLL+P Q+ PP D+++PP + AGG++L+ P

Sbjct 5531 YYLLNPRQVTSPDRIGPPPDAMMPPRYGIPLPPPHAFPPGVAGGNMLSQHPPPPHARMMD 5352

Query 455 pAYALRQRLGNGFVPIPSQP-MHPHFFHGPGQRP---------FPPKF--PSRFAL--PP 500

PA +R+ NG P P MH H GPG RP F PK+ R+ L PP

Sbjct 5351 PAAFVRR---NGPYAAPPPPPMHGHTAGGPGMRPRYLTPAQRAFMPKYNQQQRYPLMAPP 5181

Query 501 PLGAHVHHG-------------PNGPFPSVPTPPSKITLFVGKQKFVGIGRTLQQAKHDA 547

+G H+G P + +K+TL VGKQKFVG+GRTLQQAKHDA

Sbjct 5180 HMGGAPHNGMVPPPPMPHPHHGPLPHRYGISAALTKVTLVVGKQKFVGLGRTLQQAKHDA 5001

Query 548 AARALQVLKTQAISASEEALEDSMDEGDKKSPISQVHEIGIKRNMTVHFKVLREEGPAHM 607

AARALQVLKTQA + E L +S++E D KSPIS VHEIGI+R++TVHFKVLREEGPAHM

Sbjct 5000 AARALQVLKTQAKTQISEELNNSIEESDSKSPISLVHEIGIQRSLTVHFKVLREEGPAHM 4821

Query 608 KNFITACIVGSIVTEGEGNGKKVSKKRAAEKMLVElqklppltptkqtplkRIKVKTPGK 667

K F+TAC+VGSIVTEGEGNGKK SKKRAAEKML E K P ++P++RIKVKTP K

Sbjct 4820 KKFVTACVVGSIVTEGEGNGKKTSKKRAAEKML-EELKKLPPMSPTKSPVRRIKVKTPNK 4644

Query 668 SGAAAREGSVVSGTDGPTQTGKPERRKRLNPPKDKLIDMD-DADNPITKLIQLQQTRKEK 726

A+A VSG G + ERRKR + + ++D DA+NPIT+LIQLQQTRKEK

Sbjct 4643 GSASADAAGGVSGKAG---SAVGERRKRGSGSIKEKNEVDADAENPITRLIQLQQTRKEK 4473

Query 727 EPIFELIAKNGNETARRREFVMEVSASGSTARGTGNSKKLAKRNAAQALFELL-EAVQVT 785

EPIF+LIAKNGNE +RRREF+MEV+A G ARGTGNSKKLAKRNAAQ L + E+ ++

Sbjct 4472 EPIFDLIAKNGNENSRRREFIMEVTAHGMVARGTGNSKKLAKRNAAQNLLTAMGESGSIS 4293

Query 786 PTNET--------------------------QSSEECSTSATMSavtapaveataeGK-- 817

N T S E+ +T+ + + A A

Sbjct 4292 AENNTVLASSNTEPAVKEQFASATTVVATVTASQEQTTTTTVLQPIAEVANAAATTAAPV 4113

Query 818 -----VPMVATPVGPMPGILILRQNKKPAKKRDQIV 848

+P+V+T G +PGILILR NKK K+ + V

Sbjct 4112 QAHLDLPLVSTTAGQVPGILILRHNKKHFTKKKEYV 4005

**Conserved domains on [lcl|ORF34]**

**
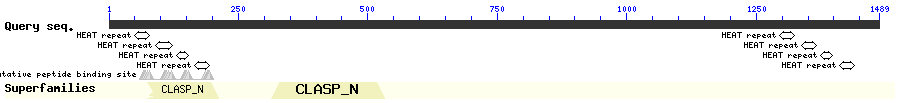
**

**Elp-1**

**>TRINITY_DN33357_c0_g1_i4 len=4660**

TCGCCTTTTTTGCTGCTGTCAAAACAAACCCATCTGATGACAACAACATGACATCTGTGC

ACGTTTTGCGCAATTTTCATTTTAGCATATCTGTACCGTTGCCACTTTGTGAACACTAAA

GTCGTGTTTTTTATGCGTAATTTAAGAAAGTAATTAGTAATTTTCTAAATTATGCGTAAC

CTCAAGTTGCAGTCGTGCAAACAACTGGACTTAAAAGTGTCCAATGTTAAATACCTGCTA

CTGGATCCAAACGCAAGCCGAAAGGAGGCTGATACACTTGTCTATGTAGTGACAGATTCT

GAAGTGAACGAAATAAAAACATCAACCGGGAGCATAAAAGAAATAGCCTCCGTTCCTGGT

ATCGTAGCAGCAGAGTATTTGGCGCTAAATAATGAGATTTGCCTGGCCACACAGGCAGGC

GAAGTACTCGCTGTTTCACCCAGCACCGGAACCATCAACGAGTGCACTTTTTGTGGGGTA

GGTTTGCAGTGCATGGCATGGAGTCCTGATCAGGAAGTTGCTGTTTTTATAACAAATTCA

GGCAATGTTGTTGTTATGACTTGCACCTATGACGTCATAACCGAGCATGTTTTGGAGGAA

CAATGCGATCCCGACAGCCAATTCGTTAATGTAGGTTGGGGTAAAAAGGAAACACAATTT

CATGGTACGGAAGGTAAGGCAGCCGCTAAAACGAGAAACGATTTCAAGCCACCGGCGAAA

GTAGAGGAACTGCCACAAGATGTGAATATTACATGGCGCGCAGATGGCGCTTACTTTGCC

GTATCATTTGTTAGTTCTGAAGCGGGTCGTATGTTCAAAGTGTTCAATAAAGAAGGCGAT

TTGACATATATATCTGAACGTTGGAATGATTTGCAACCACCGATCGCATGGCGACCTTCG

GGCACTTGGATTGCAGTGCCTCAAATATTTCCCAATAAAAGCACCGTAGCTCTCTTTGAA

AAGAATGGCTTGCGGCATCGTGAAATCGTACTGCCGTTCTCATTGATGGATGAGCTCATA

CGCAGTCTGCGCTGGAGTAACGATTCAGACATACTCGCAATTGAAACGTTAGATTCAACC

GCGCAAAAGCAACGCCTGTACTTTTATACGATCGCAAATTACCATTGGTATCTAAAGCAG

GTGCTAACTTTTGACAGAACGGATCCCATTGCATACCACTGCTGGGACCAACGAATAGGC

GAGGAAAAGACTTTGCACGTGTGGCTAGAAAGTGGTAGATACCTAATTTACCGCTGGCGA

TTCGATTTCGATCGCTTTGCGCGCAGTGGCATTGTGGCTGTCATCGACGGTAAAAAGTTG

CTATTGACCGACTTTTCCAAGGCAATTGTGCCACCGCCAATGTGTAGCAAAGAAATTGAG

TCAGATACGTACATCAACGCATGTGTCATTTCAAGAAATAATAGCGGAAATTTGCAGTTG

TGCATTTACGACGCCAATGAGCAGCTGCAGCTGTTTGTAGCGAACCATGTTGAGAATTCA

CTAGCTTTTGAAAAAGTTTGCGTTTTAGAAAAGCTACAAACATATTTGGGGGAGTACAAT

ACACCACCATCGGAGCTGGGCAACTTGTTTTGGTTCGATGAAAACTACTTAGTAGCAACC

GTTAACGTTAATGAAAAGAGCAAAGTGCTTTTGCTTATATTGGATACTGGAGCGAAAACC

TATGACGTGGTCACTGCCTTACAGCTGAACTCCAACGCGGCTTGCAGCTGCATGGGTTTT

GTTACATTGGAGCAGTGCTTTGTGCAAACTACGGATGGAAAGATTCAGCAACTATCACAA

CAAAACGATACTACGCTCAAATTGGACAAAACATACCAAGAACTGGAGCAAGCCGCATTG

CAGCTGGAGTGGCATCAAACGCAGTCAGCGGAGTCTGAGTGTCTTATTGCTTTACTACAA

AATCGGCGCCTATATATTGACAGCGAGCTAGTGTCCGGCGACGTGACGTCCTTCTGCTTG

GCAGGCAACTACTTAGCCTATACTAAACTGACTGAATTGAATTTCGTGCTACTGTCCACA

CGCCAACACGTTTACAGACGCAATATGGAGCGTGGCGGCAGAATCGTTACGACTATGGCG

AATGATGCGCGTGTCGTGCTACAAATGCCGCGCGGCAATTTGGAAGTTATTTCGCCGCGT

GTGCTGGCGCTGGAGATTATCGGAAAACAATTGGATCAAAAACGCTACAGTGAAGCTTTC

AATATATTGCGAAAGCAACGCATAAATTTGAATATTCTTTGTGATCATAATATGATAAGC

TTTGTGCAAAACGTAACTATCTTCTTGAGTCAGATCACGAATCCGAATTGGTTGAACTTA

TTCCTGACTGATCTGCAGAACGAGGACTTTTCGAAAACAATGTACGCAAGCAACTACACC

GCAGCTCACCAAGCATATCCCGATGGCTTCAAAATCGAATCCAAAGTTGCATATCTCTGT

GCGCTGTTGTGCGCGCGTATGGCGGAAATTAATAAAGAACGTTTTCGATTGCCCATCATT

ACGGCGTATGTGAAAACAGGCAAGCTGGAGCAAGCGCTCCAATTGATTTGGCAAGTGAAG

AAAGAGCAAATTGAATTGGCGAAACAGTCCGAGGGCCCTGTGCAAGAAGCAGCCGAGGAG

GCACTCAAATATTTACTCTACCTAGTCGATGTAAATGAATTGTATAATGTCGCCCTAGGC

ACGTACGATTTTGGTCTGGTGCTTTTTGTCGCGCAAAAATCGCAAAAAGATCCGAAGGAA

TTTTTGCCATTCCTCAATGAACTAAAACAGCTGGAGGTGAATTATCGTAGATTCAAAATC

GATGAGCACTTGAAGCGCTATGAAAAGGCGCTGGAAAATATCTCCAAATGTGGCGTTGAA

AAATTCTCCATAGCTTTGGAGTTCATCCAGCTGCACGAGCTGTATAAGCTGGCATTGAAG

TTTTACAAAATAGATGTAGAAGAAAATGAAGCGAAAGAAGGACAACATTCTTTACGCGAA

TGCCAGCGACAGATATGTTTAGCTTTTGCAGACTATCTGCGCGCTAAGAATGAACTCGAA

AGTGCGAGTATTATGTACGAGCGTGGCGGCAATCTCACACAAGCGCTGCTCAGCGCCAAA

AACGTATTGGATTGGCGGCGTGTTTTGATGTTGGCGCAAAAGGATGGAAAGGATATAGCG

ACGGCGGCATTATCCATGGTATCTGCGCTGCAAGAGCAAGGTCGGTACGATATTGCCCAT

CAGCTGCTCAAAACGTATGGCACAAACTTTAAAGAAGCGCTGCAATGCCTGCTCAAGGGT

AATCTCTATTTGGCAGCGATTCTGGAAGTCCGCATGCACTGTCAACAGATCGATTTGCTA

GATGAGGTCGTAAAGCCAGACTTGATTGCATACAGCAAGCAATTGGCACAGCAGTTGGCT

GATGATGAAAAGCTCTTCGTTGAGCACAAACAACGTTTGCATGATGTGCGTGTGCTGGCG

CAAAAAAAGCGCGATGGACTCGTGAATTGTTATGATCAGCCAGATATTGACGAGGCTGAT

TTGCTGTCTGACACTACCAGTTTGCGCTCATCACGCTACACGGGTAGCTCGCAAGGCACT

GGCAAAACATTCCGCTCGAGCAAGAATCGGCGAAAGCACGAACGAAAATTGCTAAGTCTC

AAGCCGGGCAATCCATTCGAAGACATCGCACTTATCGACGCGCTGTATAATCTAGTCATG

AAGTTGGCCAATCAGCAGCAACAGATGCGTGACACATGCAAAGCCTTAATTGAATTGCAG

TTGGATGAGGTGGCTGGGGCGCTACAAACGCAATACGGTCACTTATTGACGTTGATGCAA

GACTCCTTCGATGCGATATGGACAGACGAAATGGTGAATACGCAAGCAATGCAGTACAAA

CCAACACCCTACACCGATTACACACAACTGCAAAATGAGCAACGCTACGCCGTGTTAGCT

CCTCAGAAGCGTTTCAAGCCGCAAATAAATCTTATTGATTGGAAGTGTAAAATACTATCT

TAAGCGTGCACGACACATACAAATCAAATTAACCAACAAATCACTAACATCTTTAGCTGC

AAGCATGCGCACTTTGGTTGGTCTTGGTCTGGTGTCTAATCGTCTAGCATAGCATAAATT

AGCGTAAAGTACTTTAGTAATTAATCAAATTTGTATTTACAAGCAACATTTTTATATACA

AATGTACACGTATACAACAACAGTAAAACATTGTAATAATAAAAATATGTGTTTGTATAT

AAATTGCAATGTTTACGTTAAATTTGGTACATACGTAGTTGATTAAAACAGAAAGTGAAA

CTCTTTTATGTTAAATGAAAAATTATGCAAATTTTTTTTTGTTTTTTTTCTTTTTTAAAG

AAATGTTTTTACTTTTTTCAAATACTGCTCAACTGGCACGCTACTGATTGAGTGCGTTGT

TTGATGGATGTCTGGCGGTAGTTGGGCAACCGTGAAAATCGATGTTTTTAAGTAAAAAAT

TATAACTAACACCGTGCAAATATACAGATTCCTATATTACTTTCACCATTAGAGTTTTAA

TATTTTGCATAATTTACAATAACAAAATGATAAGTTGTTTGCCCAAAAATGTATACTGCG

AAATAATTGAAAATAACAATAATTATATGATGAACATAAT

**tBLASTn(First hit)**

Score = 1102 bits (2849), Expect = 0.0, Method: Compositional matrix adjust.

Identities = 625/1299 (48%), Positives = 856/1299 (66%), Gaps = 65/1299 (5%)

Frame = +1

Query 1 MRNLKLRYCKELN-AVAHPQHLLLQPELNGGASD-IYFVVADNKIYAVQESGDVRLKVIA 58

MRNLKL+ CK+L+ V++ ++LLL P + +D + +VV D+++ ++ S +K IA

Sbjct 172 MRNLKLQSCKQLDLKVSNVKYLLLDPNASRKEADTLVYVVTDSEVNEIKTSTG-SIKEIA 348

Query 59 DLPDIVGVEFLQLDNAICVASGAGEVILVDPQTGATSEGTFCDVGIESMAWSPNQEVVAF 118

+P IV E+L L+N IC+A+ AGEV+ V P TG +E TFC VG++ MAWSP+QEV F

Sbjct 349 SVPGIVAAEYLALNNEICLATQAGEVLAVSPSTGTINECTFCGVGLQCMAWSPDQEVAVF 528

Query 119 VTRTHNVVLMTSTFDVIAEQPLDAELDPDQQFVNVGWGKKETQFHGSEGKQAAKQKESDS 178

+T + NVV+MT T+DVI E L+ + DPD QFVNVGWGKKETQFHG+EGK AAK + +D

Sbjct 529 ITNSGNVVVMTCTYDVITEHVLEEQCDPDSQFVNVGWGKKETQFHGTEGKAAAKTR-NDF 705

Query 179 TFIRDEQELNQDVSISWRGDGEFFVVSYVAAQLGRTFKVYDSEGKLNHTAEKSANLKDSV 238

+EL QDV+I+WR DG +F VS+V+++ GR FKV++ EG L + +E+ +L+ +

Sbjct 706 KPPAKVEELPQDVNITWRADGAYFAVSFVSSEAGRMFKVFNKEGDLTYISERWNDLQPPI 885

Query 239 VWRPTGNWIAVPQQFPNKSTIALFEKNGLRHRELVLPFDLQEEPVVQLRWSEDSDILAIR 298

WRP+G WIAVPQ FPNKST+ALFEKNGLRHRE+VLPF L +E + LRWS DSDILAI

Sbjct 886 AWRPSGTWIAVPQIFPNKSTVALFEKNGLRHREIVLPFSLMDELIRSLRWSNDSDILAIE 1065

Query 299 T--CAKEEQRVYLYTIGNYHWYLKQVLIFEQADPLALLHWDTRCGAEHTLHVLKESGKHL 356

T ++QR+Y YTI NYHWYLKQVL F++ DP+A WD R G E TLHV ESG++L

Sbjct 1066 TLDSTAQKQRLYFYTIANYHWYLKQVLTFDRTDPIAYHCWDQRIGEEKTLHVWLESGRYL 1245

Query 357 VYRWAFAVDR--NNSIVGVIDGKRLLLTDFDEAIVPPPMSKIVLKFETYINAFI-----S 409

+YRW F DR + IV VIDGK+LLLTDF +AIVPPPM ++ +TYINA + S

Sbjct 1246 IYRWRFDFDRFARSGIVAVIDGKKLLLTDFSKAIVPPPMCSKEIESDTYINACVISRNNS 1425

Query 410 HGTSLWVYTCDRKI--YLNEHI-HTLGKE----LQKPIMLMPDAELSGLHLANLTHFSPH 462

L +Y + ++ ++ H+ ++L E L+K + + L NL F +

Sbjct 1426 GNLQLCIYDANEQLQLFVANHVENSLAFEKVCVLEKLQTYLGEYNTPPSELGNLFWFDEN 1605

Query 463 YLLATHSSAGSTRLLLLSYKDNDNKPGEWFYRVHSSVRINGLVNAVAVAPYAMNEFYVQT 522

YL+AT + +++LLL G Y V +++++N + + + +VQT

Sbjct 1606 YLVATVNVNEKSKVLLLILD-----TGAKTYDVVTALQLNSNAACSCMGFVTLEQCFVQT 1770

Query 523 VNNGHTYEVSLKADKTLKVERSYVQLHEPADQIDWVIVKGCIWDGYTGALVTLRNQHLLH 582

+ G ++S + D TLK++++Y +L + A Q++W + + L+ L L+

Sbjct 1771 TD-GKIQQLSQQNDTTLKLDKTYQELEQAALQLEWHQTQS----AESECLIALLQNRRLY 1935

Query 583 IDGYRIGEDVTSFCVVTNYLVYTQLNAMHFVQLDDRRQVASRNIERGAKIVTAVARKARV 642

ID + DVTSFC+ NYL YT+L ++FV L R+ V RN+ERG +IVT +A ARV

Sbjct 1936 IDSELVSGDVTSFCLAGNYLAYTKLTELNFVLLSTRQHVYRRNMERGGRIVTTMANDARV 2115

Query 643 VLQLPRGNLEAICPrvlvlelvgdllergKYQKAIEMSRKQRINLNIIFDHDVKRFVSSV 702

VLQ+PRGNLE I PRVL LE++G L++ +Y +A + RKQRINLNI+ DH++ FV +V

Sbjct 2116 VLQMPRGNLEVISPRVLALEIIGKQLDQKRYSEAFNILRKQRINLNILCDHNMISFVQNV 2295

Query 703 GAFLNDINEPQWLCLFLSELQNEDFTKGMYSSNYDASKQTYPSDYRVDQKVEYVCRLLEQ 762

FL+ I P WL LFL++LQNEDF+K MY+SNY A+ Q YP ++++ KV Y+C LL

Sbjct 2296 TIFLSQITNPNWLNLFLTDLQNEDFSKTMYASNYTAAHQAYPDGFKIESKVAYLCALLCA 2475

Query 763 QMNRF-VSRFRLPLITAYVKLGCLEMALQVIW---KEQQEdasladqllqhllyl----- 813

+M RFRLP+ITAYVK G LE ALQ+IW KEQ E A ++ +Q

Sbjct 2476 RMAEINKERFRLPIITAYVKTGKLEQALQLIWQVKKEQIELAKQSEGPVQEAAEEALKYL 2655

Query 814 ---VDVNDLYNVALGTYDFGLVLFVAQKSQKDPKEFLPYLNDLKALPIDYRKFRIDDHLK 870

VDVN+LYNVALGTYDFGLVLFVAQKSQKDPKEFLP+LN+LK L ++YR+F+ID+HLK

Sbjct 2656 LYLVDVNELYNVALGTYDFGLVLFVAQKSQKDPKEFLPFLNELKQLEVNYRRFKIDEHLK 2835

Query 871 RYTSALSHLAACGEQHYEEALEYIRKHGLYTDGLAFYREHI-------------EFQKNI 917

RY AL +++ CG + + ALE+I+ H LY L FY+ + E Q+ I

Sbjct 2836 RYEKALENISKCGVEKFSIALEFIQLHELYKLALKFYKIDVEENEAKEGQHSLRECQRQI 3015

Query 918 YVAYADHLRAIAKLDNASLMYERGGQLQQALLSAKHTLDWQRVLVLAKKLSEPLDQVAQS 977

+A+AD+LRA +L++AS+MYERGG L QALLSAK+ LDW+RVL+LA+K + + A S

Sbjct 3016 CLAFADYLRAKNELESASIMYERGGNLTQALLSAKNVLDWRRVLMLAQKDGKDIATAALS 3195

Query 978 LVGPLQQQGRHMEAYELVKEHCQDRKRQFDVLLEGHLYSRAIYEAGL---EDDDVSEKIA 1034

+V LQ+QGR+ A++L+K + + K LL+G+LY AI E + + D + E +

Sbjct 3196 MVSALQEQGRYDIAHQLLKTYGTNFKEALQCLLKGNLYLAAILEVRMHCQQIDLLDEVVK 3375

Query 1035 PALLAYGVQLESSLQADLQLFLDYKQRLLDIRRNQAKSGEGYIDT--DVNLKEVdllsdt 1092

P L+AY QL L D +LF+++KQRL D+R K +G ++ ++ E DLLSDT

Sbjct 3376 PDLIAYSKQLAQQLADDEKLFVEHKQRLHDVRVLAQKKRDGLVNCYDQPDIDEADLLSDT 3555

Query 1093 tslhssQYSGTSRRTGKTFRSSKNRRKHERKLFSLKPGNPFEDIALIDALHNHVTKIAQQ 1152

TSL SS+Y+G+S+ TGKTFRSSKNRRKHERKL SLKPGNPFEDIALIDAL+N V K+A Q

Sbjct 3556 TSLRSSRYTGSSQGTGKTFRSSKNRRKHERKLLSLKPGNPFEDIALIDALYNLVMKLANQ 3735

Query 1153 QQPVRDTCKallqlanaadadplaaalqREFKTLLQAVDAALDEIWTPELRGNGLMADHL 1212

QQ +RDTCKAL++L A L ++ LL + + D IWT E+ M

Sbjct 3736 QQQMRDTCKALIELQLDEVAGALQT----QYGHLLTLMQDSFDAIWTDEMVNTQAMQYKP 3903

Query 1213 TGPNVDYLALQKEQRYALLSPLKRFKPQLIMMDWQHEIL 1251

T P DY LQ EQRYA+L+P KRFKPQ+ ++DW+ +IL

Sbjct 3904 T-PYTDYTQLQNEQRYAVLAPQKRFKPQINLIDWKCKIL 4017

**Conserved domains**


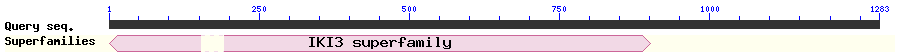


**GLD-1**

**>TRINITY_DN24535_c0_g1_i2 len=1790**

TTTGGCAAAGTGTAAAATTTAATTCAATTCGTTCTAAAAATTGTTCTTACAATTGCAGTA

AATTAGAAAAACGTGTCACAACTGCAAACATACAAATACAGACATAAGTATACACATATA

TACACACATATGAAAATACAAAAATTGTGCATTCAACAAAGAAATTTTACTGCTTGTGCA

TTAATAATTGCAAATAAGTTTTCATAACACGCGTGTTTCTTAAATAAAACCCAACAAAAA

AAAAAACAAAGTGTGCAATAAATTCTTTGAAAATCAAAAAATCAAATCGAATTAAAATTC

CCAAGAACTTTACAAGTTAAAAGTGTGGCTAGTGAATTTTCATTTATCAAGCCATTCAAA

ATCTAATCTAAGAATTGCTTTTAAAACGCAAAATTTTAATTAAATAAATCAAAACTATTA

TTTGTAATCAAATCTTAAAAACCCACAAAAAACAAAAAAAGCTTCAAAATGAGTCTGTGT

GATGTCAGCGCCATTACTATTCAACAAACGCCCCAACAGCAACATCAGAGTACACAAAGC

ATAGCCGATTATTTGGCTCAATTGCTGAAGGATCGCAAACAAATGGCAGCATTTCCAAAT

GTCTTCAATCATGTAGAACGTCTACTCGATGAAGAAATTGCGCGAGTACGTGCCTCACTG

TTTCAGATAAATGGCGTCAAGAAGGAACCACTTACGCTGCCAGAACCTGAGGGCACTCCT

GTGCAGCTCAATGAGAAAGTCTATGTGCCAGTGCGAGAACATCCAGATTTCAATTTCGTT

GGCAGAATTTTGGGCCCACGTGGCATGACAGCTAAGCAATTAGAACAAGAGACTGGTTGT

AAAATAATGGTTCGAGGCAAAGGCTCGATGAGAGATAAAAAGAAGGAGGATGCAAATCGC

GGCAAACCCAACTGGGAACACTTGTCCGATGATCTGCATGTGCTGATCACCGTGGAGGAC

ACAGAGAATCGTGCCAAAGTCAAATTAGCGCAGGCTGTTGCTGAAGTGCAAAAGTTGCTC

GTACCGCAAGCCGAAGGCGAAGATGAACTTAAAAAACGTCAACTCATGGAATTAGCTATA

ATTAATGGCACTTATAGGGATACGTCTGCGAAAAATCCCGTTCCAGCCTGTGAGGAGGAA

TGGCGTCGTGTGATCGCTGCATCGGCTGAAAATCGTCTACTAGCGCCAGCATTGCCTGGC

CTGGCACAGCAGATACGCGCTCCACAAGCCACCACCTTGGGCGCTCCGTTGATACTCTCG

CAACGCATGACCGTGCCCACTACAGCGGCGAGCATACTGTCCGGCCAAGCTGCGCCCACT

GCATTCGAGAATCCCGCACACGGCATGATTTTCGCACCATACGGCGATTATGCGAATTAT

GCTGCTCTCGCTGCCGGTAATCCCTTGCTGGCGGAATACACAGACCATAGCGTAGGCGCA

ATTAAACAGCAGAGACGTTTGGCTAACAGAGATCATCCATATCAGAGGGCTGCCGCCGCG

GTCGGTGTCACAGCTAAGCCAGGTTTCATTGAGATACAATAAATGCCAGAATCGCATTGA

TTATGACGAAAAGAAGCATAAACTTAAATCAAAAAGCTAAAGTTAATGAGTGAAATTACA

ACAACAGTTACAATGTTATAGTTAAAGTTAAATATTGTTTATCAACTTTGCTATGCATAC

ATACATACATACATGCAATTTATCACTATTTGTATATGTATGCATGTATATGGCTCCATT

AATCTCTTTCGTTTAATTTTTTCTTTGCATACTCGTATAAGAAACTAAAG

**tBLASTn(First hit)**

Score = 527 bits (1358), Expect = 0.0, Method: Compositional matrix adjust.

Identities = 289/335 (86%), Positives = 301/335 (90%), Gaps = 8/335 (2%)

Frame = +1

Query 76 ADYLAQLLKDRKQLAAFPNVFTHVERLLDEEIARVRASLFQINGVKKEPLTLPEPEGSVV 135

ADYLAQLLKDRKQ+AAFPNVF HVERLLDEEIARVRASLFQINGVKKEPLTLPEPEG+ V

Sbjct 544 ADYLAQLLKDRKQMAAFPNVFNHVERLLDEEIARVRASLFQINGVKKEPLTLPEPEGTPV 723

Query 136 TMNEKVYVPVREHPDFNFVGRILGPRGMTAKQLEQETGCKIMVRGKGSMRDKKKEDANRG 195

+NEKVYVPVREHPDFNFVGRILGPRGMTAKQLEQETGCKIMVRGKGSMRDKKKEDANRG

Sbjct 724 QLNEKVYVPVREHPDFNFVGRILGPRGMTAKQLEQETGCKIMVRGKGSMRDKKKEDANRG 903

Query 196 KPNWEHLSDDLHVLITVEDTENRATVKLAQAVAEVQKLLVPQAEGEDELKKRQLMELAII 255

KPNWEHLSDDLHVLITVEDTENRA VKLAQAVAEVQKLLVPQAEGEDELKKRQLMELAII

Sbjct 904 KPNWEHLSDDLHVLITVEDTENRAKVKLAQAVAEVQKLLVPQAEGEDELKKRQLMELAII 1083

Query 256 NGTYRDTTAKS-VAVCDEEWRRLVAASDSRLLTSTGLpglaaqirapaaaplgaplilNP 314

NGTYRDT+AK+ V C+EEWRR++AAS L + LPGLA QIRAP A LGAPLIL+

Sbjct 1084 NGTYRDTSAKNPVPACEEEWRRVIAASAENRLLAPALPGLAQQIRAPQATTLGAPLILSQ 1263

Query 315 RMTVPTTAASILSAQAAPTAAFDQTGHGMIFAPY-DYANYAAL-AGNPLLTEYADHSVGA 372

RMTVPTTAASILS QAAPT AF+ HGMIFAPY DYANYAAL AGNPLL EY DHSVGA

Sbjct 1264 RMTVPTTAASILSGQAAPT-AFENPAHGMIFAPYGDYANYAALAAGNPLLAEYTDHSVGA 1440

Query 373 IKQQRRLATNREHPYQR--ATVGVPAKPAGFIEIQ 405

IKQQRRLA NR+HPYQR A VGV AKP GFIEIQ

Sbjct 1441 IKQQRRLA-NRDHPYQRAAAAVGVTAKP-GFIEIQ 1539

**Conserved domains**

**STAR_dimer and SF1_like-KH domain-containing protein**


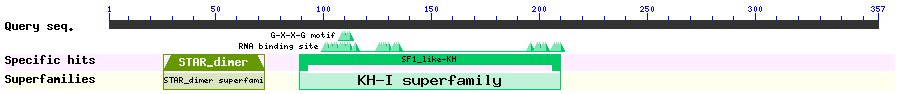


**ACO**

**>TRINITY_DN30096_c0_g1_i6 len=2626**

AAAAAGCAAATGCAACCTTGTACACTGATCGCACATGCACCTACGATAGTAACAAAACAA

ATACCAAACCGAGCCAAAAAATACCGGGTAGCAACAATTGCACAAACACAAAATACTGGG

ATAGAAAGTGTGCAGAATTTGACGGAAACGTTGAAAATGTTAAAACAAATATCTGTGGCT

GCTGCCCGTCCTCGTGTATGTGGTGCTTTGTGTTTTTGGAGCTAGTGTTGGGGACGATGT

TGTAGAGTGGAGTAGTGCGCCGCTTCGAGTTCAAAATTCTCACGCGTACAGTGCGTTCGG

TTGTTCGTTGGAACGTTCATAGTGTGTGTGAATTCATTGTATTGCGTGTCGCCAATGTTC

ATATTGCCAGTTCGCTATCCCCATTGCTACAGTTGTTGCCATCTTGTTCCGTCGCAGTGA

TCGCGGTCGCGGTTCAGTCCTCGTTTTCAATACTGTTGTTCCGCCGTAGTTGTTATTGGT

GTAATTTCGATGTTTGTTTATTTGCATTTGTGGAGCGAACTATCACCAAAGGCAAAATAC

GACAAAAAAACAATTTTCTACAGCGATATTGCAATAATTCAACAGTTTAAATAAAATCTT

TCCACCCACCAAAAGAACCAACGCAATATAAGGAGCGATAAGCAACGCATTGTCATATTC

AGCTACAATAGTCAGAGCAGCGATTAACAGTCTTGGCAGTGCAGTTGGGCAATAAAACTA

TTACTACGTAGAACAATTCCAAGGCAACAGCCAACAATTATTAAAACAATCATAGAACTT

CTTTGTCGCTGAGCCGCATCGACAGCGACCGACGATCGTCAGCGTGCATTGCAATTGGCA

TTATCAGAGTAGTAAATAAACAGCCAAAGCCAAAGCAATAAGTTAGCAACAGTGCCAGTG

TCAGTACCAATTGGTGATTGTAAACACAGAAATAGCAGCAAAGGCAAAAGCAGCAGACAA

CTACAGCATCCGCAGTAACACCAAGAACAAACCAAATAAAAAAACAGAAAAAAGAACAGA

CAGCAAAAATGTTGAAAACAAAAGCGAATGACGATAAAGTTGATACATTGCTGACCAAGG

GTCTGGTGCCGATAATTGATCTCGCTCATTGCGGGACCGAAGAGGCTCCAGTACGTTCGG

TTGTGAATCGTGTTGGACATCAATTACAAAAATGTTTGTCTGAAAAAGGGCTATGTCTGC

TTGTAAACCATGGCATATCCGATGAAAAGTTAAAGACCGCTTGGGATCATCTTGATGACT

TCGTCGATCTACCTGTCGATGTGAAGGAGACTTACATACGCACCGGTGATGATAACCACG

GTTATGTGCGTCCCGCCATGGAGCGTTTCGATGGCAAAACACCCGAACTACGCCATGCTT

TCAATATTTGCACATTAAATACAAAAAATCTACCCGAAGAACCATTACCCGGCTTTTCGG

AGCATATATCTTCTTTGGCACAGGATTTCAAAGCCCTCTCGCGTTTCATACTGCAGGCAC

TCGCCGTTTCGCTCGATATACCGCAATCGTTCTTTCTGGAGAAACACTCACACATGTTGT

CTGGTGATCATGATAACGAGTCTACACTGCGTCTACTCTACTATCCACCCATCATTGAGG

ATAAGGATGAGAATAATGACTTTATCAAAGGCAGTTGCATCTATAGTTATCAACGTTGTT

TGTCGGATAAGCCCGACTTCCGGCCAGAAACGAATCCGCGTGATGAAATCAATGAAGTGG

ATGGCAATGATGAGAGCAAGGACACCACCGAACGTCAGTTTCCTAACGGTGAAATAGTAC

GCTGTGGCGCACATACTGATTATGGCACATTCACACTGTTGGCACAGGACTCGGAGGGTG

GCCTTGAGGTGAAATTGCCGGGTACCGTGAAATGGCAACGTGTTGGTCATTTACCTGGTG

CCATACTGATAAACTGCGGCGAAATATTGTCGATATGGACGAAAGCACGGTATCCAGCAT

TGCAACATCGTGTTGTGGTGCCAGAGCAGCCACACATACGCACGCGTGGCCGCCATTCCA

TCGCCTTCTTCTGCCATCCAGATAACTTGACGATGATTTCGCCAAATGACCTGCCCAACA

ACGAAGTCGGCACAGACACAGTCGACAAGAAGCCACGCAAGAAGTCCTTCAAAGCGGCCA

AAGAAAAGGTCTACAACGCATACCAGTTGATACAGAAGAGATTCCGGGAAACCTACGGCC

ACCAAAATTCGCACTGATGATCACAAATACACACACAAACAAACAACTTAGCTTATAGCT

CATAGCTACTACATATATACTTACTTACGTACATACATTAGTCCATAACTATTTTAGGTG

AATAAATGTTGGTACTGACTTCAGTTGTAAATTCGTTGCGGAGCCAGAGGCAGTAGACAT

GCTCGCAAATGTCGTATGTTTTTCTGTGGAGAAACCAGCATCGTTTGGAGAGATGTTGTG

ACGAAAATACAGTGCAGTTGTTTTTGTCTTCGCTATTAAGTTTAATTTATTTAGTATATT

TTTCCTTAAATATTTGTATATTTTAAACACAATTTGTAATTATAATTGTATTTGTATTTG

TATTTATACACATCGTTTTAATTATTATTATTTTACCCTAGTTAAT

**tBLASTn(First hit)**

Score = 753 bits (1944), Expect = 0.0, Method: Compositional matrix adjust.

Identities = 369/402 (92%), Positives = 386/402 (96%), Gaps = 0/402 (0%)

Frame = +3

Query 1 MLKTKANDDKVDTLLTKGLVPIIDLAHCGTEEAPVRSVVNRVGHQLQKCLSEKGLCLLVN 60

MLKTKANDDKVDTLLTKGLVPIIDLAHCGTEEAPVRSVVNRVGHQLQKCLSEKGLCLLVN

Sbjct 1029 MLKTKANDDKVDTLLTKGLVPIIDLAHCGTEEAPVRSVVNRVGHQLQKCLSEKGLCLLVN 1208

Query 61 HGISDEKLKTAWDHLDDFVDLPNDVRELYIRTGDDNHGYVRPGVERFDGKTPELRHAFNI 120

HGISDEKLKTAWDHLDDFVDLP DV+E YIRTGDDNHGYVRP +ERFDGKTPELRHAFNI

Sbjct 1209 HGISDEKLKTAWDHLDDFVDLPVDVKETYIRTGDDNHGYVRPAMERFDGKTPELRHAFNI 1388

Query 121 CTLNATNLPEEPLPGFAEHISSLAQDFKALSRFILQALAVSLDIPQSFFLEKHSHMLSGD 180

CTLN NLPEEPLPGF+EHISSLAQDFKALSRFILQALAVSLDIPQSFFLEKHSHMLSGD

Sbjct 1389 CTLNTKNLPEEPLPGFSEHISSLAQDFKALSRFILQALAVSLDIPQSFFLEKHSHMLSGD 1568

Query 181 HDNESTLRLLYYPPIIEDTDDKNDFVKGSCIYSYQRCLSDQPDFRPESNPRDEINELDGN 240

HDNESTLRLLYYPPIIED D+ NDF+KGSCIYSYQRCLSD+PDFRPE+NPRDEINE+DGN

Sbjct 1569 HDNESTLRLLYYPPIIEDKDENNDFIKGSCIYSYQRCLSDKPDFRPETNPRDEINEVDGN 1748

Query 241 EENKNTTVRQLPNGEMIRCGAHTDYGTFTLLAQDSEGGLEVKLPGSEKWQRVGHLPGAIL 300

+E+K+TT RQ PNGE++RCGAHTDYGTFTLLAQDSEGGLEVKLPG+ KWQRVGHLPGAIL

Sbjct 1749 DESKDTTERQFPNGEIVRCGAHTDYGTFTLLAQDSEGGLEVKLPGTVKWQRVGHLPGAIL 1928

Query 301 INCGEILSIWTKERYPALPHRVVIPEQQHIRSRGRHSIAFFCHPDNLTMISPNDLPNTEA 360

INCGEILSIWTK RYPAL HRVV+PEQ HIR+RGRHSIAFFCHPDNLTMISPNDLPN E

Sbjct 1929 INCGEILSIWTKARYPALQHRVVVPEQPHIRTRGRHSIAFFCHPDNLTMISPNDLPNNEV 2108

Query 361 GTDTVDkkprkksfkaakekVYNAYQLIQKRFRESYGQQNSH 402

GTDTVDKKPRKKSFKAAKEKVYNAYQLIQKRFRE+YG QNSH

Sbjct 2109 GTDTVDKKPRKKSFKAAKEKVYNAYQLIQKRFRETYGHQNSH 2234

**Conserved domains**


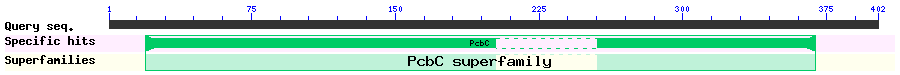


**Scavenger receptor**

**>TRINITY_DN31545_c2_g1_i7 len=4182** CGACGACTACAGCTAGCAATGCTCCCGAACGCCACTGGCCAGAGTAAACACAAAGGCAAT

TCAACAGGCAGCAGCAGGCACAGTCGTTATCAAGCGTTTGACGCGTCTAGAACACGCTCC

TCGGTAATAATACAGATTGTACGTACATTTGTATATAAGTAATTTACGAAACATTGAATT

TATACAAAGAAGTCGCACACCAACCTAAGATACGGAAACAAAAATCCGTCCGTTTATCAA

GTGAAATATTAAGAATAAAGGAAACAGGAGCCGGAGTTCGATTCTATGAAATCATAAATG

TAGATCGCGTGTTGGTGAAATTCCTTACCATGGTTTGAATGCTATGCTTTAAGAAGTTCG

ATTACTGCATCTAGGCGCCAAAAAGACAATACCGCAATCAACTACGAAAGATAAGAAGGT

CAAAGTTCGGTGCGTTATATACGAAATAAATACTTATGTATGTACGTTTATGAGTAAATA

TCAGAAGTGTTTTAAATTTTGAACAATTGTTTCGGCTAATACATACAAACATATATTTAT

GCAGTTAATCTAAAAACAAAACTAATACTAAAAACAAAACATCTGGATCTAATAATTGTT

TACACGCGCCGTTCTTGGCTTTGATGACCGTTCCTTTGAAATATTATATATTTTACTATT

TTTTAATAAATGAGAGCAAAGGCTATCAGCCATCGAGTGTGGAACCAAATCAAGTGCAAT

ATTTAAACGCCTGCATTCAACCGCCTGCCATACTTTTTTATTTCGTTTAAAAGCCAAACA

AATATTTCGAAATGCCGAAGAAGGCAGATAGTCTGAAAAAATGGCAGCAAAACAATCGCA

AAGTGATTGTTGGCATTGTTGGTTTTTGTTTGAGTATTTTTGGCGTTTTGTGCGGCATGT

TTTGGGAGCAGATCTTCAATGGCATAATGGAGAAGGAAATGATCTTACGCCCTAACTCAG

AGGTCTACGACAAGTGGAAAAGTCCTCCCATGACTTTGAGCTTAGACATTTACCTCTATA

ATTGGACGAATCCCGAAGATTTCAAAAATCTCAGCACAAAACCGAATTTCGAGCAACTCG

GTCCTTATCGTTTCACCGAAAAGCAGGATAAAGTTAACATACAGTGGAATCCGGCGAATG

CATCGGTGACGTATCGCAAAAAAAGCGATTTCTACTTTGATGCGAAGGGTAGTAATGGCA

GTTTGGATGATGTGATAGTCACCTTGAATGCTGTAGCACTGTCCGCTGCCGGTAAGGCTA

AACGTTGGAACCCTGTACGACGCAATTTGGTGGATGTGGGCTTAAAATTATATGGCCAAG

AAATATCTATAACGCGTACAATCGATGAGATGCTCTTTACTGGTTACAGCGATGATATGT

TGGATATGGCGCGTGCCATGCCACTCTTTGGCAAGGATGTTGAGGTGCCTTTCGATAAAT

TTGGTTGGTTTTACACGCGAAATGGCAGCACCGATTTAACTGGCGTCTTCAATGTATACA

CTGGCGCAGATGACATTTCGAAAATCGGACAGATGCACACATGGAACTACAAACACCATA

CTGGCTACTTTCAATCGACTTGTGGACTCGTTAACGGTTCAGCGGGCGAATTTCATCCAC

CATATTTAAAGCAGTATGGCAGCGTATCATTTTTCACGCCCGATTTATGTCGTACAGTGC

CATTGGATTATTTGGAAACCGCAGAGATAGAGGGCTTGCTTGGCTACAAGTACCATGGTG

GAGTGCGATCTGTCGATAATGGCACCCTATATCCTGAGAACACCTGCTTCTGCGGCGGTC

AATGTGTTCCTTCAGGAGTTATGAATATTTCATCCTGTCGCTTTGGCTCGCCTGTGTTCA

TGTCCTTTCCTCATTTCTACAATGCTGATCCGTACTATGTGCAACAAGTTGATGGCATGC

AGCCTGAGCAGGATAAACATGAATTCTATATGATTTTGGAGCCGCGTACCGGAGTTGCTT

TGGAAGTGGCAGCGCGTTTCCAAGTCAATATGCTTGTGGAGCCCATCAAAGGCATAACCC

TTTACGAGAATGTGCCGCGCGTCTTCTTTCCATTGATCTGGTTTGAGCAGAAGGTACGCA

TAACACCCGATCTCGCCGCCGATCTGCAACTGTTGCCGAAAGTTCTCATGGGTGGCCAAA

TTTTCGCCTGTATTTGCTTTGGTGTTGGCTTAATATTGCTTTGTTGGTATCCCATCGAAT

TGCTTTGGACGCGTCAGCGATCTGTTGATCTCAAATGCACACCGACAACAGTGGAAAATG

GCAAACAACTAGCGAAATTTTCCAGCGTCGAAGAATTGAAAAATCGACCACCACCTGTTG

TCACGACGAATAATAACAAAACGCTTGACAGCTCGCCTTTGTTGGAGCGCGGCAATGGCA

AGAGCGCCACAATTGTGTCCAAGTCGGAGACAAATGAAAGTGTTGCCACTGACAAAACGG

CTGTAAGCAACGTCGCAAAAGAGTAATAAAGCGAGGGAAGCGTAGTGTGTTTTTTGCGGG

AATTTCCCAAATAATCGTCCTATTGTACGCCTTTTTCCATTAGAATTAGTAGTTCGTAAT

TAAACTTTAGTTTTTTGTTAAATTTTATACAGCGTAGTTATGTAAGTGCAACTGGTGATC

TAGCAGAACAGTTGGTTACTCAAATTTCTATTTGATAATTGCCATTAGATTGTTAATTAT

AGTTATGGAAATTTGTAAACGGAATACTCCAGTTAAATGAAAACGAATGCGTTTTGTATA

AAGCGAGCGATTACTATCTAAACCTGTAAAGACAATATAAATATAAGAAATCAGCAAAGT

GAATTTGTATTTGTAACGAAATATTTTAATTTTACTCATACTTACCTATATGAGAGCAGA

TATGACAGGGAGCAAATATGGAATAAAATGTAAGATTTGTTACGGTTTTATTCAAATGTT

AGTGGGTAACAATCGGCATTGAATGGCAATATGTTTAATGATAGTAGCTAGTTTTTACAT

ACCATATATACAAAAGTTTAGAGCAAAAGAGATGTGATATATTAAATGTCTAAACACAAA

AGTGGCCTCAAATCAAAGTAGATATTACCAGCAATAAGTCTACCAAAGTATTTTTTATAA

GAAAAAAATGATTGCTAGTGCGATTTTGTACTTTTATATGAGACAAAAAAATATTTTCTT

ATAAATGACAACATTTGCTATTCTCAAAATAATATAATAATGCATATACGTATGTAAGTA

TGAAAAAATATGGTAAACATTAAACCTACAAAATATCGCCTAAATATAAAATTACATTCC

TTGATGGCTAGCACTTTTGAGCTGAAAAATAGGTACATTTACGAAGATATGTAAATGCAA

AGTTAAATTTTAAACTCAAAATTAAATTTTATTACATTGTAATAGAATCCCGCAAGAACG

GAAACCGTTAAATGGACTTCAGGATGCATAAAATATACAACTACTTTAGTCATAGTTAGT

TGTACTTTAGAATTCGTTACATATACAGTACCGGGCAAAATAATAGGCACAATAAGCTTT

CCTCATTCATTTAAGGAATTACAACATTTGCGTAATGCTCACCGTTTTCGTTTCAATTAC

ATGGTATTCATCCTTTAATTCAATTTTGAAACCTAGTTTTATAGTTATTATATTTGATAC

GAGAAAGGTGATAGAATCTAAAATGTTAAAAAATTGAGTTTTCATGAGGACAAAAGTATA

AGCATAATTTTTCATTCTTTCCCCATTTCTAACACTCAATCTTTGTTATTTTTGATTTGA

AAAACTTGATTCCATTGTCGTTATTGTGCTTATATTTATGTCCGTCACTGTACTTTACAA

TAGTTAATTTCAAATTATATCCATCGATCGGGTTCTAAAGTACAAAGGACTTATCTGAAG

TTTACTTAACGCTTTCTGAATGACAATTGAATTTATTGCAGTGAATTTGTCCGTTTTTGC

GGTAGAGTTGAAGCAGGTGTACATAAAATATTTCTCAAAAATTTAATCTCATTTAAATAA

ATATTTTTTTGCATTCGCTCAATCGAATATGTTTAGGATTGCCTTTTACTCGCCGTAAGT

TTTAACATTTGCTGTTTGTAATTACTTTGTAACTTACTTACTTTTAGTTATTATTTTAAA

TTTTCTTAGAAATAAAGACAAATAAATGAATTTATATATAAG

**tBLASTn(First hit)**

Score = 717 bits (1852), Expect = 0.0, Method: Compositional matrix adjust.

Identities = 364/552 (66%), Positives = 447/552 (81%), Gaps = 6/552 (1%)

Frame = +3

Query 11 QKSNRKliigifgfclglfgilcgMFWVDLFDWIMHKEMALAPDTRVYENWKSPPIDLSL 70

Q++NRK+I+GI GFCL +FG+LCGMFW +F+ IM KEM L P++ VY+ WKSPP+ LSL

Sbjct 825 QQNNRKVIVGIVGFCLSIFGVLCGMFWEQIFNGIMEKEMILRPNSEVYDKWKSPPMTLSL 1004

Query 71 DIYLYNWTNPEDFGNLSTKPILEQVGPYRFIERPDKVDIHWHPENASVTYRRRSLFYFDA 130

DIYLYNWTNPEDF NLSTKP EQ+GPYRF E+ DKV+I W+P NASVTYR++S FYFDA

Sbjct 1005 DIYLYNWTNPEDFKNLSTKPNFEQLGPYRFTEKQDKVNIQWNPANASVTYRKKSDFYFDA 1184

Query 131 AGSNGSLDDEIttlnavalsaaataKYWPPVKRSLVDVGLKMYGAEMSVQKSIDELLFTG 190

GSNGSLDD I TLNAVALSAA AK W PV+R+LVDVGLK+YG E+S+ ++IDE+LFTG

Sbjct 1185 KGSNGSLDDVIVTLNAVALSAAGKAKRWNPVRRNLVDVGLKLYGQEISITRTIDEMLFTG 1364

Query 191 YNDAMIDVAMAMPIFGDEVKVPFDKFGWFYTRNGSADLTGVFNVFTGADQLAKLGQMHSW 250

Y+D M+D+A AMP+FG +V+VPFDKFGWFYTRNGS DLTGVFNV+TGAD ++K+GQMH+W

Sbjct 1365 YSDDMLDMARAMPLFGKDVEVPFDKFGWFYTRNGSTDLTGVFNVYTGADDISKIGQMHTW 1544

Query 251 NYQENTGFFDSYCGMTNGSAGEFQPQHLKPGDSVGLFTPDMCRTIPLDYVETVDIEGLEG 310

NY+ +TG+F S CG+ NGSAGEF P +LK SV FTPD+CRT+PLDY+ET +IEGL G

Sbjct 1545 NYKHHTGYFQSTCGLVNGSAGEFHPPYLKQYGSVSFFTPDLCRTVPLDYLETAEIEGLLG 1724

Query 311 YKFSGGPRSVDNGTQYPENLCFCGGQCVPSGVMNISSCRFGSPVFMSYPHFFNADPYYPD 370

YK+ GG RSVDNGT YPEN CFCGGQCVPSGVMNISSCRFGSPVFMS+PHF+NADPYY

Sbjct 1725 YKYHGGVRSVDNGTLYPENTCFCGGQCVPSGVMNISSCRFGSPVFMSFPHFYNADPYYVQ 1904

Query 371 QVEGLSPNQKDHEFYMVVQPSTGIPLEVAARFQVNMLVEPIQGISLYTGIPRIFFPLVWF 430

QV+G+ P Q HEFYM+++P TG+ LEVAARFQVNMLVEPI+GI+LY +PR+FFPL+WF

Sbjct 1905 QVDGMQPEQDKHEFYMILEPRTGVALEVAARFQVNMLVEPIKGITLYENVPRVFFPLIWF 2084

Query 431 EQKVRITPDMADQLKVLPIVMLSGHIFAGICLIVGITLLCWTPVQILLASCRNRRYDLRT 490

EQKVRITPD+A L++LP V++ G IFA IC VG+ LLCW P+++L R R DL+

Sbjct 2085 EQKVRITPDLAADLQLLPKVLMGGQIFACICFGVGLILLCWYPIELLWT--RQRSVDLKC 2258

Query 491 KTKTNGQYKSRSQFSSAEELKSKASTLVCEKSVKGSPDSSPLLEK--GRKPTII-KSQTG 547

T K ++FSS EELK++ +V + K + DSSPLLE+ G+ TI+ KS+T

Sbjct 2259 TPTTVENGKQLAKFSSVEELKNRPPPVVTTNNNK-TLDSSPLLERGNGKSATIVSKSETN 2435

Query 548 ESVATASTAISD 559

ESVAT TA+S+

Sbjct 2436 ESVATDKTAVSN 2471

**Conserved domains**

**CD36 domain-containing protein**


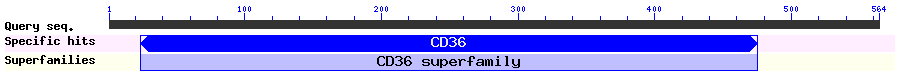


**Eater**

**>TRINITY_DN33643_c4_g2_i2 len=5001** CTTCTACAATAAATCCCACTGAATCAGGTAGTGAAGTTATGAAGTCGACTACTGTGGGGA

GAGCGGGTCGAATGTTTGCCGGCTTCGCCAATCCGTCCGACGAGGAGGATAGTGAAGAGG

ATAATAGCGATAGCTCGGGAGATTTTAGTGGTACCGACATCGGATTCGAATTGAGATGCA

TTCGACGCTGTACAAACGGAACTTGCTACAAGAACGAATGCATCTGCGAGGAGGGTTACG

TGCTGCAGGAGCACATTCATGAGGATATCTGTATTGCACGTTGCGGTGAATTGATTGGCA

ATAAGTACACTGGATGTGTGAATGGTTTTTGCAAGGAGCCCGATGTTTGCATCTGCTTCG

AAGGATTTCAGCGATCTATAGCAAATGAGTTCAAATGTGTGCCGCCGCACAACGAGGAAC

AACAAGCGGACGGGATTTTCAAATATCTACGGTTGTACATTATTTTCGCAGCCTTGTCTT

TGCCACTGGCTTTGTTAGTGTTGTACTTACTGTGCCATATAAGCTGCGAAAAGTCGTACA

ATGTAGCGAAAAGAGAGAACCAAGTGGGACTGGCGCATCTATCGCGCAATGTCGACATCA

TAAGAGTGCCTTAGTAAGGTAGTATTAGCTATATAAAATGCGCGTAGGTGCGTATGTATG

TATGTAGTAAGAATATCTCACATTTGATGCTTTAAAGTGATGACAGTGATCTTCAATTCT

TTATTTTTGTGTATATAGTTAAAGAGGGATATATGGATATAAATACTTAGAGGTGATAAG

GAAACGCAATATATTATATTTGTATGTATGTATAGGTTAAGAGTAATTATAATTTTTAAT

GCATTTATTAAGATACATGGGGATAATGAAAAATAAGTATATTTTGAGTTTGTTATTTTG

TTAAAAAAAATCTGAAAGCAAATTAATGGAATTTCAAAAATATGGGAGTTTCTTTCAATA

CATGGAAGAACGGAATGCTTGAAGCTGAGTTATTATACAACAAATTTTATTCATAACAAC

ATTCCAACACATATATTACGAATTGTTCTGTAATTGTATTTGTAATCGTGTTATACAGAC

TAAGGACTCTAAAATTAATTGTCAATATAAGTCACGAAACTTTGGTTTTTTATTTCGAAG

TTTTTCCTTTTTTGAAATTTAGGAATTTTCGAAGCTTTTGTTTTATATTCCGAAAACCGT

ATTAAAAGGACCTAATTTTGTTTTACATTTTGGGTTTTGCGGAATTTTCAATTTCCTTCT

CATTCAATATTTGTGGCGCTTCATATTTTGTGACACAGAAAAATTCTTGGATATTCCCAA

TACCATTTTTGCTGGACTGAATATTTTGCTAAAATTTCGTATTTATGCCTTCTTCGTATT

GTACTTTTGGGAATTAATAAATTTTTATTTGCAAAATTGCTTTGAAATTTCAGAATTTTC

GAAATACGAAAATCTTCATTTATTTTTAATGCACTCTTCATATATAGAATTTTCGGGAAC

TAACACAAGTTCAGAGGTTCGAGATTTTATATTTTTAAATTCAACCTTCATCCACTCAAT

TTTTCCTACTTTTTCCGGAAGTTAATGCATTTTTTAATTGTGGGGTTTTTTTTTTTGGAA

ATTCCCGGATTTTCGAAGTTTTTTACTAAATTCTGAAACCCGTATTAAAGGGACCTGAAA

TTATTTTTTTGTTTTGTAATTCAAAAAAAGTCCAGGGCACGCGAGAATTTCAATTTATTT

CTAATTTAGTGTTTTCGAATATTATTTTCATGAATTTCATAATATTTTCAATACAAATAT

TTGTTTTCAATTGATCTTCATTTCTCAAATTATTACAGGAATCCCGAATTAGTACCGTTT

TTCAGGACTACAAAATTTTTTAAATTGCATTTATTGGCGATCATCTTCGAAATTCTGGCG

TTTTATGATTTTTAGTGCGCTCTCGGTAGATAGGCTCATTCCTTTTTCCGGAGCAATATA

AAATATAAGTAGAATATATCTTCTAAATTCAGCAAAAGTTGTTTTTATTAGCAATGTAAG

CATTTAAACATATCCGAAAGTAAAAAAATTAGAAATGTATTAAATTAACAAAAAAATATT

CTTTAGCAAGAAATGACTAAAGATTTATTTGTTGGTGTATGTATTTATTTAATTTTAAAA

GGCTATGTATTTATGAAATATTCCTTTTAAGACCATTTTTTTTTATTTTTATTTTATTTT

CAAGTAAGCTAGAAGTAAAGGTTTTTTTTTTAACTTTTTTTCATAAAGTTATAATATATT

TTGTTGTATACATCAACTGTAAATTATAAAATTTTTGAATATCACATCGGCGCTTGTCGC

GCTGACTTTGCTTCACAGCTGTAAATTTTATCGAATTGGCTGTCTATGTATGAGTGTGTA

ACTAAAATTTACGATACTTCGCTCCAAAGCTCAACTCTCGTCTTCTTCCTGCATGTTGAG

GCCGTCCTCTGCGCCTGCGCCGGGCATTACTACGCCGAATGTTGGATTTTCTATCAAGCG

CACGCCATGTTTCTCCCGGAAACGCCTGCGCGCATATTCCCGATAAACCAGCACCAAAGC

CAGTGCACACAAAGCTATCAGCAACAAAGACATTGCACCGATGGTCCAGCGTGAAACCGA

GACTCCACTCAATACCAGGTGATCCTCTATAGACCCCAACTCTGATACACATGCGCCACG

CCGCTCATTGTAGATCTTCCCCTGCACACACTGGCATATACCATTGACATCACATTTTCC

ACCCAAACAGTTCCATTCGTTGCAATAAGCCACACACACTGGCAGATCGGTGTCGTTGCT

GAGTGTGAGACGATAGCCCAGATCACAACTGCATGCGTTTGGCCCAATACACCTGCCATG

GCCACAGTTCTGATCGCATATCGGCGCACACTCTGTGCGACTACCGTTGAGAAAGTCGTA

TCCTTCCAAGCAAACGCACTCATCTGGCCGCATGCACTCGGCATTAATGCAAGTGTCTCT

ACAGATTGCCCGACAACGATGCGGTTCGCTAGAGTTCATCACGCGATAACCATCGCTACA

TTGGCACTTATTAGGCGCCACACACATGCCATTCTCACACGATGGCCGGCAATGAGCGAT

GCAGAGGCTTCTGTGACGCGTTTCCTCATATCCGCTCCAGCATTCGCACATATCCGGTTC

AACACAATTACTGTTGATACATGCCGGCAGGCAGATGGGATCACAGATCGCGTTCGAGCC

TTTGCGTAGCCGGAAACCTTCATAGCAGCGGCATATACCGCCCGCCACACAAATGCTATT

GACGCACTTTTGGGCACAGTGCGGCTCGCAACGTCCCGTCAACCATTTCTTGGCGTAACC

TTCTTCGCACTGACAAACACCCGGCGCAATGCATTGACCATGCCCGCAGTCAAAATTGCA

AACGGGCTCACACTCATGTGGGCGTTTTGTATTCGGGCGATATCCGCTGAAACAGCGGCA

TGTGTCCGGAGCCGTGCAGGTGCCATGCACGCAAGAATGCGTGCAAACCGGTATGCAGAG

GTAAAAAAGGAGTGATTGGTAACCGGGTAAGCAGCTGCACACACCTGGACTAACACAATT

GCCATTCTTACAGCCACGCGGACAATGCGGCTCACACTCCGCGCGTGAACCATTCTTAAA

AATGTAGCCCTCGTTGCAAGTGCATGTGTCCGGCGCAGAACAGTGTCCATTTATACACTC

ATCCTTACAAATTGGCTGACAGACATGCGCTTCCACTTGCTTGTACCCTTGGTGGCACTC

GCAGCGTCCCGGTTCATTACAGAAGCCATTCTGACAGCCGCGTGCACAAGTCGGTTCACA

TTCCGTGCGCGAATTGTTGCGGTAGATATGCCCCTCCAGACAGGTACACTGATTAGGCGC

TGTGCAATGCGCATTCACGCACTTCTCCTTACAACTCGGCACACAGGCCAGTCCGAGTAG

GTTGTAGTAGCCCAACTGGCATTTGCATACATTCGGTGCGCTGCAGTACCCATTCGAACA

ACCGCCCTGGCATACAGGTTCACACTTGCCAAGCGTGCCCAAACGCAACTCAAAACCGCT

ATGACAGTCGCACTCGTTAGGCGCTTTGCAAATACCATTCTCGCATGGCTCCTCGCATAC

AGGTATACAGAGGCCCAGCGTTTCATTGCGCTCATAACCGAAATTGCAAGTACATTTTTC

CGGCGAGTCACAGCGTCCATTTGTGCAATCGATCAGACACTTAGTTTCACACTCGGTTAT

ACTGCTGTTGATGAAACTGAAGCCCTCGTTGCAGGTGCATGTGTCTGGCGCGATGCACTG

CCCATTGATGCAGGGTTCACTACACTCCGGCTCGCAAATGGTGCTGTTCTTCTGGTTATT

ATAGCCGGCGTCACATAGGCACACATCTGGACGCACGCACTTGCCCGTCTTGCAGTCCGC

GCACTTCGGCTGGCAATCACCGCTTTTATTATCGCGAACGTACCCATCACAGCAAATCTC

ATCCGTATCCGTGTAACTCTCTTTCACCGTACGCAGAACCAGTGGATTATAGTTGGGGGC

GCAATTACTTGTACAGTTTGCATCTACCGGAAATTTTCGCATAGTCACCAGCGACACGCG

CGTCTTGATTTGCGGTACTTTTACAACGCATGCTGACACAATCACCACATGAAGCAGAAG

ACTTCCATAAAACTGACAACTTCTATGTATTTTCATTATTATAATTTTTATTTTAATAAT

TTGCAACAATCACTTCAAGTTTTAATCCTTTAGAATTGAGTTGCGTTGTCTGAATATTTT

TGAATTAGCGCCCGAATTCTCAATTCAAATTAATCACTTTTAATTTCCATCAAGCACTGC

TTGCCATAATAGCAACCACTTGCAGTCTTGCTACTCCCTGCCTGAATGAGTAAATCTGTT

TAATTGCTAAATTTAATGATACCCATTTGTAGGCGAGCAATACTAAGCGGCGACAATCTT

TATCGGCTTATCAACGCTAAG

**tBLASTn(First hit)**

Score = 370 bits (949), Expect = 6e-107, Method: Compositional matrix adjust.

Identities = 229/562 (41%), Positives = 305/562 (54%), Gaps = 24/562 (4%)

Frame = -1

Query 270 CQNGVCVAPNECSCNAGY--TKLEGVCTPVCKDGCVNGFCASPEKCSCNDGYEM--DSEN 325

C+ G CV P+ C C+AGY K +C P C + C+NG C +P+ C+CN+G+ S

Sbjct 4434 CKTGKCVRPDVCLCDAGYNNQKNSTICEPECSEPCINGQCIAPDTCTCNEGFSFINSSIT 4255

Query 326 RCSPVCSGGCKNGFCVAPGKCSCDEGYIKG-TGNSCKPICSKGCENGFCDAPEKCSCNDG 384

C C C NG C +P KC+C+ GY + T C P+C + CENG C AP +C C+ G

Sbjct 4254 ECETKCLIDCTNGRCDSPEKCTCNFGYERNETLGLCIPVCEEPCENGICKAPNECDCHSG 4075

Query 385 YEMD--GENRCSPVCSGGCKNGFCVAPGKCSCDEGYSKETGNSCKPICSKGCENGFCDAP 442

+E+ +C PVC GGC NG+C AP C C GY G +C P C + C N C AP

Sbjct 4074 FELRLGTLGKCEPVCQGGCSNGYCSAPNVCKCQLGYYNLLGLACVPSCKEKCVNAHCTAP 3895

Query 443 EKCSCNDG--YEMDSENRCSPVCSGGCKNGFCVAPGKCSCDEGYSKETGNSCKPICSKGC 500

+C+C +G Y +S C P C+ GC+NGFC PG+C C +GY + + C+PIC C

Sbjct 3894 NQCTCLEGHIYRNNSRTECEPTCARGCQNGFCNEPGRCECHQGYKQVEAHVCQPICKDEC 3715

Query 501 ENGFCDAPEKCSCNDGYEMDGENR--CSPVCSGGCKNGFCVAPEKCSCDEGYSKETGNSC 558

NG C AP+ C+CN+GY +R C P C GCKNG CV+P CSC GY C

Sbjct 3714 INGHCSAPDTCTCNEGYIFKNGSRAECEPHCPRGCKNGNCVSPGVCSCLPGYQSLLFYLC 3535

Query 559 KPICSNGCENGFCDAPEKCSCNDGYEMDGE--NRCSPVCSGGCKNGFCVAPGKCSCDEGY 616

P+C++ C +G C AP+ C C GY + + + C PVC+ C +G C+APG C C+EGY

Sbjct 3534 IPVCTHSCVHGTCTAPDTCRCFSGYRPNTKRPHECEPVCNFDCGHGQCIAPGVCQCEEGY 3355

Query 617 SKE-TGNSCKPICSKGCENGFCDAPEKCSCNDGYEMD--SENRCSPVCSGGCKNGFCIAP 673

+K+ C+P C++ C N C A C C +G+ + S C P+C C N C+ P

Sbjct 3354 AKKWLTGRCEPHCAQKCVNSICVAGGICRCYEGFRLRKGSNAICDPICLPACINSNCVEP 3175

Query 674 GKCSCDEGYSKETGNS-CKPICSKGCENGFCDAPEKCSCNDGYEM---DSENRCSPVCSG 729

C C GY + S C C CENG C AP KC C+DGY + +RC +C

Sbjct 3174 DMCECWSGYEETRHRSLCIAHCRPSCENGMCVAPNKCQCSDGYRVMNSSEPHRCRAICRD 2995

Query 730 GCKNGFCVAPGKCSCDEGYSKETGN--SCKPICSKGCENGFCEAPEKCSCNDGYEMDGEN 787

C N C+ P +C C EGY G+ C PIC + C +G C P CSC+ GY + N

Sbjct 2994 TCINAECMRPDECVCLEGYDFLNGSRTECAPICDQNCGHGRCIGPNACSCDLGYRLTLSN 2815

Query 788 RCS-PVCSGGCKNGFCIAPGKC 808

PVC C C+ GKC

Sbjct 2814 DTDLPVCVAYCNEWNCLG-GKC 2752

**Conserved domains**

**
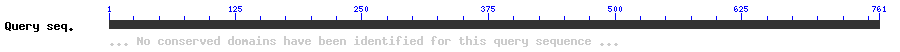
**

Clathrin Heavy chain (chc)

**>TRINITY_DN29160_c0_g1_i4 len=6606** GGAGTTTCAGTTGTCAGAAAGAATTTTTCTTGTCATTTTCGCTTTTCGGTGGTGGAGAAA

TCAATATGATTAAACGCGTTTTAAGTGCAATTTAAGTGAATTTTAAATGCAATCTTAACC

AATTAAGAGACAAAGAACGTTACAACAGTAAATGTGTTTTTGTGGACAAAACGTAGTGAT

CGAGTTGAAAGAATTGTTGTAAATGGATGTATGTTTTGTAAGACCGAAAGTTGAACCTGA

GAATTTGAATCAATTCTAAGAAGTAATTTGAAAATCTTTATTAAAAAGTATTATAAAATA

GCAGTGATTACATTGAATTGCCAGCTTTATGGCATACAACTAATTGTGCCGCTGCGTATT

TAGAAATCATTATTTTGCTCATTCAATCATTTTGTTTTTCTTGTTTTCTCATTTTCACTG

CGACTTCAACAGAATAAGGTTCACATAAAAGAATAAATAAATAAAGTGAAAAGTGCTAAG

TAATAGCAGACAAGTAAAAAATTGAAAAGCGAACGAAAAAGCGGCCGAACGAACGAAGCA

AAAATTTAAAAGACGCATTGCTGTTCTGCTCGTGATTTACCTGAAGAATCAGTAAAATCT

TAAAGAAATAATTAAAATAACGATAGCAACTAGTCACGCACTGTGAACCTAAAACAGCAA

TAAGAGAAGCAGTCAACCACATCTTTAGCTTGATTGCATCGCTGTTGCAGTGAAAATTCG

TTAGTGTGTTAAAAGTGTTGCAGCAGCAAAATGACGCAAGTTTTGCCAATACGCTTCCAG

GAGCATTTACAGCTCACAAATGTCGGCATCAATCTCAATTCGATTTCGTTCAGCACTCTC

ACCATGGAGTCGGACAAATTCATTTGTGTGCGTGAAAAGGTCAACGATACCGCACAAGTG

GTCATTATCGACATGAACGATGTCAGCAATCCGACCCGGCGTCCCATCTCTGCCGACTCG

GCAATCATGAATCCAGCCAGCAAAGTCATTGCGCTCAAAGCTCAGAAAACCTTGCAAATT

TTCAATATTGAAATGAAATCGAAAATGAAGGCGCACACAATGACCGAGGATGTTGTATTT

TGGAAGTGGATTTCTTTGAATACGTTGGCGCTGGTCACCGAAACCTCCGTCTATCACTGG

TCTATGGAGGGCGATTCGACGCCGCAGAAAATGTTTGATCGTCATTCGTCGTTGAATGGT

TGTCAAATTATCAATTACCGTTGTAATCCATCTCAACAGTGGTTGTTGTTGGTGGGCATT

TCGGCATTACCGAATCGTGTTGCAGGCGCTATGCAGCTGTACTCGGTGGAGCGCAAGGTG

TCGCAAGCTATTGAAGGTCACGCCGCCTCATTCGCCAGCTTTAAGATGGAGGGCAACAAA

GAACCCTCAACGCTATTCTGCTTCGCTGTGCGCACCGCTAGCGGTGGAAAGTTGCACATT

ATTGAAGTAGGAGCTCCACCAGCGGGCAATCAGCCTTTCCCGAAGAAGTCGGTGGATGTG

TTTTTCCCACCAGAGGCACAAAGCGATTTTCCCGTCGCCATGCAAGTTTCCGCCAAATAT

GATACCATTTATTTGATCACCAAATATGGTTATATCCATCTGTACGACATGGAAACGGCT

ACGTGCATCTACATGAATCGTATCTCAGCGGATACGATTTTTGTGACGGCTCCGCACGAG

GCCAGTGGCGGCATAATCGGCGTAAATCGCAAGGGTCAAGTGCTTTCGGTGACCGTTGAT

GAGGAACAAATCATACCTTACATTAATACCGTATTGCAGAACCCGGATTTGGCATTGCGC

ATGGCCGTCCGTAATAACCTCTCTGGCGCTGAAGATCTGTTTGTGCGCAAATTCAATAAG

CTTTTCACAGCTGGCCAATATGCGGAAGCAGCCAAGGTTGCTGCACTGGCACCAAAGGGC

ATATTGCGTACACCACAAACCATACAACGTTTCCAGCAGGTGCAGTCGCCCGCTGGCTCA

ACGACGCCGCCATTATTGCAATACTTTGGCATACTGCTGGATCAAGGCAAGCTCAACAAA

TATGAATCGTTGGAGTTGTGCCGACCGGTGCTGGTGCAGGGTAAGAAGCAACTGTGCGAG

AAGTGGCTGAAAGAGGAGAAGCTCGAATGCAGCGAGGAACTGGGTGATTTGGTGAAGACA

TCTGATTTAACGTTGGCGCTTTCGATCTACCTGCGTGCCAATGTGCCGAATAAGGTTATA

CAATGTTTCGCTGAGACGGGTCAATTCCAAAAGATCGTGCTGTATGCTAAAAAGGTCAAC

TACACGCCCGATTATATTTTCCTCTTGCGTTCAGTAATGCGCACCAACCCAGAACAGGGT

GCTGGTTTCGCTTCAATGTTGGTGGCCGAAGAGGAGGAGCCACTCGCGGACATAAACCAA

ATTGTGGATATCTTTATGGAGCATTCAATGGTGCAACAGTGTACTGCCTTCCTCCTGGAT

GCACTCAAGCACAACCGCCCCAACGAAGGTGCTTTGCAGACGCGTTTACTTGAAATGAAT

TTGATTTCAGCGCCGCAGGTGGCTGATGCCATACTTGGCAACGCCATGTTCACGCACTAC

GATCGTGCGCACATCGCCCAGCTGTGCGAGAAGGCGGGTCTACTGCAGCGTGCACTGGAA

CACTACACCGACTTGTATGACATCAAACGCGCAGTTGTGCATACGCATTTGTTGAATGCC

GATTGGTTGGTCAGCTACTTTGGCACGCTGTCCGTCGAGGATTCGCTGGAATGCTTGAAA

GCTATGCTGACGGCGAATATTCGCCAAAATTTGCAGATCTGCGTGCAAATCGCCACAAAA

TATCACGAACAATTGACCACCAAAGCCCTGATCGATCTCTTTGAGAGTTTCAAATCTTAC

GAGGGTCTCTTCTACTTCCTCGGCTCGATCGTTAACTATTCACAGGATCCGGAAGTGCAT

TTCAAGTATATACAGGCCGCTTGCAAAACGAATCAAATCAAAGAAGTGGAGCGCATCTGT

CGCGAATCTAACTGCTACAACGCCGAGCGCGTTAAGAACTTCCTGAAGGAGGCCAAACTC

ACGGATCAATTGCCGCTGATCATTGTTTGCGATCGTTTTGATTTCGTGCACGATCTGGTG

CTCTACTTGTACCGCAACAATCTGCAGAAATATATTGAGATTTATGTGCAAAAGGTGAAT

CCGTCACGTTTGCCCGTCGTCGTAGGTGGCCTCTTGGATGTCGATTGCTCCGAGGATATT

ATCAAAAATTTGATACTTGTCGTAAAGGGCCAGTTCTCGACCGATGAGCTAGTGGCCGAG

GTGGAAAAACGCAATCGTTTGAAATTGCTGTTGCCATGGTTGGAGTCGCGCGTGCATGAC

GGTTGTGTCGAGCCTGCTACTCACAATGCCCTGGCCAAGATCTACATCGATTCGAATAAC

AATCCCGAACGTTTCCTTAAGGAGAACCAATACTACGATAGTCGTGTTGTCGGTCGCTAT

TGTGAGAAACGCGATCCCCATTTGGCCTGTGTTGCCTACGAACGTGGTCAATGCGATCGC

GAGTTGATCGCTGTTTGCAATGAGAATTCACTGTTCAAGAGTGAGGCGCGTTATTTGGTG

CGTCGTCGCGATCCCGCACTCTGGGTGGAGGTATTATCCGAATCAAATCCCTATAAGCGC

CAACTGATCGACCAAGTCGTACAGACAGCGCTCTCCGAAACTCAGGATCCAGATGATATC

TCGGTGACGGTGAAGGCCTTCATGACTGCCGATCTGCCCAATGAATTGATTGAATTGCTC

GAGAAAATCATTCTCGATTCGTCTGTGTTCTCCGATCATCGCAACTTGCAGAACCTGTTG

ATCTTGACCGCAATCAAGGCGGATCGCACACGCGTTATGGACTACATCAATCGCCTGGAT

AACTACGATGCACCCGATATAGCGAACATAGCTATTTCGAATCAACTGTACGAGGAAGCA

TTCGCCATCTTTAAGAAGTTTGACGTGAACACCTCCGCTATACAAGTGCTCATTGAGCAG

GTGAATAATTTGGAGCGCGCTAACGAGTTTGCTGAACGCTGCAATGAGCCAGCCGTTTGG

TCACAACTAGCAAAGGCTCAGTTGCAACAGGGACTCGTCAAGGAGGCCATTGACTCGTAC

ATCAAAGCGGACGATCCGAGCGCGTACATGGATGTGGTCGATGTCGCCTCGAAGGCTGAC

TCATGGGACGACCTGGTGCGTTATCTACAGATGGCACGCAAAAAGGCGCGCGAGTCGTAT

ATCGAAAGCGAACTGATCTATGCCTACGCACGCACCGGCCGTCTTGCCGATCTCGAAGAA

TTTATCTCCGGACCGAATCATGCGGACATACAGAAGATTGGCGATCGCTGCTTCAACGAT

GGCATGTACGATGCAGCTAAATTGTTATATAATAACGTGAGCAACTTTGCGCGTCTAGCC

ATCACATTGGTGTACTTGAAGGAGTTCCAAGGCGCCGTAGACTCGGCACGCAAAGCCAAT

TCGACACGTACTTGGAAGGAGGTGTGCTTCGCTTGCGTCGATGCCGAAGAATTCCGTCTA

GCGCAAATGTGTGGCCTACACATCGTCGTGCACGCCGACGAATTAGAAGATCTAATTAAT

TATTATCAAGATCGTGGTTACTTTGAAGAGCTCATTGCGCTGTTGGAGTCCGCTTTGGGA

TTGGAACGCGCACACATGGGCATGTTCACCGAACTGGCTATACTCTACTCGAAATTCAAG

CCGTCTAAGATGCGTGAGCATCTAGAGCTTTTCTGGTCGCGCGTCAACATACCAAAAGTG

TTGCGCGCTGCCGAATCGGCTCACCTTTGGTCCGAACTGGTATTCCTCTACGACAAGTAT

GAAGAGTACGATAATGCAGTGCTTGCCATGATGGCACATCCGACGGAGGCTTGGCGTGAG

GGTCACTTTAAGGACATCATCACCAAAGTGGCCAATATCGAACTCTACTACAAAGCTATA

CAATTCTATTTGGATTATAAACCGTTATTGTTGAACGATATGTTATTAGTATTGGCACCA

CGTATGGATCATACACGTGCTGTGAGCTTTTTCTCGAAGACCGGTCACTTGCAATTGGTG

AAACCATATTTACGTTCGGTGCAATCGCTCAACAATAAAGCGATCAACGAGGCGCTGAAC

GGGCTGCTCATTGAAGAGGAAGATTATCAGGGTCTGCGTAATTCCATCGATGGATTCGAT

AATTTCGACACAATCGCACTGGCACAAAAGTTGGAAAAGCACGAGTTAACCGAATTCCGA

CGCATTGCTGCCTATTTGTACAAAGGCAACAACCGCTGGAAGCAGAGCGTCGAATTGTGC

AAAAAGGACAAACTCTACAAGGACGCAATGGAGTATGCGGCCGAATCGGGCAAACAGGAA

ATTGCCGAAGAGCTTTTGGGCTGGTTCCTTGAACGCAACGCTCACGACTGCTTTGCTGCC

TGCCTATTCCAATGTTACGATTTGCTGCGGCCTGATGTTATACTGGAGTTGGCATGGAAA

CACAACATTATGGACTTTGCCATGCCGTATTTGATACAAGTAATACGCGAATACACCTCA

AAAGTGGATAAACTGGAGCAAACCGAAGCGCAACGCGAAAAGGAGGACGAAACAGTCGAA

CATAAGAACATCATCACAATGGAACCACAATTGATGATAACCGCGGGTCCGGCCATGGGC

ATACCACCACAATATGCGCAGAACTATGCGGCGGCGCCTGGCTATGCGCCAAACATGGCA

TATCCTGGCTATGGAATGTAGGCTACAACTACAATCACATACACCACCACACAACATTAT

TAAGAGCGAAATTTAAGAACTACAAAAACAACATATGTAACATGAAAATTGCGAACACTA

CCGATTGCGTTGGAAAACACACACACGCACAGACAGACTCAAGACTCAAGAAAAATGCAA

CGTATATATAAAAAAAATATTTAAATTTCGTGCATAAGAGAATATAAAGCAGAAAAAAAA

GAAAAATTATAAAATTTAAATTAAAAAACCACAAACGCGAAATCAAAATGTCCCATGTAA

AAGTAACTGTAGTTACAGCAGCATTAAAATTGGAACGCAAAAACGAAACACTACATTACA

TAAATGCATATGTCAGGCGTGTGTGTTGCGCATACACATATATTTTTGGCATATAATAAA

CTACTATATACAACTATACATAAATGTATTACAAGTATAGTATATATAATAATTATTACA

CATATACATTACCATTATTACAATTACAAATTAAATTTATAATTACAAGTTGTTGTTTGT

TTGATTATTTCGTGCTTTGTTGTCTCTACTTTGCTTGCATATAATTGAAAATTTGTTTCC

CTTGTGTTTCTGTATTTATTCTTGACGTATTTGAACTTTTTTCTTATTATTCCATTTGCC

CCACTCAATTGTTTGGCTTGTATTTACGTTTCGAAAACAAAAAGTAATAATCCATAACAA

AAACCAAATGCAAAGCACGTCTGGTGTTGAACAAATGATGAAGAGAATCAACAAGAACAC

CAACAAAATAACGTAATTGAAAGGAAAAATTTATGCTAATTTTTTTTGTCTTTATTATAA

GTGAAC

**tBLASTn(First hit)**

Score = 3150 bits (8167), Expect = 0.0, Method: Compositional matrix adjust.

Identities = 1583/1677 (94%), Positives = 1630/1677 (97%), Gaps = 6/1677 (0%)

Frame = +1

Query 1 MTQPLPIRFQEHLQLTNVGINANSFSFSTLTMESDKFICVREKVNDTAQVVIIDMNDATN 60

MTQ LPIRFQEHLQLTNVGIN NS SFSTLTMESDKFICVREKVNDTAQVVIIDMND +N

Sbjct 751 MTQVLPIRFQEHLQLTNVGINLNSISFSTLTMESDKFICVREKVNDTAQVVIIDMNDVSN 930

Query 61 PTRRPISADSAIMNPASKVIALKAQKTLQIFNIEMKSKMKAHTMNEDVVFWKWISLNTLA 120

PTRRPISADSAIMNPASKVIALKAQKTLQIFNIEMKSKMKAHTM EDVVFWKWISLNTLA

Sbjct 931 PTRRPISADSAIMNPASKVIALKAQKTLQIFNIEMKSKMKAHTMTEDVVFWKWISLNTLA 1110

Query 121 LVTETSVFHWSMEGDSMPQKMFDRHSSLNGCQIINYRCNASQQWLLLVGISALPSRVAGA 180

LVTETSV+HWSMEGDS PQKMFDRHSSLNGCQIINYRCN SQQWLLLVGISALP+RVAGA

Sbjct 1111 LVTETSVYHWSMEGDSTPQKMFDRHSSLNGCQIINYRCNPSQQWLLLVGISALPNRVAGA 1290

Query 181 MQLYSVERKVSQAIEGHAASFATFKIDANKEPTTLFCFAVRTATGGKLHIIEVGAPPNGN 240

MQLYSVERKVSQAIEGHAASFA+FK++ NKEP+TLFCFAVRTA+GGKLHIIEVGAPP GN

Sbjct 1291 MQLYSVERKVSQAIEGHAASFASFKMEGNKEPSTLFCFAVRTASGGKLHIIEVGAPPAGN 1470

Query 241 QPFAKKAVDVFFPPEAQNDFPVAMQVSAKYDTIYLITKYGYIHLYDMETATCIYMNRISA 300

QPF KK+VDVFFPPEAQ+DFPVAMQVSAKYDTIYLITKYGYIHLYDMETATCIYMNRISA

Sbjct 1471 QPFPKKSVDVFFPPEAQSDFPVAMQVSAKYDTIYLITKYGYIHLYDMETATCIYMNRISA 1650

Query 301 DTIFVTAPHEASGGIIGVNRKGQVLSVTVDEEQIIPYINTVLQNPDLALRMAVRNNLAGA 360

DTIFVTAPHEASGGIIGVNRKGQVLSVTVDEEQIIPYINTVLQNPDLALRMAVRNNL+GA

Sbjct 1651 DTIFVTAPHEASGGIIGVNRKGQVLSVTVDEEQIIPYINTVLQNPDLALRMAVRNNLSGA 1830

Query 361 EDLFVRKFNKLFTAGQYaeaakvaalapkaILRTPQTIQRFQQVQTPAGSTTPPLLQYFG 420

EDLFVRKFNKLFTAGQYAEAAKVAALAPK ILRTPQTIQRFQQVQ+PAGSTTPPLLQYFG

Sbjct 1831 EDLFVRKFNKLFTAGQYAEAAKVAALAPKGILRTPQTIQRFQQVQSPAGSTTPPLLQYFG 2010

Query 421 ILLDQGKLNKFESLELCRPVLLQGkkqlcekwlkeeklecseelGDLVKASDLTLALSIY 480

ILLDQGKLNK+ESLELCRPVL+QGKKQLCEKWLKEEKLECSEELGDLVK SDLTLALSIY

Sbjct 2011 ILLDQGKLNKYESLELCRPVLVQGKKQLCEKWLKEEKLECSEELGDLVKTSDLTLALSIY 2190

Query 481 LRANVPNKVIQCFAETGQFQKIVLYAKKVNYTPDYVFLLRSVMRSNPEQGAGFASMLVAE 540

LRANVPNKVIQCFAETGQFQKIVLYAKKVNYTPDY+FLLRSVMR+NPEQGAGFASMLVAE

Sbjct 2191 LRANVPNKVIQCFAETGQFQKIVLYAKKVNYTPDYIFLLRSVMRTNPEQGAGFASMLVAE 2370

Query 541 EE-PLADINQIVDIFMEHSMVQQCTAFLLDALKHNRPAEGALQTRLLEMNLMSAPQVADA 599

EE PLADINQIVDIFMEHSMVQQCTAFLLDALKHNRP EGALQTRLLEMNL+SAPQVADA

Sbjct 2371 EEEPLADINQIVDIFMEHSMVQQCTAFLLDALKHNRPNEGALQTRLLEMNLISAPQVADA 2550

Query 600 ILGNAMFTHYDRAHIAQLCEKAGLLQRALEHYTDLYDIKRAVVHTHMLNAEWLVSFFGTL 659

ILGNAMFTHYDRAHIAQLCEKAGLLQRALEHYTDLYDIKRAVVHTH+LNA+WLVS+FGTL

Sbjct 2551 ILGNAMFTHYDRAHIAQLCEKAGLLQRALEHYTDLYDIKRAVVHTHLLNADWLVSYFGTL 2730

Query 660 SVEDSLECLKAMLTANLRQNLQICVQIATKYHEQLTNKALIDLFEGFKSYDGLFYFLSSI 719

SVEDSLECLKAMLTAN+RQNLQICVQIATKYHEQLT KALIDLFE FKSY+GLFYFL SI

Sbjct 2731 SVEDSLECLKAMLTANIRQNLQICVQIATKYHEQLTTKALIDLFESFKSYEGLFYFLGSI 2910

Query 720 VNFSQDPEVHFKYIQAACKTNQIKEVERICRESNCYNPERVKNFLKEAKLTDQLPLIIVC 779

VN+SQDPEVHFKYIQAACKTNQIKEVERICRESNCYN ERVKNFLKEAKLTDQLPLIIVC

Sbjct 2911 VNYSQDPEVHFKYIQAACKTNQIKEVERICRESNCYNAERVKNFLKEAKLTDQLPLIIVC 3090

Query 780 DRFDFVHDLVLYLYRNNLQKYIEIYVQKVNPSRLPvvvgglldvdCSEDIIKNLILVVKG 839

DRFDFVHDLVLYLYRNNLQKYIEIYVQKVNPSRLPVVVGGLLDVDCSEDIIKNLILVVKG

Sbjct 3091 DRFDFVHDLVLYLYRNNLQKYIEIYVQKVNPSRLPVVVGGLLDVDCSEDIIKNLILVVKG 3270

Query 840 QFSTDELVEEVEKRNRLKLLLPWLESRVHEGCVEPATHNALAKIYIDSNNNPERYLKENQ 899

QFSTDELV EVEKRNRLKLLLPWLESRVH+GCVEPATHNALAKIYIDSNNNPER+LKENQ

Sbjct 3271 QFSTDELVAEVEKRNRLKLLLPWLESRVHDGCVEPATHNALAKIYIDSNNNPERFLKENQ 3450

Query 900 YYDSRVVGRYCEKRDPHLACVAYERGLCDRELIAVCNENSLFKSEARYLVGRRDAELWAE 959

YYDSRVVGRYCEKRDPHLACVAYERG CDRELIAVCNENSLFKSEARYLV RRD LW E

Sbjct 3451 YYDSRVVGRYCEKRDPHLACVAYERGQCDRELIAVCNENSLFKSEARYLVRRRDPALWVE 3630

Query 960 VLSESNPYKRQLIDQVVQTALSETQDPDDISVTVKAFMTADLPNeliellekiilDSSVF 1019

VLSESNPYKRQLIDQVVQTALSETQDPDDISVTVKAFMTADLPNELIELLEKIILDSSVF

Sbjct 3631 VLSESNPYKRQLIDQVVQTALSETQDPDDISVTVKAFMTADLPNELIELLEKIILDSSVF 3810

Query 1020 SDHRNLQNLLILTAIKADRTRVMDYINRLENYDAPDIANIAISNQLYEEAFAIFKKFDVN 1079

SDHRNLQNLLILTAIKADRTRVMDYINRL+NYDAPDIANIAISNQLYEEAFAIFKKFDVN

Sbjct 3811 SDHRNLQNLLILTAIKADRTRVMDYINRLDNYDAPDIANIAISNQLYEEAFAIFKKFDVN 3990

Query 1080 TSAIQVLIDQVNNLERANEFAERCNEPAVWSQLAKAQLQQGLVKEAIDSYIKADDPSAYV 1139

TSAIQVLI+QVNNLERANEFAERCNEPAVWSQLAKAQLQQGLVKEAIDSYIKADDPSAY+

Sbjct 3991 TSAIQVLIEQVNNLERANEFAERCNEPAVWSQLAKAQLQQGLVKEAIDSYIKADDPSAYM 4170

Query 1140 DVVDVASKVESWDDLVRYLQMARKKARESYIESELIYAYARTGRLADLEEFISGPNHADI 1199

DVVDVASK +SWDDLVRYLQMARKKARESYIESELIYAYARTGRLADLEEFISGPNHADI

Sbjct 4171 DVVDVASKADSWDDLVRYLQMARKKARESYIESELIYAYARTGRLADLEEFISGPNHADI 4350

Query 1200 QKIGNRCFSDGMYDAAKLLYNNVSNFARLAITLVYLKEFQGAVDSARKANSTRTWKEVCF 1259

QKIG+RCF+DGMYDAAKLLYNNVSNFARLAITLVYLKEFQGAVDSARKANSTRTWKEVCF

Sbjct 4351 QKIGDRCFNDGMYDAAKLLYNNVSNFARLAITLVYLKEFQGAVDSARKANSTRTWKEVCF 4530

Query 1260 ACVDAEEFRLAQMCGLHIVVHADELEDLINYYQNRGYFDELIALLESALGLERAHMGMFT 1319

ACVDAEEFRLAQMCGLHIVVHADELEDLINYYQ+RGYF+ELIALLESALGLERAHMGMFT

Sbjct 4531 ACVDAEEFRLAQMCGLHIVVHADELEDLINYYQDRGYFEELIALLESALGLERAHMGMFT 4710

Query 1320 ELAILYSKFKPSKMREHLELFWSRVNIPKVLRAAESAHLWSELVFLYDKYEEYDNAVLAM 1379

ELAILYSKFKPSKMREHLELFWSRVNIPKVLRAAESAHLWSELVFLYDKYEEYDNAVLAM

Sbjct 4711 ELAILYSKFKPSKMREHLELFWSRVNIPKVLRAAESAHLWSELVFLYDKYEEYDNAVLAM 4890

Query 1380 MAHPTEAWREGHFKDIITKVANIELYYKAIEFYLDFKPLLLNDMLLVLAPRMDHTRAVSY 1439

MAHPTEAWREGHFKDIITKVANIELYYKAI+FYLD+KPLLLNDMLLVLAPRMDHTRAVS+

Sbjct 4891 MAHPTEAWREGHFKDIITKVANIELYYKAIQFYLDYKPLLLNDMLLVLAPRMDHTRAVSF 5070

Query 1440 FSKTGYLPLVKPYLRSVQSLNNKAINEALNGLLIDEEDYQGLRNSIDGFDNFDNIALAQK 1499

FSKTG+L LVKPYLRSVQSLNNKAINEALNGLLI+EEDYQGLRNSIDGFDNFD IALAQK

Sbjct 5071 FSKTGHLQLVKPYLRSVQSLNNKAINEALNGLLIEEEDYQGLRNSIDGFDNFDTIALAQK 5250

Query 1500 LEKHELTEFRRIAAYLYKGNNRWKQSVELCKKDKLYKDAMEYAAESCKQDIAEELLGWFL 1559

LEKHELTEFRRIAAYLYKGNNRWKQSVELCKKDKLYKDAMEYAAES KQ+IAEELLGWFL

Sbjct 5251 LEKHELTEFRRIAAYLYKGNNRWKQSVELCKKDKLYKDAMEYAAESGKQEIAEELLGWFL 5430

Query 1560 ERDAYDCFAACLYQCYDLLRPDVILELAWKHKIVDFAMPYLIQVLREYTTKVDKLELNEA 1619

ER+A+DCFAACL+QCYDLLRPDVILELAWKH I+DFAMPYLIQV+REYT+KVDKLE EA

Sbjct 5431 ERNAHDCFAACLFQCYDLLRPDVILELAWKHNIMDFAMPYLIQVIREYTSKVDKLEQTEA 5610

Query 1620 QREKEDDSTEHKNIIQMEPQLMITAGPAMGIPPQYAQNYPPgaatvtaaggRNMGYP 1676

QREKED++ EHKNII MEPQLMITAGPAMGIPPQYAQNY NM YP

Sbjct 5611 QREKEDETVEHKNIITMEPQLMITAGPAMGIPPQYAQNYAAAPGYAP-----NMAYP 5766

**Conserved domains**

**protein containing domains Clathrin-link, Clathrin_H_link, Clathrin, and CLH**

**
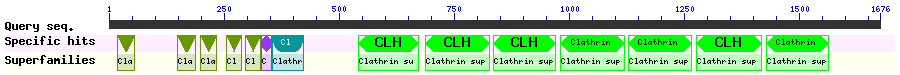
**

**Adaptor protein 50 (AP50)**

**>TRINITY_DN29475_c0_g1_i1 len=1961**

ACAGAGTTAAGATTGGCAGAGATAACGTATTTTTTAATATTTTCTAGACACTTATTAGTC

CGCAAAATATTCCTTGCTTTTAGCTACATTAAATTAGCGGTTATATTTGTGCAAATTTGC

TAACTGCTCCAAAAGATAGAAGAGAAATCTAAGAATCGTAAAGTGAGAGCATTGTTCGAT

ATGAAGAAACGCTTCTACAATTAACGTTGACGATGTAAATAAAAATACAAAATAATATCA

TAGGTATAAAATTGAGGAATAGACATAAGTGCATCTAATAACGTTAGAAAGTTGGGATTA

TATAATAGGAAATGTGATTAAATTATATGATTAATACGCATACATGTATGTATAAATGTA

GATAATAAAGGATATACACACATTTTACAACTAGTGTGGAATATAAGATATTCAATTTGA

TTGAGAATACAAATCGGGAAAATTCGGTATGCGTCTACACGCCTAAACTTCACCTGTTCA

ACAGCGAGTTTCATATAGGCCACTGCGTCCTATGTAGCGCACCCATTTCACAACATCGTG

GTCAGAGTAATTGAGCTTTGGTTCAAATACTTTCAAATAGCGTACTTTAAAGCCGGATGG

CGCAAATGGTACTTCGAAATTCATTGAAATAGGTGGACGTGTCCATTTTTTCTTCGTGTC

CGTCTCCAATAACTCGATTTCTGCCGAAAGTTGTGTTTCTTTCATGCCGGCCATCCGTTT

AATTTTCCAGACAATTGCATTGTCCGAAGCTTTATATTTTGCCTTGCCTTTCAGGCAAAT

CAATTGGACACCCGAAGTATTCAATGGTGTTGGAATTTTTACTTCAATTTTCTGACCCAA

TAGTGAAGGTTTAAAATTGGACTTGAGTACGATTTTCACCTCCATTTTAGTTCGCCCTAC

CTCTCGTACAAGCGGTATAACTCGGAATGGCAGTGAAATGTCTTTGGTAGTACGATAGCG

CATGAGCTCGAATTCACCATCGGGTGGAATAAAACTAATAGAGTGTTCTGTCTCGAATTT

GCTCAACTTGACACATTGATGAAACTGACAATCGTCGATTACTACAACAGGCTTGCCTGA

ACGAGAAGTCTCCGCCTCGGAATTGCCCCCAATACCACGACCTTTCGACTCCATCACTAT

TTTGTCATTAATACCAAATTTACACTCGGGCATGCCCGAAAGATACGACTTCATGACCAC

TTTTCCAGCAACATGGGCGGAAAGTACTTGCCCTTGTGGGCTCATCAGCAGATTCACGTA

CTCCAACACGTCGAGGAAAAGTTCATTGCGACGGTATTTTATACCTTCGCGCCGCCAGCC

GATCTGACCGGTCACCTGAGAGGTGATTTGCATCTGCTCTTCCTTGGTGGCGGATTTAAT

GCCCTGTTGGGTGATGAACGTCTTCAGCGTGCCCGAGTCCGTGTTCTGCGGATAGCCGAA

ATCGAGAATCTCGTCCAGCAATTCATATATAAGAACGAAGTTATTCTTGATGTTCTCTTC

AGAAATTTTACCAAAGTATGATTGCATTACTTCGATAATTTTTAATAAGAATTCGAATAC

CATCGCCGCATTCACATTTTGCTTCGTAACCGCAGCCAGCCAAATATTGGCACGCTTTAT

GTGGAAAAAGCTTGTTCGAGCAATATTCGTTACCGGGGAACGTACTTGCTGCCGTGCATG

GATGACGTTGACACGAAAAGCATCCACTGCATTACGGCCAATATCATCGCGATATACCCT

ACTGATTAATACCTCCCCCTTGTGATTATACACGAATAATCCACCAATCATTTCAGCAAA

AACACTTTTTTACTTCCTTAATGAGGATAGATAACACTGAAACGCCCTATTTAAATTGAT

AATTTTATTAGTTTTTCACTTTACTACGATTATCGATCAACACCTTCTTTTTTCGGCCCT

CCTGACAGCTGTCAGCGAATTCGCCTAATTGTACACAGTTG

**tBLASTn(First hit)**

Score = 899 bits (2323), Expect = 0.0, Method: Compositional matrix adjust.

Identities = 433/437 (99%), Positives = 436/437 (99%), Gaps = 0/437 (0%)

Frame = -3

Query 1 MIGGLFVYNHKGEVLISRVYRDDIGRNAVDAFRVNVIHARQQVRSPVTNIARTSFFHIKR 60

MIGGLFVYNHKGEVLISRVYRDDIGRNAVDAFRVNVIHARQQVRSPVTNIARTSFFHIKR

Sbjct 1791 MIGGLFVYNHKGEVLISRVYRDDIGRNAVDAFRVNVIHARQQVRSPVTNIARTSFFHIKR 1612

Query 61 ANIWLAAVTKQNVNAAMVFEFLLKIIEVMQSYFGKISEENIKNNFVLIYELLDEILDFGY 120

ANIWLAAVTKQNVNAAMVFEFLLKIIEVMQSYFGKISEENIKNNFVLIYELLDEILDFGY

Sbjct 1611 ANIWLAAVTKQNVNAAMVFEFLLKIIEVMQSYFGKISEENIKNNFVLIYELLDEILDFGY 1432

Query 121 PQNTDSGTLKTFITQQGIKSATKEEQMQITSQVTGQIGWRREGIKYRRNELFLDVLEYVN 180

PQNTDSGTLKTFITQQGIKSATKEEQMQITSQVTGQIGWRREGIKYRRNELFLDVLEYVN

Sbjct 1431 PQNTDSGTLKTFITQQGIKSATKEEQMQITSQVTGQIGWRREGIKYRRNELFLDVLEYVN 1252

Query 181 LLMSPQGQVLSAHVAGKVVMKSYLSGMPECKFGINDKIVMESKGRGLSGNSEAETSRSGK 240

LLMSPQGQVLSAHVAGKVVMKSYLSGMPECKFGINDKIVMESKGRG+ GNSEAETSRSGK

Sbjct 1251 LLMSPQGQVLSAHVAGKVVMKSYLSGMPECKFGINDKIVMESKGRGIGGNSEAETSRSGK 1072

Query 241 PVVVIDDCQFHQCVKLSKFETEHSISFIPPDGEFELMRYRTTKDISLPFRVIPLVREVGR 300

PVVVIDDCQFHQCVKLSKFETEHSISFIPPDGEFELMRYRTTKDISLPFRVIPLVREVGR

Sbjct 1071 PVVVIDDCQFHQCVKLSKFETEHSISFIPPDGEFELMRYRTTKDISLPFRVIPLVREVGR 892

Query 301 TKMEVKVVLKSNFKPSLLGQKIEVKIPTPLNTSGVQLICLKGKAKYKASENAIVWKIKRM 360

TKMEVK+VLKSNFKPSLLGQKIEVKIPTPLNTSGVQLICLKGKAKYKAS+NAIVWKIKRM

Sbjct 891 TKMEVKIVLKSNFKPSLLGQKIEVKIPTPLNTSGVQLICLKGKAKYKASDNAIVWKIKRM 712

Query 361 AGMKETQLSAEIELLETDTKKKWTRPPISMNFEVPFAPSGFKVRYLKVFEPKLNYSDHDV 420

AGMKETQLSAEIELLETDTKKKWTRPPISMNFEVPFAPSGFKVRYLKVFEPKLNYSDHDV

Sbjct 711 AGMKETQLSAEIELLETDTKKKWTRPPISMNFEVPFAPSGFKVRYLKVFEPKLNYSDHDV 532

Query 421 VKWVRYIGRSGLYETRC 437

VKWVRYIGRSGLYETRC

Sbjct 531 VKWVRYIGRSGLYETRC 481

**Conserved domains**

**AP-2_Mu2_Cterm domain-containing protein**


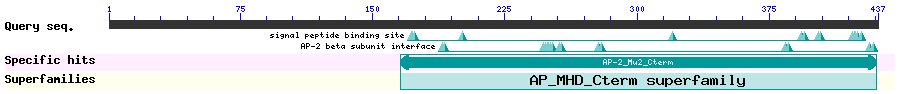


**TRF3**

**>TRINITY_DN30474_c2_g1_i5 len=1157**

AAAATAAAGAAAAATTAATCAATTGGTGATTCCCGAGCATTTCGACAGCCCACTGAGCTG

CTGCAAAGCCGGCTCCGATCTTGACGACGACAACGCCAACGAAGACGGCGACGGCGACAA

CGAAATAGAGCCGCCGCATCGGCTATAAAAAGAAACTGGCTGGCGATGCACTCAACAGTT

AAGTATTGGACGCGTTTGAATGAGCAGCGCTAAAATGGGTTATACGACAATAGTTTACTT

GACTACGGCCCTATTGGGCGTGGTCAGTTTGGCTTCGGTCCAAGCTGACGAGCAAATATA

TCGCATGTGCGTGCCGCAACTGTATTACAAAGATTGTTTGGATCTGTTGAAGGATCCCTC

GGAGGCTGGCATACAAATGGAATGCGTAGCTGGACGTGATCGCATCGATTGTTTGGACAA

AATCAATCAGCGCAAGGCTGATGTGCTCGCCAGCGAACCGGAGGATATGTACGTCGCCTA

TCACACGAAGAATCAGGATTATCGCGTCGTCTCCGAAATTCGCTCCAAGGCTGATAAGGA

CGCGGAGTTCCGTTATGAGGGCATTATTTTGGTGAAGAAAAATTCGAATATTAACAGCTT

GAAGGAGTTGCGCGGCAAGAAATCCTGTCACACCGGCTTCGGACGCAATGTGGGCTATAA

GATTCCAATTACTAAATTGAAGAATACGCATATTCTTAAAATCTCTCTGGATCCCGAAAT

AACCGCCACCGAACGTGAATTGAAAGCCCTGTCGGAGTTCTTCACACAATCCTGTCTGGT

TGGTACCTATTCGCCATATCCCGAAACTGATCGTCTGCTGAGTAAGTCACTTGAAGTTGA

GCCTTAAAAGCTGGCACGCTCAGCTGCAATTGAATTTGAATTTCTAATGCGATATTCTTC

GTTTCCAACAGAGAAGAAATACTCCAATTTGTGCGCACTGTGCGAGAAACCCGAACAATG

CAACTATCCTGACATATTTTCTGGTTATGATGGCGCAATTCGTTGCTTGGACAAGGGCAA

GGGTGAGGTGGCCTTCACCAAGGTGCAATACATCAAGTCATACTTTGGCGTAAGTAGTGA

ATGACGAGTGATAAAAGCAAAAGATATGTCCACCTTACAATTCAACTTAACATTTGCAGC

TCACGCCCGGCTCCAAA

**tBLASTn(First hit)**

Score = 294 bits (753), Expect = 5e-098, Method: Compositional matrix adjust.

Identities = 134/174 (77%), Positives = 156/174 (90%), Gaps = 0/174 (0%)

Frame = +2

Query 28 HAEEPIYRLCVPQIYLAECQQLLADPSEAGIRMECVAGRDRVDCLELIEQRKADVLATEP 87

A+E IYR+CVPQ+Y +C LL DPSEAGI+MECVAGRDR+DCL+ I QRKADVLA+EP

Sbjct 281 QADEQIYRMCVPQLYYKDCLDLLKDPSEAGIQMECVAGRDRIDCLDKINQRKADVLASEP 460

Query 88 EDMYIAYHRKNEDYRVISEIRTQQDKDASFRYEGIILVKKDSPIRTLQQLRGAKSCHTGF 147

EDMY+AYH KN+DYRV+SEIR++ DKDA FRYEGIILVKK+S I +L++LRG KSCHTGF

Sbjct 461 EDMYVAYHTKNQDYRVVSEIRSKADKDAEFRYEGIILVKKNSNINSLKELRGKKSCHTGF 640

Query 148 GRNVGYKIPITKLKNTHILKVSADPQISATERELKSLSEFFAQSCLVGTYSTHP 201

GRNVGYKIPITKLKNTHILK+S DP+I+ATERELK+LSEFF QSCLVGTYS +P

Sbjct 641 GRNVGYKIPITKLKNTHILKISLDPEITATERELKALSEFFTQSCLVGTYSPYP 802

**Conserved domains on [lcl|ORF2]**

**
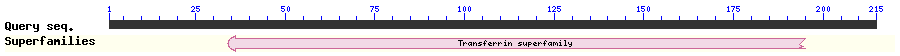
**

**Sortilin Like Receptor (SLR)**

**>TRINITY_DN26733_c0_g2_i34 len=12561**

GCACATTTCAAAGCAAAGATTTATTTCGTTTATTTTTAGTCGCTGTTCATTTATTTTTTT

TACTTTTATTATTAATATTAATTATTTATTTACTTATTAACGCTTTATACAATTATTTGC

CATACATAACGATAGTCGCATCGATTTTCTTTTTCCTTTTAGTTTCGTTTTTGTTTTTTG

CTTTTTTCATTTACATACAAATATAAGTAGAAAAATTAATTATTACTTTGAAGTGTTTTA

ATGCATTAGTATTAATTATTATAATATTAGACTGGCGTACATTTTTTGTTGTGGTCAAAA

ACTATAACTTTTTACTAAAAGTTCGCATTCAAACTATAACGTAGTTAAAAAATAATAATT

TGAGCATTAAATTCCAAAACTAAAACTTTCATTCAAATTAAATATGTTTCTTTACCACTT

CAATCCTTTCCTTGCCACGGGGCAACTCAGCTGCGAAAATGAACAGAAAAATATTGTTTA

TGTACGCGTGACTAACATCAATTTACAGATAATATTTTCGCACTAGTTTTATGGGTTATT

TTTTAAGGTTTTTACTTAACTCTAAGTTAATTAAAACTAAAAGAAATTTCCGCTTGTACG

TCTCAAAATTTTGAACGAACAAAATTAACGCAGTGGAAAAAACGAGTGGCGGCGCTGGGC

AGTTTTTGAAAGTTGTCGACTTTTTGGTATTTGGAATCAGTTTTCTTCGAAAAAATTAAA

CTTACAAATTAAGAAATTATTGCAAAATATATGACACAAGTTTCATTAAAATTAGCTTTG

TTGTATTGTTGTTGTGCGCTTAAGTTAAAATCTGTTACAAAATGAGAGAAGAAAAAAAAA

AAAATAGAATACCGAATGAGTTGGACTCGACGTGCTTTTCGTTTAATCTGCATTTTTTGT

TGTTAGCTTTCGAATCCATTAAATTATCTGTGAAGAATTTATATTTATAAAAAAATGTAT

ATATAATATTTAAAAGCTACGTTCAAACGACTGGTGGTTCGGTGCCAAGAGACTCGTCGT

CGGCTTGCGGACATATGTCGACATTCTCGCCACTAATGGCCGCTGTGCCCAGTTGTATTA

AACCGTGCGCATCACCTTCTACGACGCGCACGCAGCCGTTGAAGTTCTTGGTGTAGCGGC

CGCCAGTGAATTTGGCAACGTCCGGGGTGCCACCAATGAACACATGTCCCGGTAGACTCA

TCTTGTCCTTGCCGGTCGGACGTGTTTCGCCGGCATCCAATAGGTGGTCCAGCTCCAGTA

TGGCAGTATTGTCCGTGCGTTTGATCAACACAATGTGGCGACGTCCATCGTCGATGCGTT

TGTCCGGATTGCGTATGACGGTTTCTTCGCCGTTGAGACGGAACGAGAATTCCACAGTGC

CATCGACTATGGCTAACGCCATGAAGTCTTGGCCGGTGTATTCCTCGTCCCTTTTCTGAC

CCCACCAAAGCAGCAGACCATTTGGATCGGTTGTGGAGAAGACCATTGCTGCAAATGAGT

ATTTTTGGTCGACGTCTGAGTTGAATTGATTGCGATCTACTTCAAGGTAGCCGTTGCCAT

GGAAGTTGGCATCGTATTCGATAGTTATGTGTTCTTCACAGTGCCTTCCGGTGAAGCCAA

TTGTGCACGCGCATACGGCCTTGCCATTGTGTACGCTGCAATTGCCGCCATTTTCACAAG

GATCACTCGCGCATGCGTCTACTTGTTGGTCCTCCTTTTCACTCGGCGATTCAATTGGTT

TGCCTGACGCTTGTTGAATATTTGTCGCTTCGTGCTCGTAGTTGTCGAACGATTCATTCT

GGTCGATCTCATTGATTTCACCACAGTTCTGGATGTTCGCCGCGTCATGGATGTCGGCAA

TAAGATTGATCTGTCGTTGGCCTTCGAACAAGCTCGAGATGCAACCATCGAAGCCTTGTG

TGATATTCACGTCACGGTTGAGTTTAATATTTTCGTGATCGTAGCCTCCGAGGTATAGCG

GTGTCTTTATGTACAGAATGCGTGCCAATTTAGTCGCCTTCGCGCGCTGCTCTGGCTCAT

TGCCAACGCGCAAAATGCCTTCGCCGAAACGGCGTGACACTTCAATTTCGGTCCATTTGT

TTATCGGCAGTGGATTCTGCGAACGCACCAATATAGGGTTTAGACGTGCGCCGGTATTTA

TAATCAGTTCCACGTGTTTGTCATGTAGCAAGACAGCAATGAAGTCGCCGCTGGGTAGTT

TTGATTCCGCGGCGTAAAGGAGTACGGCGTCTTGCAACGAGTTTGGTCGTACTTTGAATT

TGACATTCAGTTTGGAAGCGCGTGGTGTGGGATAAGCTGCGTAGCTGTTGAGTTTGAATG

CCAAGCTGTTTTCATTCAGATGCTCAATATATTGACAACGATCGCCGGTCTTGTTCAATG

GACAAAGGCATTCCATGCCGAACTCAGTGTTCTCACAACGTCCACTACCACAAATGCCGG

TGGTGCACTGTGTGCCCTCGACGCCGCAGTTATTTCCGGTCCAACCCTGTTGGCAGATAC

AAGTGTACGCCGTTTCAGTTTGACTCTCCAAACAGATGCCGCCATTTTTGCATGGGTTTT

GTTGACACGGACGGCAAGCAGTGACGCCCTCTTTCAGTTTGGCCTCCTTCATTAACTCAA

CAATTGTACCCTGAAGCGTGAGTCGGCTAATACAACCAACAAAGCCGACTTGTTGGTCGA

TGGCGTCAAGCGGCAGCATATCCCAGTTAGGCACGCCACCAATGAACAAATCTTCGATTA

GATCCAGCGGAGAGATCTGCGCTTGTGTGGGGAATGCAACTGGATGCTGATCATCGACCT

GCATATAGCCATCGCGACGGAAGCGATTAACGCGTATCGTGTGCCATTCTTTGAGTGAGA

CTGGACGTTCGGCGCGTATAACCAACGGTTTGCCATCGAAGTCGAAGCGGAATTCGGGAT

AGCGGTCTTTCAGCGACAGTGAGATATAGTCACCGCTGCCGCGCTTTTGGCCGTTGAACA

GCAACAGACCGTTGCCTTGTTCGGGTCGGAATGTGATATCAAAGTTAAACTTAATGTACG

ACTCGGGTAGTGTGGGGAAAGACATATAACTGATGGGCTCCTGATGGAACTGTGGAATGG

CACCAGTAACTACAAGTATGGTGGGATAGTCGATCGACAGTCCATCAGGTGTATTCGACG

TGCAAATATAAGTGCCGGCGTCATTCGCTTGGACATCTTGCAATTCAAGGCGCTCATTGA

ATAGAACGCGATCGGCTTGCATTTGTCCGTACTGACGAGACCAACGATAGCTGCCCGGTA

TTTCGGCGTCGCAGTTCAATTGTGCAGTGGAACCAACACTGGCGTGCACCAACTTCGGGC

GCTTCCATTCGTGCGGCCACTCTTCGATACCTAATTCGTAGCTAGTGGTATACAGGTCGC

CCTCGTCGGTCTTGCACTTGCAGACATAGTTACCCGCGTCGGAAGCGGAGACATGTGAAA

TGATGAGGCGATTGCCGATTTGCTGTATGTGCGGTGGTAGTGGTGAGCCATCGGCACGTT

CCCAAATGACGTCTGTGTAGCTGCTGTCCGAAGAGTAACATTCAACCTCTGTCGATTCAC

CGATGCGGAGCTGCGATGACTGTTGGTCCAGCTTTAGGCTCAAATCGCGTGGGCGTGTCT

GCTGCTGAGGAGGTGGGGTGGGACGCTCCGGTAGCGGCTGTGGTATTATTGGCTTACGGA

ACTGTGGCGCTGCCGCTGGCTTGATATTCAGATCTACAAATGATGGCTGTGAACGGCCGC

CGCCAGTCGAATATGACTCGCAGACGTAACGGCCTTCATCCTGTTTCAGCACACCGCGCA

GGTAGAGTCGTTGATCGCGGATCAGAGCACTGCGTGGCAGCGGGCGACCATCTTGGCGAC

GCCATTCGTATCTTATTTCGCCCCGTATAGGTTCGCGCGCGTTCTGCACATCACACTGCA

GCGTCACATCTTCACCCTCGGGAATATCATAGACTTGAGCGCGCGGTGCTGGCTGATAGG

CTCCATTACGGTTCACGACCACTTCGGCCATGTTGGTGGTGTTGCCGTAGCGGTTCGAGG

CCGAGCAGTAGTAGCGGCCCGCGTCAGCTGGGGTAATGGAGACGAAGCGCAAGTATTGCC

CATCTATACGCACGGAGTCAGTGAGTGGTCGGTTGTTGTCCGTGTGCCAGGCGACGTCGA

TGGGCTGTTCACCAGTGACTTCACAGTAGATGTCGATGTTGTCATTCGGCGCCACCACGA

TGGGCATCTTGGGGTGGAATGTGATGTGTGGAATGGGCACTAAAACCAGCTCGGCGATGA

CGAATGTCACAACAGCGCCGCGGTTGTCCAATGCATTGCAGCGGTACTTGCCGCTGTTCT

GCTCCTCGACACGCTTGATGATCAGACTGTTGCGTTCGAAGTATGAGTTATCTGGTAGGG

GACGACCATCCACACGCTCCCAACGTGTTGACAGGTTCGGTACGTTATTCAAGTCACAGG

TGAGTGTGACGGCGTCGCCCTCATTGGCGCGATAAATATTTTGTGGTGGACCGTAGCCTG

GCTGACGCGTTGGTGGGTATTGTGGGGGTGGTTGTGATTGGTACGGATATGGATATTGTG

GATTCCGAATTACATCGCTGTCGTTGGACGCATCGCCGCGGCGTGGACGTACATCCACAG

AGATGTAGCTCTCCGCAGTGCCTGCTTCGTTGCTTGCGATGCACTTGTACGATTTGGCAT

CATTGCGGTCGATGCGATATTTCTCCAGGAAGGCCTCGTTGTATTGTGGACTGGTCGGCA

GGGAGGCACGCGTGAATTCGGTACTCTCGTCCACCCATTGCACAGTTGGGCTGGGTACGC

CAGTGGCGGTGCAGACCACCTTGAGCTCATCACCTTCAGTTAAGCTCAGAACTTCATCGC

TTGGCTCTAGAGTTATCATTGGTGGCACCAAAACGCGTACTGTTTGTATGCCAGTCGCGC

GTCCAACGTCGTTCTTTGCGACACACTCATAATCACCAGAGTCTTCTACTTGAATATTTC

CGATGATTATGTAACCGGCGCTGATTTCCTTGCTACGTGGTGATAACGGTTTGCCGTCGA

CGCGATGCCACTGTACGCTGGGATCGGGTATGCCAGTGGCACTGCAGTAAAGCATAGCTT

CGGTGCCCACATTTACCGTTTGCGGCTCAGGTGGATGTATATCGACGGAAGGGCTTTCAC

GTGGTTCAACATCAATAATAGTGTTCGATTCATGAGAGCCAGCAACATTCTCAGCGATAC

ACAGGTACATGCCGCGGTTCTCAGGACGCGCGTTTATGATGCGCAAAACGTTACCGGTTT

GTTGCACATTGTCAGCGAGCGCTTCATGAACCTTAGTCCATTTGATGTTGGGGTAGGGCG

AGCCGGTGGCCTCACAAGTAATGGAGAAATCTTGGCCCTGCACGATATGGTTCTCATCTG

GCAAGCGCTTTACAACGGGTGCAATAGTGCCCGGCAGTGGTCCTGGCTGAGGTCCTGGTA

CGGAATATGAACTATCTTCGATCACTACCTGCGCGTCGTTGGTGTAGTTGCGTCGTCCTC

GTTGATCAATGCCGATGCAAGTGTAGAGACCGGAGTCGCGAGGCGTGGACTCGCGGATCT

CAATCGTATTGCCACTGATGATAACGTTGCGCTGGCGGTACACTGGATAACCGTCTTTAT

TCCACTCGTAAACGAGCAAGATAGAGGTGGTTGGTTGGCAGGTGAGACGCACGATATCAC

CCGTTTCGCCAGTGTATGATGGCGGATCGATGTAGACAAGTTCACGTGGTGGCGGTGGTG

GTTGGGGTGGCGCAATGTGTACATACACTTGCGTGTACTTCTCATCGATGCCGATTTCGT

TGGTGGCCTGGCAGCGATATCTGCCCTCGTCGGATTTACGTGGTGCATCGAAGATCAAAC

GGGAGCCATCTGTGTGAGCTTCGAGGGACATTTGACCGTCGACGCGCGTCCAGGTGACAG

TTGGCGTAGGATGACCGTTAACTTCGCAGTAAATCTCGTTGGGCTGGTACTCCTCGAAGG

AAATGACTTGTGGCAGGTTGATGATCTGTGACGGTGTACGCTGAGAGTCCTCTGTTATTG

TGATGGAAATCTGATCTTCGAAAACGCGCTGCGTCTGGTTGTTCACTGCGCGACAGATGT

ACACACCGGAGTCATAGATTTGCAGGTCATACAAACGCAAGACACCGCCATCCTCTTCGG

CGTTATATGGCAGGCGGCCATTGAGCTTCGACCAAGACACGAAGACGGGTACGCCACTCC

AGGCCATGCGACCGGTGCACGACAATGTCAGTGAGCCCCCAATCGGTATGATGGTAATCT

CAGGTGGTGCAATCGAGACGGTAATTTGCGTTTTGTAGTCGGGTTGTGGCGCGGGTGTTG

GTGGCGCCTGAGTGGGGCGTTCATAGTCATCTCGCCGATTGCAGTCGAGTCGTAGTTTCG

TAATGTTGCCTATTTTTCGGTTACCCCTAAACAACTGCAGTGTTTCATTGTATGCGATTG

TGACATTGGGCACGAGGTCCACGTGATAGTCATTGCAGCAGACGCACACTAGACTATCCG

GTTTGTCAATGAGTGTTGATTTTTCAGAGCTTTCGTTTTTATTTGCTTTAACATCAATTT

GACACTGTTGGCCGGAGTAGCCTGATCTGCACTGACAGAGCACTTCGCCATTGTTTTGTA

GGCTGCAGCCGACGGTGGTGTCTTCGCGGCACGGGCATGCCACGCAGCTGCCATAACTGT

CCTTGTAATGTAGTGGTTCGCACTTTTCGCACGAGTTGCCTGAGTAGCCGGCGGGGCAGC

TGCAGACCTCTACTTCGACGGCCAGTTGCGCGTCGGGTGTGCGATGCTCCACGGCGGATT

CGAGTATTACGTCGCGTATGCTAGTGCGTGTGGTTGGTATTTTGGGTGTCGCACGTATCA

ATATGTGCTCAAGGTTGGCTAGTACGCTCATTATATCCAAACGCGAGGCACGCACTGAGT

GGCCGCGTTCGTTGCGTAGCCAGTTTTCGTCTTCATGCAAGCGCACGCGGTACTCGGTGT

CTTCTTGTTCATCGCTAGATCTCGACCAGAGCAATTTCATGCCATTTCCAATGAGTATGA

CATCAGTTCCTGGTTCATAGTGGCCATATGAGTCCACATCTAGAGCGTAGGATAGATGCC

CGCCATATGAGTAGAGCTGGTTGCCTAGTACAGAGCCGCGCAGACTCCAGTATTTCTCCG

TGTACGAAGTGAAGCTGTAGGTGTACATGTTGGTGGGTAGGTCGAAGCTTAGATTTTCAG

TGTCCACAATATTGCCTTCGCCATCTGTGATCAATGGCTGACTGCTGATGAAGTCGACAG

GTATGAGTTGGCGGTACAGGGAAGCAGAGTTGCAACGGCTGCTCTTGCCGGAGCAGTAGC

ATTCTTCGCAGCCATCGGGGTTGCGTTCAGAGAGGCCATACGTGCCTGGTCGGCATTGGT

CGCAGTATGGACCCACTACATTGGGTTTACAGGCACAGTAGCCGCCGTCGCAAGACACAG

TACCATCGCGGTTACAGTAACTGCATTGTGTCTGATTGCCCGGTGCTGGTGGTGGGTAGG

GGCTTGGTCCACTATCAGGTGAGCAGTCGTATGGCGTCCTACCGGAGGCATTACCAGTGT

ATCCAGGAGAGCAGCGCTCGCAGAATTCGCCGGCGGTATTATGGTCGCAATTCATACAAA

CACCAGTTTCAGCATCGCATTGGTTTGAGTGTCCATTGCAATCGCATGGTTCGCAGACAC

CCAAGTAGAGACCGGCCTCAGTGTCACGTTTGTAGCCAGGTGCACACCGCTCGCAGGATA

AACCAATGTAGCCTACTGGACAGCGGCATTCCTCCACTTCGAAAGCGCGTTGTGTACCGA

TTTTGTTCTGTGTAGCAATGTCAAGCGAAACGTGCGTAAGCTGACCGTCCTTCGTGCTGG

TGGTGTAAGTGGCCTTGATGTAGATCGCGTCGATCTTCGAAAGCGCCATCAACAAGTGTT

CGCGATTGACCACTTGTCCGTCGGAACGCTGCCAGGCGCTTTCGATAATCGGCACGGAGT

ATGAGCTGGGTTGGTTGGGGTTTACGCCCGCTTTACGGTAGTGAATAATCGTCAAGTCTT

CACCGCTCTTGATAACCACATCGGGTGCGTTGTTGCGGGACATAAGTCCGCCTGGCAGTG

GGTTGTAGCTGAGATTGTAGGAGAGTTTGCCGCCGTAGGCAGTGATCCTGTCGCCAAGGA

ATGGCGCCGGCAGGCTCCAGTAGAGCGGATCAGAGGTCGATTGTTGACTAAATTGTAGCG

CACTATTGGCCTGTTGGAAGCTAACTTGGGATGGTTGAGAGGTTTCGTAGTCTCGAATAA

GGTTGAAACCGTGCGGTGCGCGAGAGCGTCCAAAGTTCGAAGATACCTGGTCACGATACC

AACTGCTGCTGGAACATTGTTTTGTCAGACCACTGCAGAAGCACTCAATGCAACCGGTAT

AAGTGAAAGCATTCAAATGGAAGGATTCAGGTGCGCAGGTGTCGCAACGTGGGCCGACCA

CCAGTGGCTTACACTCGCATGTGCCGTTAGGATGTGGATAGTAAGTGCCCTCGGCGTTAC

ATGTGCTCTCTGGTATGCGGTGGCAGCTGCCACCTGGGATCAGCGGGTTGCCTTGGTACC

CAGGTGCGCAGATCTCACAACGTCTGCCTGTGTACCCCTCGTAGCAGTTGCAGGTAACAT

CACCGTCAGGGCTGAGTTGACAACCGCTGGCAAAGTTATTGGCGCCAGTTTGTGGGCATG

GGCATTGACGGCATGGTATGCCACGACCGGGATCGCCATAGGTACCAGGTTGGCAAGGCT

CCGGAACGAACGGTACGCAGCGGCCCAACCAAGGACCATACTTCTCGCGTACATAACCAG

GCGCACAAGATTCGCAAGAGTCACCAACGTAACCGGGTGGACAGGCGCATTTTTCGACCA

ATGAAGCTGAGCCCAAGCCTTGGTCGTCCAGACCAGCTGAGTCCATAACAATATTGGTGA

GCTCTACTTCACGTTCAGTGGCATCAATGTAACTCAAGCGTATGAGGATGTTGTCGACAT

TAGCCAAAACCATCATAATCTCTTCGCGTGTAGCACGACGTCCGTCAGTCTTGTACCAGT

TTCCAGAGTTAAATTGTACTGAGATCTTGTTGTTGACGTTTGGTTCTGGTTGACTGCGTA

CGCGATGTGTGAGCGTAAATCCATTTCCAGTGATTATGACGTCTGGTGCCTGGTTGAGGC

GTCCATTACCCATGTAGTTAACCTCATAGCGCAGATAGCCGCCATAAGACTTGAGCTGGT

TGCCCATGTATTCGTTAGGTAGCGCCAAATACGGCTGCTCGCGACTATTATATCCCACAT

CGGATACGCGGAACTGCACGCCATGATGATGACTGAGAATATCAGGCGACTGTGCATCGT

TGATGATAATGTCACTGTATGGGTTAAGTTCAACGTTCTTGACCTTGTGCGAAATTATAG

TCGGTTGGATGGCAAAGTTGTAGAGATTAGCGCTCTTACAAGACTTCGATATGCCAAAGC

AGAAGCAGTTGATACATTCATCTTGGCGACGGGCGAGCATGTTGAAGAAGCCAGCGGGAC

AAACATCCACATGTTCACGAGCATCGACGGTCACCTGAGTATCGGGAGTCACAAAGTTGC

GTCCCTTCGAGTTGAGTATTTCACAAGAGTAGGCACCTTGGTCCTGAGTATCCATATCGG

GGCAGTAGAGTGTGCCGGTGCCAGCATGGCTCTTTGCGACACACTTTTCGGGTACGTGAC

CCCAGTTCAAACGCCAGACGATCAGTGGCACTGGTACACCAGTACCCACACAAGTCAAAT

TCAATGGATCGCCTTGTTGCAGGCGAATGTAAGGTGGTGGTGGACGAATTGGAGCAGGAG

CCATGCAACCGATTTCATCGGTACCATCGATACAATCATTTGTGTCGTCACACTGGAAGT

TTTTGGGAATGCAATGGCCACTGCGGCATTGGAATTCGTCATAGCGGCATGGCGCACCAC

TTGGCTCGGGATCGCAGCTATCTTCATCGGAGTTGTCGAAGCAATCGTCTTCACCGTCAC

AGCGCCAGATGCGATCAATACAACGCGAGTTGCGACACATGAACTGATTGGGCTGACATT

TGAGGCCGTGGCTACAGCTCTCTTCATCAGAGCCGTCCGAACAGTGTGGGATTCCGTCAC

AGATTTGAGATTTGTCGATACATTCGTTGTTGCGACAAGTAGCCTGGTTTGCGCGACAAG

CTGTGGGGGGTCGGTCGTTCGTGGGATTGGATTTTTCAACGGTTAAATGTACGGTAACTT

GTTGTCCAGGCACATGACGGGGATAGCCAGCAGCTTCACAAATATATTGACCAGAGTCCT

CAGAGCGAATGTTTGGAATCTCCAGACGACCATCGCCATTGTCTCGTGAACCGATTGGCA

GTGGGCGACCGCCAGGGCGGGTCCACTTGACACGGGCGCGCACTGGACCTTCGTCACGAC

AACGGAAGATGACTTCACGACTTTCTTTGATAATTTGACTATCAGGGTAGGTCCTCAGAT

TCAATTGTGGATTGGACGGTGATGCGTCTGGAACTCTGCACGAAAGCTCATCCGAACTGT

CGGCACAATCGGTGTAGCCGTCACAACGCGCCGCCGCATTGATGCACTGCCCGTTGCTGC

AACGGAACTGCGAAACGGAACAACCGCGTCCCGCATCATAGCTGGGCTCTGAGCAGTCCT

CCTCGTCCGAGCCGTCGCGACAATCACGATAACCATTGCAATAAGCACTGCCCGAAACAC

AATCACCGTTACGGCAGCGGAACTGATTGGCGGCACAGCGACAAATACTTTCGTCGCTGC

CATCCTTGCACTGTCGTTTGCCATCGCAGACTTGCGTTTTCGGTATGCAACCGCCGCCGT

GCAAACATTTCCACTTGTCGCTGCTGCAGGTGCACAAATTATAGTCTTCGTCTTCGCGAC

GCGCGCAATCGGCTGTGCCGTCGCAAACGCTCTCGATCGGAATGCAATCGCCATTTTGGC

ACGTAAACTCAATATTTTCGAGACATGTGTTGTTGTAGCTAGGCGCAACGGTGGTAGCAA

TACGCGTTGTGGTTGGGAACGTCGTGTTCCATCTACCAGCTGTTGGTCGCGGCGTTGTTG

TGGCAGCCGTGGTGCGCCACCATGGTATCATCTGTCCAGCCTCCGTCGTCCGCATCAAAT

TGGAGGGCCGCGCTGTGGTGCGACGTACTGCATTGTACTGATCGCAGCCATACTCGTCCG

TCATATCATTGCAGTGGTAGAAACCGTCGCAACGCAGTTGCAATGGTATGCAAACATTGT

TGCCACACCTGAATTGCTGACTGTTGCAGGGATTCCGCGCGCTGGCCTTTTGATAGAGGT

TCTGCTGAACGAGGTAGTACTCTTGCGCGTCCAGACCATCGAGCGAGGGTCCCTCTGTCG

GGGCGGGTTTGCGCTGGTGTGGCGCGTATTCGCGTATGATTTCATCCTCGTCGAAGATGT

TCCGCTCAATATCCTCAATAGCACAATCCTTTTCATCTGAATAATCTACGCAATCATAGA

TATTATCGCATAACTCACGCATCGGTATGCACTGACCGTTGTCGCATTGGAATTCGTTGT

ATCTGCATTCGGACTCACCACTGCCTTCACATTGGTCCTCATCGTCGCCGCCTATGCAGT

CGGTCACGCGATCGCAAACCTTCGTGCTGCTAATGCAGCTTTGGCCATCCCTGCAAAGGT

ATTCGTCCTCGCGGCAACCGATAAGGCAGCTGGCGTCCTCATCATCGCCATCGGGGCAGT

CACGCACGCGATCACAGCGACGCGCACTGTCAATGCAATTGCCGGCACGACATCTGTACT

CATTCGGTGAGCATTCTTTCGCTTCCTCGGTTGGGCAGTTCGCCTCATCGGAGCCATCGA

GACATTCCTCTGCGCCATTGCATAGGTACTCACGGGGCAAACAACGTTCATCACATTTAA

ATTCGTATTCGGCACAAACCTTTTCATAGTCTTCTTCCTTGCCGTAGCCGTCTTCGCCTC

CGCCATTCTCCTCTTCGGCGCATCCTATTTCGTCTTCACCATCTGGACAGTCTTCGTGAT

CATCACAGCGTTGTTCGTCACATATCTGCACACCGCTTTCGGCGCAAGTGAACGAAGCAT

CACCGCGGCAACCGAATGGTGTGAAAAGCGTAGAGCGATCCGTCGATATGCCATGCGTGT

CGTCGCCAGAACCCTCATCAAAATCAGAGCCATGAGAAACGCCTTCCGGTACTTCAGTGA

CATCGGGTTGTGCAGGTCGTATGGGTGTTTCGGAGTCAACAGGTTCTTCGTAAGGGTCTC

GGCCACGTTCGTATTCATTACGACGACGTTCTTCTTCTTCATATTCAGCACGTAGACGTT

CGTCTTCAGCGCGACGGAGTTCCTCTTCTTCTTGTTCAGCACGTAGACGTTCATCTTCGG

CACGACGACGCTCTTCTTCAT

**tBLASTn(First hit)**

Score = 856 bits (2212), Expect = 0.0, Method: Compositional matrix adjust.

Identities = 445/565 (79%), Positives = 482/565 (85%), Gaps = 14/565 (2%)

Frame = -3

Query 1 EYKCRAGNCIDSARRCDRVPDCPDGDDEDASCLIGCREDEYLCKDGMRCISNSKVCDRVT 60

EY+CRAGNCIDSARRCDRV DCPDGDDEDASCLIGCREDEYLC+DG CIS++KVCDRVT

Sbjct 11941 EYRCRAGNCIDSARRCDRVRDCPDGDDEDASCLIGCREDEYLCRDGQSCISSTKVCDRVT 11762

Query 61 DCIDADDEDGCEGSGESACRYNEFQCGNGQCIPMRELCDNIYDCVDYSDERDCDLEDIGR 120

DCI DDED CEGSGES CRYNEFQC NGQCIPMRELCDNIYDCVDYSDE+DC +EDI R

Sbjct 11761 DCIGGDDEDQCEGSGESECRYNEFQCDNGQCIPMRELCDNIYDCVDYSDEKDCAIEDIER 11582

Query 121 NIFDEDEIIREYAPHDHRVLTTEAPVLEGLDAKEYYLYNSGLYEKSNAQHSCNERQFECG 180

NIFDEDEIIREYAPH + TE P L+GLDA+EYYL LY+K++A++ CN +QF CG

Sbjct 11581 NIFDEDEIIREYAPHQRKPAPTEGPSLDGLDAQEYYLVQQNLYQKASARNPCNSQQFRCG 11402

Query 181 NKVCIPLQLRCDGFYHCNDLTDEYGCDQYRPESRRTTLPPPKLVRTTHS----PWWNtta 236

N VCIPLQLRCDGFYHCND+TDEYGCDQY RRTT P L+RTT + PWW TTA

Sbjct 11401 NNVCIPLQLRCDGFYHCNDMTDEYGCDQYNA-VRRTTARPSNLMRTTEAGQMIPWWRTTA 11225

Query 237 attttsr-----psttttDANIVTPVVPSYNNNTCLENLEFACRNGDCIPIESVCDGAAD 291

ATTT +T T I T V PSY NNTCLEN+EF C+NGDCIPIESVCDG AD

Sbjct 11224 ATTTPRPTAGRWNTTFPTTTRIATTVAPSY-NNTCLENIEFTCQNGDCIPIESVCDGTAD 11048

Query 292 CRQREDEDYNLCNCSSDKWKCLRGGGCIAKTQVCDGRRQCKDGSDESICRCAANQFRCRN 351

C +REDEDYNLC CSSDKWKCL GGGCI KTQVCDG+RQCKDGSDESICRCAANQFRCRN

Sbjct 11047 CARREDEDYNLCTCSSDKWKCLHGGGCIPKTQVCDGKRQCKDGSDESICRCAANQFRCRN 10868

Query 352 GDCVSSNAQCNGYQDCRDGSDEEDCSEPAYVV--PCTGSQFRCNNGQCINAAARCDGYTD 409

GDCVS +A CNGY+DCRDGSDEEDCSEP+Y C+ SQFRC+NGQCINAAARCDGYTD

Sbjct 10867 GDCVSGSAYCNGYRDCRDGSDEEDCSEPSYDAGRGCSVSQFRCSNGQCINAAARCDGYTD 10688

Query 410 CADSSDEISCTV-DFPASNPELNLKTYPESQIIKESREVIFRCRDEGSLRARVKWTRPGG 468

CADSSDE+SC V D SNP+LNL+TYP+SQIIKESREVIFRCRDEG +RARVKWTRPGG

Sbjct 10687 CADSSDELSCRVPDASPSNPQLNLRTYPDSQIIKESREVIFRCRDEGPVRARVKWTRPGG 10508

Query 469 RPLPIGARDNGDGRLEIPNIRVEDSGPYICEAAGYPRHVSGQQVTVHLTVEKLNPDNERP 528

RPLPIG+RDNGDGRLEIPNIR EDSG YICEAAGYPRHV GQQVTVHLTVEK NP N+RP

Sbjct 10507 RPLPIGSRDNGDGRLEIPNIRSEDSGQYICEAAGYPRHVPGQQVTVHLTVEKSNPTNDRP 10328

Query 529 PTACRAYQATCRDNQCIDKSQICDG 553

PTACRA QATCR+N+CIDKSQICDG

Sbjct 10327 PTACRANQATCRNNECIDKSQICDG 10253

**Conserved domains**

**
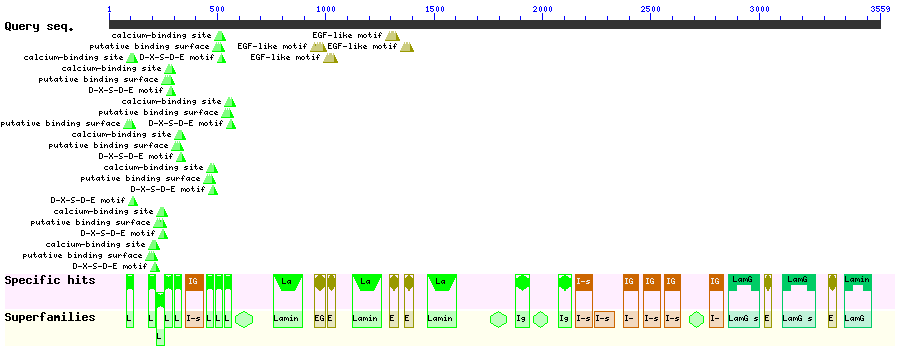
**

**Innexin**

**>TRINITY_DN33133_c1_g1_i6 len=2819**

GTTTAAACGTTATTTGCGCTGCTTGTTAGTTTAGATTTATTGTTGTTATTATTATTATTG

TTTATTTTTCACTATGCTCTCCCAATGTCTTTAAAGTTTACGCTCTATACTTGAACTTCC

CCTCATCAACATTCATTCCGCCTGTACGCCCGTCTTCTGACATGCATTTTTGCATGCACT

CGTAAATTGCTAGCAGTATCAATTAAATTCATAAATTTCTTAAACAATGATCACAACTCT

TATTAAATATTTGTATAAAAAAATTGAATTCTCATTTATGATAGTGCTTTCAACTTCTCG

TTTGTTTGTTTTTTGTTTGTCTGTTTGATTTAAGTGTTTGTGTGTATATGAAAATATGTG

TGGCTTTCTCTCACAAACAGTATACATATGTATGTGTATGTACCTATGTATTGTTGTTTC

TACATTTTTACAAAAATACATTTTGTCATCACTTTTGTATTTATTTTCCGGTTTTTTTAT

CTTCATTAGCAAAAAACTAACAGATTCATAAGATTCTTTTCCTTTTACTCCTATAATTAA

TTTCTATTTATTATTATTATTATTTTGTTTTTTTTTGTGTAAAAATCTTTTCTTTTTTTG

ATAAAATTTTTTCATTGTAAAAATGCTTGAACTATTTGTATGATTTGTCTCTTTTACTTT

AGAATGTTAACTTTACAATTTTTCGATTTGTAGGCAATTTGAATGGAGTGTTGTTGTTAT

ATATTCATAGTCGATATTTTGTAGACTTTGCTGTTTCATCGTTTTTGTTTTTTTTTGTTT

TTGCTTTTGTATAGAATTAAAATTATATAATTACTTCTTATTGCTTAAAATATTTATTTG

TATACAAAAAGACAAATATCAGAAATTCGCAATTTCACAAAATTTAAACAAGAATTGACG

TGTCACAAAATCCGTTTCGTCTTCACTTCGTGCATAGCACTTAAAAAAGTGTTTTCCAAT

AGCGGTCCTCTCTCAGCAGACAATGGCAAGCTGTTGAGAGTATTTGAGAGTGAGAGAAAT

CCTCACAAACAAAATTAACTGTCATTCGGAGGGGACATAAAACTGTTAGCCGCTCCACTT

GTGCAAAACATCATTAGGCACACCACAAATACACCTATACATATGTACGTATATATACAT

ATATCATAAGGCGAAAAAAAAAAATTCGCAGATTTCTTTGACACGCCAATTTTTGTCTGA

TTTGCGGTCCAGGAATCTCATTGTGCTTGTGTGTATACAAACAACTCGTAATTGTGTGTG

TGTCTGTATAGGCTATTTATTATTTTAAGTGTGCGCTTGTATATTTGTGTATCGTTTGTT

GTTGTTGTTTTTTCTTCTTTTTTCTTGTTGTTGTAGATATGTATGTAGTTTGTATGTGGC

ATTCATGTATGTTTGTATGTATGTATAAGTGCTGGAGGATGCTGAAACATCTTAGTTTAG

TATTCAGTGGCACTGACGTTGCGATTCGCAAGAGAAAATTGGCCATTTGCGGCCATTTAA

GCTTCGAATGGTTGCTTGCTGCTCGAATGTTCGCCCATTTCACGCGCCAAATCCGCTATC

ACCTCCTTATAGATCAATGGATCGATATTCTTGCCCAGCTGATACAGCAGGAACCAATCG

CCAATATTACACTTATTTGCCACCGCCTCAACCTCTTCGCTCTCGGCCAAACGGGAACGG

GCACGCAACAGCAAATGACGTAGTTTAGGTCCAATGATAACAGCCATGCGATAAACCAAT

GAAATGCCAGACAATATGCTCAAAATGATGAACCAGAACCACAAGAATACGTAGATTTTC

TCATTGACAATATTCAATGGCAACACACAGAGACCGTCGAATTTCTGCACCGAACCGGAT

GGACCGTATTTGTGGAAGGTGCATTTGGTGACTTTCGGAAAGACACGTGCCATCGGATCG

ATACGTTCATCCGGTTCCATTTCGGTGAATTTCAGCACATCACTGCCGTATGTGGAGAAT

TCGCCATCCAAAAAGAAATCCACAAAGAATATTTGTCCAATAACATTGGCAAAATTCAAA

GCCTCACAAACGAAAAAACGGAATGCATAGAAGTTGTGACGATTCAAATTGCCCATAAAA

TATTCGACGAGTATCTTCTTGCGATCATTCTTACAATCATCATTCACAATGGGACAATTC

AAATCCATAACCAACATTTTGAGGCGACCACCTTCCCATGATTTCCACAAATAGCGAGGC

ACATAGAAGAGTATGGCTTGGAAGAAGAGCACGAAACACACCCACTGATAGTATTTGTGA

TATTTCACTTCGTCCTCACCCTCTATATGCGAACCAACACCCGGCTGGACAACATCACGT

CCGGTGATGCCCGTCAAACGTTCCGGTACGGTAAAGGTGGAATAAATCCAGCAGTAAGTG

TCCATCACACCGAGCGGAATCTCATCGACAATGCAATCGATGGGATCGCCAATGTATTGA

CGCGAGGTGACGAGCAGCGAGAAGGCAATCAATATGATCACCGTCGCCTTGTAGTGCATG

CGGAAGACATTGTTGTCGATGCAGACCTGATCGATCTTCAGCAGCCCCTTGACGGAGCCG

AATACATCAAACATTTTGGCGTGCTTTGTTGCGTGTTAAATAAAAGTCAACACAAATTTT

TCAAGAAAATTTTAAAAAGAGGAACCGTCGACGTTACTACGGGAATGTTAGCGAATGTGA

TTTTGTTAATTCGAAAAAAAGGCAGTGCTTGTAGCGACGCGCAATGGCAATACTTTCGTT

TGGAAATTTAGTACTCGTTTGTTTGCGGTATGCGATATGCGGTTTGTTGTGTTTTACTT

**tBLASTn(First hit)**

Score = 644 bits (1660), Expect = 0.0, Method: Compositional matrix adjust.

Identities = 342/367 (93%), Positives = 357/367 (97%), Gaps = 2/367 (1%)

Frame = -1

Query 1 MFDVFGSVKGLLKIDQVCIDNNVFRMHYKATVIILIAFSLLVTSRQYIGDPIDCIVDEIP 60

MFDVFGSVKGLLKIDQVCIDNNVFRMHYKATVIILIAFSLLVTSRQYIGDPIDCIVDEIP

Sbjct 2594 MFDVFGSVKGLLKIDQVCIDNNVFRMHYKATVIILIAFSLLVTSRQYIGDPIDCIVDEIP 2415

Query 61 LGVMDTYCWIYSTFTVPERLTGITGRDVVQPGVGSHVEGEDEVKYHKYYQWVCFVLFFQA 120

LGVMDTYCWIYSTFTVPERLTGITGRDVVQPGVGSH+EGEDEVKYHKYYQWVCFVLFFQA

Sbjct 2414 LGVMDTYCWIYSTFTVPERLTGITGRDVVQPGVGSHIEGEDEVKYHKYYQWVCFVLFFQA 2235

Query 121 ILFYVPRYLWKSWEGGRLKMLVMDLNSPIVNDECKNDRKKILVDYFIGNLNRHNFYAFRF 180

ILFYVPRYLWKSWEGGRLKMLVMDLN PIVND+CKNDRKKILV+YF+GNLNRHNFYAFRF

Sbjct 2234 ILFYVPRYLWKSWEGGRLKMLVMDLNCPIVNDDCKNDRKKILVEYFMGNLNRHNFYAFRF 2055

Query 181 FVCEALNFVNVIGQIYFVDFFLDGEFSTYGSDVLKFTELEPDERIDPMARVFPKVTKCTF 240

FVCEALNF NVIGQI+FVDFFLDGEFSTYGSDVLKFTE+EPDERIDPMARVFPKVTKCTF

Sbjct 2054 FVCEALNFANVIGQIFFVDFFLDGEFSTYGSDVLKFTEMEPDERIDPMARVFPKVTKCTF 1875

Query 241 HKYGPSGSVQTHDGLCVLPLNIVNEKIYVFLWFWFiilsimsgisliYRIAVVAGPKlrh 300

HKYGPSGSVQ DGLCVLPLNIVNEKIYVFLWFWFIILSI+SGISL+YR+AV+ GPKLRH

Sbjct 1874 HKYGPSGSVQKFDGLCVLPLNIVNEKIYVFLWFWFIILSILSGISLVYRMAVIIGPKLRH 1695

Query 301 lllrarsrlaeseevelvANKCNIGDWFLLYQLGKNIDPLIYKEVISDLSREMSGDEHSA 360

LLLRARSRLAESEEVE VANKCNIGDWFLLYQLGKNIDPLIYKEVI+DL+REM EHS+

Sbjct 1694 LLLRARSRLAESEEVEAVANKCNIGDWFLLYQLGKNIDPLIYKEVIADLAREMG--EHSS 1521

Query 361 HKRPFDA 367

K+PF+A

Sbjct 1520 SKQPFEA 1500

**Conserved domains**


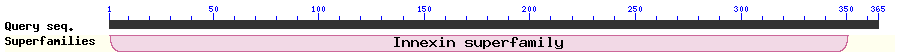


**Low density lipoprotein receptor (LDL)**

**>TRINITY_DN19392_c0_g3_i1 len=2365**

CCGATTCGTCAGCACCATCGGCGCAGTGATCCCAACCGTCGCACAGTAGTGTGGCCGAGA

TGCAGAGCTTATTGATGGGACACTGAAACTCGCCCGGCCGCTTGCAGCAGTCCGCCTCAT

CATGACCATTTGCACAATTCGTAGTGCCGTCACACACCAGCGCCTTATCGATGCATTCGC

CCGATTGACAGCTAAACTGATCGGGTCGACATGAGGGACAGCCCACTTCATCCGATTTAT

CGGGACAATCACTTTGGCCATCGCAACGCCATGATGCTGGTATACAATCTTTATTGATAT

CACTGCTAGAGCCATCGCCCTTAACAGGCGCCGCACAAGTGAAATGATCCGGCCCGCAAG

CAGGTAAAGCGCCACAATTTTGTCTGTCCTCAAGCAACATAAGATTCTTCGGACAAGAGC

AGATATCACGTACGCGTCCCTCGGATGAAGCAATACAAATATGCGAGCATATGGTGCGGC

TATGTAAACAGGTATGATTGCGAAATAGTTTCGACTCGGGCGTCCAAACGGCCACAATGT

CAGTTATTTGTGGTAGGCGTTGCATTTCAGGGCGACGACCATCACCGTTTACGGTTATGC

GCTCCACACCAGTTTTATCGTCCAGCCAATAGACGAAACCTTGAATTGCGGCGAGCGAGA

TGACTTGCGAAATGTGCGTGGACACGAGAATTTTCTTATTTTTGCCATTTATGTCCATCG

AATCAATGCGCTTGCCATGCGCATAGTAAACCATGTCACTTTGTTGATCCACCGCAAGCG

CTGTGACTCCCTCCAGCTTAAAAGCAAGCACTACACGCTCAGCGCCGTCGATGCGCGAAC

GTATGATGGCTTGCTGACTACCCACATCAGTCCAAAACAACAGACGCTTCATGGCGTGCA

CGGCAATGTTGCGCGGTTTCTCCGAGTCGCCTGTATCTATGACACCTATGGAATCACCGT

CGAATCTCGTCACATTGATGCTGTTCGAATGTGAACAGGTCCAGAAGAGTAGACGACCAA

TCACATCCAATGCAATATCGAAGGGCTGCGAACCCGAGCCTACCAAAACCTTCACAAAAG

TGCTGTTCGCGGGTGAGTGCTTGATGCTGTGGCTACGTCCTTCCACCCAGTAAATGTTAT

GTGAAATCGGGTCGTATTCCACCGCTCGTATATTCTTGCCCGACAGCGGCAATGGCACAT

TCGGACAGTCGCTCGTGTTCGGCAGTAAACGACCAAAGCTATTACGCTGGCTATAGATGA

TATAGTTCTTGGGTGGTAGACAGGACATGTTATCCTTGGACAAAGTGTAGTGCGTTGGAC

AGGCGCAAATCATACCGCGACGCGTCGGTTGTGCCAAACACAAATGGGAGCAGCCACCAT

TGTTGATTTTACATGGATTACTGCCTGTCTGACGATTTTGGTAGAATATCAGCAACGAAC

GTATGTAAGTCATGCCGCTGTGCACAAGACTGCGATTCTGTCCGGTCATCTTGTGCACTC

GTTCAATGTCACCCGTATTCCAATCGCTCCAGTAGACAAAATCCTGATAGAGCGACATTG

CATAGGGCTCGTCGACATCGGTGTTGATCAGCACCTGGCGTTTCTTGCCATCCCAATCGG

CAGATTCGATTTTTGTGGGTCGCGATTGTGTTGCCCAGTAGAGACGCCGCGTTTCTGGAT

CTAACGTAAGTCCAGTCGCATGATTCGCACCCGAAATTATTGTCTGCAATTCGGACCCAT

CCATGCCGGCACGTCTTATCGAATCTGAAGGCGACTCGATCCAGTACATATAGCCACGTT

TGGGCTCTAATACTAAAGAACGCGGCTCCTCTACGCCTTTCCAGAGCAGCACGCGCCGAC

TGGTGCCATCCAAACGTGCAACTTCAATGCGTCGCGCTTCAGAGTCGGTCCAGTAAATAT

TGTGCGCCAACCAATCAACTGCTATACCTTCGGGACAAATGAGTCCGGCATCGATAATGC

GCTGCACGAAGGAACCATTCAAAAAGGCGCGGTAAATACATTTCGACTTCTGATCAGTCC

AGTAGATGCGTTTGTCTGCCACATCCACATCCAGCGCATGTGCATCGCGCACATCTTTAA

AAGGAATCTTCTCATCATTATGATTACCCTCGTTGTTGTCAATCGAAATTCGTCCGATGT

GTTCCTGTCGCGAAAACAGCAAAAATGCTTCGGGTATGACACATGTTTTCTTGTCATTCG

CCAGCTCGTACTCTATGGAACAGCGACAGACGTAGTCGCTTGGCCGATTGAGACAAAGTT

GCGTGCAACCGCCGTTGCGTACCGCGCATGAATTCGAACCCTTAACGTCGCGTAACCGCG

TCACTTTCAGACCCATCAGATCGGG

**tBLASTn(First hit)**

Score = 1407 bits (3641), Expect = 0.0, Method: Compositional matrix adjust.

Identities = 652/790 (83%), Positives = 728/790 (92%), Gaps = 6/790 (1%)

Frame = -1

Query 649 PDLMGLKVTRLREVRGQNACAVRNGGCSHLCLNRPRDYVCRCAIDYELANDKRTCVVPAA 708

PDLMGLKVTRLR+V+G N+CAVRNGGC+ LCLNRP DYVCRC+I+YELANDK+TCV+P A

Sbjct 2365 PDLMGLKVTRLRDVKGSNSCAVRNGGCTQLCLNRPSDYVCRCSIEYELANDKKTCVIPEA 2186

Query 709 FLLFSRQEHIGRISIEYNEGNHNDERIPFKDVRDAHALDVSVAERRIYWTDQKSKCIFRA 768

FLLFSRQEHIGRISI+ NEGNHNDE+IPFKDVRDAHALDV VA++RIYWTDQKSKCI+RA

Sbjct 2185 FLLFSRQEHIGRISIDNNEGNHNDEKIPFKDVRDAHALDVDVADKRIYWTDQKSKCIYRA 2006

Query 769 FLNGSYVQRIVDSGLIGPDGIAVDWLANNIYWSDAEARRIEVARLDGSSRRVLLWKGVEE 828

FLNGS+VQRI+D+GLI P+GIAVDWLA+NIYW+D+EARRIEVARLDG+SRRVLLWKGVEE

Sbjct 2005 FLNGSFVQRIIDAGLICPEGIAVDWLAHNIYWTDSEARRIEVARLDGTSRRVLLWKGVEE 1826

Query 829 PRSLVLEPRRGYMYWTESPTDSIRRAAMDGSDLQTIVAGANHAAGLTFDQETRRLYWATQ 888

PRSLVLEP+RGYMYW ESP+DSIRRA MDGS+LQTI++GANHA GLT D ETRRLYWATQ

Sbjct 1825 PRSLVLEPKRGYMYWIESPSDSIRRAGMDGSELQTIISGANHATGLTLDPETRRLYWATQ 1646

Query 889 SRPAKIESADWDGKKRQILVGSDMDEPYAVSLYQDYVYWSDWNTGDIERVHKTTGQNRSL 948

SRP KIESADWDGKKRQ+L+ +D+DEPYA+SLYQD+VYWSDWNTGDIERVHK TGQNRSL

Sbjct 1645 SRPTKIESADWDGKKRQVLINTDVDEPYAMSLYQDFVYWSDWNTGDIERVHKMTGQNRSL 1466

Query 949 VHSGMTYITSLLVFNDKRQTGVNPCKVNNGGCSHLCLAQPGRRGMTCACPTHYQLAKDGV 1008

VHSGMTYI SLL+F RQTG NPCK+NNGGCSHLCLAQP RRGM CACPTHY L+KD +

Sbjct 1465 VHSGMTYIRSLLIFYQNRQTGSNPCKINNGGCSHLCLAQPTRRGMICACPTHYTLSKDNM 1286

Query 1009 SCIPPRNYIIFSQRNCFGRLLPNTTDCPNIPLPVSGKNIRAVDYDPITHHIYWIEGRSHS 1068

SC+PP+NYII+SQRN FGRLLPNT+DCPN+PLP+SGKNIRAV+YDPI+H+IYW+EGRSHS

Sbjct 1285 SCLPPKNYIIYSQRNSFGRLLPNTSDCPNVPLPLSGKNIRAVEYDPISHNIYWVEGRSHS 1106

Query 1069 IKRSLANGTKVSLLANSG-QPFDLAIDIIGRLLFWTCSQSNSINVTSFLGESVGVIDTGD 1127

IK S AN T V +L SG QPFD+A+D+IGRLLFWTCS SNSINVT F G+S+GVIDTGD

Sbjct 1105 IKHSPANSTFVKVLVGSGSQPFDIALDVIGRLLFWTCSHSNSINVTRFDGDSIGVIDTGD 926

Query 1128 SEKPRNIAVHAMKRLLFWTDVGSHQAIIRARVDGNERVELAYKLEGVTALALDQQSDMIY 1187

SEKPRNIAVHAMKRLLFWTDVGS QAIIR+R+DG ERV LA+KLEGVTALA+DQQSDM+Y

Sbjct 925 SEKPRNIAVHAMKRLLFWTDVGSQQAIIRSRIDGAERVVLAFKLEGVTALAVDQQSDMVY 746

Query 1188 YAHGKRIDAIDINGKNKKTLVSMHISQVINIAALGGFVYWLDDKTGVERITVNGERRSAE 1247

YAHGKRID++DINGKNKK LVS HISQVI++AA+ GFVYWLDDKTGVERITVNG+ R E

Sbjct 745 YAHGKRIDSMDINGKNKKILVSTHISQVISLAAIQGFVYWLDDKTGVERITVNGDGRRPE 566

Query 1248 LQRLPQITDIRAVWTPDPKVLRNHTCMHSRTKCSHICIASGEGIARTRDVCSCPKHLMLL 1307

+QRLPQITDI AVWTP+ K+ RNHTC+HSRT CSHICIAS EG R RD+CSCPK+LMLL

Sbjct 565 MQRLPQITDIVAVWTPESKLFRNHTCLHSRTICSHICIASSEG--RVRDICSCPKNLMLL 392

Query 1308 EDKENCGAFPACGPDHFTCAAPVSG---ISDVNKDCIPASWRCDGQKDCPDKSDEVGCPT 1364

ED++NCGA PACGPDHFTCAAPV G SD+NKDCIPASWRCDGQ DCPDKSDEVGCP+

Sbjct 391 EDRQNCGALPACGPDHFTCAAPVKGDGSSSDINKDCIPASWRCDGQSDCPDKSDEVGCPS 212

Query 1365 CRADQFSCQSGECIDKSLVCDGTTNCANGHDEADCCKRPGEFQCPINKLCISAALLCDGW 1424

CR DQFSCQSGECIDK+LVCDGTTNCANGHDEADCCKRPGEFQCPINKLCISA LLCDGW

Sbjct 211 CRPDQFSCQSGECIDKALVCDGTTNCANGHDEADCCKRPGEFQCPINKLCISATLLCDGW 32

Query 1425 ENCADGADES 1434

++CADGADES

Sbjct 31 DHCADGADES 2

**Conserved domains**


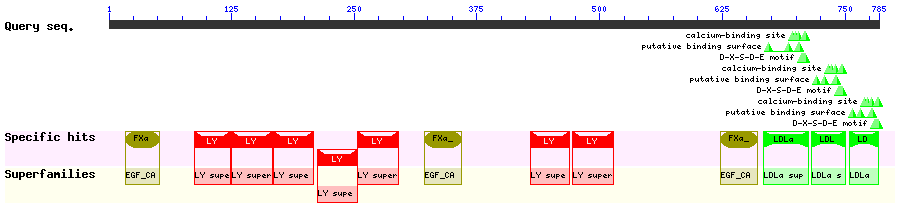


**TRF2**

**>TRINITY_DN32249_c1_g1_i3 len=3950**

TTTGCTATAAACATTTTATTAAAAGAATTTTTCCAACAAAATTAAATATTATTATGTAGA

CAATTCGCTTATAATTTTACATACTATGGGTAAAAAAGTACCCAGTCAAATAAATTGATA

TTTTCTCGTATATTGTTAATAATAATGGATGGAATGATAAAAAAAGTGGAAAAATATATT

TGATAACGTTTTAATAATTACTTAGTCGCCCTATTTTCTATCGCTATATTTTTCTCACTC

AAAAAATTCAATTTTTTGACATCTGATAACTCCCAGTGATTAAGGGAATCGCAATGTGGA

CCCTGCTGTGCGAATATTGTTGCATTCTTAAGCTAAAAAAAAATATTCAAAAAACATGTT

CATAATTCTTACTAAAATTTAAAAAATATATAAATTTTCATAGTAGGGCTATAATTGGTT

CTGATTTAAAATTTATTTTAAGAGATTCTTTTCTGTTACAATAGTAAACGTTGGACGGAA

TGTTATATATAAGGCACTCACTACTTATGCTTACAGACTATTGAAATTATATTCAGCAAA

AATATTTGTTGCTTATTCGTCATATGTATGTGTGTACATATATAAAAGCATAGATTTTTG

TTCAATAACTTGTGCTTACAATTTGTACGCGCATGTACCCTATTTATTATGCTTATATGT

ACTCAGAGCTGGGTCGTAAAATAATTGCTTTGAGAATGAAATGCTTCTAATCAACAACGA

AGGAATTTATTACTTTTTCAATGCCTTAGAAGTATTTTTTTCAATCCCATTAGCTACACC

GACCTCGATTATTCTTTCCCTACAACAACTTTTACTAATATTCTTAATTCGATGGCCTAG

TTTAATCATAATTGTTACATAATCTGTAAAAAGTTTTTGTTTCATAAACTTGATAATGAA

ATAAATAAATAAAGAATATCAAGACTCGTTTTTAAGTTTTGAGAACAATAAAAATTTCCC

ATTGCACATTCGTAAATTAACTTTTAGTTTCTCAATTACTTTTATAATTCCTTTGTGGAT

TTGAAAAAAAAAACTACTAAAGTACTCTTGCAATTTTAGATGTTGGAAAAAACTTACATT

GCTAGCACATTATTAATTGCAATTGCAAAATATAATTCCTTGTAATGGAATTGCAAAATA

GTTACTTCTGAATTTACTTCTAAGGAATGGAAAAAGCAACAATCACTTTCCTGTGGACTG

AAGGAGTTGATTACTAATTACAATTACTGAGGAATGCTTATACTTAACTATTTGCTTGTA

AGAGCGCTAGTTTTGGGATATTTTGTTTTTTGTATGTATATTTGATATGTAAACTGGAGG

CAACTCGAATTCGTCAAACCCTTAGTAGTACAGCACTAATTGCGAGAAAGAGTGGCAATG

AAGCAACTAATTGGCCTGCCCCAGCATAACAGTCGGTGATGCGGCGAGCACGCATAAAGT

TGCTGCCCAAGTATATATCATACCGTCGTTGCTCGGGTGGTATGACACGCAACTGTCGCG

TTGCATCTTGGAAGATGAGGTCGTAAAAACCCATCGGTGAAGAAAACATGCTGAAGGTGA

ACGAATCGACATCCTTTCGACCATACAGTTGTAGGGCGTAGGTTAGTAAATTAATGTATG

CATTGACTTGGGTGTCATTGTAGTTGACGCCACCACGCGTTACAATCGCATTGGCTTTTA

CCTTACCCAAATTACACTTCCTGTAGTCTTGCAGTTCTGCTCGAGTACCATCAGTGCACA

GCAGTTCAAAATCGTCATTAAGCGCATTTCTTGCCCACCACTCTTTGCGTTTGCCACCCG

TACTCTCCATAACGGTTGTGTGCTTCATGAAAGCCACATGACCACCTCCCTCCACGAGGC

AACGGAATGCACCAGTGTGGCCATAGTAGTCTTCAGACGCATCACGTCTGCAATACCGAT

AACTTGTGCCATGACAAAGATCACACATGCTATCATAAGGTACGCCAGTGTTATATTCAT

TACTGATAGCTCCTGGTACACAAGATTTGGTGAAGTATTCGGCTGCAGCACGCACCGAAT

CACAACCATATGGGCGTATCCATCCATTTGAAATAAGGAATGCCATTGGGTAGGTCCAAC

CAGCCGCAGTATTAATACCAGTGTGACAAGTATATTTGCCTTTGAGATAAGTCAGTTCGG

TATCGGGATCTTCTTCCTTAGCAACGGCTACAACATAATACTCTGGCTCGTCCAAATTGT

AAACCTCAGACATAAAAGGCACTAATTCATAATTTAAACCTCCGGTATATACATCACCAG

CGTCGAATACTGCGATGTCGGCTTTGCCAGATTGGATCCATTGCATGCAATTTATATGCG

AGTGCATCTTCTTGCATATTAGTTCAGGTTTTAAGAGCTGAGCTTTTAATGCCGTCTTCA

TTTTGATGCATTTCTCTAGTTCAGGATCAGAGGTTACACATAGAGTCATACTTGGTACTG

GACACTCACGTATGCCATAGATATACTGCATGGTATTTTGCAAATATTTACTGAAGGATT

GATCATCTTCTGCGATGGGAGATAACGAACGAGCCGAATCTTGGAACATCAGATTCGCCT

TGCCATACCGTCGTGACTCAAATATCCGGAACTTTTCATACAAAATGGATTCGTTGGTAC

CTTCCCCGTAGTTCCGTTCTGTTGTGAAACTCGAATCTAAGCGATCATTACGAAAACCGC

TGTTCCCACCATCATAACTATTTCGATTATATGTATTGGATGTGTCATATGGGTTATTGA

TATTTACACCATAAGGGTTGCCGTACTGATCTTTGTTATTATTGTTATTGTAATTGAAGT

TATTGTTGCCATATTCCGTATTACCGCGCAAGTTCTGCTGTTCGGCTTCATCGCGCAAAG

CATCTGAGTAAAGTTCAATTATGCGACGCAAAAATTGTTGAAATTTCTTTCGCTCAAGTG

AGTTTCTAGCAGATGAAGTAACTATAGCATCGGAAGGAATAATACCCCAGTTGCAATGCC

GATATTCGGTTACAGGCATGCGACGGCCGTCTCGACAAAGTAGTTCAAATCGATCGGGTG

ATAAGCTTTTGAATTCATCAGTTTGTAACATTTCTGTAACGGTTAAGTCGCGTAAAAAAG

CTACCTCTCCTGCTTCCAGAAGACAACGGAAAGCGCCGTCATAGCCATAGTATGGATCTC

TCGCTGAACACCTGCCACCAGGTATTTTACCAGTGCATAGTCTACAGAGTTTATCTGAAT

TATCGCCAATTGGATTGTATTTGTTGATCAATGAATAAACGGCACAAGAATTATTAAAGT

AGTTTGCTGCTGTCTTTACTTGATTATTGCAGTCCACTATTTCCATCCCTCCGTCACGTT

GCAACGTATATATAGGCACAATCCATCCGGCCATGCTACCCACCCACGGGAAGCAGGCAC

GTTTATTTCGAAGATCGCGTAAACTGTAGACATCTTGTAGTGTATCTTTTTTAATTACAG

CCACAGCATGATAATTCGTCAGATTTCCAACAAACTTCTCTTGCATTATGGGCACTAGGG

ATTTGTATCTACCTGCAGAGAATACGTCGCCAGCATCAAGCGTTGTTACATGAGCTTTGT

CGCGATCAATGTGGTGAATACATTCATCGGCGCTGTACCCCAAAAAACACGTCAAATTTA

GAAATGCATCATCGAAAATGGCTCGATCTCGTTCGATAGCAACAGTCAGGTTCTGACATT

TATATTGTTCTTCTAAGCTTTTCGTGCACCAAATTAGATTCTTCACTTGTTTTTCATTTT

CATAGTGGTGCTGAGAATCTATGCACTTCTCTTGCAGCAGGCACAGAAAAATAGTTAGCA

AACATATTTTAAACATGTTCACTATGTTTCCTAAAAAAATAAACCACCTAAATATATTGT

TTACTGCAACTAATGAAATGACACACCCAACAAAATGTTTAGAATGGCGG

**tBLASTn(First hit)**

Score = 1307 bits (3383), Expect = 0.0, Method: Compositional matrix adjust.

Identities = 616/812 (76%), Positives = 709/812 (87%), Gaps = 23/812 (3%)

Frame = -2

Query 6 VFVALVGALCFTLANAQHHYDEHKTTR-MVWCTKSQAEQYKCQNLTVAIERDRALFDEVF 64

+F+ L+ C ++QHHY+ K + ++WCTKS EQYKCQNLTVAIERDRA+FD+ F

Sbjct 3832 IFLCLLQEKCI---DSQHHYENEKQVKNLIWCTKSLEEQYKCQNLTVAIERDRAIFDDAF 3662

Query 65 LNLTCFMAYSADECIHHIDREKAHITTLDAGDVFTAGRYNSLIPIMQEKLEGGFADYQSV 124

LNLTCF+ YSADECIHHIDR+KAH+TTLDAGDVF+AGRY SL+PIMQEK G +Y +V

Sbjct 3661 LNLTCFLGYSADECIHHIDRDKAHVTTLDAGDVFSAGRYKSLVPIMQEKFVGNLTNYHAV 3482

Query 125 AVIKKGSLPDLNNLRDMRNKRVCFPWVGSLAGWIVPIHTLQREGGMEVVDCNNQVKTAAS 184

AVIKK +L D+ +LRD+RNKR CFPWVGS+AGWIVPI+TLQR+GGME+VDCNNQVKTAA+

Sbjct 3481 AVIKKDTLQDVYSLRDLRNKRACFPWVGSMAGWIVPIYTLQRDGGMEIVDCNNQVKTAAN 3302

Query 185 YFNNSCAVYSLSDKHNPIGDNSDKLCTLCTGKIPGGRCSSADPYFGYEGAFKCLLEKGDV 244

YFNNSCAVYSL +K+NPIGDNSDKLC LCTGKIPGGRCS+ DPY+GY+GAF+CLLE G+V

Sbjct 3301 YFNNSCAVYSLINKYNPIGDNSDKLCRLCTGKIPGGRCSARDPYYGYDGAFRCLLEAGEV 3122

Query 245 AFLRHSTVNEMLQTTEFKNIAPDTFELLCRDGRRASINDYRQCNWGQVPADAIVTSSARS 304

AFLR TV EMLQT EFK+++PD FELLCRDGRR + +YR CNWG +P+DAIVTSSAR+

Sbjct 3121 AFLRDLTVTEMLQTDEFKSLSPDRFELLCRDGRRMPVTEYRHCNWGIIPSDAIVTSSARN 2942

Query 305 FSDRKQYQQFLKRIAELYSDGTRDDQSRQG-------GQSFNSRNNINDQNAYGQ---FD 354

+RK++QQFL+RI ELYSD RD+ +Q G + + NN N+++ YG +

Sbjct 2941 SLERKKFQQFLRRIIELYSDALRDEAEQQNLRGNTEYGNNNFNYNNNNNKDQYGNPYGVN 2762

Query 355 NNDPYRTQNQYDQ---------YRSERLDSSFAEERNQQDGTNTSILYEKFRIFESKRYG 405

N+PY T N Y++ +R++RLDSSF ERN +GTN SILYEKFRIFES+RYG

Sbjct 2761 INNPYDTSNTYNRNSYDGGNSGFRNDRLDSSFTTERNYGEGTNESILYEKFRIFESRRYG 2582

Query 406 KPNLLFQDSSRALTVIPEDDQSFTKYLGPAINFIYGIRECPVPAMTLCVTSENELDKCIK 465

K NL+FQDS+R+L+ I EDDQSF+KYL + +IYGIRECPVP+MTLCVTS+ EL+KCIK

Sbjct 2581 KANLMFQDSARSLSPIAEDDQSFSKYLQNTMQYIYGIRECPVPSMTLCVTSDPELEKCIK 2402

Query 466 MRTALKAHLLKPELICKKMHSHINCMQFIEAGKADISVFDAGDVYTGGLNYDLVPFMSEV 525

M+TALKA LLKPELICKKMHSHINCMQ+I++GKADI+VFDAGDVYTGGLNY+LVPFMSEV

Sbjct 2401 MKTALKAQLLKPELICKKMHSHINCMQWIQSGKADIAVFDAGDVYTGGLNYELVPFMSEV 2222

Query 526 YNLGEPEYYVVAVAKEDDPDTELTYLKGKNTCHTGINTAAGWTYPMALFISNGWIRPYGC 585

YNL EPEYYVVAVAKE+DPDTELTYLKGK TCHTGINTAAGWTYPMA ISNGWIRPYGC

Sbjct 2221 YNLDEPEYYVVAVAKEEDPDTELTYLKGKYTCHTGINTAAGWTYPMAFLISNGWIRPYGC 2042

Query 586 DSVRAAAEYFTKSCVPGAISNEYNTGVPYDSMCDLCHGTSYRYCRRDASEEYYGHTGAFR 645

DSVRAAAEYFTKSCVPGAISNEYNTGVPYDSMCDLCHGTSYRYCRRDASE+YYGHTGAFR

Sbjct 2041 DSVRAAAEYFTKSCVPGAISNEYNTGVPYDSMCDLCHGTSYRYCRRDASEDYYGHTGAFR 1862

Query 646 CLVEGGGHVAFMKHTTVMESTGGKRKEWWARNALNDDFELLCTDGTRAEIQDYKRCNLGK 705

CLVEGGGHVAFMKHTTVMESTGGKRKEWWARNALNDDFELLCTDGTRAE+QDY++CNLGK

Sbjct 1861 CLVEGGGHVAFMKHTTVMESTGGKRKEWWARNALNDDFELLCTDGTRAELQDYRKCNLGK 1682

Query 706 VKANAVVTRGGVNYNETQMNAYINLLTYAQQLYGRKEVDAFSFSMFSSPIGHYDLIFQDA 765

VKANA+VTRGGVNYN+TQ+NAYINLLTYA QLYGRK+VD+F+FSMFSSP+G YDLIFQDA

Sbjct 1681 VKANAIVTRGGVNYNDTQVNAYINLLTYALQLYGRKDVDSFTFSMFSSPMGFYDLIFQDA 1502

Query 766 TRQLQVIPPNKRRYDAYLGSDFMRARRITDCY 797

TRQL+VIPP +RRYD YLGS+FMRARRITDCY

Sbjct 1501 TRQLRVIPPEQRRYDIYLGSNFMRARRITDCY 1406

**Conserved domains**

**PBP2_transferrin domain-containing protein**


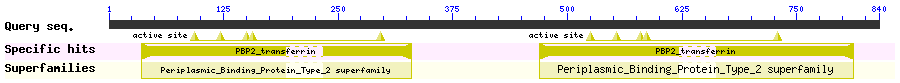


**Vha16**

**>TRINITY_DN29956_c2_g1_i7 len=4212** TTATTCATTCTCTCAAGTTGTTGGAAATTAGAAATTACACTTTATTCATTCTCTCAAGTT

GTTGTTTGTTTGTTGGTTGATTTGTGATGGTAATTTGTTTATGTTTATCGATCGTCGTAA

TACAAGTACAATACAAAAAGTCGTTTTTTTTCTCAAAATAATAAAAATAATCTGCCGCTC

TGCTGCTACCGCTACTGCCGCCTTCCACTTCTTTTTTCGCTCTGCTGCGACTGCCATTGT

TAGCAATTCTTCTAATTCGCAGATCTCCAACAAAGCAATACAACGAACGAAAAGCCAGAC

GCGCTAATATTAATATGACTTAAACTAACTAATAACGAAATGTAATGACAATATTTCTAT

ACGAAAAATGTATATGTGTATGTAATTTGTGCAACAGATGTTTTGTTGTTACTATACAAA

TACAATTTGTTATTGTTTATGTGAATATTAGTACGCTAATTCTTGTTGTTGTTTGAAGAA

GCCCTACAGTGCAGTAGCCGCCCGCACGCACGCATGGGAGAAGCCACTATGCCAAAGACA

GCACAGTCACACATCGCTGGTATACTCTTGACATCTGTCAGCGCTCTGTGGAACACTTCA

TACGTTTGCGCTTTGCACATACACCACCACCGCCATACAAGCAATGGCTGCTCACAGGTT

GCACACCTCCACGCTCTCAGCATCACAAACGTATCGGCATGCAAGGGGAGCGCCACTGGC

AGCGTTGTGGTTGGCAAGTGTATATGTTGGATGCGCACGCGTCTGCACGCCACTGCAACT

GGCCAGCAAATGCTCAAGGTACACCACTGTTGTGTGCTGGTGGCTTTTACATAGTTACTC

GCTTTTATTACTTACAAATCAGATTGCTTAGTACTGTATACGAAATTTCTAATTTTTATG

TTGGGGATTTTGCTATTTTTTTGGTTGATTTTTTCATATTTTTTTAGATTTTTGTTCCTT

CTTCAATTCATTTTTTCTACTACGTCTTCGACTTTCAACGCTATAGCGCCTACTTTTCGA

CTTCGACTACTGCTTATTCAACATTTTATATTCTTCAGCGAAATTTCACTAAATACGACA

CATGGTTGTTATTTTCATATATGTAATAATCATATTTTACATAATATTTTTGTTTGCATT

TTTAATTTCATATATATATAATTATTTGCATTTCTATACATAACAGAGGAAATATGTATA

TGTGGGTGTGTAGTGTATTGCACAAAGAACTGTTTTTTACCAATTTTTGAAAAATATAAT

TATTCGTTCTCCAGCAACAGGGGAAATTGTCTTTGTGCAATTGGCATCAATAGGTGAGTG

AATGAGAAGTGATTTAAGAGTTTGTGTGCAAGTATGCTACTGCGAACACAGTAATTCCGC

TATTTTCAACGCGCACACATACTTGGTTCTTATTGTATCAACTTCAGCCGCAACTTCAAC

TCCAACTGCTGCTATTTTTTCTAGGTTTGCTTAAACGCTACTCCACATACACTTTTTCAT

TTTAATTTTCGCTTTTTTTGCAGTTCTATAAATTACTTGTTTTTGTAACAACTTCAGCGC

CTTTGCACCTCCATTTTGTTTAAATATGTATAGTATGTATAATTATTATTAATAATTATA

TATATATATATTGTATTATGTATGTATGTATATTAAGTATTTATAGATATAATAATACAT

TTATAAATACCATTAATTTAGTTTAGTTTTTACGTTTTCGCTTCAAAATTTCAAATGTTT

CATAATCAACTTCCATTTAAGCTTATCACAAAAAAAAGAAATATAATATAATTATTATTA

ATAATAGTTCTTACAAATTGAACAATAATTAAAAATAAAAACACATATATAGGGACTTAG

TTACAGTGTGTGTGTGTGTTGGCGTGTGTTTTGATGTTTTTGGGTATATCGATGTTGTTT

CAATTATTCCTTTATTTTGCATAGATGTGTGTGTTTCCTTTTTAGATGGCTGTTGGTTGT

AAAAGTTCGTCCTTTTGTTATTTTTATAGTTTTATAGTTATTTTGCGTTAATTTCGACGA

GTAGTTGTTCGTTCTCAACAATGTGGTCTTCCCTTCAATCAATGCAACAAAAAGATATCA

TGCGAACCGCATATGAAGTGGAGTGTAGAGTATATTCATAAGAAAAATTTTACCGCCTAA

AAGTAGGCTTGAAGTGGTGGTTACAAAATTTTGGGCCGCATATAGTTGCTGCTAAACAAC

CTTCTCTTCGATGTGTGTGTGTGTGTTGTTTATAATGCGATGTACGCAAGCGCCCCGTTG

AGAAGTGTGTGAATATACCAATGGAAAATCAATAAATTTTACGGTTCAATGTACTTTTTG

TCATGATCACGCCCCACTTTTTCCAGCATCCAAATTGCAGTCGCCTGTTTTCAGTTAAGC

GTTCTCATTTTGTTGTTTATGGTGGTGGTGGTTTTATTCATTTAATGGTTGTCTTTGTTG

TTGCTTTGTTGGTGGTTTCTCACTAATTATTTAATATTTTTAACTATATACTGTTTCTTT

GTTTGCTTTATTTAAGTTTAATTAATTTTAATATTAATTTTAAATTTAAATTTACATGTA

CATTTTATTTAATAAGTTTGTTGGTTGGTTGATTCCATTCCCATTTGCTTTTTAAACTTT

TACTGCTTTTGGTTGTAGTGTTTTTGCTTTCTTTCTACTTTTAATACGCAAGTTTTTCTT

TAAATAACAACAATAAACGTTGTTGAAAATATTTATAATTATAATTACACAAAAAACAAA

AAAAGAAATATCAGAAAAAACATTAACAAAAAAGAAAATCGTTGTTAATATAAACGCAAA

AAACGCGAAAAGAGAAAGTTTTTTCAAATCTTACAGCAGAAAACGTTTTTCCCCTTTGTT

TTTTTTTAACAAAATACGGAAAATTAACAAAAAAAAAACAATTGGCAGCGTCGATTGTTG

CGATCTAACACGCTTTGAGGTAACCTACTAGCGTTGTTATTTGTAACGATTTATGTGTTG

AAATATTAATAACATTTACGTGGTTACTTTGCTTCCTTGCTTTCATCGCGGCGAAAAAAA

TAGACCCAATCTTTCTTTTTAGTTTAACACTTGGTTACTTTATGTATATACAAATATAAA

TTGTTGAGTTTTTGTTGTTGTCGATAATGGTCTTAGCTCCCCGAAAATAAATTAAAATCA

CAAAGTAACGTAACATAAATAGAATCAAGCTGGGTAATGGTAATATAGACATACATAGAG

ACGAAACAGTTTTTTGGCTCGACGCATATCCCGCTGCATAATATCGAATGTGCATTTTGT

AATTCAATATTTGTGCAACGATTCATCTCTTCCCTTATTCGATGTTATGAATATTTCAAT

ACTAAACCAGCCATTCATTTGTAGAATTTTTTCTTTGGCTCTGTGCAACCAATTCTTTCA

GCTTTGGGAGATCAGCTACTGCTGTTTTCATCTTTGCTTGACAGCTGCTTTTTCTGTAAA

TCTTGTTGGTGATCTTTCTCGCTTCCACTTTGTTTTTGGTTGTTGCTGCGCTTATATTAT

TACAAATTTCTTTGATGATGTTGCTTGTTGCATTTTAATGATTGTTTGGTTGGTTGATTT

GTTTGCATTTTGTTGCTTTATTTGATGTGGATGTTGGATTTGTTTTTGTTGTAATTGTTG

AATTGATTTATTTTGTGTACAAGTAAATGGCAACAATAAGACCGTACAGACCCAATACTT

CAGCGAAAATAAGAATCAAAATCATACCGACGAACAGACGCGGCTGTTGTGCTGTACCTC

TTACACCAGCATCACCAACAATACCGATCGCAAAACCGGCCGCTAAACCAGAGAAACCTA

CCGATAGACCGGCACCCAAATGAATGAAACCCTTGAACAGTGAGTAATCTGAAGGTTGTT

CCAAAGCACCAGCAATAAGGACAGCTACGACCAAACCGTAAATGGCAATAATACCAGCCA

TGACAACGGGAATGATGGATTTCATGATCAATTCTGGTCGCATCACTGACATGGCAGCGA

TACCAGTTCCTGATTTGGCGGTACCATAGGCGGCTCCCAGAGCACTGAAAATAATGGCTG

AGGCTGCTCCCATAACTCCGAAGAAGGGTCCGTAGATGGGGTTATCGCTCTGTGAATCAG

ACATGATTTCTGTTTTGTGGTGTATGTGCGGGGTGTTTTATAAGTTTTTCTTCTGTCGTC

GGCGTGTTCGTG

**tBLASTn(First hit)**

Score = 284 bits (727), Expect = 2e-088, Method: Compositional matrix adjust.

Identities = 149/157 (95%), Positives = 154/157 (98%), Gaps = 0/157 (0%)

Frame = -3

Query 3 SEVSSDNPIYGPFFGVMGAASAIIFSALGAAYGTAKSGTGIAAMSVMRPELIMKSIIPVV 62

S+ SDNPIYGPFFGVMGAASAIIFSALGAAYGTAKSGTGIAAMSVMRPELIMKSIIPVV

Sbjct 4141 SDSQSDNPIYGPFFGVMGAASAIIFSALGAAYGTAKSGTGIAAMSVMRPELIMKSIIPVV 3962

Query 63 MAGIIAIYGLVVAVLIAGALEEPSKYSLYRGFIHLGAGLAVGFSGLAAGFAIGIVGDAGV 122

MAGIIAIYGLVVAVLIAGALE+PS YSL++GFIHLGAGL+VGFSGLAAGFAIGIVGDAGV

Sbjct 3961 MAGIIAIYGLVVAVLIAGALEQPSDYSLFKGFIHLGAGLSVGFSGLAAGFAIGIVGDAGV 3782

Query 123 RGTAQQPRLFVGMILILIFAEVLGLYGLIVAIYLYTK 159

RGTAQQPRLFVGMILILIFAEVLGLYGLIVAIYLYTK

Sbjct 3781 RGTAQQPRLFVGMILILIFAEVLGLYGLIVAIYLYTK 3671

**Conserved domains**

**V_ATP_synt_C and ATP-synt_C domain-containing protein**


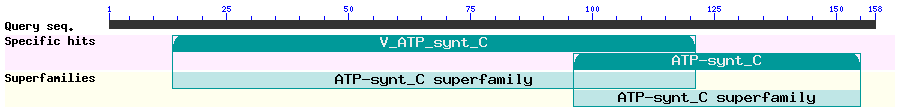


**VhaSFD**

**>TRINITY_DN26174_c1_g1_i6 len=1596**

CTTCATTTGTCTATATTTATTATATAATACATTCATTAAAATATTCATTGAATTTCTTCG

ATTAACTATATTTGCATAATATTTCAGCAGCCCATAGCCACGAAAATTAACACAAAAGTA

GAATTTTGTACACATCCCAAAAATACTGACTCAATTTTTTTTAAATGATGCACAAACTTT

GAGGAATAATAATAAAAACAAACATAGCGATCAAAATATTTCAACAACAACAATAATAAT

TTTTAAATAATAGTACGCAAAATAAAATGGAATGATGAAACAATTAAATATAAAATACAA

TTTTTTGGATTGAACTGCCAGAATTTTCATGTACACTTCTGCTGCTGCTGTTATACTTTA

GTTTTTTAAGATGGAGTTATATTTATTCAGAGTAACTATAAATGATTATGGTATTTATTA

TTTTAGGAGAATTTTAGTACAATCAACATTTATTTCATCGACACCTATTCGGCGTACATT

ATAGCCGATTGTTTTTGACAAGCGAACGCATATTTTTTAAGTTACTTAGGCTTTACCACT

GATCGGCGCTGAACCTCCTTTTTGAGGTTGATTTTCACTTTCCTTTTCCAGGTGTTTGCC

CAAGTATTCCCAATTGTGTACCATCAATTTTTGTACCGCTAAGAGAGCCTCATAACGCAC

ATTTGGATCGTCGTGAGCCAGAAGCTGCATTACAATTTGTTTACCACCCAACTGCTCCAA

AACGTGTTTGCCTCTTGGATAATGACGCACATATTCCCCAATGTCAAAACAAGCCACTGA

GAGTATAATGTGATCTTTTGAGGTCTCCAACAAATGTACAAGAATACGCAATAGTTCGTA

ATTTTTCTCGTTCAAACGATGAGCATTTTCACGCCAGAATTTAGCCGACTTATGCACTGG

TGACCATTCAAGGCGAGCGCTTCGTAACTCAGTCGCATATTCGTCGAAGGAACTCAAATC

TTGTACAGAGTTCTGCAGTTTCTCGGTGAGGAATTCAACATCGGCGGAGATATCCTCATC

ATCGAAGCGACGTTGCTCCAAAATGGACAACTGTTTCAGAACTTTGCACTGAACCATTGC

AATACAATGCTCCTTGGCTACTTGTGGATCAGTAGGTTTCTCGATTAGATTACGAAAAAC

GGCCAAAATTATGCGAGTGACCTTTTCCTTGGCGCAATCATTCAGGATATCTGCCAAAAT

TGGTATAACACTGAACTTATTCATTTTGGTGGCCAAAAGTGGATTGAAAGTAAGCACCCA

TAAACAAAACACTAATTGGTATTGCACCTGGAAATTAACACGAGACGAAAGAATACTAAT

CAGTGTGCTAATGCCGTCCACCGACACAAAAGCAAAACGATATTCGTCAATACGAAGCAT

CATTTGAAGACAGCGGGCGACCGATTGAATATACTCATTGGCATTCACGGTCAGCTGATC

CTTCAAGAACTGCAAGTAGAAGTTCAAATCCGACTTTGGCATGAGTTCATGACCCCAGCA

AGCAAGTTTTGCTAGAATACGCGATGACATATTTACAATGAAACCATCTTGACGATTTAA

TAAGTTAAGGAAAGGCCCCCAAACACATTCTTTGTT

**tBLASTn(First hit)**

Score = 675 bits (1741), Expect = 0.0, Method: Compositional matrix adjust.

Identities = 320/355 (90%), Positives = 335/355 (94%), Gaps = 2/355 (1%)

Frame = -1

Query 116 KQCIWGPFLNLLNRQDGFIVNMSSRILAKFACWGHETMPKSDLNFYLQFLKDQLASNNNE 175

K+C+WGPFLNLLNRQDGFIVNMSSRILAK ACWGHE MPKSDLNFYLQFLKDQL N NE

Sbjct 1593 KECVWGPFLNLLNRQDGFIVNMSSRILAKLACWGHELMPKSDLNFYLQFLKDQLTVNANE 1414

Query 176 YIQSVARCLQMMLRVDEYRFAFVGVDGISTLIRILSTRVNFQVQYQLIFCLWVLTFNPLL 235

YIQSVARCLQMMLR+DEYRFAFV VDGISTLI ILS+RVNFQVQYQL+FCLWVLTFNPLL

Sbjct 1413 YIQSVARCLQMMLRIDEYRFAFVSVDGISTLISILSSRVNFQVQYQLVFCLWVLTFNPLL 1234

Query 236 AAKMNKFSVIPILADILSDCAKEKVTRIILAVFRNLIEKPEDSSVAKDHCIAMVQCKVLK 295

A KMNKFSVIPILADIL+DCAKEKVTRIILAVFRNLIEKP D VAK+HCIAMVQCKVLK

Sbjct 1233 ATKMNKFSVIPILADILNDCAKEKVTRIILAVFRNLIEKPTDPQVAKEHCIAMVQCKVLK 1054

Query 296 QLSILEQRRFDDEDITADVEYLSEKLQNSVQDLSSFDEYATEVRSGRLEWSPVHKSAKFW 355

QLSILEQRRFDDEDI+ADVE+L+EKLQNSVQDLSSFDEYATE+RS RLEWSPVHKSAKFW

Sbjct 1053 QLSILEQRRFDDEDISADVEFLTEKLQNSVQDLSSFDEYATELRSARLEWSPVHKSAKFW 874

Query 356 RENAQRLNEKNYELLRILVHLLETSKDAIILSVACFDIGEYVRHYPRGKHVLEQLGGKQI 415

RENA RLNEKNYELLRILVHLLETSKD IILSVACFDIGEYVRHYPRGKHVLEQLGGKQI

Sbjct 873 RENAHRLNEKNYELLRILVHLLETSKDHIILSVACFDIGEYVRHYPRGKHVLEQLGGKQI 694

Query 416 VMQHLGHEDPNVRYEALLAVQKLMVHNWEYLGKQLEKENENQKQ--GAAPIAGKA 468

VMQ L H+DPNVRYEALLAVQKLMVHNWEYLGK LEKE+ENQ Q G+API+GKA

Sbjct 693 VMQLLAHDDPNVRYEALLAVQKLMVHNWEYLGKHLEKESENQPQKGGSAPISGKA 529

**Conserved domains**


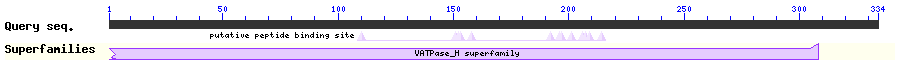


**Small Rab GTPases (Rab7)**

**>TRINITY_DN30000_c1_g3_i9 len=1870**

GACTATTTTTTTTATTCTACAAAATGTCAACAATACAGAAATAAATTTCAGTCGGTATTA

AATGAGTTGTTAAAAATTTTTTTCGACACCGGACTATAACATGTCTCCATACATGTATGG

GGAAAATTTATGCTACAACAACAACAACGCAATTTAAATGTAATCATATACAGTTTGAGA

TTTATTTATGTACTTTATTTCAAAGATACTTTAAATTTAGCTTACTTGTGTTTAATAAAA

ACTGAAATGTACATACATAACTACATAGAAAGAAAAAAATATATGTGTTGAATTGCGTAT

AAACATTGCAAAATTACATTAACCAATATAATACTCAGTAAATCATAAATGTAAACTGAA

GAAATACACACGCCGCAAGCAAGAGTGTTATAAATTTGCTCCCACCCTACCAACCAGCAT

ATCTGTTCATGTAATTTACATATCATTTTATTCTCTACACCGCAGCAGTATTCCAGCGCA

CAAACATATACACTTTTAGCTCATACTGTATAAACTTATTACTACTATATGCAGTGGTGT

GTTGAAAATCACTTTAAAGAGATTTTGTTAAACTAAATATTACTATTTGCGTTTCACACA

CAATTATTTCTCTTAAAAAATGAGTAATAAGTTGCAATTTACAATGGGCACACAAAATCA

AGTGTTTACAATGTTAAAAAAAAGGAAGTTAAATATGTGACAAGGCCAGAAATAATGTGT

ATTTACATTTACACATTTCAGTTTTGTTTGGCGAAATTTTTACGCTTACTTTTGCTGTTT

AATTATGTTGTTTGATCGGCTTAAAAAACTGCTTTTACATTTTTTCATTTCTCATTTTTC

ATTTATTTCGTTTGGTAAATTATTTTACTTAATGTAACCTCCTTTTGCATTATGTATCGT

TTTATCATCGTTTATCATATATGTTTAATTTTGTATAGTATTATTTTAATAATAATATTT

ATATTTAAATTATGTTCAGTTGCGAAACTATGCAAATATATTATTTAGGTATTTTCGTTT

GTTTTTTTGTTGTTGTGTTTTTTTCACTACTATTCCAATTGCATCTGCATCTATTTGATA

TTGCCAGTTTTTGCTATAGTCGCTAACGCGTTTTGAATATCTCTATATATTTCTCATTTG

CGGTAATTTCACAACTACATGTCGACGCCGCAAAGGCTAATATATTTAATAGCGGTAATT

ACATTAGCACTGGCAGTTATCAGCATTACCATTGCGATTGTTTCGGTCGGTATTAAGGCG

GATTTGATCAGGAAATTCATTATATAGTTCAACCTCGGCTTCCTGAGCAAGCGCATTCTT

TGCAATGGTCTGGAATGCCAACTCCACATTAGTGCCGTCCTTTGCTGATGTTTCAAAGTA

TGGCAAATCGTTTTTAGATTGGCACCACTGTTGGGCACGTCGCGTGGACACCTGTCGGTT

ATCCATGTCAACCTTATTGCCCAGCACTACGAATGGAAAGTGATCGGGATCTCTGGGACT

GGCCTGTATCAAAAATTCATCGCGCCATGAATCGAGATTCTTGAATGAATTTGGTGATGT

GACATCGTAGACGAGCACACAACAGTCAGCGCCACGATAAAAAGCCACACCAAGCGATTG

AAAACGTTCTTGTCCAGCAGTATCCCAAATTTGCATTGTGACTACTCGGTCGTCAGCAAT

GATTTCTTTTGTACAGAAATCAGCACCGATCGTTGCCTTGTATTGATTTGAGAAACGTTT

ATTTATATATTGATTCATGAGTGACGTCTTGCCGACGCTACTATCACCGAGAATGATCAC

TTTCAGCAAAGACTTCTTGCGAGATGCCATAGCTGCTTATTTCGTTGTGATTCCTATAGA

GCTTTGCTTT

**tBLASTn(First hit)**

Score = 371 bits (952), Expect = 9e-125, Method: Compositional matrix adjust.

Identities = 180/208 (87%), Positives = 195/208 (94%), Gaps = 1/208 (0%)

Frame = -2

Query 1 MSGRKKSLLKVIILGDSSVGKTSLMNQYVNKRFSNQYKATIGADFCTKEVVVNDRVVTMQ 60

M+ RKKSLLKVIILGDSSVGKTSLMNQY+NKRFSNQYKATIGADFCTKE++ +DRVVTMQ

Sbjct 1830 MASRKKSLLKVIILGDSSVGKTSLMNQYINKRFSNQYKATIGADFCTKEIIADDRVVTMQ 1651

Query 61 IWDTAGQERFQSLGVAFYRGADCCVLVYDVTAPNSFKNLDSWRDEFLIQASPRDPDHFPF 120

IWDTAGQERFQSLGVAFYRGADCCVLVYDVT+PNSFKNLDSWRDEFLIQASPRDPDHFPF

Sbjct 1650 IWDTAGQERFQSLGVAFYRGADCCVLVYDVTSPNSFKNLDSWRDEFLIQASPRDPDHFPF 1471

Query 121 VVLGNKVDLDNRQVSTRRAQQWCQSKNDIPYYETSAKEGINVEMAFQVIAKNaleleaea 180

VVLGNKVD+DNRQVSTRRAQQWCQSKND+PY+ETSAK+G NVE+AFQ IAKNAL EAE

Sbjct 1470 VVLGNKVDMDNRQVSTRRAQQWCQSKNDLPYFETSAKDGTNVELAFQTIAKNALAQEAEV 1291

Query 181 eVINDFPDQITLGS-QNNRPGNPDNCQC 207

E+ N+FPDQI L + +NNR GN DNCQC

Sbjct 1290 ELYNEFPDQIRLNTDRNNRNGNADNCQC 1207

**Conserved domains**

**Rab7 domain-containing protein**


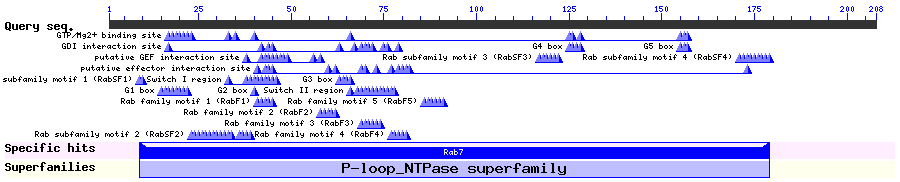


**Ligth**

**>TRINITY_DN31345_c1_g2_i1 len=2901**

CAGCTGACCGAGCAATATCTTGTAAATTTTGTTTTGTTCTAGCAGACAAGTGCAGTTTTA

CATTTAGTATTAAATAGAAAATGAAATAGAGTAAGGACTCTTAAAATAAATAAAAATAAT

ATACATTTAAAATATTGATCTCCGCATCTTCACCATGGCTGAGCAAGAGCATGTTAACAA

CAGCGATACAGAAACTGAAAGTACGGAGGATGAAATTGAACCAAAATTTAAATATCAACG

CATTGCTAACGATCTGCGGAAGATTCTCAACTCAGATGTTGTGACTTGCAGTGCTGTTCA

TCCAAAGTTTTTAATGTTTGGCACATTCTTGGGTCGTGTCTACATATTCGACCATCAAGG

CAATTCGGTGACTTCACATCTTTCAGATGGACCAAACGATTTTTCACACACTGTCGCTGT

CAATCATATAGATGTGGACTCGAAGGCAGAATATGTGGCCACCTGCTCTGACGACGGCAA

GGTAAATATAACCGGCCTATTCAGTGATGAAAATAATCAGAACTTAAACTTGGGGAAATC

GATAAAGGCTGTTGCGCTAGATCCCGATCCAAAGACAAGTGCCGGTAAACGATTTGTTGT

AGGCGACGATAAGCTCACGCTATACGAAAAAAATTTTCTAAAAAAGCTAAAAACAACAGT

GCTATCTAGCGCCGAGGGATATGTGCTATCAGTATGCTGGAATGGTCCCTTTGTGGCATG

GGCAAGTTACCTAGGGGTACGTGTCTATGATCTAAATGAAAAGTGTTCACTAGGTCTTAT

GAAGTGGGAAGAGCCAGTCAATGCGCGTTTAGAAAATTTCCGATGTAATTTTCGTTGGTC

AAATGCCACGACGTTGCTCATAGGTTGGGTGGATACAATACGAATCTGTGTGATACGTAG

ACGAAATTCCATTGAAGTAGCTTCCCGAGAGCTACCAGGATATATAGTTGATCCCATATC

CACATTTCAAACTACCTTTTACGTTTCCGGCCTGGCGCCACTCACATCCAATCAATTGGT

CGTATTGGGTTGTCCAAAGGAGAAAGATGCAGAACGTAAGTCATTAAGACCTGTTCTCTG

CGTTATGGAATATAAGTTGAATACCAGCGAAGAGATTTGCACAGACAGTCTGTCATTGCG

TGGATATCAGGAATATACCGTCAATGATTACAGTTTGGGCTGCATTATTGAGGAAAATCG

TTACTTTATTGTTGCACCAAAGGATATAGTTGTCGCTAGTCTATACGAAACGGATGATAG

AGTCAAATGGCTGGTCGATCATCGCAAATTTGAAGAAGCCATGGAAGTTATATCAACACA

CGGCGGAAGTTGGTCGTTACTGTCTGTGGCCAAATTGTATATAAATCACCTGCTCGCTAT

GAAGCAATACGATGACGCTGCTAAATTATGCCTACGCGTTTTAGGGAATAAAAAATCACT

TTGGGAGGAGGAAGTTTTCAAATTTGTCAAGTGCCAGCAATTGCGCTCGGTTAGCGCTTA

TCTACCTACCTCTGACGACTGTAAACTTGATCCTCACGTATACGAAATAGTGCTGTATGA

GTATTTGAAATTCGACGCCAAAGGATTTCTAAATCTCATCAAGGAATGGCCATCCCATTT

GTACAATTGCAAGGCGGTCATCAATGCAATACACGATAATTTTCGCAAACAGAATGCGAA

TGAATTATTGGAGGCGCTGGCAATACTCTATTTGCATCAGCGCGACTATGAAAGTGCATT

GAGAATGTACTTAAAATTGCAAAACTCAGATGTATTCGAAATGATACGTCGTTATAATCT

GTATGATGCGATACATAAAATGATTATACCACTCATACAGCTGGATCGTGAGCGTGCCTT

TAAGATTTTATTCGAAAAAAATAAAATTCCACCAGAAATTGTTGTACAGCAATTAGAGCA

AAATCAAGAGTATTTATATTGGTATTTGGATGCTTTGGACAAGGTGAATAAAAGCGGTAA

ATATCATTGGAAATTAGTGAATTTATATGCGAAATATGAGCCTGAAAAATTGCTACCATT

TTTGAAACGATCCAATCACTATCCAATGCAAGAGGCATTGGATATATGTAAACGCGAACT

ATTTTATCCCGAAATGGTTTATTTGTTGGGACAGATGGGCAATACTATAGAGGCATTAAA

CATAATTATTGAAAAGATAAAAGACATTGAAATGGCTATTGAGTTCTGCAAAGAACGTAA

TGATTCGGATTTGTGGAATATATTGATCGACGAGTCAGTCAAGGAGCCCGCCATTGTGCT

TAAACTGTTAGACGGCATAGTAGATTATGTCGATCCTGTAGCAGTTGTTGAAAAAATCAA

ACTTGGCCAGAAAATTCCAGGCTTACGCGATGCTGTCGTCAAACTTTTGTGGGACTATCG

CTTACAGGTGGAAATTCGCAAAAGCGCGCAAAAGCTTCAACTAGAATTCTACTACGATCA

GCATGCCAGAGTGGTAAACACACAAAATCGAGGTCGGTATATTTCCTCTTCCGAGCGGTG

CTTGAAGTGCAATCGCTCTGTACTCAGTATGAACGAAAACATTGCTCCTCTTAATGATAT

TGTCGTATTCTTGTGTGGTCATGTTTATCATACTAACTGTGTACCGGGTGGCATCGGCAA

TGAACACTGCGAATTTTGCAATCCTAGCGATTTCAATCAAGAAGACGACTTCGCACTACT

TTTGCAATATAGTAGAAAGTAATAATTTTCAATAACTCGATGACTTTGAGCTGTTGTCTT

GTAAGCGACTTTATCTTCATGCATACGAGGCCTGTTAGAAAAGTATCCGACTTTGTTTTT

TATTTCTAACACCTGACAAATTTGAATTACGCAATTACGATCTGCAACCTCCTGCACATG

TGTGAATTTTTTCCCGTCTGT

**tBLASTn(First hit)**

Score = 1113 bits (2879), Expect = 0.0, Method: Compositional matrix adjust.

Identities = 566/839 (67%), Positives = 673/839 (80%), Gaps = 9/839 (1%)

Frame = +2

Query 1 MAKALPLISCDSWADSINEEDVEPKFKYHRLANDLKYMLNADVITCSAVHLKFLIFGTFR 60

MA+ + + D+ +S E+++EPKFKY R+ANDL+ +LN+DV+TCSAVH KFL+FGTF

Sbjct 155 MAEQEHVNNSDTETES-TEDEIEPKFKYQRIANDLRKILNSDVVTCSAVHPKFLMFGTFL 331

Query 61 GRVCIFDHQGNSVYSNLS-ASERHTHQVAVNNIDVDHKGEYVATCSDDGKVNITGLFSSD 119

GRV IFDHQGNSV S+LS +H VAVN+IDVD K EYVATCSDDGKVNITGLFS +

Sbjct 332 GRVYIFDHQGNSVTSHLSDGPNDFSHTVAVNHIDVDSKAEYVATCSDDGKVNITGLFSDE 511

Query 120 NNHSLSFGKFIKVVSLEPDSKAHI-KRFVVGDDKLILYERNLLKKLKPVELCSVEGSVLS 178

NN +L+ GK IK V+L+PD K KRFVVGDDKL LYE+N LKKLK L S EG VLS

Sbjct 512 NNQNLNLGKSIKAVALDPDPKTSAGKRFVVGDDKLTLYEKNFLKKLKTTVLSSAEGYVLS 691

Query 179 ICWHGNFIAWASHIGVRVYDLNERCSLGLIKWEVPPQERLENFRCHLRWSNKHTLLIGWV 238

+CW+G F+AWAS++GVRVYDLNE+CSLGL+KWE P RLENFRC+ RWSN TLLIGWV

Sbjct 692 VCWNGPFVAWASYLGVRVYDLNEKCSLGLMKWEEPVNARLENFRCNFRWSNATTLLIGWV 871

Query 239 DTIRVCVIRKRNSIEASTGNLPVYIVDPISTFQTTFYVCGLAPLSAKQLVVLGFRKEKSS 298

DTIR+CVIR+RNSIE ++ LP YIVDPISTFQTTFYV GLAPL++ QLVVLG KEK +

Sbjct 872 DTIRICVIRRRNSIEVASRELPGYIVDPISTFQTTFYVSGLAPLTSNQLVVLGCPKEKDA 1051

Query 299 CFKALRPVLCVIEYKMNNSEEICTDSLTLRGFEEYTVNDYSLGGIIEENRFYIVAPKDIV 358

K+LRPVLCV+EYK+N SEEICTDSL+LRG++EYTVNDYSLG IIEENR++IVAPKDIV

Sbjct 1052 ERKSLRPVLCVMEYKLNTSEEICTDSLSLRGYQEYTVNDYSLGCIIEENRYFIVAPKDIV 1231

Query 359 VASLIETDDRIEWLIKHSKFEEAMELISANGGNVPVLSVAKLYINHLLALKKYDDAAKLC 418

VASL ETDDR++WL+ H KFEEAME+IS +GG+ +LSVAKLYINHLLA+K+YDDAAKLC

Sbjct 1232 VASLYETDDRVKWLVDHRKFEEAMEVISTHGGSWSLLSVAKLYINHLLAMKQYDDAAKLC 1411

Query 419 LRMLGNDKVLWEEEVFKFVKCQQLRSVSAYLPTSDECKLDPHVYEMVLYEFLKFDVCGFL 478

LR+LGN K LWEEEVFKFVKCQQLRSVSAYLPTSD+CKLDPHVYE+VLYE+LKFD GFL

Sbjct 1412 LRVLGNKKSLWEEEVFKFVKCQQLRSVSAYLPTSDDCKLDPHVYEIVLYEYLKFDAKGFL 1591

Query 479 NLIKEWPSHLYDGLAVINAIHDNFRKHYANQLLESLALLYSYQGDFESALRMYLKLQNKD 538

NLIKEWPSHLY+ AVINAIHDNFRK AN+LLE+LA+LY +Q D+ESALRMYLKLQN D

Sbjct 1592 NLIKEWPSHLYNCKAVINAIHDNFRKQNANELLEALAILYLHQRDYESALRMYLKLQNSD 1771

Query 539 VFQLIRRYELYDVISKLIIPLIQLDRDCAFEILLDKKKIKTEIVVHQLEHNQEYLYWYLD 598

VF++IRRY LYD I K+IIPLIQLDR+ AF+IL +K KI EIVV QLE NQEYLYWYLD

Sbjct 1772 VFEMIRRYNLYDAIHKMIIPLIQLDRERAFKILFEKNKIPPEIVVQQLEQNQEYLYWYLD 1951

Query 599 SLLKKDPSNVFQKKLISLYAIFDRNKLLPFLKRSKDYDIQEALVICKQENFYPEIVYLLG 658

+L K + S + KL++LYA ++ KLLPFLKRS Y +QEAL ICK+E FYPE+VYLLG

Sbjct 1952 ALDKVNKSGKYHWKLVNLYAKYEPEKLLPFLKRSNHYPMQEALDICKRELFYPEMVYLLG 2131

Query 659 CMGGVEASEALNIIIHRIRDIEMAIEFCKEHDDSDLWNALINEFSKHPEIVTKVLDGIVD 718

MG EALNIII +I+DIEMAIEFCKE +DSDLWN LI+E K P IV K+LDGIVD

Sbjct 2132 QMGN--TIEALNIIIEKIKDIEMAIEFCKERNDSDLWNILIDESVKEPAIVLKLLDGIVD 2305

Query 719 YFSPAVVVGKIKMGQNIPNLRQSLIKMLRHYNLQGEILSSAQQIQLNDYFEIHSEIVTTQ 778

Y P VV KIK+GQ IP LR +++K+L Y LQ EI SAQ++QL Y++ H+ +V TQ

Sbjct 2306 YVDPVAVVEKIKLGQKIPGLRDAVVKLLWDYRLQVEIRKSAQKLQLEFYYDQHARVVNTQ 2485

Query 779 RRGQQVSYEQLCSLCHRPVLMAGTH---LYCIIRLECGHVYHKPCIQGELL-KNCNECN 833

RG+ +S + C C+R VL + L I+ CGHVYH C+ G + ++C CN

Sbjct 2486 NRGRYISSSERCLKCNRSVLSMNENIAPLNDIVVFLCGHVYHTNCVPGGIGNEHCEFCN 2662

**Conserved domains**

**protein containing domains WD40, CLH, and RING_Ubox**


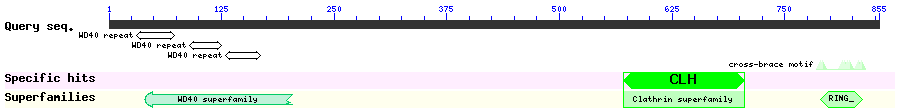


**Idlcp (Exocytocis)**

**>TRINITY_DN46925_c0_g1_i1 len=920**

GAGAAGACAAGCCAATCAAGAATAAATCAAAATTTTCAAACTGACGGGAAATATTTAAGT

TTATATTTTTTATATACAGATCAAAGATTATTTAATTTTTTATAAGTGCAATATTAAATA

ACCAACTAGCAAATATGGATGAAGTGGATGTAGCTACAGTGGATCAGTATCAGACACTGG

TGCGCTATAATAATCCAGTGTTAGTAGTGAAACACCCTGACAAAAAGGGTGTACCAACTG

AAATTGAATTAAAACGACCACTAACGGCTGGCGCTTTGTTGGACACCAAACGCGAAACTG

AGGAGATACTTAATTCCATATTGCCTCCGCGCTGCTGGGAGGAGGATGGGCAGTTATGGC

AACAAACGGTATCAAGTACACCAGCCACTCGACAGGATGTCATCAACTTACAAGAGATGC

TGGACACACGTCTTCAACAAACGCAGGCTCGTGAGACGGGCATTTGCCCCATACGCCGAG

AGTTGTATACACAGTGCTTTGATGAAATCATTCGTCAAGTCACCATTAATTGTTCAGAGC

GTGGCCTGCTACTGTTACGTATACGAGACGAAATCGCCATGTCTATGGAGGCATATGAAA

CACTATACTGCAGTTCGGTGGCATTTGGTATGCGTAAAGCATTGCAGGCACACGAAGAGA

AAGAAATGCTGCGCGATCGTGTCAAAACATTGGAAACCGAAAAGGAATCGCTCGAGGAGA

TTATCAACGACATGAAGATCAAGCAAGAGCAAGCAGAACGTCGTAATGCAGAACTGCGCG

CGTCAGAGGAGAAGAAATATGCGGAAGAGGTTGCATTCCTCAAGAAGACCAACACACAAT

TAAAGGCTCAACTGGAGGGCATTACAGCGCCAAAGAAGTGAGGTACTGCATTAACTTTTT

ATATTTGTGCAATATGGAAG

**tBLASTn(First hit)**

Score = 463 bits (1191), Expect = 1e-164, Method: Compositional matrix adjust.

Identities = 225/250 (90%), Positives = 239/250 (96%), Gaps = 2/250 (1%)

Frame = +3

Query 1 MEELDITSVGQFQTLVRYNNPVLVVKHPDKKGGAPLTEIEMKRPQTAGALLDTKRETEEI 60

M+E+D+ +V Q+QTLVRYNNPVLVVKHPDKKG P TEIE+KRP TAGALLDTKRETEEI

Sbjct 135 MDEVDVATVDQYQTLVRYNNPVLVVKHPDKKG-VP-TEIELKRPLTAGALLDTKRETEEI 308

Query 61 LNSILPPRCWEEDGQLWQQSVSSTPATRQDVINLQEMLDTRLQQTQARETGICPVRRELY 120

LNSILPPRCWEEDGQLWQQ+VSSTPATRQDVINLQEMLDTRLQQTQARETGICP+RRELY

Sbjct 309 LNSILPPRCWEEDGQLWQQTVSSTPATRQDVINLQEMLDTRLQQTQARETGICPIRRELY 488

Query 121 SQCFDEIIRQVTINCSERGLLLLRIRDEIAMSMEAYETLYCSSVAFGMRKALQAHEEKEM 180

+QCFDEIIRQVTINCSERGLLLLRIRDEIAMSMEAYETLYCSSVAFGMRKALQAHEEKEM

Sbjct 489 TQCFDEIIRQVTINCSERGLLLLRIRDEIAMSMEAYETLYCSSVAFGMRKALQAHEEKEM 668

Query 181 LRDRVKTLELDKEALEEIIADMKLKQEQAERRNAELRASEEKKFTEEITFLKKTNAQLKA 240

LRDRVKTLE +KE+LEEII DMK+KQEQAERRNAELRASEEKK+ EE+ FLKKTN QLKA

Sbjct 669 LRDRVKTLETEKESLEEIINDMKIKQEQAERRNAELRASEEKKYAEEVAFLKKTNTQLKA 848

Query 241 QLEGITAPKK 250

QLEGITAPKK

Sbjct 849 QLEGITAPKK 878

**Conserved domains**

**Ax_dynein_light domain-containing protein**


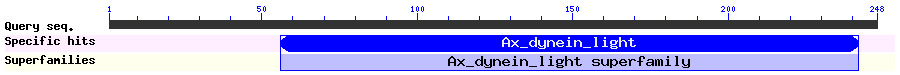


**FBX011**

**>TRINITY_DN32848_c4_g1_i12 len=6504** CATTGCTCCCGTAGAGGCGAAGGAAGTTGTGGGCCAAACACTTATTTTGTGCCAATTGAA

GTAAAAATAAAACATTTTTTTATAAAAAGTAGTTGATTAAGTGTCTAAGGGATTCTAATA

TATTCTATAGTGTGTTCCTTAAAGTATAATTTTTAGGGGAAGAAAAATTCTCTATGCACA

TAAAATTAACGTTCTCTGCTAACACTAATCTCAAAGGGAAATAAGCAATAAAACAAAAAA

AAAAATAAACGAGTCGTGATAGCTGTGAAGAGCTCAAGAGCGAGTGAAATGGGGGCAGAT

CAATTATCGAATAGAGTTTTGCTTCTTTGACAAGTGTTTGGCAAGGCAAAAGTGGAAAAT

TCAATACCGTCTTAAAACGAAAACAAAAGCGAAACGCAGTAATACATAGAAATATACAAT

AAATAGGAATAACAGCATAAAGAAGAAAAGGTGTAAGTGGAGTTAATGGCGACTGCAAAC

TCCAAGTGGGAAAGGAAAAAGGCGATGTCGATTACCGAAAATGGCGTTCTACTCGGGTTG

GTCTGTAACCTTTCCCTTTTGATCGGTTGCAAGGACTGGGCATAGAAAACGGCAGTGACT

TAAAAAGTTGTATATATATATAGTAAAGACTTTTGTAATGTTAATTCAAAATGGATATAA

ATTGTGTAGTGTAAAATCAGTAAAACATAGTGAGCTCTAACAAAGGACAATTAAAACAAA

GGAATTCATAATATCTGAATGCACTATTTTAAAATAATACTTAAAAGGATTGTACCTAGC

AATATTCTTAACAACATATAAGGAAAAATGCCCAGTGCTTCCTTCACTTCGTCGCGCTCG

TATGTGCGGCGTTCTAGACGTAAAGGTGCCAACCGCATTGCTTTACCCAATCGTCCATCC

GGTAACATAATGGATCCTATGATACAAAATATACCCGGTTCAAGTGCTTCTTCAAACGCT

AGTGCAGGTGCCAACTCCAATGCTATTGCACCGCCACTTCAAAATAATACTAATCCTCCA

AACTCGAGTGTCATCTCTACAACTATGAATGTGTCATCAATTCCATCTGGTTCACTTTCC

ACACCACAATTATCGTCGTGTTCTTCAGGTGACACATCATCGCAATCGGCTGCTATTGTT

AGTGCATCTTCAGCAGCTATGACAGCCTCAGCAGCTGCAGGTGCAGTTTCATCAGAAACG

TTTGCTAATGTTTCTGGTGCTTCCTGCTCGCAGTCATCAACTACTTCTGGACCTTCAACG

TCTAGGTCTGTTGCTGGTTCCTGCAACCTTGGAGTAGCAGGCGGACAAAAAATGCTTGGT

GCCACATGTTCCAGTAACTTAATAAGTTCGTCAGTTTTTAACACAGGAGGCGGTTTTGTA

GGAGCACATTTGATGGATACAGAAACAAGTAGCAATTGTGCAGGTTCCTTTAGTAGTTCA

GTGAATATTACTGCCGACACTTCCAGTAGTAAAAATAATACTGGCTCAAGCAATTCAATG

TATTCAAGTTCCGTGCCATGCAACTCATCAACTATGTCAAACGTATCTGCATCCACATCC

ACATCTGTGGCCAGTTGCTCACATCAATCACCTTATGATCTTCGTCGCAAGATGCCCGCT

AATGGTCACGAACAATGGTTCCCGTCCGCTTCCAATACTCTTGCCAACAGCAGTACGTCT

AGTGCATCTGCTAACACTAGCAATAGTAGCCCACAATCTGTTTCCTGCGGTCCGCCACAC

ATGGGTGGAAGCTGCAACATCTCATCTATATCTGCCATGCCATCATCGTCATCTTCAACT

TCATCTTCAGCCATTGTTCACGCGCCATCAACTAGTGCCACTTTTCCTGTGAATTCAACC

GCTACCGGTGCTCCACTAGGATCATCACCTACAGTACACAGTTCCATACCACAGCAACAT

TGCAGTGCTTTGCCGATTGGTGGCATTGAGGATAATAATCTTATGTCGCCAGCTCGCAAA

CGGTCACGCCGTTCATATGCACAAACGGCTGACATGCCTTGCATGGCAGCTTGTACGGGA

AGCACTACACCGCCCGGTCCAACAGCAGCGCAATATTTACAATATGAACTGCCGGACGAA

GTTCTACTTGCCATATATTCGTATCTTTTAGAGCAGGATTTGTGCCGTTTGGCAATTGTT

TGCAAACGCTTCAATACTATTGCAAATGATAATGAATTGTGGAAACGGTTGTATCAGTCT

GTGTTCGAATACGATCTGCCACTCTTCAGTACCGAACAAAGTAAGTTTGTCTTTGAAAAA

CCAGAAGAATCCGAGTATTCCAATCCATGGAAAGAGAGTTTTAGACAACTCTATAGTGGA

GTGCATGTTCGTGCTGGTTTTCAGGACAAAAAATATCCCGGACGTAATATATTATTCTTT

AACACCATACAAGCTGCACTTGATTACCCAGAACAAGCGGCCGCAGCCGCAATTGGCGGG

TATACAAATGTTCCCGCATCTGATCCTACCACGACCAATGATCTGATCTCTATCTACCAA

GACAGTATTACACCTGAAGACCATCCAGGCCCACTAATTTTCGTTCATGCTGGCCATTAT

AAGGGTGAATACTTGTATATTGAATCAGATGTGACATTAATTGGGGCGGCCTCTGGTAAC

GTGGCAGAGTCTGTGGTGTTAGAACGTGAAGCGGGTTCAACAATGATGTTTGTAGAAGGA

GCTAAATATGCTTACGTTGGCTATTTGACGCTAAAATTCTCACCGGACGTAACATCGACA

GTCTCCCATCATAAACATTACTGTCTAGATATTGGAGAAAACTGCTCACCAACTGTAGAC

AACTGCATTATACGCAGTTCGTCGGTTGTTGGCGCCGCAGTGTGTGTAAGTGGTGTTAAC

GCTAATCCAGTCATACGTAATTGTGATATAAGCGACTGCGAGAATGTTGGTCTTTATGTC

ACTGACTACGCTCAAGGAACTTATGAGCACAATGAAATCAGCCGCAATGCTTTGGCCGGT

ATTTGGGTAAAGAACTTCGCCAGTCCTATTATGCGCGAAAATCACATTCACCATGGGCGC

GATGTAGGCATATTTACGTTTGAGAATGGCATGGGTTACTTTGAGAAAAATGATATACAT

AATAACCGAATCGCTGGGTTCGAAGTGAAAGCTGGTGCCAATCCTACCGTTGTCAAATGT

GAGATACATCATGGCCAGACAGGCGGAATTTATGTGCATGAGAATGGCTTGGGTCAGTTC

ATTGAAAATCGCATACACTCGAATAATTTTGCAGGCGTTTGGATAACATCGAATAGTAAT

CCCACTATACGAAAAAATGAGATTTACAATGGACATCAAGGGGGTGTTTACATCTTTGGC

GAGGGGCGTGGGCTTATAGAACATAATAATATTTATGGCAATGCATTGGCTGGCATACAA

ATACGAACAAACAGTGATCCGATAGTGCGTCACAATAAAATACATCATGGCCAACATGGA

GGCATTTACGTGCACGAAAAGGGTCAAGGTTTAATCGAAGAAAATGAAGTATACTCGAAC

ACATTAGCTGGTGTTTGGATAACTACAGGCAGCACCCCAGTTTTGCGACGTAATCGCATA

CATTCCGGCAAACAGGTCGGCGTATATTTTTATGACAATGGGCATGGCAAGCTTGAGGAT

AATGACATTTTTAATCATCTTTACTCTGGTGTACAAATACGAACTGGCAGCAATCCCGTT

ATACGTGGCAATAAAATTTGGGGCGGACAAAATGGTGGCGTATTAGTATACAATGGTGGT

CTCGGCTTATTAGAACAAAACGAAATATTTGACAATGCCATGGCTGGAGTTTGGATAAAA

ACTGATTCAAATCCGACGTTAAAACGCAATAAGATTTATGACGGACGAGATGGTGGCATT

TGTATATTTAATGGTGGCAAAGGTATTCTTGAAGAGAACGATATCTTCCGCAACACGCAG

GCTGGCGTTCTCATCTCTACGCAATCCCACCCGATTTTGCGACGTAATCGTATTTATGAT

GGCCAAGCAGCTGGAGTTGAAATTACAAACAATGCATCTGCTACCTTAGAGCATAATCAG

ATTTTCAAAAATAAATTCGGTGGCTTGTGTCTAGCTAGTGGCGTGCATCCCGTGGTACGT

GGTAACAATATATTCAACAACGAAGACGAGGTCGAGAAAGCAGTCTCAGGCGGTCAGTGC

CTGTACAAAATTAGCAGCTATACATCATTTCCAATGCATGACTTTTATCGTTGCCAAACT

TGCAATACGACTGACCGTAATGCGATTTGTGTGAATTGTATTAAACATTGTCATGCTGGA

CACGATGTAGAATTCATTCGACATGATCGCTTCTTTTGTGATTGTGGTGCTGGTACGCTA

ACAAATCAGTGCCAATTACAAGGCGAGCCGACTCAAGATACGGATACACTGTATGATTCG

GCAGCACCTATGGAATCTCATACACTTATGGTGAATTAATTTTGCTAGTTTATAAGTATT

TCATTAAGGCCTACATTCGAATATAAATTTTATGAACGGGAGAGACAGTCAGATGATTTG

AGTTAACATATAATGTTGCATCATATATGTTATGATATTTTTATGTTATTTTGTGAGAAT

TCTCTCTGCACATCCTGATATTTCCTGTTATGAATCTAATATTTTTTTTAAGTTTATTCG

GCTTTATCCTTATTTTCGTGTGAAAAAATCATTGCTATAATTTGCAATAAAATACAAAAA

AATGTTTTGTAATTTATCATTTTTTTTTGTTTAGCATTGTAGTTTATGGGAATGTTTCGT

TCATATGGAATTCACGTTAAGGCTGAATAAACCACAGCAACACATTATGAGAGTGGAGTG

TGAGATACATAGTTTTGTTAGTAGAATACGATAACATATTCAACACAATTCTCTCAAATG

GCGATTTTAGTGGTTACCACTTGGCATAAACCCAATTATACTTACTTGCTTACGTTTTGC

TTTAATAAAAAGCAGAGCATGAAATTTGTAGATTCTTCAAAATCAAGCATTTAAAAGTAT

GTGCTATTTATTTTGACGTAGGCTTCTGTTAAGAAATCGATTATTACTATTCTTTTTTAT

ATACACATCATTTTAAATTAATTTTCCGATAAAAAACCTATTTTTAAACTAATAATTTTA

TATTCCCGAGCAACTCCGATAATATCAGGATGAACTGGGATATTTCATAGTTTTTTTTGT

TTAAATACTTCTAATTTTTGTTAATTGTAATTAAAAAAAAATTATTAAACATTTACATGA

CGTTTCGTTTGATTAGATTTTAATTGCTTGAAATATAGCGTATAGTAGTCCCACGAAACA

AATTATTCGTAAATTAAAAAAATTATTACAGTTTCGAATAATGTTATAAAAATTGCATCC

CTTACGAAACGTAATTAATTTATTCATATCCCATTCGTCCTATTCATTTCTATTGATATT

AGGCCTACAAGCAATATAAATGAACTTAAGTGAACATTAGTTAGGTATCTACTCAAATCA

CATGTGGATGGATGTGTAAAATAATTTCTGCATTATTATTTCGTAAACTCCAATTTATAT

GTTCACTTTAATCACGCATGTACAGCAAAGGATGGACGAAATAAAACTTCACGTCCATAA

TACAAATTGCGATAAATTTCAGAAGAAAAAAAGAATATGAAGTCATGAGTTTATGAGCAT

TTTGTCACATGGAGTAGAAAAGGAACACAAGATACTAGAAACACGATAATACAAAAATGA

AAAATTAACATAATCGGTTGAAAGCAGAAATTGACAAGTACTCATATACAAAGTTAATTT

AAGTTATATGATTTTTTTACATTTTATACAAATTGTATATTATAAAGAAAAGTTAAATTG

CGAAAAACAACTATGAACGATGCATAAATACACACATACAATGTGGTAAAGCAAAATTTA

AAATGTGCAATTAGAGAAAACTAATCAAACTACTAGAAGGATTGATCTATGAAAATATAT

AAACCACATTAGAAATTGCTTAGGTACTAGCACAAAAGTACATACAAACTCGACGTATAT

GATTATCAAGGTTATATGTAAGATAATCACCCACCCCACGCAACTCTTCTAATACTTAAC

CAGTTGAAGGGAACACTAACTATCTTCCTGTGTATTTAGGAAATGTTAAATTAGTTTACG

ACCTCGAGCGTCTCGCACGCATCAATTTCATTTTATTTCATAGAAGTAGACGTTTTCTTT

TTTATTTTGCATTTTTTCATGCAGTTTTTATATGGCAAATTATATTATTAAATTAAGTAT

ATGGTAGTAATAGGAGATTTCACACTGAATCCGTTAAAGTTGTACTTGTTATTCGATAAC

TCGATAAACTTTTCGAATACCGCTTCGCTCTTTTTACTCGTTAAAATGATAACTAATTTT

GTAAATCAACAGACATTTACAGAAGGAATTTTATTTATTGGAAAGACACAATTAAAATAG

TATACCCTATGATCAAAAAATACC

**tBLASTn(First hit)**

Score = 1540 bits (3987), Expect = 0.0, Method: Compositional matrix adjust.

Identities = 787/915 (86%), Positives = 826/915 (90%), Gaps = 28/915 (3%)

Frame = +1

Query 210 VQAPSTSATFPVNNAPTTGATLAQPPNVHSSVPQQHCGALPVGAAIEDNNYMLPARKRSR 269

V APSTSATFPVN+ TGA L P VHSS+PQQHC ALP+G IEDNN M PARKRSR

Sbjct 1816 VHAPSTSATFPVNST-ATGAPLGSSPTVHSSIPQQHCSALPIGG-IEDNNLMSPARKRSR 1989

Query 270 RLYTQGGEMgpsagatgeaagagtaasggagcaptaaQYLQYELPDEVLLAIFSYLMEQD 329

R Y Q +M A TG G A+ QYLQYELPDEVLLAI+SYL+EQD

Sbjct 1990 RSYAQTADMPCMAACTGSTTPPGPTAA----------QYLQYELPDEVLLAIYSYLLEQD 2139

Query 330 LCRLALVCKRFNTIANDTELWKRLYQSVFEYDLPLFNPELCKFVFEKPEESEYANPWKES 389

LCRLA+VCKRFNTIAND ELWKRLYQSVFEYDLPLF+ E KFVFEKPEESEY+NPWKES

Sbjct 2140 LCRLAIVCKRFNTIANDNELWKRLYQSVFEYDLPLFSTEQSKFVFEKPEESEYSNPWKES 2319

Query 390 FRQLYRGVHVRPGYQERRSSGRSIVFFNTIQAALDYPEERaaagvfvpagagagnvvsag 449

FRQLY GVHVR G+Q+++ GR+I+FFNTIQAALDYPE+ +A

Sbjct 2320 FRQLYSGVHVRAGFQDKKYPGRNILFFNTIQAALDYPEQ----------------AAAAA 2451

Query 450 LsntsasagvggasvnalvnIYEEQVAPTEHPGPLIFLHAGHYKGEYLFIDSDVALVGAA 509

+ + + N L++IY++ + P +HPGPLIF+HAGHYKGEYL+I+SDV L+GAA

Sbjct 2452 IGGYTNVPASDPTTTNDLISIYQDSITPEDHPGPLIFVHAGHYKGEYLYIESDVTLIGAA 2631

Query 510 PGNVAESVILEREAGSTVMFVEGAKYAYVGYLTLKFSPEVTSTVSHHKHYCLDIGENCSP 569

GNVAESV+LEREAGST+MFVEGAKYAYVGYLTLKFSP+VTSTVSHHKHYCLDIGENCSP

Sbjct 2632 SGNVAESVVLEREAGSTMMFVEGAKYAYVGYLTLKFSPDVTSTVSHHKHYCLDIGENCSP 2811

Query 570 TVDNCIIRSTSvvgaavcvggvnanpvIRNCDISDCENVGLYVTDYAQGTYEHNEISRNA 629

TVDNCIIRS+SVVGAAVCV GVNANPVIRNCDISDCENVGLYVTDYAQGTYEHNEISRNA

Sbjct 2812 TVDNCIIRSSSVVGAAVCVSGVNANPVIRNCDISDCENVGLYVTDYAQGTYEHNEISRNA 2991

Query 630 LAGIWVKNFASPIMRENHIHHGRDVGIFTFENGMGYFEKNDIHNNRIAGFEVKAGANPTV 689

LAGIWVKNFASPIMRENHIHHGRDVGIFTFENGMGYFEKNDIHNNRIAGFEVKAGANPTV

Sbjct 2992 LAGIWVKNFASPIMRENHIHHGRDVGIFTFENGMGYFEKNDIHNNRIAGFEVKAGANPTV 3171

Query 690 VKCEIHHGQTGGIYVHENGLGQFIENRIHSNNFAGVWITSNSNPTIRKNEIYNGHQGGVY 749

VKCEIHHGQTGGIYVHENGLGQFIENRIHSNNFAGVWITSNSNPTIRKNEIYNGHQGGVY

Sbjct 3172 VKCEIHHGQTGGIYVHENGLGQFIENRIHSNNFAGVWITSNSNPTIRKNEIYNGHQGGVY 3351

Query 750 IFGEGRGLIEHNNIYGNALAGIQIRTNSDPIVRHNKIHHGQHGGIYVHEKGQGLIEENEV 809

IFGEGRGLIEHNNIYGNALAGIQIRTNSDPIVRHNKIHHGQHGGIYVHEKGQGLIEENEV

Sbjct 3352 IFGEGRGLIEHNNIYGNALAGIQIRTNSDPIVRHNKIHHGQHGGIYVHEKGQGLIEENEV 3531

Query 810 YSNTLAGVWITTGSTPVLRRNRIHSGKQVGVYFYDNGHGKLEDNDIFNHLYSGVQIRTGS 869

YSNTLAGVWITTGSTPVLRRNRIHSGKQVGVYFYDNGHGKLEDNDIFNHLYSGVQIRTGS

Sbjct 3532 YSNTLAGVWITTGSTPVLRRNRIHSGKQVGVYFYDNGHGKLEDNDIFNHLYSGVQIRTGS 3711

Query 870 NPVIRGNKIWggqnggvlvyngglgllEQNEIFDNAMAGVWIKTDSNPTLKRNKIYDGRD 929

NPVIRGNKIWGGQNGGVLVYNGGLGLLEQNEIFDNAMAGVWIKTDSNPTLKRNKIYDGRD

Sbjct 3712 NPVIRGNKIWGGQNGGVLVYNGGLGLLEQNEIFDNAMAGVWIKTDSNPTLKRNKIYDGRD 3891

Query 930 GGICIFNGGKGILEENDIFRNTQAGVLISTQSHPILRRNRIYDGQAAGVEITNNATATLE 989

GGICIFNGGKGILEENDIFRNTQAGVLISTQSHPILRRNRIYDGQAAGVEITNNA+ATLE

Sbjct 3892 GGICIFNGGKGILEENDIFRNTQAGVLISTQSHPILRRNRIYDGQAAGVEITNNASATLE 4071

Query 990 HNQIFKNKFGGLCLASGVQPITRGNNIFNNEDEVEKAVSSGQCLYKISSYTSFPMHDFYR 1049

HNQIFKNKFGGLCLASGV P+ RGNNIFNNEDEVEKAVS GQCLYKISSYTSFPMHDFYR

Sbjct 4072 HNQIFKNKFGGLCLASGVHPVVRGNNIFNNEDEVEKAVSGGQCLYKISSYTSFPMHDFYR 4251

Query 1050 CQTCNTTDRNAICVNCIKNCHAGHDVEFIRHDRFFCDCGAGTLSNQCQLQGEPTQDTDTL 1109

CQTCNTTDRNAICVNCIK+CHAGHDVEFIRHDRFFCDCGAGTL+NQCQLQGEPTQDTDTL

Sbjct 4252 CQTCNTTDRNAICVNCIKHCHAGHDVEFIRHDRFFCDCGAGTLTNQCQLQGEPTQDTDTL 4431

Query 1110 YDSAAPMESHTLMVN 1124

YDSAAPMESHTLMVN

Sbjct 4432 YDSAAPMESHTLMVN 4476

**Conserved domains**

**protein containing domains F-box-like, Beta_helix, and zf-UBR**


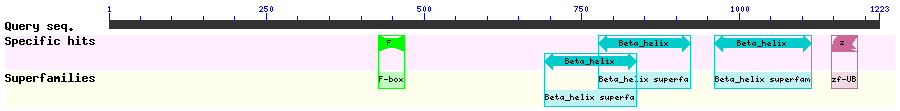


**HPS4 = CG4966**

**>TRINITY_DN31238_c0_g1_i2 len=2455** GTGGAGGAAGGCGTGTCCACACCAACGGCTGTGTTGCAAACGCCGGCAGCCGTGCGACCA

AAATCGTTGTTGATACGTCCCACCCACCTACCGTTGCGGCATCGCAACGGCATGGGTAGT

TCGAAGGAGCTGCCTGAGTCGGGCATAGCTTCGATAAACTTTGATGAGACAGATTCGTAT

CCGGAGTTCATTGGACGCACCAGTGTGTGTTCAACACCGATGACTGAGAATAAAGTGCTG

CAAGTGGGGAATATTATGTCAATTTGCGCCAATCCAGAGGATGAAAGTAATTCGAATAAG

CAAAAGACACAAACTGCGAACCGTCGTAATTCGTTGCGTCTTGATTTTGAGAAATTCTTT

CAAAATTTTATTGCTAATCCTAATAAACAATTGGAGCGACGTAAATCCTTTACCGATTTA

CAAGATTCACTTAAGAAAATCTCGAAGAAATTGTCACTGAAACAATTTTCGCATAGCTTT

AAGACTGACGTCAATCGCAATGGCAGCATCAGCGGCATCGAGGCCATCGAATCGCCCGAT

TTTATAGAGAATGACGATGATGCAGAGCAAAATTCAGACAACTCGGATGAGAATAACCGC

ATTTCACGAACCATAACAGATCCCACCTATCCTGTTTTTAACGAGAATGGCCAACCCATA

TCTCGTAGTCTTTTCCAAGAGTTTATCGAAAAATACTATCGCCTTTGGGCGGAAAGTGGC

AGTGGCAATGGCAGCGCAAAAAAGGATATAGAAATCGCACAACTCATTGAGGAATTCAAA

GAGTTTGATAAAGAGCTGAAAAAGTTGGATGGGTCGATACGTCAGGAAATAGGCGACAAT

ACCACCAACACCGCCACCACAACGCTCAAAGCAGATCGAAATTTAAATACAGAAGCACCT

GCAGTTGTGGCAACGCAAAAAGCTAAAACTCCACTAGACAAGAAGTCCCTTAGTCTTCCT

CTGAAGCCACTCGCCGATACAGCGTCAAACGAGCAATTTGCTGCAGCGAGTCGCAAGCAG

CCGGGTGGTGTGCCACTCACGCCACTCATGGCTAAGCTTTCAGTGTTGGCTTTGAATGAG

GAACATACGAGTAATACGTGGGATGCCAGTTCGGTAGAGATTCAGACGCCACTAAACACA

TCGAAAGTGTTTTCGCGCCGCAGTTCATTGAAATGTGAGGACGCTGTAGACGCCTTGGCG

ACATTGCCTACCAACGTACAGAGCACTTCGAATGGCTTAAAGCGGCTGGAGTTGTATATG

TGTGGCCAGCAAAATATGACTTTGTTGTTGTTAATGGAAGAGGGAACCGCGCGCCAGCAG

CCCATTGTGCATAATATGTTCGATATTTGCGTGGCGAAATTTCCGCACATGGAATCACAT

TTAAATCAAACACTCAATGTCAATGTGGAGGGCGATAAGCGTGAAGGCGGCTACAGTTTC

ATGTGCATCGATGCAAAGTGGGATACGCTAGAGCGCAATGGACCATGGAATCCGTTGGAA

TTGAATACACTGGAATGCATACATCAAGACTTACAGTCGAATGTTGAATTAACGGATGTG

GTTTTGAGGTCACACGACGCCGTCTTTTATGGCTACAAATCGGGACGCACCGAAGTTTTT

TACAAGGGAGCAGCACACCCAACCAGCGGCATACCGCCACCCTCCGATCCCATGGGAAAT

GTGGCGATGCGCGCCAAAACTCAGCTGGAGCGTGATCATTCGTACATTCTATTTTAAAAT

GAAGTAGTGGTAGGTTTTTAAAAGAACTTATGGAATTCTCAGCATTTGTGTGTTGCCATT

TTGCCACAAAGCAGCTGTTAGCAAAGTTAAAGCGTATGCAAAATGAAGTTAGTAGTATTT

TTTCTCTATTTTTTGTTTCTATAGTAGCGATATTCAGAAGCCAAAATATGTAGTTGCAAC

AAAAAAAGGAAACATTCACCTTAACTAGATAACTCAATAGTGCGCCACTGGCCTAACGCC

GCTTATCATACATACCTACATCATAAATACGAGATACTCTGACGAAACTACTCGCAACAT

AAAGTTTAATGTGAAAGTTAAAAGTGAATAGAAAGAAATGTGGTTGGCAGAATGTAATAT

TTACACAGTTAGTTGCTTTCTGTGGACGTGGCTGTATGTAGCTTTTTAATTATTGTATGT

ACTTATGCAATACGTTTTTTAGTTGATAGGCGAGAAAGCGCATGCCAAGGCTGTCGGATG

GTGAATATTTATAAAGTGACCTCGAATGGAAAGCAGTTTTAGAAAAACGGAGATCTTAAC

TCTCTTTTCTGCTCATAAAAGTATGCATCACTTTTTGTTGAAAACTTTTTAAAACAATGG

ACTAAAAATAGGAAATTGCGGAAAATTTATAAAAATTACTTATAAAATTAACCTAACTTT

TTTCTTGAAATTTTATTAAAATTTTTTGTTACAATTTTATTAAAAAAAAATAGCA

**tBLASTn(First hit)**

Score = 604 bits (1558), Expect = 0.0, Method: Compositional matrix adjust.

Identities = 353/583 (61%), Positives = 424/583 (73%), Gaps = 67/583 (11%)

Frame = +1

Query 305 AVEQLPPA-RPKSM--RPTHLPLRIKSM--QSKELPESGIASINFDETDSYPQFIGRTSV 359

AV Q P A RPKS+ RPTHLPLR ++ SKELPESGIASINFDETDSYP+FIGRTSV

Sbjct 28 AVLQTPAAVRPKSLLIRPTHLPLRHRNGMGSSKELPESGIASINFDETDSYPEFIGRTSV 207

Query 360 CNTPMTENKVLPVANVMSICANPEDEGKEEDIHNSNGK---THSRRNSLKVDVEKFFQNF 416

C+TPMTENKVL V N+MSICANPEDE NSN + T +RRNSL++D EKFFQNF

Sbjct 208 CSTPMTENKVLQVGNIMSICANPEDES------NSNKQKTQTANRRNSLRLDFEKFFQNF 369

Query 417 ISNPNKQLTRRKSSADLQDALRAISKKLN--NFTHGLKTdvnrngsgngdvssdsPDFIE 474

I+NPNKQL RRKS DLQD+L+ ISKKL+ F+H KTDVNRNGS +G + +SPDFIE

Sbjct 370 IANPNKQLERRKSFTDLQDSLKKISKKLSLKQFSHSFKTDVNRNGSISGIEAIESPDFIE 549

Query 475 DDDKI------------TSRTISDPTYPVFNTNGQQISRSLFQQFLDQYRKLW---GVAS 519

+DD SRTI+DPTYPVFN NGQ ISRSLFQ+F+++Y +LW G +

Sbjct 550 NDDDAEQNSDNSDENNRISRTITDPTYPVFNENGQPISRSLFQEFIEKYYRLWAESGSGN 729

Query 520 EQAHEDAELAALVAEFQEFNAEIQKLDEHMRQQAAEAS--------SADRNLNVSA---- 567

A +D E+A L+ EF+EF+ E++KLD +RQ+ + + ADRNLN A

Sbjct 730 GSAKKDIEIAQLIEEFKEFDKELKKLDGSIRQEIGDNTTNTATTTLKADRNLNTEAPAVV 909

Query 568 ----AKTPLDKRSMTLPLKSAGESTFGErasgrsgag--gVPLTPLMAKLSVLALSE--- 618

AKTPLDK+S++LPLK ++ E+ + S GVPLTPLMAKLSVLAL+E

Sbjct 910 ATQKAKTPLDKKSLSLPLKPLADTASNEQFAAASRKQPGGVPLTPLMAKLSVLALNEEHT 1089

Query 619 -----TTPIEIQTPLTTSKVFPRRSSLKCEdavdalaalttapaQPPGPIQP--DGLQRT 671

+ +EIQTPL TSKVF RRSSLKCEDAVDALA L P +Q +GL+R

Sbjct 1090 SNTWDASSVEIQTPLNTSKVFSRRSSLKCEDAVDALATL-------PTNVQSTSNGLKRL 1248

Query 672 ELYICGQQNMTLLLLMEEGTCQQQQVVQKMFDICVAKFPHMESQLNQTLNVNVEGDNRDG 731

ELY+CGQQNMTLLLLMEEGT +QQ +V MFDICVAKFPHMES LNQTLNVNVEGD R+G

Sbjct 1249 ELYMCGQQNMTLLLLMEEGTARQQPIVHNMFDICVAKFPHMESHLNQTLNVNVEGDKREG 1428

Query 732 SNYSFMCVDSKWDVLQRNGPWNPLELNILESMHSIHSSGHHLTDLILRSNDSVYYGHKNG 791

YSFMC+D+KWD L+RNGPWNPLELN LE +H S LTD++LRS+D+V+YG+K+G

Sbjct 1429 G-YSFMCIDAKWDTLERNGPWNPLELNTLECIHQDLQSNVELTDVVLRSHDAVFYGYKSG 1605

Query 792 RTEFFYKEPTHQINGIPPPSDPIGNIQSRAKSRLERDHSYMLF 834

RTE FYK H +GIPPPSDP+GN+ RAK++LERDHSY+LF

Sbjct 1606 RTEVFYKGAAHPTSGIPPPSDPMGNVAMRAKTQLERDHSYILF 1734

**Conserved domains**


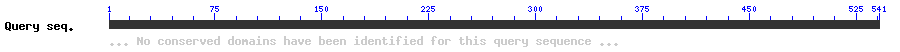


**SRRT = Ars2**

**>TRINITY_DN31881_c2_g1_i5 len=2707** CTAGGACATTTTAACTGTTTATGTTGAAGTCATAAATTGTACATGGTTATTAATTCGCTG

AACTTAATTTCTTGTTTTTTTTTTTTGGTAGTTTTCCTTTTGCCTCTTTCAGACTCTACA

TAGTATGTGTAGGTGTTTAATTTTATTCTGAAATGTGGCGTGTAACTATAGCAGTTGTAA

GTTGTTGTAGTAGTACTCCTACGAATCAAGACAATTTATTCGAACATATCGAAATTAGCA

GGCGCATCCAGGTCATGATAACCAATTATACGACGTTGCTGTTCCATTGGTTCTCTGCGG

CCAGGGCCAAATCCGCGTCCTCCGCGACCAGGCATCATCATCGGCGGTGCATAAGCAGCA

TATGGAGGCATAAATGCAGGTGGATATACTGGCGGTCGATAGCCTAAGCCACCAGATTCT

GAGACTGTACGCTTAGTGGAACTAGCATGTTCTGGAAGCTGTGGGCGTTTGGGGTCCCGC

AAATAGTTATTGAAGAACTCTACCTCCTTGCGTACTTCATCTACCTTTTCGGTATGCTTA

TTGAATATGTGTTTTCGTATAAATTCAGGACCTTTGAATTTCTTGCCCGATAACGGACAT

AACCACTTGTCCTTTGCTAATTCCTGTGTGTTCGCCTGCACGAACTTCTCTACTTCAACC

TCGGCATCTTTGCATCCCAAATTCTTAAGCTCTTCTTCTTCAACAGTTGTCGTCTTCGCT

AAGAATGTTTGCATTTTATTTTCAAAGTTTTTTATATAATCCTGTATATCATTTTGAGAC

ACTTTCGATGGCGGCGGTCCACGGGCGTGTATTATGCCACAACGATTTGGCATTTCATCT

TCATAGGGATACTCGCAGTGATTATAAAAATCGACTGAGTGAACGATGCGAAGATATAAA

ATCAGGCTATCAAGCACAGAAATAAGCTGTGCATCTCTTTCAATTATCTCCCCTTCAGAC

TCTTTATTTTCCCCAGATATACCAAGTAGTTCTTCTTCCTCTGCTGATGCTTCCTCGATT

AGGTAATCCGTAATATTTTGTAAAACTGGATTATTCGACTTAAAACCATAGGACGCACTG

ATATCACCATTGTCCTTGGAGTTCTCTGCATTTTGCTTAATATCACTATCGGACCCCTTA

TCCCATAATTTGAATTTTTCGTCCAAGTTCAAAGCAATCTTCGCACATAATTTGATGTCA

GCTCGTACAATAGATTTATGTGCAGTCATTCCATTTACTGGGCGTACTCGTCGGCTTAAG

TCACGATTCACAATTGCACCCATCTCGCAATCACGTAGTCGTGTATTGTTTAAATTCCAA

CATATCTCTTTGATGTTGACATCTCGTTTGAATGTTACCCAACCGCGACGGTACCATCGC

CGATCGACCAAAGGATCAGCTATTGCCACACGCAAATAACCATCAAAACGTTGGCATAAC

GTTTCAATTTCTGCCTTGGTTATAGAAGGCGCAAGATTCCGCAAAAATATCGAAGATGTT

CGATGAAGCGCGCGAGGTTGTGGACCATCCTTGACTTTGTCCAAGTCGATAGTCTCAGGT

TGAACATTATCCGGGTTTTCCTTTTCTTCAGTTTCAATTTCTTTTTTAGCTTCATCAACT

ACTGAATCATCTTTATCTTCCACCTCTTCCTGATCTTTAGCATCATCACTGTTTAAACTT

GTATCTTTCTTACTCTCATCATCTAGAATTTTTTCAACAACTTTATCAGTATCAATTTTC

TCCGCATGTTCTTCCTCATTATTATTTCCCTCATTATCTTCTGTCCTTTTCTCCTCAACA

AAACCATTTTGAGTAACTAACGGTTCTTTTTCATCCTCGCCATTTAATTTACTCTCAACC

GGAGGCGCTTCCGATTCTTCCTCAACAGCTTTATCCGTGTCATTTTTGTCTTTAAATTCG

ACTCCTTCATCCTTATCCGCTTCTTCTCCCTTTTCTTCTTTTACACTATCCAAGTCATAT

TTGTCTTTTACTTTATCATCATCATCTTCATCACTGGAGCTGGAACTACTGGAATCAGAA

TCCGAAGACGACGAAGAAGATGATTCTGAACTGGCTCGTTTACGTTTTAAGCACTTTTTA

TCCTTAGCCTTCTCGTCGTCTTCACTTTTATCTTCGTCATCAGACATATCTCTCTTATCC

CCACTTTTGTTAGCAATATTCTTACTGTCGTCACCATCATCATCGTCCCAGTTCTCCTCA

TCATCTGAAAGTTGGGCGCGACGTATCGGGCGTGGCGATACAACTTTCGGTTCATCATCG

TCTTCGTTTCGTTTTTTGCCCACCGAAGTTATAATCACATCGTCGTCATGTTCCGGCTTT

TTCTCGAAAGTTCTTTCTGGGAAGGTATACTCTCTAGGTTTCTCATCGAGTATTTTCAGA

TCATCATCCGTCCCACCTTCCAGTTTAATAACCACTGTATCAAGCAAACGAAGCAAGGGA

TCCGTTTGTGACGTATCAACTGATACAGTCTTTACTTGGCCATTTTCCAAGACTTCATTG

AAAACATCAACTCGTCTCTTTAAGAAACCGAGTTGTTCCTCCTTCCGCTTAACACTATCT

ACGGGATGATATTTGTTTTTAAACCATTCCTCGTCTTTGTGTGCAACGAAAAATTCATTT

AACTGCTGCCGCTTAAATTCAATTTTGTATTCGCTGTACTTTCTCAAAACTTCTGAGTCA

GAAATTC

**tBLASTn(First hit)**

Score = 635 bits (1637), Expect = 0.0, Method: Compositional matrix adjust.

Identities = 330/452 (73%), Positives = 366/452 (81%), Gaps = 4/452 (1%)

Frame = -3

Query 493 VETETIDLDKVKDG-QPRALHRTSSIFLRNLAPSITRSEIEAVCNRFSGYLRVAIADPLV 551

V+ ETIDLDKVKDG QPRALHRTSSIFLRNLAPSIT++EIE +C RF GYLRVAIADPLV

Sbjct 1565 VQPETIDLDKVKDGPQPRALHRTSSIFLRNLAPSITKAEIETLCQRFDGYLRVAIADPLV 1386

Query 552 ERRWYRRGWITFMRDVNIKEICWGLNNQRLRDCEMGAIVNRDLSRRVRPANGITAHKQVV 611

+RRWYRRGW+TF RDVNIKEICW LNN RLRDCEMGAIVNRDLSRRVRP NG+TAHK +V

Sbjct 1385 DRRWYRRGWVTFKRDVNIKEICWNLNNTRLRDCEMGAIVNRDLSRRVRPVNGMTAHKSIV 1206

Query 612 RSDIKLCAKIALNLDEKFRLWAEGPKDDsnsaranessengsGSTYGFNSQNPVLQNITD 671

R+DIKLCAKIALNLDEKF+LW +G D N ++YGF S NPVLQNITD

Sbjct 1205 RADIKLCAKIALNLDEKFKLWDKGSDSDIKQNAENSKDNGDISASYGFKSNNPVLQNITD 1026

Query 672 YlieeasaeeeellgltgeNKDTEGEPIERDEQLISVLDRLVLYLRIVHSVDYYNHCEYP 731

YLIEEASAEEEELLG++GENK++EGE IERD QLISVLD L+LYLRIVHSVD+YNHCEYP

Sbjct 1025 YLIEEASAEEEELLGISGENKESEGEIIERDAQLISVLDSLILYLRIVHSVDFYNHCEYP 846

Query 732 YEDEMPNRCGIIHARGPPPVRVTNNDVQEYIKIYESKLQQFLTKTVPLSDEEIKNLGAKD 791

YEDEMPNRCGIIHARGPPP +V+ ND+Q+YIK +E+K+Q FL KT + +EE+KNLG KD

Sbjct 845 YEDEMPNRCGIIHARGPPPSKVSQNDIQDYIKNFENKMQTFLAKTTTVEEEELKNLGCKD 666

Query 792 AETEVEKFVQANTQELAKDKWLCPLSGKKFKGPEFIRKHIFNKHEEKVDEVRKEVQYFNN 851

AE EVEKFVQANTQELAKDKWLCPLSGKKFKGPEFIRKHIFNKH EKVDEVRKEV++FNN

Sbjct 665 AEVEVEKFVQANTQELAKDKWLCPLSGKKFKGPEFIRKHIFNKHTEKVDEVRKEVEFFNN 486

Query 852 YLRDPKRPQLPEHPGTSKRPESESArgggggyrppmyppFSAMPYgfgppmmgggrggrn 911

YLRDPKRPQLPEH ++KR SES G P PY P M G

Sbjct 485 YLRDPKRPQLPEHASSTKRTVSESGGLGYRPPVYPPAF---MPPYAAYAPPMMMPGRGGR 315

Query 912 fpparrELPLEHQRRLIGYHDLDAPANSDMFD 943

R P+E QRR+IGYHDLDAPAN DMF+

Sbjct 314 GFGPGRREPMEQQRRIIGYHDLDAPANFDMFE 219

**Conserved domains**


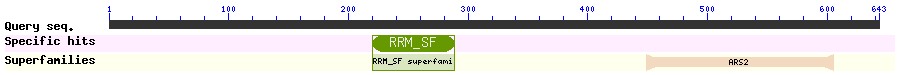


**CG4572**

**>TRINITY_DN33767_c1_g1_i2 len=5894** GTAGCGATAAACTTCTTTTATTTATTTCTTTTTTATAACAATTTTAGTTCTTTGGTTGAA

TTATATTCGCATTCCTTTAATTTTTTATTATAAAAATAGTTAATTGTGTTTGCAGAACAC

TTGAAATCACGCTGACACCAGTTTTTGCCTTTTATGTATTTATTCAACCGTTTTGTAGTG

GGTTAAATGATCTATCATCCAATGCAACCACTTTGGTTGGTCACCAGGCGCCATATGACC

AGCGTTGCGTACCAAAATTTCAATTAAACGACCAGCATGTTTGACATACCCGGCGATTTC

ATTGTCCACGCGCCAAATTCTTCGCTCTGCAATTTTATATTTGTCGGCGTCAGCGAATTT

AAGATGATTTAAGTAGTTGCGTGTCAAAGGGTAAGCTACAATTATATCCAACTGACCACT

ATAAATGCAAACAATATATTCATTTAACAGCTCCGCCACCCATGGTGCCACAGTATCCAT

CACATCCTCCTTCAAAAACTTCTCTACCTTATTTACCTTGTCGGTATCATGGAATGTCAT

GTTCCCGACGTGAATTGCTCTGCGAGTTAGTGTCGATTGTATGAAATTGCCAAGCGCTTC

ACCTGGTTCATCACTGTCGGTTTTCAAATAGTTATAGTAGAAATTATAACCAGTGAGATT

ATGAAAAATAGATCCATTTGTAAGATCCCCGTTAATAAGACCGTCGAATATGTCAAATGC

GCAGTCCATATCATGATCTTTTATGCATTTTTCACCCTTCGCTTCTTGGGTATGGAACTC

TTTTAAACCGTTATCGTCTATTAAACCCAACTGATATAAATAATCACCATATTTGAGCTG

ATGTAAAGGATCAGTAAGTCCATTACCTATAGCCAAACCTTTTAGTGGTATATAAACACG

TGTATCCACAGCATTTTGCATCAGATGAATATGATATGCCAGCGCTGGTACATATTTGCC

GGCGTAGGATTCCCCCGTTATCCAGAATCCACTTGAATTGTTCCATTCGAAGAGTTCATA

TAATTGCATTACAGCCTCGTGCAGGTTACGCCCTACATCTTTTTCGTTTCGAGCGTACCC

CTCATCACTACCTGTAAAGCTGAAACCAGTACCAACGGGATTGTCAATGTATATAAGGTT

GTGAGTGCGACTCCAAGTGTAGTTGCGCTTTTGCAAATGTCCGCGGGAGTCAAATTCAAA

AGGCCCATTTTCTGCAAATAAACCAAACAACGAAGATGCGCCAGGTCCTCCTTGTAACCA

CAAAACTACCGGTGCATATGTTGGATCTTTCTCAGCAGGAAAATGCCAAAAGAACATATT

TGATTTGCGCCCTGCATCAACTGTTAAATACCCAGCGTAGCTTTCTACATCATGGAACTG

ACTGCCAATTACAGCCGCCTTTTTTCGCACTTGGTCTTTCGGTATTTTCTCATCATGTAT

TAATGGCGTGAGGAAAAGTGGTTCACCGGGATCACCACCATCCTTATATTCATTAAAACG

TGGATACGGATTTATAAAACTTCGAGGGTACTTTAATTTGCGAGACTTCTTCGCATCGGC

GCTGATACATAGCGCCAACACCGCTAAGAGGGCGTACATTACAAAACGAGTGCCAAGATT

CATGGTTAATGGAATATTAGTAAGTAGCATTCTATTTGACGCAACATCTACCGTACCGAA

ATCAAAAATTTACTTTAGTACTTCACAAAGCAACCCTACCGAGTTGCTAGTTTTTTCCAC

ATCGTTTCTTTTCATATTCCTCTCAGTTACTCTCACAATCAATTCATTTGACAACTTGCA

CTACATTTTAACCGGAGAACGTATTTCAACGATGGCGTATTTTTCTAGATATGAACTATT

CCAATTTATTAATAATTAAAAAGAAATTAAGGGCTTAAAAACATAGAAGAGTGTTTGAAA

GAAAGAAAAGAAAAAGTGTAAATTAACTTTCTACAAAATATGATTTCAAAGAAGTGATGA

TACAAATAAATAGCAAAGTGGGTGAAAAAGTGTGGAAATGTTGAAGAAAATGTTTGGTCG

GAGGTTGGTGATGTCGCGATCGTGGACGTCGAAGTCGATTTTGTTTTTGCGTTGGAACAG

TGTGATTTGTTTCGGTTTATGTGTTTTGATTGCATCAGCCGCGGTGACTGCGACAACAGT

CACGCCAGGCGCAACAAAAATAAAAACAGTTGAAAATGCACATAACATATCGAAAGAAAA

TGGAAATAACAGTACATCTGAGGATAATACTGCAACCGAACAAGTACGAGTGTACCGCCT

GAGGGAGGCATGCACTGATTTGGCGCGAGACGAAGAAACATTAATGGTTTTCTCAACATT

GGGGGGCGGATTAACAGCCATCGATCCGATAACAACTGAAATTCGTTGGACCATAGCAGA

CGATCCCCCGATACGCGCAGAGCAAGAGCAAAATGTGCAAGTGCCACAATATTTTCCAGA

TCCGCGAGATGGCAGTATTTACCAACTTAGTGATTTGGGCAACTTGAAGAAATTGCCCTA

CACCATACCACAGTTGGTGGCCAGTGCGCCATGTCGCTCGTCTGATGGCATCCTGTATTC

GGGCAAAAAGAGCGATACTTGGTTCATGGTCGATCCTAAGTCGGGTAGACGTGAGAAAGT

CATGGGCTTCGGGTCGGGCGGTTCACAAGCCGCACAAAATCCAGAGGAAAATGAAAATAG

AGCAAAGCATACCAATTCGCATTCTATCTACTTGGGGCGCACACAGTACACCGTAATGAT

GTACGACAGCTTGGCCAAAGGAAAAAATGCTAAGCCTTGGAATATCACTTTCTATGATTA

CAGTGCGCACACCATGACGCCAGATATATCTAAAGAATATGAATATTTGCATTTAACCAC

CACATCCAATGGCAATGTAGTGACTTTGAATCGCAAGAATGGTAAATTTCTGTGGAAGCA

CGATCTAACGAGTCCCGTAGTGGCGGCATTTCTGCTCGGTTCGGAGGGATTGCTAAGCGT

GCCTTTTACCACTGTCTCCGATGAGGCTTTCGAAGCCATTCTGGAAGAATCCAAAACCGG

CAATGTGAATACCATCAAACTATTCCAATCGCTTTATGTGGGCGAGCATAGTCATGGCTT

ATATGCGTTGCCATCGTTGGTGGATAAAGATACACCACGCATTTCAGCAAGCTCTCCAAT

AAAACTTTTGGATGGCCCCAATTCTGTTGGTGATGAATCAGATTCCAAAATGGTTTATCT

GGACGATGTTATACGCAAAAATGTTGGCATTGTTCTCGGCCATTATAATATGCCGAACGA

AGGTTCTTTGGAAATATCGCCTTCGCCCGCAAAAGATGAAAACAGCCATGACTTGGCTAC

CATCAATCATTACCCCGATGTCACCACTATGGGCTCCAACGGCTATAGCATTCTTACAGA

ATCGAATAGCGAGAAGAATTCCGCTGAAATTGGCGTACAAACTGACCCAGTAATTGAAAT

AAAAACAGATTCAACTAACGCCTTTAACAAGACAAAAAGGATCATTCTGGCGAATAGCAA

TCGAATACAAAAATTCTTCAACGAATGGTTTATGGATCATCCGAGCGGCCAAGTGCATCA

GATACTGATCGTTTTGGTTTTGGTTATGGTCGCCATGTTCTGGTACATGTGCAGCACGAT

GCGTGAGCTGAAATATCAAAGTGAAAATGGTTCTAAAACTTACTCTGGTTCCAACAAGAG

TAGCATTGCCAGCAGTAGTGTAAATGCAAAGGACTTGATTGATCTTGGCGATGGAAATAT

ACGAGTCGGGAAAATTTCCTTCAACTCAAATGAGGTATTAGGCAAAGGGTGCGAGGGCAC

ATTTGTGTTCAGAGGCAGTTTTGAGGAGCGCTCTGTGGCGGTGAAACGGCTGCTGCCTGA

ATGTTTCACATTTGCCGATCGTGAGGTGGCTTTGTTGCGTGAAAGCGATGCGCACGAAAA

TGTGGTGCGCTATTTCTGCACTGAACAGGATCGGCAGTTTCGTTATATAGCAGTGGAGTT

GTGTGCGGCTACGCTGCAGGATTATACAGAGGGTGAGCGTAGCAGTGAGCTGCGTTCACA

GATCAACGTTTGGGAGATATTGCGCCAAGCGGCAGCTGGTGTGAGTCACTTGCATTCATT

GAATATCGTGCACCGCGATATCAAGCCGCAAAACGTTTTACTTTCATTGCCCGATGTCAG

TGGCACAGTTCGCATTATGATATCCGATTTCGGTTTATGCAAGAAGTTGAATTATGGCAA

GGCCAGCTTTTCACGCCGCTCTGGCGTTACCGGCACAGATGGTTGGATTGCGCCCGAAAT

GATGCGCGGCCAACGCACGACAACTGCTGTGGACATTTTTTCACTTGGTTGTGTCTACTA

TTATGTGCTCACCGCTGGTCATCACGCTTTCGGCGATGCGCTCAAGCGGCAGCACAACAT

ACTGTCGCACGAGTACAATCTGTCGAAACTTAAGGTGGACACAGATAATGAAACGAGCAT

GCCCGAGGAGGCGAGCAAATTTATTCTTGCTGAACAGTTAATAGCAGATATGATTCATAA

TGACGCGCAATGTCGTCCGCTGGCGCGCTGTATAAGCGCCCACCCGGTCTTCTGGAATAA

CCAGAAGATACTGGCATTCCTACAGGACGTCAGCGATCGCGTTGAGAAGCTGCAGTTTCA

TGTGGAACCGTTGAAGTCGTTGGAAAAGAATGGGCGCTGCATTGTGCGCAACGACTGGAA

TGCGCACGTCGATCCACTCATAACGGATGACTTGCGCAAGTATCGCGGTTACATGGGCGC

CAGTGTACGCGACTTACTGCGCGCGCTTCGCAATAAAAAACATCATTACCACGAACTCAC

TCCGGAAGTGCAGCAATTACTGGGTTGCATTCCGAATGACTTTACCAATTATTGGATTAA

TAAATTCCCCGAATTAATATCCCATGCGTATCATGCATTTACTATCTGTGCGGAAGAGCC

TATTTTTAGGCATTATTACAACACCAACTATCGCTTCAGTCGCCCATGGTATTTCGATGC

AGACGAAAATCTTTTCCCATCGCTAAAAGACGATCCCAAACCTTTGATGAAGCAAACTGC

GACGGGCACTAAAGACGAGAGCGCCAGTCCGAAACGCCTGCCGCGCACCCTTGACAAACA

ACAACAACAAGTGAAGCAGCGCAAAGGTGTTTATAAATTCCGCAAAAAGTCCGACCCGGA

GGCTGTTGGTGTTGGCGTTGGTTTGCAACGCAACCTGGAGTTAATGCCCACCTCTGCTTC

AGTGGCAGCTGAGGAAGGGGATGATGCGGGCAACAAACGAGATGCGTACGCGAATTTTAA

ATTTCGCCGGAATTATGGCAAATCAGCAAATCGAAATTTTAGTAATTTGAATGCGAATGC

GAATGACAACACTAATAATGAGGGCAACAAAGAAAAAATCGTTACATGGACGTTGCCTAG

CAAGCCGGCGAACGATGAATGAGAAAGTAAATTACACAAAAATTATTACTTAGTTTAATT

AAGTAAACTTTTTTAATTAAGTACACTGTTAGCAAGTATTTAGTTTAGATTGATAGCTAT

TTTAACTTTTAAACAATTTTTATTACAAGGACGTTATGTAGATTAGGAAAAGGATCAGTT

CTTTCCTGGTAAAGTGCTATGACAACCATACGAACTTTTTCGAAGCAATGCCACTTTGGC

TCAGGGTTTGGCATTTTGCCCTATCACTGAATTGCACTACTACTAATTTTTTAGTGGTAG

TTGAAATAAGGGTTTCATGATCACTAAATATTCAGTTGTTTAGTTTTCTTTCCGCTAAAT

CTTCAGTTTCCGCTTATTGCATTAATTATTTAGTGCATTATGCCCAACCCTGCTTTGGCA

TTATTATTTTGGAT

**tBLASTn(First hit)**

Score = 749 bits (1935), Expect = 0.0, Method: Compositional matrix adjust.

Identities = 355/484 (73%), Positives = 405/484 (84%), Gaps = 3/484 (1%)

Frame = -3

Query 1 MKTATHCAFLIIATIVAISGAKGVEGERP--YRRSFINPYPRYQFFDDGVDPGEPLFLTP 58

M T + ++A+ + + R Y RSFINPYPR+ + DG DPGEPLFLTP

Sbjct 1623 MNLGTRFVMYALLAVLALCISADAKKSRKLKYPRSFINPYPRFNEYKDGGDPGEPLFLTP 1444

Query 59 LINNASMSKQEVQKLARVVGSQFHGVESYSGYLTVDPGFKSNMFFWYFPAEQEPEYAPVV 118

LI++ + K +V+K A V+GSQFH VESY+GYLTVD G KSNMFFW+FPAE++P YAPVV

Sbjct 1443 LIHDEKIPKDQVRKKAAVIGSQFHDVESYAGYLTVDAGRKSNMFFWHFPAEKDPTYAPVV 1264

Query 119 LWLQGGPGASSLFGLFTENGPLELDGHGKLQKRNYTWSKTHNLIYIDNPVGTGFSFTEND 178

LWLQGGPGASSLFGLF ENGP E D G LQKRNYTWS+THNLIYIDNPVGTGFSFT +D

Sbjct 1263 LWLQGGPGASSLFGLFAENGPFEFDSRGHLQKRNYTWSRTHNLIYIDNPVGTGFSFTGSD 1084

Query 179 AGYATNEKDVGRNLHEAVMQLYELFEWSNSSGFWVTGESYAGKYVPALAYHIHKVQNAIE 238

GYA NEKDVGRNLHEAVMQLYELFEW+NSSGFW+TGESYAGKYVPALAYHIH +QNA++

Sbjct 1083 EGYARNEKDVGRNLHEAVMQLYELFEWNNSSGFWITGESYAGKYVPALAYHIHLMQNAVD 904

Query 239 TRVYVPLKGVAIGNGLSDPLHQLKYGDYLYQLGLIDEHGLQSFHDAEAKGAECIKSHDME 298

TRVY+PLKG+AIGNGL+DPLHQLKYGDYLYQLGLID++GL+ FH EAKG +CIK HDM+

Sbjct 903 TRVYIPLKGLAIGNGLTDPLHQLKYGDYLYQLGLIDDNGLKEFHTQEAKGEKCIKDHDMD 724

Query 299 CAFDVFDSLINGDLTNGSLFSNLTGYNWYYNYLKTHDDD-GANLGEFLQAGATRRAIHVG 357

CAFD+FD LINGDLTNGS+F NLTGYN+YYNYLKT D+ G LG F+Q+ TRRAIHVG

Sbjct 723 CAFDIFDGLINGDLTNGSIFHNLTGYNFYYNYLKTDSDEPGEALGNFIQSTLTRRAIHVG 544

Query 358 NKTFHDLDKENKVELHLKKDIMDSVAPWIAELLAHYTVCIYSGQLDIIVAYPLTRNYLNQ 417

N TFHD DK NKVE LK+D+MD+VAPW+AELL Y VCIYSGQLDIIVAYPLTRNYLN

Sbjct 543 NMTFHDTDKVNKVEKFLKEDVMDTVAPWVAELLNEYIVCIYSGQLDIIVAYPLTRNYLNH 364

Query 418 LKFPGSDKYKVAPREVWRVGKEVAGYVKHAGHLVEIMVRNAGHMAPHDQPKWLYMMIDHL 477

LKF +DKYK+A R +WRV E+AGYVKHAG L+EI+VRNAGHMAP DQPKWL+ MIDHL

Sbjct 363 LKFADADKYKIAERRIWRVDNEIAGYVKHAGRLIEILVRNAGHMAPGDQPKWLHWMIDHL 184

Query 478 THYK 481

THYK

Sbjct 183 THYK 172

**Conserved domains**

**protein containing domains Luminal_IRE1, STKc_IRE1, and RNase_Ire1**


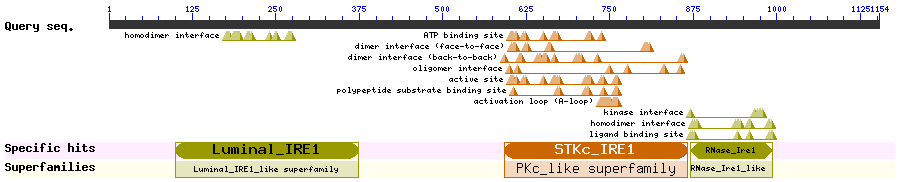


**Egghead**

**>TRINITY_DN32129_c1_g1_i5 len=3857** ACTAATCGTTGTTGCTGATACGGAATAAGGAAACGTTTTTTTATCTTTGACAGAAAAACT

TCATAGCAATCGAGATATTGGACGGTGTTCCAACAGTGATAAGGAATGACATCAAATTTC

AACATAAAAACCCTACGATATGCTAGATAAAAACACAACTATCACTCAGCACTTTATATG

AAGAGATTTAAAAACGATATCGATAATATGCCCGAACCTTGCCGACGACATCTCGACCAG

ACAGAAGCACACGAGGAACGTTGAAATTGTAAAAGCCAAAAAACTCAAAAATCCCGTACA

AATTGGATTGTGTGTCGCGATCAAAAAACACAAAAAAAAAAATAGAAAAACTCACATAAA

TGATAGAAAAAAACAAATATTTCGACAAAAATCGAATTCGACAACTCTAATAGTTAGAGT

TACCCAATAATTAGATTTGTAAGCCGAATAGTTAGCGAACTATAAGCTAAGATTTGCACA

CCCGTAACGCGAATGTGACTCGATTTCTTCGTTTGGTCAGCAAACAAAAAATTATCAGCA

GGCACAAATTTAAGTAAACTCTAAAGGCGACATTTTTGTTAACAAACCAAAAAAGAAGTA

GAAATTTAAAAGCATAAAAAAACCATCACGTATGCACTTAAGCATTTAAAAAACGTAACT

ATCGCACCAAAAATAACCACAAACAACAACTAGTAAATAGGGGCAAAGTATATAAACACA

AATTCAAAATAACGCCTGAATATCTTAACTGCAAGCACACTTGAAACTTAAGCAAAAAGA

ACTTAAATCCCTAGCTAAACAACTAAGTAAGCAACATAGCAAGTCAACAAGCTGTTAACA

AGCAATTTTGTACATTACAAATCGCGGTCATTTAAACCTCATTCACATACACAGATCCGT

CTCAGCAAACAATATAGTATAACAGCAAAAGCAGCAATAATCAAAAAGAAAGAACAAGAA

GAAGAAGAAAAAGGAAAGGAGGGAGAAATATTAAATAGCAAATTATTAACAAATTTAATA

AATATACGATATACGCCGTCAACAACACCATCAGCATCGGCAACAGCAACAGAAACATAA

TGAACTCAAGCACAAAGCATCTGCTGCATTGCACACTCCTTATCATTGTGATAATAACAT

TTGAAGTATTCTCTGGTGGTATTAAAATTAACGAGAATTCATTTACGCTCGTTGATCCAT

GGACTGAGTACGGCCAAGTGGCCTCCGTACTTTTGTACTTATTACGATTCCTTACTATTC

TTACGTTACCGCAAGTATTGTTTAATTTTTGCGGTCTCGTTATCTATAATGCCTTCCCCG

AGAAGGTGGTGCTAAAGGGTAGTCCAATTCTGGCGCCATTCATATGTATACGCGTGGTGA

CGCGCGGTGACTTTGCCGATTTGGTTAAGTCGAATGTGTTGCGCAACATGAACACTTGTC

TAGATACAGGTTTGGAAAATTTCCTCGTCGAAGTGGTCACCGACAAGCCAGTTAATTTAG

CACAACATCGACGTATACGTGAGATAGTCGTGCCGAAGGAGTACAAAACACGTACCGGTG

CATTGTTCAAGTCACGTGCTTTACAATATTGTCTAGAGGATGATGTGAACGTGTTGAACG

ACAGCGATTGGATCGTACATTTGGATGAAGAAACTTTGCTGACCGAGAATTCGGTGCGTG

GTATCATTAATTTTGTATTGGACGGCAAGCATCCATTTGGACAGGGTCTCATCACCTATG

CCAATGAAAATGTGGTTAATTGGCTAACTACGTTGGCGGATAGTTTCCGTGTGTCGGATG

ATATGGGCAAATTGCGTTTGCAATTCAAATTGTTTCACAAGCCGTTGTTCAGCTGGAAGG

GCAGCTATGTTGTTACGCAGGTTGCTGCTGAACGCCAAGTATCCTTCGATAACGGCATCG

ACGGCTCTGTAGCTGAGGACTGTTTCTTTGCCATGCGAGCCTTTGCCCAGGGTTACACTT

TCAATTTCATCGAAGGTGAAATGTACGAGAAGTCACCATTCACGCTGCTCGATTTCTTAC

AACAACGCAAACGCTGGTTACAAGGCATATTACTCGTCGTACACTCAAAAATTATACCAC

TCAAGCACAAACTCTTGCTTGGCATCAGCGTCTACTCGTGGGTTACCATGCCGCTATCCA

CTTCGAACATTATTTTTGCCGGCCTCTATCCAATACCATGCCCGAATTTAGTGGATTTCG

TGTGCGCTTTTATTGCCGCCGTTAATATATATATGTATGTATTCGGTGTCATAAAATCAT

TCTCGCTGTATCGTTTTGGGCTACTAAAATTTATGGCCTGTGTATTGGGCGCCGTTTGCA

CCATACCAGTGAATGTGGTTATCGAAAATATTGCCGTAATTTGGGGGCTATTCGGCAAGA

AGCACAAATTCTATGTGGTGCAAAAAGACGTGAGAGCAATGGAAACTGTTTGAGTGGAGA

GCGGAGAGTCTGCATCACCTTCACATTGCTTCAGCACTCTTTTATTAGCTGTCGCTATTA

AAACCACATAAGCGTAAAGATTGTGCTGCCCAGGACAAATATACTACAATTACCAAAAAA

TCAACACATATGGTGCGATACTTTGCTTATGCGTCCTTTGCTCTATGCCATTGTAGCCAG

CGTCCAATTAGCCGTCGCGGTCACCAGTGCCATCGCCTGGACACCATTATAGTTGATATT

GCTGTTGCCACAAAACAAATCTCCTGCGCACTTTTCACCTTTTGTCAAATCCTCATGAAA

ACTTGAATGAACAAATGCGAAATCACGCACAGACGCTCCTACCCAGCACCACCACCAACA

CTACGAACACCATCACGCAAACAGCACAAACCCATTTACAAAATTAGTTCACTCACACTT

ATAGCATAAATATCATTATAGTTTATATATATACATATATACATATATTTTACATACAGT

AATTAGATCTAAATTTGTTTAATATTATTACATGTTATGGAGAAAGAGTTATTGCATCAG

TTGTGTGTGAATAGTCAACTAATAATGATGTTGATTTGGTGATGATTAAAATGTTAATTT

TGTTGAACAATTCTATCAGACCCTAAGTATTTACTTATATATGTATACAGAAGCTTGCAA

CAAATATTTGTAAAAACTAACCGTAGTGTAACTTCTAACCATGAACTACTGTATGTACAA

GCAGACACCGTTTCACGAAAAAAGCGCACATGCGAATTGGTTTCGACATAAAGTGTCTGA

CCCAAAAGACATCTAATACATCTACTTCGTACACATTACCCCACTCTCTTTTATAATTTT

GTATAATACAAAGTTTTATTTTCTCTTTAACTAGTTTGAGTTACTCTATTGTAATTTTTT

TTCCACAAAAATTTAATTATTTAAGTCAAATATAGTTAAAAATTAATGTAGCAAAAGTCG

TAAATATTTTTAGTTTAAAGTACACAATCTACAATATAAGTTAAAAAAATACATCCCAAT

TGTTGCAAGCATAGAAAAAATTGAAAAAGCAACAAAAACACACTGTAAAAAAGCTTTAAC

AAATTGAATGAATATTTATGTACATATTATTTTCTATATGTTTTTCTTTTTTATATACTT

ATACAACCTCTTATTATTTTTGTTTATATATTGTCTCATTGTCGCATTAGTTGTAAGCCT

AAAACAAAGTGAAGCAAATTATTAAAATCATCATCTTCGACAAAGTTAAATTACATTAAA

TTGTTAACACGCATACAATACAAAAAGTAAAATTTAAATAAAACAACAACAAAGTATTAC

CATGAAATGCTCTTTTTTTGTTCCATCGTATTGTTTTACATATAATAACATATATGCAGT

GGTTACATATATACAGT

**tBLASTn(First hit)**

Score = 863 bits (2229), Expect = 0.0, Method: Compositional matrix adjust.

Identities = 428/457 (94%), Positives = 447/457 (98%), Gaps = 0/457 (0%)

Frame = +3

Query 1 MNSTTKHLLHCTLLITVIVTFEVFSGGIKIDENSFTLVDPWTEYGQlatvllyllrfltl 60

MNS+TKHLLHCTLLI VI+TFEVFSGGIKI+ENSFTLVDPWTEYGQ+A+VLLYLLRFLT+

Sbjct 1080 MNSSTKHLLHCTLLIIVIITFEVFSGGIKINENSFTLVDPWTEYGQVASVLLYLLRFLTI 1259

Query 61 ltlPQVLFNFCGLVFYNAFPEKVVLKGSPLLAPFICIRVVTRGDFPDLVKTNVLRNMNTC 120

LTLPQVLFNFCGLV YNAFPEKVVLKGSP+LAPFICIRVVTRGDF DLVK+NVLRNMNTC

Sbjct 1260 LTLPQVLFNFCGLVIYNAFPEKVVLKGSPILAPFICIRVVTRGDFADLVKSNVLRNMNTC 1439

Query 121 LDTGLENFLIEVVTDKAVNLSQHRRIREIVVPKEYKTRTGALFKSRALQYCLEDNVNVLN 180

LDTGLENFL+EVVTDK VNL+QHRRIREIVVPKEYKTRTGALFKSRALQYCLED+VNVLN

Sbjct 1440 LDTGLENFLVEVVTDKPVNLAQHRRIREIVVPKEYKTRTGALFKSRALQYCLEDDVNVLN 1619

Query 181 DSDWIVHLDEETLLTENSVRGIINFVLDGKHPFGQGLITYANENVVNWLTTLADSFRVSD 240

DSDWIVHLDEETLLTENSVRGIINFVLDGKHPFGQGLITYANENVVNWLTTLADSFRVSD

Sbjct 1620 DSDWIVHLDEETLLTENSVRGIINFVLDGKHPFGQGLITYANENVVNWLTTLADSFRVSD 1799

Query 241 DMGKLRLQFKLFHKPLFSWKGSYVVTQVSAERSVSFDNGIDGSVAEDCFFAMRAFSQGYT 300

DMGKLRLQFKLFHKPLFSWKGSYVVTQV+AER VSFDNGIDGSVAEDCFFAMRAF+QGYT

Sbjct 1800 DMGKLRLQFKLFHKPLFSWKGSYVVTQVAAERQVSFDNGIDGSVAEDCFFAMRAFAQGYT 1979

Query 301 FNFIEGEMYEKSPFTLLDFLQQRKRWLQGILLVVHSKMIPFKHKLLLGISVYSWVTMPLS 360

FNFIEGEMYEKSPFTLLDFLQQRKRWLQGILLVVHSK+IP KHKLLLGISVYSWVTMPLS

Sbjct 1980 FNFIEGEMYEKSPFTLLDFLQQRKRWLQGILLVVHSKIIPLKHKLLLGISVYSWVTMPLS 2159

Query 361 TSNIIFAALYPIPCPNLVDFVCAFIAAINIYMYVFGVIKSFSLYRFGLFRFLACVLGAVC 420

TSNIIFA LYPIPCPNLVDFVCAFIAA+NIYMYVFGVIKSFSLYRFGL +F+ACVLGAVC

Sbjct 2160 TSNIIFAGLYPIPCPNLVDFVCAFIAAVNIYMYVFGVIKSFSLYRFGLLKFMACVLGAVC 2339

Query 421 TIPVNVVIENVAVIWGLVGKKHKFYVVQKDVRVLETV 457

TIPVNVVIEN+AVIWGL GKKHKFYVVQKDVR +ETV

Sbjct 2340 TIPVNVVIENIAVIWGLFGKKHKFYVVQKDVRAMETV 2450

**Conserved domains**

**Glyco_tranf_2_3 and Glyco_trans_2_3 domain-containing protein**


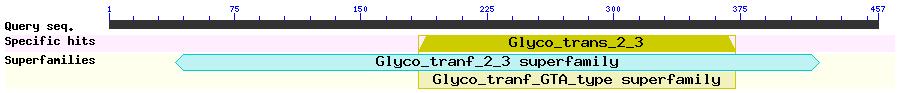


**ninaC**

**>TRINITY_DN26176_c0_g1_i5 len=6061**

GGCGTCTGTGTTTTGATTGATATGTAATAATTTATTAAAATGGCGAAAACGTTTGCGGCT

ACCACAACATTGAAGAAAATTACCCTTAATTACTAAAAAACTAGCAATGACAATTAAAGT

ACAATATTAAATATCGACAGCGTATCCAGATAATGTAACCCCCGGTGCAATTTCCTCCTC

AACGTATGAACCAGCCTCTTCAAATGACTTGGCATCTGTATTTTCGAAACTCTTACCAAC

CACGGTAGCAGTTTGTTTTAATGGTGGTGCTCGGTTGGAAAATTTTATTTGGTCAGTTTG

CATATTATTTGAAGACGAATTTTCGAAATCATCTAAGCGTCGTCCCGTGTGACGTAGTTT

CGATGCTTGAAAGTTTATGTTTTCATTGTTGTTATTTGGCATGTCGTTATTAAATTCATA

AATGGTTGAGTCAGGGCGAGAATAATTAGTTTTACGAAGCATAGCTTTGAAATTAAACGG

AGGATTTTCAGAACTATCCTCATCATTGGAACGGCGCACTATGTCCTTCAACTCTCGCAC

CGGGTCGGAAGGTATAAATGAAGAACGCTGTTGATAGTTCTGATTCATGCGATTCATGTG

ATTTTGGGCTTGTTGCGCCATTCGCGACTGCATTTGATCATTTTGATAAACCATGTGTGT

GGGAGGCATGGGAGGAGGCGGCGGTGCCGAATAACCCTTTAAAGAGTTTCGTCGCGAACC

CGCACGGTTGCTCCATATTTGTGAAGATGCATTGTTGTAATTATTATTGTATTGGCTATA

ATTGTTGTATGCATTCATATTCATTTGCACTTGATTCCTTTTTATTAGCTGTGGGTCACG

GTAATAGTTTTGGTTGTAGATTACATCACTTTCTCCTATTCGATCCCAGTTTGTCTGGCA

AGCTTGCTCCTTTTTATGCGCGTTATATGTAAGTAGACATGATGTCATTGATGGATTTCG

CTGCAGCGGACTATCCCAAACCTCTTCATCATCATGACTGATCAAAGAATTTGAAAAATT

TTGCCTTGAAACTGAATTCGCCGGGTTAACCATGTACTGAGTATCATAAAAAGGTATCTG

GTCTAAACGAAAGGGTAGTTTCTTTGTAGGTATTGGCAATGGCCCAAGTTGAGAAGAGTT

GACTTCGCGTGTATTTATACGATCAAACGGAACGTTGCGTGTGCATTTATTTATTCCAGC

AAAAAATCGCTGATTGTAAATGTGTACCTGCTGCGCCAAATTAACAAAATCTTGGAATCG

CGCTGCTCGGTAGTGTAATAAAACTTGAAATAGAGACTTCTCGCGCCACTTCTTAGCATA

ACCGCGTATAAAATCCGCCGTCTGCTCATTTAACTGGCCGGTCTTTTCATTGACCAAAGG

CTGAAATCGTTGTCTCTCATGATACCCACGAAATGCTGTAAGTGAAATACAATAATACAA

TAATGCGATATTTGTATATTTGTGTATTGTTTATTTAATTAAAATAAAAAATCACAAATC

TCCTTATTTTTCGTTTTTATTGATAGGCTGGGCAACCTCCAATATAATGCAATAAACGTA

CATATGAGAAACTATTTTAGAAAATCGAGGCTCTTATGTTCTTGTAAGTATATTTAAAAT

GTTTGAAATTTATTTTTTACTTTTCTATGTGCCTCACTTTTGCTTAAGCCAGCACTAAAA

TATTTGAAATGTTGTAAAGTGGAACGAATGCGTCATTCTTTGCGTTACTTGCACAAGTGT

TCCCAGTTGAACTAGGGATCATCGAGCACTGGAGGAGTTCGAGAGAATCTTCCCGTACCC

CTACTAAATGAGTAATGAGAGAGTTTTGGTTGAAAATCTCACTTTCCTCCCAACCGAGCA

ATGAAGGAGTTTTGGATAACCATCCCTCAAGCCTCCTATATAAGCAATTGAAGAGTAAGT

ATTGATACCTCTTAAAATAAAGAGTACTTATAAAACCCTTATAAGCGAAGAATAAGATAT

TACTTTAACAAATCAAAAAAATTACCAATTTGAAAAAGATAAGAGTAACTATATCGCTTT

TTTCATTGATTTAATTCAGAGGCAATTTGAAAGTACTCGTTTATTAATGACTTAAAAAGA

ACAAATAAAGGAGAAATCTCTTCTATTGCTCAAAAAGTATGTCAAAGAAAGTACTCTTTT

ATTGATAACTTGGGAAGACCTTACTTCAAGTTTGTATAACGTTATTTTTGTAGCACAACC

AAAGTAACCTCCCTTCAATATAGGCTAAAAAATGTCTTGCCGCTTATTTAAACTTTTTTT

ATATATGTATATACATACAAACAATTGCATGTAAATTTTCCTTTATTTGTATGCATAGAA

GTAAATCTGGTATTTTGGTTATGAATATAGCTTAAATTTGAAAAGCAGCTGAAGGTACGA

AAATTGAAGTATGGCATTATTTTCATGGCATGTTATTATTAAAATTTTGCTAATTGTAGA

GAATATCCTGTGATAATTTCTAATTTTTTTTGTAATTTTGTATTTGGTATTTTTGTCTAA

TCGTCCTCCGTTTGAGTTGCCTGCTCCACTTCCGCTAAACGTGCAGCTGAAAGTGCCTCT

TCCTTGCGTGCAGATTCAAAGAGTTCCTCCGCTTCAGATGGCGTTTCACTGGCATCAAAG

TATTCAACATCAAAATCTTTTTCCGAGCGCTCCTTATTACCTTTTTGAATTTTAGCGGCA

GCCTCATCATTGCGCGTGATTCCTTTGGCTTTTTTGGGATCCTTGCCTCCTTTCATGCGC

TTTCTTGCCAGCAAAGCTCTCATCATTGATTGAACCTTAATGACTTTTTTTACTTGAACT

TCATACAGTCTGGCCAGAAATTCATCATTATAATAACGTAGAAATACTTTCATTTTTCCA

ATGGCCCACCCTTCCATTTTTAAACGTATCAGCAAAAGGCGGCAGTTTTCCTTAGTAATA

TCCACGGTTTCATCAAAATCGAAAGCTAAAAATTGGTAGCGTCTAAGGAACTCTTGGAAT

GTGATCCGACACGAGTAGCCGCGTTGCCTTCCGATGACTGTATCCAACACACCAAGAGCC

TTCATTTGTTGTTGTACCATGTCAGCGTGGAATGCTCGTGGTTTGTATTCCAAGTCTGCT

CTTATGCATCGAACAAAATGCACACCAAGATTGATATTGCCTGTCAAAGTCTTTAATAAA

TTTAAACACGTGAATCGAAAATTTGCTGCTAAAGTTCGTAAGTTGTTCACTTGCGAAATA

CACCCGGCTGATAAAGTATTTAGTGGATATGATTTCCTCTGTGAGTCTTCCGGCTTGTGC

TGCACGCTTTCAAATGGCATTGTTAAGTTACCTGCTTTCGTGAGTTGGTTAGTAAACATC

ATCATGATATTTTCATCCATCGACGAGCGAAAGGATTCTATCATTTCCGGCGGCACAAAG

TCACGATTGATATCTGTGAAGGCGCGCGTATCATATATGATGCGCCCTGTATAATGAGCA

ACAGAAATTTCGGTAGCTGTATGTTTTTTAACAAATTGGCTGTGCTTTTCCACAACGCGA

TCCATAATAAGGTCTTGGTCTTGACTGGTGCGAGAAGCATCATCAATAATGTAAAATAGT

CCATCTGGTTTTGTCAGTAAATTATCAAGTGCTACTTTATTATCGTAGAAATTCAAATTG

TCAGTGGGTATATCATCCGCTTCCATTTCAAGCATTTCATTCACGAAAATACGTTGATTA

TAATGGTACTGCATTTGCTCATTGAAAGTATTTATCATCAGTTGTTCTAGACCATTTCTA

TGGAAGCATTCGAATCCGTACATATCGTGAATTACAACAGCATTGGTGTCGCCAAAAACT

GCACGAGGAAATGACAAATTCATATTAATCTTATTGATAATCCAGTCGACGAGGCGGCAG

TAAATAGTTGATGCAACAGCATCCCGAGCATCGCGTGCTTCTTCAGTGCTATACTGACGG

CGTTCTGCTATTCCACCTTTCACCATAATGAAGTTAGTTAATGACCACATATACTTTTTT

TCATCCACACGCAACAATTCCGCAATGCGCGTAACCATTTCGGTATTTTCCACTTCAGCA

TATTTACCAGAATGTCGGAAACGAATGTTGCCAATATTTAAGATAGCTGCAAGTACCTTC

CGTAACGTTTCCAGCTGTTTGTGGTTAAAGTCCAGGTCTTGCAAAATAGATTCAAAACGT

TTATAATTTTCAACATTTCCAATGGGGTCATCACGATGGTATTTTAATTTAGTGGTTTGG

GTATCTGCAGGAATACGAAGATAACGATACCCACGGTCATTCTTTAAATGATATTCCTTT

AGCAGATTTCCACTGTTCATGAAATCGTAGAAATAGTAGAAGATATGAAAATTGTGCTGA

TTCATATCGGTTGTAGAAACTCGAAGCTTCTCCAGCATATACATGTTGAAAACGGCGCCA

CTAAGTTTTCCGGTTTGACCGAAGGTCAGGAAGTACTGCAATACACATCTTGTGGATTCG

TTGTTAATGGGTGTACCAGCGTTAACCAGCATTTGAATTGCGTCAATCGAGCTCTCCACT

CGTGTAGTAGCTCCACGATTGCCGGATCCCAAGTAGCATAAATGGTTTATAAGTAAACGG

ACATTGGTTGATTTACCGGAAAAGCTCTCCCCTGAAAATACAATATGTTGCGGCTCCTTA

TGATGTAACATATCCTGGTAAGCAATGTCCGCCACAGCAAAAATGTGTGGGGAATTTTGT

GAGCGCGACTTAAATTTATATTTACTGTGAAACTCTTCATCGAAATCTTTTTGCAAATCG

TTCGAATTTAGTGATAATAAAACGTCTCCGATAAAACTATAAGAACCACCAAGCTCTATG

CGATTTCGCAGTGACTCCAAAATCATTTCTTCTGTAGCATTCTCGATAGCAGCCAAGTCT

TCTGGATACATTCGTTCTGGCTTTCCATCGAATCGTTTTATATAACCTCTATCAACAAAG

ATTTCTGGTTCTTTGTATATCGCTGGTGCGTCTCTACAGAAAACGAGTAGTTCACGAATA

TCTGCTTGCATCTCTTCATCATTGTCGAATAGTTCATTCAGAAATGGATGTTCGATCATT

TCCACCATCATTGGCCTATTTTCAACATTTTTTTCCAAACACTCTGATACGAAATCATTT

ATTTGTTGAGACCAATTCGTTGGCCGTAATAAAGTAGGCGGAGGGTTACGCACTATTTGG

AACATAACACGTGTGGGATGCATATCAGCAAAAGGTGCTTGGCCATCTGCCAATTCAATT

GTTGTAATACCTAGCGCCCATACATCTGCCCTTGAGGTGACTTCAGGTTCCTTTGCACTC

ATCGCAGTTACAACCTCTGGCGCCATCCAACAGGGTGATCCGATGCATGTGCCCCGCTTA

CCGAATGTTGAATCTACTTCTCGCGATAAACCGAAATCACAAAGTTTCACTCGCCCATCC

TTTGTAAGTAGGATATTATCACCTCGTATATCTCTATGAATTACGTGATTTCTATTTAGC

TCAATAGCTCCCCGACATGTTTCACGTATAATGTAAGCGATATGCTCTTCTCGCATGCGA

CGTTCAAGTTTTACCAATTTATTAACCATATCCACAGCTGTGCCACCACTGCAATACTCC

ATGACAAACCATATTTCATCAGGCGCATTTGGCTTTACAAGCTTATAGACGCCATAAAAT

TCTGGCAGGTTCGGGTGGTTGCAATGATCACGCAGGGTGCGGTATTCTTCTTCGATTGAT

ACTTGATGATCTTCATCATAGTGTTGAATTTTTAAAGCTACCATCCGATCATTCTCCAAC

TCCTTTGCTCGAAATACCTTTGCGTTGACACCTTGAGCAATTTCTTCGTAGATTTCAAAT

TTGTCTGTCGGATCCGGCAGTTGTGTGTAAGGTAAATACATTTTTTGTGTTTTTCAAAAT

CCTATAGATCTTAATTTTTACTTATTTTATATTATTAAACTAAATTCACACTTTTACTAG

CTTTTGAAAAACTTTACTTCACCGTAACAGCCTGCAGCTCCATACGCTGCATAACGCGCA

C

**tBLASTn(First hit)**

Score = 1894 bits (4905), Expect = 0.0, Method: Compositional matrix adjust.

Identities = 895/1082 (83%), Positives = 998/1082 (92%), Gaps = 2/1082 (0%)

Frame = -3

Query 2 MYLPYAQLPDPTDKFEIYEEIAQGVNAKVFRAKELDNDRIVALKIQHYDEEHQVSIEEEY 61

MYLPY QLPDPTDKFEIYEEIAQGVNAKVFRAKEL+NDR+VALKIQHYDE+HQVSIEEEY

Sbjct 5921 MYLPYTQLPDPTDKFEIYEEIAQGVNAKVFRAKELENDRMVALKIQHYDEDHQVSIEEEY 5742

Query 62 RTLRDYCDHPNLPEFYGVYKLSKPNGPDEIWFVMEYCAGGTAVDMVNKLLKLDRRMREEH 121

RTLRD+C+HPNLPEFYGVYKL KPN PDEIWFVMEYC+GGTAVDMVNKL+KL+RRMREEH

Sbjct 5741 RTLRDHCNHPNLPEFYGVYKLVKPNAPDEIWFVMEYCSGGTAVDMVNKLVKLERRMREEH 5562

Query 122 IAYIIRETCRAAIELNRNHVLHRDIRGDNILLTKNGRVKLCDFGLSRQVDSTLGKRGTCI 181

IAYIIRETCR AIELNRNHV+HRDIRGDNILLTK+GRVKLCDFGLSR+VDST GKRGTCI

Sbjct 5561 IAYIIRETCRGAIELNRNHVIHRDIRGDNILLTKDGRVKLCDFGLSREVDSTFGKRGTCI 5382

Query 182 GSPCWMAPEVVSAMESREPDITVRADVWALGITTIELADGKPPFADMHPTRAMFQIIRNP 241

GSPCWMAPEVV+AM ++EP++T RADVWALGITTIELADG+ PFADMHPTR MFQI+RNP

Sbjct 5381 GSPCWMAPEVVTAMSAKEPEVTSRADVWALGITTIELADGQAPFADMHPTRVMFQIVRNP 5202

Query 242 PPTLMRPTNWSQQINDFISESLEKNAENRPMMVEMVEHPFLTELIENEDEMRSDIAEMLE 301

PPTL+RPTNWSQQINDF+SE LEKN ENRPMMVEM+EHPFL EL +N++EM++DI E+L

Sbjct 5201 PPTLLRPTNWSQQINDFVSECLEKNVENRPMMVEMIEHPFLNELFDNDEEMQADIRELLV 5022

Query 302 LSRDVKTLYKEPELFVDRGYVKRFDEKPEKMYPEDLAALENPVDENIIESLRHRILMGES 361

RD +YKEPE+FVDRGY+KRFD KPE+MYPEDLAA+EN +E I+ESLR+RI +G S

Sbjct 5021 FCRDAPAIYKEPEIFVDRGYIKRFDGKPERMYPEDLAAIENATEEMILESLRNRIELGGS 4842

Query 362 YSFIGDILLSLNSNEIKQEFPQEFHAKYRFKSRSENQPHIFSVADIAYQDMLHHKEPQHI 421

YSFIGD+LLSLNSN+++++F +EFH+KY+FKSRS+N PHIF+VADIAYQDMLHHKEPQHI

Sbjct 4841 YSFIGDVLLSLNSNDLQKDFDEEFHSKYKFKSRSQNSPHIFAVADIAYQDMLHHKEPQHI 4662

Query 422 VLSGESYSGKSTNARLLIKHLCYLGDGNRGATGRVESSIKAILMLVNAGTPVNNDSTRCV 481

V SGES+SGKSTN RLLI HLCYLG GNRGAT RVESSI AI MLVNAGTP+NN+STRCV

Sbjct 4661 VFSGESFSGKSTNVRLLINHLCYLGSGNRGATTRVESSIDAIQMLVNAGTPINNESTRCV 4482

Query 482 LQYCLTFGKTGKMSGAVFNMYMLEKLRVATTDGTQHNFHIFYYFYDFINQQNQLKEYNLK 541

LQY LTFG+TGK+SGAVFNMYMLEKLRV+TTD QHNFHIFYYFYDF+N N LKEY+LK

Sbjct 4481 LQYFLTFGQTGKLSGAVFNMYMLEKLRVSTTDMNQHNFHIFYYFYDFMNSGNLLKEYHLK 4302

Query 542 ADRNYRYLRVPPEVPPSKLKYRRDDPEGNVERYREFENILRDIDFNHKQLETVRKVLAAI 601

DR YRYLR+P + +KLKY RDDP GNVE Y+ FE+IL+D+DFNHKQLET+RKVLAAI

Sbjct 4301 NDRGYRYLRIPADTQTTKLKYHRDDPIGNVENYKRFESILQDLDFNHKQLETLRKVLAAI 4122

Query 602 LNIGNIRFRQNGKYAEVENTDIVSRIAELLRVDEKKFMWSLTNFIMVKGGIAERRQYTTE 661

LNIGNIRFR +GKYAEVENT++V+RIAELLRVDEKK+MWSLTNFIMVKGGIAERRQY+TE

Sbjct 4121 LNIGNIRFRHSGKYAEVENTEMVTRIAELLRVDEKKYMWSLTNFIMVKGGIAERRQYSTE 3942

Query 662 EARDARDAVASTLYSRLVDFIINRINMNMSFPRAVFGDTNAIIIHDMFGFECFNRNGLEQ 721

EARDARDAVAST+Y RLVD+IIN+INMN+SFPRAVFGDTNA++IHDM+GFECF+RNGLEQ

Sbjct 3941 EARDARDAVASTIYCRLVDWIINKINMNLSFPRAVFGDTNAVVIHDMYGFECFHRNGLEQ 3762

Query 722 LMINTLNEQMQYHYNQRIFISEMLEMEAEDIDTINLNFYDNKTALDNLLTKPDGLFYIID 781

LMINT NEQMQYHYNQRIF++EMLEMEA+DI T NLNFYDNK ALDNLLTKPDGLFYIID

Sbjct 3761 LMINTFNEQMQYHYNQRIFVNEMLEMEADDIPTDNLNFYDNKVALDNLLTKPDGLFYIID 3582

Query 782 DASRSCQDQDLIMDRVSEKHSQFVKKHTATEISVAHYTGRIIYDTRAFTDINRDFVPPEM 841

DASR+ QDQDLIMDRV EKHSQFVKKHTATEISVAHYTGRIIYDTRAFTDINRDFVPPEM

Sbjct 3581 DASRTSQDQDLIMDRVVEKHSQFVKKHTATEISVAHYTGRIIYDTRAFTDINRDFVPPEM 3402

Query 842 IETFRSSLDESIMLMFTNQLTKAGNLTMPFEAVQHKDE-SERKSYALNTLSAGCISQVNN 900

IE+FRSS+DE+IM+MFTNQLTKAGNLTMPFE+VQHK E S+RKSY LNTLSAGCISQVNN

Sbjct 3401 IESFRSSMDENIMMMFTNQLTKAGNLTMPFESVQHKPEDSQRKSYPLNTLSAGCISQVNN 3222

Query 901 LRTLAANFRFTCLTLLKMLSQNANLGVHFVRCIRADLEYKPRSFHSDVVQQQMKALGVLD 960

LRTLAANFRFTCL LLK L+ N NLGVHFVRCIRADLEYKPR+FH+D+VQQQMKALGVLD

Sbjct 3221 LRTLAANFRFTCLNLLKTLTGNINLGVHFVRCIRADLEYKPRAFHADMVQQQMKALGVLD 3042

Query 961 TVIARQKGFSSRLPFDEFLRRYQFLAFDFDEPVEMTKDNCRLLFLRLKMEGWALGKTKVF 1020

TVI RQ+G+S R+ F EFLRRYQFLAFDFDE V++TK+NCRLL +RLKMEGWA+GK KVF

Sbjct 3041 TVIGRQRGYSCRITFQEFLRRYQFLAFDFDETVDITKENCRLLLIRLKMEGWAIGKMKVF 2862

Query 1021 LRYYNDEFLARLYELQVKKVIKVQSMMRALLArkrvkggkvfklgkkgPEHHDVAASKIQ 1080

LRYYNDEFLARLYE+QVKKVIKVQSMMRALLARKR+KGGK K K +D AA+KIQ

Sbjct 2861 LRYYNDEFLARLYEVQVKKVIKVQSMMRALLARKRMKGGKDPKKAKGIT-RNDEAAAKIQ 2685

Query 1081 KA 1082

K

Sbjct 2684 KG 2679

**Conserved domains**


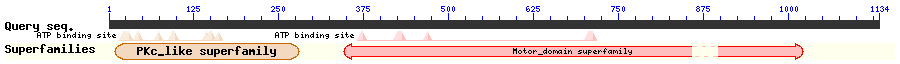


**Snipper**

**>TRINITY_DN31391_c0_g1_i1 len=2466**

GTACGAGTATATTTTTATTAGTTTACGCATATAAAATGTATTTGTATAAAAAATGCAGTT

TCTTTAAAATTGTTTTTAAAATTTAGGCCATTTGCAGGCTATAGTTGATATTCTGCCTCT

TCTACTCAATAATACCTAACCTAATTTATTATATGCTGTATGTAGTTGTGCTGTAATAAT

TTATGTATACCAAAAACAAAAAAAAGTACAAAAAAACCAACAACAAAAAAAATCGAATAG

TTAGTCTTTCATTAAAGTACTTGTTACGATTACAGACTTGCAGCTGAATTCAGACAAAAT

AAAATAAGTTCCAAATACGTCACTAATTTAATTACTTCAAAGCTTATTTTAATTTACTTA

TTCAAATATGTTTTTTTTTAGTAATTAGTAGTTCATAAATAGAAAAATATATAGTATTGC

AATATTGTATTTTTTATAAGCAACTCAAATCCGTTTATTTTCATGAACAAAAGCTTTTAC

TGATCAAACTATATAATAAAAAAAAAAATTGTGTAGAAAAATACGTCACTAAACGCTAGA

TGCCTAAGATATGTGCAGAGTAGAGCAAAAAAGGACAAACTTTCATTTAAATAAACATAA

ACGCACATGCACGTAGGTATATGACTAAATTCAGTTAATAAAACTAAACTTTAAAACAAC

AAAAAACACTTCACTATTACTCAACTAGTACCAAAAGCTGGGCCTTTGATAATACATAAA

ATTCAATTTAATCAAAAACCACTTGCAACTCAACTTTTAATACAAAACCTTGTTCGCTAA

ATATTATTACGAAATTCGCATACATTCAGGGTATATTCTCTTGGGCTCAATGTAATTTCA

CAAATAAAAAACTAAAATCTATTCCGCCCACGCACTGTTCTCCTCCCAACATTTCAGTCG

ATCGCTGCTTGTCGCTTATAGTATACAATTCGAGTTGAGTTGAAATGGCGCGAGGTCCTT

AGTTATCGCAAATTGTGCCCCATCGCTTGCAATCTTGTACGCCAGCGCTGCCAAATTGCG

CGCATCATCCCTTCCCGAATGCTCACGTCCTTCAAATACAAGCTTAACATGCGACAACGC

ATCGGCAAAATTGATGGGTCTGTATTTATACCATTGGCGAAAAATTGCACGCAAATCAAT

CCACTGATTGAAGTAAGACGGTTTCTTCAGCAACTTGCGCTTACATTCTTTCGCCAAACA

AATACCGAAATCCCAGTCAGTCCAGGTGACAAAGGCACAATTACCAATCAGTTTATCTTT

GGACATTTTAGGCAGTTGTAGATTGCGTGCCCGCAACTCTTTGCGTAACCACTCTTGAAA

CATCATAATTGCAGTTTGTAGTGGGACACCATTGTCGACAGTTTGCTGTTGAATGCCAGT

CAGTTCGGTGCAGTAAGCGCTCAATTTGGGCGATTCAATGGGCATAACGTATTTGTGAAA

TTCTGCTTCAATTTTACCCGTCTGCAGGTTGACCAACATGGCTGGGAATTCTATTATTTC

CGACTCACGCCACTTAGGTGGCGCTTGGTTTTCCCAGCAAGTCGCCTCAAAGTCTATGCA

TATGACATATTTGTATGGCTGCATGGTTAAATGTGGCTTGCGTAGCTTTGTTAAGTAACT

AGAAGTATTGGTTTTATCAGCGCCAGGTTCGCCTTCGACGTATATAGTCTCAAGTAGACC

CAGTTGCCTAGCCAGTCTTGTCAGAGCCATCTCGATGTGGTCAAATGTGAATAGAAGAAA

TTCCTAAGGATAAATGCGCTCTTCTATATGTCCGGATATCCACTCAACAGTTCGTTGTCC

TTTTCGCAGTTGGGCAGCAGCAGCAGTCGAAGACGAAAGCGAGTTGCAATGCAATTGTTT

GTGTGTTTGTTGGCTATTTCACCACTTTTCACTGTCTGCAGGTTTAAAACAAAAAAAAAA

AAATAATTTTGGCAGTGATTTCTTTTCTGCAGCTCTTCTAAACGACACAACCGCAATGGT

AACACTTTCTCTAGCTCACCGGCGCTTCTCACGCTTATCATAAATTTTTTACGAGTTTCT

ACGTCCCCTCTTACGCCAATTTGTTACTTTGCAGCGGTTATTGCACCACAAACGCGTATT

GTTTGTGTTTTTATTGTTGCGTTGTTGTTTTTGCCGGTCGCTTGTTTATCACATACCGCA

CTAATTTGACTACTTGTTTTCTATAGACGTCGTATTTTGTTTTCGATTTTTATCAAGTTC

AACTGCTTACCTGTTATTCTGCTCAAATGCTTTTGTTTTCTCACGTTTATTTCTTCTTTC

GTTTGGTTTTTTTAATCAGTTTCTCGGTGTGTTTACATTACATTTTACGCAAATTAAATA

AATTACCGCCGTTTGATGGGTATTTAACATTAGCATTTATTACAAAAAATCTATTTTTAT

ATTCTTGACATTGGTTAGTTGTTCCACAGAAATTGGGAAATTTTCAGTTACTTATTATTT

ATTGTA

**tBLASTn(First hit)**

Score = 388 bits (996), Expect = 7e-128, Method: Compositional matrix adjust.

Identities = 183/281 (65%), Positives = 220/281 (78%), Gaps = 17/281 (6%)

Frame = -1

Query 1 MALIKLARQLGLIDTIYVDGARPDPNNDPEESFNEDEVTEANSVPAkskksrkskrLAMQ 60

MAL +LARQLGL++TIYV+G +P D + + + L MQ

Sbjct 1710 MALTRLARQLGLLETIYVEG---EPGADKTNT-------------SSYLTKLRKPHLTMQ 1579

Query 61 PYSYVIAVDFEATCWEKQAPPEWREAEIIEFPAVLVNLKTGKIEAEFHQYILPFESPRLS 120

PY YVI +DFEATCWE QAPP+WRE+EIIEFPA+LVNL+TGKIEAEFH+Y++P ESP+LS

Sbjct 1578 PYKYVICIDFEATCWENQAPPKWRESEIIEFPAMLVNLQTGKIEAEFHKYVMPIESPKLS 1399

Query 121 AYCTELTGIQQKTVDSGMPLRTAIVMFNEWLRNEMRARNLTLPKMNKSNILGNCAFVTWT 180

AYCTELTGIQQ+TVD+G+PL+TAI+MF EWLR E+RARNL LPKM+K ++GNCAFVTWT

Sbjct 1398 AYCTELTGIQQQTVDNGVPLQTAIMMFQEWLRKELRARNLQLPKMSKDKLIGNCAFVTWT 1219

Query 181 DWDFGICLAKECSRKGIRKPAYFNQWIDVRAIYRSWYKYRPCNFTDALSHVGLAFEGKAH 240

DWDFGICLAKEC RK ++KP+YFNQWID+RAI+R WYKYRP NF DALSHV L FEG+ H

Sbjct 1218 DWDFGICLAKECKRKLLKKPSYFNQWIDLRAIFRQWYKYRPINFADALSHVKLVFEGREH 1039

Query 241 SGIDDAKNLGALMCKMVRDGALFSITKDLTPYQQLNPRFVL 281

SG DDA+NL AL K+ DGA F+ITKDL P+ QLN +L

Sbjct 1038 SGRDDARNLAALAYKIASDGAQFAITKDLAPF-QLNSNCIL 919

**Conserved domains**

**ERI-1_3'hExo_like domain-containing protein**


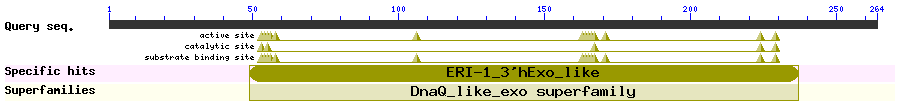


**Nibbler**

**>TRINITY_DN29782_c2_g2_i1 len=3535**

GTAGAATCAATATTACCTTGCATAGATTTGTCTGTCGTTTTTCGAGTTTTTCGAATAAAT

ACTTCTTTTTTTCACGTGTGGCTTCAGTTTTATTTAGTTTCTCTTTACGTACATGTTTAG

TGAGTTCACAAGTCGTGCTATTTGTCAGTAATTAAATATCTTTGAAATATTTAATGAACA

TTAATACTTTCTTATGAATGCATCGTATTTCTCAAACATATGCATTAAATTTTTTTGTAT

ACTTTGGCATGGTTCAATTTAGATAAGTGTGTACATAAATATCAGTATAAATTCCGAAGC

ATGGCAGCGCTGTCTGAAACTACACAATAGCAGAGAATTTGGAATTACTTGTTTTGACCT

TAAAAAAAGCGAATAATATTGAATTGCATTGTAAAATAACTACTCACGATGAGTATTTGT

CGTATTCCTGCCGGCTATGAGGACGATGAGGATGACTTAAGTGAAGTGATGCAAAAAATC

ACTGTCGTACCAGACATTACAGTCGGTGCGGGTCTGAATAACGAAAATTTCGACCGCAAC

CTCAGCTCCGAAGTAGCCTCCTGGTTCTTTTTCTACAAGGATGAATGGAATTCATACAAA

CGTATGCCGTCTGCTCAGCACACTCTCAAAGCTATGCTAATGACTGATGAGGATCCTCTG

CTATTTGCCTTAAAATTATTTGCAAATTGTCCAGGATGCAATAACTTAAAAAACAAAAGC

TTGGCGCTATTCATACTGGAAACCGTTTGTGACCTTCATAAAACAAAGCCAGAGATATCT

ACTAATTGCACAGACAACACCCGCATGATTGCTTTCAATTTTGTGAAGACTTGCGGCATA

GTACCGCTATGTAAGGCTGTGATTTCCACGTATGAATTAAAAAAGATTCGTGACCTTTTG

GTGCCTAAAATTAAGGATTTAATAGCAGCAGGGCATATCAAAGATGCTGCACATTGGACG

ATGCACTTGCAAATTACACATATTTTTGGGATTTTTGATATCGTATTCCCATTAATTCTA

CAAGACAAAGTCTCACTAGCGGAGGAGTATCTTAATGTTGCCAAAGATTTGGCATTGCCA

ACAGTGAAGTTTCTTGATTCCCTACTGGATAAACAAAAGACTGTTATAGACCATTGTGAA

GAAGTATTAACAAAATATGAGTACAAAGATGTGAAGTGTAATATCTTAACCTATCGCCCC

ATGTCCAAATTAGTCACACGGCTGGCGAAGAAATACAAGATTGACGCTCATGATACACCA

AATCTGAATTTTACAAAAGCTTGCTCGTATTTACATTACTTGTGGCGTAAATACCGAGAT

GGTGGAATGAGTCACGATGCCTGGGTTGAATTGGCACACGACGCTGCAGTTACAAAACCA

ATACAAATGGATTTGATTAAAAATGTGGTCGCTTATGGCGACATTAAAGAGGCGGGCTAT

TGGATAAGTTTCTATAAAATACCACTGGAAGAGTGTCCGGATGCTGTACTCGACTATTTG

AAACAAAATCGACCTTATGGAACTGACAAACATAATACCAGCAATAAATCCTCAAAATCC

GCTAATAGTAGTCGCGGAGCAATGAATAAATCTAAACGCTCGAATTCTTTCAAAAACAAT

CAATATTTAACGTTAAATCTCCCCATTGACTGCATTATTATTGTCGATGATCGTGTAAAA

TTCCTTCAAATGTTAGATCATTTAGAAGGTCAATTGCTGATAGCGTTCGATTCCGAGTGG

AAGCCAACGGTTTGCAACGAAAACGTTATTTCAGTCTTACAGATGGCTACTATGGATCGC

GTTTATTTATTAGATTGCCTATCGTCCAATCTTAGCAATGAGTTGTGGCAGCAGCTCGGA

CGTAGAGTGTTTAATAATTTGGAGATTCTGAAAATAGGTTTCTCCTTACACCAGGATTTG

CGTATGTTGCATAAATCATTGCCATTAGAATTAAACCTCAATGCAAAAACGTGTTACTTG

GATTTACGCGAACTATGGCGACGCTTAAAGTACTTACAAATTGTTAAATTTCCATTTGAG

TGTGCCTCTAACTCGGGCGAGAGTTTATGCACATTGACCGAAATATGTTTGGGGAAGAAA

TTAGATAAATCAAATCAATTCTCGAATTGGGCCAATCGACCGTTGCGTCACGATCAAGTC

ATATACGCGGCACTGGATGCGCACTGTTTATTGCTAGTATACCAGGTAGTCTCAGAAATA

CTGGTACGTATGGGTTTAGATGTTGACCAGGTAGTCGATGAGGTGACAACTGGGAAAACC

GGCTACTTGTTCCAAAAGAAACGCGTTATTACCGAGCAGAACGATGAGCGCAGTTCAACT

AGGCCAGTTAAAATGGATGCAGAAGGAGTTAATTCAGTTAGTGCAAAAAATGAAAACAAT

AGGTCAATTGGGACAAAATTTATTTGTGATACCATGATGACTGGGCTGTCGAAAGAGTTC

CGTAAATTAGGCATCGATTGTGTGGAAATTGTCAATAACGATTTGGGCTATTATATTGAA

TTAGCTAGAAAAGAACACCGTTATATACTTACACGAGATTCTCGTTACGATTTATTCGTG

AACGAATTGCCGCCAGAACAATGTCTACAAATTCCTAGTGACTCCAGCGTAGACCAAGTA

CTAAATATAATACGCCTGCTGGATATAAAAATATATGAATGCAATCTCTTTACACGTTGC

TTGAGCTGTAATAGTAATGAATTCATATTCGCACTGAGGCATGAAATGCAAACGATGCGT

TTTGGACAGGCTCTGGACGAGATTAAAGATATGAGTGCGTTAAATATGAATCCATATGGA

AAAACTTATCACTTATTGAATGTTAGCAGTCATATTGTGAAAACAAAAACGACCTATCGG

GGCAAGTTTATCAAGTTGAATCGGATTAGGTCGCACATATTACGCTCCAAGGAATATTTT

TATATATGTGATAACTGTGGCATTTGTACATGGGACGGTGCTTACTCCATACATAGCAGT

GTCAAGGATGCTGTTCTAAGCGATGTCTTCGAAGCTGCTACTGCTCCTGGAATTTGAGCA

CATAAATATATATGTATTGTATATATATAATATTAAATCATGACGACTGACTTTTTTGCA

TTGAGTGGTATTAAGTTAACACTGAGAAGATACTTTTTTTCAATTTCTCAAACACATAAG

ATCCTTTTTATATAGCTCTATATGTGAAAATGTATACCTTTATATTAATGAAATCCTATT

TTCATTTTGTGTATTCAAATATAAACGCCTATTAACTTATACATATGTATATTCGTATTT

TTAAATTAGTACAAAATCAACAATATTACAAATAATGAAAAGTACATTGACGTAAATTGA

GAGTACATGAAAGAAACGAAATACAAATTAAATAATGCATATGTATGTTAGTAGTAAGTA

CATTTAATCTAGAAGTAACAAGTAAGAGGATACATTAGTGTAGCGTGCAAATCTCAATAC

AAGTAAGACATACATATGTGTCTGTAATAAATGAATTTAATAAAATGTATGCTAG

**tBLASTn(First hit)**

Score = 475 bits (1222), Expect = 2e-152, Method: Compositional matrix adjust.

Identities = 269/605 (44%), Positives = 387/605 (64%), Gaps = 23/605 (4%)

Frame = +1

Query 11 IPAGFESDEENMENLMSNLKIKRLEDITTGAGIDGCNFDATLDAKAEEFFKLFREKWNMY 70

IPAG+E DE+++ +M KI + DIT GAG++ NFD L ++ +F ++++WN Y

Sbjct 424 IPAGYEDDEDDLSEVMQ--KITVVPDITVGAGLNNENFDRNLSSEVASWFFFYKDEWNSY 597

Query 71 SKKKSPHLRQEFGRALMGHQDPLLLALKIFANCPDSSNIKTKSLSHFVLDTVCKLHKDFP 130

K+ P + LM +DPLL ALK+FANCP +N+K KSL+ F+L+TVC LHK P

Sbjct 598 --KRMPSAQHTLKAMLMTDEDPLLFALKLFANCPGCNNLKNKSLALFILETVCDLHKTKP 771

Query 131 HLGEGCDPNTSMIAFNFVKTSGLLALNNAVIHAYSLRQIRDlllpklrellDNGLYKEVT 190

+ C NT MIAFNFVKT G++ L AVI Y L++IRDLL+PK+++L+ G K+

Sbjct 772 EISTNCTDNTRMIAFNFVKTCGIVPLCKAVISTYELKKIRDLLVPKIKDLIAAGHIKDAA 951

Query 191 QWSISLQLTHEFDMLELAFPLIAIEKLPLAEEYLDHATQQRLPFVKFLDSLLHKEKSVLE 250

W++ LQ+TH F + ++ FPLI +K+ LAEEYL+ A LP VKFLDSLL K+K+V++

Sbjct 952 HWTMHLQITHIFGIFDIVFPLILQDKVSLAEEYLNVAKDLALPTVKFLDSLLDKQKTVID 1131

Query 251 LCEHLLDRYKNLKISHNVLSYRPMAKIVARLAKKYGFDDAVTPNYKFTKTCSYLHYLYRE 310

CE +L +Y+ + N+L+YRPM+K+V RLAKKY D TPN FTK CSYLHYL+R+

Sbjct 1132 HCEEVLTKYEYKDVKCNILTYRPMSKLVTRLAKKYKIDAHDTPNLNFTKACSYLHYLWRK 1311

Query 311 YEKTRMNLASFREVVSVHAFNHELRTDFVKYLASAGAHSEAIYWYTEFNIDPKDCPLEIE 370

Y M+ ++ E+ A ++ D +K + + G EA YW + + I ++CP +

Sbjct 1312 YRDGGMSHDAWVELAHDAAVTKPIQMDLIKNVVAYGDIKEAGYWISFYKIPLEECPDAVL 1491

Query 371 TQVSQN---GAGK-------------ASGWESPGKERCPSSRCDMYLTMDLPDECLIIVN 414

+ QN G K + G + K R S + + YLT++LP +C+IIV+

Sbjct 1492 DYLKQNRPYGTDKHNTSNKSSKSANSSRGAMNKSK-RSNSFKNNQYLTLNLPIDCIIIVD 1668

Query 415 KADEFDRMLYHLQQECVIYLDSEWMQSVCGDNQLCVLQIATGHNVYLIDCLARESLRSEH 474

+F +ML HL+ + +I DSEW +VC +N + VLQ+AT VYL+DCL+ +L +E

Sbjct 1669 DRVKFLQMLDHLEGQLLIAFDSEWKPTVCNENVISVLQMATMDRVYLLDCLS-SNLSNEL 1845

Query 475 WRLLGANIFNNVNIRKVGFSMVSDlsvlqrslplqlrlqMPHHYLDLRNLWLELKKQRFG 534

W+ LG +FNN+ I K+GFS+ DL +L +SLPL+L L YLDLR LW LK +

Sbjct 1846 WQQLGRRVFNNLEILKIGFSLHQDLRMLHKSLPLELNLNAKTCYLDLRELWRRLKYLQI- 2022

Query 535 VELPFGNVNRAGDALTDLSLACLGKKLNKSNQCSNWANRPLRREQILYAAIDARCLMLIY 594

V+ PF + +G++L L+ CLGKKL+KSNQ SNWANRPLR +Q++YAA+DA CL+L+Y

Sbjct 2023 VKFPFECASNSGESLCTLTEICLGKKLDKSNQFSNWANRPLRHDQVIYAALDAHCLLLVY 2202

Query 595 NTLIE 599

+ E

Sbjct 2203 QVVSE 2217

**Conserved domains**

**
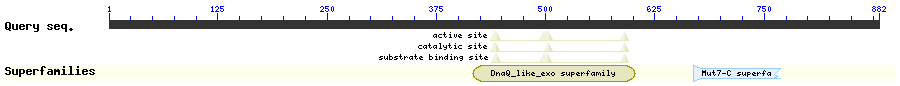
**

**Saposin receptor**

**>TRINITY_DN32577_c3_g2_i1 len=3661**

ATTAAATTTTAAAATACACTTTTATTTCATTACTAATAACATTACATACTTGTATTTCAT

ATCAATTAATTGGAATCTTATCATAGAAAAATCGTTCAAACCGAATGTGACATACAGACT

TTACCGTGATATCAGTGCTTTTCATTACATAGTAGATATTTTCATTTTCGTTTTCAAAGC

AATGTCGCTTACATGTATGTATATATAATATATTTTGTTATTCTTATTAACATAAAACTT

TTGCGTTCCTATAAAAATTTTGGTACCATTTCATACTTCAAGTTTCTTAACCAAGCTTTA

TACAAAACCACTCAAATTACCGCAGCACTTAAGAAATTAAAATATGCATTCATAAATTGA

ATATGCAAAAGTGCAATTTATCTTTTTTTATTCATAATGGTAGAACGTGTTAATGATAAT

TTAGTAGTAAACAATAAAAGTAATCTTCTGCGTTCTTTACATACATAGGTAATCATCTAA

TTTGTGTATATGAAATTAAAATAGTACACAGATTTCAGAAATTTTAAGTAAAAATAATGG

TTCAAAAGAAGTCAATTGCATCACACATAATTATTTCGCCTTCGTCCATCTTGTTTACTT

TAAAACTTCTAAAAAGCGATTTTTAAGCGGAAACTCTGGCAAAGGCTAAGCTCGATTTCT

CGCCGCTAAAGCACTTGCCAATCTTTGCGCAAATGCTTGATTCATCAGTTTCGCCGCCCT

GTGCCAGATTCAATATGCTCTGGCCGTATGTTCTAATCATGTGGTTACAAATATCGTAAT

ATTTGGCATCGAGGACTTGACAGCCCTCCAAAAGCATATAGTACTCCGTAATGGACTCGT

GATCCAAATGTTGCTTGAAGTATGGCAGCAGAGCAGAGGTGGCGCCATGACAAACGCCGC

ATTCAATGACCTCATCGCTAACCACATCCAGTCCCGCGAAACACAACTGCATTTGTTGAC

ACATCATTTTGGGTGGTACGCTGCCGATCAACGCTAAAATCGTGCTGGTATATTGATCGA

TGAATTTGTTGCAGCTTTTAGTAACAGTTTTCGGCATAATTTTGCATATATTCTCAACAG

TGTGTTTGATTTCCTCCTGTGTCGATGCGTTCTTTATTTCGGATTCTAGTTTAGTCATGA

CAAATTCACATAGCACACAAGTGGGTGGATCGTTCACCATATCATCCATGCCGACAAAAC

GATCGTTGTGTGGCGCATATTTTTGACCGGGCAATACAACAACATCATACTTCAATGCCT

CATCGATATCCAGATCCTCTTGTTCGCTCCAAATGCACAAACCGATCTCGCGGCAAATCA

TTTTCGGTGCCATTTCCTTAACCAACAAATCGGCGATTTTATCCCCATATTTAGCTATGT

ATTTATGGCATTTATCACGTAAATTTTTGCGAAGTTTATCGCAAGAATGATCCAGGGACT

TCTTAATTTCATCCTTGGTTGTGTGCTTGCCAATTCTTTTCTCGGCAATCTTAATGACCT

CCTCACATAACAAACAATTGGGTGGTTCAATGCGTCCAAATGCAAGTTCCACGGAATCAT

TGCTATCGATTTCATTTACTCCTTCTTCGCGGCTATTGCTTTCCAAACTAATGCCCAACT

CATTCAGCTCATCGCTTGAGTTTTGGCATAGTTTTATAGCAACACAGATCTCTTGTGGCT

TAAAATCGGTGATCAACATGTCGATGAGTTCGTTCATATAAGTTTCAACAAAATCAACAC

ATTCGGATTTTAATTTCGACGGTAAATGTGCACACAAATTATCGAGCACATTCTTGATGT

TTGCCTTTGACTTGTTGTCCTTAATCTTTTCTTGCGCTTGTTCGACAGCAAACAGGCAAA

GTGGGCATGTGGGTTTATCGCTTTCATCAACGTGTTGCATTTCGCTAACTGAAGGCTTAG

AAATTTTCGGCGCAAATATCTCTACGTCATCGAAATTTTCGTGATTGGCTGGGCACATTT

TCATTTTAGGACAGACTTCACGTGGATTGAGACCTTGAACGAGGAGCGCAATCACAGCAT

CGCCGTACATATCAAGGAAATTGTGGCATTCGCCACGAAGTGTGCGACCCAAACGGCTGC

AAATGTTATTAACGACATCCTTGATTTTGTCCTCTGTGGTTGACGTCGACAATTCGACTT

GAATATAATGCAGCACATATTCACAGAATGTGCATAGCTCTCCGTTATCGACCAGCAGTC

CAGGATTGGCTGCACCCATCAGTGTATCAATTGGCAAGGTCATAGCATGTATCTCCTCCT

GTGTGAATTTCGGTTCGTTGGCACCCAATTTACGTAGTGTCACTTTGATTTCAGCTGGTT

GCACGGAAGGCAACAATGGCCTGATTTCACCTTTGAACGCCTCAGCATTACCTTTCGGGC

ACACACCAATCATAAAGCAAGCACCATTAGCATCCAAATTGTTTACCAATGCATTATAGA

TAGCATCATAGTACTGCTCCACCAAACTGTTGCATTCCTCGCGGAAACCTTTGGTTTGAT

TGCACAAGCCATGCAATACGTCTTTAAATTCAGATTCGGTTGTATTGGCAATCAACACGT

CACGTAAATGTTGTACTAATTGTTCGCACAATTTGCATGGTATATCATCGCCCAAACTGG

ATGCGAGGGCAACAGCATCTTCTGGCTCTGTGGGATCTTCGGCGTGTTTGTGATAATTGG

CAGCGCACGAACCCGACATGTGGCATATGCCAGCAGCATTCAAGTGCTCCTTCATGTGAT

TATAAATATCATTGAAATAAGTAAGTACAATATTGGAACAGGCATCAGAGAAGGACGACA

TTTCACCGCAGAGATGCAAAATATTTTCGAGAATTTCATCGCGATTGGCTTTGTTAAATT

TCTCTGTAATAAGTGAGCCCAAATGGTTGCAATTGCTGCAAGTAAGCAGGTGCTGCTGTT

GGGGAGCTATAGCATCCACTTCTTTTCTTTCCTTTGACTCATCGGATTTCTCCTCCGCCA

ATGTGCCATCCAAAGCCGATTGATAGTAGAATTGCAACAATTCATCGATACGCGCATTAT

TGCAAAGGCCAGCAACCGAACAAACTTGATCTGGATTCATTTCGGAAGAGAGTGCCTCAA

CCAATTCAGGCACAAAGTCATCAGCCAACTTGGTGCATTCCTTCTTTACAATTTTGATCG

GTATGAGAGCACAAGAGCCTTCGAACACTTCCTTCAGCTCTTCCATAGTTTCATTACTAC

GCAATTGGTCGCGTGCCTGTGTTACCATATCTTTGCAAATCTTACAGATAGAATCGTCGT

CCACGGGTACATTTTGAGTCTCCCACACAGTTTGAATGCAGTGACGTACAGCATGGCAAC

CTTTTGCATTTGTTAGATTTTTACACCAGAAACTGGGTCCCCATGTGCATTGTTTAGCTC

CTAACATTGGCGTTGCACTCACAAAGAGTGTACCAGCCAATAGCGCGATTGCAGCGCAAA

GTACAAAGCTGCGTTGCATTTTGCTGAAAATCCCTATACACCACTTTGTATCACTACTAA

TTTTCCTCGACAATCAATTACACTAATTTGAACAAACAAAATCCAATTTTCTAAAATACA

ATAAAATGGCAAGTTTACTAGAGTATTTTTTTCACAATAAGAACCGTGCAACACAAATGA

T

**tBLASTn(First hit)**

Score = 1021 bits (2640), Expect = 0.0, Method: Compositional matrix adjust.

Identities = 521/903 (58%), Positives = 667/903 (74%), Gaps = 49/903 (5%)

Frame = -1

Query 1 MVTQARDQLKSNQTEEELKEVFEGSCKLIPIKPIQKECIKVADDFLPELVEALASQMNPD 60

MVTQARDQL+SN+T EELKEVFEGSC LIPIK ++KEC K+ADDF+PELVEAL+S+MNPD

Sbjct 3268 MVTQARDQLRSNETMEELKEVFEGSCALIPIKIVKKECTKLADDFVPELVEALSSEMNPD 3089

Query 61 QVCSVAGLCNSARIDELYKNGIQAGLDGTVQNE--DDSSEETEL-AMQPNQ---LSCGNC 114

QVCSVAGLCN+ARIDEL + Q+ LDGT+ E D+S E E+ A+ P Q L+C NC

Sbjct 3088 QVCSVAGLCNNARIDELLQFYYQSALDGTLAEEKSDESKERKEVDAIAPQQQHLLTCSNC 2909

Query 115 NLLSRLMHSKFAATDRDDMVETMLHMCGSLSSFSDACANIVLTYFNDIYDHVSKHLTTDA 174

N L L+ KF +RD+++E +LH+CG +SSFSDAC+NIVLTYFNDIY+H+ +HL

Sbjct 2908 NHLGSLITEKFNKANRDEILENILHLCGEMSSFSDACSNIVLTYFNDIYNHMKEHLNAAG 2729

Query 175 VCHVSGVCASRYHQHEEEKQPQEALVALDA--GDDIPCELCEQLVKHLRDVLVANTTETE 232

+CH+SG CA+ YH+H E+ E VAL + GDDIPC+LCEQLV+HLRDVL+ANTTE+E

Sbjct 2728 ICHMSGSCAANYHKHAEDPTEPEDAVALASSLGDDIPCKLCEQLVQHLRDVLIANTTESE 2549

Query 233 FKQVMEGFCKQSKGFKDECLSIVDQYYHVIYETLVSKLDANGACCMIGICQKNSASSMKD 292

FK V+ G C Q+KGF++EC S+V+QYY IY LV+ LDANGAC MIG+C K +A + K

Sbjct 2548 FKDVLHGLCNQTKGFREECNSLVEQYYDAIYNALVNNLDANGACFMIGVCPKGNAEAFKG 2369

Query 293 VPIMPLLPVIEPAQVKITIEKLEKHEKKQLGASEPKFSQQEILDMQLPIDHLMGAANPGA 352

I PLLP ++PA++K+T+ KL GA+EPKF+Q+EI M LPID LMGAANPG

Sbjct 2368 -EIRPLLPSVQPAEIKVTLRKL--------GANEPKFTQEEIHAMTLPIDTLMGAANPGL 2216

Query 353 LVEGGELCTLCEYMLHFIQETLATPSTDDEIKHTVENICAKLPSGVAGQCRNFVEMYGDA 412

LV+ GELCT CEY+LH+IQ L+T +T+D+IK V NIC++L + G+C NF++MYGDA

Sbjct 2215 LVDNGELCTFCEYVLHYIQVELSTSTTEDKIKDVVNNICSRLGRTLRGECHNFLDMYGDA 2036

Query 413 VIALLVQGLNPRDVCPLMQMCPKNLPKKEDVEVFNPQPAS---------DEQDPPTCPLC 463

VIALLVQGLNPR+VCP M+MCP N +DVE+F P+ + DE D PTCPLC

Sbjct 2035 VIALLVQGLNPREVCPKMKMCPANHENFDDVEIFAPKISKPSVSEMQHVDESDKPTCPLC 1856

Query 464 LFAVEQAQMKIRDNKSKDNIKKVLNGLCSHLPNEIKEECVDFVNTYSNELIDMLITDFKP 523

LFAVEQAQ KI+DNKSK NIK VL+ LC+HLP+++K ECVDFV TY NELIDMLITDFKP

Sbjct 1855 LFAVEQAQEKIKDNKSKANIKNVLDNLCAHLPSKLKSECVDFVETYMNELIDMLITDFKP 1676

Query 524 QEICVQLKLCPKTTYALWDLRISLEDDVDGEDKSSSEEISFNDIESLEELPPQLAFDPGF 583

QEICV +KLC ++ L +L ISLE S+S E N+I+S + + +LAF G

Sbjct 1675 QEICVAIKLCQNSSDELNELGISLE--------SNSREEGVNEIDSNDSV--ELAF--GR 1532

Query 584 TAAPNCLICEELVKTLEKRMGKHPTRDSIKHILEESCDRMRKPMNTKCHKVIDKYGDKIA 643

PNCL+CEE++K EKR+GKH T+D IK L+ SCD++RK + KCHK I KYGDKIA

Sbjct 1531 IEPPNCLLCEEVIKIAEKRIGKHTTKDEIKKSLDHSCDKLRKNLRDKCHKYIAKYGDKIA 1352

Query 644 DLLLKEMDPKLICTELGMCIladlddlevdealKYDVIALPRQDNK--------LSSSIK 695

DLL+KEM PK+IC E+G+CI ++ +DL++DEALKYDV+ LP Q + +

Sbjct 1351 DLLVKEMAPKMICREIGLCIWSEQEDLDIDEALKYDVVVLPGQKYAPHNDRFVGMDDMVN 1172

Query 696 EPPTCVLCEFIMTKLDADLKNKTEQDDIKRAIEAVCNRLPATVRKQCDTFVDGYASAVLK 755

+PPTCVLCEF+MTKL++++KN + Q++IK +E +C +P TV K C+ F+D Y S +L

Sbjct 1171 DPPTCVLCEFVMTKLESEIKNASTQEEIKHTVENICKIMPKTVTKSCNKFIDQYTSTILA 992

Query 756 LLSDVPPKQVCQKLQLCFSV--AVTDEVLECGVCHGVTQALLPFLREKKDNVSEVTALQM 813

L+ VPPK +CQ++QLCF+ V+DEV+ECGVCHG T ALLP+ ++ D+ S +T M

Sbjct 991 LIGSVPPKMMCQQMQLCFAGLDVVSDEVIECGVCHGATSALLPYFKQHLDHES-ITEYYM 815

Query 814 TSVGCENLPAKYYKICSEMISIYGSSIKNLAKRPYIDQSHICAEIGKCFESEKSSLAFAR 873

GC+ L AKYY IC+ MI YG SI NLA+ D+S ICA+IGKCF EKSSLAFAR

Sbjct 814 LLEGCQVLDAKYYDICNHMIRTYGQSILNLAQGGETDESSICAKIGKCFSGEKSSLAFAR 635

Query 874 ISA 876

+SA

Sbjct 634 VSA 626

**Conserved domains**

**SapA and SapB domain-containing protein**


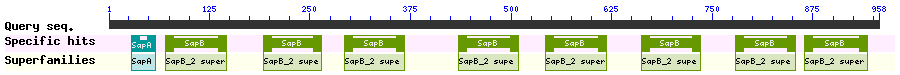


**V-ATPase V0-domain**

**>TRINITY_DN27448_c0_g3_i1 len=729**

CTTTTCGTTTAGTTTGTTTTATTATTTACAAATTGAATTGTTGTGCATATTCGTTCAGGC

ACAAAAATGCTTTAGAAAATGCAGTTTTAATTCAAGAAAAGAAAATTTTAAAGCAAACTA

GGGAATTCTTATATTCATTGTTATAAAAATTTTTTATCTTTGCATTACGATTAAGGTGTG

CTTATTGTAATACCTAAATGACGTCACATGCTCATCAGCGCAACTAAATCACCGTAACAC

CATTTTTTCACGATCGATCACTTAATTAGTTTTTTTGTTGCTAACTCAATCAATTTTTTT

GAATGATCCTTCCTCATGTTGTGCTCCACCAAAATATGCACGATAAATGTACAGGCCCAG

CGCTAGATGTAGTGCTACAATTGCGCCCACAGCTGAATAAATGTTGGTCATCATCTCGGA

CATAGTAAAGAATCGGTCCAGCACCGTAGACTTAAGTAAGAAGAATGTGGCGACAGGCAG

GAAAACAATTAACGAAGAATAGAAGAAGACCACTTTGAATGCGCTGTAATCTTTTGAATC

ATTTATTTGTTGCTGTGTGCGACTGTTTGTATTATTACCCTTTTTGTTAGCCATTATTCG

CAGTAAACAATCCAATTTTGTATAATACCTGCACGTCTTTTTACAATAGAGGTAGTCAAA

TGTCTTTTAATGTTTTCTGTTTTCTCTTTTTTTCTTTATTCACAATTGTCAAAATGGGAA

GACGTCACG

**tBLASTn(First hit)**

Score = 115 bits (289, Expect = 6e-032, Method: Compositional matrix adjust. Identities = 61/99 (62%), Positives = 73/99 (74%), Gaps = 5/99 (5%

Query 5 NKKAAGGNGVAPKQTRQQSHDSQDYSSFKTVLFYCMLIVFLPVLTFFVLKGFVLDQFLDI 64

NKK N +T+QQ +DS+DYS+FK V FY LIVFLPV TFF+LK VLD+F +

Sbjct 588 NKKGNNTNS----RTQQQINDSKDYSAFKVVFFYSSLIVFLPVATFFLLKSTVLDRFFTM 421

Query 65 SEVKVNIASAVGAVVALHIALGLYIYRAYF-GAPGSKGS 102

SE+ NI SAVGA+VALH+ALGLYIYRAYF GA +GS

Sbjct 420 SEMMTNIYSAVGAIVALHLALGLYIYRAYFGGAQHEEGS 304

**Conserved domains**

**VMA21 domain-containing protein**


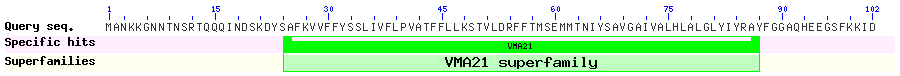

Supplement: Supplementary file 2 [file Table_2.DOCX]
