# Supplementary material for: The South American Fruit Fly: An Important Pest Insect With RNAi-Sensitive Larval Stages
Source: Front Physiol. 2019 Jun 27;10:794. doi: 10.3389/fphys.2019.00794 (PMC6610499; doi:10.3389/fphys.2019.00794)
Supplement: Supplementary file 3 [file Table_3.DOCX]

Supplementary Material

Table S1. Primers used in the South American fruit fly bioassays

| Gene | Primer name | Primer sequence (5’ to 3’) | Product size (pb) |
| --- | --- | --- | --- |
| *V-ATPase* | dsvtp_F | TAATACGACTCACTATAGGGAGATGCATATTCGTTCAGGCACA | 483 |
|  | dsvtp_R | TAATACGACTCACTATAGGGAGACAGCGCATTCAAAGTGGTCT |  |
|  | vtp_F | CCTTCCTCATGTTGTGCTCC | 219 |
|  | vtp_R | CAGCGCATTCAAAGTGGTCT |  |
| *GFP* | dsgfp_F | TAATACGACTCACTATAGGGAGATCGTGACCACCCTGACCTAC | 560 |
|  | dsgfp_R | TAATACGACTCACTATAGGGAGATCGTCCATGCCGAGAGTGAT |  |
| *Actin* | act_F | TACACTGGAACTAACGCGGT | 212 |
|  | act_R | GTCGAACCACCACTCAACAC |  |
| *α-Tubulin* | tub_F | CGAGGCCTCAAACATGATGG | 155 |
|  | tub_R | GGCACCAGTCCACAAATTGT |  |
| *Dicer 2* | dcr2_F | CCGTAGCACTTTCGTTAGA | 122 |
|  | dcr2_R | GGCCGATATTCGTTGTTTG |  |
| *Argonaute 2* | ago2_F | GCAGAGACAGACTCCTATTC | 118 |
|  | ago2_R | GCTTCTTTGGGACGTAGAT |  |

The T7 RNA polymerase promoter is underlined.

Table S2. Overview of the Illumina sequencing and *de novo* assembly statistics of the life stages of *Anastrepha fraterculus*

| Total of paired-end reads | 103,808,135 |
| --- | --- |
| Total of contigs | 84,105 |
| Total of transcripts | 163,359 |
| GC (%) | 38,82 |
| Contig N50 | 1,898 |
| Average contig length (bp) | 956.50 |
| Median contig length (bp) | 448.00 |
| Total assembled bases | 156,252,865 |


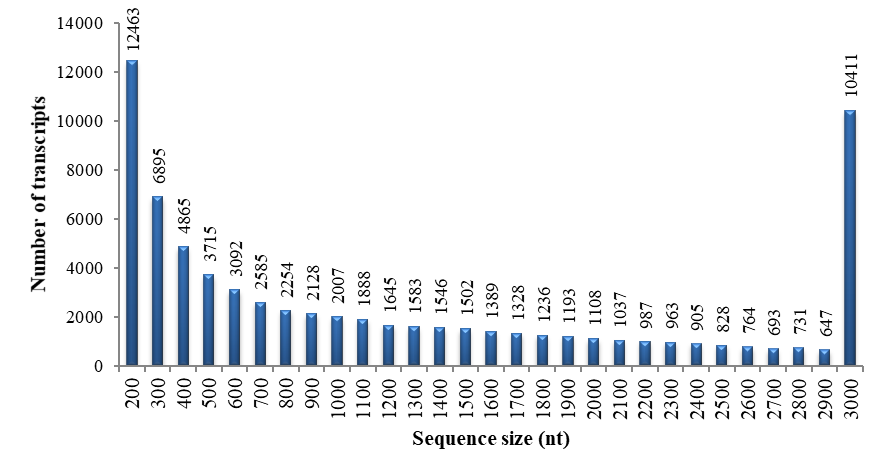


Figure S1. Length distribution of contigs in *Anastrepha fraterculus* transcriptome (only contigs of Eukaryote).


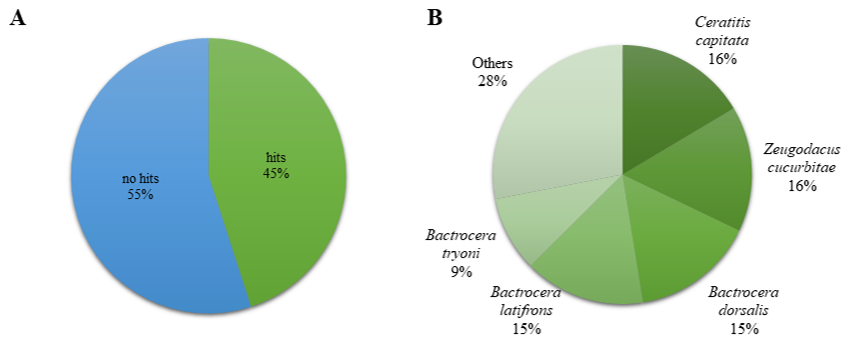


Figure S2. Distribution of Diamond similarity search. A) Distribution of the total hits against the UniProt-trEMBL database. B) Sequence comparison to insect species from the distribution of Diamond hits (E-value 1e-10).

Table S3. Species distribution of top 30 hits in Diamond searches (e-value 1e-10) of the data against the UniProt-trEMBL database.

| Top30 | Species | hits | (%) |
| --- | --- | --- | --- |
| 1 | *Ceratitis capitata* | 12,050 | 16.46 |
| 2 | *Zeugodacus cucurbitae* | 11,463 | 15.66 |
| 3 | *Bactrocera dorsalis* | 11,226 | 15.34 |
| 4 | *Bactrocera latifrons* | 11,044 | 15.09 |
| 5 | *Bactrocera tryoni* | 6,883 | 9.40 |
| 6 | *Tabanus bromius* | 1,240 | 1.69 |
| 7 | *Lasius niger* | 1,141 | 1.56 |
| 8 | *Acyrthosiphon pisum* | 999 | 1.36 |
| 9 | *Acromyrmex echinatior* | 692 | 0.95 |
| 10 | *Lucilia cuprina* | 578 | 0.79 |
| 11 | *Musca domestica* | 503 | 0.69 |
| 12 | *Lygus hesperus* | 491 | 0.67 |
| 13 | *Harpegnathos saltator* | 487 | 0.67 |
| 14 | *Drosophila ananassae* | 450 | 0.61 |
| 15 | *Corethrella appendiculata* | 445 | 0.61 |
| 16 | *Stomoxys calcitrans* | 437 | 0.60 |
| 17 | *Drosophila subobscura* | 391 | 0.53 |
| 18 | *Bombyx mori* | 387 | 0.53 |
| 19 | *Dufourea novaeangliae* | 365 | 0.50 |
| 20 | *Camponotus floridanus* | 347 | 0.47 |
| 21 | *Nasonia vitripennis* | 346 | 0.47 |
| 22 | *Drosophila melanogaster* | 327 | 0.45 |
| 23 | *Fopius arisanus* | 324 | 0.44 |
| 24 | *Cuerna arida* | 277 | 0.38 |
| 25 | *Lepeophtheirus salmonis* | 262 | 0.36 |
| 26 | *Rhodnius prolixus* | 243 | 0.33 |
| 27 | *Trachymyrmex zeteki* | 241 | 0.33 |
| 28 | *Trachymyrmex septentrionalis* | 235 | 0.32 |
| 29 | *Homalodisca liturata* | 229 | 0.31 |
| 30 | *Trachymyrmex cornetzi* | 226 | 0.31 |

**Table S4. Number accession of sequences used in phylogenetic analysis**

| **Number accession** | **Species** |
| --- | --- |
| **Dicer-2** |  |
| TRINITY_DN32516_c1_g2_i1 | *Anastrepha fraterculus* |
| ABB54747.1 | *Drosophila melanogaster* |
| NP_001107840 | *Tribolium castaneum* |
| AUM60046.1 | *Diabrotica virgifera virgifera* |
| K7J5H5 | *Nasonia vitripennis* |
| A0A172M4U9 | *Bombus lapidarius* |
| NP_001180543.1 | *Bombyx mori* |
| OWR42902.1 | *Danaus plexippus plexippus* |
| CCF23094.1 | *Blattella germanica* |
| AJF15703.1 | *Agrilus planipennis* |
| **Argonaute-2** |  |
| TRINITY_DN30039_c4_g1_i5 | *Anastrepha fraterculus* |
| ADQ27048.1 | *Drosophila melanogaster* |
| NP_001107828 | *Tribolium castaneum* |
| AUM60042.1 | *Diabrotica virgifera virgifera* |
| XP_395048.4 | *Apis melífera* |
| XP_008214882.1 | *Nasonia vitripennis* |
| NP_001036995 | *Bombyx mori* |
| EHJ72821.1 | *Danaus plexippus plexippus* |
| XP_024214272.1 | *Halyomorpha halys* |
| **V-ATPase** |  |
| TRINITY_DN27448_c0_g3_i1 | *Anastrepha fraterculus* |
| XP_011205737.1 | *Bactrocera dorsalis* |
| NP_788549.1 | *Drosophila melanogaster* |
| XP_016934184.1 | *Drosophila suzukii* |
| XP_015834455.1 | *Tribolium castaneum* |
| XP_023015994.1 | *Leptinotarsa decemlineata* |
| XP_001120244.1 | *Apis mellifera* |
| XP_011304607.1 | *Fopius arisanus* |
| NP_011619.3 | *Saccharomyces cerevisiae* |
| XP_453740.2 | *Kluyveromyces lactis* |
| NP_001017980.1 | *Homo sapiens* |
| NP_001074825.1 | *Mus musculus* |
| XP_003710030.1 | *Pyricularia oryzae* |
| XP_001586304.1 | *Sclerotinia sclerotiorum* |
| NP_565728.1 | *Arabidopsis thaliana* |
| XP_015635612.1 | *Oryza sativa* subsp. *japonica* |
| XP_007212280.1 | *Prunus persica* |


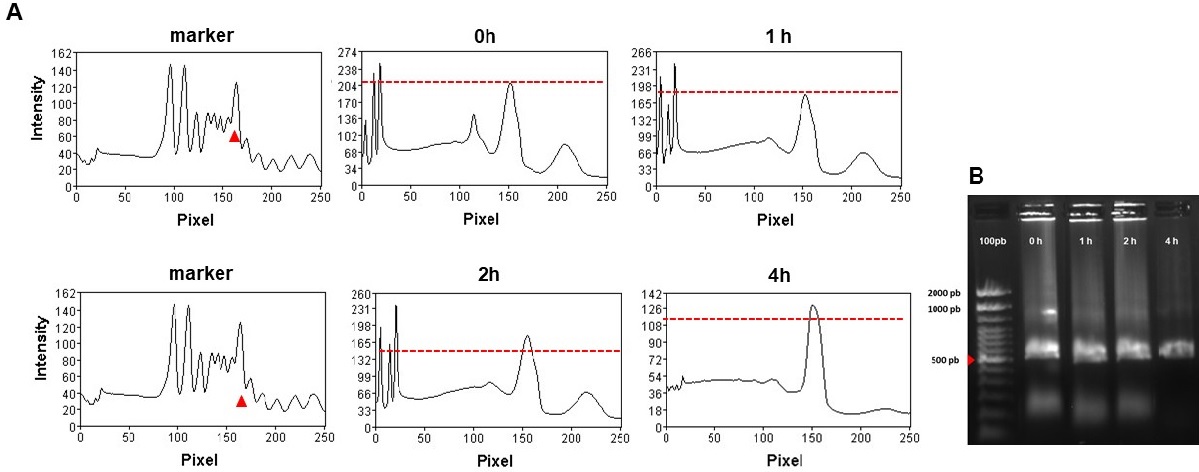


**Figure S3.** **dsRNA degradation assay. The peak at 150 pixels (∆) indicate the band intensity of the dsRNA when incubated(A). Agarose gel image show the dsRNA (500 pb) degradation (B). The triangle (∆) indicate the fragment size of the ds*GFP*. Incubation of 20 µl (500 ng) ds*GFP* with 2 µl of body fluid from *Anastrepha fraterculus* larvae. Aliquots were removed at the times indicated. The samples were visualized by electrophoresis on a 1.5% agarose gel and analyzed using the Gel Analyzer software. Marker used was 100 pb.**
