# Supplementary material for: The South American Fruit Fly: An Important Pest Insect With RNAi-Sensitive Larval Stages
Source: Front Physiol. 2019 Jun 27;10:794. doi: 10.3389/fphys.2019.00794 (PMC6610499; doi:10.3389/fphys.2019.00794)
Supplement: Supplementary file 4 [file Table_4.DOCX]

Supplementary Material

**BLASTp for identify confirm of machinery genes**

**Sequences of *Anastrepha fraterculus* transcriptome**

**Dicer-1**

**>lcl|ORF40**

MSFHWCDNIHTTIFTPRDYQIELLSAAFERNVMICLGHKSSKEFIALKLL

QELARPARIAEKINLYLTCEQNAPSITTMIEHLTDLKVYKEERTGAADKQ

EIHIPKDFQLYVLHPRTCLAALRCGKLSMTNVHLLILEDCHEAEVYRDLR

QIFQDHFNVSGKDMPKVLGLAGPLHSAECSLEELSAMLNTLEDSVHCKAE

TASDIVTVLRYCAKPSEYIVQCTPYPRDELTAVLEEVICTRKAFLIDHRY

DPFEIYCTEEYMEELKDIPDPKEEPMEFLEIMLFVLYEMGPWCADRVAVN

MFHRIEKQKIKTPHERHYILLCVVNTALLEFHAICEQTFKKFNNHKELVE

RYSSPKVFRLLEILRLFKPEELLTKNETIQKMSQDLDQMDFQKLSRSIEY

SCQNVENVTTKLQLESRSIMDNLDPIVKSPTTDVAKQQTLQANTTHKTES

SASTNRRHGVGGSHGRFKRRPFNRRQMRDNGDALDTLCAIIFCNSNYTAR

VLFDLLSEMSRHDPELKFLKCQFTADRVADPITEPKEAEVEHRRQEEVLK

RFRMHDCNVLIGTSVLEEGIDLPKCNLVVRWDAPTTYRSYVQCKGRARAA

PAYHVILVSPAYNAILCENEQLSDQSHRYLCKLEVGQDDVIDTDSCSDDE

EECNGKSNKFTIGSSKGIVKILNPEVITSKPPTKCEITLKEIKDELPAIK

GIENEDTNTIDLVEIESELAPITELQNETSSIKEVSKTKSVANESLHDCF

DVLDLSDMISDISSNNSSRILLEETATTPASASVTPKQKKEKRRFRCICE

NENCAQHGLETGLDATINQIEFTTNKIVEQLAEYREIEKMLLQKCANTEP

SEAEHQLADYFNKCVKRYKPCEHLLTGASVDLTSSIALVNKYCSKLPSDT

FTKLTALWRCTKTIRCGVEMYQYTIRLPINSPLKYDIVGLPMSSNILARR

MAALQACIELHKTGELDDNLLPIGKEGFRAMEPDWENFDLEEEDDKIVLE

NSEPRPGTTKRRQYYYKRIASEFSDCRPTAGVPCYLYLIDLTLQCPIPEE

QNTRGRKIYPPEDAQQGFGILTLKRIPKVSSFPIFTRSGEVKVSIVLAKE

RVVLTETQIRCINTFLNYTFTNVLRLQKFLMLFDPDSTENCVFIVPTVKR

DGGKVIDWDFLELIERNASMMPTPVPEDMRKSIDFDANKFKDAVVMPWYR

NQDQPQYFYVAEICPQLSPQSCFPGENYRTFKHYYFLKYGLTIQNVKQPL

LDVDHTSARLNFLTPRYVNRKGVALPTSSEETKRAKRENLEQKQILVPEL

CTVHPFPASLWRTAVCLPCILYRINGLLLADDIRKKVSLDIGLGQQEIPD

DFEWPMLDFGWSLSEVLKRSKENNASLDKKTEEQKEKEANNKQSNGFAKD

IGVNGEFCAEDNQTKAKSANEIIIEGEKKLKDSNFIEIGTWSNDMANDIN

FNKYSDSLDDDEEECLKYFPSNVSFCDQQMRYGSPTFWDVKESQLNKKPD

DCAQSKNTTETNNFRKNSFAYYDSDDSFASSYDGNEPTNYSEDEDDECGP

LRIAFTSRNEAETIETDQEIEKRNKKLSIIQATNANERNYQKTKNLLVGY

NFEDLKPNIEAELCDSIAKFTASTRLLKANIEQSGMLVKYNQPVMLARKE

RTTNESAAHGEAATTEAFLKLLPYADKDVLMSILSKQANVLTIADIARLN

ADYLKSHPLELFEIQGCGDIFDNFNDKDMLKLEGWGEGYKKLIKLNFERK

AGATGCVSLIDVKQESKNMQGNTFSFDRQPDLAGHPGPSPSIILQGLTMS

NANDGINLERLETIGDSFLKYAITTYLYITYENVHEGKLSHLRSKQVANL

NLYRLGRRKKLGEYMIATKFEPHDNWLPPCYYVPKELEKELIEAKIPPHH

WKLVDLANIKKLNSAEICALVREKADKLGLFGDEYNCSEDASAVDEIAEG

NDFSCFIPYNLVTQHSIPDKSVADCVEALIGAYLIECGPRGALLFMAWLG

IRVLPFVRKPYNPQEPRIPGSSNPDVNGEITVYGEWTAPKSPLLHYSPNA

PQELESMLEGFDEFENTLGYKFRDRSYLLQAMTHASYSPNRLTDCYQRLE

FLGDAVLDYLITRHLYEDPRQHSPGALTDLRSALVNNTIFASLAVRHGFH

KYFRHLSPGLNEVIDRFVRIQNENGHSISEEYYLLSEEECDDAEDVEVPK

ALGDVFESIAGAIFLDSNMSLDVVWQVYSNMMKPEIEQFSNSVPKSPIRE

LLELEPETAKFGKPEKLADGRRVRVTVDVFCKGTFRGIGRNYRIAKCTAA

KCALRQLKKQGLIKKD

**Graphical representation - ORF**


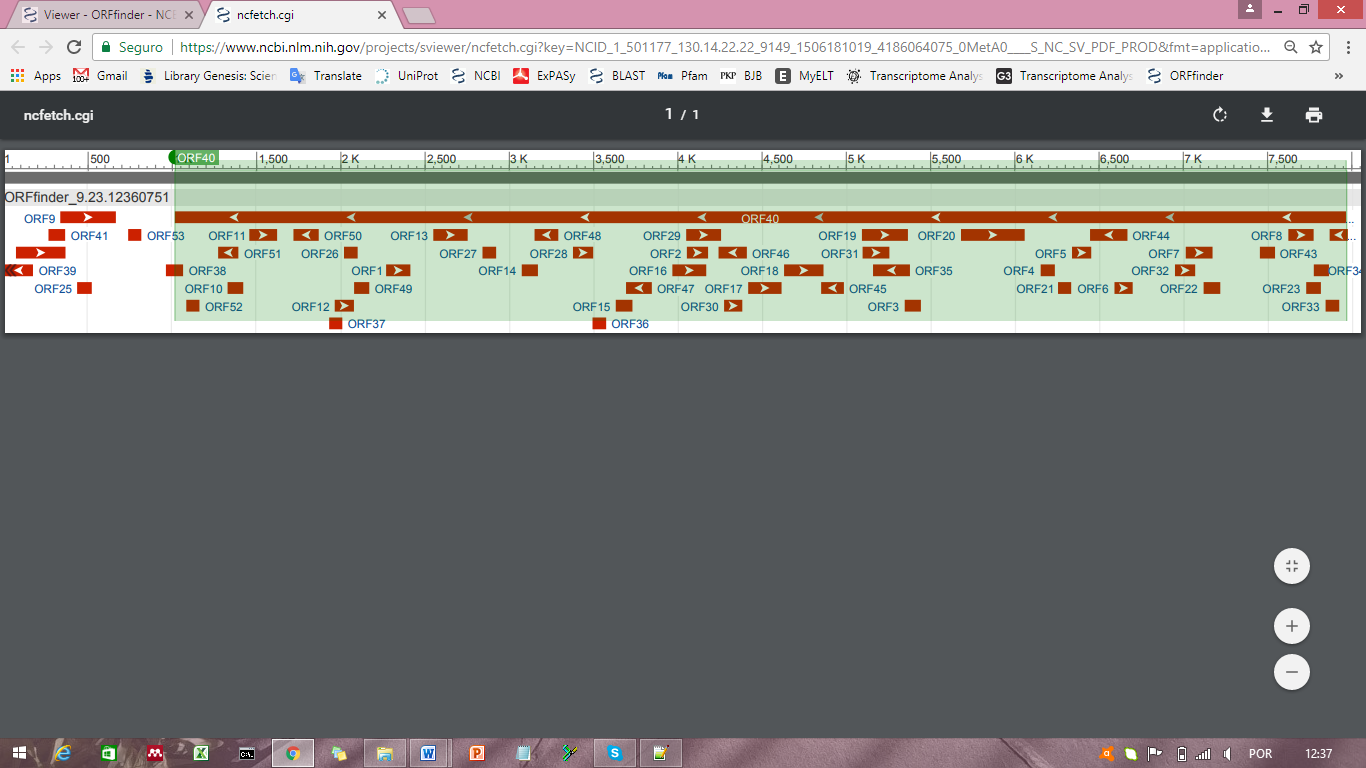


**BlastP (Non-redundant NCBI sequences)**

PREDICTED: endoribonuclease Dcr-1 [Rhagoletis zephyria]

Sequence ID: [XP_017475087.1](https://www.ncbi.nlm.nih.gov/protein/1048028689?report=genbank&log$=protalign&blast_rank=1&RID=WD00JS22014) Length: 2316 Number of Matches: 1

Range 1: 1 to 2314

Score: 4078 bits(10577)

E-value: 0.0

Identities: 1986/2324(85%)

Query 1 MSFHWCDNIHTTIFTPRDYQIELLSAAFERNVMICLGHKSSKEFIALKLLQELARPARIA 60

MSFHWCDNIHTTIFTPRDYQIELLSAAFERNVMICLGHKSSKEFIALKLLQELAR ARIA

Sbjct 1 MSFHWCDNIHTTIFTPRDYQIELLSAAFERNVMICLGHKSSKEFIALKLLQELARQARIA 60

Query 61 EKINLYLTCEQNAPSITTMIEHLTDLKVYKEERTGAADKQEIHIPKDFQLYVLHPRTCLA 120

KINLYLTCEQN PSITTMIEHLTDLKV+KEE +GA DKQ +HIP FQLYVLHPR+CL

Sbjct 61 GKINLYLTCEQNTPSITTMIEHLTDLKVFKEE-SGAGDKQLVHIPHGFQLYVLHPRSCLE 119

Query 121 ALRCGKLSMTNVHLLILEDCHEAEVYRDLRQIFQDHFNVSGKDMPKVLGLAGPLHSAECS 180

A++CG LS+ NVHL+ILEDCHEA +YRDLRQIF + FN S DMPKVLGLAGPLHSAECS

Sbjct 120 AMKCGILSLKNVHLMILEDCHEAAMYRDLRQIFCEFFNGSSDDMPKVLGLAGPLHSAECS 179

Query 181 LEELSAMLNTLEDSVHCKAETASDIVTVLRYCAKPSEYIVQCTPYPRDELTAVLEEVICT 240

LEELSAMLNTLE+SVHCKAETASDIVTVLRYCAKPSEYIVQC PY RDELT +LEE+ICT

Sbjct 180 LEELSAMLNTLENSVHCKAETASDIVTVLRYCAKPSEYIVQCMPYERDELTVILEEIICT 239

Query 241 RKAFLIDHRYDPFEIYCTEEYMEELKDIPDPKEEPMEFLEIMLFVLYEMGPWCADRVAVN 300

RKAFLIDHRYDPFEIY T+EYMEELKDIPDPKEEPMEFLE MLFVLYEMGPWCADRVA+N

Sbjct 240 RKAFLIDHRYDPFEIYSTDEYMEELKDIPDPKEEPMEFLETMLFVLYEMGPWCADRVAIN 299

Query 301 MFHRIEKQKIKTPHERHYILLCVVNTALLEFHAICEQTFKKFNNHKELVERYSSPKVFRL 360

MFHRIEKQKIKTPHERHYILLCVVNTALLEFHAICE TFKK HKELVER+SSPKV RL

Sbjct 300 MFHRIEKQKIKTPHERHYILLCVVNTALLEFHAICEHTFKKLTTHKELVERFSSPKVSRL 359

Query 361 LEILRLFKPEELLTKNETIQKMSQDLDQMDFQKLSRSIEYSCQNVENVTTKLQLESRSIM 420

LE LR+FKPEEL+TKNETIQKMSQDLDQMDFQKLSRSIEYSC +VENVTTKLQ+ESRSI+

Sbjct 360 LETLRVFKPEELMTKNETIQKMSQDLDQMDFQKLSRSIEYSCHSVENVTTKLQIESRSII 419

Query 421 DNLDPIVKSPTTDVAKQQTLQANTTHKTESSASTNRRHGVGGSHGRFKRRPFNRRQMRDN 480

DNLDPIVKSP TD +QQTLQ+NT HK+E++ TNRRHG G SHGRFKRRPFNRR MRDN

Sbjct 420 DNLDPIVKSPPTDQTRQQTLQSNTAHKSENNTGTNRRHGAGSSHGRFKRRPFNRRLMRDN 479

Query 481 GDALDTLCAIIFCNSNYTARVLFDLLSEMSRHDPELKFLKCQFTADRVADPITEPKEAEV 540

D +DTLCAIIFCNSNYTARVLFDLLSEMSRHDPELKFLKCQFT DRVADPITEPKEAE+

Sbjct 480 SDTMDTLCAIIFCNSNYTARVLFDLLSEMSRHDPELKFLKCQFTTDRVADPITEPKEAEI 539

Query 541 EHRRQEEVLKRFRMHDCNVLIGTSVLEEGIDLPKCNLVVRWDAPTTYRSYVQCKGRARAA 600

EHRRQEEVLKRFRMHDCNVLIGTSVLEEGIDLPKCNLVVRWDAPTTYRSYVQCKGRARAA

Sbjct 540 EHRRQEEVLKRFRMHDCNVLIGTSVLEEGIDLPKCNLVVRWDAPTTYRSYVQCKGRARAA 599

Query 601 PAYHVILVSPAYNAILCENEQLSDQSHRYLCKLEVGQDDVIDTDSCSDDEEECNGKSNKF 660

PAY+VILVSPAY A+ C+NEQLSDQ H YLCK+E GQDD ID+DS ++DEE N NKF

Sbjct 600 PAYYVILVSPAYKAVHCKNEQLSDQCHGYLCKIEDGQDDGIDSDSGNEDEETSNDNGNKF 659

Query 661 TIGSSKGIVKILNPEVITSKPPTKCEITLKEIKDELPAIKGIENEDTNTIDLVEIESELA 720

TIGSSKGIVKILNPEVITSKPPTK EITLKEIKDELPAI+G E+E T+ +LVEIE EL

Sbjct 660 TIGSSKGIVKILNPEVITSKPPTKSEITLKEIKDELPAIEGTESETTDAFNLVEIEDELP 719

Query 721 PITELQNETSSIKEVSKTKSVANESLHDCFDVLDLSDMISDISS---NNSSRILLEETAT 777

ITEL+ ET SI+E+ K V + + C++VLDL+D ISDISS N++S LEET+T

Sbjct 720 SITELKYETPSIQEI---KLVPIKPIDICYEVLDLNDAISDISSVLNNSASSSFLEETST 776

Query 778 TPASASVTPKQKKEKRRFRCICENENCAQHGLETGLDATINQIEFTTNKIVEQLAEYREI 837

+ PK +K KRRF+C+CEN ++ ++TG+DA + QIE+TTNKIVEQLAEYREI

Sbjct 777 ---ATKAPPKAQKVKRRFQCVCENAKDSE-SMDTGVDAVVQQIEYTTNKIVEQLAEYREI 832

Query 838 EKMLLQKCANTEPSEAEHQLADYFNKCVKRYKPCEHLLTGASVDLTSSIALVNKYCSKLP 897

EKMLLQKCANTEPSE EHQ ADY+NKCV+RYKPCE+LLTGASVDLTSSIALVNKYCSKLP

Sbjct 833 EKMLLQKCANTEPSEMEHQFADYYNKCVRRYKPCEYLLTGASVDLTSSIALVNKYCSKLP 892

Query 898 SDTFTKLTALWRCTKTIRCGVEMYQYTIRLPINSPLKYDIVGLPMSSNILARRMAALQAC 957

SDTFTKLTALWRCTKT+RC E+YQYTIRLPINSPLKYDIVGLPMSSNILARRMAALQAC

Sbjct 893 SDTFTKLTALWRCTKTMRCDAEVYQYTIRLPINSPLKYDIVGLPMSSNILARRMAALQAC 952

Query 958 IELHKTGELDDNLLPIGKEGFRAMEPDWENFDLEEEDDKIVLENSEPRPGTTKRRQYYYK 1017

IELHKTGELDDNL PIGKEGFRA+EPDWENF+L EED+KIV ENSEPRPGTTKRRQYYYK

Sbjct 953 IELHKTGELDDNLQPIGKEGFRAVEPDWENFELAEEDEKIVHENSEPRPGTTKRRQYYYK 1012

Query 1018 RIASEFSDCRPTAGVPCYLYLIDLTLQCPIPEEQNTRGRKIYPPEDAQQGFGILTLKRIP 1077

RIASEFS+CRPTAGVPCYLY IDLTLQCPIPEEQNTRGRKIYPPEDAQQGFGILTLKRIP

Sbjct 1013 RIASEFSECRPTAGVPCYLYFIDLTLQCPIPEEQNTRGRKIYPPEDAQQGFGILTLKRIP 1072

Query 1078 KVSSFPIFTRSGEVKVSIVLAKERVVLTETQIRCINTFLNYTFTNVLRLQKFLMLFDPDS 1137

KVSSFPIFTRSGEVKVSIVL+K+RV+LTETQI CINTFLNYTFTNVLRLQKFLMLFDPDS

Sbjct 1073 KVSSFPIFTRSGEVKVSIVLSKDRVILTETQICCINTFLNYTFTNVLRLQKFLMLFDPDS 1132

Query 1138 TENCVFIVPTVKRDGGKVIDWDFLELIERNASMMPTPVPEDMRKSIDFDANKFKDAVVMP 1197

TENCVFIVPT+K+DGGK+IDWDFLELIERN+SMMPTPVP+++RK DFDANKFKDAVVMP

Sbjct 1133 TENCVFIVPTLKKDGGKLIDWDFLELIERNSSMMPTPVPDELRKCTDFDANKFKDAVVMP 1192

Query 1198 WYRNQDQPQYFYVAEICPQLSPQSCFPGENYRTFKHYYFLKYGLTIQNVKQPLLDVDHTS 1257

WYRNQDQPQYFYVAEICPQLSP SCFPGENYRTFKHYY LKYGLTIQNVKQPLLDVDHTS

Sbjct 1193 WYRNQDQPQYFYVAEICPQLSPLSCFPGENYRTFKHYYSLKYGLTIQNVKQPLLDVDHTS 1252

Query 1258 ARLNFLTPRYVNRKGVALPTSSEETKRAKRENLEQKQILVPELCTVHPFPASLWRTAVCL 1317

ARLNFLTPRYVNRKGVALPTSSEETKRAKRENLEQKQILVPELCTVHPFPASLWRTAVCL

Sbjct 1253 ARLNFLTPRYVNRKGVALPTSSEETKRAKRENLEQKQILVPELCTVHPFPASLWRTAVCL 1312

Query 1318 PCILYRINGLLLADDIRKKVSLDIGLGQQEIPDDFEWPMLDFGWSLSEVLKRSKEN-NAS 1376

PCILYRINGLLLADDIRKKVS+DIGLGQQ+I D+FEWPMLDFGWSLSEVLKRSKE+ NAS

Sbjct 1313 PCILYRINGLLLADDIRKKVSMDIGLGQQDIADNFEWPMLDFGWSLSEVLKRSKESKNAS 1372

Query 1377 LDKKTEEQKEKE-ANNKQSNGFAKDIGVNGEFCA---EDNQTKAKSANEIIIEGEKKLKD 1432

+ ++ ++ KE +N KQ+N F D G+NGE C E+NQ+KAKSANEIIIEGEKKL+D

Sbjct 1373 VGQENKDNNGKEESNGKQNNSFGTDNGINGE-CGNSVEENQSKAKSANEIIIEGEKKLQD 1431

Query 1433 SNFIEIGTWSNDMANDINFNKYSDSLDDDEEECLKYFPSNVSFCDQQMRYGSPTFWDVKE 1492

S FIEIGTWSNDMA+D+NF+K SDSL+DDEEECLKYFPSNVSFCDQQ+RYGSPTFWD++E

Sbjct 1432 STFIEIGTWSNDMADDLNFSKNSDSLEDDEEECLKYFPSNVSFCDQQLRYGSPTFWDLEE 1491

Query 1493 SQLNKKPDDCAQSKNTTETNNFRKNSFAYYDSDDSFASSYDGNEPTNYSEDEDDE-CGPL 1551

S+ NKK ++ Q+K N++RKNSFAYYDSDDS ASSYDGNEP YS+DEDD+ CGPL

Sbjct 1492 SEQNKKTNEGMQNKGNV-PNHYRKNSFAYYDSDDSLASSYDGNEPAKYSDDEDDDDCGPL 1550

Query 1552 RIAFTSRNEAETIETDQEIEKRNKKLSIIQATNANERNYQKTKNLLVGYNFEDLKPNIEA 1611

RIAFTS+NEAETIET QEIEK NK+LSIIQATNANERNYQKTKNLLVGYNF DLKP+IEA

Sbjct 1551 RIAFTSKNEAETIETVQEIEKHNKQLSIIQATNANERNYQKTKNLLVGYNFVDLKPSIEA 1610

Query 1612 ELCDSIAKFTASTRLLKANIEQSGMLVKYNQPVMLARKERTTNESAAHGEAATTEAFLKL 1671

EL +SIA+FTAST++L+ IE SGMLVKYNQPV LARKE++ S E T E F KL

Sbjct 1611 ELRESIAQFTASTKILRMTIEHSGMLVKYNQPVALARKEKSGGNSYVPDELPTAEMFFKL 1670

Query 1672 LPYADKDVLMSILSKQANVLTIADIARLNADYLKSHPLELFEIQGCGDIFDNFNDKDMLK 1731

LPYADK +LMSIL ++ N+LT ADIA LNADYLKSHPLE+FE+QGCGDIFDNFNDKDMLK

Sbjct 1671 LPYADKSLLMSILDERGNLLTPADIALLNADYLKSHPLEMFEVQGCGDIFDNFNDKDMLK 1730

Query 1732 LEGWGEGYKKLIKLNFERKAGATGCVSLIDVKQESKNMQGNTFSFDRQPDLAGHPGPSPS 1791

LE WGEGYKKLI+L F+RK A SL+ ++E K + + FSFDRQPDL GHPGPSPS

Sbjct 1731 LERWGEGYKKLIQLRFDRKVSAESNDSLMPAREEIKKVYSDLFSFDRQPDLTGHPGPSPS 1790

Query 1792 IILQGLTMSNANDGINLERLETIGDSFLKYAITTYLYITYENVHEGKLSHLRSKQVANLN 1851

IILQ LTMSNANDGINLERLETIGDSFLKYAITTYLYITYENVHEGKLSHLRSKQVANLN

Sbjct 1791 IILQALTMSNANDGINLERLETIGDSFLKYAITTYLYITYENVHEGKLSHLRSKQVANLN 1850

Query 1852 LYRLGRRKKLGEYMIATKFEPHDNWLPPCYYVPKELEKELIEAKIPPHHWKLVDLANIKK 1911

LYRLGRRKKLGEYMIATKFEPHDNWLPPCY+VPKELEK LIEAKIPPHHWKLVDL++IKK

Sbjct 1851 LYRLGRRKKLGEYMIATKFEPHDNWLPPCYFVPKELEKALIEAKIPPHHWKLVDLSSIKK 1910

Query 1912 LNSAEICALVREKADKLGLFGDEYNCSEDASAVDEIAEGNDFSCFIPYNLVTQHSIPDKS 1971

LNSAEICALVREKADKLGLF DE N SEDASAVDE EG DF+CFIPYNLVTQHSIPDKS

Sbjct 1911 LNSAEICALVREKADKLGLFSDESNYSEDASAVDESGEGKDFTCFIPYNLVTQHSIPDKS 1970

Query 1972 VADCVEALIGAYLIECGPRGALLFMAWLGIRVLPFVRKPYNPQEPRIPGSSNPDVNGEIT 2031

VADCVEALIGAYLIECGPRGALLFMAWLGIRVLPFVRKPYNPQ PRIPGS+ PD NGEIT

Sbjct 1971 VADCVEALIGAYLIECGPRGALLFMAWLGIRVLPFVRKPYNPQTPRIPGSTCPDDNGEIT 2030

Query 2032 VYGEWTAPKSPLLHYSPNAPQELESMLEGFDEFENTLGYKFRDRSYLLQAMTHASYSPNR 2091

VYGEW APKSPLLHYSPNA QELE MLEGFDEFE TLGYKFRDRSYLLQAMTHASYSPNR

Sbjct 2031 VYGEWAAPKSPLLHYSPNATQELEYMLEGFDEFEATLGYKFRDRSYLLQAMTHASYSPNR 2090

Query 2092 LTDCYQRLEFLGDAVLDYLITRHLYEDPRQHSPGALTDLRSALVNNTIFASLAVRHGFHK 2151

LTDCYQRLEFLGDAVLDYLITRHLYEDPRQHSPGALTDLRSALVNNTIFASLAVRHGFHK

Sbjct 2091 LTDCYQRLEFLGDAVLDYLITRHLYEDPRQHSPGALTDLRSALVNNTIFASLAVRHGFHK 2150

Query 2152 YFRHLSPGLNEVIDRFVRIQNENGHSISEEYYLLSEEECDDAEDVEVPKALGDVFESIAG 2211

YFRHLSPGLNEVIDRFVRIQNENGHSISEEYYLLSEEECDDAEDVEVPKALGDVFESIAG

Sbjct 2151 YFRHLSPGLNEVIDRFVRIQNENGHSISEEYYLLSEEECDDAEDVEVPKALGDVFESIAG 2210

Query 2212 AIFLDSNMSLDVVWQVYSNMMKPEIEQFSNSVPKSPIRELLELEPETAKFGKPEKLADGR 2271

AIFLDSNMSLDVVWQVYSNMMKPEIEQFSNSVPKSPIRELLELEPETAKFGKPEKLADGR

Sbjct 2211 AIFLDSNMSLDVVWQVYSNMMKPEIEQFSNSVPKSPIRELLELEPETAKFGKPEKLADGR 2270

Query 2272 RVRVTVDVFCKGTFRGIGRNYRIAKCTAAKCALRQLKKQGLIKK 2315

RVRVTV+VF KGTFRGIGRNYRIAKCTAAKCALRQLKKQGLI K

Sbjct 2271 RVRVTVEVFLKGTFRGIGRNYRIAKCTAAKCALRQLKKQGLITK 2314

**Argonaute-1**

**>lcl|ORF2**

MSTERELAPGGPAQLHTLPVAYSDLGATLQLNTAVRIIGKVYESQWTPSP

PRPQSPSQSQTNYDPHTSPPATGSSVNPTAVTSPSAQNVAVGGATVGGAG

ATGHSTQAAAVASPLGAAAATSPTTQPELPVFTCPRRPNLGREGRPIVLR

ANHFQVSMPRGYVHHYDINIQPDKCPRKVNREIIETMVHAYSKIFGVLKP

VFDGRNNLYTRDPLPIGNDRLELEVTLPGEGKDRIFRVTIKWMAQVSLFN

LEEALEGRTRQIPYDAILALDVVMRHLPSMTYTPVGRSFFSSPDGYYHPL

GGGREVWFGFHQSVRPSQWKMMLNIDVSATAFYKAQPVIDFMCEVLDIRD

INEQRKPLTDSQRVKFTKEIKGLKIEITHCGAMRRKYRVCNVTRRPAQMQ

SFPLQLENGQTVECTVAKYFLDKYRMKLRYPHLPCLQVGQEHKHTYLPLE

VCNIVAGQRCIKKLTDMQTSTMIKATARSAPDREREINNLVKRADFNNDS

YVQEFGLTISNSMMEVRGRVLPPPKLQYGGRVSSMTGQQLFPPQNKVSLA

SPNQGVWDMRGKQFFTGVEIRVWAIACFAPQRTVREDALRNFTQQLQKIS

NDAGMPIIGQPCFCKYATGPDQVEPMFRYLKNSFNALQLVVVVLPGKTPV

YAEVKRVGDTVLGMATQCVQAKNVNKTSPQTLSNLCLKINVKLGGINSIL

VPSIRPKVFNEPVIFLGADVTHPPAGDNKKPSIAAVVGSMDAHPSRYAAT

VRVQQHRQEIIQELSSMVRELLIMFYKSTGGYKPHRIILYRDGVSEGQFP

HVLQHELTAIREACIKLEAEYRPGITFIVVQKRHHTRLFCAEKKEQSGKS

GNIPAGTTVDVGITHPTEFDFYLCSHQGIQGTSRPSHYHVLWDDNHFDSD

ELQCLTYQLCHTYVRCTRSVSIPAPAYYAHLVAFRARYHLVEKEHDSGEG

SHQSGCSEDRTPGAMARAITVHADTKKVMYFA

**Graphical representation - ORF**


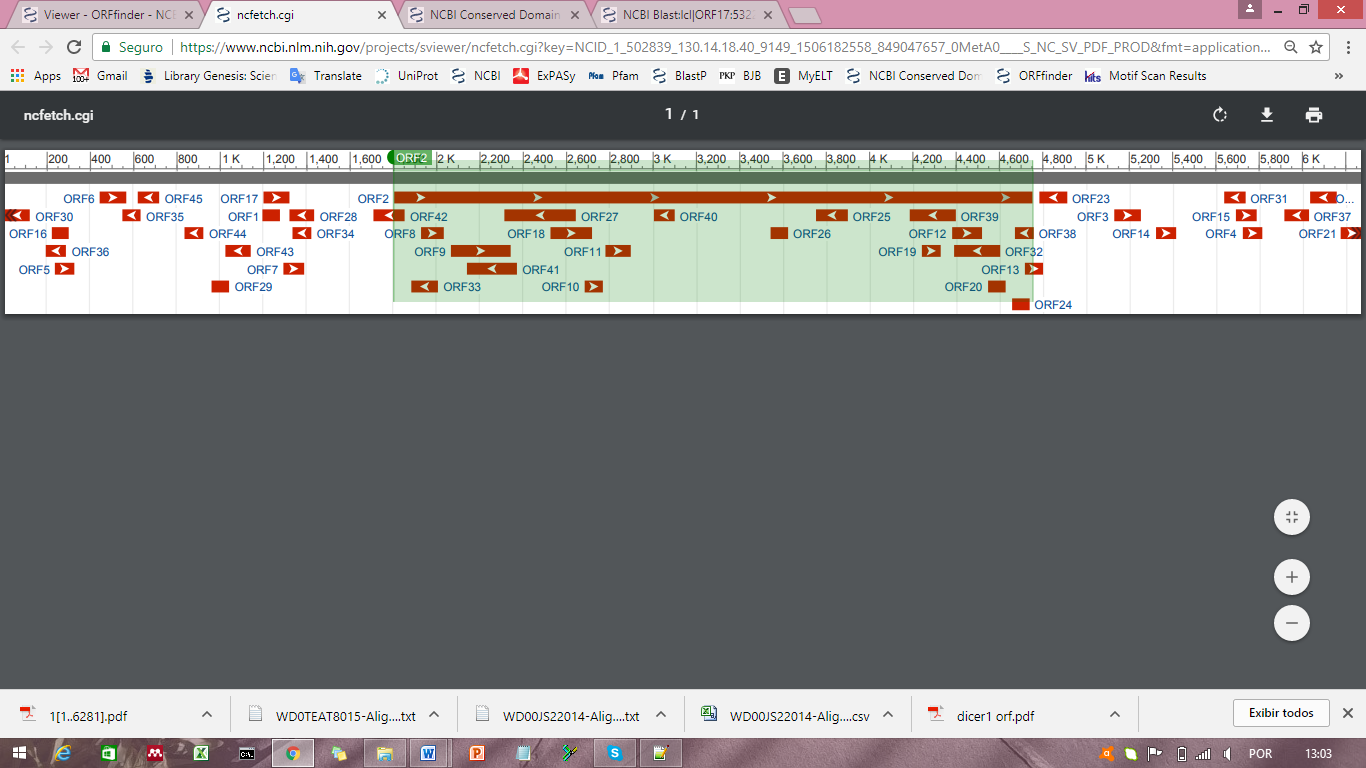


**BlastP (Non-redundant NCBI sequences)**

protein argonaute-2 isoform X1 [Ceratitis capitata]

Sequence ID: [XP_004529840.1](https://www.ncbi.nlm.nih.gov/protein/498982357?report=genbank&log$=protalign&blast_rank=1&RID=WD1HUPHK014) Length: 982 Number of Matches: 1

Range 1: 1 to 982

Score: 2035 bits(5272)

E-value: 0.0

Identities: 971/982(99%)

Query 1 MSTERELAPGGPAQLHTLPVAYSDLGATLQLNTAVRIIGKVYESQWTPSPPRPQSPSQSQ 60

MSTERELAPGGPAQLHTLP+AYSDLG TLQLNTAVRIIGKVYESQWTPSPPRPQSP+QSQ

Sbjct 1 MSTERELAPGGPAQLHTLPIAYSDLGTTLQLNTAVRIIGKVYESQWTPSPPRPQSPTQSQ 60

Query 61 TNYDPHTSPPATGSSVNPTAVTSPSAQNVAVGGATVGGAGATGHSTQAAAVASPLGAAAA 120

T++DP TSPPATGSSVNPTAVTSPSAQNVAVGGATVGGAGA G +TQA+AVASPLGAAAA

Sbjct 61 TSFDPLTSPPATGSSVNPTAVTSPSAQNVAVGGATVGGAGAAGLATQASAVASPLGAAAA 120

Query 121 TSPTTQPELPVFTCPRRPNLGREGRPIVLRANHFQVSMPRGYVHHYDINIQPDKCPRKVN 180

SPTTQPELPVFTCPRRPNLGREGRPIVLRANHFQVSMPRGYVHHYDINIQPDKCPRKVN

Sbjct 121 ASPTTQPELPVFTCPRRPNLGREGRPIVLRANHFQVSMPRGYVHHYDINIQPDKCPRKVN 180

Query 181 REIIETMVHAYSKIFGVLKPVFDGRNNLYTRDPLPIGNDRLELEVTLPGEGKDRIFRVTI 240

REIIETMVHAYSKIFGVLKPVFDGRNNLYTRDPLPIGNDRLELEVTLPGEGKDRIFRVTI

Sbjct 181 REIIETMVHAYSKIFGVLKPVFDGRNNLYTRDPLPIGNDRLELEVTLPGEGKDRIFRVTI 240

Query 241 KWMAQVSLFNLEEALEGRTRQIPYDAILALDVVMRHLPSMTYTPVGRSFFSSPDGYYHPL 300

KWMAQVSLFNLEEALEGRTRQIPYDAILALDVVMRHLPSMTYTPVGRSFFSSPDGYYHPL

Sbjct 241 KWMAQVSLFNLEEALEGRTRQIPYDAILALDVVMRHLPSMTYTPVGRSFFSSPDGYYHPL 300

Query 301 GGGREVWFGFHQSVRPSQWKMMLNIDVSATAFYKAQPVIDFMCEVLDIRDINEQRKPLTD 360

GGGREVWFGFHQSVRPSQWKMMLNIDVSATAFYKAQPVIDFMCEVLDIRDINEQRKPLTD

Sbjct 301 GGGREVWFGFHQSVRPSQWKMMLNIDVSATAFYKAQPVIDFMCEVLDIRDINEQRKPLTD 360

Query 361 SQRVKFTKEIKGLKIEITHCGAMRRKYRVCNVTRRPAQMQSFPLQLENGQTVECTVAKYF 420

SQRVKFTKEIKGLKIEITHCGAMRRKYRVCNVTRRPAQMQSFPLQLENGQTVECTVAKYF

Sbjct 361 SQRVKFTKEIKGLKIEITHCGAMRRKYRVCNVTRRPAQMQSFPLQLENGQTVECTVAKYF 420

Query 421 LDKYRMKLRYPHLPCLQVGQEHKHTYLPLEVCNIVAGQRCIKKLTDMQTSTMIKATARSA 480

LDKYRMKLRYPHLPCLQVGQEHKHTYLPLEVCNIVAGQRCIKKLTDMQTSTMIKATARSA

Sbjct 421 LDKYRMKLRYPHLPCLQVGQEHKHTYLPLEVCNIVAGQRCIKKLTDMQTSTMIKATARSA 480

Query 481 PDREREINNLVKRADFNNDSYVQEFGLTISNSMMEVRGRVLPPPKLQYGGRVSSMTGQQL 540

PDREREINNLVKRADFNNDSYVQEFGLTISNSMMEVRGRVLPPPKLQYGGRVSSMTGQQL

Sbjct 481 PDREREINNLVKRADFNNDSYVQEFGLTISNSMMEVRGRVLPPPKLQYGGRVSSMTGQQL 540

Query 541 FPPQNKVSLASPNQGVWDMRGKQFFTGVEIRVWAIACFAPQRTVREDALRNFTQQLQKIS 600

FPPQNKVSLASPNQGVWDMRGKQFFTGVEIRVWAIACFAPQRTVREDALRNFTQQLQKIS

Sbjct 541 FPPQNKVSLASPNQGVWDMRGKQFFTGVEIRVWAIACFAPQRTVREDALRNFTQQLQKIS 600

Query 601 NDAGMPIIGQPCFCKYATGPDQVEPMFRYLKNSFNALQLVVVVLPGKTPVYAEVKRVGDT 660

NDAGMPIIGQPCFCKYATGPDQVEPMFRYLKNSFNALQLVVVVLPGKTPVYAEVKRVGDT

Sbjct 601 NDAGMPIIGQPCFCKYATGPDQVEPMFRYLKNSFNALQLVVVVLPGKTPVYAEVKRVGDT 660

Query 661 VLGMATQCVQAKNVNKTSPQTLSNLCLKINVKLGGINSILVPSIRPKVFNEPVIFLGADV 720

VLGMATQCVQAKNVNKTSPQTLSNLCLKINVKLGGINSILVPSIRPKVFNEPVIFLGADV

Sbjct 661 VLGMATQCVQAKNVNKTSPQTLSNLCLKINVKLGGINSILVPSIRPKVFNEPVIFLGADV 720

Query 721 THPPAGDNKKPSIAAVVGSMDAHPSRYAATVRVQQHRQEIIQELSSMVRELLIMFYKSTG 780

THPPAGDNKKPSIAAVVGSMDAHPSRYAATVRVQQHRQEIIQELSSMVRELLIMFYKSTG

Sbjct 721 THPPAGDNKKPSIAAVVGSMDAHPSRYAATVRVQQHRQEIIQELSSMVRELLIMFYKSTG 780

Query 781 GYKPHRIILYRDGVSEGQFPHVLQHELTAIREACIKLEAEYRPGITFIVVQKRHHTRLFC 840

GYKPHRIILYRDGVSEGQFPHVLQHELTAIREACIKLEAEYRPGITFIVVQKRHHTRLFC

Sbjct 781 GYKPHRIILYRDGVSEGQFPHVLQHELTAIREACIKLEAEYRPGITFIVVQKRHHTRLFC 840

Query 841 AEKKEQSGKSGNIPAGTTVDVGITHPTEFDFYLCSHQGIQGTSRPSHYHVLWDDNHFDSD 900

AEKKEQSGKSGNIPAGTTVDVGITHPTEFDFYLCSHQGIQGTSRPSHYHVLWDDNHFDSD

Sbjct 841 AEKKEQSGKSGNIPAGTTVDVGITHPTEFDFYLCSHQGIQGTSRPSHYHVLWDDNHFDSD 900

Query 901 ELQCLTYQLCHTYVRCTRSVSIPAPAYYAHLVAFRARYHLVEKEHDSGEGSHQSGCSEDR 960

ELQCLTYQLCHTYVRCTRSVSIPAPAYYAHLVAFRARYHLVEKEHDSGEGSHQSGCSEDR

Sbjct 901 ELQCLTYQLCHTYVRCTRSVSIPAPAYYAHLVAFRARYHLVEKEHDSGEGSHQSGCSEDR 960

Query 961 TPGAMARAITVHADTKKVMYFA 982

TPGAMARAITVHADTKKVMYFA

Sbjct 961 TPGAMARAITVHADTKKVMYFA 982

**Loquacious**

**>lcl|ORF5**

MKTPVSILQELLSRRGITPNYELVQIEGAIHEPTFRYRVAFNDKDVPFTA

MGAGRSKKEAKHSAARALIDKLTGVQLPDTAQACAGAGVSTASSGITATS

AGSDANVNAVGSGDGADKIVGNPIGWLQEMCMSRRWPPPTYETETEVGLP

HERLFTIACSILNYREVGKGKSKKIAKRLAAHKMWTRLQENPLDNNQICE

SMRVDLDGDNRNCANYYGDLKDITVPTLTSQHSNKVSQFHKTLKNATGKK

LLKLQKTCLKGPKIDYVKLLSEIANENQFEVTYVDIEEKTFTDQCQCLVQ

LSTLPVGVCHGSGPTAADAQKQAAQNALEYLKIMTKK

**Graphical representation - ORF**


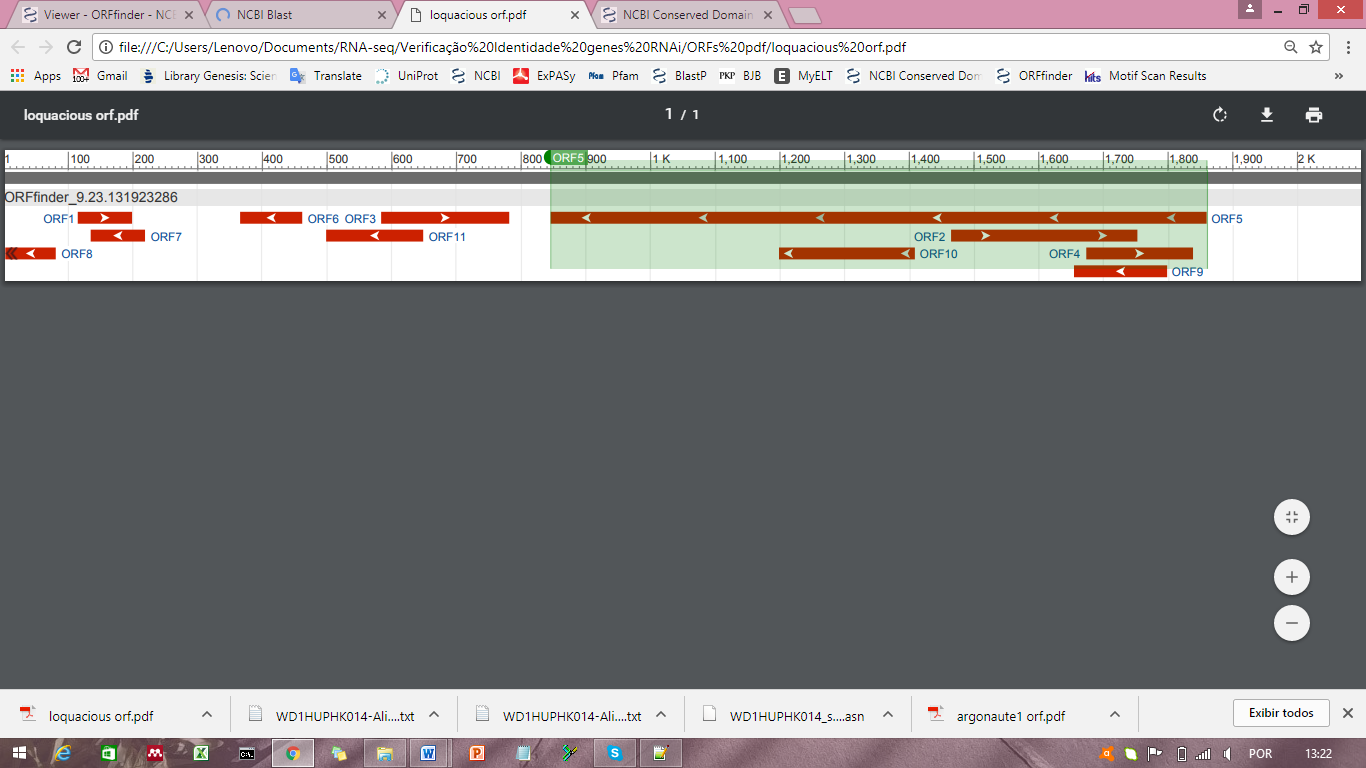


**BlastP (Non-redundant NCBI sequences)**

PREDICTED: RISC-loading complex subunit tarbp2 isoform X1 [Rhagoletis zephyria]

Sequence ID: [XP_017467180.1](https://www.ncbi.nlm.nih.gov/protein/1048012544?report=genbank&log$=protalign&blast_rank=1&RID=WD2JBVNV015)Length: 424Number of Matches: 1

Range 1: 88 to 424

Score: 659 bits(1700)

E-value: 0.0

Identities: 309/337(92%)

Query 1 MKTPVSILQELLSRRGITPNYELVQIEGAIHEPTFRYRVAFNDKDVPFTAMGAGRSKKEA 60

MKTPVSILQELLSRRGITPNYELVQIEGAIHEPTFRYRVAFNDKD+PFTAMGAGRSKKEA

Sbjct 88 MKTPVSILQELLSRRGITPNYELVQIEGAIHEPTFRYRVAFNDKDIPFTAMGAGRSKKEA 147

Query 61 KHSAARALIDKLTGVQLPDTAQACAGAGVSTASSGITATSAGSDANVNAVGSGDGADKIV 120

KHSAARALIDKLTGVQLPDTAQ AGVST+SSGITAT+ G D N NAVGSGDGADKIV

Sbjct 148 KHSAARALIDKLTGVQLPDTAQVGGSAGVSTSSSGITATNVGGDGNANAVGSGDGADKIV 207

Query 121 GNPIGWLQEMCMSRRWPPPTYETETEVGLPHERLFTIACSILNYREVGKGKSKKIAKRLA 180

GNPIGWLQEMCMSRRWPPPTYETETEVGLPHERLFTIACSILNYREVGKGKSKKIAKRLA

Sbjct 208 GNPIGWLQEMCMSRRWPPPTYETETEVGLPHERLFTIACSILNYREVGKGKSKKIAKRLA 267

Query 181 AHKMWTRLQENPLDNNQICESMRVDLDGDNRNCANYYGDLKDITVPTLTSQHSNKVSQFH 240

AHKMWTRLQENPLDNNQI E+MR D+DG++RNCANYYGDLKDITVPT+TSQHSNKVSQFH

Sbjct 268 AHKMWTRLQENPLDNNQISEAMRSDIDGEHRNCANYYGDLKDITVPTITSQHSNKVSQFH 327

Query 241 KTLKNATGKKLLKLQKTCLKGPKIDYVKLLSEIANENQFEVTYVDIEEKTFTDQCQCLVQ 300

KTLKNATGKKLLKLQKTCLKGPKIDY+KLLS+IANENQFEVTYVDIEEK+FTDQCQCLVQ

Sbjct 328 KTLKNATGKKLLKLQKTCLKGPKIDYMKLLSDIANENQFEVTYVDIEEKSFTDQCQCLVQ 387

Query 301 LSTLPVGVCHGSGPTAADAQKQAAQNALEYLKIMTKK 337

LST PV VCHG+GPT DA+K+AAQNALEYLKIMTKK

Sbjct 388 LSTFPVAVCHGTGPTTTDAKKRAAQNALEYLKIMTKK 424

**Drosha**

**>lcl|ORF22**

MNFQHLSTMPPLPTENVPTKTQPPLPVGMPPPPPSAPETFHLSTSKDVDC

ANFSNYSVAPPMPQTPATNSFHSFPSTFYQNSRIEGGTPIYPHFRDATVF

ADGYLKEEGYKGPHPAPRKTEYIPYEPTYNEYNYHKQKYEDSIHRNYRSG

RDMVMNKVHYKPPDGARAFSYEYNNSTSNPGHGVPRTAFRNTSNSTYTHR

LPFTPTSTQRGYGYAKYSESSRFYNQRERKNESRNDSKEQKYETERDRLL

KSWRSNYCETSDDIAEKLAELGEQDENSRSVWIRSSPADLFYRRTNVSNE

VVSTSRLEALCKLFEEKLVQRAERISDTLPPYEAPTRKPRRRLCKHKSEA

CSSSSEDSSDDDLKIEQDCCMEELTRKIQHPYRVHADLWHNDSGEMNDGP

LCRCSAKARRIGIRHGIYPGETGYSKCVMNSNNASRLYHYRITISPPTNF

LTKTPTIIKHDEHEFLFEGFSMLTHSPLADLPTCKVIRFNIEYTILYVEE

KMPENFTIRELELFNKYLFHELLELVDFSLYPSGMSSSETCPAFHFFPRF

VRDLPDNGKEVLAMSEVLRYLLDSAAPLVDREQLRCIKDISQHDWQDYVD

FIKGMLVTKPGFKPCSVRVDQLDRNVSDLPECFDVEENVTYPAIVHFGIR

PPQLSYAGNPEYQKAWREYVKYRHLMANMSKPSFVDKRKLEEKEARLQEM

RTQGRMKRNITVAVSSKSYYRTGIMCDIVQHAMLIPVLTGHLRFHRSLDL

LEQNIGYNFKNRYLLQLALTHPSYKENYGTNPDHARNSLTNCGIRQPEYG

DRKIHYLNTRKRGINTLINIMSRFGKEYETMSNITHNERLEFLGDAVVEF

LSSIHLFFMFPDLEEGGLATYRAAIVQNQHLALLAKKLHLEEYMLYAHGS

DLCHELELRHAMANCFEALMGALLLDGGISVADEVFMNALYMEDEQLREI

WKNYPEHPLQEQEPLGDRACIASYPVLQELTKFEESIGIQFKHIRLLARA

FTDRSIGFTHLTLGSNQRLEFLGDTVLQLICSEYLYRHFPEHHEGHLSLL

RSSLVNNRTQAVVCDDLGMTKYAVYSNPKVELKTKDRADLLEAFLGALYV

DKGLLYCEQFCHVCLFPRLQIFIMNQDWNDPKSKLQQCCLTLRTMEGGEP

DIPIYKVIESIGPTNTRVYTVAVYFRSKRLATATGSSIQQAEMNAAKQAL

ENSRDLFPQLDHQKRVIAKSIKKQKGTELKPELEEILAEQTKPKITNSME

DESHLPKQYRVREDISSDELPEDDINDAGNITNKGHSGHTSSSSSSSDDE

ILEKGRVPKLKKRSRTKKLEFIGHGSRSNLFSSKRMCHQVEEEDHQNTNK

RNCEDVSSDDCFGENTISTVSFTAENHKLSPNKPMPSSACKNVRSVGNTM

PHDLKDLVEDVSSNSDIESGELSS

**Graphical representation - ORF**


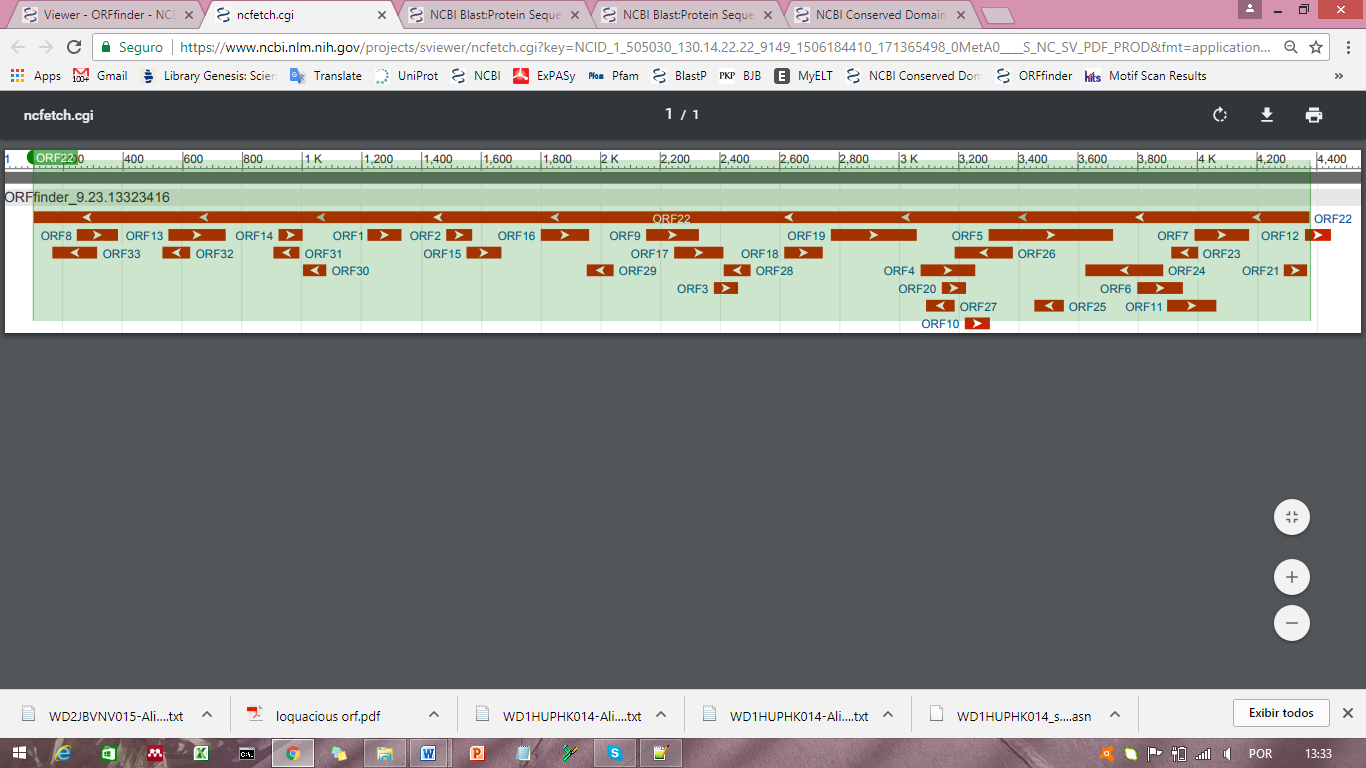


**BlastP (Non-redundant NCBI sequences)**

PREDICTED: ribonuclease 3 isoform X1 [Rhagoletis zephyria]

Sequence ID: [XP_017471286.1](https://www.ncbi.nlm.nih.gov/protein/1048021546?report=genbank&log$=protalign&blast_rank=1&RID=WD36KVUB015) Length: 1423 Number of Matches: 1

Range 1: 14 to 1423

Score: 2320 bits(6011)

E-value: 0.0

Identities: 1186/1430(83%)

Query 1 MNFQHLSTMPPLPTENVPTKTQPPLPVGMPPPPPS------APETFHLSTSKDVDCANFS 54

MN+ HLS PPLP E+V T+ QPPLP G+PP PP + E HL + D N S

Sbjct 14 MNYLHLSVAPPLPMESVATEPQPPLPPGLPPLPPPPPPPAPSSEPLHLYNTSAGDTTNQS 73

Query 55 NYSVAPPMPQTPATNSFHSFPSTFYQNSRIEGGTPIYPHFRDATVFADGYLKEEGYKGPH 114

S+ PQ P ++ H FPS +YQ ++G TP+YPH+R+++ + +GY E GY+

Sbjct 74 FTSI----PQVPGASNLHPFPSAYYQTPLLDGVTPLYPHYRESSTYVEGYYNEHGYRISS 129

Query 115 PAPRKTEYIPYEPTYNEYNYHKQKYEDSIHRNYRSGRDMVMNKVHYKPPDGARAFSYEYN 174

R+TEYIPYEPTYNEYNYHK KY+D+ H YRSGR + YK +G R+F YE+

Sbjct 130 STQRRTEYIPYEPTYNEYNYHKSKYDDNFHNKYRSGRGL------YKCAEGVRSFPYEHG 183

Query 175 NSTSNPGHGVPRTAFRNTSNSTYTHRLPFTPTSTQRGYGYAKYSESSRFYNQRERKNESR 234

+ N H R++FRNTS+STY HR P S+ R YGYAK+ + YN RE+ +SR

Sbjct 184 KTNINHVHSATRSSFRNTSSSTYIHRPHILPISSPRSYGYAKHPDYIGSYNPREKHYDSR 243

Query 235 NDSKEQKYETERDRLLKSWRSNYCETSDDIAEKLAELGEQDENSRSVWIRSSPADLFYRR 294

NDSK ++ ETERDRLLK+WRSNYCETS+DIAEKLAELGEQDE++R+VW+RSSPAD FYRR

Sbjct 244 NDSKGKRLETERDRLLKNWRSNYCETSEDIAEKLAELGEQDEDTRNVWVRSSPADPFYRR 303

Query 295 TNVSNEVVSTSRLEALCKLFEEKLVQRAERISDTLPPYEAPTRKPRRRLCKHKSEACSSS 354

TN SNEVVSTSRLEALCKLFEEKL+QRAER+ TLPPYE PTRK RRRLCKHKSEACSSS

Sbjct 304 TNASNEVVSTSRLEALCKLFEEKLIQRAERVRKTLPPYEPPTRKSRRRLCKHKSEACSSS 363

Query 355 SEDSSDDDLKIEQDCCMEELTRKIQHPYRVHADLWHNDSGEMNDGPLCRCSAKARRIGIR 414

SEDSSDD+LKIEQDCCMEEL+RKIQHPYRVHADLWHNDSGEMNDGPLCRCSAK+RRIGIR

Sbjct 364 SEDSSDDELKIEQDCCMEELSRKIQHPYRVHADLWHNDSGEMNDGPLCRCSAKSRRIGIR 423

Query 415 HGIYPGETGYSKCVMNSNNASRLYHYRITISPPTNFLTKTPTIIKHDEHEFLFEGFSMLT 474

HGIYPGETGY KCVMNSNNA RL+HYRITISPPTNFLTKTPTIIKHDEHEFLFEGFSMLT

Sbjct 424 HGIYPGETGYPKCVMNSNNAPRLHHYRITISPPTNFLTKTPTIIKHDEHEFLFEGFSMLT 483

Query 475 HSPLADLPTCKVIRFNIEYTILYVEEKMPENFTIRELELFNKYLFHELLELVDFSLYPSG 534

HSPLADLPTCKVIRFNIEYTILYV EKMPENFTIRELELF+K+LF ELLELVDFS+YP+G

Sbjct 484 HSPLADLPTCKVIRFNIEYTILYVAEKMPENFTIRELELFDKFLFDELLELVDFSMYPNG 543

Query 535 MSSSETCPAFHFFPRFVRDLPDNGKEVLAMSEVLRYLLDSAAPLVDREQLRCIKDISQHD 594

M+ +ETCPAFHF PRFVRDLPDNGKEVLAMSEVLRYLLDSAAPLVDREQL CI DI+QHD

Sbjct 544 MTGAETCPAFHFLPRFVRDLPDNGKEVLAMSEVLRYLLDSAAPLVDREQLHCINDINQHD 603

Query 595 WQDYVDFIKGMLVTKPGFKPCSVRVDQLDRNVSDLPECFDVEENVTYPAIVHFGIRPPQL 654

WQDYVDFIKGMLVTKPG+KPCSVRVDQLDRNVSDLPEC D EENV++PAIVHFGIRPPQL

Sbjct 604 WQDYVDFIKGMLVTKPGYKPCSVRVDQLDRNVSDLPECIDAEENVSHPAIVHFGIRPPQL 663

Query 655 SYAGNPEYQKAWREYVKYRHLMANMSKPSFVDKRKLEEKEARLQEMRTQGRMKRNITVAV 714

SYAGNPEYQKAWREYVKYRHLMANMSKPSF+DKRKLEEKEARLQEMRTQGRMKRNITVAV

Sbjct 664 SYAGNPEYQKAWREYVKYRHLMANMSKPSFIDKRKLEEKEARLQEMRTQGRMKRNITVAV 723

Query 715 SSKSYYRTGIMCDIVQHAMLIPVLTGHLRFHRSLDLLEQNIGYNFKNRYLLQLALTHPSY 774

SSK YYRTGIMCDIVQHAMLIPVLTGHLRFHRSLDLLEQNIGYNFKNRYLLQLALTHPSY

Sbjct 724 SSKGYYRTGIMCDIVQHAMLIPVLTGHLRFHRSLDLLEQNIGYNFKNRYLLQLALTHPSY 783

Query 775 KENYGTNPDHARNSLTNCGIRQPEYGDRKIHYLNTRKRGINTLINIMSRFGKEYETMSNI 834

KENYGTNPDHARNSLTNCGIRQPEYGDRKIHYLNTRKRGINTLINIMSRFGKEYETMSNI

Sbjct 784 KENYGTNPDHARNSLTNCGIRQPEYGDRKIHYLNTRKRGINTLINIMSRFGKEYETMSNI 843

Query 835 THNERLEFLGDAVVEFLSSIHLFFMFPDLEEGGLATYRAAIVQNQHLALLAKKLHLEEYM 894

THNERLEFLGDAVVEFLSSIHLFFMFPDLEEGGLATYRAAIVQNQHLALLAKKLHLEEYM

Sbjct 844 THNERLEFLGDAVVEFLSSIHLFFMFPDLEEGGLATYRAAIVQNQHLALLAKKLHLEEYM 903

Query 895 LYAHGSDLCHELELRHAMANCFEALMGALLLDGGISVADEVFMNALYMEDEQLREIWKNY 954

LYAHGSDLCHELELRHAMANCFEALMGALLLDGGISVADEVFMNALYMEDEQLREIWKNY

Sbjct 904 LYAHGSDLCHELELRHAMANCFEALMGALLLDGGISVADEVFMNALYMEDEQLREIWKNY 963

Query 955 PEHPLQEQEPLGDRACIASYPVLQELTKFEESIGIQFKHIRLLARAFTDRSIGFTHLTLG 1014

PEHPLQEQEPLGDR CIASYPVLQELTKFEESIGIQFKHIRLLARAFTDRSIGFTHLTLG

Sbjct 964 PEHPLQEQEPLGDRGCIASYPVLQELTKFEESIGIQFKHIRLLARAFTDRSIGFTHLTLG 1023

Query 1015 SNQRLEFLGDTVLQLICSEYLYRHFPEHHEGHLSLLRSSLVNNRTQAVVCDDLGMTKYAV 1074

SNQRLEFLGDTVLQLICSEYLYRHFPEHHEGHLSLLRSSLVNNRTQAVVCDDLGMTKYAV

Sbjct 1024 SNQRLEFLGDTVLQLICSEYLYRHFPEHHEGHLSLLRSSLVNNRTQAVVCDDLGMTKYAV 1083

Query 1075 YSNPKVELKTKDRADLLEAFLGALYVDKGLLYCEQFCHVCLFPRLQIFIMNQDWNDPKSK 1134

YSNPKVELKTKDRADLLEAFLGALYVDKGLLYCEQFCHVCLFPRLQIFIMNQDWNDPKSK

Sbjct 1084 YSNPKVELKTKDRADLLEAFLGALYVDKGLLYCEQFCHVCLFPRLQIFIMNQDWNDPKSK 1143

Query 1135 LQQCCLTLRTMEGGEPDIPIYKVIESIGPTNTRVYTVAVYFRSKRLATATGSSIQQAEMN 1194

LQQCCLTLRTMEGGEPDIPIYKVIESIGPTNTRVYTVAVYFRSKRLATATGSSIQQAEMN

Sbjct 1144 LQQCCLTLRTMEGGEPDIPIYKVIESIGPTNTRVYTVAVYFRSKRLATATGSSIQQAEMN 1203

Query 1195 AAKQALENSRDLFPQLDHQKRVIAKSIKKQKGTELKPELEEILAEQTKPKITNSMEDESH 1254

AAKQAL+NSRDLFPQLDHQKRVIAKSIKKQKG ELK E EEI+ EQTKPK TNSMEDESH

Sbjct 1204 AAKQALDNSRDLFPQLDHQKRVIAKSIKKQKGAELKTEFEEIMPEQTKPKTTNSMEDESH 1263

Query 1255 LPKQYRVREDISSDELPEDDINDAGNITNKGHSGHTSSSSSSSDDEILEKGRVPKLKKRS 1314

LPKQYR+REDISSDELPEDDIND G +T GH H+SSS SSS++E+ EK K KK+

Sbjct 1264 LPKQYRIREDISSDELPEDDINDGGIVTIAGHLRHSSSSHSSSEEEVSEKHFTSKRKKKK 1323

Query 1315 RTKKLEFIGHGSRSNLFSSKRMCHQVEEEDHQNTNKRNCEDVSSDDCFGENTISTVSFTA 1374

RTKK I SN SSK MCH++ ED+QN N+RNCEDVSSDDCF EN+IS +

Sbjct 1324 RTKKTNSIEMS--SNSCSSKVMCHEM--EDNQNANRRNCEDVSSDDCFEENSISNL---- 1375

Query 1375 ENHKLSPNKPMPSSACKNVRSVGNTMPHDLKDLVEDVSSNSDIESGELSS 1424

ENH+L K P+S+ ++ R V NTM HDLKDLVEDVSSNSDIESGE+SS

Sbjct 1376 ENHELPSIK--PASSKEDPRVVKNTMLHDLKDLVEDVSSNSDIESGEISS 1423

**Pasha**

**>lcl|ORF7**

MSSESDNTQRSPSSPPSLPSSPQPHKRMRLDEQTLHEEATAMKEKGFSLS

AIEEMKTRVEESNQSGVLDQQLRQFQVLDEVQGSGSEDSDIASENGDQNH

EHGDGYESSDLDIDDNEIENLLNENLPDDLKEPKKPKYEERFKTVLEEKG

HNHFEVLPEGWVQVTHNSGMPLFLHKQSRVVSASRPYFLGHGSVRKHAIP

LGAIPCLNYRRALEEEEAEMKRLNEASTKEVSVDATRDADEAIVDNIPKC

PYTGASSSAITPTESSQSTQVSSSTNGIAVNNENMDTSDLTISTQSAATL

TTPASTSTTATPTATATTTPGDLAANIAALKSLVPPAKIVTVTENTQNES

LTPEKLNQYCTKLFKFKVIRVLRFRSWNARRKFTKNRKHIKNLQRPTLPD

GTKLIKFPILAPSGDASANTRGRKEWIMNPNGKSYVCILHEYVQHALKKQ

PTYEFKELENAATPYSATVSINELKYGTGYGTSKKQAKSDAARETLEILI

PEMKDKITGIKQDKNAPKSNHKDLSVFDDIKIQDPRVAEFCNKTTEPSPH

AILLTCLQRNFGLGDVQINYEINRTKNKKNEFTMTVGKHTAKVLCKNKRE

GKQLASQAILQILHPHIKTWGSLLRLYGNNSIKTFKEKKLEEQEITVLQS

KAAINQPNYAILDKLKAEMLKLAEKNKSVKSMGTFVPPSDVDLPSSSGSN

LNNVEL

**Graphical representation - ORF**


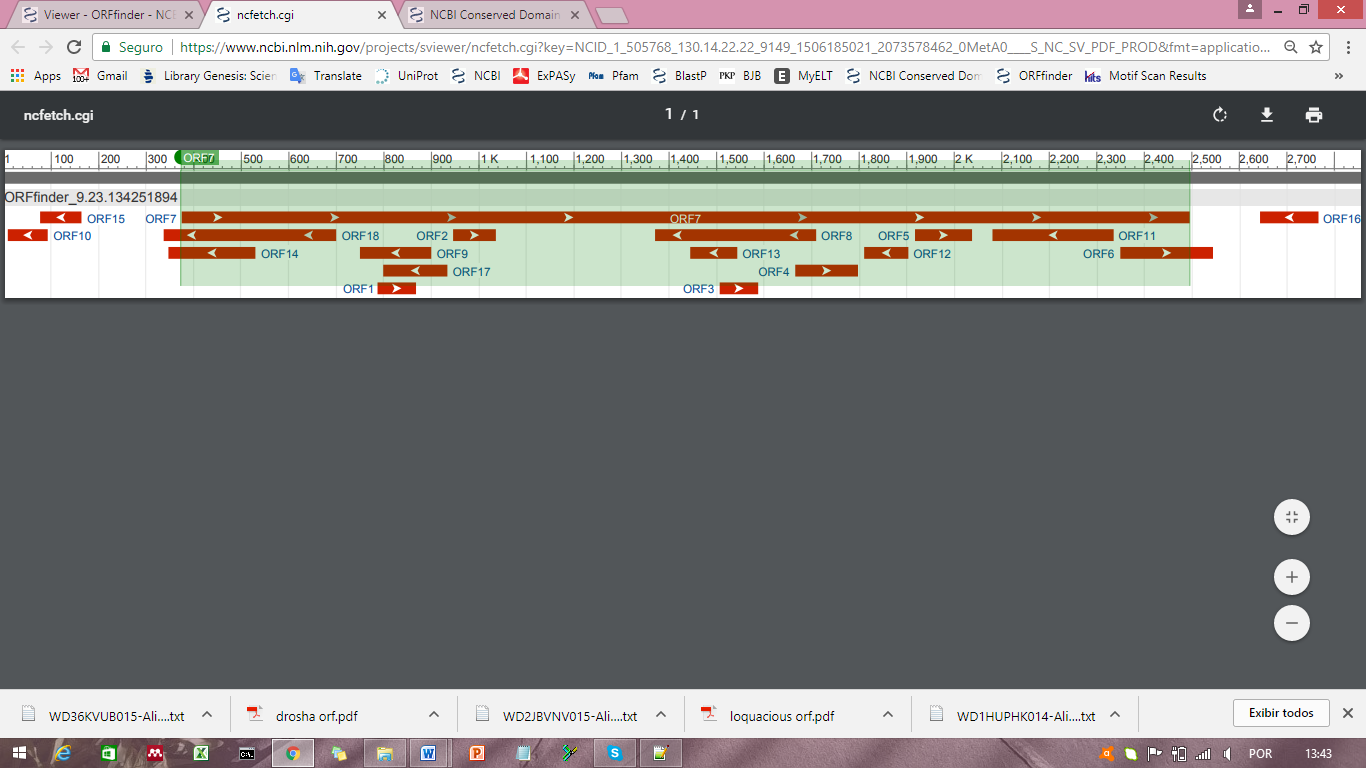


**BlastP (Non-redundant NCBI sequences)**

PREDICTED: microprocessor complex subunit DGCR8 [Bactrocera dorsalis]

Sequence ID: [XP_011210191.2](https://www.ncbi.nlm.nih.gov/protein/1132392233?report=genbank&log$=protalign&blast_rank=1&RID=WD4685NP01R) Length: 685 Number of Matches: 1

Range 1: 1 to 685

Score: 1231 bits(3184)

E-value: 0.0

Identities: 603/708(85%)

Query 1 MSSESDNTQRSPSSPPSLPSSPQPHKRMRLDEQTLHEEATAMKEKGFSLSAIEEMKTRVE 60

MSSESD QRS SSP + SPQ K++RLDEQ LH++ATAMKEKGFSLSAIEEMKTRVE

Sbjct 1 MSSESDFIQRSLSSPST---SPQLAKKIRLDEQALHQQATAMKEKGFSLSAIEEMKTRVE 57

Query 61 ESNQSGVLDQQLRQFQVLDEVQGSGSEDSDIASENGDQNHEHGDGYESSDLDIDDNEIEN 120

E+NQSG LDQQLRQFQVLDEVQGSGSEDS+IASENGD N ++GDGYESSDLDIDDNEIEN

Sbjct 58 ENNQSGTLDQQLRQFQVLDEVQGSGSEDSEIASENGDTNFDNGDGYESSDLDIDDNEIEN 117

Query 121 LLNENLPDDLKEPKKPKYEERFKTVLEEKGHNHFEVLPEGWVQVTHNSGMPLFLHKQSRV 180

LLNENLPDDLKEPKKPKYEERFKTVLEEKGHNHFEVLPEGWVQVTHNSGMPLFLHKQSRV

Sbjct 118 LLNENLPDDLKEPKKPKYEERFKTVLEEKGHNHFEVLPEGWVQVTHNSGMPLFLHKQSRV 177

Query 181 VSASRPYFLGHGSVRKHAIPLGAIPCLNYRRALEEEEAEMKRLNEASTKEVSVDATRDAD 240

VSASRPYFLGHGSVRKHAIPLGAIPCLNYRRA++EEE E RLN+ ++++ S +

Sbjct 178 VSASRPYFLGHGSVRKHAIPLGAIPCLNYRRAVDEEETE--RLNQKTSEKNSAMQAEEVI 235

Query 241 EAIVDNIPKCPYTGASSSAITPTESSQSTQVSSSTNGIAVNNENMDTSDLTISTQSAATL 300

E +PKCP+ GA+ SA+ T+SSQ QVSSSTNG + MD S+

Sbjct 236 ETNTVKVPKCPFAGANVSAVVSTDSSQFAQVSSSTNG-----KEMDISE----------- 279

Query 301 TTPASTSTTATPTATATTTP--GDLAANIAALKSLVPPAKIVTVTENTQNESLTPEKLNQ 358

PA+T+ + TPT A T+ DLAAN+A LKSLVPPAKIVTVTENTQ ESLTPE+LNQ

Sbjct 280 --PAATAISDTPTTIAPTSANAADLAANVATLKSLVPPAKIVTVTENTQKESLTPEQLNQ 337

Query 359 YCTKLFKFKVIRVLRFRSWNARRKFTKNRKHIKNLQRPTLPDGTKLIKFPILAPSGDASA 418

YC KLFKFKVIRVLRFRSWNARRKFTKNRKHIKNLQRPTLPDGTKLIKFPILAPSGD +A

Sbjct 338 YCAKLFKFKVIRVLRFRSWNARRKFTKNRKHIKNLQRPTLPDGTKLIKFPILAPSGDTNA 397

Query 419 NTRGRKEWIMNPNGKSYVCILHEYVQHALKKQPTYEFKELENAATPYSATVSINELKYGT 478

NTRGRKEWIMNPNGKSYVCILHEYVQHALKKQPTYEFKELENAATPYSATVSIN+LKYGT

Sbjct 398 NTRGRKEWIMNPNGKSYVCILHEYVQHALKKQPTYEFKELENAATPYSATVSINDLKYGT 457

Query 479 GYGTSKKQAKSDAARETLEILIPEMKDKITGIKQDKNAPKSNHKDLSVFDDIKIQDPRVA 538

GYGTSKKQAKSDAARETLEILIPEMKDKITGIKQDKNAPK+NHKDLSVFDDIKIQDPRVA

Sbjct 458 GYGTSKKQAKSDAARETLEILIPEMKDKITGIKQDKNAPKNNHKDLSVFDDIKIQDPRVA 517

Query 539 EFCNKTTEPSPHAILLTCLQRNFGLGDVQINYEINRTKNKKNEFTMTVGKHTAKVLCKNK 598

EFCNKTTEPSPHAILLTCLQRNFGLGDVQINYEINRTKNKKNEFTMTVGKHTAKVLCKNK

Sbjct 518 EFCNKTTEPSPHAILLTCLQRNFGLGDVQINYEINRTKNKKNEFTMTVGKHTAKVLCKNK 577

Query 599 REGKQLASQAILQILHPHIKTWGSLLRLYGNNSIKTFKEKKLEEQEITVLQSKAAINQPN 658

REGKQLASQAILQILHPHIKTWGSLLRLYGNNSIKTFKEKKLEEQEITVLQSKAAINQPN

Sbjct 578 REGKQLASQAILQILHPHIKTWGSLLRLYGNNSIKTFKEKKLEEQEITVLQSKAAINQPN 637

Query 659 YAILDKLKAEMLKLAEKNKSVKSMGTFVPPSDVDLPSSSGSNLNNVEL 706

YAILDKLK+EMLKLAEKNKSV SMGTFVPP DVDLPSSSGSNLNNVEL

Sbjct 638 YAILDKLKSEMLKLAEKNKSVVSMGTFVPPCDVDLPSSSGSNLNNVEL 68

**Exportin-5**

**>lcl|ORF1**

MTQSGNVAALGEELAQAIELIMRPDTAQQSRMEAYMACERFKEESPLCAQ

VGLYLASGQQFGQNVKHFGLQLMEYTIKFKWNSISHEEKLFIKENAMKLI

HFGVGPAEDASLAHLKDALSRIIVEMIKREWPQQWTTLLSELSEACNKGE

PQTELVLLVFLRLVEDVALLQTIESNQRRKDMYQALNNNMNDIFEFFQRL

IELHVTSFREATAHGNFQKANAHGRVVEVVLLTLTGFVEWVSMNHITSNN

CKLLQILCILLNDKAFQCNAAECLSQITNRKGQVKERKPLLMLFGEDPMR

YIFTASQMLPDAAVAGSLEQNHNFLKKLLNMLSGLGQQIVILWGKEDGSI

QRPQHFEIFLECLLLLTRHPSLTVAHDASLIWNMLLKHDGISKDATVVPY

IPKLIHVIAPRIIKTQYPSTRSLPTSVSTAAYICLEYDSEEEFAVFYYRC

RTDFLEVFRQSTLVQPVVTFTYCEQWLNARLAKAHTERDNVNCSVHDPVY

MEWEALVCVIDGVLSRILLVSERPSVQSGLRLLEECLKVETSNPLLLSIL

LSCISALFVFLSMSSCQITPNNCVAMSGVALLPRVLERIFEALVFRNPTE

PSTLTTRAQATKNLRRHAASLMVKLGHKYPLLLLPVFDQIDTHVKVLLDD

PRHALGKMERTTLQEALMLISNHFCDYERQTAFIANIMKSTLGHWSTFAE

VFKSAYTFIQFVGLDKPAVTAFQSDPLCINRGILLDSLNVVLAVIKRCTW

PDDPDRASRGGFVVGFTELGNPICRNPATPHIIPLLPHILALMRVLNELY

RPQAKALLSEDFRNVYSMLEHEKKTLLGVCTPPADPLDPTVKTVTSTVDR

MQQFMSLLYEGCYHMMGSAGPTLGRDLYQLQGISDALINTVFASLEDVPD

YRLRPIVRVFFKPFVYSCPPAFYDTVLVPIFAHFAPFMCYHLVQRWTYIS

SLYESGQLNEESNDTQEVLEDMLNRSLTREYLDVLKIALVGFGTDNVHAA

ANVTDVAMEPEEHSMDGTAHSRAAQSALLSDIISDLGAKLLRNDATGNHI

LMTLMAALSWQDSACSMKAVNVVAPVMRFLATNEIQLMDQHKAMTAFHAV

LQGLQVHGMHEANQAGLITLGVQFYELLRPKFPILSDVLRNIPNVSGADV

HKFDEKVSVAPLKGNKVDKAKKDIFKKMTARLVGRSVNQMFCRQIEILNL

PPMQAHAPKPHTDIVDITENAGLTQLFRTEK

**Graphical representation - ORF**


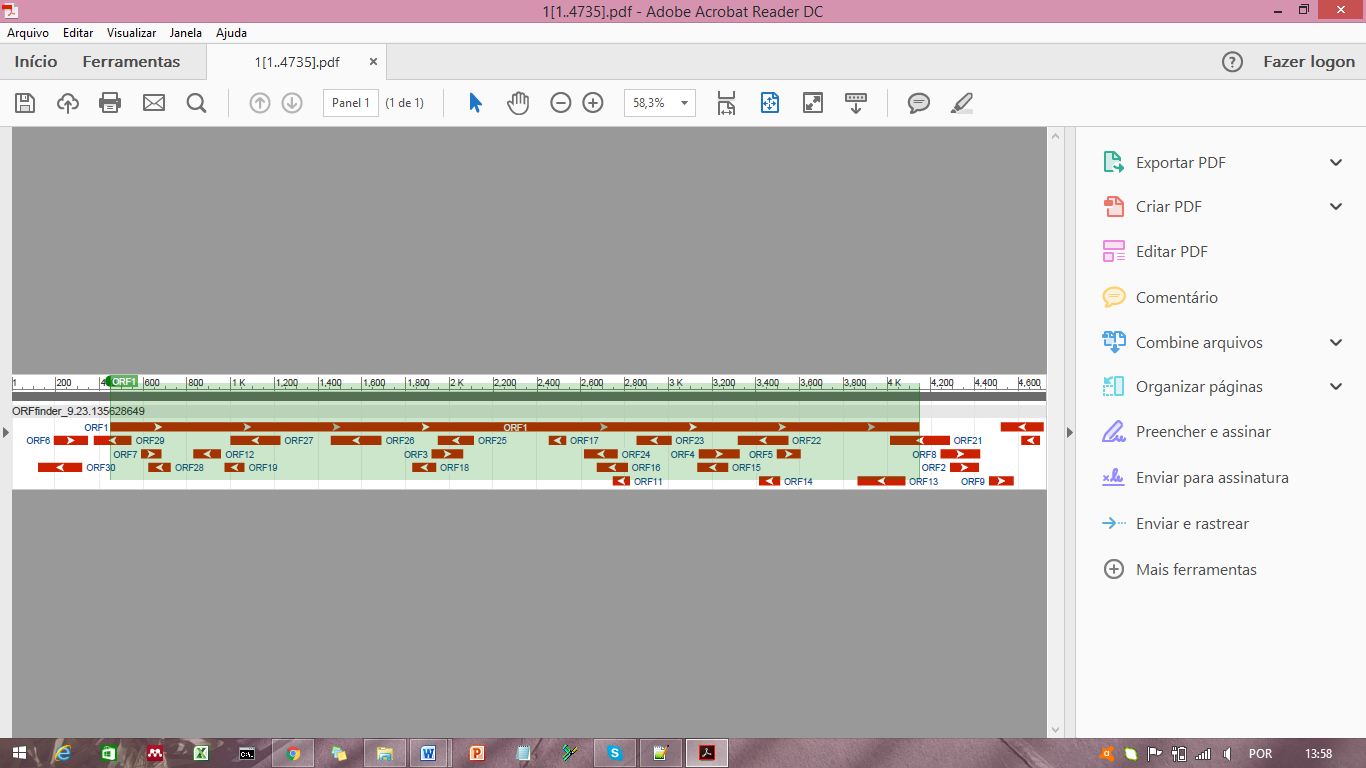


**BlastP (Non-redundant NCBI sequences)**

PREDICTED: exportin-5 [Rhagoletis zephyria]

Sequence ID: [XP_017473793.1](https://www.ncbi.nlm.nih.gov/protein/1048026238?report=genbank&log$=protalign&blast_rank=1&RID=WD4U3UDR015) Length: 1233 Number of Matches: 1

Range 1: 1 to 1233

Score: 2362 bits(6121)

E-value: 0.0

Identities: 1122/1233(91%)

Query 1 MTQSGNVAALGEELAQAIELIMRPDTAQQSRMEAYMACERFKEESPLCAQVGLYLASGQQ 60

MTQ GNVAALGEELAQAIELIMRPDTAQQ+RMEAYMACERFKEESPLCAQVGLYLASGQQ

Sbjct 1 MTQRGNVAALGEELAQAIELIMRPDTAQQARMEAYMACERFKEESPLCAQVGLYLASGQQ 60

Query 61 FGQNVKHFGLQLMEYTIKFKWNSISHEEKLFIKENAMKLIHFGVGPAEDASLAHLKDALS 120

FGQNVKHFGLQLMEYTIKFKWNSISHEEKLFIKENAMKL+HFGVGPAEDASLAHLKDALS

Sbjct 61 FGQNVKHFGLQLMEYTIKFKWNSISHEEKLFIKENAMKLLHFGVGPAEDASLAHLKDALS 120

Query 121 RIIVEMIKREWPQQWTTLLSELSEACNKGEPQTELVLLVFLRLVEDVALLQTIESNQRRK 180

RIIVEMIKREWPQQWTTLL+ELS+ACNKGEPQTELVLL+FLRLVEDVALLQTIESNQRRK

Sbjct 121 RIIVEMIKREWPQQWTTLLAELSDACNKGEPQTELVLLIFLRLVEDVALLQTIESNQRRK 180

Query 181 DMYQALNNNMNDIFEFFQRLIELHVTSFREATAHGNFQKANAHGRVVEVVLLTLTGFVEW 240

DMYQAL NNMNDIFEFFQRLIELHVTSFREATAHGNFQKANAHGRVVEVVLLTLTGFVEW

Sbjct 181 DMYQALTNNMNDIFEFFQRLIELHVTSFREATAHGNFQKANAHGRVVEVVLLTLTGFVEW 240

Query 241 VSMNHITSNNCKLLQILCILLNDKAFQCNAAECLSQITNRKGQVKERKPLLMLFGEDPMR 300

VSMNHITSNNCKLLQILCILLNDKAFQCNAAECLSQITNRKGQVKERKPLLMLFGE+PMR

Sbjct 241 VSMNHITSNNCKLLQILCILLNDKAFQCNAAECLSQITNRKGQVKERKPLLMLFGEEPMR 300

Query 301 YIFTASQMLPDAAVAGSLEQNHNFLKKLLNMLSGLGQQIVILWGKED--GSIQRPQHFEI 358

YIFTASQ+LPD A G+LEQNHNFLKKLLNMLSGL QQIVILWGKED GSIQRP HFEI

Sbjct 301 YIFTASQILPDIATPGALEQNHNFLKKLLNMLSGLAQQIVILWGKEDEDGSIQRPPHFEI 360

Query 359 FLECLLLLTRHPSLTVAHDASLIWNMLLKHDGISKDATVVPYIPKLIHVIAPRIIKTQYP 418

FLECLLLL RHPSLTVAH A+LIWN+LLKHDGISKD + PYIPK I VIAPRIIKT YP

Sbjct 361 FLECLLLLARHPSLTVAHGATLIWNVLLKHDGISKDPIMTPYIPKFIDVIAPRIIKTLYP 420

Query 419 STRSLPTSVSTAAYICLEYDSEEEFAVFYYRCRTDFLEVFRQSTLVQPVVTFTYCEQWLN 478

+ R+LPT+VSTAAYICLEYDSEEEFAVFYYRCRTDFLE+FRQSTL+QP+VTF+YCEQWLN

Sbjct 421 NMRTLPTAVSTAAYICLEYDSEEEFAVFYYRCRTDFLELFRQSTLIQPIVTFSYCEQWLN 480

Query 479 ARLAKAHTERDNVNCSVHDPVYMEWEALVCVIDGVLSRILLVSERPSVQSGLRLLEECLK 538

ARLAKAHTERDN+NCSV DPVY+EWEALVCVIDGVLSRILLVS+RP+VQSGLRLLEECLK

Sbjct 481 ARLAKAHTERDNINCSVQDPVYLEWEALVCVIDGVLSRILLVSDRPAVQSGLRLLEECLK 540

Query 539 VETSNPLLLSILLSCISALFVFLSMSSCQITPNNCVAMSGVALLPRVLERIFEALVFRNP 598

VETSNPL+LSILLSCISALFVFLSMSSCQITPNNCVAMSGV+LLPRVLERIF+ALVFR+P

Sbjct 541 VETSNPLILSILLSCISALFVFLSMSSCQITPNNCVAMSGVSLLPRVLERIFQALVFRDP 600

Query 599 TEPSTLTTRAQATKNLRRHAASLMVKLGHKYPLLLLPVFDQIDTHVKVLLDDPRHALGKM 658

TEP+ +TTRAQA KNLRRHAASLMVKLGHKYPLLLLPVFDQIDTHVK LLDDPRHAL K+

Sbjct 601 TEPNIMTTRAQAAKNLRRHAASLMVKLGHKYPLLLLPVFDQIDTHVKALLDDPRHALNKI 660

Query 659 ERTTLQEALMLISNHFCDYERQTAFIANIMKSTLGHWSTFAEVFKSAYTFIQFVGLDKPA 718

ERTTLQEAL+LISNHFCDYERQTAF+ANIMK TL HW TFAEVFKSA+TFIQFVGLDKPA

Sbjct 661 ERTTLQEALLLISNHFCDYERQTAFVANIMKDTLPHWPTFAEVFKSAFTFIQFVGLDKPA 720

Query 719 VTAFQSDPLCINRGILLDSLNVVLAVIKRCTWPDDPDRASRGGFVVGFTELGNPICRNPA 778

VT QSD L NRG +LD+LNVVLAVIKRCTWPDDPDRASRGGFVVGFTELGNPICRNPA

Sbjct 721 VTPIQSDTLSANRGHMLDALNVVLAVIKRCTWPDDPDRASRGGFVVGFTELGNPICRNPA 780

Query 779 TPHIIPLLPHILALMRVLNELYRPQAKALLSEDFRNVYSMLEHEKKTLLGVCTPPADPLD 838

TPH+IPLLPHILALMRVLNELYRPQA ALLSEDFR+VY+MLEHEKK LLGVCTPPADPLD

Sbjct 781 TPHVIPLLPHILALMRVLNELYRPQAMALLSEDFRHVYTMLEHEKKALLGVCTPPADPLD 840

Query 839 PTVKTVTSTVDRMQQFMSLLYEGCYHMMGSAGPTLGRDLYQLQGISDALINTVFASLEDV 898

PTVK VT+TVDR+QQFMSLLY+ CYHMMGSAGP+LGRDLYQLQGI+DALIN+VFASLE+V

Sbjct 841 PTVKKVTTTVDRLQQFMSLLYDSCYHMMGSAGPSLGRDLYQLQGIADALINSVFASLEEV 900

Query 899 PDYRLRPIVRVFFKPFVYSCPPAFYDTVLVPIFAHFAPFMCYHLVQRWTYISSLYESGQL 958

PDYRLRPIVRVFFKPFVYSCPP FYD+VLVPIFAH PFMCYHLVQRWTYISSLYESGQL

Sbjct 901 PDYRLRPIVRVFFKPFVYSCPPTFYDSVLVPIFAHLTPFMCYHLVQRWTYISSLYESGQL 960

Query 959 NEESNDTQEVLEDMLNRSLTREYLDVLKIALVGFGTDNVHAAANVTDVAMEPEEHSMDGT 1018

NEESN TQEVLEDMLNRSLTREYLDVLKIALVG G DNVHA ANVTDVAMEPEEHSMDGT

Sbjct 961 NEESNGTQEVLEDMLNRSLTREYLDVLKIALVGAGPDNVHAPANVTDVAMEPEEHSMDGT 1020

Query 1019 AHSRAAQSALLSDIISDLGAKLLRNDATGNHILMTLMAALSWQDSACSMKAVNVVAPVMR 1078

HSRAAQSALLSDIISDLGAKLLRN AT N+ILMTLMAALSWQDS CSMKAVNVVAPVMR

Sbjct 1021 THSRAAQSALLSDIISDLGAKLLRNGATSNYILMTLMAALSWQDSTCSMKAVNVVAPVMR 1080

Query 1079 FLATNEIQLMDQHKAMTAFHAVLQGLQVHGMHEANQAGLITLGVQFYELLRPKFPILSDV 1138

FLAT E QLMDQ+KA TAFHAVLQGLQVHGMHEANQAGLITLGVQFYELLRPKFP+LSDV

Sbjct 1081 FLATTEAQLMDQNKATTAFHAVLQGLQVHGMHEANQAGLITLGVQFYELLRPKFPVLSDV 1140

Query 1139 LRNIPNVSGADVHKFDEKVSVAPLKGNKVDKAKKDIFKKMTARLVGRSVNQMFCRQIEIL 1198

LR IPNV+ AD+ KFDEKV+VAPLKGNKVDKAKKD+FKKMTARLVGRSVNQMFCRQIEIL

Sbjct 1141 LRTIPNVNAADIQKFDEKVNVAPLKGNKVDKAKKDLFKKMTARLVGRSVNQMFCRQIEIL 1200

Query 1199 NLPPMQAHAPKPHTDIVDITENAGLTQLFRTEK 1231

NLPPMQA APKP++DIVDIT+NAGLT+LFR EK

Sbjct 1201 NLPPMQARAPKPNSDIVDITQNAGLTKLFRAEK 1233

**Dicer-2**

**>lcl|ORF17**

MTTNLVTNIIPKKAMEPRHYQLDLLNYVMDRNAIIYLPTGAGKTYVAIMA

LKRFSHQMQETIENGGKRAIFMCNTVELARQQAIELKRCTNLQIGFYVGE

RDVDSWSRKKWDEEISVNQVLVGTAQIFVDIVSQNYIKITDLSVVVIDEC

HHATRNHPMHEFMRHFQEVADKTKLPRVIGLTGVLLKGNKLTRIREELEC

LESTFRGNIVTVSSMEKYQNVMLYSTKPKEQLITFTRQTHDFQLLKHIER

IVSESFDVIKQWDLGTISVKQTKNLAGYRQNKKTFITNLLKDFLYQTNEF

GLYASAIAIMSPIMEFEIKKRQAETLALRNLYRYTISVCEKIRHMLVMEL

KEEDSLPDTVCHTLDIILNFSTPKMRTLLLLAKKLFSNKDPADIRCLIFV

ERRYTAKCVFYVLKKYAELEPALTKVLRPQFMVGRNSIMPSIESVLDQKW

NKSAIESFRSGECNVIVCSNVLEEGIDVQACNYVFAYDPLKTFNSYVQSK

GRARSNESIYAIFTPEVDKLQVASQIKKYQEAHQIIQNYLIGRILDRDDP

KEEKIAEQFVDLIKPFTLPSGAILFPSRALSLLYRYAQLLPSDAFGVALP

WFTKTSTERGIAVELKMPLQSSVKETIISDTFPTTKEAKISAAFKACIKL

YEHGELNDRLLPVTKRECVQKVAEELFDHWKKFNDDVTSKTAGKQQRRLY

NRKYPDELQNAIPHLNEVCYAYEIHAYPHFEINDYSLHISTLLQSNRNYA

ILSRKRIPPLAEMPLFMNQGKISVKIAPQPLTLTITNEQQLQKLCKFHLM

LFRDLLECWKTFFVLDQRNQENSYLIVPINAGKIDWELVENFQRLLPQRK

YSVSERKQKIYKPEDYIGKVVNKWYSGRENQRFVVTKVLTDRTPLSPFDN

NDYSSYVDFIDCKYKNEVDCVVQPNQFMLEVRALTSRRNFFINAVGKSSN

VHKNNCAIRLIPELCHNFMYPGDMWLKALFLPSILHRVHFLLHAENLRCR

VNTFLGIVDSVEYQPKQLAVDGSLKRAVDFDGNAMEEPDEPKKLLPAMPT

SSIEAEFEMLHIDDVAWKEYLEPEDFSRKPESLFPVELDYYYKFISGLAI

ELDALKLTEGEWSLSQSQLNMPNQNAAGSPVRGKDLCSISLCDVPLADKR

RLDILELTLSEQTLKTAEQADFLAAITTAGANDVFDMERFEFLGDSFLKF

SISLYLAHKYPKWHEGFLTEIKGKLVSNRNLIYCMLQTDIPECICGYLFK

PPHEWLPPLISLPRNLLELLKNNEQVLENLTPSDLYGIELDDDEILSGCC

SAVHLTKLNASSRQAHKLAEEERLDSGLNLFAYKEVLRDKVVADTLEAIL

GVCVKNYGIINTFRMLEFFGICKPDADQSFTRLMDLKLGGPLLHTNISSR

QVDSYIINYPKLEENLGYKFRDRAYLLQALTHPSYPTNRITGCYQELEFI

GDAILDFMVSCYIFERYSHMDPGMLTDLRSALVNNITLGCVCVRHRFHLF

LLAENSSLAESIKNFDKYQEKHNYYVTDQVHILSEENITTENEVEGATYN

ISDNVDVPKALGDILEALIAAVYLDSRDLRTTWHVIYGLLEQEFNQFAQQ

VPIDAVRQLNDHKHANPKYSKPISDNETYMVKCQFTCLDKSVEVNGFGLN

GKQAKKAAAKHALQILAKYSS

**Graphical representation - ORF**


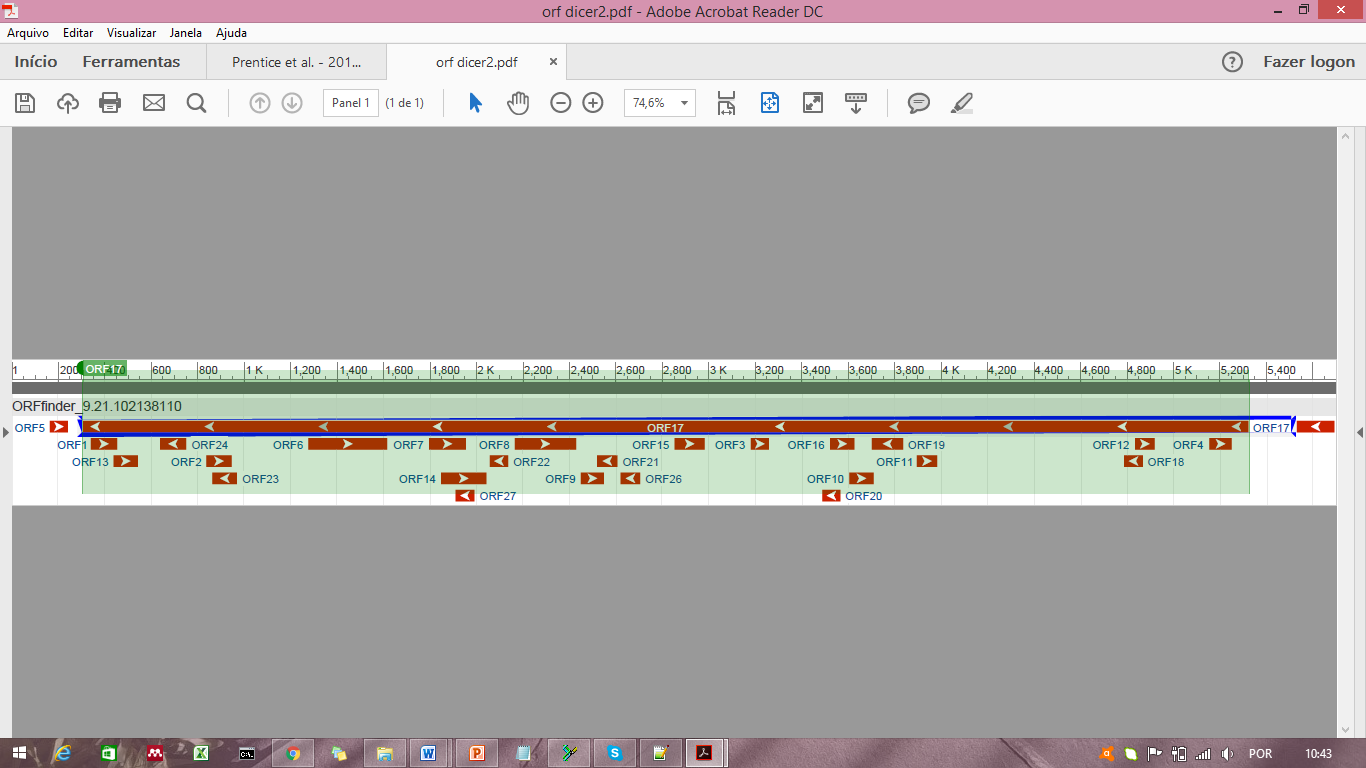


**BlastP (Non-redundant NCBI sequences)**

dicer2 [Bactrocera dorsalis]

Sequence ID: [AHI44612.1](https://www.ncbi.nlm.nih.gov/protein/582890011?report=genbank&log$=protalign&blast_rank=2&RID=WA2VWG0J015) Length: 1672 Number of Matches: 1

Range 1: 6 to 1672

Score: 2573 bits(6670)

E-value: 0.0

Identities: 1231/1672(74%)

Query 7 TNIIPKKAMEPRHYQLDLLNYVMDRNAIIYLPTGAGKTYVAIMALKRFSHQMQETIENGG 66

TN K MEPR YQ++LL+YVM RNAIIYLPTGAGKTY+AIMALKRFSHQMQETIENGG

Sbjct 6 TNTSDKAVMEPRRYQMELLSYVMQRNAIIYLPTGAGKTYIAIMALKRFSHQMQETIENGG 65

Query 67 KRAIFMCNTVELARQQAIELKRCTNLQIGFYVGERDVDSWSRKKWDEEISVNQVLVGTAQ 126

KRAIFMCNTVELARQQAIELKRCTNL +GFYVGERDVD+WS KKWD+EI +NQVLVGTAQ

Sbjct 66 KRAIFMCNTVELARQQAIELKRCTNLNVGFYVGERDVDNWSSKKWDDEIKINQVLVGTAQ 125

Query 127 IFVDIVSQNYIKITDLSVVVIDECHHATRNHPMHEFMRHFQEVADKTKLPRVIGLTGVLL 186

IFVDI+SQNYIKITDLSVV+IDECHHA NHPMHEFMRH++ ADK++LPRVIGLTGVL+

Sbjct 126 IFVDIISQNYIKITDLSVVIIDECHHARNNHPMHEFMRHYRMTADKSQLPRVIGLTGVLI 185

Query 187 KGNKLTRIREELECLESTFRGNIVTVSSMEKYQNVMLYSTKPKEQLITFTRQTHDFQLLK 246

KGNKL +IREELE LE+ F G IVTV SME+YQNVM YSTKP E+L+ +TRQTH F+L++

Sbjct 186 KGNKLNKIREELENLEAIFHGKIVTVGSMEEYQNVMAYSTKPTEKLVAYTRQTHIFRLVE 245

Query 247 HIERIVSESFDVIKQWDLGTISVKQTKNLAGYRQ-NKKTFITNLLKDFLYQTNEFGLYAS 305

I+ I+ E ++ QWDLG I VKQ+KNL RQ NKK FITNLL DF+YQ +EFGLYA+

Sbjct 246 TIKDIIKECAVMVNQWDLGAIKVKQSKNLGSLRQPNKKIFITNLLNDFVYQMDEFGLYAA 305

Query 306 AIAIMSPIMEFEIKKRQAETLALRNLYRYTISVCEKIRHMLVMELKEEDSLPDTVCHTLD 365

AIAIMSPI+EFE+KKR AET+ LRNLYRY IS CEKIRH+++ ELKEE + PD +TLD

Sbjct 306 AIAIMSPIIEFELKKRAAETMVLRNLYRYVISTCEKIRHIIIDELKEEITDPDDAYNTLD 365

Query 366 IILNFSTPKMRTLLLLAKKLFSNKDPADIRCLIFVERRYTAKCVFYVLKKYAELEPALTK 425

II N+STPKMR+LLLLAK+LFSNKDPADI CLIFVERRYTAKCV+YVL+ Y LEP L K

Sbjct 366 IIFNYSTPKMRSLLLLAKELFSNKDPADIHCLIFVERRYTAKCVYYVLQHYINLEPILAK 425

Query 426 VLRPQFMVGRNSIMPSIESVLDQKWNKSAIESFRSGECNVIVCSNVLEEGIDVQACNYVF 485

LRPQFMVGRNS++ SIES+L QKWN +AIESFR+G+CN++VCSNVLEEGIDVQACNYVF

Sbjct 426 SLRPQFMVGRNSVVASIESLLYQKWNNTAIESFRNGDCNLLVCSNVLEEGIDVQACNYVF 485

Query 486 AYDPLKTFNSYVQSKGRARSNESIYAIFTPEVDKLQVASQIKKYQEAHQIIQNYLIGRIL 545

AYDPLKTFNSYVQ+KGRARS++++YAIF + D+ V +IK YQEAH+ IQ++LIGRIL

Sbjct 486 AYDPLKTFNSYVQTKGRARSSDALYAIFAEKSDEAIVVQKIKGYQEAHKTIQDFLIGRIL 545

Query 546 DRDDPKEEKIAEQFVDLIKPFTLPSGAILFPSRALSLLYRYAQLLPSDAFGVALPWFTKT 605

D+D+PKE+ IAEQF DLIKPF SGA L S AL LLYRY LLP+DAFGVA PWF K

Sbjct 546 DQDEPKEQNIAEQFEDLIKPFRTKSGAYLLASNALPLLYRYCALLPTDAFGVATPWFNKQ 605

Query 606 STERG-IAVELKMPLQSSVKETIISDTFPTTKEAKISAAFKACIKLYEHGELNDRLLPVT 664

T+ I V L+MPLQSS+K TI+S+ + T K+A+ISAAF+AC LYE GELN+RLLPVT

Sbjct 606 ITDNNMIRVALQMPLQSSIKLTILSEGYRTVKQAQISAAFEACKMLYEQGELNERLLPVT 665

Query 665 KRECVQKVAEELFDHWKKFNDDVTSKTAGKQQRRLYNRKYPDELQNAIPHLNEVCYAYEI 724

K ECV KV+EELF HWKKFND+VTSKTAGKQQRRLYN +YPDEL NA+P +NE+CYAYEI

Sbjct 666 KTECVAKVSEELFSHWKKFNDNVTSKTAGKQQRRLYNHRYPDELYNAVPQINELCYAYEI 725

Query 725 HAYPHFEINDYSLHISTLLQSNRNYAILSRKRIPPLAEMPLFMNQGKISVKIAPQPLTLT 784

AYP FE + Y+ HI TLL +N NYAIL+RK +P LAEMPLFMNQGK+SVKIA +P+TLT

Sbjct 726 RAYPQFEADSYTEHIVTLLNTNCNYAILTRKPMPALAEMPLFMNQGKLSVKIAEKPITLT 785

Query 785 ITNEQQLQKLCKFHLMLFRDLLECWKTFFVLDQRNQENSYLIVPINAGKIDWELVENFQR 844

I +EQQL++L KFHLM+FRD+LECWK+F VLD+RN+EN+Y I P+N GKIDWELV+NFQR

Sbjct 786 IRSEQQLKQLAKFHLMIFRDILECWKSFLVLDRRNKENAYFIAPLNKGKIDWELVQNFQR 845

Query 845 LLPQRKYSVSERKQKIYKPEDYIGKVVNKWYSGRENQRFVVTKVLTDRTPLSPFDNNDYS 904

LLPQRKYSVSER++K+YKPEDY+GKVVNKWYSGR+NQRFVVTKVLTD TP S FDN+ +S

Sbjct 846 LLPQRKYSVSERQKKVYKPEDYLGKVVNKWYSGRDNQRFVVTKVLTDLTPQSAFDNDQFS 905

Query 905 SYVDFIDCKYKNEVDCVVQPNQFMLEVRALTSRRNFFINAVGKSSNVHKNNCAIRLIPEL 964

SY++FID KYKNEVDCVVQ +QFMLEVRALTSRRNFF+NAVGKS+ H N+ IRLIPEL

Sbjct 906 SYIEFIDAKYKNEVDCVVQKDQFMLEVRALTSRRNFFVNAVGKSAKSHHNSSIIRLIPEL 965

Query 965 CHNFMYPGDMWLKALFLPSILHRVHFLLHAENLRCRVNTFLGIVDSVEYQPKQLAVDGSL 1024

CHNFM+PGDMWLKAL LPSILHRVHF+LHAENLR RVN FLGI +V YQPK+L +D SL

Sbjct 966 CHNFMFPGDMWLKALLLPSILHRVHFMLHAENLRRRVNKFLGIACNVAYQPKKLMIDESL 1025

Query 1025 KRAVDFDGNAMEEPDEPKKLLPAMPTSSIEAEFEMLHIDDVAWKEYLEPEDFSRKPESLF 1084

RA+D DGNAMEEP EPKK+LPA+PT + E F M +++DV WK+YLEPED SRK E +F

Sbjct 1026 ARAIDMDGNAMEEPQEPKKVLPALPTQAKETVFTMTNLNDVLWKDYLEPEDLSRKQELMF 1085

Query 1085 PVELDYYYKFISGLAIELDALKLTEGEWSLSQSQLNMPNQNAAGSPVRGKDLCSISLCDV 1144

PVELDY+YKFI+G LD L L+E EW S+SQL+MP + A + ++ + LCD

Sbjct 1086 PVELDYFYKFINGSI--LDTLTLSEEEW--SESQLDMPKKPALETAGNSMNIDRLKLCDA 1141

Query 1145 PLADKRRLDILELTLSEQTLKTAEQADFLAAITTAGANDVFDMERFEFLGDSFLKFSISL 1204

PL +KR L+ILELTLS + LK+AEQADFLAA+TTAGANDVFDMERFEFLGDSFLKFS+SL

Sbjct 1142 PLTEKRHLNILELTLSGENLKSAEQADFLAALTTAGANDVFDMERFEFLGDSFLKFSVSL 1201

Query 1205 YLAHKYPKWHEGFLTEIKGKLVSNRNLIYCMLQTDIPECICGYLFKPPHEWLPPLISLPR 1264

YL HK+PKWHEGFLTE+KGK VSNRNLIYCML TDIPE I GYLFKPPHEWLPPL+SLP

Sbjct 1202 YLVHKFPKWHEGFLTEVKGKFVSNRNLIYCMLDTDIPERISGYLFKPPHEWLPPLVSLPS 1261

Query 1265 NLLELLKNNEQVLENLTPSDLYGIELDDDEILSGCCSAVHLTKLNASSRQAHKL-----A 1319

NLLELL NE +L++LTPSDLY I+L +DEI++G CS+ L+++ S Q H + A

Sbjct 1262 NLLELLNKNENILKSLTPSDLYAIQLSEDEIINGACSSERLSQI-VSGSQRHCIDDGPAA 1320

Query 1320 EEERLDSGLNLFAYKEVLRDKVVADTLEAILGVCVKNYGIINTFRMLEFFGICKPDADQS 1379

EE RLD+ LNLF YKE LRDKVVADTLEAILGVCVKNYGI NTFRMLEFFGICKPD +S

Sbjct 1321 EERRLDNELNLFIYKEALRDKVVADTLEAILGVCVKNYGIYNTFRMLEFFGICKPDPGKS 1380

Query 1380 FTRLMDLKLGGPLLHTNISSRQVDSYIINYPKLEENLGYKFRDRAYLLQALTHPSYPTNR 1439

F+ LMDL LL +IS ++VDS++INY KLEENLGYKFRDRA+LLQALTHPSYPTNR

Sbjct 1381 FSHLMDLNFTSALLQASISPKEVDSFLINYQKLEENLGYKFRDRAFLLQALTHPSYPTNR 1440

Query 1440 ITGCYQELEFIGDAILDFMVSCYIFERYSHMDPGMLTDLRSALVNNITLGCVCVRHRFHL 1499

ITGCYQELEFIGDAILDF++SCYIFERY HM PGMLTDLRSALVNN+TLG VCVRHRFHL

Sbjct 1441 ITGCYQELEFIGDAILDFLISCYIFERYQHMTPGMLTDLRSALVNNVTLGGVCVRHRFHL 1500

Query 1500 FLLAENSSLAESIKNFDKYQEKHNYYVTDQVHILSEENITTENEVEGATYNISDNVDVPK 1559

F+LAENS+LAE IK+F KYQEKHNY V+D V IL EE I NE GA +N++ NV+VPK

Sbjct 1501 FILAENSALAEKIKDFAKYQEKHNYIVSDDVQILMEETIVPTNETNGAAFNMAANVEVPK 1560

Query 1560 ALGDILEALIAAVYLDSRDLRTTWHVIYGLLEQEFNQFAQQVPIDAVRQLNDHKHANPKY 1619

ALGDILEALIAAVYLDSRDLRTTWHVIYGLLEQE NQF Q +P+DAVRQL +HK ANPK+

Sbjct 1561 ALGDILEALIAAVYLDSRDLRTTWHVIYGLLEQEINQFTQNIPLDAVRQLVEHKFANPKF 1620

Query 1620 SKPISDNETYMVKCQFTCLDKSVEVNGFGLNGKQAKKAAAKHALQILAKYSS 1671

+ PI D + YMVKCQFTCL++SVEVNGFG NGK AKKAAAK ALQILAK S+

Sbjct 1621 ADPICDEDVYMVKCQFTCLNRSVEVNGFGANGKLAKKAAAKQALQILAKRST 1672

**Argonaute-2**

**>lcl|ORF7**

MGKKNKNKTGGGQSSGEPAPTGPTPPQGQQRQPPQHEMPTSSGSASAQQS

QQSQRQQEQRPKQQEQLSGQQHPQQRKQGQGQQQPQGQKQQQKQARGQQQ

QYEQQEQTWRKQQPQGQQQQQQQQQPQGQQQQQQRKQAQGQQQPHQQQQP

QLQEQTWRQQQPQQEKQAWGQQQPQQQEQPQQRKQARGQQQQQQKQPQEQ

QQPQQRKQARGQQQQQQQKQPQEQQQPQQRKQAWGQQQPHEQQQPQLQEQ

TWRQQQPQQQKQAWGQQQPQHQKQAWGQQQPQQQEFKQQPSSSSYTQSSG

AVPKQAGEQPSPQRQTPIQAASQTASSRSSRTSISQSSSSTSITSSTSQR

SIQPGTLGIKGEVEANYLVLNLGKMPDIAYHYDVTITPDRPKKFFRSAFK

QFINTHLPGQTVAFDGVKSCYMVERLPNPVYEGDVKIADSGSRQIQFRVS

IKLTDNPEVELRSLKTYHNERVFDKPMRALQCIEVVLANDCHNKGIRAGR

SFFTQPDKTMDLDEGYELYTGLYQAAILGEQPYLNVDISHKSFPMPYDLI

TYLESVLNCNRQSNLDPRNLQRLSKHLKNLKVVYNPPPSFGAGPRSYKVN

DISREPAATLSFTTDNGEKFTVQKYFQSRGYNLRYPNLNCVVAGSTIRPN

YFPMELCSIEAGQAIRRKDGSRQVQKMIRFAATSTDERKRKIMQKLAYFN

INADRLVQAFGISVGEQFIKVPMRLLKAPAIEYHSSKYVEPRNGSWRNLP

FLETGAAFKKSGHKWAIIYTPSRFLKYPTLMDLANMLYNGAKRMGINLDA

QKDIKETGNIVATLEEYKRNDYDLVIVVIPGFGTSYADIKQKAELVCGLL

TQCIKEQTLNRGVNDMLISNLLLKVNSKLNGSNHKISANHHVVLDHVMFM

GADVTHPSPDQNNIPSVVGVAASHDLNGSVYNMQYRLQEPAKEEIVDMRT

IASHHLRVYFQKQKCYPNNIIYYRDGVSDGQFQKVEMLELGAIRAVCKEL

RITPKITCIIVVKRHHTRFFPTKPTGDKWNNVLPGTVVDQKIVHPNETQF

FMVSHQSIQGTAKPTRYNVIVDDAKMSMDDLQKMTNNLCYMFPRCNRAVS

YPAPAYLAHLVAARGRVYIDGPPLRRALPEEYKKRLINERFMNTTPMFFV

**Graphical representation - ORF**


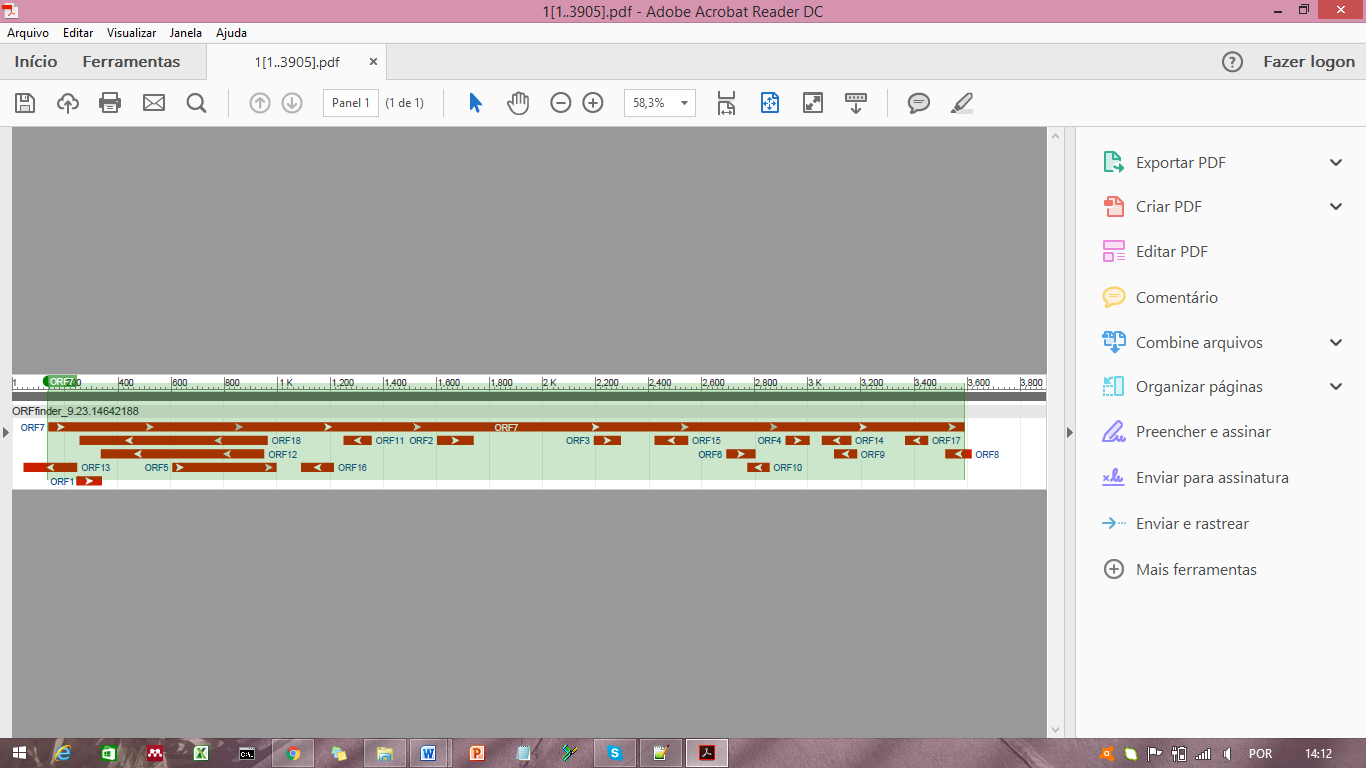


**BlastP (Non-redundant NCBI sequences)**

PREDICTED: protein argonaute-2 [Rhagoletis zephyria]

Sequence ID: [XP_017474419.1](https://www.ncbi.nlm.nih.gov/protein/1048027426?report=genbank&log$=protalign&blast_rank=1&RID=WD5E1PWC015) Length: 1140 Number of Matches: 1

Range 1: 246 to 1140

Score: 1483 bits(3839)

E-value: 0.0

Identities: 713/896(80%)

Query 263 AWGQQQPQHQKQ-----AWGQQQPQQQEFKQQPSSSSYTQSSGAVPKQAGEQPSPQRQTP 317

WG++Q Q Q WG+ Q Q Q PSSSS Q+ ++PKQ G P QRQ P

Sbjct 246 GWGKEQQATQPQKPNLGGWGRGQQQPQASAMGPSSSS-IQARESIPKQVGGPPPSQRQAP 304

Query 318 IQAASQTASSR---SSRTSISQSSSSTSITSSTSQRSIQPGTLGIKGEVEANYLVLNLGK 374

AASQ+ + + R S+S+SSS+ S +SS SQ+SIQPGTLGI+GEVE NYLVLNL K

Sbjct 305 QAAASQSQTPSPQYTPRQSMSKSSSTASFSSSVSQKSIQPGTLGIRGEVETNYLVLNLDK 364

Query 375 MPDIAYHYDVTITPDRPKKFFRSAFKQFINTHLPGQTVAFDGVKSCYMVERLPNPVYEGD 434

MPD+AYHYDVTITPDRPKKFFR+AFKQFINT LPG+TVAFDGVKSCYMV++L NPVYEGD

Sbjct 365 MPDVAYHYDVTITPDRPKKFFRNAFKQFINTFLPGRTVAFDGVKSCYMVQKLTNPVYEGD 424

Query 435 VKIADSGSRQIQFRVSIKLTDNPEVELRSLKTYHNERVFDKPMRALQCIEVVLANDCHNK 494

VKIADSG+R+IQFRVSIK TDNPEVELRSLKTYHN+RVFDKPMRALQCIEVVLAN CH+K

Sbjct 425 VKIADSGNREIQFRVSIKPTDNPEVELRSLKTYHNDRVFDKPMRALQCIEVVLANACHDK 484

Query 495 GIRAGRSFFTQPDKTMDLDEGYELYTGLYQAAILGEQPYLNVDISHKSFPMPYDLITYLE 554

GIRAGRS+FT+P KTMDLDEGYELYTGLYQAAILGEQPYLNVDISHKSFPMPY L Y+E

Sbjct 485 GIRAGRSYFTRPPKTMDLDEGYELYTGLYQAAILGEQPYLNVDISHKSFPMPYGLTEYME 544

Query 555 SVLNCNRQSNLDPRNLQRLSKHLKNLKVVYNPPPSFGAGPRSYKVNDISREPAATLSFTT 614

VL N Q LDPR+ Q L HL+NLKVVY PP FGA PRSYKVND+SREPA+TLSFT

Sbjct 545 KVLRINLQDRLDPRSFQSLCTHLRNLKVVYTPPAVFGAAPRSYKVNDVSREPASTLSFTL 604

Query 615 DNGEKFTVQKYFQSRGYNLRYPNLNCVVAGSTIRPNYFPMELCSIEAGQAIRRKDGSRQV 674

+ GEK TVQKYF+ RGY LRYPNL CVVAGSTI+PNY P+ELCSIEAGQA++RKDGSRQV

Sbjct 605 EGGEKLTVQKYFEGRGYRLRYPNLPCVVAGSTIKPNYLPIELCSIEAGQALKRKDGSRQV 664

Query 675 QKMIRFAATSTDERKRKIMQKLAYFNINADRLVQAFGISVGEQFIKVPMRLLKAPAIEYH 734

QKMIRFAATSTDERKRKIM KL YF N D LVQ FGI +G++FI VPMRLLKAP+IEY

Sbjct 665 QKMIRFAATSTDERKRKIMDKLHYFMHNTDHLVQGFGIRIGDRFITVPMRLLKAPSIEYR 724

Query 735 SSKYVEPRNGSWRNLPFLETGAAFKKSGHKWAIIYTPSRFLKYPTLMDLANMLYNGAKRM 794

SSK++EPRNGSWRNLPFL+TG A +++GHKWAIIY+PSRFL++ TL DL++MLYN AKRM

Sbjct 725 SSKFIEPRNGSWRNLPFLQTGIASQQTGHKWAIIYSPSRFLRHATLQDLSSMLYNSAKRM 784

Query 795 GINLDAQKDIKETGNIVATLEEYKRNDYDLVIVVIPGFGTSYADIKQKAELVCGLLTQCI 854

INLD ++DI+ET NIV TLEEYK+ +YDLV+VVIPG+GT+YADIKQKAELVCGLLTQCI

Sbjct 785 NINLDEKRDIQETKNIVQTLEEYKKKNYDLVVVVIPGYGTTYADIKQKAELVCGLLTQCI 844

Query 855 KEQTLNRGVNDMLISNLLLKVNSKLNGSNHKISANHHVVLDHVMFMGADVTHPSPDQNNI 914

KEQTLNRGVND L+SNLLLKVNSKLNG+NHKI+AN+HVVLDH MF+GADVTHPSPDQ NI

Sbjct 845 KEQTLNRGVNDQLVSNLLLKVNSKLNGANHKIAANNHVVLDHAMFLGADVTHPSPDQRNI 904

Query 915 PSVVGVAASHDLNGSVYNMQYRLQEPAKEEIVDMRTIASHHLRVYFQKQKCYPNNIIYYR 974

PSVVGVAASHDLNG+ YNMQYRLQE KEEIVDM++I HHL+VYFQKQ YPN IIYYR

Sbjct 905 PSVVGVAASHDLNGACYNMQYRLQESTKEEIVDMQSIIRHHLKVYFQKQNRYPNYIIYYR 964

Query 975 DGVSDGQFQKVEMLELGAIRAVCKELRITPKITCIIVVKRHHTRFFPTKPTGDKWNNVLP 1034

DGVSDGQFQKVE+LELGAIRAVCKELRI+PKITC+IVVKRHHTRFFPTKPTGDKWNNVLP

Sbjct 965 DGVSDGQFQKVEILELGAIRAVCKELRISPKITCVIVVKRHHTRFFPTKPTGDKWNNVLP 1024

Query 1035 GTVVDQKIVHPNETQFFMVSHQSIQGTAKPTRYNVIVDDAKMSMDDLQKMTNNLCYMFPR 1094

GTVVDQKIVHP ETQFFMVSHQSIQGTAKPTRYNVIVDDAKM+MDDLQKMTNNLCYMFPR

Sbjct 1025 GTVVDQKIVHPGETQFFMVSHQSIQGTAKPTRYNVIVDDAKMTMDDLQKMTNNLCYMFPR 1084

Query 1095 CNRAVSYPAPAYLAHLVAARGRVYIDGPPLRRALPEEYKKRLINERFMNTTPMFFV 1150

CNRAVSYPAPAYLAHLVAARGRVYI+GPPLRR L EEY KRLINE FMNTTPMFFV

Sbjct 1085 CNRAVSYPAPAYLAHLVAARGRVYIEGPPLRRTLREEYNKRLINENFMNTTPMFFV 1140

**R2D2**

**>lcl|ORF1**

MFKFAFNVDIDNDNPFSRKSNEKELNLIPTTSQNELNNEPEEWYEAENIP

ISIETLENLDVYKLNARILNIGDISIKHIMTKFLLDHITGNSEDNKGISK

AEQKHSDLIPGIYEGGAKIWECTEDLLQYLVKTFKPQEWRGKRVLDLGCG

AGLLGIYAYKCGSTVHFQDYNKDVLTQITIPNVLLNVARVKSDVDKSGIS

NAEQIQRNEIDALSKSLQFYSGDWRKYSDLTSQGTTDKFDFILTSETIYN

PKNQQKLLETLYRKLSPGGVVLVAAKTYYFGVGGGLRQFEDLITADKRFQ

CKIVWTSTDGVGREILELSLNKQ

**Graphical representation - ORF**


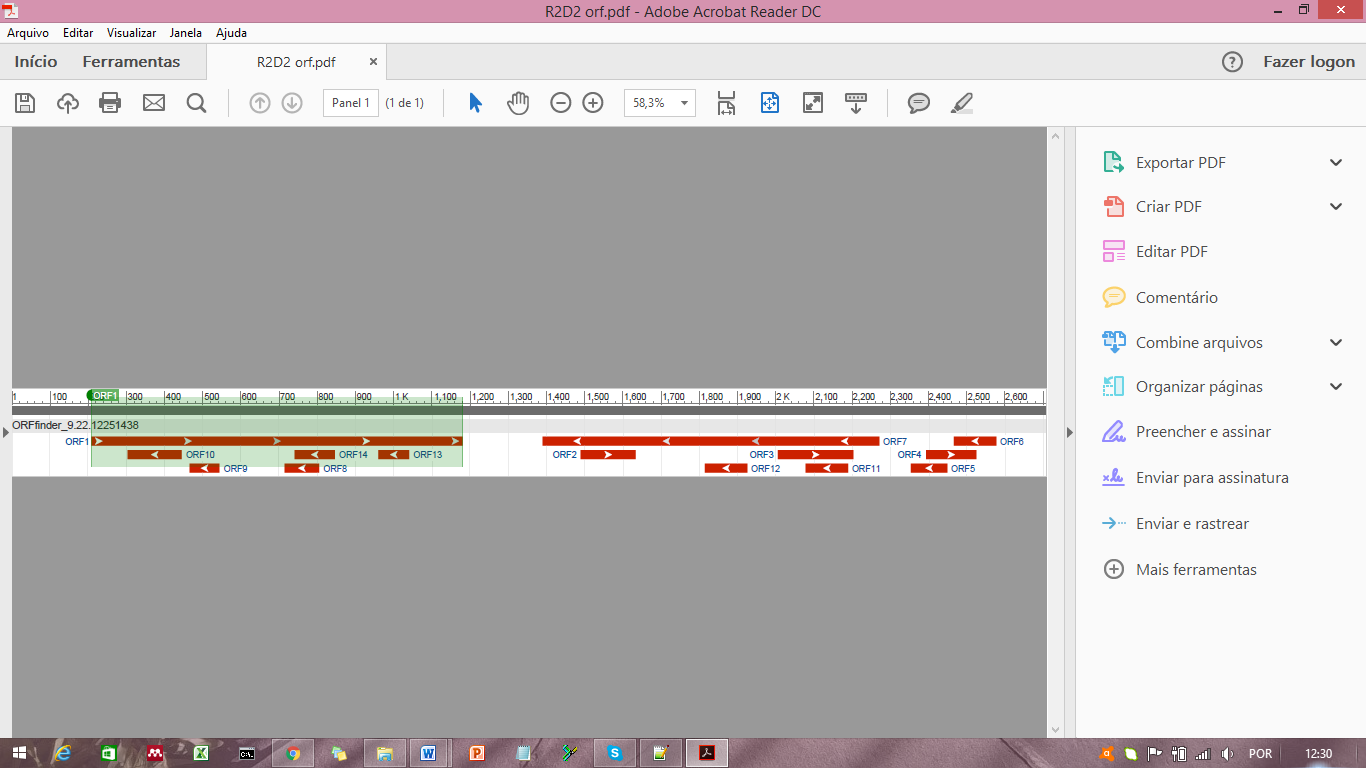


**BlastP (Non-redundant NCBI sequences)**

PREDICTED: histidine protein methyltransferase 1 homolog [Rhagoletis zephyria]

Sequence ID: [XP_017469335.1](https://www.ncbi.nlm.nih.gov/protein/1048016618?report=genbank&log$=protalign&blast_rank=1&RID=WAB2WEY2014) Length: 325 Number of Matches: 1

Range 1: 1 to 325

Score: 461 bits(1186)

E-value: 5e-161

Identities: 230/328(70%)

Query 1 MFKFAFNVDIDNDNPFSRKSNEKELNLIPTTSQNELNNEPEEWYEAENIPISIETLENLD 60

MFKFAFNV+IDN+NPFS E + TS+N + PEEWY AE + IS E LENLD

Sbjct 1 MFKFAFNVEIDNENPFSVNIKESGSD---QTSENVGKDGPEEWYTAEKVVISSEILENLD 57

Query 61 VYKLNARILNIGDISIKHIMTKFLLDHITGNSEDNKGISKAEQKHSDLIPGIYEGGAKIW 120

VYKLNA LN+GD+ I H++T FLL HIT NS DNK ISKAEQ HSDLIPG+YEGGAKIW

Sbjct 58 VYKLNATTLNVGDLPIHHVVTGFLLQHITSNSGDNKDISKAEQTHSDLIPGVYEGGAKIW 117

Query 121 ECTEDLLQYLVKTFKPQEWRGKRVLDLGCGAGLLGIYAYKCGSTVHFQDYNKDVLTQITI 180

ECTEDLL+YL K FKPQ WRGKRVLDLGCGAGLLGIYAY+CG+ VHFQDYNKDVLTQITI

Sbjct 118 ECTEDLLKYLAKNFKPQSWRGKRVLDLGCGAGLLGIYAYRCGAIVHFQDYNKDVLTQITI 177

Query 181 PNVLLNVARVKSDVDKSGISNAEQIQRNEI--DALSKSLQFYSGDWRKYSDLTSQGT--- 235

PNVLLNV VK D S +A + + + + D L K+++FYSGDW KYS+LTS+

Sbjct 178 PNVLLNVVDVKPAKDNSNSEDATESKVDLVNTDDLGKNVEFYSGDWSKYSELTSESVECK 237

Query 236 TDKFDFILTSETIYNPKNQQKLLETLYRKLSPGGVVLVAAKTYYFGVGGGLRQFEDLITA 295

+DKFD+IL SETIYNPKNQQKLL+T Y KL P GVVLVAAKTYYFGVGGGLRQFE LI

Sbjct 238 SDKFDYILASETIYNPKNQQKLLDTFYVKLKPDGVVLVAAKTYYFGVGGGLRQFETLIAN 297

Query 296 DKRFQCKIVWTSTDGVGREILELSLNKQ 323

DKRFQ +VWTSTDGVGREILEL L++Q

Sbjct 298 DKRFQSNVVWTSTDGVGREILELKLSEQ 325

**Argonaute-3**

**>lcl|ORF1**

MSARGRGFLLSLKVDKDTTDGEGSLKDSGLGSRSLNSGGVEFRRVGRGKL

LDDLASSCTNMTLEGRSSDDTNQGNTTSTTDSSKPLSGGRGRANVFKNLF

RDEKPEPQLDAKPTPVPVAVPDVITKGNTVTVPVSMPVAVPKAEVIQEVM

PSHIYNPEVKHGSKGMPVRLACNYIRLSSDPEKGVFVYEVRFHPPVDSLS

LRMKYLNEHRDKFGGTKTFDGVTLYLPILLKDKLTTFISKNIADDSDIEI

RILFKRKEALKNCIHLYNVLFDRVMKTLNYVRFDRKQFDPTAPKIIPQAK

LEVWPGYVTAIDEYEGGLMLCCDVSHRLLCQKTVLETLVEIYRSNTALFQ

ENAKKYLLGSVVITRYNNRTYRIDDICFDKNPKSTFQTKQAELSYIDYYR

QSHNILIKDETQPLIISIKKQKTADKQAAEDLIVCLIPELCYLTGLRDEI

RSDYKLMREIATFTRVSPNQRLLGLEKFFNNINKCPEAQNILQSWGLTLK

NAHECVNGRQFEEEQILFAKKQFSAGINADFSKYVGNNEVLEVVHLTNWL

LIHCKNDTRCAKNFCEHIERNSRALGIRVDKPKIITLDNDRVDTFVRALR

LNIDGQTQIVVCISPTNRDDRYAAIKKVCCAEIPVPSQVINARTLLNDAK

NRSIVVKIMLQMNCKLGGSLWAVKIPFKNVMICGIDSYHDAAQKGNSVAA

FVASLNSNYTKWYSKAVIQGKREEIVNGLCASFTAAVTRFHRENGRFPDN

IIIYRDGVGDGQLPLCSGHEIPQLEIACKRAFKDYTVKITFIVIQKRINT

RYFAMNGTNADNPPPGTVVDNSITRSKMYDFYLVSQAVRQGTVTPSHYIV

LRDDAKYSPDIIQRLTYKLCFMYYNWPGTIRIPACCQYAHKMAYLIGQSI

RRATSEELSDRLFYL

**Graphical representation - ORF**


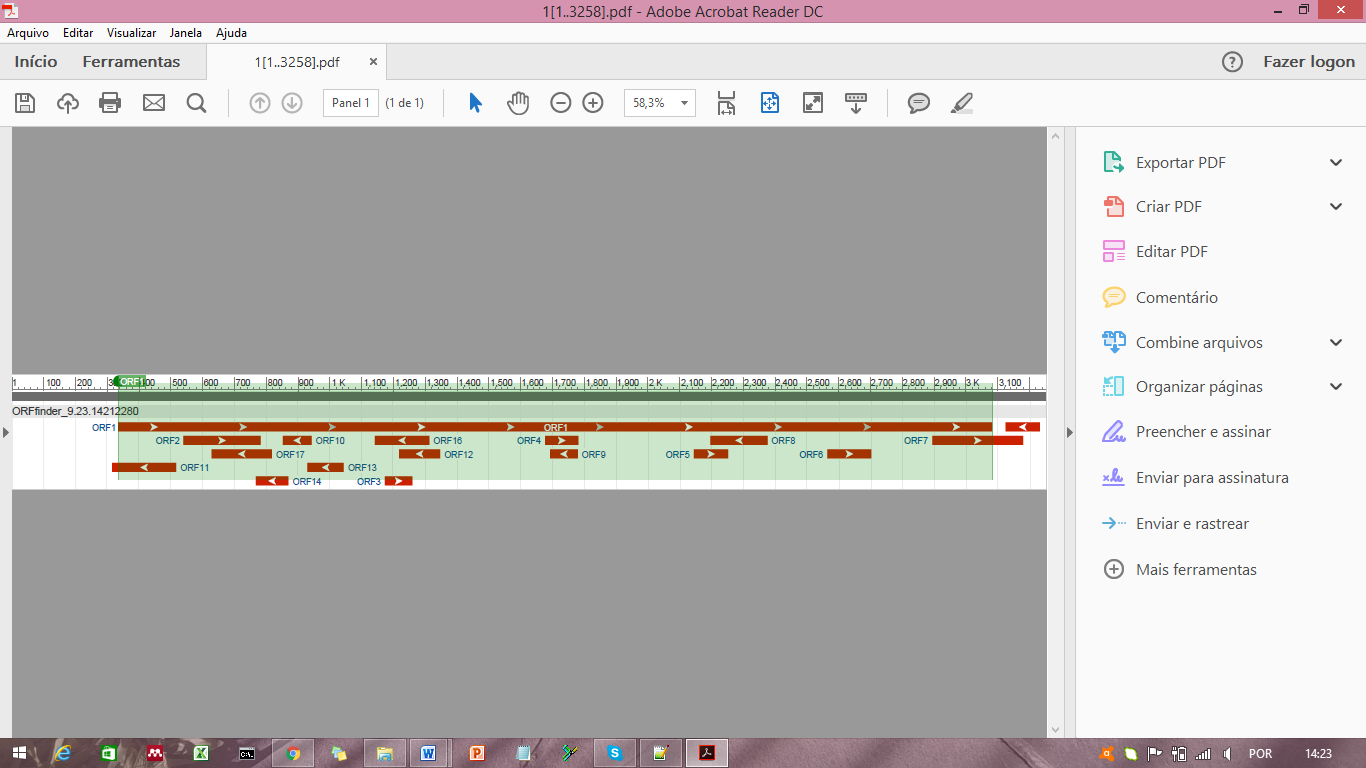


**BlastP (Non-redundant NCBI sequences)**

protein argonaute-3 [Ceratitis capitata]

Sequence ID: [XP_004536573.1](https://www.ncbi.nlm.nih.gov/protein/499009467?report=genbank&log$=protalign&blast_rank=1&RID=WD641EDG015) Length: 909 Number of Matches: 1

Range 1: 1 to 909

Score: 1655 bits(4285)

E-value: 0.0

Identities: 850/918(92%)

Query 1 MSARGRGFLLSLKVDKDTTDGEGSLKDSGLGSRSLNSGGVEFRRVGRGKLLDDLASSCTN 60

M+ RGRGFL SLKVDKD+T E +L+DSGLG++SL S G E+RR GRGKLLDDLASSC+N

Sbjct 1 MATRGRGFLFSLKVDKDSTGSESTLRDSGLGTKSLQSVG-EYRRFGRGKLLDDLASSCSN 59

Query 61 MTLEGRSSDDTNQGNTTSTTDSSKPLSGG---RGRANVFKNLFRDEKPEPQLDAKPTPVP 117

MTLE RSSDDTNQGN TSTTDS+KP + G RGRA+VFK+LF +EKPEP+LD KP VP

Sbjct 60 MTLEVRSSDDTNQGNITSTTDSNKPSTSGGRGRGRASVFKSLFSEEKPEPKLDTKPISVP 119

Query 118 VAVPDVITKGNTVTVPVSMPVAVPKAEVIQEVMPSHIYNPEVKHGSKGMPVRLACNYIRL 177

+P V P + E ++V+PS +YNPEVKHGSKG PVRLACNYIRL

Sbjct 120 FPMP--------VKKSEQKPESTLDPEPAEDVIPSDVYNPEVKHGSKGKPVRLACNYIRL 171

Query 178 SSDPEKGVFVYEVRFHPPVDSLSLRMKYLNEHRDKFGGTKTFDGVTLYLPILLKDKLTTF 237

SSDPEKGV+VYEVRFHPPVDSL+LRMKYLNEHRDKFGGTKTFDGVTLYLPILLKDKLTT+

Sbjct 172 SSDPEKGVYVYEVRFHPPVDSLNLRMKYLNEHRDKFGGTKTFDGVTLYLPILLKDKLTTY 231

Query 238 ISKNIADDSDIEIRILFKRKEALKNCIHLYNVLFDRVMKTLNYVRFDRKQFDPTAPKIIP 297

ISK++AD++DIEIRILFKRKEALKNCIHLYNVLFDRVMKTLNYVRFDRKQFDPTAPKIIP

Sbjct 232 ISKSVADNTDIEIRILFKRKEALKNCIHLYNVLFDRVMKTLNYVRFDRKQFDPTAPKIIP 291

Query 298 QAKLEVWPGYVTAIDEYEGGLMLCCDVSHRLLCQKTVLETLVEIYRSNTALFQENAKKYL 357

QAKLEVWPGYVTAIDEY+GGLMLCCDVSHRLLCQKTVLETL EIYRSN+ LFQENAKKYL

Sbjct 292 QAKLEVWPGYVTAIDEYKGGLMLCCDVSHRLLCQKTVLETLTEIYRSNSNLFQENAKKYL 351

Query 358 LGSVVITRYNNRTYRIDDICFDKNPKSTFQTKQAELSYIDYYRQSHNILIKDETQPLIIS 417

LGSVVITRYNNRTYRIDDICFDK+PKS FQTK AELSYIDYYRQSHNILIKDETQPLIIS

Sbjct 352 LGSVVITRYNNRTYRIDDICFDKSPKSKFQTKNAELSYIDYYRQSHNILIKDETQPLIIS 411

Query 418 IKKQKTADKQAAEDLIVCLIPELCYLTGLRDEIRSDYKLMREIATFTRVSPNQRLLGLEK 477

IKKQKTADKQAAEDL++CLIPELCYLTGLRD+IRSDYKLMREI TFTRVSPNQRLL LEK

Sbjct 412 IKKQKTADKQAAEDLVICLIPELCYLTGLRDDIRSDYKLMREIVTFTRVSPNQRLLALEK 471

Query 478 FFNNINKCPEAQNILQSWGLTLKNAHECVNGRQFEEEQILFAKKQFSAGINADFSKYVGN 537

FF N+N CPEAQ+ILQ WGLTLKN+HE +NGRQF EEQILFAKKQF AG+NADFSKYV N

Sbjct 472 FFTNVNNCPEAQSILQCWGLTLKNSHETLNGRQFNEEQILFAKKQFPAGVNADFSKYVCN 531

Query 538 NEVLEVVHLTNWLLIHCKNDTRCAKNFCEHIERNSRALGIRVDKPKIITLDNDRVDTFVR 597

NEVLEVVHLTNW+L+HCK+DTRCAKNF EH++RNSRALGIRVDKPK++TL+NDR+DT+V+

Sbjct 532 NEVLEVVHLTNWILMHCKSDTRCAKNFYEHVDRNSRALGIRVDKPKMVTLENDRIDTYVK 591

Query 598 ALRLNIDGQTQIVVCISPTNRDDRYAAIKKVCCAEIPVPSQVINARTLLNDAKNRSIVVK 657

ALR +IDGQTQIVVCISPTNRDDRYAAIKKVCCAEIP+PSQVIN+RTLLN+AKNRSIV K

Sbjct 592 ALRTHIDGQTQIVVCISPTNRDDRYAAIKKVCCAEIPIPSQVINSRTLLNEAKNRSIVQK 651

Query 658 IMLQMNCKLGGSLWAVKIPFKNVMICGIDSYHDAAQKGNSVAAFVASLNSNYTKWYSKAV 717

IMLQMNCKLGGSLWAVKIPFKNVMICGIDSYHDAAQK NSVAAFVASLNSN+TKWYSKAV

Sbjct 652 IMLQMNCKLGGSLWAVKIPFKNVMICGIDSYHDAAQKSNSVAAFVASLNSNFTKWYSKAV 711

Query 718 IQGKREEIVNGLCASFTAAVTRFHRENGRFPDNIIIYRDGVGDGQLPLCSGHEIPQLEIA 777

IQGKREEIVNGLC+SF AAVTRFHRENGR+PD+IIIYRDGVGDGQLPLCSGHEIPQLE A

Sbjct 712 IQGKREEIVNGLCSSFIAAVTRFHRENGRYPDSIIIYRDGVGDGQLPLCSGHEIPQLEAA 771

Query 778 CKRAFKDYTVKITFIVIQKRINTRYFAMNGTNADNPPPGTVVDNSITRSKMYDFYLVSQA 837

CKRAF DYTVKITF+V+QKRINTRYFA+NG+N +NPPPGTVVD+ ITRSKMYDFYLVSQ

Sbjct 772 CKRAFNDYTVKITFVVVQKRINTRYFAINGSNVENPPPGTVVDDCITRSKMYDFYLVSQT 831

Query 838 VRQGTVTPSHYIVLRDDAKYSPDIIQRLTYKLCFMYYNWPGTIRIPACCQYAHKMAYLIG 897

+RQGTVTPSHYIVLRDD+KYSPDIIQRLTYKLCFMYYNWPGTIRIPACCQYAHKMAYLIG

Sbjct 832 IRQGTVTPSHYIVLRDDSKYSPDIIQRLTYKLCFMYYNWPGTIRIPACCQYAHKMAYLIG 891

Query 898 QSIRRATSEELSDRLFYL 915

QSIRRATSEELSDRLFYL

Sbjct 892 QSIRRATSEELSDRLFYL 909

**Piwi**

**>lcl|ORF13**

MSDDHSRGRRRPSREQRYSRSRSPLDTYSSNTGGGDSQRRRRERQSPDES

RPPKVYKKEPVSPDRSSSSSRNGNGRREPRDQPSTSGAAGGGGFGADGDG

GDSADHGMKKERYEIVHTRPSDIQSKIGSGGTPIILQANYYRLLTKPTWR

IFQYHVDFTPKVELRRVRGGILSEHRSTLGGYLYDGTKMFTSCKLPEDKT

IIHAKSKVGDSYTIVIKYVGVISMTEWQSLQILNLILRRAMEGLKLQLVG

RNFYDAIAKIDLREYRLQLWPGYQTSIRQHESDILLCAEIAHKVMRTETV

YDILMKCTETARDYQEEFRRHVLGLTVLTDYNNKTYRINDVDFNKNPSKT

FSCKEKDVSFIDYYYQKYHIRIRDPKQPLLISKPKERAMRTGGSDIIILI

PELCRPTGLTDTMRNNFQLMRAMADHTRMNPDRRIDRLRIFNNRLQQTDA

SIQVLNDWNMTLDRHLVELNGRVLEPQRIVFSEHKKASAGEQADWTRLFR

DNGLFTTPSRGLDRWSVIATNRNSRELRNFVESLIRAAGGMQMRINRPRE

VMLYDDRNHSYIQAMEDCSRHDPQLILCLVPNNNAERYASIKKKGCLERA

IPTQVITQKSANNQRGLMSIATKVAIQINCKLGYTPWMIDLPLSGLMTIG

YDVAKSTRDRSKAFGALVASMDMKTNATFYSTVAECSSHDVLANSLWPMM

TKALRQYRKEHDGKLPTRILFYRDGVGEGSLRQVYEHEVKDVVEKLEQEY

KRVGSEKPPMFAYVVVSKNINTRLFARGRNPPPGTIVDDVVTCPERYDFF

LVSQSVRQGTVSPTSYNIVYSNIRLTPDQMQLLTYKMTHLYYNWSGTTRV

PAVCQYAKKLATLVATSLYQPPQNALEKKLYYL

**Graphical representation - ORF**


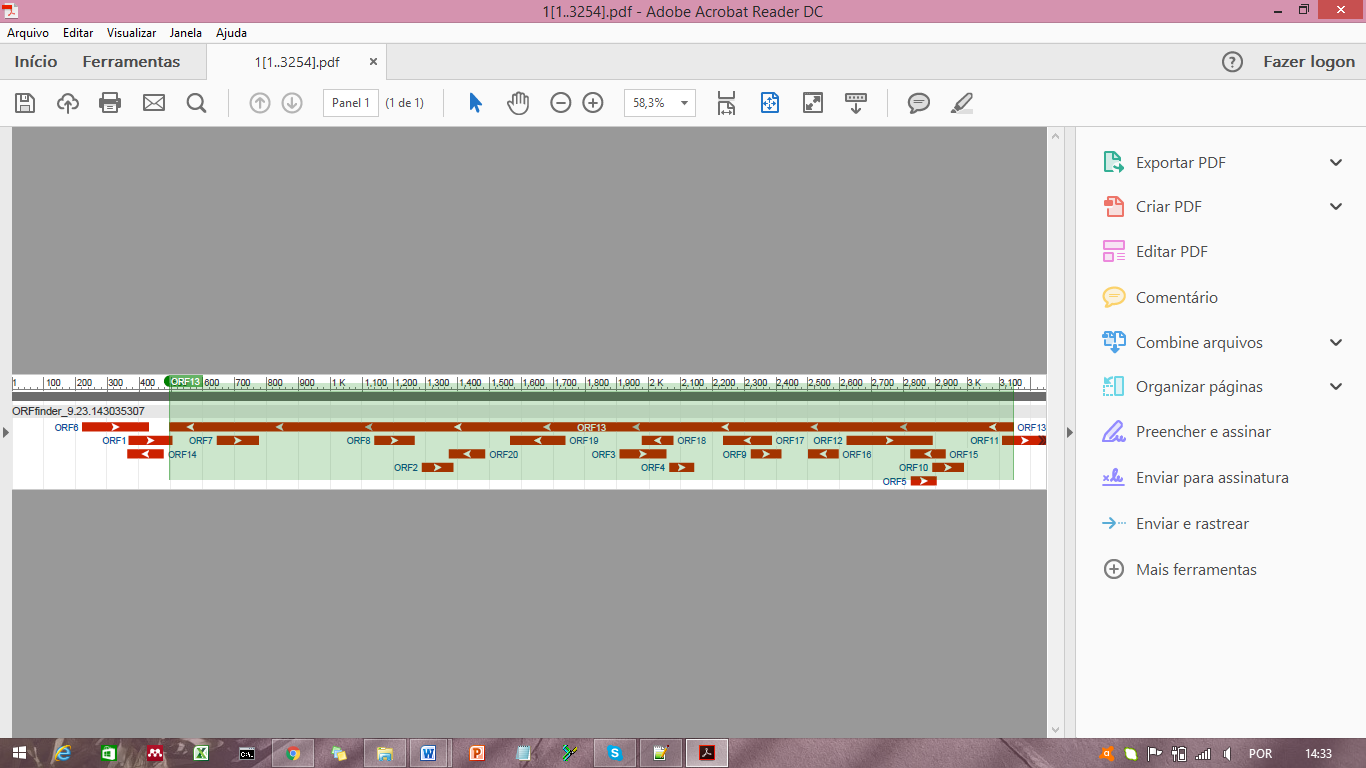


**BlastP (Non-redundant NCBI sequences)**

PREDICTED: protein piwi [Bactrocera oleae]

Sequence ID: [XP_014087635.1](https://www.ncbi.nlm.nih.gov/protein/929356820?report=genbank&log$=protalign&blast_rank=1&RID=WD6KMA4G015) Length: 880 Number of Matches: 1

Range 1: 1 to 880

Score: 1636 bits(4237)

E-value: 0.0

Identities: 783/884(89%)

Query 1 MSDDHSRGRRRPSREQRYSRSRSPLDTYSSNTGGGDSQRRRRERQSPDESRPPKVYKKEP 60

MS+D +RGRRRPSREQ+ SRSRSP +T S+ GGGDS RRRRER++ DE RP KVYKKEP

Sbjct 1 MSEDQNRGRRRPSREQQGSRSRSPQETLPSSVGGGDSHRRRREREASDEPRPSKVYKKEP 60

Query 61 VSPDRSSSSSRNGNGRREPRDQPSTSGAAGGGGFGADGDGGDSADHGMKKERYEIVHTRP 120

+SPDRSS+SSRN N R+E +QPSTS AA GG GD D D+ K+ERYEIVHTRP

Sbjct 61 ISPDRSSTSSRNENSRKEQHEQPSTSSAARSGG----GDSTDIGDNASKRERYEIVHTRP 116

Query 121 SDIQSKIGSGGTPIILQANYYRLLTKPTWRIFQYHVDFTPKVELRRVRGGILSEHRSTLG 180

SDIQ+K+G GTPI LQANYYRLLTKPTWRIFQYHVDF+P +ELRRVRGG+LSEHR TLG

Sbjct 117 SDIQTKMGKVGTPITLQANYYRLLTKPTWRIFQYHVDFSPHIELRRVRGGMLSEHRETLG 176

Query 181 GYLYDGTKMFTSCKLPEDKTIIHAKSKVGDSYTIVIKYVGVISMTEWQSLQILNLILRRA 240

GYLYDGTK+FTSCKL E++T+I A SK GDSYTIVIKYVGVISMTEWQSLQILNLILRR+

Sbjct 177 GYLYDGTKLFTSCKLSEERTVIRAVSKFGDSYTIVIKYVGVISMTEWQSLQILNLILRRS 236

Query 241 MEGLKLQLVGRNFYDAIAKIDLREYRLQLWPGYQTSIRQHESDILLCAEIAHKVMRTETV 300

MEGLKLQLVGRNFYDA+AKIDLREYRLQLWPGYQTSIRQHE DILLCAEIAHKVMRTETV

Sbjct 237 MEGLKLQLVGRNFYDALAKIDLREYRLQLWPGYQTSIRQHERDILLCAEIAHKVMRTETV 296

Query 301 YDILMKCTETARDYQEEFRRHVLGLTVLTDYNNKTYRINDVDFNKNPSKTFSCKEKDVSF 360

YDIL +CTETARDY+EEFRR+VLGLTVLTDYNNKTYRINDVDF+KNP+KTF CKE +VSF

Sbjct 297 YDILKRCTETARDYEEEFRRNVLGLTVLTDYNNKTYRINDVDFSKNPTKTFKCKENEVSF 356

Query 361 IDYYYQKYHIRIRDPKQPLLISKPKERAMRTGGSDIIILIPELCRPTGLTDTMRNNFQLM 420

IDYYYQKYHIRIRD KQPLLISK K+RA+R G SDIIILIPELCRPTGLTDTMRNNFQLM

Sbjct 357 IDYYYQKYHIRIRDQKQPLLISKAKDRALRGGSSDIIILIPELCRPTGLTDTMRNNFQLM 416

Query 421 RAMADHTRMNPDRRIDRLRIFNNRLQQTDASIQVLNDWNMTLDRHLVELNGRVLEPQRIV 480

RAMADHTRMNPDRRI+RLRIFN RLQQT+AS+QVLNDWNM LDRHLVELNGRVLEPQRIV

Sbjct 417 RAMADHTRMNPDRRIERLRIFNQRLQQTEASVQVLNDWNMALDRHLVELNGRVLEPQRIV 476

Query 481 FSEHKKASAGEQADWTRLFRDNGLFTTPSRGLDRWSVIATNRNSRELRNFVESLIRAAGG 540

FSEH+K SAG QADWTR FR+NGLFTTPSRGL+RWSVIATNRNSRELRNFVESLIRAA G

Sbjct 477 FSEHRKESAGVQADWTRYFRENGLFTTPSRGLERWSVIATNRNSRELRNFVESLIRAASG 536

Query 541 MQMRINRPREVMLYDDRNHSYIQAMEDCSRHDPQLILCLVPNNNAERYASIKKKGCLERA 600

MQMRI+RPREVMLYDDRNHSYIQAMEDCSR DPQLILCLVPNNNAERYASIKKKGCLERA

Sbjct 537 MQMRISRPREVMLYDDRNHSYIQAMEDCSRQDPQLILCLVPNNNAERYASIKKKGCLERA 596

Query 601 IPTQVITQKSANNQRGLMSIATKVAIQINCKLGYTPWMIDLPLSGLMTIGYDVAKSTRDR 660

IPTQVITQKSA NQRGLMSIATKVAIQINCKLGYTPWMIDLPLSGLMTIGYDVAKSTRDR

Sbjct 597 IPTQVITQKSAGNQRGLMSIATKVAIQINCKLGYTPWMIDLPLSGLMTIGYDVAKSTRDR 656

Query 661 SKAFGALVASMDMKTNATFYSTVAECSSHDVLANSLWPMMTKALRQYRKEHDGKLPTRIL 720

SKAFGALVASMDMKTNATFYSTVAECSSHDVLANSLWPMMTKALRQYR+EH+ KLPTRIL

Sbjct 657 SKAFGALVASMDMKTNATFYSTVAECSSHDVLANSLWPMMTKALRQYRREHENKLPTRIL 716

Query 721 FYRDGVGEGSLRQVYEHEVKDVVEKLEQEYKRVGSEKPPMFAYVVVSKNINTRLFA-RGR 779

FYRDGVGEGSLRQVYEHEVKDVVEKLEQEYKRVGSEKPPMFAYVVVSK+INTR F RG+

Sbjct 717 FYRDGVGEGSLRQVYEHEVKDVVEKLEQEYKRVGSEKPPMFAYVVVSKSINTRFFMNRGQ 776

Query 780 NPPPGTIVDDVVTCPERYDFFLVSQSVRQGTVSPTSYNIVYSNIRLTPDQMQLLTYKMTH 839

NP PGTIVDDVVT PERYDFFLVSQSVRQGTVSPTSYNIVYSNIRLTPDQMQLLTYKMTH

Sbjct 777 NPTPGTIVDDVVTLPERYDFFLVSQSVRQGTVSPTSYNIVYSNIRLTPDQMQLLTYKMTH 836

Query 840 LYYNWSGTTRVPAVCQYAKKLATLVATSLYQPPQNALEKKLYYL 883

LYYNWSGTTRVPAVCQYAKKLATLVATSLYQPPQNALEKKLYYL

Sbjct 837 LYYNWSGTTRVPAVCQYAKKLATLVATSLYQPPQNALEKKLYYL 880

**Aubergine**

**>lcl|ORF2**

MDKLTNAGRSRGRGRASNPHQPRGDARSRRTGQPGLQAEASGGPGPSRNL

PQAPPTSAWGQPLPGVARGSTIAPKPSCSTAPVQQRPAAPVMQGRATAHR

GAPIGDAPVVKEDSRGAVRGKRVLHEVVSSRPSTCITKTGCTGKKVVIQT

NYFRVLKKPQWSIHQYRVDFAPDVDMIRLRRAYLAQHKETFGGYIFDGTM

LFCTKYLEKPQMELLTKNREGETIQIKIKHVGQLEVTDSQQLQVLNLILR

RAMSGLNLELVGRSFFDPKAKHCLNSFYLELWPGYQTSIRQHEQDILLCA

EIAHKVMRTDTLYKILQDCVGKPDFHDAFKREVVGTIILTDYNNKTYRIN

DVDFQQSPKSKFATKEGEISYIDYYKKRYNIKISDANQPLLMSRPTERDI

RGGIDDFIMLIPELSRATGLTNAMRSNFSLMRAMSEFTRLAPERRIERLR

VFNRRLNQAPESVQVLESWSMRLDTNLVEVPGRIIPANRIVFGNNKRYDC

NEFADWTREFRNNSMYKHVDIKRWYVITPSRNLREAQNFVQMCIRAANGM

RMGIAEPIYQQISDDRSGSYSQAINTVSTGDPQIVMVVLVSANEEKYGCV

KKKCCVDRPMPSQVVTLRTIAPRGDKAAGLMSIATKVVIQMNAKLMGAPW

LTEIPVSGLMTVGFDVCHSPREKTKSYGALVATMDLKSKPIYFSSVSEHV

KGQELSNEIALRMGYALKAYQQEHGMLPKKILFYRDGVGDGQLHQVFSTE

VKFLIERLNKMYSDYEKKPTICPLAFIVVSKRINTRYFINGRNPPPGTVV

DDVITLPERYDFFLVSQSVRQGTVSPTSYNVIYDTMGFDADKLQMLTYKM

THLYYNWSGTCRVPAVCQYAHKLAFLVAESIHRLPSNALEKQLYFL

**Graphical representation – ORF**


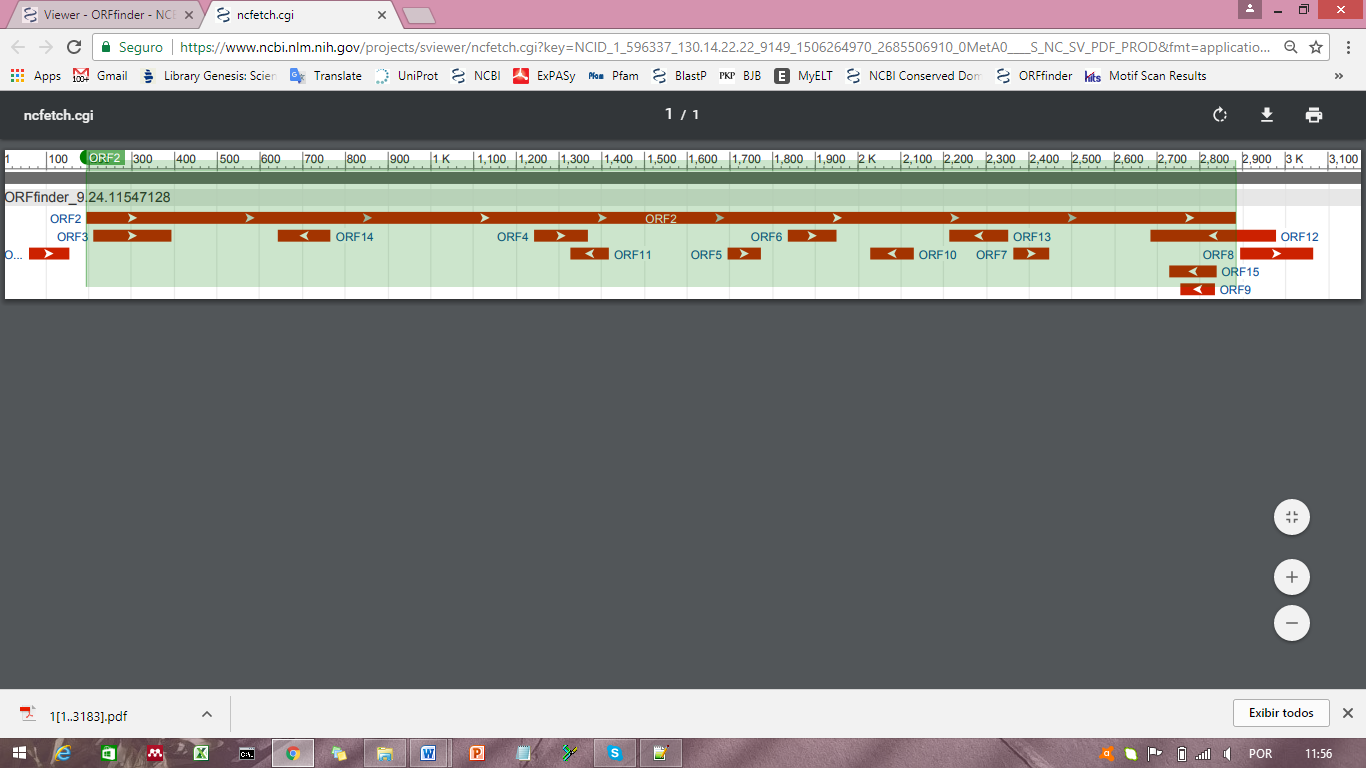


**BlastP (Non-redundant NCBI sequences)**

PREDICTED: protein aubergine [Rhagoletis zephyria]

Sequence ID: [XP_017472383.1](https://www.ncbi.nlm.nih.gov/protein/1048001812?report=genbank&log$=protalign&blast_rank=1&RID=WFJ0XZBJ015)Length: 908Number of Matches: 1

Range 1: 1 to 908

Score: 1574 bits(4075)

E-value: 0.0

Identities: 741/909(82%)

Query 1 MDKLTNAGRSRGRGRASNPHQPRGDARSRRTGQ-------PGLQAEASGGPGPSRNLPQ- 52

MDKLT+AGRSRGRGRA+NP QPRGDAR+RR GQ PG Q E PGP R P

Sbjct 1 MDKLTSAGRSRGRGRATNPQQPRGDARNRRVGQQGETSRAPGQQGEPLRAPGPPRGYPTT 60

Query 53 APPTSAWGQPLPG-----VARGSTIAPKPSCSTAPVQQRPAAPVMQGRATAHRGAPIGDA 107

AP TSAWGQPLP VARGST+ +PSCSTAP+QQR AAPVMQGRAT HR P+G+A

Sbjct 61 APTTSAWGQPLPASRGSAVARGSTVPQQPSCSTAPIQQRAAAPVMQGRATTHREIPVGEA 120

Query 108 PVVKEDSRGAVRGKRVLHEVVSSRPSTCITKTGCTGKKVVIQTNYFRVLKKPQWSIHQYR 167

PVV+ED+RGAVRGKRVL EV++SRP+TC K+G TGKKVVIQTNYFRVLKKPQWSIHQYR

Sbjct 121 PVVREDTRGAVRGKRVLTEVIASRPATCENKSGKTGKKVVIQTNYFRVLKKPQWSIHQYR 180

Query 168 VDFAPDVDMIRLRRAYLAQHKETFGGYIFDGTMLFCTKYLEKPQMELLTKNREGETIQIK 227

VDFAPD+DM+RLRRAYL HK+ FGGYIFDGT+LFC KYLE Q+ELLTKNREGETIQIK

Sbjct 181 VDFAPDIDMVRLRRAYLYHHKDLFGGYIFDGTVLFCAKYLENKQIELLTKNREGETIQIK 240

Query 228 IKHVGQLEVTDSQQLQVLNLILRRAMSGLNLELVGRSFFDPKAKHCLNSFYLELWPGYQT 287

IKHVGQLEVTD+QQLQVLNLILRRAM GLNL+LVGRSFFDPKAKH L+S+ LELWPGYQT

Sbjct 241 IKHVGQLEVTDAQQLQVLNLILRRAMEGLNLQLVGRSFFDPKAKHSLDSYCLELWPGYQT 300

Query 288 SIRQHEQDILLCAEIAHKVMRTDTLYKILQDCVGKPDFHDAFKREVVGTIILTDYNNKTY 347

SIRQHE DILLCAEIAHKVMRTDT+YKILQ+C KPD+ + FKREVVGTI+LTDYNNKTY

Sbjct 301 SIRQHENDILLCAEIAHKVMRTDTVYKILQECFDKPDYQNVFKREVVGTIVLTDYNNKTY 360

Query 348 RINDVDFQQSPKSKFATKEGEISYIDYYKKRYNIKISDANQPLLMSRPTERDIRGGIDDF 407

R++DVDF SP SKF+TK+GEISY++YYKKRYNI + DA QPLL+SRPTER+IRGG ++F

Sbjct 361 RVDDVDFNSSPMSKFSTKDGEISYMEYYKKRYNIHLKDAKQPLLVSRPTERNIRGGQNEF 420

Query 408 IMLIPELSRATGLTNAMRSNFSLMRAMSEFTRLAPERRIERLRVFNRRLNQAPESVQVLE 467

IMLIPELSR+TGLTNAMRSNF LMRAMSEFTRLAPERRIERL+VFNRRLNQAPESV++L

Sbjct 421 IMLIPELSRSTGLTNAMRSNFRLMRAMSEFTRLAPERRIERLKVFNRRLNQAPESVELLN 480

Query 468 SWSMRLDTNLVEVPGRIIPANRIVFGNNKRYDCNEFADWTREFRNNSMYKHVDIKRWYVI 527

SWSMRLDTNLVEVPGRIIP+ +IVFGNNKRY+CN++ADWTREFRNN+MYK+VD+KRWYVI

Sbjct 481 SWSMRLDTNLVEVPGRIIPSCKIVFGNNKRYECNDYADWTREFRNNAMYKNVDMKRWYVI 540

Query 528 TPSRNLREAQNFVQMCIRAANGMRMGIAEPIYQQISDDRSGSYSQAINTVSTGDPQIVMV 587

P RNLRE QNFVQMCIRAANGM+MGIAEP YQQ++DDR+G+YSQAINTV+ DPQ++MV

Sbjct 541 APGRNLREVQNFVQMCIRAANGMQMGIAEPRYQQMNDDRAGTYSQAINTVAAEDPQLLMV 600

Query 588 VLVSANEEKYGCVKKKCCVDRPMPSQVVTLRTIAPRGDKAAGLMSIATKVVIQMNAKLMG 647

VLVSA+EEKY C+KKKCCVDRP+PSQVVTLRTIAPRGDKA+GLMS+ATKVVIQMNAKLMG

Sbjct 601 VLVSAHEEKYSCIKKKCCVDRPVPSQVVTLRTIAPRGDKASGLMSVATKVVIQMNAKLMG 660

Query 648 APWLTEIPVSGLMTVGFDVCHSPREKTKSYGALVATMDLKSKPIYFSSVSEHVKGQELSN 707

APWLT+IP+SGLMTVGFDVCHS +EK KSYGALVATMDLKSKP YFS+VS+H+KGQELSN

Sbjct 661 APWLTDIPISGLMTVGFDVCHSAKEKNKSYGALVATMDLKSKPHYFSAVSQHMKGQELSN 720

Query 708 EIALRMGYALKAYQQEHGMLPKKILFYRDGVGDGQLHQVFSTEVKFLIERLNKMYSDYEK 767

EI + M YALKAY+ EHGMLPKKILFYRDGVGDGQLHQVF TEVKFL E+L+++Y +

Sbjct 721 EIGMNMTYALKAYRNEHGMLPKKILFYRDGVGDGQLHQVFHTEVKFLKEKLDEIYKN-AG 779

Query 768 KPTICPLAFIVVSKRINTRYFINGRNPPPGTVVDDVITLPERYDFFLVSQSVRQGTVSPT 827

+P CP+AFIVVSKRINTRYFINGRNPPPGTVVDD+ITLPERYDFFLVSQSV QGTVSPT

Sbjct 780 EPGPCPMAFIVVSKRINTRYFINGRNPPPGTVVDDIITLPERYDFFLVSQSVNQGTVSPT 839

Query 828 SYNVIYDTMGFDADKLQMLTYKMTHLYYNWSGTCRVPAVCQYAHKLAFLVAESIHRLPSN 887

SYNVIYDTMG DADK+QMLTYKMTHLYYNWSGTCRVPAVCQYAHKLAFLVAES+HRLP+N

Sbjct 840 SYNVIYDTMGLDADKIQMLTYKMTHLYYNWSGTCRVPAVCQYAHKLAFLVAESLHRLPNN 899

Query 888 ALEKQLYFL 896

ALEKQLYFL

Sbjct 900 ALEKQLYFL 908

**Zucchini**

**>lcl|ORF1**

MMENRVLRYTFYSLTTGISVVLLSEILYRGYLYVRERMWRRDSSDDEVNE

VIWTNELTQSCAAGHILKSPSERKSPTQENPSKGMVENGHITKLPVPSTS

PTCQNRFCASKNVGRLISLIDKSKHTIDLAMYTFTSYDLSQAFMRAIRRG

VSVRIISDKEMVYSSGSQIITLTKAGVAVRCPMTTMLMHHKFCLLDGPRR

ARTVLANVGKTYDSKTAKGIVMSGSLNWTMQGFGGNWENIVISSNKVLLE

QFEDEFERMWLTFAPKMST

**Graphical representation – ORF**


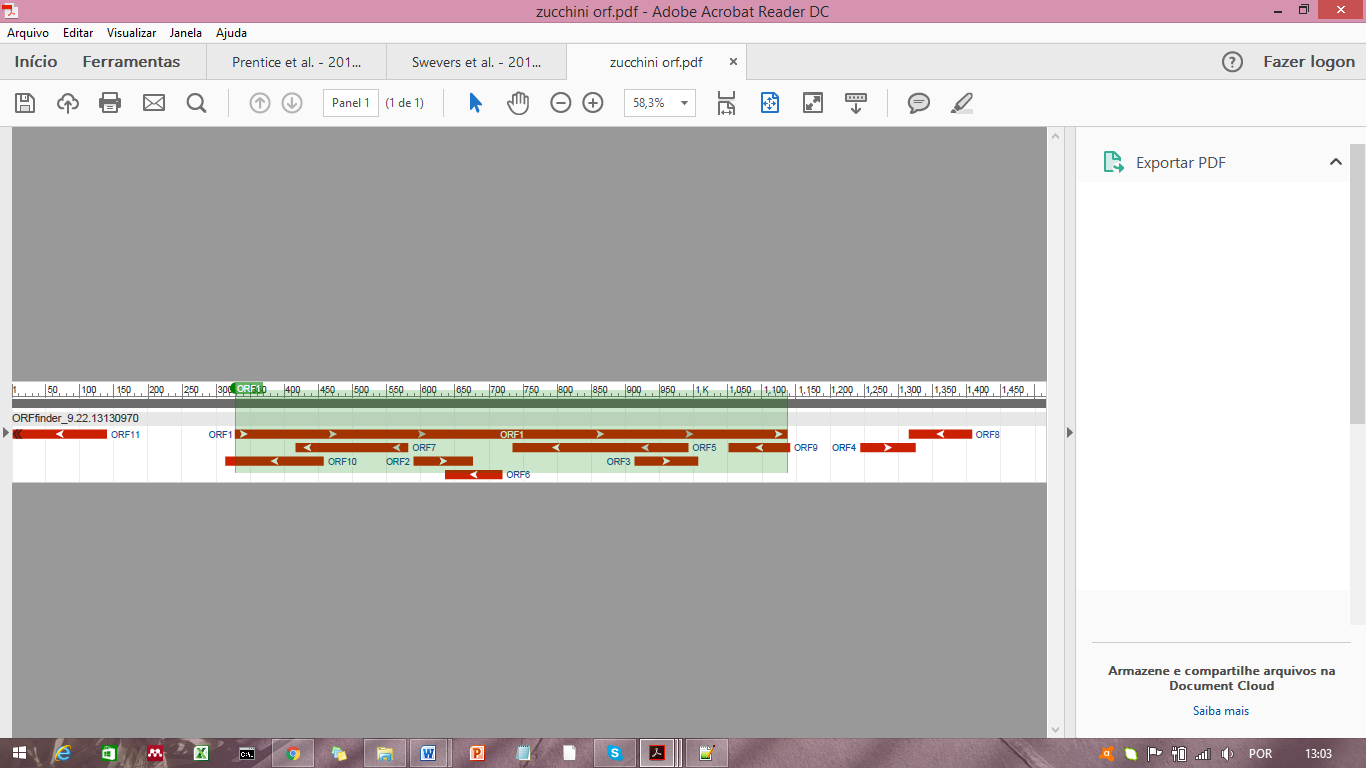


**BlastP (Non-redundant NCBI sequences)**

PREDICTED: mitochondrial cardiolipin hydrolase [Bactrocera latifrons]

Sequence ID: [XP_018793284.1](https://www.ncbi.nlm.nih.gov/protein/1098613508?report=genbank&log$=protalign&blast_rank=1&RID=WAD1J2RY014) Length: 269 Number of Matches: 1

Range 1: 1 to 269

Score: 502 bits(1292)

E-value: 5e-179

Identities: 229/269(85%)

Query 1 MMENRVLRYTFYSLTTGISVVLLSEILYRGYLYVRERMWRRDSSDDEVNEVIWTNELTQS 60

M+ENRV+RYTFYSLTTGISVVLLSEI YRG+LYVRER+WRRD+SDDE+ EVIWTNELTQS

Sbjct 1 MLENRVVRYTFYSLTTGISVVLLSEIFYRGFLYVRERLWRRDASDDEIAEVIWTNELTQS 60

Query 61 CAAGHILKSPSERKSPTQENPSKGMVENGHITKLPVPSTSPTCQNRFCASKNVGRLISLI 120

CA GHILKSP+ERKSPT E+P+KGMVENGH+TKLP P+ SP+CQNR+CA+ NVGRLI+LI

Sbjct 61 CAGGHILKSPAERKSPTYESPAKGMVENGHVTKLPTPNLSPSCQNRYCAANNVGRLIALI 120

Query 121 DKSKHTIDLAMYTFTSYDLSQAFMRAIRRGVSVRIISDKEMVYSSGSQIITLTKAGVAVR 180

DKSK+TIDLAMYTFTSYDLSQAFMRAIRRGVSVRIISDKEMVYSSGSQIITLTKAGVAVR

Sbjct 121 DKSKYTIDLAMYTFTSYDLSQAFMRAIRRGVSVRIISDKEMVYSSGSQIITLTKAGVAVR 180

Query 181 CPMTTMLMHHKFCLLDGPRRARTVLANVGKTYDSKTAKGIVMSGSLNWTMQGFGGNWENI 240

CPMTTMLMHHKFCLLDGP+RAR+VL N+GKT D + KG++MSGSLNWTMQGFGGNWENI

Sbjct 181 CPMTTMLMHHKFCLLDGPKRARSVLGNIGKTDDLRNVKGVLMSGSLNWTMQGFGGNWENI 240

Query 241 VISSNKVLLEQFEDEFERMWLTFAPKMST 269

VISSNKVLLE FEDEF+RMWL FAP++S+

Sbjct 241 VISSNKVLLEHFEDEFQRMWLAFAPRLSS 269

**Tudor-SN**

**>lcl|ORF23**

MSGPSATAPATATNKDGDSAAVVPKRRGVVKQVLSGDTVVIRAQKGAPPP

EKQITFSYVLAPKLARRPGAGGDETKDEPWAWDSREFLRKKLIGEEVLFS

FEKPTNSNREYGFVWLGKDAESGENVVETMVREGFVTVRREGRPSSELQR

LIELEDQAKSANRGRWSHVPTVDKVRYIKWTQENPAHLVEYYGGKPVKAI

IEHVRDGSTVRAFLLPEFQYITLMISGIRCPGVKLDADGKPDLSVKIPFA

DEARFFVESRLLQREVEIRLESVNNSNFIGTIIFPKGNIAESLLREGLAK

CVDWSMAVMKSGADKLRAAERTAKDKRLRLWQDYQSKAPTVNAKEKDFTG

TVVEVFNGDAISVRLSNGLVKKIFFSSIRPPRDTRSGVGADGEVVLPPRG

KNYRPLYEIPFMFEAREFLRKKLINKKVQCNLDYISPARDNFPEKYCYTV

LVGGQNVAEAMVAKGLANCVRHRQDDDQRSSVYDQLLAAESQAIKGQKGM

FGKKDNVPLRINDLTVDHSRIKVQYLPSWQRALRTEGIVEFVASGSRLRL

YVPKDSCLVTFLLAGISCPRSSRPALNGSPAQEGEPYGEEALAFTRDRVL

QRDVSVHIDTTDKAGSSVIGWLWTDNNVNLSVALVEEGLAEVHFSAEKSE

YYRQLKNAEDRAKAAKKNIWANYVEQVVEEKPVVVEEEKDEKAPVERKVN

YEDVIVTEITADLTFFAQAVENGAKLEAMMAKLHADFQVNPPIVGAYTTK

RGDVCAAQFSADNQWYRAKVERVQGNNATVLYIDYGNKETVPTNRLAALP

SAFTSDRPYATEYALALVQLPSDNEDKEEALRLFAEDVLNRTVKLNVELK

PATGPALATVHDPATNADIGKQLVADGYVLAEKRRERKLKDLVEQYRAAQ

QAALTAHLGIWKYGDITQDDAPEFSR

**Graphical representation – ORF**


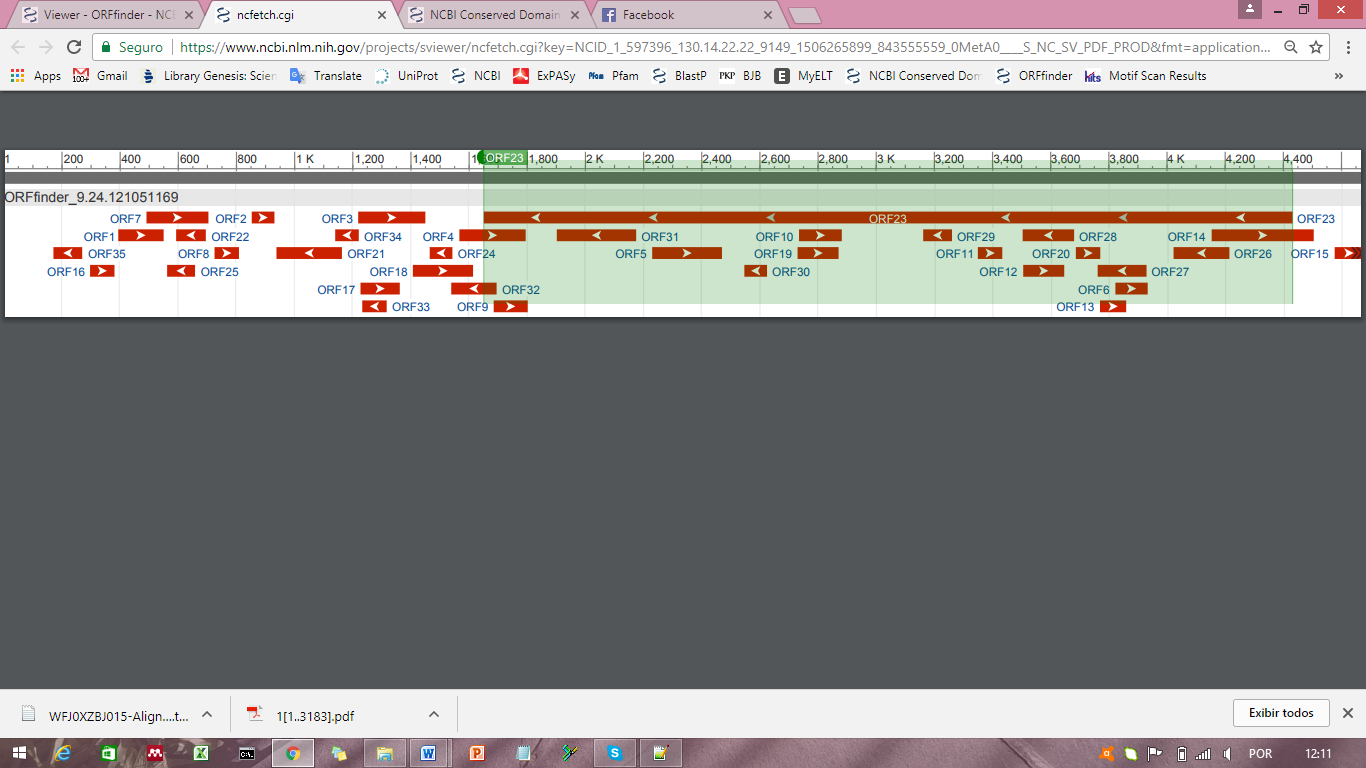


**BlastP (Non-redundant NCBI sequences)**

PREDICTED: staphylococcal nuclease domain-containing protein 1 isoform X1 [Rhagoletis zephyria]

Sequence ID: [XP_017484049.1](https://www.ncbi.nlm.nih.gov/protein/1048062347?report=genbank&log$=protalign&blast_rank=1&RID=WFJX50SB014) Length: 926 Number of Matches: 1

Range 1: 1 to 926

Score: 1782 bits(4616)

E-value: 0.0

Identities: 887/926(96%)

Query 1 MSGPSATAPATATNKDGDSAAVVPKRRGVVKQVLSGDTVVIRAQKGAPPPEKQITFSYVL 60

MSGP+A+APATATNKDGDSAA PKRRGVVKQVLSGDTVVIRAQKGAPPPEKQITFSYVL

Sbjct 1 MSGPAASAPATATNKDGDSAAAAPKRRGVVKQVLSGDTVVIRAQKGAPPPEKQITFSYVL 60

Query 61 APKLARRPGAGGDETKDEPWAWDSREFLRKKLIGEEVLFSFEKPTNSNREYGFVWLGKDA 120

APKLARRPGAGGDETKDEPWAW+SREFLR+KLIGE+VLFSFEKP NSNREYGFVWLGKDA

Sbjct 61 APKLARRPGAGGDETKDEPWAWESREFLRRKLIGEDVLFSFEKPANSNREYGFVWLGKDA 120

Query 121 ESGENVVETMVREGFVTVRREGRPSSELQRLIELEDQAKSANRGRWSHVPTVDKVRYIKW 180

E+GEN+VETMVREGFVTVRREGRPSSELQRLIELEDQAKSANRG+WSHVP V+KVR+IKW

Sbjct 121 ETGENMVETMVREGFVTVRREGRPSSELQRLIELEDQAKSANRGKWSHVPPVEKVRHIKW 180

Query 181 TQENPAHLVEYYGGKPVKAIIEHVRDGSTVRAFLLPEFQYITLMISGIRCPGVKLDADGK 240

TQENPAHLVEYYGGKPVKAIIEHVRDGSTVRAFLLPEFQYITLMISGIRCPGVKLDADGK

Sbjct 181 TQENPAHLVEYYGGKPVKAIIEHVRDGSTVRAFLLPEFQYITLMISGIRCPGVKLDADGK 240

Query 241 PDLSVKIPFADEARFFVESRLLQREVEIRLESVNNSNFIGTIIFPKGNIAESLLREGLAK 300

PDLSVKIPFAD+ARFFVESRLLQR+VEIRLESVNNSNFIGTIIFPKGNIAESLLREGLAK

Sbjct 241 PDLSVKIPFADQARFFVESRLLQRDVEIRLESVNNSNFIGTIIFPKGNIAESLLREGLAK 300

Query 301 CVDWSMAVMKSGADKLRAAERTAKDKRLRLWQDYQSKAPTVNAKEKDFTGTVVEVFNGDA 360

CVDWSMAVMKSGADKLRAAERTAK+KRL LWQDYQSKAPTVNAKEKDFTGTVVEVFNGDA

Sbjct 301 CVDWSMAVMKSGADKLRAAERTAKEKRLHLWQDYQSKAPTVNAKEKDFTGTVVEVFNGDA 360

Query 361 ISVRLSNGLVKKIFFSSIRPPRDTRSGVGADGEVVLPPRGKNYRPLYEIPFMFEAREFLR 420

ISVRLSNG VKK+FFSSIRPPRD RS VGADGEVVLPPRGKNYRPLYEIPFMFEAREFLR

Sbjct 361 ISVRLSNGQVKKVFFSSIRPPRDNRSVVGADGEVVLPPRGKNYRPLYEIPFMFEAREFLR 420

Query 421 KKLINKKVQCNLDYISPARDNFPEKYCYTVLVGGQNVAEAMVAKGLANCVRHRQDDDQRS 480

KKLINKKVQCNLDYISPARDNFPEKYCYTVLVGGQNVAEAMVAKGL +CVRHRQDDDQRS

Sbjct 421 KKLINKKVQCNLDYISPARDNFPEKYCYTVLVGGQNVAEAMVAKGLGHCVRHRQDDDQRS 480

Query 481 SVYDQLLAAESQAIKGQKGMFGKKDNVPLRINDLTVDHSRIKVQYLPSWQRALRTEGIVE 540

SVYDQLLAAESQAIKGQKG+FGKKDN PLRINDLTVDHSRIKVQYLPSWQRALRTEGIVE

Sbjct 481 SVYDQLLAAESQAIKGQKGIFGKKDNAPLRINDLTVDHSRIKVQYLPSWQRALRTEGIVE 540

Query 541 FVASGSRLRLYVPKDSCLVTFLLAGISCPRSSRPALNGSPAQEGEPYGEEALAFTRDRVL 600

FVASGSRLRLYVPKDSCLVTFLLAGISCPRSSRPALNG PAQ+GEPYGEEALAFTRDRVL

Sbjct 541 FVASGSRLRLYVPKDSCLVTFLLAGISCPRSSRPALNGVPAQDGEPYGEEALAFTRDRVL 600

Query 601 QRDVSVHIDTTDKAGSSVIGWLWTDNNVNLSVALVEEGLAEVHFSAEKSEYYRQLKNAED 660

QRDVSVHIDTTDKAGSSVIGWLWTDNNVNLSVALVEEGLAEVHFSAEKSEYYRQLKNAED

Sbjct 601 QRDVSVHIDTTDKAGSSVIGWLWTDNNVNLSVALVEEGLAEVHFSAEKSEYYRQLKNAED 660

Query 661 RAKAAKKNIWANYVEQVVEEKPVVVEEEKDEKAPVERKVNYEDVIVTEITADLTFFAQAV 720

RAKAAKKNIWANYVEQVVEEKPVVVEEEKDEKAPVERKVNYEDVIVTEITADLTFFAQAV

Sbjct 661 RAKAAKKNIWANYVEQVVEEKPVVVEEEKDEKAPVERKVNYEDVIVTEITADLTFFAQAV 720

Query 721 ENGAKLEAMMAKLHADFQVNPPIVGAYTTKRGDVCAAQFSADNQWYRAKVERVQGNNATV 780

ENGAKLEAMMAKLHADFQ NPPIVGAYT KRGD+CAAQFSADNQWYRAKVERVQGNNATV

Sbjct 721 ENGAKLEAMMAKLHADFQANPPIVGAYTAKRGDICAAQFSADNQWYRAKVERVQGNNATV 780

Query 781 LYIDYGNKETVPTNRLAALPSAFTSDRPYATEYALALVQLPSDNEDKEEALRLFAEDVLN 840

LYIDYGNKETVPT+RLA+LPS FTSDRPYATEYALALVQLP+DNEDKEEALRLFAEDVLN

Sbjct 781 LYIDYGNKETVPTHRLASLPSGFTSDRPYATEYALALVQLPTDNEDKEEALRLFAEDVLN 840

Query 841 RTVKLNVELKPATGPALATVHDPATNADIGKQLVADGYVLAEKRRERKLKDLVEQYRAAQ 900

RTVKLNVELKPATGPALATVHD AT ADIGKQLVADGYVLAEKRRERKLKDLVEQYRAAQ

Sbjct 841 RTVKLNVELKPATGPALATVHDAATIADIGKQLVADGYVLAEKRRERKLKDLVEQYRAAQ 900

Query 901 QAALTAHLGIWKYGDITQDDAPEFSR 926

Q AL AHLGIWKYGDITQDDAPEFSR

Sbjct 901 QTALAAHLGIWKYGDITQDDAPEFSR 926

**Vasa intronic**

**>lcl|ORF5**

MDGSGNNRYELLFMDDDISDPLDNVASKSKKQVKTAGGSGGSAPSAKTSN

KQNVDGATRAQSNKKPNQAEKENKPSALNKNELNKKSVAGTAVDRTNKQG

GSAANANRKRTGPASDGGQGGQQQQGTRNLNFRQQNGETREQRNNRRNAR

ENGFAGNFGAQIEGGSSQQQPRQFRDRENRGPPRNRNFEGGNRGGKREFD

RQSGSDKTGVKAIDKRDGAGAHNWGSVKEAIDDVNKSDVEAGNVTDKAEE

SGNEQVDQTPVEEETKELTLDEWKAQKQQRVKPTFNIRKAGEGEDTTQWK

KMIVLNNNKKKDNESEEELEYDPAMYPQRVGRQQRVLDIQFNFNDGRRGG

GFGGRGGRARGPRPGGAVPNTNTNYNNSVTERPVVGGRGVNTGGRGGKRN

FGYKQNVAPKVNDERQFPTLA

**Graphical representation - ORF**


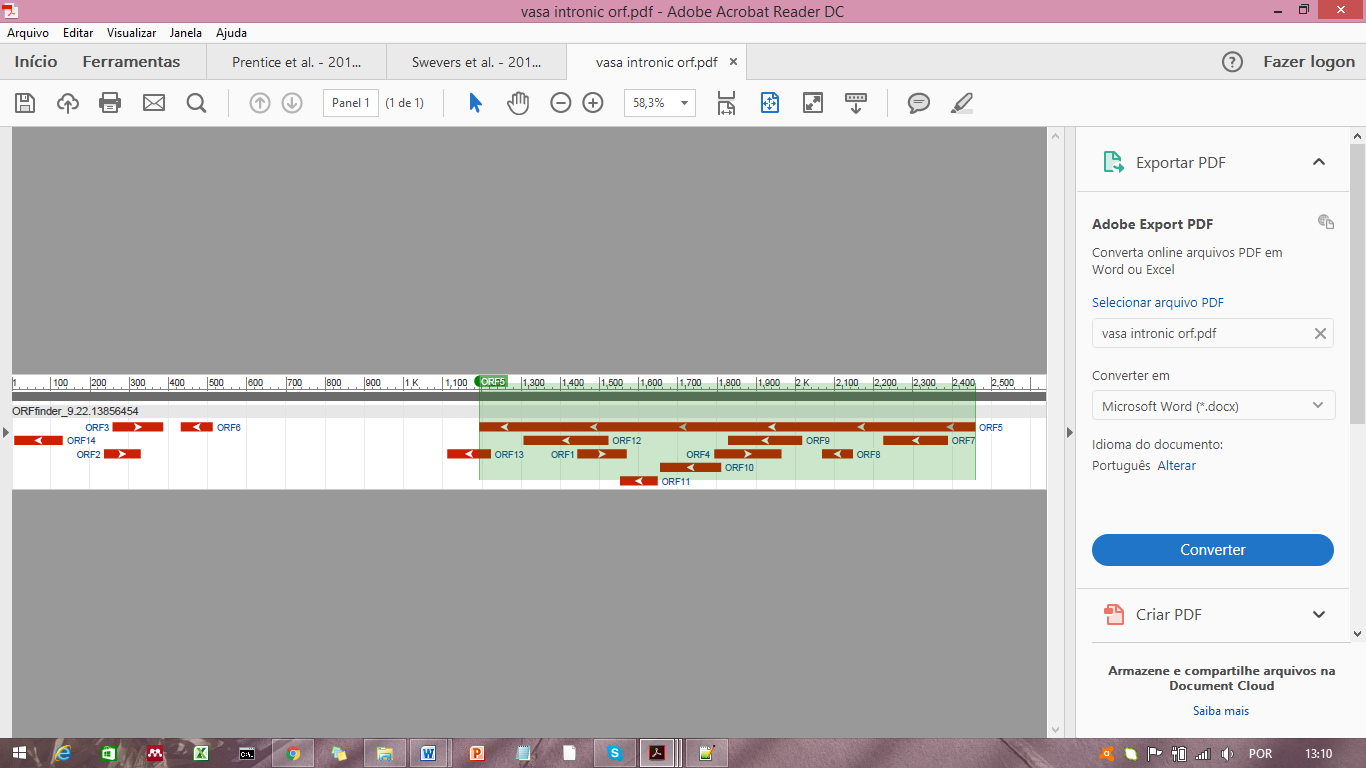


**BlastP (Non-redundant NCBI sequences)**

PREDICTED: plasminogen activator inhibitor 1 RNA-binding protein [Rhagoletis zephyria]

Sequence ID: [XP_017466331.1](https://www.ncbi.nlm.nih.gov/protein/1048010958?report=genbank&log$=protalign&blast_rank=1&RID=WADFCEUM01R) Length: 419 Number of Matches: 1

Range 1: 1 to 419

Score: 650 bits(1676)

E-value: 0.0

Identities: 383/422(91%)

Query 1 MDGSGNNRYELLFMDDDISDPLDNVASKSKKQVKTAGGSGGSAPSAKTSNKQNVDGATRA 60

MDGSGNNRYELLFMDDDISDPLDN+A KSKKQVKTAGGSGGSAP AK+SNKQNVD R

Sbjct 1 MDGSGNNRYELLFMDDDISDPLDNIAIKSKKQVKTAGGSGGSAPLAKSSNKQNVDSVPRT 60

Query 61 QSNKKPNQAEKENKPSALNKNELNKKSVAGTAVDRTNKQGGSAANANRKRTGPASDGGQG 120

QS+KKPNQAEKENKPSALNKNE+NKKS AGTA DRTNKQGG ANANRKRTGPASDGGQG

Sbjct 61 QSSKKPNQAEKENKPSALNKNEVNKKSTAGTAADRTNKQGGPVANANRKRTGPASDGGQG 120

Query 121 GQQQQGTRNLNFRQQNGETREQRNNRRNARENGFAGNFGAQIEGGSSQQQPRQFRDRENR 180

GQQQQGTRNLNFRQQNGETREQRNNRRN RENGF GNFG QIEGGSSQQQPRQFR+RENR

Sbjct 121 GQQQQGTRNLNFRQQNGETREQRNNRRNVRENGFTGNFGMQIEGGSSQQQPRQFRERENR 180

Query 181 GPPRNRNFEGGNRGGKREFDRQSGSDKTGVKAIDKRDGAGAHNWGSVKEAIDDVNKSDVE 240

GPPRNRNFEGGNRGGKREFDRQSGSDKTGVKAIDKRDGAGAHNWGSVKEAIDDVNKSDVE

Sbjct 181 GPPRNRNFEGGNRGGKREFDRQSGSDKTGVKAIDKRDGAGAHNWGSVKEAIDDVNKSDVE 240

Query 241 AGNVTDKAEESGNEQVDQTPVEEETKELTLDEWKAQKQQRVKPTFNIRKAGEGEDTTQWK 300

AGNVTDKAEESGNEQ DQ PVEEETKELTLDEWKAQKQQRVKPTFNIRKAGEGEDTTQWK

Sbjct 241 AGNVTDKAEESGNEQADQAPVEEETKELTLDEWKAQKQQRVKPTFNIRKAGEGEDTTQWK 300

Query 301 KMIVLNNNKKKDNESEEELEYDPAMYPQRVGRQQRVLDIQFNFNDGRRGGGFGGRGGRAR 360

KM+VLNNNKKKDNESEEELEYDPAMYPQRVGRQQRVLDIQFNFNDGRRG G G GR R

Sbjct 301 KMVVLNNNKKKDNESEEELEYDPAMYPQRVGRQQRVLDIQFNFNDGRRGPG-FGGRGRGR 359

Query 361 GPRPGGAVPNTNTNYNNSVTERPVVGGRGVNTGGRGGKRNFGYK-QNVAPKVNDERQFPT 419

GPRPG VPNTNTN+NN+ TERPV GGRGV T GRGGKRNFG++ QN+APKVNDERQFPT

Sbjct 360 GPRPGAGVPNTNTNFNNA-TERPVAGGRGVIT-GRGGKRNFGFRQQNIAPKVNDERQFPT 417

Query 420 LA 421

LA

Sbjct 418 LA 419

**FXMR1**

**>lcl|ORF14**

MDDLMVEVRLDNGAYYKGMVTAVCDDGVFVEVDGCPEVQKYPFSNVRFPP

EENENPPTFEEGMEVEVFTRSTERESCGWWIASIKMIKAEICAVAYIGFE

TPYTEICELKRLRLKNQNPPLTSKSFYQFSIPVPEELRQEAQKDGIHKEF

QRTIGAGVCVYNRDLDSLIVISKWEYTQKRASMLKDMHFRNLAQKVMLLK

RTEEAARQLESTKLLNRGYTDEFQVREDLMGLAIGSHGANIQAARLLEGV

TNIELEEKSCTFKITGESEDSVQRARAMLEYAEEFFQVPRELVGKVIGKN

GRIIQEIVDKSGVFRIKIAGDDEQDQNIPREHGHVPFVFIGTVESISNAK

VLLEYHLGHLKEVELLRQEKLEIDQQLRAIQESTMGSMQNFPVTRRSERG

YSSDIESVRSGRGGPRGRGRGRGGGNGGNQRYHQGRRDDDDYNSRGDHRY

NDRSGGGGGGGYRGGNDRRGGGGKQNGRREQNGRDLGRDHYHNHNDDMRE

DTRELSSVERAESNSSYEGSSRRRRKQKNSNAAHNTNGGVTNNKSQQIKQ

PQNVENNKNNSGGGGGGAGSSSERSKQQSVNSKDGSNVAIKGNNEKPQQQ

QQQQLQQQ

**Graphical representation – ORF**


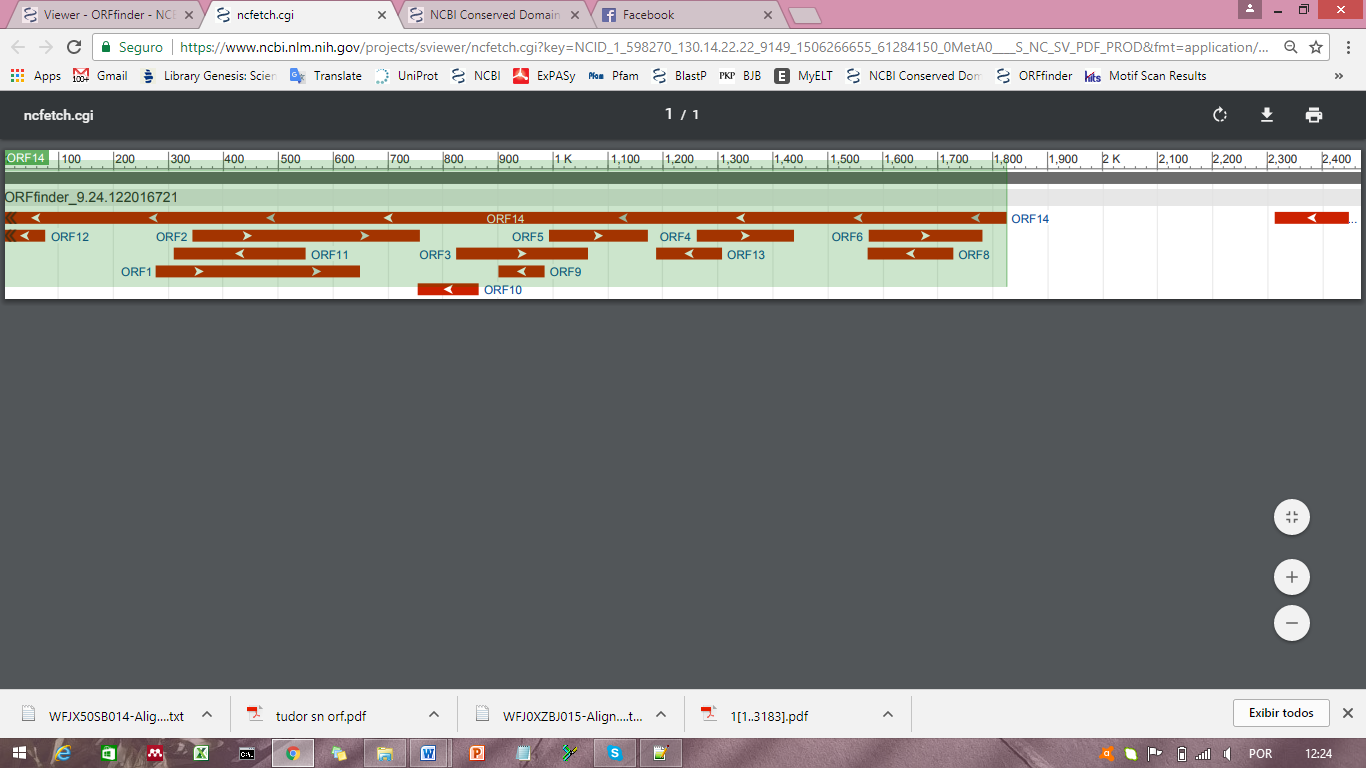


**BlastP (Non-redundant NCBI sequences)**

PREDICTED: synaptic functional regulator FMR1 isoform X1 [Rhagoletis zephyria]

Sequence ID: [XP_017481170.1](https://www.ncbi.nlm.nih.gov/protein/1048054192?report=genbank&log$=protalign&blast_rank=1&RID=WFKMFENC014) Length: 681 Number of Matches: 1

Range 1: 1 to 609

Score: 1228 bits(3177)

E-value: 0.0

Identities: 595/609(98%)

Query 1 MDDLMVEVRLDNGAYYKGMVTAVCDDGVFVEVDGCPEVQKYPFSNVRFPPEENENPPTFE 60

MDDLMVEVRLDNGAYYKGMVTAVCDDGVFVEVDGCPEVQKYPFSNVRFPPEENENPPTFE

Sbjct 1 MDDLMVEVRLDNGAYYKGMVTAVCDDGVFVEVDGCPEVQKYPFSNVRFPPEENENPPTFE 60

Query 61 EGMEVEVFTRSTERESCGWWIASIKMIKAEICAVAYIGFETPYTEICELKRLRLKNQNPP 120

EGMEVEVFTRSTERESCGWWIASIKMIKA+ICAVAYIGFETPYTEICELKRLR+KNQNPP

Sbjct 61 EGMEVEVFTRSTERESCGWWIASIKMIKADICAVAYIGFETPYTEICELKRLRVKNQNPP 120

Query 121 LTSKSFYQFSIPVPEELRQEAQKDGIHKEFQRTIGAGVCVYNRDLDSLIVISKWEYTQKR 180

LTSKSFYQFSIPVPEELRQEAQKDGIHKEFQRTIGAGVCVYNRDLDSLIVISKWEYTQKR

Sbjct 121 LTSKSFYQFSIPVPEELRQEAQKDGIHKEFQRTIGAGVCVYNRDLDSLIVISKWEYTQKR 180

Query 181 ASMLKDMHFRNLAQKVMLLKRTEEAARQLESTKLLNRGYTDEFQVREDLMGLAIGSHGAN 240

ASMLKDMHFRNLAQKVMLLKRTEEAARQLESTKLLNRGYTDEFQVREDLMGLAIGSHGAN

Sbjct 181 ASMLKDMHFRNLAQKVMLLKRTEEAARQLESTKLLNRGYTDEFQVREDLMGLAIGSHGAN 240

Query 241 IQAARLLEGVTNIELEEKSCTFKITGESEDSVQRARAMLEYAEEFFQVPRELVGKVIGKN 300

IQAARLLEGVTNIELEEKSCTFKITGESED+VQRAR+MLEYAEEFFQVPRELVGKVIGKN

Sbjct 241 IQAARLLEGVTNIELEEKSCTFKITGESEDAVQRARSMLEYAEEFFQVPRELVGKVIGKN 300

Query 301 GRIIQEIVDKSGVFRIKIAGDDEQDQNIPREHGHVPFVFIGTVESISNAKVLLEYHLGHL 360

GRIIQEIVDKSGVFRIKIAGDDEQDQNIPREHGHVPFVFIGTVESISNAKVLLEYHLGHL

Sbjct 301 GRIIQEIVDKSGVFRIKIAGDDEQDQNIPREHGHVPFVFIGTVESISNAKVLLEYHLGHL 360

Query 361 KEVELLRQEKLEIDQQLRAIQESTMGSMQNFPVTRRSERGYSSDIESVRSGRGGPRGRGR 420

KEVELLRQEKLEIDQQLRAIQESTMGSMQNFPVTRRSERGYSSDIESVRSGRGGPRGRGR

Sbjct 361 KEVELLRQEKLEIDQQLRAIQESTMGSMQNFPVTRRSERGYSSDIESVRSGRGGPRGRGR 420

Query 421 GRGGGNGGNQRYHQGRRDDDDYNSRGDHRYNDRSGGGGGGGYRGGNDRR-GGGGKQNGRR 479

GRGGGNGGNQRYHQGRRDDDDYNSRGDHRYNDRS GGGGGGYRGGNDRR GGGGKQNGRR

Sbjct 421 GRGGGNGGNQRYHQGRRDDDDYNSRGDHRYNDRSAGGGGGGYRGGNDRRGGGGGKQNGRR 480

Query 480 EQNGRDLGRDHYHNHNDDMREDTRELSSVERAESNSSYEGSSRRRRKQKNSNAAHNTNGG 539

EQNGRDLGRDHYHNHN DMREDTRELSSVERAESNSSYEGSSRRRR+QKN+NAAHNTNGG

Sbjct 481 EQNGRDLGRDHYHNHNADMREDTRELSSVERAESNSSYEGSSRRRRRQKNNNAAHNTNGG 540

Query 540 VTNNKSQQIKQPQNVENNKNNSGGGGGGAGSSSERSKQQSVNSKDGSNVAIKGNNEKPQQ 599

VTNNKSQQ KQPQN +NNK NSGGGGGGAGSSSERSKQQSVNSKDGSNVAIKGN EKPQQ

Sbjct 541 VTNNKSQQNKQPQNADNNKINSGGGGGGAGSSSERSKQQSVNSKDGSNVAIKGNLEKPQQ 600

Query 600 QQQQQLQQQ 608

QQQQQLQQQ

Sbjct 601 QQQQQLQQQ 609

**Rm62 = p68 RNA helicase**

**>lcl|ORF28**

MFKILVQSLAKVSCENRTPSHVAAVACVKNLLIFPSAVINPATSTISRRH

FLFSSTNRGPVNFAFSKYQLFTGKETIAQAEFKKSSNLGGITSSSDSFYR

HFHKTSENLLEAEATPVEKQKFTATKRHQDSFTKTSIYIDPDEMAPHDRN

FGTRGGRGGDDRQARHGGSAGGMRHGGGGGGVGGGDFHGVRNGRIDKNRG

GGRGGSFGGGSGFGGGAGGYGGGNSRGGTQDITLGSVDFSNLAPFKKNFY

QEHPIVANRSPYEVQRFRDEHEITIRGNAPNPIQDFSEAYFPDYVMKEVR

RQGYKAPTAIQAQGWPIAMSGSNFVGIAQTGSGKTLGYILPAIVHINNQK

PLERGDGPIALVLAPTRELAQQIQQVATEFGSSSYVRNTCVFGGAPKGGQ

MRDLQRGCEIVIATPGRLIDFLSMNATNLKRCTYLVLDEADRMLDMGFEP

QIRKILSQIRPDRQTLMWSATWPKEVKQLAEDFLGNYIQINIGSLELSAN

HNIRQVIDVCEEHDKEDKLKSLLSEIYDTSENPGKIIIFVETKRRVDNLV

RFIRSFGVRCGAIHGDKSQSERDYVLREFRSGKSNILVATDVAARGLGKF

HSLRSLLRTLHHYHSHILLYWFDYIFK

**Graphical representation – ORF**


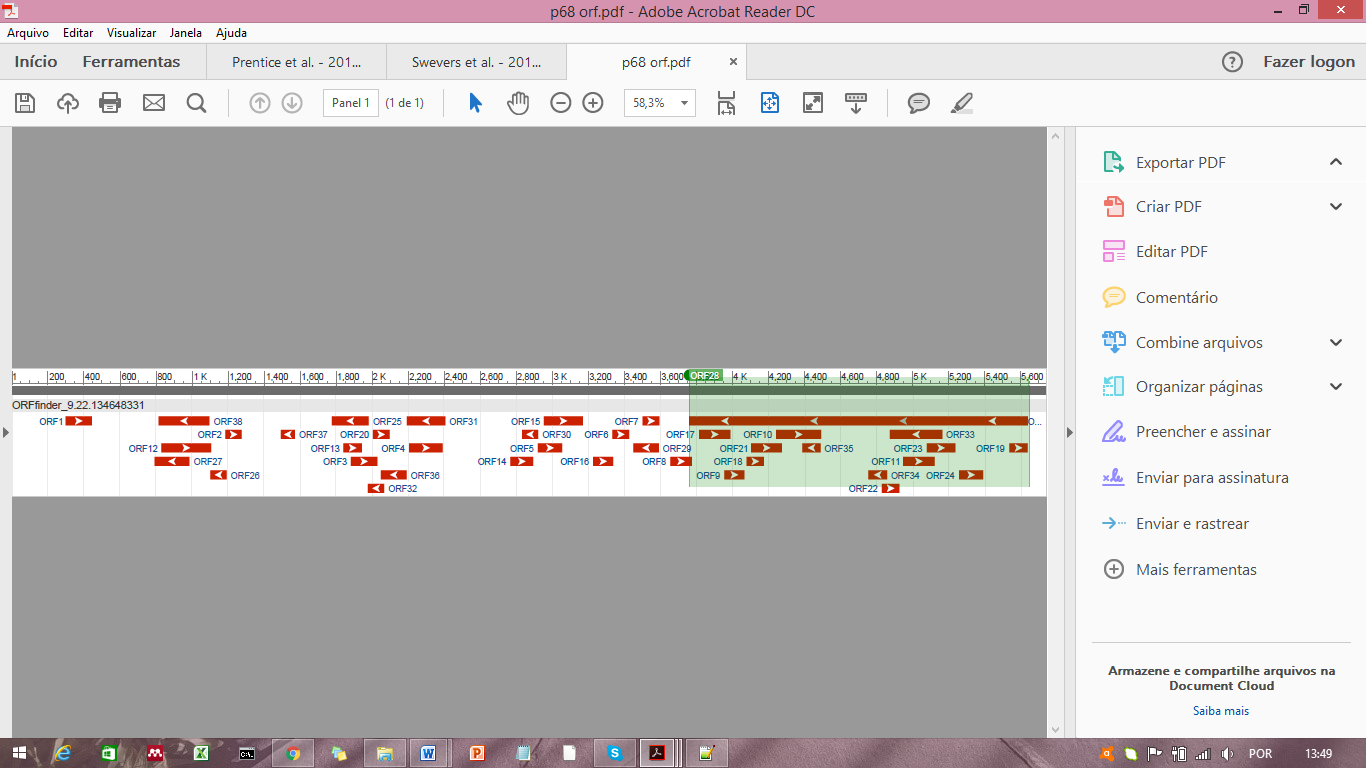


**BlastP (Non-redundant NCBI sequences)**

PREDICTED: ATP-dependent RNA helicase p62 isoform X3 [Zeugodacus cucurbitae]

Sequence ID: [XP_011191631.1](https://www.ncbi.nlm.nih.gov/protein/751440798?report=genbank&log$=protalign&blast_rank=1&RID=WAFR15A2015) Length: 609 Number of Matches: 1

Range 1: 1 to 598

| Score: 956 bits(2470) |
| --- |

E-value: 0.0

Identities: 497/603(82%)

Query 1 MFKILVQSLAKVSCENRTPSHVAAVACVKNLLIFPSAVINP-ATSTISRRHFLFSSTNRG 59

MFKILVQSLAK SCE+R PSH+AAV CVKN LI P+A ATSTI+RRHFLF TNR

Sbjct 1 MFKILVQSLAKNSCESRAPSHIAAVTCVKNSLILPAANNRSIATSTIARRHFLFL-TNRR 59

Query 60 PVNFAFSKYQLFTGKETIAQAEFKKSSNLGGITSS----SDSFYRHFHKTSENLLEAEAT 115

PV+FAF G A AEFKK N SS S SFYR+FH TS NL+EAEA+

Sbjct 60 PVSFAFHHQDQPAGSGKDAVAEFKKIKNTFLTNSSGIINSGSFYRYFHNTSRNLVEAEAS 119

Query 116 PVEKQKFTATKRHQDSFTKTSIYIDPDEMAPHDRNFGTRGGRGGDDRQARHGGSAGGMRH 175

P EKQK + + SFT +S+YIDPDEMAPHDRNFG R RGGDDR ARHGG

Sbjct 120 PAEKQKSSLKTSKKHSFTNSSLYIDPDEMAPHDRNFGARTARGGDDRHARHGGGGMRN-- 177

Query 176 GGGGGGVGGGDFHGVRNGRIDKNRGGGRGGSFGGGSGFGGGAGGYGGGNSRGG-TQDITL 234

GG G G DFHGVRNGR+DK+RGG GG G G GG G G G G TQD+TL

Sbjct 178 --GGAGGSGSDFHGVRNGRVDKSRGGPGGGRGGSGGFGGGSGFGGGYGGGNRGGTQDLTL 235

Query 235 GSVDFSNLAPFKKNFYQEHPIVANRSPYEVQRFRDEHEITIRGNAPNPIQDFSEAYFPDY 294

G+VDFSNLAPFKKNFY+EHP+VANRSPYEVQR+RDEHEITIRGNAPNPIQDF EAYFPDY

Sbjct 236 GTVDFSNLAPFKKNFYEEHPVVANRSPYEVQRYRDEHEITIRGNAPNPIQDFGEAYFPDY 295

Query 295 VMKEVRRQGYKAPTAIQAQGWPIAMSGSNFVGIAQTGSGKTLGYILPAIVHINNQKPLER 354

VMKEVRRQGYKAPTAIQAQGWPIAMSGSNFVGIAQTGSGKTLGYILPAIVHINNQKPLER

Sbjct 296 VMKEVRRQGYKAPTAIQAQGWPIAMSGSNFVGIAQTGSGKTLGYILPAIVHINNQKPLER 355

Query 355 GDGPIALVLAPTRELAQQIQQVATEFGSSSYVRNTCVFGGAPKGGQMRDLQRGCEIVIAT 414

GDGPIALVLAPTRELAQQIQQVATEFGSSSYVRNTCVFGGAPKGGQMRDLQRGCEIVIAT

Sbjct 356 GDGPIALVLAPTRELAQQIQQVATEFGSSSYVRNTCVFGGAPKGGQMRDLQRGCEIVIAT 415

Query 415 PGRLIDFLSMNATNLKRCTYLVLDEADRMLDMGFEPQIRKILSQIRPDRQTLMWSATWPK 474

PGRLIDFL+MNATNLKRCTYLVLDEADRMLDMGFEPQIRKILSQIRPDRQTLMWSATWPK

Sbjct 416 PGRLIDFLAMNATNLKRCTYLVLDEADRMLDMGFEPQIRKILSQIRPDRQTLMWSATWPK 475

Query 475 EVKQLAEDFLGNYIQINIGSLELSANHNIRQVIDVCEEHDKEDKLKSLLSEIYDTSENPG 534

EVKQLAEDFLGNYIQINIGSLELSANHNIRQV+DVCEEHDKEDKLK+LLSEIYDTSENPG

Sbjct 476 EVKQLAEDFLGNYIQINIGSLELSANHNIRQVVDVCEEHDKEDKLKALLSEIYDTSENPG 535

Query 535 KIIIFVETKRRVDNLVRFIRSFGVRCGAIHGDKSQSERDYVLREFRSGKSNILVATDVAA 594

KIIIFVETKRRVDNLVRFIRSFGVRCGAIHGDKSQSERDYVLREFRSGKSNILVATDVAA

Sbjct 536 KIIIFVETKRRVDNLVRFIRSFGVRCGAIHGDKSQSERDYVLREFRSGKSNILVATDVAA 595

Query 595 RGL 597

RGL

Sbjct 596 RGL 598

**Translin**

**>lcl|ORF10**

MANFVDLEIFAKYQKYMENEQEIRENIKIKVREIEQLAKESTIQLQVIHS

DLSNIDNACKEARKQISACSNIYKQLAELVPMGQYYRYSDHWTFITQRLV

FLIAMVVYLEAGFLVNRETVAEMLGLKTNQAHGFHLDIEDYLMGILQMAS

ELSRFATNSVTMGDYDRPLNISHFMANLNSGFRLLNLKNDGLRKRFDALK

YDVKKIEEVVYDITIRGLRNNATQDGGEESSSK

**Graphical representation – ORF**


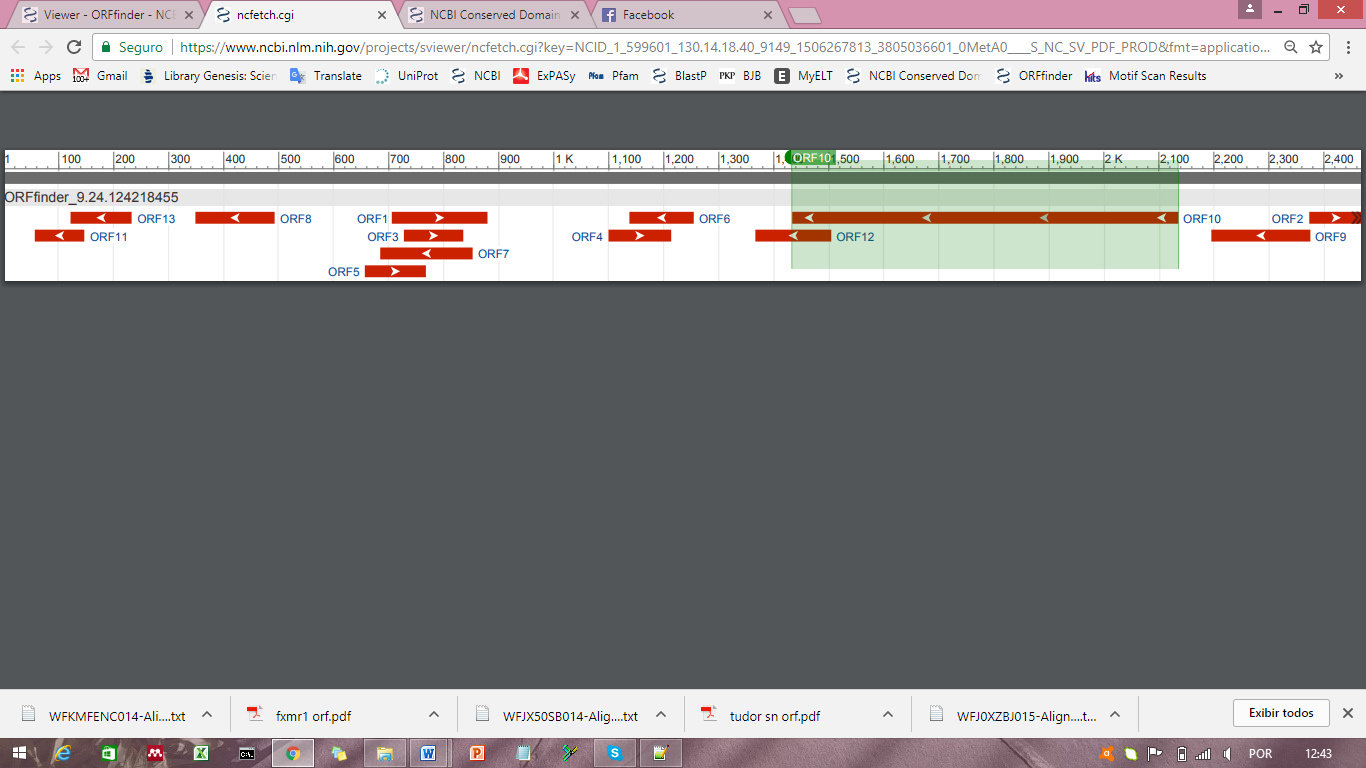


**BlastP (Non-redundant NCBI sequences)**

PREDICTED: translin [Rhagoletis zephyria]

Sequence ID: [XP_017466183.1](https://www.ncbi.nlm.nih.gov/protein/1048010694?report=genbank&log$=protalign&blast_rank=1&RID=WFMUKSWY01R) Length: 245 Number of Matches: 1

Related Information

Range 1: 1 to 228

| Score: 419 bits(1077) |
| --- |

E-value: 3e-147

Identities: 199/228(87%)

Query 1 MANFVDLEIFAKYQKYMENEQEIRENIKIKVREIEQLAKESTIQLQVIHSDLSNIDNACK 60

MA+FV+LEIF+ YQKY++NEQEIRENI+ KVREIEQLAKE+TIQLQVIHSDLS IDNACK

Sbjct 1 MASFVNLEIFSNYQKYIDNEQEIRENIRTKVREIEQLAKEATIQLQVIHSDLSKIDNACK 60

Query 61 EARKQISACSNIYKQLAELVPMGQYYRYSDHWTFITQRLVFLIAMVVYLEAGFLVNRETV 120

EARKQ+SAC+ IYKQL+EL+P+GQYYRYSDHWT+ITQRLVFLIAMVVYLEAGFLV RE

Sbjct 61 EARKQVSACAGIYKQLSELIPVGQYYRYSDHWTYITQRLVFLIAMVVYLEAGFLVKREIA 120

Query 121 AEMLGLKTNQAHGFHLDIEDYLMGILQMASELSRFATNSVTMGDYDRPLNISHFMANLNS 180

AEMLGLK N A GFHLDIEDYLMGILQMASELSRFATNSVTMGDYDRPLNISHFMANLNS

Sbjct 121 AEMLGLKINHADGFHLDIEDYLMGILQMASELSRFATNSVTMGDYDRPLNISHFMANLNS 180

Query 181 GFRLLNLKNDGLRKRFDALKYDVKKIEEVVYDITIRGLRNNATQDGGE 228

GFRLLNLKNDGLRKRFDALKYDVKKIEEVVYDI+IRGLRNN + D E

Sbjct 181 GFRLLNLKNDGLRKRFDALKYDVKKIEEVVYDISIRGLRNNPSLDCKE 228

**Translin associate fator X**

**>lcl|ORF6**

MSQNFRFRNNNRRNQPKQKNVQSLSATDEQNPVLKAFQEYANELDDKHDR

HERLVKLSRDITIEAKRIIFLLHTIDARKNNRFRVLEEAEERLNKIIDTN

FKAIALELNGYDPYQYRWAYSPGLQEFIEAYSFMEYFKCGGDKPLDTMSD

WIILQEKMKYKQEHEKVSQTDDDSEESVVAPDQKQQDTDKAVEKIEPQNV

EFNIDPTEYILGIADLTGELMRRCINSLGSGETDVCLESCKVLKQLFTGY

ISLNVQRCRELSRKIYTMRQSVLKAENVCYNVKVRGGEAAKWGAVFETKA

AEDLDEGFF

**Graphical representation – ORF**


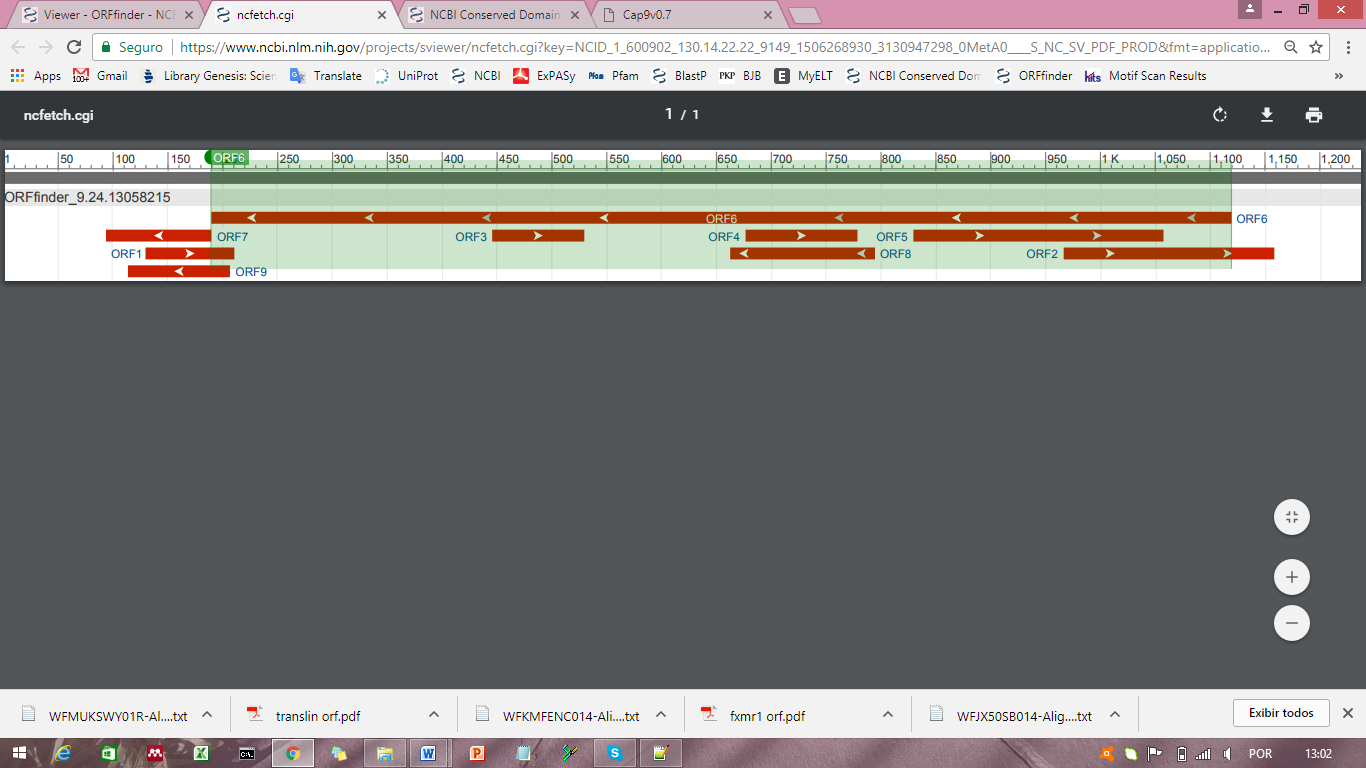


**BlastP (Non-redundant NCBI sequences)**

PREDICTED: translin-associated protein X [Bactrocera latifrons]

Sequence ID: [XP_018798707.1](https://www.ncbi.nlm.nih.gov/protein/1098677621?report=genbank&log$=protalign&blast_rank=1&RID=WFNVTUZC015) Length: 305 Number of Matches: 1

Range 1: 1 to 305

Score: 473 bits(1216)

E-value: 4e-166

Identites: 228/309(74%)

Query 1 MSQNFRFRNNNRRNQPKQKNVQSLSATDEQNPVLKAFQEYANELDDKHDRHERLVKLSRD 60

MSQN RFR+NNRRNQ KQK VQ ++ ++E NPV+K FQEYA ELDDKHDR+ER+VKLSRD

Sbjct 1 MSQNSRFRHNNRRNQQKQKTVQDINFSEELNPVVKVFQEYALELDDKHDRYERIVKLSRD 60

Query 61 ITIEAKRIIFLLHTIDARKNNRFRVLEEAEERLNKIIDTNFKAIALELNGYDPYQYRWAY 120

ITIE+KRIIFLLHTID RKNNR +VLEEAE+RL K+I+ NFK+IALEL G DPYQYR AY

Sbjct 61 ITIESKRIIFLLHTIDGRKNNRSKVLEEAEKRLTKVIEVNFKSIALELTGQDPYQYRCAY 120

Query 121 SPGLQEFIEAYSFMEYFKCGGDKPLDTMSDWIILQEKMKYKQEHEKVSQTDDDSEESVVA 180

SPGLQEFIEAYSFMEY+K G D+ D MSDW+ LQ+KM+Y+++ +K+++ DDS+ES+ +

Sbjct 121 SPGLQEFIEAYSFMEYYKSGADEKSDKMSDWMFLQQKMQYERD-DKLNRVPDDSQESIES 179

Query 181 PDQKQQDTDKAVEKIEPQNVEFNIDPTEYILGIADLTGELMRRCINSLGSGETDVCLESC 240

P+Q ++D K Q EF +DPTEYILGIADLTGELMRRCINSLG GETDVCLE+C

Sbjct 180 PNQLDIESDV---KTVTQKFEFFVDPTEYILGIADLTGELMRRCINSLGGGETDVCLETC 236

Query 241 KVLKQLFTGYISLNVQRCRELSRKIYTMRQSVLKAENVCYNVKVRGGEAAKWGAVFETKA 300

KVL+QL++GYISLNVQRCRELSRKIYTMRQSVLKAENVCYNVKVRGGEAAKWGAVFE K

Sbjct 237 KVLQQLYSGYISLNVQRCRELSRKIYTMRQSVLKAENVCYNVKVRGGEAAKWGAVFENKI 296

Query 301 AEDLDEGFF 309

+DLDEGF+

Sbjct 297 NDDLDEGFY 305

**Armitage**

**>lcl|ORF32**

MLSFLKNLWDSMDAEKQKRMYAEKLEEEQRHLDAEIEKEENKKIEYGDTS

KMQTILRSLNGSENDEVTTTSKEEGTCFERLGVITTLKSNHGTIDHSIFF

DINVAGGLASDLKVGCPVKYLAFQQVHSDHIKVVQIKEVSELYWDESLPT

PEKIEQQITELRNEKPKFFNTHQRNILGLIMERKPGTIVVDTEHKLMNIN

LDTVEIDFIPQAGDRVFICCNVQSDEHFVNRQGEVLQEVSVHPARLLKQE

KCEVRRLNSDWGVLNDDCYFTFDIVSNTCKLNVGDIVIADLIECERDPYI

WRCIKLSLLERIVPQPTTPAGTKKPINGERRSTPKARNESNHAITVSEDQ

RCVFSSTFKTEKVEIIIKNNIERKMRVLSIEFLGRRRDSQVKLVDPELDE

TNFELPGHGSRTLHFEVESKFYGESREVFLIKFETFRVKRSISIVVCETE

AEAEAARAQTSNSIANTSPGMHVMRQRSRHYANQVWSNKGEVIPGVGLAT

KRRFLSHRIAFYEVPERLRYTYLTAANRQEMVDNLEGMFPCLKEELNIKN

YAIRFQTLVYLEEVEYFVNFRNYDRERAHFTRDGEFLSLTIENLSERRPS

LVIGDTVRAINPWCNEDDKRSYDGVIHKVLFNRILLKFNEGFQSKYNGED

FRLEFYYSRFCFRKQHYAVSRIIKNVGEQFLFPSRVYTRDNPQLQIELDS

DNNLMLQGTRCPWFNPLLNPIQKRAIRNILRGEAQNMPYIIFGPPGTGKT

MTLVETILQLVKLIPSSRLLIGTPSNGSADLITTRLIACKQLQPGDFVRL

VSQNQIEKELIPDHLMPYCATVDMAADGTCDDSMIVTESGMKLRCQMKYL

GRHRLTISTCTTLGNFLQMGFPPGHFTHLLIDESGQCTEPEIIVPMSLLS

QKRSQVILAGDPHQLQAIVINRFASERGLSLSLLERILGRTPYLRDLMRF

PDTSGFDPRLVTKLLYNYRALPSILNVYSELFYDSELRAMINENDSREAD

MLKNLDALLPASEKRPKSHGVFFYGTRSENKQEADSPSWFNPLEARNVFL

MTIKLYRQGIQPENIGIITPYMKQVKHLRTLFIEADIAMPKIGSVEEFQG

QERDIILISTVRSSQKLISRDLRHALGFVQSSKRMNVAISRARYLMFIFG

NPHLLYLDHCWRTCIKYCVDNEAYLGCDLPDDFHSRPEQDEQEDTFEITS

KSALPA

**Graphical representation – ORF**


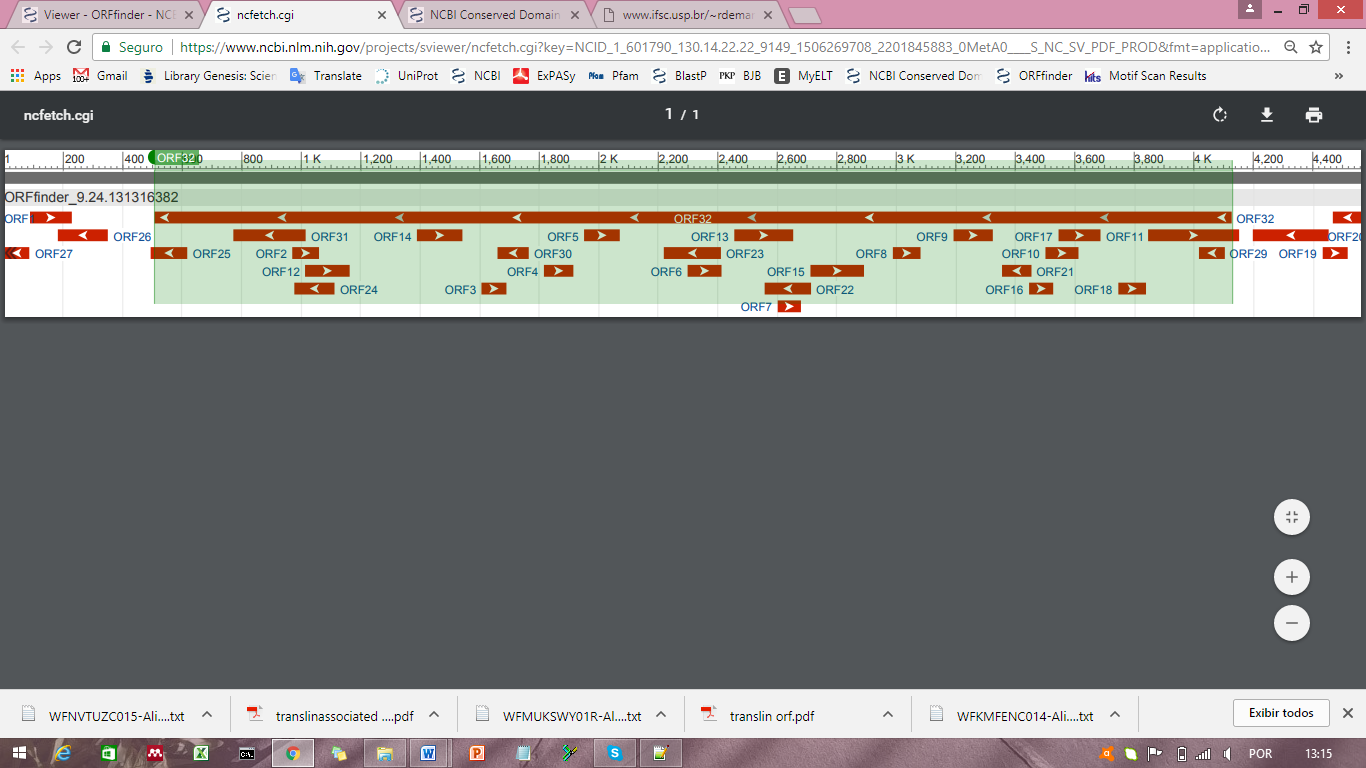


**BlastP (Non-redundant NCBI sequences)**

PREDICTED: probable RNA helicase armi [Rhagoletis zephyria]

Sequence ID: [XP_017472706.1](https://www.ncbi.nlm.nih.gov/protein/1048024184?report=genbank&log$=protalign&blast_rank=1&RID=WFPNF236014) Length: 1218 Number of Matches: 1

Range 1: 1 to 1218

Score: 2196 bits(5689)

E-value: 0.0

Identities: 1060/1218(87%)

Query 1 MLSFLKNLWDSMDAEKQKRMYAEKLEEEQRHLDAEIEKEENKKIEYGDTSKMQTILRSLN 60

MLSFLKNLW+SMDAEKQ++MYAEKLEEEQR LDAEIEKEE +K++ GD S MQ +L S+N

Sbjct 1 MLSFLKNLWESMDAEKQRQMYAEKLEEEQRRLDAEIEKEETEKLKSGDASNMQNVLASIN 60

Query 61 GSENDEVTTTSKEEGTCFERLGVITTLKSNHGTIDHSIFFDINVAGGLASDLKVGCPVKY 120

G++N T +KE+ TCFERLGVITTLKSNHGTIDHSIFFDI+ AGGLASDLKVGC VKY

Sbjct 61 GTKNTNTTLNNKEDDTCFERLGVITTLKSNHGTIDHSIFFDIDSAGGLASDLKVGCHVKY 120

Query 121 LAFQQVHSDHIKVVQIKEVSELYWDESLPTPEKIEQQITELRNEKPKFFNTHQRNILGLI 180

LAFQQ+HSDHIKVVQIKEVSEL+WD+SLPT EKI++QI ELRNEKP FFNTHQRNILGLI

Sbjct 121 LAFQQLHSDHIKVVQIKEVSELFWDDSLPTAEKIDEQIAELRNEKPTFFNTHQRNILGLI 180

Query 181 MERKPGTIVVDTEHKLMNINLDTVEIDFIPQAGDRVFICCNVQSDEHFVNRQGEVLQEVS 240

MERKPG IVVDTEHKLMNINLDTVEIDFIPQAGDRVFICCNVQSDEHFVNRQGEVLQEVS

Sbjct 181 MERKPGAIVVDTEHKLMNINLDTVEIDFIPQAGDRVFICCNVQSDEHFVNRQGEVLQEVS 240

Query 241 VHPARLLKQEKCEVRRLNSDWGVLNDDCYFTFDIVSNTCKLNVGDIVIADLIECERDPYI 300

VHPARLLKQEKCEVRRLN+DWGVLN+DCYFTFD+V NTCKL+VGDIV+ADLIECERDPYI

Sbjct 241 VHPARLLKQEKCEVRRLNADWGVLNEDCYFTFDVVPNTCKLDVGDIVLADLIECERDPYI 300

Query 301 WRCIKLSLLERIVPQPTTPAGTKKPINGERRSTPKARNESNHAITVSEDQRCVFSSTFKT 360

WRCIKL+LLER VPQPT+PA T NG+RR TP ARNES HAITVSEDQRCV +STF++

Sbjct 301 WRCIKLTLLERTVPQPTSPAATGPSTNGDRRPTPNARNESKHAITVSEDQRCVLASTFQS 360

Query 361 EKVEIIIKNNIERKMRVLSIEFLGRRRDSQVKLVDPELDETNFELPGHGSRTLHFEVESK 420

+KVE++IKNN+ERK+RVLSIEFLGRRRDSQV+LVDP+ + T FELPG SRTL FEVESK

Sbjct 361 QKVEMVIKNNVERKLRVLSIEFLGRRRDSQVRLVDPDPNATAFELPGKSSRTLVFEVESK 420

Query 421 FYGESREVFLIKFETFRVKRSISIVVCETEAEAEAARAQTSNSIANTSPG-MHVMRQRSR 479

FYGESREVF+IKFETFRVKRSI +VVCETEAEAEAARAQ ++SI N+S G H +RQRSR

Sbjct 421 FYGESREVFVIKFETFRVKRSILLVVCETEAEAEAARAQPTHSITNSSSGGSHNLRQRSR 480

Query 480 HYANQVWSNKGEVIPGVGLATKRRFLSHRIAFYEVPERLRYTYLTAANRQEMVDNLEGMF 539

HYANQVWSNKGEVIPGVGLATKRRFL+HRIAFYEVPERLR TYLTA++RQEM+DN+E MF

Sbjct 481 HYANQVWSNKGEVIPGVGLATKRRFLAHRIAFYEVPERLRNTYLTASSRQEMMDNVESMF 540

Query 540 PCLKEELNIKNYAIRFQTLVYLEEVEYFVNFRNYDRERAHFTRDGEFLSLTIENLSERRP 599

PCLKEEL+IKNYAIRFQTLVYLEE+EYFVNFRNYDRERAHFTRDGEFLSLTIENLSERRP

Sbjct 541 PCLKEELSIKNYAIRFQTLVYLEEIEYFVNFRNYDRERAHFTRDGEFLSLTIENLSERRP 600

Query 600 SLVIGDTVRAINPWCNEDDKRSYDGVIHKVLFNRILLKFNEGFQSKYNGEDFRLEFYYSR 659

SLVIGDTVRAINPW NEDDKRSYDGVIHKVLFNR+LLKFNEGFQSKYNGEDFRLEFY+SR

Sbjct 601 SLVIGDTVRAINPWSNEDDKRSYDGVIHKVLFNRVLLKFNEGFQSKYNGEDFRLEFYFSR 660

Query 660 FCFRKQHYAVSRIIKNVGEQFLFPSRVYTRDNPQLQIELDSDNNLMLQGTRCPWFNPLLN 719

F FRKQHYAV+RIIK+VGEQFLFPSRVYTRD PQL IELDSD+NLML+GT+CPWFNP LN

Sbjct 661 FAFRKQHYAVNRIIKHVGEQFLFPSRVYTRDEPQLPIELDSDDNLMLKGTQCPWFNPELN 720

Query 720 PIQKRAIRNILRGEAQNMPYIIFGPPGTGKTMTLVETILQLVKLIPSSRLLIGTPSNGSA 779

PIQKRAIRNILRGE+QNMPY+IFGPPGTGKTMTLVET+LQL+K IPSSRLLIGTPSNGSA

Sbjct 721 PIQKRAIRNILRGESQNMPYVIFGPPGTGKTMTLVETMLQLIKHIPSSRLLIGTPSNGSA 780

Query 780 DLITTRLIACKQLQPGDFVRLVSQNQIEKELIPDHLMPYCATVDMAADGTCDDSMIVTES 839

DLI TRLIA KQLQPGDFVRLVSQNQIEKE+IP+HLMPYCATVDMAADGTCDDSMIVTES

Sbjct 781 DLIATRLIASKQLQPGDFVRLVSQNQIEKEMIPEHLMPYCATVDMAADGTCDDSMIVTES 840

Query 840 GMKLRCQMKYLGRHRLTISTCTTLGNFLQMGFPPGHFTHLLIDESGQCTEPEIIVPMSLL 899

GMKLRCQMKYLGRHRLTISTCTTLGNFLQMGFPPGHFTH+LIDESGQCTEPE++VP+ L+

Sbjct 841 GMKLRCQMKYLGRHRLTISTCTTLGNFLQMGFPPGHFTHVLIDESGQCTEPEVMVPVCLV 900

Query 900 SQKRSQVILAGDPHQLQAIVINRFASERGLSLSLLERILGRTPYLRDLMRFPDTSGFDPR 959

+QKR QVILAGDPHQLQAIVINRFASE GLSLSLLERILGRTPYLRD+MRFPDTSGFDPR

Sbjct 901 AQKRGQVILAGDPHQLQAIVINRFASEHGLSLSLLERILGRTPYLRDVMRFPDTSGFDPR 960

Query 960 LVTKLLYNYRALPSILNVYSELFYDSELRAMINENDSREADMLKNLDALLPASEKRPKSH 1019

LVTKLLYNYR+LPSILNVYSELFYDSELRAM+NE DSREA+MLK LD LLP SEKRPKSH

Sbjct 961 LVTKLLYNYRSLPSILNVYSELFYDSELRAMVNEKDSREAEMLKKLDVLLPTSEKRPKSH 1020

Query 1020 GVFFYGTRSENKQEADSPSWFNPLEARNVFLMTIKLYRQGIQPENIGIITPYMKQVKHLR 1079

G+FFYGTRSENKQE+DSPSWFNPLEARNVFLMTIKLYRQGI+PENIGIITPYMKQVKHLR

Sbjct 1021 GIFFYGTRSENKQESDSPSWFNPLEARNVFLMTIKLYRQGIEPENIGIITPYMKQVKHLR 1080

Query 1080 TLFIEADIAMPKIGSVEEFQGQERDIILISTVRSSQKLISRDLRHALGFVQSSKRMNVAI 1139

TLFIEADIAMPKIGSVEEFQGQERDIILISTVRSSQKLIS DLRHALGFVQS+KRMNVAI

Sbjct 1081 TLFIEADIAMPKIGSVEEFQGQERDIILISTVRSSQKLISSDLRHALGFVQSAKRMNVAI 1140

Query 1140 SRARYLMFIFGNPHLLYLDHCWRTCIKYCVDNEAYLGCDLPDDFHSRPEQDEQ-EDTFEI 1198

SRARYLMFIFGNPHLLYLDHCWRTCIKYCVDNEAYLGCDLPDDF +RPE DEQ E+T E

Sbjct 1141 SRARYLMFIFGNPHLLYLDHCWRTCIKYCVDNEAYLGCDLPDDFDTRPELDEQGEETIES 1200

Query 1199 TS----------KSALPA 1206

+ +SALPA

Sbjct 1201 ATIATTTNEPALESALPA 1218

**Homeless (spindle-E)**

**>lcl|ORF4**

MNEIEEFFNFTKDFKREPALRGYISGDLAATAGKFDNGKPIKREYFGDQY

SKPVAEKERRRMLDEDVSMEEIHSSRQGATSSTCMDELEDLSEEEDAKPK

ILRIDDGVYSKYNFNLKRDESLPIHDNKDQILNAIRKNPVVVLEGDTGCG

KTTQVPQYILDEAYENRDYCKIVCTQPRRIAAISIARRVCQERKWEEGSV

VGFQVGLHANISEDTRLIYCTTGVLLQKLIKEKSLKQFTHIILDEVHERD

QEMDFLLIVIRKLLTTNSRDVKIVLMSATINAGEFSDYFTIRRKPAPVLR

VDSRRLFQVREFYLSDLGRINSANIDVDISDPGISKEMFNIALKLIIVID

NIEKQEAAVAIDNGPLQQTSILIFLPGINEIEQMSSKLRLLSESDDNNVK

LYPIRLHSLISPDEQNKVFNNPPGGFRKVILSTNIAESSITVPDVKYVID

FCLTKSLITDTATNFSSLQLHWASRANCRQRAGRAGRVMNGRVYRMVSKN

FYDHYMEEFSTAEMLRCPLENAVLKAKLLDMGPPPDILGLAMTPPNLSDI

HNTILTLKEVGALFTTVNGVYSIQDGDLTFMGRVMAGMPLDIRLTRLILL

GYIFSALDETIIMAAGLSVRSIFKSSVDRRGQGEADAYIQKLVWADGSGS

DLFAILSAYRVWSSMREQQNIHEEGPEYNWAKRFFINLRSMKEMHLLVTE

LRSRLKSYGIREQQAYQRVCWIDREMTIILKIIIAGAFYPNYFTRSNLND

TERERGIYHTLCGNDPCNTVYFTGFNTRHIGQLYAGSIKDLFRAVRIHPK

NIEVRFQPGAERVFVTFKNDRDDDSEGGAYRLVVPGRVCPEVYKAVRMRM

LGMRTTMRVMDPRNEVKYAEERHIGRMVEGIWQPTKKQIKNPELIVLPSV

FQKMIRGYITHIESCRKFYFQPLSEMERLREINALLNNPEDLEGGRFRNP

AAISKGMMVAAPFENKYHRATVLKVLTAARQHCQFKVFFVDYGNTDVIDF

EQLRRLPYRCESLVDIPPRMFECRLVMVEPSSVKSPSGKWPEEAMEFMQQ

TADAGVVEIEVYSVVAGVSNVIIKTATGTLNDILVEKGLAHKSDENYMSK

ADHDFRLRKQSVATRFLDEDHSKQNEEYMRSIQQETDLEVDPPPREYCTK

AINLRGPFSALETKICSAVRIGTWKSVNVERDSVNSVLIDTDPQDVHERL

IVAASITEAQSAETLTARSTTLMPNIHGFGALMTLLFCPTMQIKRNSNKT

KYVTVLAGLGYNEETYKPLYEEHDIVLNLDAEILKDDIELTNQLRYCMDT

MLYTDPGCETLPILPNTRADLSAKIKNLIIRLLNKNRKYIETHVDSLDNV

WQRYDPEEVIETEPIYGKRSIFPLHAALKLYDEKFDRIHALGVHCQELHR

LRQFDGSIQPITCQLCNQSLENIVQLRIHLLSQLHRDREYQIRFKMPR

**Graphical representation – ORF**


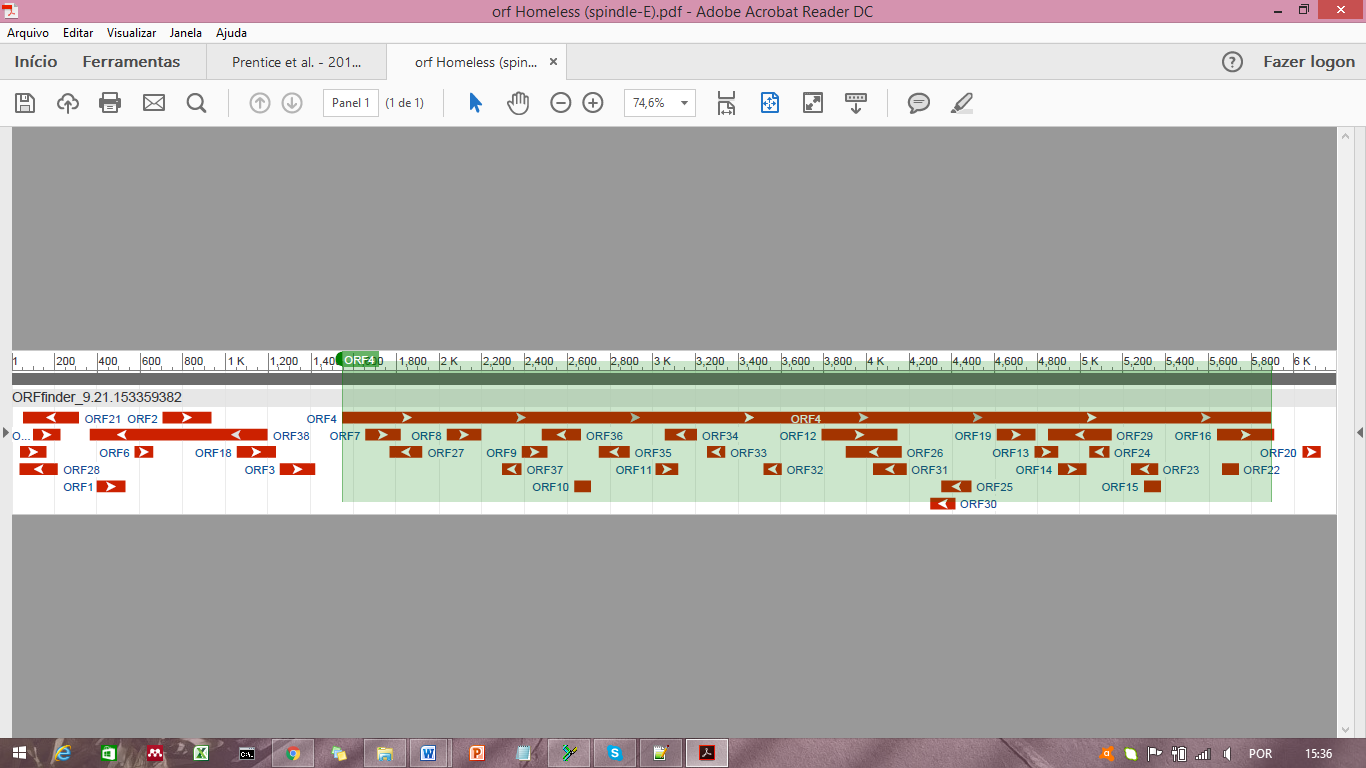


**BlastP (Non-redundant NCBI sequences)**

PREDICTED: probable ATP-dependent RNA helicase spindle-E [Rhagoletis zephyria]

Sequence ID: [XP_017468006.1](https://www.ncbi.nlm.nih.gov/protein/1048014100?report=genbank&log$=protalign&blast_rank=1&RID=W822RCZG014) Length: 1449 Number of Matches: 1

Range 1: 1 to 1449

Score: 2659 bits(6893)

E-value: 0.0

Identities: 1278/1449(88%)

Query 1 MNEIEEFFNFTKDFKREPALRGYISGDLAATAGKFDNGKPIKREYFGDQYSKPVAEKERR 60

M+E+ FF+F+K+FKRE RG +SG L ATAGK D+GKPIKREYFGDQYSK +AEKERR

Sbjct 1 MDEVMAFFDFSKEFKRESVPRGCVSGSLTATAGKIDSGKPIKREYFGDQYSKKIAEKERR 60

Query 61 RMLDE-DVSMEEIHSSRQGATSSTCMDELEDLSEEEDAKPKILRIDDGVYSKYNFNLKRD 119

RML+E D ME+ HSSR GATSSTCMDELEDLSEEE+ +PK+ RI+D VY+KYNF+LKRD

Sbjct 61 RMLEENDDPMEDAHSSRHGATSSTCMDELEDLSEEEEIQPKVHRIEDNVYTKYNFDLKRD 120

Query 120 ESLPIHDNKDQILNAIRKNPVVVLEGDTGCGKTTQVPQYILDEAYENRDYCKIVCTQPRR 179

ESLPIHDNKDQIL +IRK+PVVVLEGDTGCGKTTQVPQYILDEAYENR+YCKIVCTQPRR

Sbjct 121 ESLPIHDNKDQILESIRKHPVVVLEGDTGCGKTTQVPQYILDEAYENREYCKIVCTQPRR 180

Query 180 IAAISIARRVCQERKWEEGSVVGFQVGLHANISEDTRLIYCTTGVLLQKLIKEKSLKQFT 239

IAAISI++RVCQERKWE GSVVGFQVGLHA EDTRL+YCTTGVLLQKLIKEKSLKQFT

Sbjct 181 IAAISISKRVCQERKWEVGSVVGFQVGLHAKTCEDTRLLYCTTGVLLQKLIKEKSLKQFT 240

Query 240 HIILDEVHERDQEMDFLLIVIRKLLTTNSRDVKIVLMSATINAGEFSDYFTIRRKPAPVL 299

HIILDEVHERDQEMDFLLIVIRKLLTTNSRDV+++LMSATINAGEFS+YFTIRR PAPVL

Sbjct 241 HIILDEVHERDQEMDFLLIVIRKLLTTNSRDVRVILMSATINAGEFSEYFTIRRNPAPVL 300

Query 300 RVDSRRLFQVREFYLSDLGRINSANIDVDISDPGISKEMFNIALKLIIVIDNIEKQEAAV 359

RVDSRRLFQVREFYLSDLGRIN+ NIDVDISDPGISKEM+NIALKLIIVIDNIEKQEAAV

Sbjct 301 RVDSRRLFQVREFYLSDLGRINTTNIDVDISDPGISKEMYNIALKLIIVIDNIEKQEAAV 360

Query 360 AIDNGPLQQTSILIFLPGINEIEQMSSKLRLLSESDDNNVKLYPIRLHSLISPDEQNKVF 419

+ + PL QTSILIFLPGINEI+QM SKL LLSESD+NNVKL+PIRLHSLISPDEQNKVF

Sbjct 361 SAEISPLPQTSILIFLPGINEIDQMCSKLELLSESDENNVKLFPIRLHSLISPDEQNKVF 420

Query 420 NNPPGGFRKVILSTNIAESSITVPDVKYVIDFCLTKSLITDTATNFSSLQLHWASRANCR 479

NNPPGGFRKVIL+TNIAESSITVPDVKYVIDFCLTKSLITDTATNFSSLQLHWASRANCR

Sbjct 421 NNPPGGFRKVILATNIAESSITVPDVKYVIDFCLTKSLITDTATNFSSLQLHWASRANCR 480

Query 480 QRAGRAGRVMNGRVYRMVSKNFYDHYMEEFSTAEMLRCPLENAVLKAKLLDMGPPPDILG 539

QRAGRAGRVMNGRVYRMVSKNFY+HYMEEF TAEMLRCPLENAVLKAKLLDMGPPPDILG

Sbjct 481 QRAGRAGRVMNGRVYRMVSKNFYEHYMEEFGTAEMLRCPLENAVLKAKLLDMGPPPDILG 540

Query 540 LAMTPPNLSDIHNTILTLKEVGALFTTVNGVYSIQDGDLTFMGRVMAGMPLDIRLTRLIL 599

LAMTPPNLSDIHNTILTLKEVGALFTTVNGVYSIQDGDL+FMGRVMAGMPLDIRLTRLIL

Sbjct 541 LAMTPPNLSDIHNTILTLKEVGALFTTVNGVYSIQDGDLSFMGRVMAGMPLDIRLTRLIL 600

Query 600 LGYIFSALDETIIMAAGLSVRSIFKSSVDRRGQGEADAYIQKLVWADGSGSDLFAILSAY 659

LG+IFSAL+E+IIMAAGLSVRSI KS D RGQGEADAY QKL+WADGSGSDLFAI SAY

Sbjct 601 LGHIFSALEESIIMAAGLSVRSILKSGHDNRGQGEADAYKQKLIWADGSGSDLFAIYSAY 660

Query 660 RVWSSMREQQNIHEEGPEYNWAKRFFINLRSMKEMHLLVTELRSRLKSYGIREQQAYQRV 719

R+W++ REQ NIH E EY WAKR F+NLRSMKEMHLLVTELR RL++YGIREQ YQRV

Sbjct 661 RLWATQREQHNIHHEENEYEWAKRHFVNLRSMKEMHLLVTELRDRLQAYGIREQTTYQRV 720

Query 720 CWIDREMTIILKIIIAGAFYPNYFTRSNLNDTERERGIYHTLCGNDPCNTVYFTGFNTRH 779

CW++RE TIILKIIIAGAFYPNYFTRSNLNDTERERGIYHTLCGNDPCNTVYFTGFNTRH

Sbjct 721 CWVEREKTIILKIIIAGAFYPNYFTRSNLNDTERERGIYHTLCGNDPCNTVYFTGFNTRH 780

Query 780 IGQLYAGSIKDLFRAVRIHPKNIEVRFQPGAERVFVTFKNDRDDDSEGGAYRLVVPGRVC 839

IGQLYA SIK LFR+V I PKNIEVRFQ GAERVFVTFK D ++D+E AYRLVVPGRV

Sbjct 781 IGQLYASSIKALFRSVWIDPKNIEVRFQTGAERVFVTFKKDLENDAEDSAYRLVVPGRVV 840

Query 840 PEVYKAVRMRMLGMRTTMRVMDPRNEVKYAEERHIGRMVEGIWQPTKKQIKNPELIVLPS 899

PEVY AVRMRMLGMRTT+RVMDPRNEV+YAEER IG MVEG+WQPTKKQIKNPEL+VLPS

Sbjct 841 PEVYMAVRMRMLGMRTTIRVMDPRNEVRYAEERRIGTMVEGVWQPTKKQIKNPELVVLPS 900

Query 900 VFQKMIRGYITHIESCRKFYFQPLSEMERLREINALLNNPEDLEGGRFRNPAAISKGMMV 959

VFQKMIRGYITHI +C KFYFQPLSEMERLREI+ALLNNPEDLE GRF++PAAISKGMMV

Sbjct 901 VFQKMIRGYITHIVNCSKFYFQPLSEMERLREIHALLNNPEDLERGRFKSPAAISKGMMV 960

Query 960 AAPFENKYHRATVLKVLTAARQHCQFKVFFVDYGNTDVIDFEQLRRLPYRCESLVDIPPR 1019

AAPFENKYHRA V+KVLTAARQ+CQFKVFFVDYGNTDVIDFEQLRR YRCESL+DIPPR

Sbjct 961 AAPFENKYHRAKVVKVLTAARQYCQFKVFFVDYGNTDVIDFEQLRRFSYRCESLIDIPPR 1020

Query 1020 MFECRLVMVEPSSVKSPSGKWPEEAMEFMQQTADAGVVEIEVYSVVAGVSNVIIKTATGT 1079

MFE RLVMVEPSSVKSPSGKWP+EAMEFMQQTADAGVVEIE+YSVVAGVSNVIIKTATGT

Sbjct 1021 MFESRLVMVEPSSVKSPSGKWPDEAMEFMQQTADAGVVEIEIYSVVAGVSNVIIKTATGT 1080

Query 1080 LNDILVEKGLAHKSDENYMSKADHDFRLRKQSVATRFLDEDHSKQNEEYMRSIQQETDLE 1139

LNDILVEKG A KSDENYMSKADHDFRLRKQSVATRFLDEDHSKQNEEY+RSIQ E DLE

Sbjct 1081 LNDILVEKGFAQKSDENYMSKADHDFRLRKQSVATRFLDEDHSKQNEEYLRSIQPEADLE 1140

Query 1140 VDPPPREYCTKAINLRGPFSALETKICSAVRIGTWKSVNVERDSVNSVLIDTDPQDVHER 1199

VDPPPREYCTK+INLRGP+SALETKI SAVRIGTWKSVNVERDSVNSVLIDTDPQDVHER

Sbjct 1141 VDPPPREYCTKSINLRGPYSALETKIFSAVRIGTWKSVNVERDSVNSVLIDTDPQDVHER 1200

Query 1200 LIVAASITEAQSAETLTARSTTLMPNIHGFGALMTLLFCPTMQIKRNSNKTKYVTVLAGL 1259

LIVAASITEAQ+AETLTAR+TTLMPNIHGFGALMTLLF PTMQIKRNSNKTKYV +LAGL

Sbjct 1201 LIVAASITEAQNAETLTARATTLMPNIHGFGALMTLLFSPTMQIKRNSNKTKYVAILAGL 1260

Query 1260 GYNEETYKPLYEEHDIVLNLDAEILKDDIELTNQLRYCMDTMLYTDPGCETLPILPNTRA 1319

GYN+ETYKPLYEEHDIVLNLDA+ILKDD+EL NQLRYCMDT+LYTDPG E ILP+ RA

Sbjct 1261 GYNKETYKPLYEEHDIVLNLDADILKDDLELINQLRYCMDTILYTDPGEERPTILPSNRA 1320

Query 1320 DLSAKIKNLIIRLLNKNRKYIETHVDSLDNVWQRYDPEEVIETEPIYGKRSIFPLHAALK 1379

L+AKIKNLI+RLLNKNRKYIETHVDS DNVWQRYDPE+VIE+EPIYG RS+FP+H+ALK

Sbjct 1321 YLAAKIKNLIVRLLNKNRKYIETHVDSYDNVWQRYDPEDVIESEPIYGHRSLFPVHSALK 1380

Query 1380 LYDEKFDRIHALGVHCQELHRLRQFDGSIQPITCQLCNQSLENIVQLRIHLLSQLHRDRE 1439

LYDEKFDRIHALGVHCQELHRLRQFDG+IQP+TCQLCNQSLENIVQLRIHLLSQLHRDRE

Sbjct 1381 LYDEKFDRIHALGVHCQELHRLRQFDGAIQPLTCQLCNQSLENIVQLRIHLLSQLHRDRE 1440

Query 1440 YQIRFKMPR 1448

+QIRFKMPR

Sbjct 1441 HQIRFKMPR 1449

**Maelstrom**

**>lcl|ORF2**

MRKNKKPNGFLTFTIEWKSKYGKRMTLSQATEEAGKIWISMSMEERSPYN

ERAKQERSKLKSAPPKLTCTGKPLDQVEKDLQVADQWNVQMKRNIEMVVR

NSVNNDQLKTQSYFFVMVNYFVKSLRGDIYVPAEISVAEYSLQEGVCRKY

HTLINPGRDLYGLQFDAQDHADRTHKLPLPPNALGEENLGLIYNHVLDFV

RDPETGDYPPIYTHRDSIPIVKSVLDFLKNSSSTNVELKVYSIQYLFFIL

KEATAKEGDVRSPKCHYITDACFDRDFYEYQTDIACNKHEEMDKSKYCTQ

SCVTRWGYIFSDFMCGDVAISVVEGRHKPIRTDVDHSDNNNPAPPSTCFD

TESQISGNSESTYATRPFSENRVCYDDHKTFMSAHSSAIGTSTNNNQTND

FPYLGARKKTSKITSPSPPRSSRWNTEGFDGRTLDLDLCEGGNPWSIRSR

DVPREPDTSHFDINYTRDETTDNDRPGTSSGYGRGRMRMNHSATSNTTVG

SGRGRLCRNPDYRN

**Graphical representation – ORF**


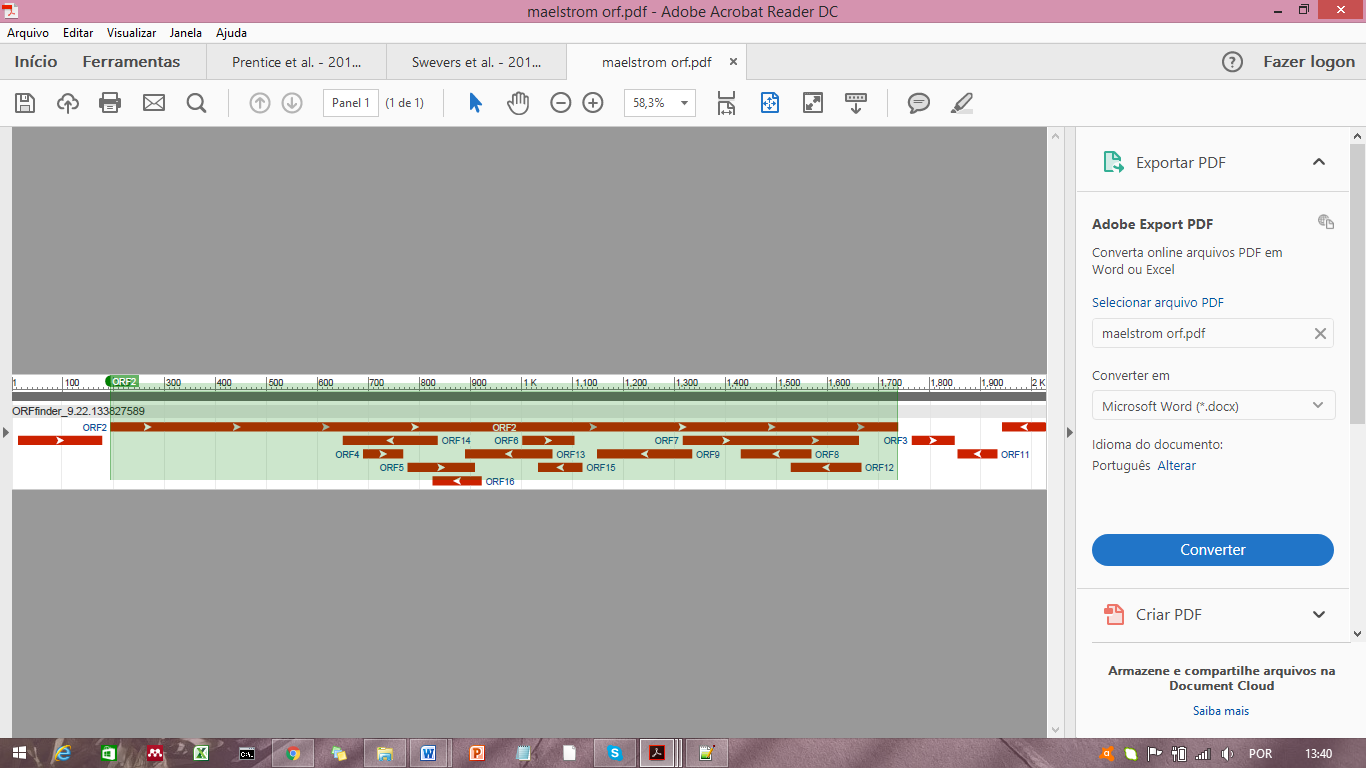


**BlastP (Non-redundant NCBI sequences)**

PREDICTED: protein maelstrom 1-like [Rhagoletis zephyria]

Sequence ID: [XP_017471127.1](https://www.ncbi.nlm.nih.gov/protein/1048021248?report=genbank&log$=protalign&blast_rank=1&RID=WAF53ZK7014) Length: 510 Number of Matches: 1

Range 1: 1 to 505

Score: 676 bits(1745)

E-value: 0.0

Identities: 334/514(65%)

Query 1 MRKNKKPNGFLTFTIEWKSKYGKRMTLSQATEEAGKIWISMSMEERSPYNERAKQERSKL 60

M K K NGF+ FT EW+SKYGK+++L++ATEEAGKIW SM++EER+PYN+RA+QER

Sbjct 1 MPKKPKTNGFMMFTTEWRSKYGKKLSLAEATEEAGKIWGSMTVEERAPYNDRARQERMGQ 60

Query 61 KSA----PPKLTCTGKPLDQVEKDLQVADQWNVQMKRNIEMVVRNSVNNDQLKTQSYFFV 116

KS PPKLTCTGK ++QVEK+ A+Q QMKRNIEM VRN V N QL+TQSYFF+

Sbjct 61 KSGRPAGPPKLTCTGKAIEQVEKERAEAEQREHQMKRNIEMTVRNCVKNCQLETQSYFFI 120

Query 117 MVNYFVKSLRGDIYVPAEISVAEYSLQEGVCRKYHTLINPGRDLYGLQFDAQDHADRTHK 176

MVNYFVKSL+G IYVPAEI VAEYSL++GVCRKYHT INPG ++YGL ++AQ H++ +HK

Sbjct 121 MVNYFVKSLKGGIYVPAEILVAEYSLKDGVCRKYHTFINPGPNIYGLHYEAQHHSETSHK 180

Query 177 LPLPPNALGEENLGLIYNHVLDFVRDPETGDYPPIYTHRDSIPIVKSVLDFLKNSSSTN- 235

LPLPPNA GE NLGLIYN+V+DF+RD TG+YPPIYTHRDSI IV+SVL+FLK+ + N

Sbjct 181 LPLPPNAKGESNLGLIYNNVVDFIRDENTGEYPPIYTHRDSITIVESVLEFLKSDLNANS 240

Query 236 VELKVYSIQYLFFILKEATAKEGDVRSPKCHYITDACFDRDFYEYQTDIACNKHEEMDKS 295

VELK+YSIQYLF+ILKEAT++ G+V PK HYITDA F+RDF+EYQT IAC HE++DKS

Sbjct 241 VELKIYSIQYLFYILKEATSEMGEVEQPKSHYITDAYFERDFFEYQTGIACTYHEDIDKS 300

Query 296 KYCTQSCVTRWGYIFSDFMCGDVAISVVEGRHKPIRTDVDHSDNNNPAPPSTCFDTESQI 355

KYCTQSCVTRWG++FSD+MC D+AI ++ GRH P T++ + NPA PST DTES I

Sbjct 301 KYCTQSCVTRWGFMFSDYMCRDIAIDLIPGRHVPESTNL--AAIINPA-PSTYGDTESHI 357

Query 356 SGNSESTYATRPFSENRVCYDDHKTFMSAHSSAIGTSTNNNQTNDFPYLGA---RKKTSK 412

S NSESTYAT+ SE++V YDDHKTFMSA S+A G S + TN+FP LGA RKK ++

Sbjct 358 SVNSESTYATKAISEHKVYYDDHKTFMSARSNATGRSIDKYDTNEFPSLGAGPSRKKHAR 417

Query 413 ITSPSPPRSSRWNTEGFDGRTLDLDLCEGGNPWSIRSRDVPREPDTSHFDINYTRDETTD 472

SPSP +++R + RT+D++ + NPWS RSR+VPREPDTSHFDI+YTRD+TTD

Sbjct 418 AVSPSPAKATRTD----HIRTIDIESADDLNPWSSRSRNVPREPDTSHFDIDYTRDDTTD 473

Query 473 NDRPGTSSGYGRGRMRMNHSATSNTTVGSGRGRL 506

N+ T +GYGRGRMRMN S SN T GSGRGRL

Sbjct 474 NET--TVTGYGRGRMRMNQSTISNATAGSGRGRL 505

**HEN1**

**>lcl|ORF9**

MFSYKLPDGGLRTCCYINAEGGIKFDPPVYEQRYTTAVRILEDSRWGQKF

KKVVDFGCADMRLLPLLRRVKGIEHILEVDVDENILRSNKLRAEPLVSDY

LMRRENPLRVELLKGSIDASVEQLLNVDAVVALEIIEHLYPKTLENVPKN

IFGFMQPKIAIFSTPNSEFNVLFQPLLANGFRHDDHKFEWTRAEFRDWAR

TICQQYPNYSVAFLGVGDAPPDNKSIGNVSQIAIFARHDLLGRRLVEPLR

SEVPVDCNTDADVQYKEIFAVDFPFNVDERSKEKKILDEARYQIDRCRRI

DRYFNQDLGVYQVPLTVMDTYIETLGASTEELLDVLKDNDIEVKGDYIIL

PEYDEDDLSYRHYDDCYDDEYGLWNDTGCDNDDELGACGGHSLANDDVTY

ESEENWD

**Graphical representation – ORF**


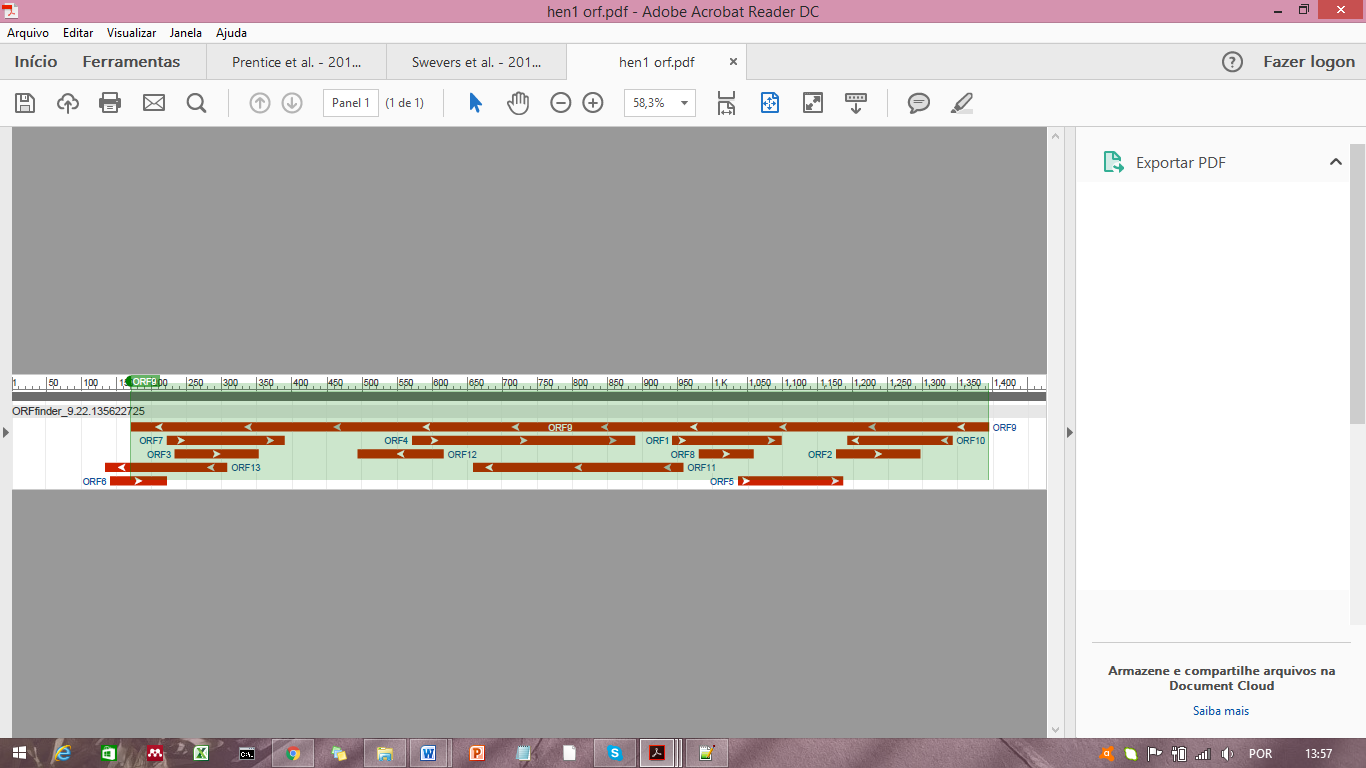


**BlastP (Non-redundant NCBI sequences)**

PREDICTED: small RNA 2'-O-methyltransferase isoform X1 [Rhagoletis zephyria]

Sequence ID: [XP_017463874.1](https://www.ncbi.nlm.nih.gov/protein/1048007305?report=genbank&log$=protalign&blast_rank=1&RID=WAGNBXAZ015) Length: 412 Number of Matches: 1

Range 1: 1 to 412

Score: 721 bits(1862)

E-value: 0.0

Identities: 350/412(85%)

Query 1 MFSYKLPDGGLRTCCYINAEGGIKFDPPVYEQRYTTAVRILEDSRWGQKFKKVVDFGCAD 60

MFSY+LPDGGLR CYIN+EG IKFDPPVYEQRYTTAVRILEDSRWG KFKKVVDFGCAD

Sbjct 1 MFSYELPDGGLRARCYINSEGDIKFDPPVYEQRYTTAVRILEDSRWGHKFKKVVDFGCAD 60

Query 61 MRLLPLLRRVKGIEHILEVDVDENILRSNKLRAEPLVSDYLMRRENPLRVELLKGSIDAS 120

MRLL LLRRV+GIE ILEVD+DE+ILRSNKLRAEPLVSDYL +RE PLRVELLKGSIDAS

Sbjct 61 MRLLSLLRRVEGIEQILEVDIDEDILRSNKLRAEPLVSDYLKKREGPLRVELLKGSIDAS 120

Query 121 VEQLLNVDAVVALEIIEHLYPKTLENVPKNIFGFMQPKIAIFSTPNSEFNVLFQPLLANG 180

VEQLLNVDAV+ALEIIEHLYPKTLENVPKNIFGFMQPKIAIFSTPNSEFNVLF+PLL NG

Sbjct 121 VEQLLNVDAVIALEIIEHLYPKTLENVPKNIFGFMQPKIAIFSTPNSEFNVLFEPLLENG 180

Query 181 FRHDDHKFEWTRAEFRDWARTICQQYPNYSVAFLGVGDAPPDNKSIGNVSQIAIFARHDL 240

FRHDDHKFEWTR EF+DWA ICQ+Y NYSVAF+GVGDAPPD K IG V+QIAIFAR+DL

Sbjct 181 FRHDDHKFEWTRTEFKDWALNICQKYHNYSVAFIGVGDAPPDKKDIGYVTQIAIFARNDL 240

Query 241 LGRRLVEPLRSEVPVDCNTDADVQYKEIFAVDFPFNVDERSKEKKILDEARYQIDRCRRI 300

L R L EP SEVPVDC+ DADVQYKEIFAVDFP+N DERSKE+KILDEARYQIDRCRRI

Sbjct 241 LDRSLTEPFSSEVPVDCDPDADVQYKEIFAVDFPYNRDERSKEQKILDEARYQIDRCRRI 300

Query 301 DRYFNQDLGVYQVPLTVMDTYIETLGASTEELLDVLKDNDIEVKGDYIILPEYDEDDLSY 360

DRYFN+DLGVYQVPLTV+ +IETLGAS +ELL +LK+NDIEVKGDYIILPEYDEDDLSY

Sbjct 301 DRYFNRDLGVYQVPLTVLKDFIETLGASMQELLAILKENDIEVKGDYIILPEYDEDDLSY 360

Query 361 RHYDDCYDDEYGLWNDTGC-----DNDDELGACGGHSLANDDVTYESEENWD 407

RHYDDCYDDEYGLWND GC +NDDELGACGG+S DD TY+SEENWD

Sbjct 361 RHYDDCYDDEYGLWNDVGCNDDETENDDELGACGGYSHTKDDATYDSEENWD 412

**RNA helicase Belle**

**>lcl|ORF24**

MSNAINQNGTGLEQQVAGLDLNGGKANNSSPITTKSSSNSGVYIPPHLRG

GGNSAPNANSDNREERTPSSKFEGREQRGGGGGEYRRGGGGRGYNNSGGG

YGGGGRRGGGGRYEENSGGFEVEGETRRGGEDWNRGGRGPQNSRTFDRRE

NGGYRGGRNQGQGGGAGTGNTSNRNSETFDDQAQPQQPRNDRWQEPERRP

EDGAQQRNERGGGGGGNGGERNYGGRWKEDRRGDIDYTKLGPRDERVETE

LFGVGNTGINFDKYEDIPVEATGQNVPPNIVSFDDVQLTEIVRNNVMLAR

YDKPTPVQKYAIPIIINGRDLMACAQTGSGKTAAFLLPILNQMYEHGMTP

PPQNNRQYSRRKQYPLGLVLAPTRELATQIFEEAKKFAYRSRMRPAVLYG

GNNTSEQMRELDRGCHLIVATPGRLEDMITRGKVGLDNIRFLVLDEADRM

LDMGFEPQIRRIVEQSNMPPTGQRQTLMFSATFPKQIQELASDFLSNYIF

LAVGRVGSTSENITQTILWVYEQDKRSYLLDLLSSIRAGAEYSKDSLTLI

FVETKKGADALEEFLYQCNHPVTSIHGDRTQKEREEALRCFRSGDCPILV

ATAVAARGLDIPHVKHVINFDLPSDVEEYVHRIGRTGRMGNLGVATSFFN

EKNRNICGDLLELLVETKQEVPGFLEEMLSSDRTHSGNRRRGGGGVGRYG

GGFGSRDYRQTSGGGGGGGGPRSGGGGGPPRSGGGGGGGSYRSNGGSSGG

GYYGGSGGGGGGGGSYGGSYSASHANSNSGPDWWGS

**Graphical representation – ORF**


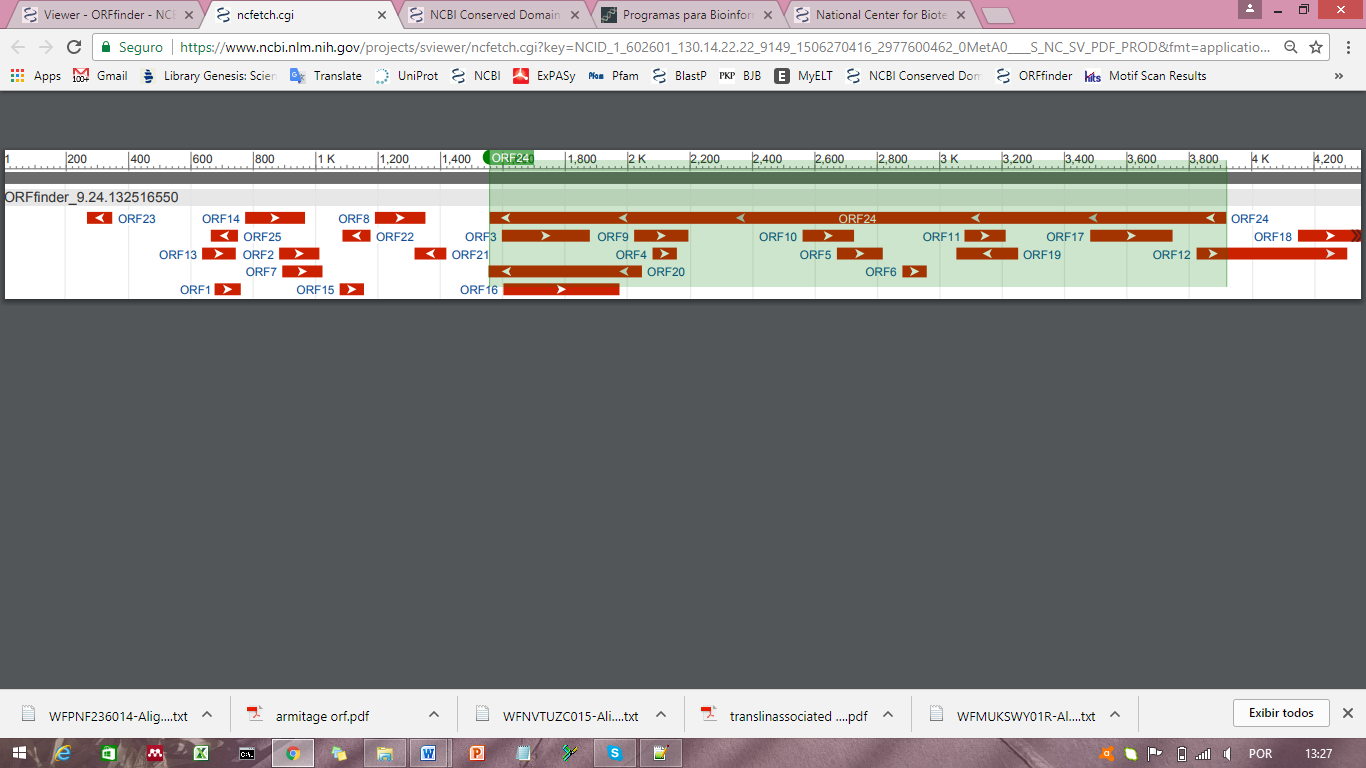


**BlastP (Non-redundant NCBI sequences)**

PREDICTED: ATP-dependent RNA helicase bel [Bactrocera latifrons]

Sequence ID: [XP_018803329.1](https://www.ncbi.nlm.nih.gov/protein/1098691965?report=genbank&log$=protalign&blast_rank=1&RID=WFR9EYJB015) Length: 777 Number of Matches: 1

Range 1: 1 to 679

Score: 1191 bits(3080)

E-value: 0.0

Identities: 614/690(89%)

Query 1 MSNAINQNGTGLEQQVAGLDLNGGKANNSSPITTKSSSNSGVYIPPHLRGGGN---SAPN 57

MSNAINQNGTGLEQQVAGLDLNGGKANNSSPITTKSSSNSG+YIPPHLR GG APN

Sbjct 1 MSNAINQNGTGLEQQVAGLDLNGGKANNSSPITTKSSSNSGIYIPPHLREGGEGGIIAPN 60

Query 58 ANSDNREERTPSSKFEGREQRGGGGGEYRRGGGGRGYNNSGGGYGGGGRRGGGGRYEENS 117

ANSDNREE PS+K+E REQRGGG EYRRG GGRG GG G R GG RYE+N+

Sbjct 61 ANSDNREEGAPSTKYEVREQRGGG--EYRRGVGGRG---YNNSGGGYGARRGGNRYEDNN 115

Query 118 GGFEVEGETR--RGGEDWNRGGRGPQNSRTFDRRENGGYRGGRNQGQGGGAGTGNTSNRN 175

G + +GE+R RGG+DWNRGGRG QN+RTFDRRENGG R GGAG G+ SNRN

Sbjct 116 SGVDGDGESRQQRGGDDWNRGGRGQQNTRTFDRRENGG---YRGGRGQGGAGGGSNSNRN 172

Query 176 SETFDDQAQPQQPRNDRWQEPERRPEDGAQQRNERGGGGGGNGGERNYGGRWKEDRRGDI 235

SE FDDQ QPQQPRNDRWQEPERRPED QQRN+RGG GGG ERNYGGRWKEDRR DI

Sbjct 173 SEAFDDQTQPQQPRNDRWQEPERRPEDAGQQRNDRGGSGGG---ERNYGGRWKEDRRADI 229

Query 236 DYTKLGPRDERVETELFGVGNTGINFDKYEDIPVEATGQNVPPNIVSFDDVQLTEIVRNN 295

DYTKLGPRDERVE ELFGVGNTGINFDKYEDIPVEATGQNVP NI SFDDVQLTEI+RNN

Sbjct 230 DYTKLGPRDERVEQELFGVGNTGINFDKYEDIPVEATGQNVPSNITSFDDVQLTEIIRNN 289

Query 296 VMLARYDKPTPVQKYAIPIIINGRDLMACAQTGSGKTAAFLLPILNQMYEHGMTPPPQNN 355

VMLARYDKPTPVQKYAIPIIINGRDLMACAQTGSGKTAAFLLPILNQMYEHG+TPPPQNN

Sbjct 290 VMLARYDKPTPVQKYAIPIIINGRDLMACAQTGSGKTAAFLLPILNQMYEHGITPPPQNN 349

Query 356 RQYSRRKQYPLGLVLAPTRELATQIFEEAKKFAYRSRMRPAVLYGGNNTSEQMRELDRGC 415

RQYSRRKQYPLGLVLAPTRELATQIFEEAKKFAYRSRMRPAVLYGGNNTSEQMRELDRGC

Sbjct 350 RQYSRRKQYPLGLVLAPTRELATQIFEEAKKFAYRSRMRPAVLYGGNNTSEQMRELDRGC 409

Query 416 HLIVATPGRLEDMITRGKVGLDNIRFLVLDEADRMLDMGFEPQIRRIVEQSNMPPTGQRQ 475

HLIVATPGRLEDMITRGKVGLDNIRFLVLDEADRMLDMGFEPQIRRIVEQ NMPPTGQRQ

Sbjct 410 HLIVATPGRLEDMITRGKVGLDNIRFLVLDEADRMLDMGFEPQIRRIVEQLNMPPTGQRQ 469

Query 476 TLMFSATFPKQIQELASDFLSNYIFLAVGRVGSTSENITQTILWVYEQDKRSYLLDLLSS 535

TLMFSATFPKQIQELASDFLSNYIFLAVGRVGSTSENITQTILWVYEQDKRSYLLDLLSS

Sbjct 470 TLMFSATFPKQIQELASDFLSNYIFLAVGRVGSTSENITQTILWVYEQDKRSYLLDLLSS 529

Query 536 IRAGAEYSKDSLTLIFVETKKGADALEEFLYQCNHPVTSIHGDRTQKEREEALRCFRSGD 595

IR G EYSKDSLTLIFVETKKGADALEEFLYQCNHPVTSIHGDRTQKEREEALRCFRSGD

Sbjct 530 IREGVEYSKDSLTLIFVETKKGADALEEFLYQCNHPVTSIHGDRTQKEREEALRCFRSGD 589

Query 596 CPILVATAVAARGLDIPHVKHVINFDLPSDVEEYVHRIGRTGRMGNLGVATSFFNEKNRN 655

CPILVATAVAARGLDIPHVKHVINFDLPSDVEEYVHRIGRTGRMGNLGVATSFFNEKNRN

Sbjct 590 CPILVATAVAARGLDIPHVKHVINFDLPSDVEEYVHRIGRTGRMGNLGVATSFFNEKNRN 649

Query 656 ICGDLLELLVETKQEVPGFLEEMLSSDRTH 685

ICGDLLELL+ETKQE+P FLE+M SSDR+H

Sbjct 650 ICGDLLELLIETKQEIPVFLEDMSSSDRSH 679

**PRP16**

**>lcl|ORF7**

MDALQIYPISQANANQRSGRAGRTGPGQAFRLYTQRQYKEDLLALTVPEI

QRTNLANTVLLLKSLGVVDLLQFHFMDPPPQDNILNSLYQLWILGALDHT

GALTPLGRQMAEFPLDPPQCQMLIVSCQMECSAEVLIIVSMLSVPSIFYR

PKGREEEADGVREKFQVPESDHLTYLNVYLQWKQNNYNSSWCNEHFIHVK

AMRKVREVRQQLKDIMVQQKLNVKSCGTDWDIVRKCICSAYFYQAARLKG

IGEYVNLRTGMPCHLHPTSALYGLGTTPDYVVYHELVMTAKEYMQCATAV

DGYWLAELGPMFFSVKETGRSGREKKKQAAEHLKEMETQMQLAQEQMEER

KLQAAQREEQMTPKQEIITPGGATPRRTPARIGL

**Graphical representation – ORF**


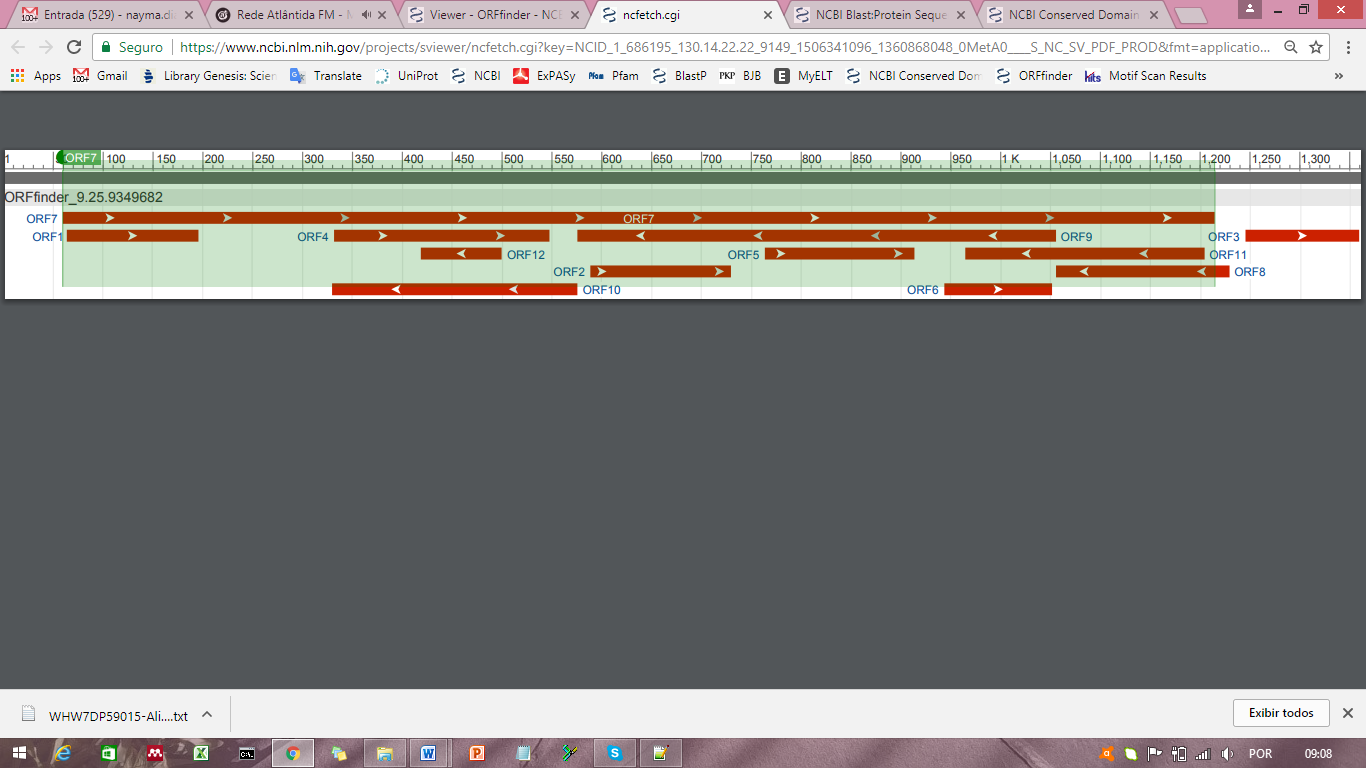


**BlastP (Non-redundant NCBI sequences)**

PREDICTED: pre-mRNA-splicing factor ATP-dependent RNA helicase PRP16 [Bactrocera latifrons]

Sequence ID: [XP_018784837.1](https://www.ncbi.nlm.nih.gov/protein/1098634226?report=genbank&log$=protalign&blast_rank=2&RID=WHW7DP59015) Length: 1257 Number of Matches: 1

Range 1: 874 to 1257

Score: 813 bits(2101)

E-value: 0.0

Identities: 381/384(99%)

Query 1 MDALQIYPISQANANQRSGRAGRTGPGQAFRLYTQRQYKEDLLALTVPEIQRTNLANTVL 60

MDALQIYPISQANANQRSGRAGRTGPGQAFRLYTQRQYKEDLL LTVPEIQRTNLANTVL

Sbjct 874 MDALQIYPISQANANQRSGRAGRTGPGQAFRLYTQRQYKEDLLPLTVPEIQRTNLANTVL 933

Query 61 LLKSLGVVDLLQFHFMDPPPQDNILNSLYQLWILGALDHTGALTPLGRQMAEFPLDPPQC 120

LLKSLGVVDLLQFHFMDPPPQDNILNSLYQLWILGALDHTGALTPLGRQMAEFPLDPPQC

Sbjct 934 LLKSLGVVDLLQFHFMDPPPQDNILNSLYQLWILGALDHTGALTPLGRQMAEFPLDPPQC 993

Query 121 QMLIVSCQMECSAEVLIIVSMLSVPSIFYRPKGREEEADGVREKFQVPESDHLTYLNVYL 180

QMLIVSC+MECSAEVLIIVSMLSVPSIFYRPKGREEEADGVREKFQVPESDHLTYLNVYL

Sbjct 994 QMLIVSCKMECSAEVLIIVSMLSVPSIFYRPKGREEEADGVREKFQVPESDHLTYLNVYL 1053

Query 181 QWKQNNYNSSWCNEHFIHVKAMRKVREVRQQLKDIMVQQKLNVKSCGTDWDIVRKCICSA 240

QWKQNNYNSSWCNEHFIHVKAMRKVREVRQQLKDIM+QQKLNVKSCGTDWDIVRKCICSA

Sbjct 1054 QWKQNNYNSSWCNEHFIHVKAMRKVREVRQQLKDIMIQQKLNVKSCGTDWDIVRKCICSA 1113

Query 241 YFYQAARLKGIGEYVNLRTGMPCHLHPTSALYGLGTTPDYVVYHELVMTAKEYMQCATAV 300

YFYQAARLKGIGEYVNLRTGMPCHLHPTSALYGLGTTPDYVVYHELVMTAKEYMQCATAV

Sbjct 1114 YFYQAARLKGIGEYVNLRTGMPCHLHPTSALYGLGTTPDYVVYHELVMTAKEYMQCATAV 1173

Query 301 DGYWLAELGPMFFSVKETGRSGREKKKQAAEHLKEMETQMQLAQEQMEERKLQAAQREEQ 360

DGYWLAELGPMFFSVKETGRSGREKKKQAAEHLKEMETQMQLAQEQMEERKLQAAQREEQ

Sbjct 1174 DGYWLAELGPMFFSVKETGRSGREKKKQAAEHLKEMETQMQLAQEQMEERKLQAAQREEQ 1233

Query 361 MTPKQEIITPGGATPRRTPARIGL 384

MTPKQEIITPGGATPRRTPARIGL

Sbjct 1234 MTPKQEIITPGGATPRRTPARIGL 1257

**Gemin3**

**>lcl|ORF9**

MEGECVAHSLDGKERTEDIKLKELSPFSKMLLSESIRKGLQKTGFIYPTA

IQAMSIPMGKSGQDLLIQSKSGTGKTLIFCVIILEAYRADLSEPQSLIVA

PTREIAVQIEMVLNQIGSCCNGFRAVSVIGGLDVAEDRKRLQGAKAVVGT

PGRLLHLIQNNVLNTSKMRLLVLDEADKMYTQSFRQDLRRIQNALPAKRQ

TVACSATFCDGLDVELAKIMRNPLLISTEERATLLIGIKQFVYELPDQKT

SILEMQSKLEGLRFIFGRIAFKQCLIFAGSQSRANSYCNYLEKEGWPCEL

ISGAQDQKTRLEMFHKFREFKSRVIITTDLMSRGVDSEHVNLVINLELPT

DMVTYLHRIGRAGRFGSHGIAINFVATEKERCILSRLISRIGNGMSVLKF

PQKQIFQEGEVDFWDFTNLEKDKEYFGQFGCEPLAPLASQDRSSFSDTQE

YIENQTDNFLNSSSSIDTNTFRVDSKDSALNIQENLLERPEYNQQALSPS

HKLTIDSRHGSSQQLPSSNTIDDASSIAADSLRSSQQDISRYQSVLMHKA

AIPTLFEFLVDPNSSESKMDAKEGETKKKPQLDLFDDYKRTVLTNSNSSL

KETEEKNMEIPVISHKIATYKSMLIDQPVPTKLVENKHDIYSDYANFNTE

ENSSSLSQETGSNKIDEGTFILSVATPSKVQDSIEEFVNSTTTSDLKNSR

SISDSLEVSKVKHFTSPAHNQTNNAIPDVIPACQRLMGMPYNSQQYHNLS

VNIGAHIEIPPHQPSPANSPALNDSIVHSQDEDHSPSISGSDSLNSRSNS

ERGGSSGFDEHNTTSVSSGIVTSDTEPERYVENGYYSSPDTETASSYEVD

PELLFPYAETYEESGEEEEDEEDDNEVKVEDEVENDEAGDENDELDDGDD

KERNDSEHCNEQTPNAVSTESGTATNKDNEENRSQEKNSAKASMKPTHSR

GHGKKTHSQKAREAQNDTHSSQCNAYGSNYEAAYALWMEMYRTQLKQIND

YVQMTLEAGKLKDEK

**Graphical representation – ORF**


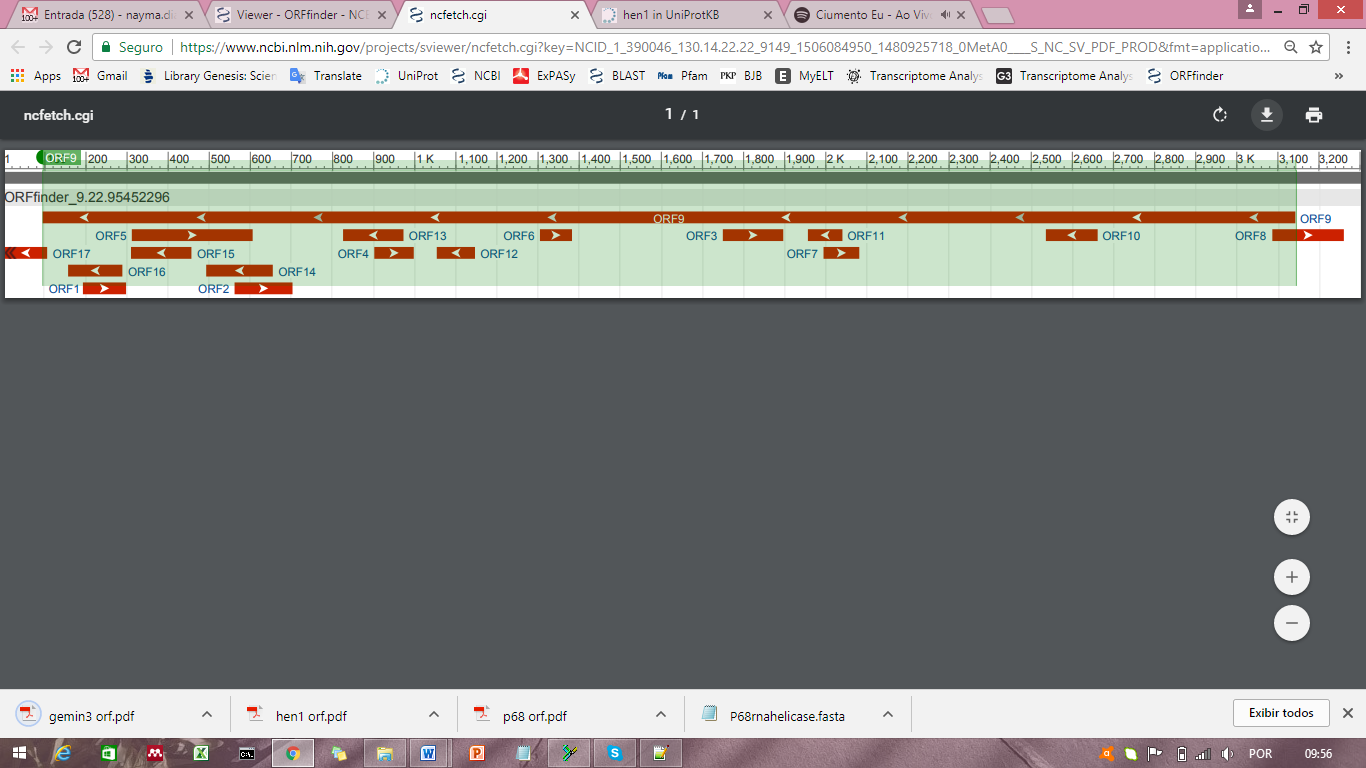


**BlastP (Non-redundant NCBI sequences)**

PREDICTED: probable ATP-dependent RNA helicase DDX20 [Rhagoletis zephyria]

Sequence ID: [XP_017473008.1](https://www.ncbi.nlm.nih.gov/protein/1048024739?report=genbank&log$=protalign&blast_rank=1&RID=WA2GH3KY014) Length: 1040 Number of Matches: 1

Range 1: 1 to 863

Score: 1488 bits(3851)

E-value: 0.0

Identities: 737/867(85%)

Query 1 MEGECVAHSLDGKERTEDIKLKELSPFSKMLLSESIRKGLQKTGFIYPTAIQAMSIPMGK 60

ME VAHSL+GK RTEDIKLKEL+PFSKMLLSES+RKGLQKTGF+YPTAIQAMSIPMGK

Sbjct 1 MEAGNVAHSLEGKVRTEDIKLKELAPFSKMLLSESVRKGLQKTGFVYPTAIQAMSIPMGK 60

Query 61 SGQDLLIQSKSGTGKTLIFCVIILEAYRADLSEPQSLIVAPTREIAVQIEMVLNQIGSCC 120

SG DLL+QSKSGTGKTLIFC IILEAYR DL EPQSLIV PTREIA+QIEMVLNQIGSCC

Sbjct 61 SGLDLLVQSKSGTGKTLIFCTIILEAYRMDLLEPQSLIVVPTREIAIQIEMVLNQIGSCC 120

Query 121 NGFRAVSVIGGLDVAEDRKRLQGAKAVVGTPGRLLHLIQNNVLNTSKMRLLVLDEADKMY 180

+ FRAVSVIGGLDVAEDRKRLQ AKAVVGTPGRLLHLIQNNVLNTSKMRLLVLDEADKMY

Sbjct 121 SNFRAVSVIGGLDVAEDRKRLQSAKAVVGTPGRLLHLIQNNVLNTSKMRLLVLDEADKMY 180

Query 181 TQSFRQDLRRIQNALPAKRQTVACSATFCDGLDVELAKIMRNPLLISTEERATLLIGIKQ 240

TQSFRQDLRRIQNALPAK+QT+ACSATFCDGLD ELAKIMRNPLLISTEERATLL+GIKQ

Sbjct 181 TQSFRQDLRRIQNALPAKKQTIACSATFCDGLDKELAKIMRNPLLISTEERATLLVGIKQ 240

Query 241 FVYELPDQKTSILEMQSKLEGLRFIFGRIAFKQCLIFAGSQSRANSYCNYLEKEGWPCEL 300

F YE+P+QKTSILEMQSKLEGLR IFGR+AFKQCLIFAGSQSRANSYCNYLEKEGWPCEL

Sbjct 241 FAYEIPEQKTSILEMQSKLEGLRVIFGRVAFKQCLIFAGSQSRANSYCNYLEKEGWPCEL 300

Query 301 ISGAQDQKTRLEMFHKFREFKSRVIITTDLMSRGVDSEHVNLVINLELPTDMVTYLHRIG 360

ISGAQDQKTRL MFHKFREFKSR+IITTDLMSRGVDSEHVNLVINLELP DMVTYLHRIG

Sbjct 301 ISGAQDQKTRLAMFHKFREFKSRIIITTDLMSRGVDSEHVNLVINLELPNDMVTYLHRIG 360

Query 361 RAGRFGSHGIAINFVATEKERCILSRLISRIGNGMSVLKFPQKQIFQ-EGEVDFWDFTNL 419

RAGRFGSHGIAINFVA+EK+RC L+RLISRIGNGM+VLKFPQ+QI Q E E D WDF+NL

Sbjct 361 RAGRFGSHGIAINFVASEKDRCTLTRLISRIGNGMNVLKFPQRQIAQTEQEYDIWDFSNL 420

Query 420 EKDKEYFGQFGCEPLAPLASQDRSSFSDTQEYIENQTDNFLNSSSSIDTNTFRVDSKDSA 479

EKD+ YFGQFGCEPLAPLASQD SSFSDTQE ENQTDNF+N+SSSIDTNTFRVDSKDSA

Sbjct 421 EKDQNYFGQFGCEPLAPLASQDHSSFSDTQENKENQTDNFINTSSSIDTNTFRVDSKDSA 480

Query 480 LNIQENLLERPEYNQQALSPSHKLTIDSRHGSSQQLPSSNTIDDASSIAADSLRSSQQDI 539

LNIQENLLERPE N LSPSHKLTIDSR GSSQQLPSSNTIDDASSIAADSLRSSQQDI

Sbjct 481 LNIQENLLERPECNHLILSPSHKLTIDSRQGSSQQLPSSNTIDDASSIAADSLRSSQQDI 540

Query 540 SRYQSVLMHKAAIPTLFEFLVDPNSSESKMDAKEGETKKKPQLDLFDDYKRTVLTNSNSS 599

SRYQSVLM KAAIPTLFEFLVDPNS ES+MDAKEGE KKKP +DLFDDYK+ VLTNS++S

Sbjct 541 SRYQSVLMQKAAIPTLFEFLVDPNSCESQMDAKEGEAKKKPPIDLFDDYKKAVLTNSDNS 600

Query 600 LKETEEKNMEIPVISHKIATYKSMLIDQPVPTKLVENKHDIYSDYANFNTEENSSSLSQE 659

LKETEE +E IS+KI YKSMLIDQPV L ENKHDIYSDYANFN EE SSSLSQE

Sbjct 601 LKETEETGLE-SHISNKITAYKSMLIDQPVQNNLAENKHDIYSDYANFNAEEYSSSLSQE 659

Query 660 TGSNKIDEGTFILSVATPSKVQDSIEEFVNST-TTSDLKNSRSISDSLEVSKVKHFTSPA 718

TGSN+ID+GTFILSVATPS++QDSIEEFVN+T T SDLKNS+SIS+SLEVS+VKHFTSPA

Sbjct 660 TGSNEIDKGTFILSVATPSRMQDSIEEFVNTTETASDLKNSQSISESLEVSEVKHFTSPA 719

Query 719 HNQTNNAIPDVIPACQRLMGMPYNSQQYHNLSVNIGAHIEIPPHQPSPANSPALNDSIVH 778

HNQ NN IPDV+P CQR M YN + HNL+VN+GA IEIPPHQ S ++SPALNDS++

Sbjct 720 HNQPNNTIPDVVPPCQR---MSYNLKHIHNLTVNVGAQIEIPPHQQSHSDSPALNDSVLR 776

Query 779 SQDEDHSPSISGSDSLNSRSNSERGGSSGFDEHNTTSVSSGIVTSDTEPERYVENGYYSS 838

S++ED SP+IS S+SLNSRSNSERGGSSGF+E NTTS SSGIVTSDTEPERYV+NGYYSS

Sbjct 777 SREEDPSPTISASNSLNSRSNSERGGSSGFNERNTTSASSGIVTSDTEPERYVQNGYYSS 836

Query 839 PDTETASSYEVDPELLFPYAETYEESG 865

+T T SSYEVDPELLFPY ++ +ESG

Sbjct 837 SETGTGSSYEVDPELLFPYPDSSKESG 863

**Gawky**

**>lcl|ORF11**

MLAANLPVCQYAGMPGQSNSNNGGTSNNNNSSTNNTKTNNSSSCNSGTSG

PNSNNNNGGISTGSVGLVGGGTSAAAAKNQLEQLNTMREALFSQDGWGCQ

HVNQDTNWEVPSSPEPTNKDPSGGPTMWKPTINNGTDLWELNLRNGGQPP

TQPVQKTPWGHTPSSNLGGTWGEDDDGADSSSVWTGSSNNPSTAGVVGVT

GVSSTGVGANGPGNGPQWGQSSVGVGVGAAANATGTPSVNVGSVSVVGGP

AGGPGVNVPGSVNAAAANNSAGNNWSDSREMSSGAVSGAIAMRGVDPRDQ

MRGTAAEPRELRMLDPRDPIRGDPRGISGRLNGTSEMWGQHHSIAHNQIP

ALNKIVGPGVSVGSGVSTGAVVTGGGVAGLGAGGVNAINAGGVSGSVNAH

WGASSALVGPKDISSMTGKVTGWEEPSPPPQRRNIPNYDDGTSLWGQQSR

VPSGSHWKDISDPMNRHLTRNTVGNQNTPNTVAGLGSGSGSGLGNNQGVL

NAPVVGGNTNNSISSVGPPGRLGSGVGPGIAVNQHKPDNTMWVHASNPNV

NARNAASWGDEANHNMGPNSGVNWMDDKTSAGIGQLGVSVGGTNSWNDPS

ASWNKNQNKMAGGATGWGTGGGNDGADLSSDWSAHGGIVGKTQQKIGGVN

VNITNLNTDVIKQSKQYRVLVENGFKKEDVERALISANMNIEEAADLLRA

NATMSMDWRRHEDPLGSYADHPNPGGSFPGRYPPTATQPTMPFPPNILNN

MSGNTVSASGNNSNLAALNSIQPLPVQKYLNSAPHNVASGPQTMNPAVAF

GQGVANVTAAVNAASNSTSQPSTQQLRVLVQQIQLAVHSGYLSSQILSEP

LPSSMLVLLYQLLTNIKHLQAAQQSLTRGGNNANNSQISYAIAKYKQQIQ

NLQNQINAQQAFILKQKQQSMPQNAQHSAAHVNSSNLEYIRGQHDAINAL

QSNFSEMNLAKHSGYPTGPNSQSKLINQWKLPEKDITSESTDFSRAPGAS

KQNLNASGNTMGPLGLQNDGTWSTGRSIGDGWPDSSTETENKDWSVAQPT

PAATYTDLVQEFEPGKPWKGSQIKSIEDDPSITPGSVARSPLSINSTPKE

ADIFANPSKSSPTDLTPLSLSSSTWSFNPSSSNQNFQSWSDSPQQCTPSE

LWASSMNKTSRGPPPGLGSGKGAGVAGTGVQSVTNSSSVVAGSSNGWIVT

GGRGVPNNNSGWTTSNSGWNSTWLLLKNLTAQIDGSTLRTLCMQHGPLAN

FHLYLNQGIALCKYATREEANKAQMTLNNCVLGSTTICAESPSENEVQSI

LQHLPQTSNSVSGSGLTGNGGNSSNPTSNSGGSVAGGNGSLSSNSGNNNG

GPCNNVGGVSGGNGNCSMANHSVVSGSGGNGCGSNAMNNGSTGGNGNSGN

NQSQPSSGACSSNGGKSGNSNISSGNSSSNATSNSVVGNNSVVAAPAWRQ

TQSQPRPSGRDEYDYISKFVCSIVDD

**Graphical representation - ORF**


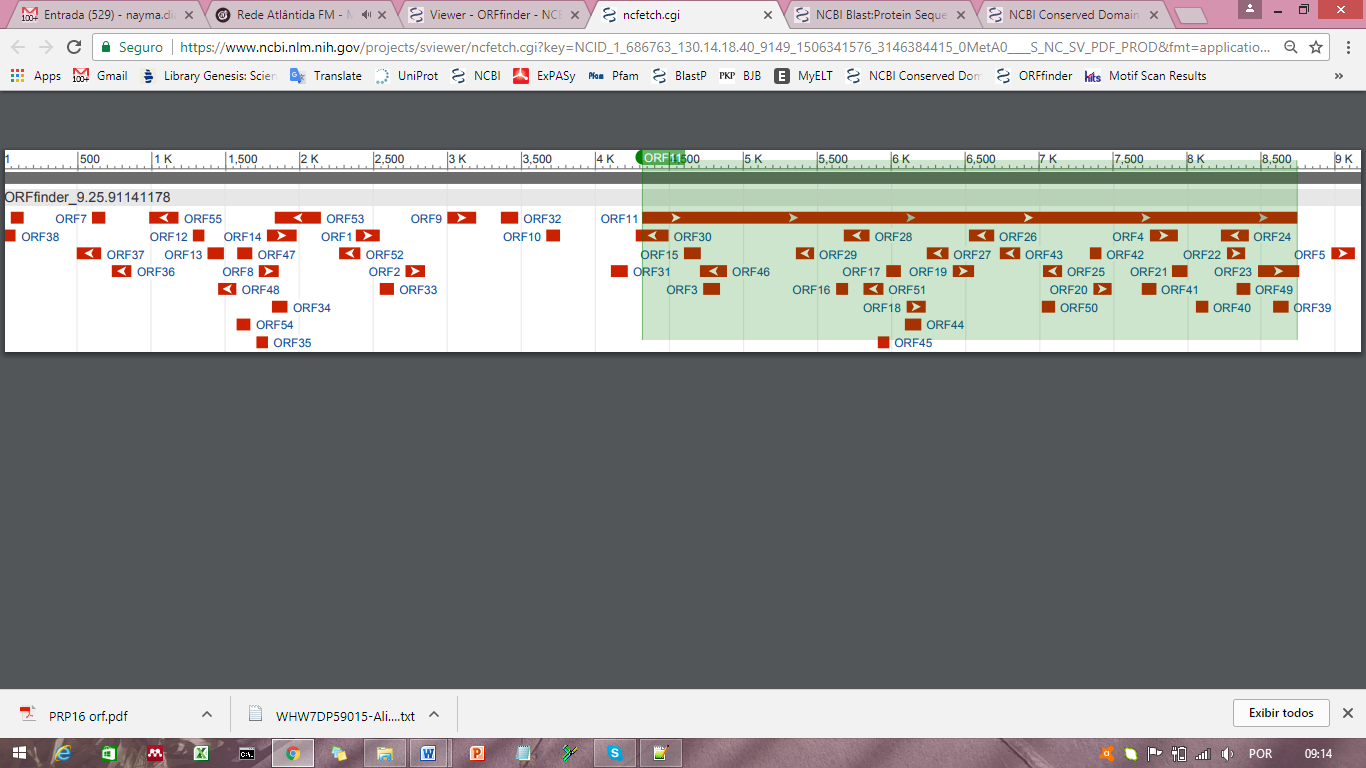


**BlastP (Non-redundant NCBI sequences)**

PREDICTED: protein Gawky isoform X2 [Rhagoletis zephyria]

Sequence ID: [XP_017467541.1](https://www.ncbi.nlm.nih.gov/protein/1048013221?report=genbank&log$=protalign&blast_rank=1&RID=WHWPED26015) Length: 1483 Number of Matches: 1

Range 1: 1 to 1483

Score: 2248 bits(5824)

E-value: 0.0

Identities: 1312/1486(88%)

Query 1 MLAANLPVCQYAGMPGQSNSNNGGTSNNNNSSTNNTKTNNSSSCNSGTSGPNSNNNNGGI 60

MLAANLPVCQYAGMPGQSNSNNGGT+NNNN S NNTKTNN+ S NSG S NSNNNN G

Sbjct 1 MLAANLPVCQYAGMPGQSNSNNGGTNNNNNGSANNTKTNNTGSGNSGPSISNSNNNNVGN 60

Query 61 STGSVGLVGGGTSAAAAKNQLEQLNTMREALFSQDGWGCQHVNQDTNWEVPSSPEPTNKD 120

S +VGLVGGGTSAAAAKNQLEQLNTMREALFSQDGWGCQHVNQDTNWEVPSSPEPTNKD

Sbjct 61 SNSNVGLVGGGTSAAAAKNQLEQLNTMREALFSQDGWGCQHVNQDTNWEVPSSPEPTNKD 120

Query 121 PSGGPTMWKPTINNGTDLWELNLRNGGQPPTQPVQKTPWGHTPSSNLGGTWGEDDDGADS 180

PSG PTMWKPTINNGTDLWELNLRNGGQPPTQPVQKTPWGHTPSSNLGGTWGEDDDGADS

Sbjct 121 PSGAPTMWKPTINNGTDLWELNLRNGGQPPTQPVQKTPWGHTPSSNLGGTWGEDDDGADS 180

Query 181 SSVWTGSSNNPSTAGVVGVTGVSSTGVGANGPGNGPQWGQSSVGVGVGAAANATGTPSVN 240

SSVWTGSSNNP++AG VGV GVSSTGV AN P NGPQWGQSSVGVGVG + NA G PSVN

Sbjct 181 SSVWTGSSNNPTSAGAVGVAGVSSTGVAANVPVNGPQWGQSSVGVGVGTSGNAAGAPSVN 240

Query 241 VGSVSVVGGPAGGPGVNVPGSVNAAAANNSAGNNWSDSREMSSGAVSGAIAMRGVDPRDQ 300

+GSVS VG GGPGVNV GSVN+ +A+ SAGNNWSDSREMSSG VSG IAMRGVDPRDQ

Sbjct 241 IGSVSAVGSATGGPGVNVTGSVNSVSASGSAGNNWSDSREMSSGTVSGVIAMRGVDPRDQ 300

Query 301 MRGT-AAEPRELRMLDPRDPIRGDPRGISGRLNGTSEMWGQHHSIAHNQIPALNKIVGPG 359

+RGT AAEPR+LRMLDPRDPIRGDPRGISGRLNGTSEMWGQHHS+ HNQIPA+NKIVG

Sbjct 301 IRGTSAAEPRDLRMLDPRDPIRGDPRGISGRLNGTSEMWGQHHSMTHNQIPAMNKIVG-P 359

Query 360 VSVGSGVSTGAVVTGGGVAGLGAGGVNAINAGGVSGSVNAHWGASSALVGPKDISSMTGK 419

VG+GVSTGAVV G GVAG+G GGVNAIN GGVSG+VNAHWGASSA+VGPKDI+SM GK

Sbjct 360 GGVGAGVSTGAVVAGAGVAGIGPGGVNAINPGGVSGNVNAHWGASSAVVGPKDITSMAGK 419

Query 420 VTGWEEPSPPPQRRNIPNYDDGTSLWGQQSRVPSG--SHWKDISDPMNRHLTRNTVGNQN 477

VTGWEEPSPPPQRRNIPNYDDGTSLWGQQSRV G SHWKDISDPMNRHLTRN VGNQN

Sbjct 420 VTGWEEPSPPPQRRNIPNYDDGTSLWGQQSRVSGGGSSHWKDISDPMNRHLTRNAVGNQN 479

Query 478 TPNTVAGLGSGSGSGLGNNQGVLNAPVVGGNTNNSISSVGPPGRLGSGVGPGIAVNQHKP 537

T NTVAGLGSG+G+GLG NQGVLNAPVVGGNTNN ISSVGP GRLGSGVGPG VNQHKP

Sbjct 480 TTNTVAGLGSGAGNGLGGNQGVLNAPVVGGNTNNPISSVGPQGRLGSGVGPGAGVNQHKP 539

Query 538 DNTMWVHASNPNVNARNAASWGDEANHNMGPNSGVNWMDDKTSAGIGQLGVSVGGTNSWN 597

DNTMWVHASNPNVNARNAA+WGDE NHN+GPNSGVNWMDDKT+AGIGQLGVSVGG NSWN

Sbjct 540 DNTMWVHASNPNVNARNAAAWGDETNHNVGPNSGVNWMDDKTNAGIGQLGVSVGGANSWN 599

Query 598 DPSASWNKNQNKMAGGATGWGTGGGNDGADLSSDWSAHGGIVGKTQQKIGGVNVNITNLN 657

DP ASWNK+QNKM GA GWG GNDG DLS+DW+ HGGIVGKTQQKIG VNVNI NLN

Sbjct 600 DPPASWNKSQNKMPVGAAGWGAATGNDGTDLSTDWNGHGGIVGKTQQKIGSVNVNIGNLN 659

Query 658 TDVIKQSKQYRVLVENGFKKEDVERALISANMNIEEAADLLRANATMSMDWRRHEDPLGS 717

TDVIKQSKQYRVLVENGFKKEDVERALI+ANMNIEEAADLLRANATM+MDWRRHE+ LGS

Sbjct 660 TDVIKQSKQYRVLVENGFKKEDVERALINANMNIEEAADLLRANATMAMDWRRHEETLGS 719

Query 718 YADHPNPGGSFPGRYPPTATQPTMPFPPNILNNMSGNTVSASGNNSNLAALNSIQPLPVQ 777

Y DHPNP GSFPGRYPPTATQPTMPFPPNILNNMSGN VSASGNNSNLAALNS+QPLPVQ

Sbjct 720 YGDHPNP-GSFPGRYPPTATQPTMPFPPNILNNMSGNAVSASGNNSNLAALNSLQPLPVQ 778

Query 778 KYLNSAPHNVASGPQTMNPAVAFGQGVANVTAAVNAASNSTSQPSTQQLRVLVQQIQLAV 837

KYLNSAPHNVASGPQTMNPAVAFGQGVANVTAAVNAASNS SQPS QQLRVLVQQIQLAV

Sbjct 779 KYLNSAPHNVASGPQTMNPAVAFGQGVANVTAAVNAASNSNSQPSAQQLRVLVQQIQLAV 838

Query 838 HSGYLSSQILSEPLPSSMLVLLYQLLTNIKHLQAAQQSLTRGGNNANNSQISYAIAKYKQ 897

HSGYLSSQILSEPLPSSMLVLLYQLLTNIKHLQAAQQSLTRGGNNANNSQISYAIAKYKQ

Sbjct 839 HSGYLSSQILSEPLPSSMLVLLYQLLTNIKHLQAAQQSLTRGGNNANNSQISYAIAKYKQ 898

Query 898 QIQNLQNQINAQQAFILKQKQQSMPQNA-QHSAAHVNSSNLEYIRGQHDAINALQSNFSE 956

QIQNLQNQINAQQAFILKQKQQSMPQN QH AAH NSSNLEYIRGQHDAI+ALQ NFSE

Sbjct 899 QIQNLQNQINAQQAFILKQKQQSMPQNTQQHPAAHANSSNLEYIRGQHDAISALQGNFSE 958

Query 957 MNLAKHSGYPTGPNSQSKLINQWKLPEKDITSESTDFSRAPGASKQNL-NASGNTMGPLG 1015

MNL+KHSGYP GPNSQSKLINQWKLPEKD+TSESTDFSRAPGA+KQNL +A GNTMGPLG

Sbjct 959 MNLSKHSGYPGGPNSQSKLINQWKLPEKDMTSESTDFSRAPGATKQNLSSAPGNTMGPLG 1018

Query 1016 LQNDGTWSTGRSIGDGWPDSSTETENKDWSVAQPTPAATYTDLVQEFEPGKPWKGSQIKS 1075

LQNDGTWSTGRSIGDGWPDSSTETENKDWSVAQPTPAATYTDLVQEFEPGKPWKGSQIKS

Sbjct 1019 LQNDGTWSTGRSIGDGWPDSSTETENKDWSVAQPTPAATYTDLVQEFEPGKPWKGSQIKS 1078

Query 1076 IEDDPSITPGSVARSPLSINSTPKEADIFANPSKSSPTDLTPLSLSSSTWSFNPSSSNQN 1135

IEDDPSITPGSVARSPLSINSTPKEADIFANPSKSSPTDLTPLSLSSSTWSFNPSSSNQN

Sbjct 1079 IEDDPSITPGSVARSPLSINSTPKEADIFANPSKSSPTDLTPLSLSSSTWSFNPSSSNQN 1138

Query 1136 FQSWSDSPQQCTPSELWASSMNKTSRGPPPGLGSGKGAGVAGTGVQSVTNSSSVVAGSSN 1195

FQSWSDSPQQC PSELWA+SMNKTSRGPPPGLGS KGAGV+ TGV SVTN SSVVAGSSN

Sbjct 1139 FQSWSDSPQQCAPSELWATSMNKTSRGPPPGLGSSKGAGVSVTGVPSVTN-SSVVAGSSN 1197

Query 1196 GWIVTGGRGVPNNNSGWTTSNSGWNSTWLLLKNLTAQIDGSTLRTLCMQHGPLANFHLYL 1255

GW+VTGGRGVPN+ SGW T+NSGWNSTWLLLKNLTAQIDGSTLRTLCMQHGPLANFHLYL

Sbjct 1198 GWVVTGGRGVPNSTSGWNTANSGWNSTWLLLKNLTAQIDGSTLRTLCMQHGPLANFHLYL 1257

Query 1256 NQGIALCKYATREEANKAQMTLNNCVLGSTTICAESPSENEVQSILQHLPQTSNSVSGSG 1315

NQGIALCKYATREEANKAQMTLNNCVLGSTTICAESPSENEVQSILQHLPQTSN+VSG G

Sbjct 1258 NQGIALCKYATREEANKAQMTLNNCVLGSTTICAESPSENEVQSILQHLPQTSNTVSGCG 1317

Query 1316 LTGNGGNSSNPTSNSGGSVAGGNGSLSSNSGNNNGGPCNNVGGVSGGNGNCSMANHSVV- 1374

L GNGGN +N SNSG SV+GGNGS+SS+SGNNN GPC +VGGVSGGN N SMA HSV

Sbjct 1318 LIGNGGNGANNASNSGVSVSGGNGSISSSSGNNNVGPCGSVGGVSGGNVNGSMATHSVAS 1377

Query 1375 SGSGGNGCGSNAMNNGSTGGNGNSGNNQSQPSSGAC-SSNGGKSGNSNISSGNSSSNATS 1433

G NGCGSNAMN+GSTGGNGN N+Q+QP SGAC S++G ++NI+SGN SSNATS

Sbjct 1378 GSGGNNGCGSNAMNSGSTGGNGNGSNSQNQPVSGACSSNSGKNGNSNNITSGNGSSNATS 1437

Query 1434 NSVVGNNSVVAAPAWRQT---QSQPRPSGRDEYDYISKFVCSIVDD 1476

NSVVG+N+ VAAPAWRQT Q+QPRPSGRDEYDYISKFVCSIVDD

Sbjct 1438 NSVVGSNNAVAAPAWRQTQNPQNQPRPSGRDEYDYISKFVCSIVDD 1483

**Staufen**

**>lcl|ORF30**

MPPKAILQNPNRVLKARLHSSAAANNIEVPTVGPLVSIEETKNVSKSSSN

NNIGSSNAVAVTETPAKAHVASNVAAAVTDGVVVAAAAAAQESLKIVQST

SQTDASFNDANKENGVSRTQSTNVTVNEVVSNNPANNNNSNILSNEESAG

SKNSKEKTPMCLVNELARFNKITHQYRLTSEKGPAHCKRFTVTLKLGEEE

YSAEGFKIKKAQHLAAAEAIEKTKYKHPVPKVVRRNAEGEGHSSRSNITP

TVELNALAMKLGEQTYYLLNPRQVTSPDRIGPPPDAMMPPRYGIPLPPPH

AFPPGVAGGNMLSQHPPPPHARMMDPAAFVRRNGPYAAPPPPPMHGHTAG

GPGMRPRYLTPAQRAFMPKYNQQQRYPLMAPPHMGGAPHNGMVPPPPMPH

PHHGPLPHRYGISAALTKVTLVVGKQKFVGLGRTLQQAKHDAAARALQVL

KTQAKTQISEELNNSIEESDSKSPISLVHEIGIQRSLTVHFKVLREEGPA

HMKKFVTACVVGSIVTEGEGNGKKTSKKRAAEKMLEELKKLPPMSPTKSP

VRRIKVKTPNKGSASADAAGGVSGKAGSAVGERRKRGSGSIKEKNEVDAD

AENPITRLIQLQQTRKEKEPIFDLIAKNGNENSRRREFIMEVTAHGMVAR

GTGNSKKLAKRNAAQNLLTAMGESGSISAENNTVLASSNTEPAVKEQFAS

ATTVVATVTASQEQTTTTTVLQPIAEVANAAATTAAPVQAHLDLPLVSTT

AGQVPGILILRHNKKHFTKKKEYVPSANCLPDEDREIERAELVVGTAVPT

VQPTAVKSNASDIVKGIDVGTAASATPPSMAATAAAHTESSNITNTASST

TNSDTTSSSNSASKQTGVHMKDQLLYLAKLLGFEVNFSDYPKGNHSEFLT

IVTLSSNPPQICHGVGSSAEESQNDAAKNALKILSELGLNNATK

**Graphical representation - ORF**


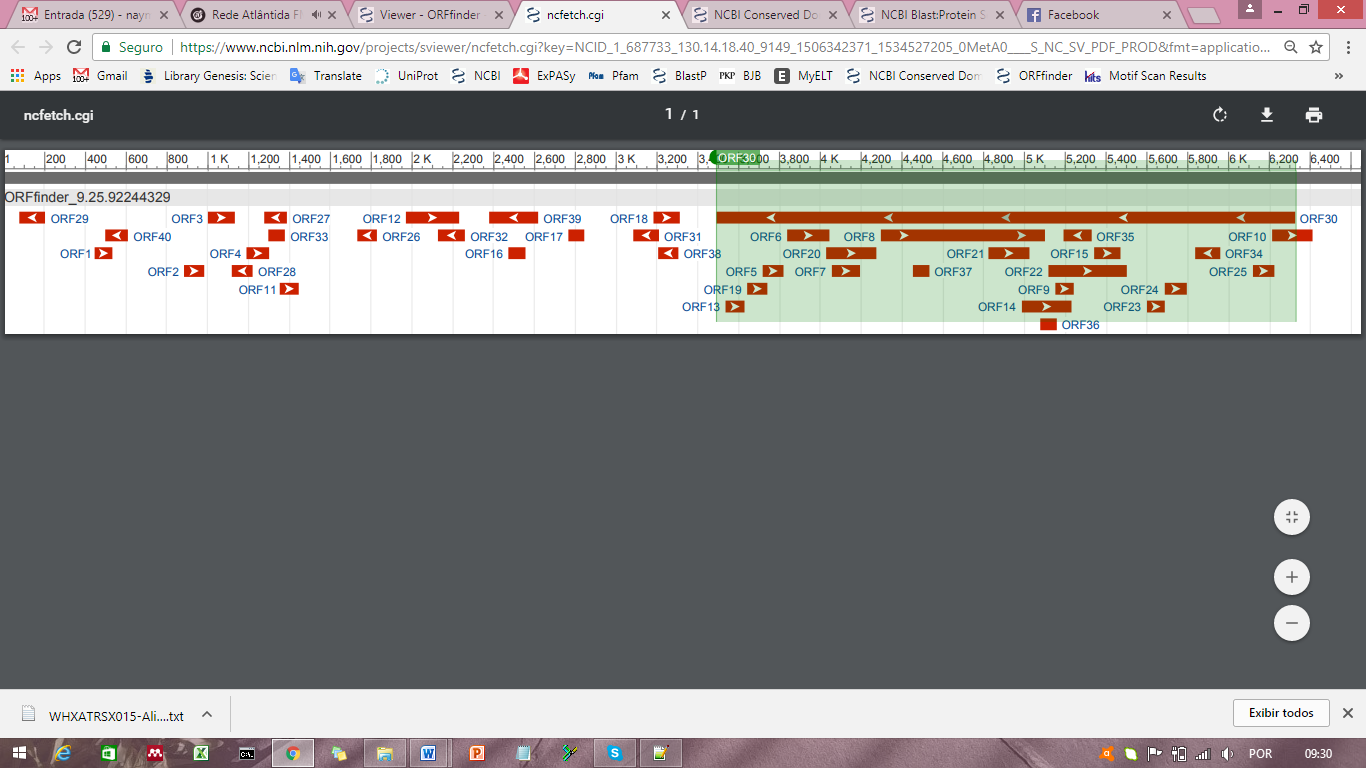


**BlastP (Non-redundant NCBI sequences)**

PREDICTED: maternal effect protein staufen isoform X2 [Rhagoletis zephyria]

Sequence ID: [XP_017481555.1](https://www.ncbi.nlm.nih.gov/protein/1048054907?report=genbank&log$=protalign&blast_rank=1&RID=WHXATRSX015) Length: 1170 Number of Matches: 1

Range 1: 224 to 1170

Score: 1169 bits(3025)

E-value: 0.0

Identities: 682/959(71%)

Query 1 MPPKAILQNPNRVLKARLHSSAAANNIEVPTVGPLVSIEETKNVSKSSSNNNIGSSNAVA 60

M PK ILQNPNR+LKARLHSSAAANNIEV T+GPLVSIEET VSKSS+ ++ G N V

Sbjct 224 MQPKTILQNPNRILKARLHSSAAANNIEVTTIGPLVSIEETNKVSKSSNISDGGGVNNV- 282

Query 61 VTETPAKAHVASNVAAAVTDGVVVAAAAAAQESLKIVQSTSQTDASFNDANKENGVSRTQ 120

A V + QE K Q+TSQ DAS NDA KEN S TQ

Sbjct 283 ---VSAAEASVKANVVVAVTASAVIPSNIRQECTKNGQNTSQNDASINDAKKENVTSGTQ 339

Query 121 STNVTVNEVVSNNPANNNNSNILSNEESAGSKNSKEKTPMCLVNELARFNKITHQYRLTS 180

N ++ S++ A +NN+NILSNE++ KNSKEKTPMCLVNELARFNKITHQYRLTS

Sbjct 340 --NAKISNEASSSNATSNNNNILSNEDNTVPKNSKEKTPMCLVNELARFNKITHQYRLTS 397

Query 181 EKGPAHCKRFTVTLKLGEEEYSAEGFKIKKAQHLAAAEAIEKTKYKHPVPKVVRRNAEGE 240

EKGPAHCKRFTVTLKLG+EEYSAEGFKIKKAQHLAAAEAIEKTKYKHPVPKV+RRN +GE

Sbjct 398 EKGPAHCKRFTVTLKLGDEEYSAEGFKIKKAQHLAAAEAIEKTKYKHPVPKVIRRNTDGE 457

Query 241 GHSSRSNITPTVELNALAMKLGEQTYYLLNPRQVTSPDRIGPPPDAMMPPRYGIPLPPPH 300

G SSR+NITPTVELNALAMKLG+QTYYLL+PRQV SPD +GPPPDAMMPPRYG+P PP H

Sbjct 458 GSSSRANITPTVELNALAMKLGQQTYYLLDPRQVASPDSMGPPPDAMMPPRYGVPPPPSH 517

Query 301 AFPP-GVAGGNMLSQHPPPPHARMM---DPAAFVRRNGPYAAPPPPPMHGHTAGGPGMRP 356

A PP G+ G+M+ QH PPP + DP A++RRNGPYA PP PP+HGH GPGMRP

Sbjct 518 ALPPPGMGSGHMMPQHAPPPPPPHVHMMDPPAYLRRNGPYAVPPAPPLHGHPTSGPGMRP 577

Query 357 RYLTPAQRAFMPKYNQQQRYPLMAPPHMGGAPHNGMVPPPPMPHPHH----GPLPHRYGI 412

RY P QRA+MPKY QQQRY LMAPPHM G PHNGM PPPPMPH H G +PHRYG+

Sbjct 578 RYGAPPQRAYMPKYGQQQRYALMAPPHMAGGPHNGMAPPPPMPHHPHHPHHGSVPHRYGM 637

Query 413 SAALTKVTLVVGKQKFVGLGRTLQQAKHDAAARALQVLKTQAKTQISEELNNSIEESDSK 472

AALT+VTLVVGKQKFVG+G TLQQAKHDAA+RALQVLK QA TQ++E LNNS+EESDSK

Sbjct 638 PAALTRVTLVVGKQKFVGMGYTLQQAKHDAASRALQVLKAQAATQLNEALNNSLEESDSK 697

Query 473 SPISLVHEIGIQRSLTVHFKVLREEGPAHMKKFVTACVVGSIVTEGEGNGKKTSKKRAAE 532

SPISLVHEIGIQRS+TVHFKVLREEGPAHMKKFVTACVVGSIVTEGEGNGKKTSKKRAAE

Sbjct 698 SPISLVHEIGIQRSMTVHFKVLREEGPAHMKKFVTACVVGSIVTEGEGNGKKTSKKRAAE 757

Query 533 KMLEELKKLPPMSPTKSPVRRIKVKTPNKGSASADAAGGVSGKAGSAVGERRKRGSGSIK 592

KMLEELKKLPP++PTKSPV+RIKVKTP+K +A+AD AGG SGK+G A GERRKRGSGSIK

Sbjct 758 KMLEELKKLPPLTPTKSPVKRIKVKTPSKAAANADGAGGGSGKSGGAGGERRKRGSGSIK 817

Query 593 EKNEVDADAENPITRLIQLQQTRKEKEPIFDLIAKNGNENSRRREFIMEVTAHGMVARGT 652

EK+E +ADAENPIT+LIQLQQ RKEKEP+F+LIAKNGNENSRRREFI+EV+AHGMVARGT

Sbjct 818 EKSEAEADAENPITQLIQLQQNRKEKEPVFELIAKNGNENSRRREFIIEVSAHGMVARGT 877

Query 653 GNSKKLAKRNAAQNLLTAMGESGSISAENNTVLASSNTE-PAVKEQFASA-TTVVATVTA 710

GNSKKLAKRNAAQNLL AMGES + +A NT A + + A KE++A A T + V++

Sbjct 878 GNSKKLAKRNAAQNLLIAMGESDTDTAGVNTASAPATIQTAAAKERYARAPATAIDGVSS 937

Query 711 SQEQTTTTTVLQPIAEVANAAATTAAPVQAHLDLPLVSTTAGQVPGILILRHNKKHFTKK 770

EQ TVLQPIAEV A +AHLDLPLVST AGQVPGILILRHNKK+ KK

Sbjct 938 HSEQ--PNTVLQPIAEVPPPAVA----AEAHLDLPLVSTPAGQVPGILILRHNKKNAVKK 991

Query 771 KEYVPSANCLPDEDREIER-AELVVGTAVPTVQPTAVKSNASDIVKGIDVGTAASATPPS 829

KE PSA+C+ E+ E +R A+L +G V VQ AVKS A+DIVK +V A SA PPS

Sbjct 992 KECAPSASCVMAENNENKRAADLTIGAGVQAVQQPAVKSAATDIVKESEVEIALSANPPS 1051

Query 830 MAATAAAHTESSN----ITNTASSTTNSDTTSSSNSASKQTGVHMKDQLLYLAKLLGFEV 885

A ++N +++ SS T + T +S+SASK GVHMKDQLLYLAKLLGFEV

Sbjct 1052 AVVQPVAAPITNNAQKAVSSAPSSATTTTTARASSSASKPAGVHMKDQLLYLAKLLGFEV 1111

Query 886 NFSDYPKGNHSEFLTIVTLSSNPPQICHGVGSSAEESQNDAAKNALKILSELGLNNATK 944

NFSDYPKGNHSEFLTIVTLSS PPQICHGVGSSAEESQNDAA+NALKILSELGLNNA K

Sbjct 1112 NFSDYPKGNHSEFLTIVTLSSEPPQICHGVGSSAEESQNDAARNALKILSELGLNNAAK 1170

**Clip 1**

**>lcl|ORF34**

MAYRKPNDLDGFIQMMPKADMRVKALLAEDLVTFLSDEANSIVCMDMGML

VDGLMPWLTGSHFKIAQKSLEAFSELIKRLGPDFNAYSATVLPHVIDRLG

DSKDTVREKAQLLLQIVMEYKVISPQATIDKLAVACFKHKNAKVREEFLQ

TIVNTLNEYGTSQLSVRTYIQPTSALLGDPTATVRDAAMQTLVEIYKHVG

DRLRVDLRKMDDVPASKLAVLEQKFDQIKAEGLLLKSALLTNPSVNNGHD

ESDNVSVRDRPTKIVKRTISASTRNKPSSADSSASGDAGAVTMEIFESSF

EIVPQLTIFHPKDMDDIYRNIIVVISDKNADWEKRIDSLKKVRSLLMLNI

HSQPQFVAQLKDLSIPFIDILKEELRSQVIREACITIAYMSKTLRNKLEP

FCLAILEALINLIQNSAKVIASSSTIALKYIIKYTHSSKIIKLVTETLQQ

AKSKDIRAALCEMLCLMFDEWQTKTMERCSQQLRDVLKRSISDADNEARR

HSRRAYWKFRRHFPDLADQIYTTLDIASQRALERERDGGNVLVEAERRSA

AATTRFQRSPGSLQKPAAGMRSVSAVDTAAAQRAKARAQYSFYPRKKLST

VSNTAGTVSSTGPAATGSLPRPRYMGGVTPAAAATTAQSNVGISPRTRGR

AGVSQSQPGSRSTSPSSKLREQYGYRPITGTIPKKASGIPRSLTSSRETS

PTRVAMKRSIYSTNSSMGSARRTPERSNSRPLTATRILQQSREAENALAD

ALSPEGERTIDYGEYSRGYTAGLRMGRKLLSRDESDDSEASSVCSERSFD

SSMTRGNNSNYSLSGSRNRLDWSCTRAPFDDIDTIIQYCASTHWSDRKDG

VISLTQYLDDGNQLTAQQLQAVLDMFRKLFMDPHTKVYALFLDAVTELIQ

THANELHDWLFILLTRLFNKLGTEMLNSMHIKIGKTLHIVHQYFPTDQQL

RDVFRILADTAQTPCTKTKIAILKFLTDLVTSYCKSTDFPSDDGPLAVDK

AVLKIVQQAGDPKSKDLRDQARKCLIALYNRSTPQMTKLLSSLPKGYQDT

AKAIIQSHLRRNSTSGTNSPSSPHSSASPKLQSPSLGPFSSLQPQFNTSP

RSRQSSVDHELYSEADVQHNIHKTSEEIRNCFGVGVGIEPVSNNTRQYHS

LSNANGYNGYVHDQQDSCASSNSKTQSATTTESNTPESTTMRLDANLLEQ

HQRMTTNVGLSSATTNSNTAVVSGTTHHGSRCNYTVASNGELVLESGLTE

SEIIRVACALKVDMPVEQLQQGLANLEICIKGGNCELPNKHFRAIMKMLL

GLLDSQTADVMIAVISVLGKIVRSTKMKETWINFLELILLRIINCYQHSK

ETAREIDLIIPRIVSSLPLNATINIVNPVIATSCYPMNLCAVKLLTELAD

RHGSELTEFHLDSIFPNLARLTDDSESMVRKAAVFCIVKLYIVMGEEKVK

PKLSVLNPSKVRLLNVYIDKQRGNSGGGSSTKNSSASSS

**Graphical representation - ORF**
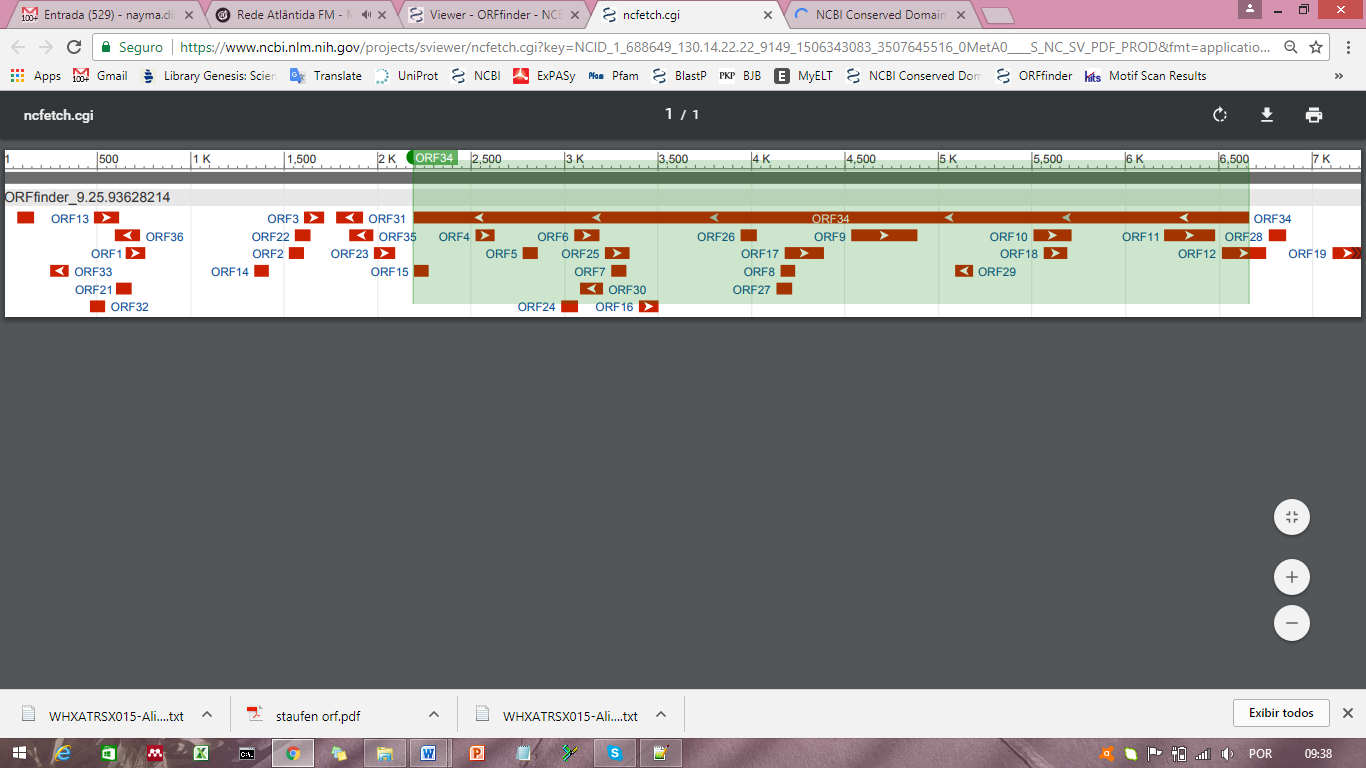


**BlastP (Non-redundant NCBI sequences)**

PREDICTED: CLIP-associating protein [Bactrocera dorsalis]

Sequence ID: [XP_011212830.1](https://www.ncbi.nlm.nih.gov/protein/751805219?report=genbank&log$=protalign&blast_rank=1&RID=WHY95SE9015) Length: 1493 Number of Matches: 1

Range 1: 1 to 1482

Score: 2805 bits(7271)

E-value: 0.0

Identities: 1359/1482(92%)

Query 1 MAYRKPNDLDGFIQMMPKADMRVKALLAEDLVTFLSDEANSIVCMDMGMLVDGLMPWLTG 60

MAYRKPNDLDGFIQMMPKADMRVKALLAEDLVTFLSDE NSIVCMDMGMLVDGLMPWLTG

Sbjct 1 MAYRKPNDLDGFIQMMPKADMRVKALLAEDLVTFLSDETNSIVCMDMGMLVDGLMPWLTG 60

Query 61 SHFKIAQKSLEAFSELIKRLGPDFNAYSATVLPHVIDRLGDSKDTVREKAQLLLQIVMEY 120

SHFKIAQKSLEAFSELIKRLGPDFNAYSATVLPHVIDRLGDSKDTVREKAQLLLQ+VMEY

Sbjct 61 SHFKIAQKSLEAFSELIKRLGPDFNAYSATVLPHVIDRLGDSKDTVREKAQLLLQVVMEY 120

Query 121 KVISPQATIDKLAVACFKHKNAKVREEFLQTIVNTLNEYGTSQLSVRTYIQPTSALLGDP 180

KV++PQATIDKLA +CFKHKN+KVREEFLQTIVNTLNEYGTSQLSVRTYIQP SALLGDP

Sbjct 121 KVLTPQATIDKLAASCFKHKNSKVREEFLQTIVNTLNEYGTSQLSVRTYIQPISALLGDP 180

Query 181 TATVRDAAMQTLVEIYKHVGDRLRVDLRKMDDVPASKLAVLEQKFDQIKAEGLLLKSALL 240

TATVRDAA+QTLVEIYKHVGDRLR DLRKMDDVPASKLA LEQKFDQIKAEGLLLKSAL

Sbjct 181 TATVRDAAIQTLVEIYKHVGDRLRADLRKMDDVPASKLASLEQKFDQIKAEGLLLKSALQ 240

Query 241 TNPSVNNGHDESDNVSVRDRPTKIVKRTISASTRNKPSSADSSASGDAGAVTMEIFESSF 300

T S NNGHDESDNVSVRDRPTKIVKRT+SAS R KP+S+DSSA GDAGAVTM+IFES+F

Sbjct 241 TTASANNGHDESDNVSVRDRPTKIVKRTVSASMRAKPNSSDSSAGGDAGAVTMDIFESTF 300

Query 301 EIVPQLTIFHPKDMDDIYRNIIVVISDKNADWEKRIDSLKKVRSLLMLNIHSQPQFVAQL 360

E+VPQLTIFHPKDMDDIYRNIIVVISDKNADWEKRIDSLKKVRSLLMLNI SQPQFVAQL

Sbjct 301 EVVPQLTIFHPKDMDDIYRNIIVVISDKNADWEKRIDSLKKVRSLLMLNIQSQPQFVAQL 360

Query 361 KDLSIPFIDILKEELRSQVIREACITIAYMSKTLRNKLEPFCLAILEALINLIQNSAKVI 420

KDLSIPF+DILKEELRSQVIREACITIAYMSKTLRNKLEPFCLAILEALINLIQNSAKVI

Sbjct 361 KDLSIPFLDILKEELRSQVIREACITIAYMSKTLRNKLEPFCLAILEALINLIQNSAKVI 420

Query 421 ASSSTIALKYIIKYTHSSKIIKLVTETLQQAKSKDIRAALCEMLCLMFDEWQTKTMERCS 480

ASSS IALKYIIKY+HS K+IKL+TETLQQ+KSKDIRA LCEMLCLMFDEWQTKTMERCS

Sbjct 421 ASSSIIALKYIIKYSHSPKMIKLITETLQQSKSKDIRATLCEMLCLMFDEWQTKTMERCS 480

Query 481 QQLRDVLKRSISDADNEARRHSRRAYWKFRRHFPDLADQIYTTLDIASQRALERERDGGN 540

QQLRDVLKRSISDADNEARRHSRRAYWKFRRHFPDLADQIYTTLDIA+QRALERERDGGN

Sbjct 481 QQLRDVLKRSISDADNEARRHSRRAYWKFRRHFPDLADQIYTTLDIAAQRALERERDGGN 540

Query 541 VLVEAERRSAAATTRFQRSPGSLQKPAAGMRSVSAVDTAAAQRAKARAQYSFYPRKKLST 600

+E ERRSAAATTRFQRSPGSLQKPAAGMRSVSAVDTAAAQRAKARAQYSFYPRKKLST

Sbjct 541 GTMEVERRSAAATTRFQRSPGSLQKPAAGMRSVSAVDTAAAQRAKARAQYSFYPRKKLST 600

Query 601 VSNTAGTVSS--TGPAATGSLPRPRYMGGVTPAAAATTAQSNVGISPRTRGRAGVSQSQP 658

V+ A + +S T AATGSLPRPRYMGG + + T Q G+SPRTRGRAGVSQSQP

Sbjct 601 VNTAAASANSGTTTSAATGSLPRPRYMGGGATSTSNTGGQLAAGVSPRTRGRAGVSQSQP 660

Query 659 GSRSTSPSSKLREQYGYRPITGTIPKKASGIPRSLTSSRETSPTRVAMKRSIYSTNSSMG 718

GSRSTSPSSKLREQYGYRPITGTIPKKASGIPRSLTSSRETSPTRVAMKRSIY+TNSS

Sbjct 661 GSRSTSPSSKLREQYGYRPITGTIPKKASGIPRSLTSSRETSPTRVAMKRSIYATNSSAS 720

Query 719 SARRTPERSNSRPLTATRILQQSREAENALADALSPEGERTIDYGEYSRGYTAGLRMGRK 778

SARRTP+RSNSR L A RILQQSREAE ALADALSPE ER IDYGEYSRGYTAGLRMGRK

Sbjct 721 SARRTPDRSNSRSLAAARILQQSREAETALADALSPETERVIDYGEYSRGYTAGLRMGRK 780

Query 779 LLSRDESDDSEASSVCSERSFDSSMTRGNNSNYSLSGSRNRLDWSCTRAPFDDIDTIIQY 838

LLSRDESDDSEASSVCSERSFDSSMTRGNNSNYSLSGSRNRLDWSCTRAPFDDIDTIIQY

Sbjct 781 LLSRDESDDSEASSVCSERSFDSSMTRGNNSNYSLSGSRNRLDWSCTRAPFDDIDTIIQY 840

Query 839 CASTHWSDRKDGVISLTQYLDDGNQLTAQQLQAVLDMFRKLFMDPHTKVYALFLDAVTEL 898

CASTHWSDRKDGVISLTQYL DGNQLT+QQLQAVLDMFRKLFMDPHTKVY+LFLDAVTEL

Sbjct 841 CASTHWSDRKDGVISLTQYLADGNQLTSQQLQAVLDMFRKLFMDPHTKVYSLFLDAVTEL 900

Query 899 IQTHANELHDWLFILLTRLFNKLGTEMLNSMHIKIGKTLHIVHQYFPTDQQLRDVFRILA 958

I HAN+L DWLF+LLTRLFNKLGTE+LNSMHIKIGKTL +VH+YFPTDQQL+DVFRILA

Sbjct 901 ILAHANDLQDWLFVLLTRLFNKLGTELLNSMHIKIGKTLQVVHEYFPTDQQLKDVFRILA 960

Query 959 DTAQTPCTKTKIAILKFLTDLVTSYCKSTDFPSDDGPLAVDKAVLKIVQQAGDPKSKDLR 1018

DTAQTPCTKTKIAILKFLTDL TSYCKSTDFPSDDGPLAVDKAVLKIVQQAGDPKSKDLR

Sbjct 961 DTAQTPCTKTKIAILKFLTDLATSYCKSTDFPSDDGPLAVDKAVLKIVQQAGDPKSKDLR 1020

Query 1019 DQARKCLIALYNRSTPQMTKLLSSLPKGYQDTAKAIIQSHLRRNSTSGTN-SPSSPHSSA 1077

DQARKCLIALYNR+TPQMTKLLSSLPKGYQDTAKAIIQSHLRRNSTSGTN S S++

Sbjct 1021 DQARKCLIALYNRNTPQMTKLLSSLPKGYQDTAKAIIQSHLRRNSTSGTNSPSSPHSSAS 1080

Query 1078 SPKLQSPSLGPFSSLQPQFN-TSPRSRQSSVDHELYSEADVQHNIHKTSEEIRNCFGVGV 1136

LQSPSLGPFSSLQPQ+N TSPRSRQSSVDHELYSEADVQHNIHKTSEEIRNCFGVGV

Sbjct 1081 PKPLQSPSLGPFSSLQPQYNTTSPRSRQSSVDHELYSEADVQHNIHKTSEEIRNCFGVGV 1140

Query 1137 GIEPVSNNTRQYHSLSNANGYNGYVHDQQDSCASSNSKTQSATTTESNTPESTTMRLDAN 1196

G+E SNNTRQYHSLSNANGYNGY+HDQQDSCASSNSKTQSATTTESNTPESTTMRLDAN

Sbjct 1141 GMEATSNNTRQYHSLSNANGYNGYLHDQQDSCASSNSKTQSATTTESNTPESTTMRLDAN 1200

Query 1197 LLEQHQRMTTNVGLSSATTNSNTAVVSGTTHHGSRCNYTVASNGELVLESGLTESEIIRV 1256

LLEQHQRMTTN+ L++AT N+NTAVV GT +H SRCNYTVASNGEL+LE+GLTESEIIRV

Sbjct 1201 LLEQHQRMTTNIVLTTATPNANTAVVGGTANHASRCNYTVASNGELLLENGLTESEIIRV 1260

Query 1257 ACALKVDMPVEQLQQGLANLEICIKGGNCELPNKHFRAIMKMLLGLLDSQTADVMIAVIS 1316

ACALK DMPVEQ+QQ LANLEICIKGGNCELPNKHFRAIMKMLLGLLDSQTADVMIAVIS

Sbjct 1261 ACALKADMPVEQVQQALANLEICIKGGNCELPNKHFRAIMKMLLGLLDSQTADVMIAVIS 1320

Query 1317 VLGKIVRSTKMKETWINFLELILLRIINCYQHSKETAREIDLIIPRIVSSLPLNATINIV 1376

VLGKIVRSTKMKETWINFLELILLRIINCYQHSKETAREIDLIIPRIVSSLPLNATINIV

Sbjct 1321 VLGKIVRSTKMKETWINFLELILLRIINCYQHSKETAREIDLIIPRIVSSLPLNATINIV 1380

Query 1377 NPVIATSCYPMNLCAVKLLTELADRHGSELTEFHLDSIFPNLARLTDDSESMVRKAAVFC 1436

NPVIATSCYP+NLCAVKLLTELADRHGSELTE HLDSIFPNLARLTDDSESMVRKAAVFC

Sbjct 1381 NPVIATSCYPLNLCAVKLLTELADRHGSELTELHLDSIFPNLARLTDDSESMVRKAAVFC 1440

Query 1437 IVKLYIVMGEEKVKPKLSVLNPSKVRLLNVYIDKQRGNSGGG 1478

IVKLYIVMGEE+VKPKLSVLNPSKVRLLNVYIDKQR NSGGG

Sbjct 1441 IVKLYIVMGEERVKPKLSVLNPSKVRLLNVYIDKQRSNSGGG 1482

**Elp-1**

**>lcl|ORF1**

MRNLKLQSCKQLDLKVSNVKYLLLDPNASRKEADTLVYVVTDSEVNEIKT

STGSIKEIASVPGIVAAEYLALNNEICLATQAGEVLAVSPSTGTINECTF

CGVGLQCMAWSPDQEVAVFITNSGNVVVMTCTYDVITEHVLEEQCDPDSQ

FVNVGWGKKETQFHGTEGKAAAKTRNDFKPPAKVEELPQDVNITWRADGA

YFAVSFVSSEAGRMFKVFNKEGDLTYISERWNDLQPPIAWRPSGTWIAVP

QIFPNKSTVALFEKNGLRHREIVLPFSLMDELIRSLRWSNDSDILAIETL

DSTAQKQRLYFYTIANYHWYLKQVLTFDRTDPIAYHCWDQRIGEEKTLHV

WLESGRYLIYRWRFDFDRFARSGIVAVIDGKKLLLTDFSKAIVPPPMCSK

EIESDTYINACVISRNNSGNLQLCIYDANEQLQLFVANHVENSLAFEKVC

VLEKLQTYLGEYNTPPSELGNLFWFDENYLVATVNVNEKSKVLLLILDTG

AKTYDVVTALQLNSNAACSCMGFVTLEQCFVQTTDGKIQQLSQQNDTTLK

LDKTYQELEQAALQLEWHQTQSAESECLIALLQNRRLYIDSELVSGDVTS

FCLAGNYLAYTKLTELNFVLLSTRQHVYRRNMERGGRIVTTMANDARVVL

QMPRGNLEVISPRVLALEIIGKQLDQKRYSEAFNILRKQRINLNILCDHN

MISFVQNVTIFLSQITNPNWLNLFLTDLQNEDFSKTMYASNYTAAHQAYP

DGFKIESKVAYLCALLCARMAEINKERFRLPIITAYVKTGKLEQALQLIW

QVKKEQIELAKQSEGPVQEAAEEALKYLLYLVDVNELYNVALGTYDFGLV

LFVAQKSQKDPKEFLPFLNELKQLEVNYRRFKIDEHLKRYEKALENISKC

GVEKFSIALEFIQLHELYKLALKFYKIDVEENEAKEGQHSLRECQRQICL

AFADYLRAKNELESASIMYERGGNLTQALLSAKNVLDWRRVLMLAQKDGK

DIATAALSMVSALQEQGRYDIAHQLLKTYGTNFKEALQCLLKGNLYLAAI

LEVRMHCQQIDLLDEVVKPDLIAYSKQLAQQLADDEKLFVEHKQRLHDVR

VLAQKKRDGLVNCYDQPDIDEADLLSDTTSLRSSRYTGSSQGTGKTFRSS

KNRRKHERKLLSLKPGNPFEDIALIDALYNLVMKLANQQQQMRDTCKALI

ELQLDEVAGALQTQYGHLLTLMQDSFDAIWTDEMVNTQAMQYKPTPYTDY

TQLQNEQRYAVLAPQKRFKPQINLIDWKCKILS

**Graphical representation -** **ORF**


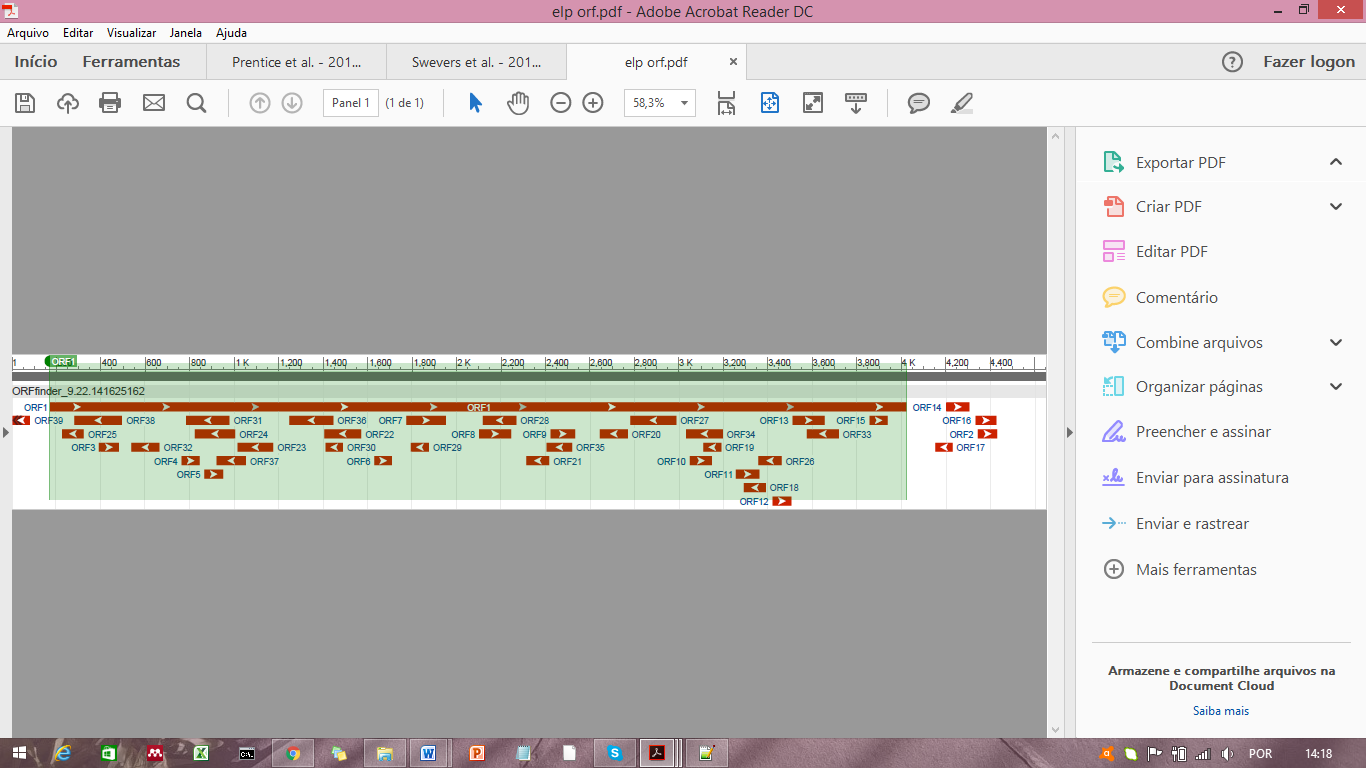


**BlastP (Non-redundant NCBI sequences)**

PREDICTED: putative elongator complex protein 1 [Rhagoletis zephyria]

Sequence ID: [XP_017487434.1](https://www.ncbi.nlm.nih.gov/protein/1048070924?report=genbank&log$=protalign&blast_rank=1&RID=WAHDCUMD014) Length: 1283 Number of Matches: 1

Range 1: 1 to 1283

Score: 2201 bits(5703)

E-value: 0.0

Identities: 1050/1283(82%)

Query 1 MRNLKLQSCKQLDLKVSNVKYLLLDPNASRKEADTLVYVVTDSEVNEIKTSTGSIKEIAS 60

MRNLKLQSCKQL KVSNVKYLLLDPNASRKE D+L +VVTDS++ E+KTSTG KEIA+

Sbjct 1 MRNLKLQSCKQLSSKVSNVKYLLLDPNASRKEIDSLAFVVTDSQLYEVKTSTGISKEIAA 60

Query 61 VPGIVAAEYLALNNEICLATQAGEVLAVSPSTGTINECTFCGVGLQCMAWSPDQEVAVFI 120

VPGIVAAEYLALNNEICLAT+AGEVLAVSPSTG INECTFC VGLQCM+WSPDQEV VFI

Sbjct 61 VPGIVAAEYLALNNEICLATEAGEVLAVSPSTGAINECTFCDVGLQCMSWSPDQEVGVFI 120

Query 121 TNSGNVVVMTCTYDVITEHVLEEQCDPDSQFVNVGWGKKETQFHGTEGKAAAKTRNDFKP 180

T SGNVVVMTCTYDVI EHVL+EQCDPDSQFVNVGWGKKETQFHG EGKAAAK DFKP

Sbjct 121 TKSGNVVVMTCTYDVIHEHVLKEQCDPDSQFVNVGWGKKETQFHGKEGKAAAKRTTDFKP 180

Query 181 PAKVEELPQDVNITWRADGAYFAVSFVSSEAGRMFKVFNKEGDLTYISERWNDLQPPIAW 240

P VE+LPQD+ ITWRADGAYFAVSFVS+EAGRMFKVFNKEGDL+YISE WNDLQPPIAW

Sbjct 181 PENVEQLPQDIEITWRADGAYFAVSFVSAEAGRMFKVFNKEGDLSYISEHWNDLQPPIAW 240

Query 241 RPSGTWIAVPQIFPNKSTVALFEKNGLRHREIVLPFSLMDELIRSLRWSNDSDILAIETL 300

RPSGTWIA+PQIFPNKSTV+LFEKNGLRHREIVLPFSL DE IRSLRWSNDSDILAIETL

Sbjct 241 RPSGTWIAMPQIFPNKSTVSLFEKNGLRHREIVLPFSLNDEPIRSLRWSNDSDILAIETL 300

Query 301 DSTAQKQRLYFYTIANYHWYLKQVLTFDRTDPIAYHCWDQRIGEEKTLHVWLESGRYLIY 360

D++ Q Q LY YTI NYHWYLKQVLTF+RT+ HCWDQRIGEEKTLH+WLESGRYLIY

Sbjct 301 DASTQNQNLYLYTIGNYHWYLKQVLTFNRTERFLCHCWDQRIGEEKTLHIWLESGRYLIY 360

Query 361 RWRFDFDRFARSGIVAVIDGKKLLLTDFSKAIVPPPMCSKEIESDTYINACVISRNNSGN 420

RWRF+FDR+ARSGIVAVIDGKKLLLTDFSKA+VPPPMCSKEI+SD YINACVIS+NN

Sbjct 361 RWRFEFDRYARSGIVAVIDGKKLLLTDFSKAVVPPPMCSKEIQSDAYINACVISKNNHEE 420

Query 421 LQLCIYDANEQLQLFVANHVENSLAFEKVCVLEKLQTYLGEYNTPPSELGNLFWFDENYL 480

LQLCIYDA+E+LQL+ SL FE+V V++K+Q L + PP EL N+FWF+EN L

Sbjct 421 LQLCIYDASEELQLYRVYRDGKSLDFERVSVMQKMQIELDLCSGPPLELANMFWFEENCL 480

Query 481 VATVNVNEKSKVLLLILDTGAKTYDVVTALQLNSNAACSCMGFVTLEQCFVQTTDGKIQQ 540

VATVN+ EKSKVLL+ LD Y + LQLNSNA SCMGFV LEQCFVQT +GK++Q

Sbjct 481 VATVNIGEKSKVLLITLDELVGAYSIAAVLQLNSNAVSSCMGFVMLEQCFVQTINGKVEQ 540

Query 541 LSQQNDTTLKLDKTYQELEQAALQLEWHQTQSAESECLIALLQNRRLYIDSELVSGDVTS 600

+S + +LKLDKTYQ+LEQ ALQ+EWHQTQSA+ +CLIALL N+RLYIDS+LVSGDVTS

Sbjct 541 ISLHSANSLKLDKTYQQLEQNALQMEWHQTQSAQGDCLIALLNNQRLYIDSQLVSGDVTS 600

Query 601 FCLAGNYLAYTKLTELNFVLLSTRQHVYRRNMERGGRIVTTMANDARVVLQMPRGNLEVI 660

FCLAGNYLAYTKLTELNFVLLSTRQ+VY+RNMERGGR+VTT+ NDARVVLQMPRGNLEVI

Sbjct 601 FCLAGNYLAYTKLTELNFVLLSTRQNVYKRNMERGGRLVTTIVNDARVVLQMPRGNLEVI 660

Query 661 SPRVLALEIIGKQLDQKRYSEAFNILRKQRINLNILCDHNMISFVQNVTIFLSQITNPNW 720

SPRVLALEIIGK LDQ+RY +AF++LRKQRINLNI+CDHN++ FV NV IFLS+ITNPNW

Sbjct 661 SPRVLALEIIGKLLDQQRYHDAFDMLRKQRINLNIICDHNLVRFVNNVHIFLSEITNPNW 720

Query 721 LNLFLTDLQNEDFSKTMYASNYTAAHQAYPDGFKIESKVAYLCALLCARMAEINKERFRL 780

LNLFLTDLQNEDFSKTMYA NYTAA QAYP+ FKIESKV YLC LLC M + ERFR

Sbjct 721 LNLFLTDLQNEDFSKTMYAGNYTAAEQAYPEDFKIESKVVYLCELLCTHMTKSGHERFRS 780

Query 781 PIITAYVKTGKLEQALQLIWQVKKEQIELAKQSEGPVQEAAEEALKYLLYLVDVNELYNV 840

PIITAYVK GKLEQALQLIW VKK+Q E+AK+S+ PVQEAAEEALKYLLYLVDVNELYNV

Sbjct 781 PIITAYVKLGKLEQALQLIWHVKKQQNEIAKKSDEPVQEAAEEALKYLLYLVDVNELYNV 840

Query 841 ALGTYDFGLVLFVAQKSQKDPKEFLPFLNELKQLEVNYRRFKIDEHLKRYEKALENISKC 900

ALGTYDFGLVLFVAQKSQKDPKEFLPFLNELKQLEVNYRRFKIDEHLKR+ KALENI KC

Sbjct 841 ALGTYDFGLVLFVAQKSQKDPKEFLPFLNELKQLEVNYRRFKIDEHLKRHVKALENIIKC 900

Query 901 GVEKFSIALEFIQLHELYKLALKFYKIDVEENEAKEGQHSLRECQRQICLAFADYLRAKN 960

G EKF +ALEFI+ H LY+ AL+FYK+ EEN+++E +SL ECQRQICLAFAD+LRAKN

Sbjct 901 GAEKFDVALEFIKRHGLYRQALQFYKLVKEENKSEEQLNSLGECQRQICLAFADHLRAKN 960

Query 961 ELESASIMYERGGNLTQALLSAKNVLDWRRVLMLAQKDGKDIATAALSMVSALQEQGRYD 1020

EL+SASIMYERGGNL QA+LSAK+VLDWRRVLMLAQKDG DIAT ALSMV ALQEQGRYD

Sbjct 961 ELDSASIMYERGGNLAQAMLSAKHVLDWRRVLMLAQKDGIDIATLALSMVPALQEQGRYD 1020

Query 1021 IAHQLLKTYGTNFKEALQCLLKGNLYLAAILEVRMHCQQIDLLDEVVKPDLIAYSKQLAQ 1080

IAH+L KTYGTNFKEALQCLLKGNLYL AILE R++ ++ +LLDE+V+P+L+AY+ QL Q

Sbjct 1021 IAHKLHKTYGTNFKEALQCLLKGNLYLEAILEARLNIEEANLLDELVRPELLAYTVQLQQ 1080

Query 1081 QLADDEKLFVEHKQRLHDVRVLAQKKRDGLVNCYDQPDIDEADLLSDTTSLRSSRYTGSS 1140

QLADDEKLFVEHKQRL VRVLAQ+KRDGL+N YDQPDIDEADLLSDTTSLRSSRYTGSS

Sbjct 1081 QLADDEKLFVEHKQRLQVVRVLAQQKRDGLLNGYDQPDIDEADLLSDTTSLRSSRYTGSS 1140

Query 1141 QGTGKTFRSSKNRRKHERKLLSLKPGNPFEDIALIDALYNLVMKLANQQQQMRDTCKALI 1200

QGTGKTFRSSKNRRKHERKLLSLKPGNPFEDIALIDALYN VMKLANQQQQ+RDTCKAL+

Sbjct 1141 QGTGKTFRSSKNRRKHERKLLSLKPGNPFEDIALIDALYNQVMKLANQQQQVRDTCKALL 1200

Query 1201 ELQLDEVAGALQTQYGHLLTLMQDSFDAIWTDEMVNTQAMQYKPTPYTDYTQLQNEQRYA 1260

ELQ DEVA ALQTQYGHLL L+QDSFDAIWTDE++N QAMQYKPTPYTDYTQLQNEQR+A

Sbjct 1201 ELQRDEVAAALQTQYGHLLALLQDSFDAIWTDELLNAQAMQYKPTPYTDYTQLQNEQRFA 1260

Query 1261 VLAPQKRFKPQINLIDWKCKILS 1283

VLAPQKRFKPQI LIDWKC +L+

Sbjct 1261 VLAPQKRFKPQITLIDWKCDVLA 1283

**GLD-1**

**>lcl|ORF2**

MSLCDVSAITIQQTPQQQHQSTQSIADYLAQLLKDRKQMAAFPNVFNHVE

RLLDEEIARVRASLFQINGVKKEPLTLPEPEGTPVQLNEKVYVPVREHPD

FNFVGRILGPRGMTAKQLEQETGCKIMVRGKGSMRDKKKEDANRGKPNWE

HLSDDLHVLITVEDTENRAKVKLAQAVAEVQKLLVPQAEGEDELKKRQLM

ELAIINGTYRDTSAKNPVPACEEEWRRVIAASAENRLLAPALPGLAQQIR

APQATTLGAPLILSQRMTVPTTAASILSGQAAPTAFENPAHGMIFAPYGD

YANYAALAAGNPLLAEYTDHSVGAIKQQRRLANRDHPYQRAAAAVGVTAK

PGFIEIQ

**Graphical representation - ORF**


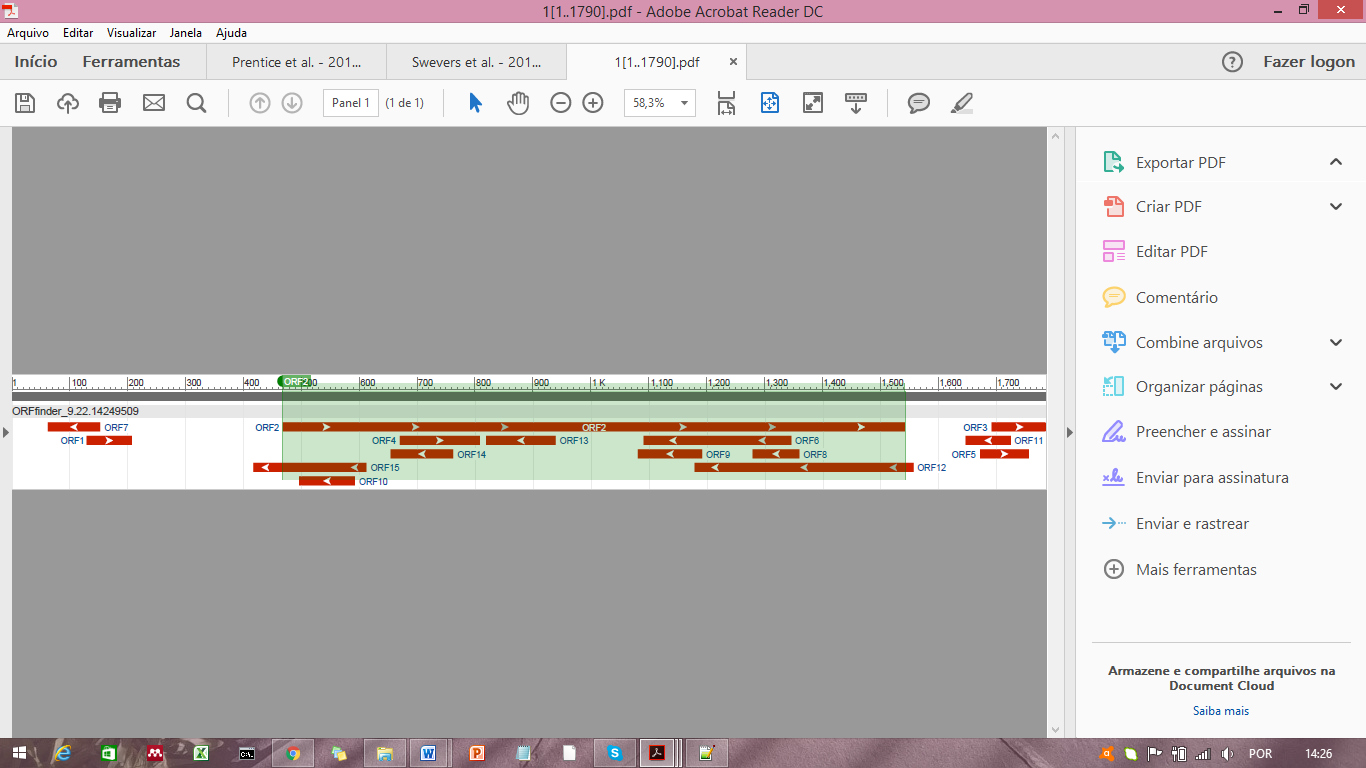


**BlastP (Non-redundant NCBI sequences)**

PREDICTED: protein held out wings isoform X2 [Bactrocera dorsalis]

Sequence ID: [XP_011202829.1](https://www.ncbi.nlm.nih.gov/protein/751786859?report=genbank&log$=protalign&blast_rank=1&RID=WAJ0ZJAT014) Length: 357 Number of Matches: 1

Range 1: 1 to 357

Score: 714 bits(1843)

E-value: 0.0

Identities: 345/357(97%)

Query 1 MSLCDVSAITIQQTPQQQHQSTQSIADYLAQLLKDRKQMAAFPNVFNHVERLLDEEIARV 60

MSLCD SA+TIQ TPQQQHQSTQSIADYLAQLLKDRKQMAAFPNVFNHVERLLDEEIARV

Sbjct 1 MSLCDASAVTIQATPQQQHQSTQSIADYLAQLLKDRKQMAAFPNVFNHVERLLDEEIARV 60

Query 61 RASLFQINGVKKEPLTLPEPEGTPVQLNEKVYVPVREHPDFNFVGRILGPRGMTAKQLEQ 120

RASLFQINGVKKEPLTLPEPEGTPVQLNEKVYVPVREHPDFNFVGRILGPRGMTAKQLEQ

Sbjct 61 RASLFQINGVKKEPLTLPEPEGTPVQLNEKVYVPVREHPDFNFVGRILGPRGMTAKQLEQ 120

Query 121 ETGCKIMVRGKGSMRDKKKEDANRGKPNWEHLSDDLHVLITVEDTENRAKVKLAQAVAEV 180

ETGCKIMVRGKGSMRDKKKEDANRGKPNWEHLSDDLHVLITVEDT+NRAKVKLAQAVAEV

Sbjct 121 ETGCKIMVRGKGSMRDKKKEDANRGKPNWEHLSDDLHVLITVEDTQNRAKVKLAQAVAEV 180

Query 181 QKLLVPQAEGEDELKKRQLMELAIINGTYRDTSAKNPVPACEEEWRRVIAASAENRLLAP 240

QKLLVPQAEGEDELKKRQLMELAIINGTYRDTSAKN +PACEEEWRRVIAASAE+RLLAP

Sbjct 181 QKLLVPQAEGEDELKKRQLMELAIINGTYRDTSAKNAIPACEEEWRRVIAASAESRLLAP 240

Query 241 ALPGLAQQIRAPQATTLGAPLILSQRMTVPTTAASILSGQAAPTAFENPAHGMIFAPYGD 300

ALP LA QIRAPQATTLGAPLILSQRMTVPTTAASILSGQAAP AFENPAHGMIFAPYGD

Sbjct 241 ALPTLAPQIRAPQATTLGAPLILSQRMTVPTTAASILSGQAAPAAFENPAHGMIFAPYGD 300

Query 301 YANYAALAAGNPLLAEYTDHSVGAIKQQRRLANRDHPYQRAAAAVGVTAKPGFIEIQ 357

YANYAALAAGNPLL EYTDHSVGA+KQQRRLANRDHPYQRAAAAVGVTAKPGFIEIQ

Sbjct 301 YANYAALAAGNPLLTEYTDHSVGALKQQRRLANRDHPYQRAAAAVGVTAKPGFIEIQ 357

**ACO**

**>lcl|ORF8**

MLKTKANDDKVDTLLTKGLVPIIDLAHCGTEEAPVRSVVNRVGHQLQKCL

SEKGLCLLVNHGISDEKLKTAWDHLDDFVDLPVDVKETYIRTGDDNHGYV

RPAMERFDGKTPELRHAFNICTLNTKNLPEEPLPGFSEHISSLAQDFKAL

SRFILQALAVSLDIPQSFFLEKHSHMLSGDHDNESTLRLLYYPPIIEDKD

ENNDFIKGSCIYSYQRCLSDKPDFRPETNPRDEINEVDGNDESKDTTERQ

FPNGEIVRCGAHTDYGTFTLLAQDSEGGLEVKLPGTVKWQRVGHLPGAIL

INCGEILSIWTKARYPALQHRVVVPEQPHIRTRGRHSIAFFCHPDNLTMI

SPNDLPNNEVGTDTVDKKPRKKSFKAAKEKVYNAYQLIQKRFRETYGHQN

SH

**Graphical representation –** **ORF**


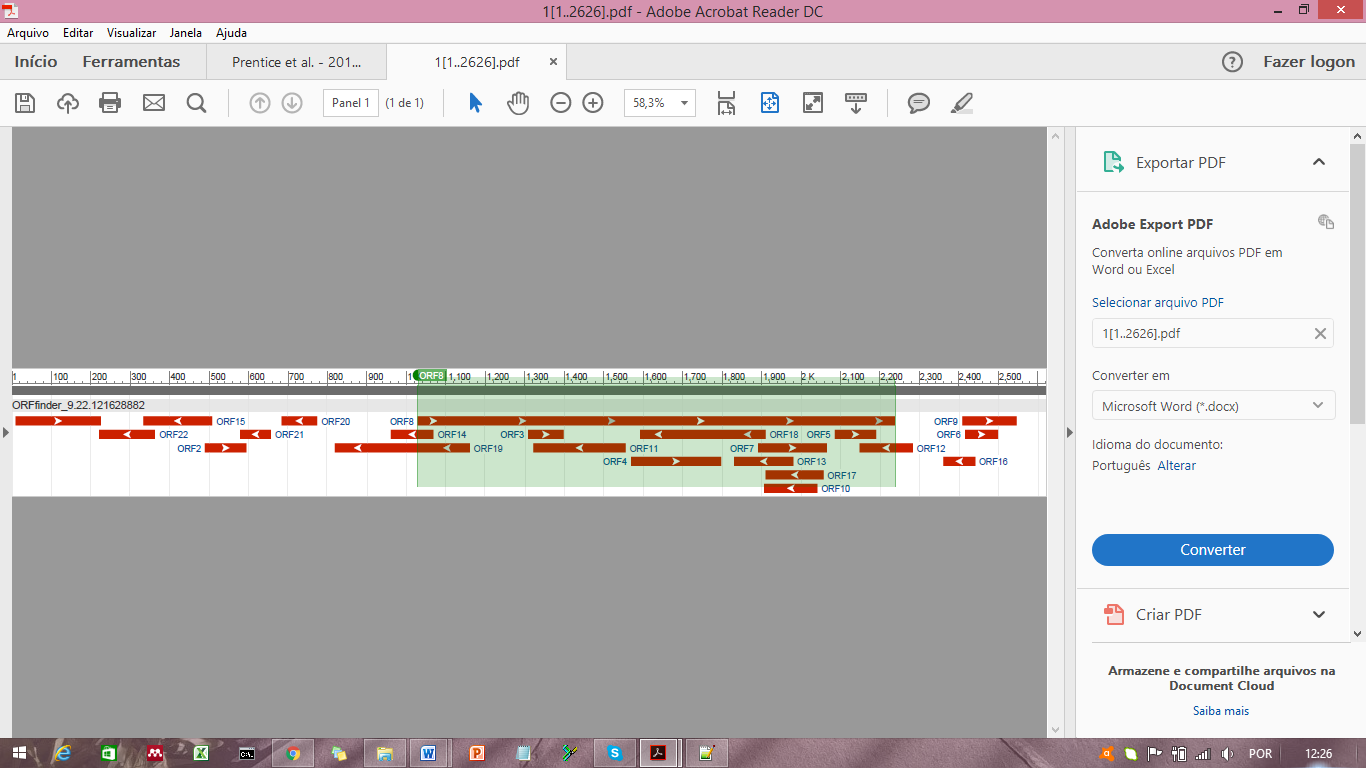


**BlastP (Non-redundant NCBI sequences)**

PREDICTED: 1-aminocyclopropane-1-carboxylate oxidase-like isoform X1 [Rhagoletis zephyria]

Sequence ID: [XP_017483038. 1](https://www.ncbi.nlm.nih.gov/protein/1048059244?report=genbank&log$=protalign&blast_rank=1&RID=WAAD4476015)Length: 402 Number of Matches: 1

Range 1: 1 to 402

Score: 793 bits(2047)

E-value: 0.0

Identities: 374/402(93%)

Query 1 MLKTKANDDKVDTLLTKGLVPIIDLAHCGTEEAPVRSVVNRVGHQLQKCLSEKGLCLLVN 60

MLK K NDDKVDTLLTKGLVPIIDLAHCGTEEAPVRSVVNRVGHQLQKC+SEKGLCLLVN

Sbjct 1 MLKAKTNDDKVDTLLTKGLVPIIDLAHCGTEEAPVRSVVNRVGHQLQKCMSEKGLCLLVN 60

Query 61 HGISDEKLKTAWDHLDDFVDLPVDVKETYIRTGDDNHGYVRPAMERFDGKTPELRHAFNI 120

HGISDEKLKTAWDHLDDFVDLP DVKE Y+RTGDDNHGYVRP +ERF+GKTPELRHAFNI

Sbjct 61 HGISDEKLKTAWDHLDDFVDLPGDVKEHYLRTGDDNHGYVRPGVERFNGKTPELRHAFNI 120

Query 121 CTLNTKNLPEEPLPGFSEHISSLAQDFKALSRFILQALAVSLDIPQSFFLEKHSHMLSGD 180

CTLN KNLPEEPLPGF+EHISSLA+DFKALSRFILQ+LAVSLDIPQSFFLEKHSHMLSGD

Sbjct 121 CTLNAKNLPEEPLPGFAEHISSLARDFKALSRFILQSLAVSLDIPQSFFLEKHSHMLSGD 180

Query 181 HDNESTLRLLYYPPIIEDKDENNDFIKGSCIYSYQRCLSDKPDFRPETNPRDEINEVDGN 240

HDNESTLRLLYYPPIIED DE NDFIKGSCIYSYQRCLSDKPDFRPETNPRDEINE+DG

Sbjct 181 HDNESTLRLLYYPPIIEDTDEKNDFIKGSCIYSYQRCLSDKPDFRPETNPRDEINELDGK 240

Query 241 DESKDTTERQFPNGEIVRCGAHTDYGTFTLLAQDSEGGLEVKLPGTVKWQRVGHLPGAIL 300

DESKDTTERQ PNGE++RCGAHTDYGTFTLLAQDSEGGLEVK+PG+ KWQRVGHLPGAIL

Sbjct 241 DESKDTTERQLPNGEMIRCGAHTDYGTFTLLAQDSEGGLEVKMPGSEKWQRVGHLPGAIL 300

Query 301 INCGEILSIWTKARYPALQHRVVVPEQPHIRTRGRHSIAFFCHPDNLTMISPNDLPNNEV 360

INCGEILSIWTK RYPALQHRVV+PEQPHIRTRGRHSIAFFCHPDNLTMISPNDLPN EV

Sbjct 301 INCGEILSIWTKGRYPALQHRVVIPEQPHIRTRGRHSIAFFCHPDNLTMISPNDLPNTEV 360

Query 361 GTDTVDKKPRKKSFKAAKEKVYNAYQLIQKRFRETYGHQNSH 402

GTDTVDKKPRKKSF+AAKEKVYNAYQLIQKRFRETYGHQ SH

Sbjct 361 GTDTVDKKPRKKSFRAAKEKVYNAYQLIQKRFRETYGHQASH 402

**Scavenger receptor**

**>lcl|ORF13**

MPKKADSLKKWQQNNRKVIVGIVGFCLSIFGVLCGMFWEQIFNGIMEKEM

ILRPNSEVYDKWKSPPMTLSLDIYLYNWTNPEDFKNLSTKPNFEQLGPYR

FTEKQDKVNIQWNPANASVTYRKKSDFYFDAKGSNGSLDDVIVTLNAVAL

SAAGKAKRWNPVRRNLVDVGLKLYGQEISITRTIDEMLFTGYSDDMLDMA

RAMPLFGKDVEVPFDKFGWFYTRNGSTDLTGVFNVYTGADDISKIGQMHT

WNYKHHTGYFQSTCGLVNGSAGEFHPPYLKQYGSVSFFTPDLCRTVPLDY

LETAEIEGLLGYKYHGGVRSVDNGTLYPENTCFCGGQCVPSGVMNISSCR

FGSPVFMSFPHFYNADPYYVQQVDGMQPEQDKHEFYMILEPRTGVALEVA

ARFQVNMLVEPIKGITLYENVPRVFFPLIWFEQKVRITPDLAADLQLLPK

VLMGGQIFACICFGVGLILLCWYPIELLWTRQRSVDLKCTPTTVENGKQL

AKFSSVEELKNRPPPVVTTNNNKTLDSSPLLERGNGKSATIVSKSETNES

VATDKTAVSNVAKE

**Graphical representation – ORF**


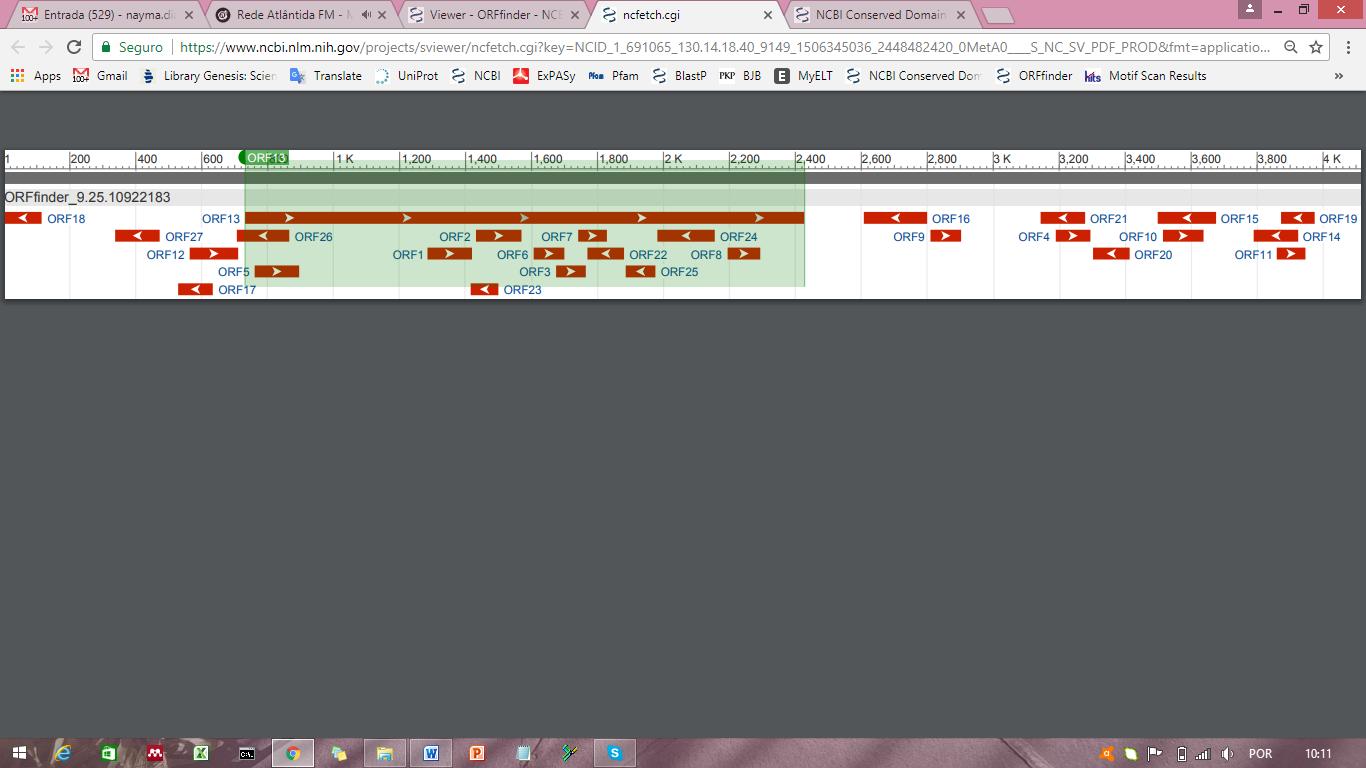


**BlastP (Non-redundant NCBI sequences)**

PREDICTED: protein croquemort [Rhagoletis zephyria]

Sequence ID: [XP_017482227.1](https://www.ncbi.nlm.nih.gov/protein/1048056170?report=genbank&log$=protalign&blast_rank=1&RID=WJ15NRX6015)Length: 570Number of Matches: 1

Range 1: 1 to 564

Score: 1025 bits(2651)

E-value: 0.0

Identities: 480/564(85%)

Query 1 MPKKADSLKKWQQNNRKVIVGIVGFCLSIFGVLCGMFWEQIFNGIMEKEMILRPNSEVYD 60

MPKK +SL KWQ +NRKVI+ I GFCL IFGVLCGMFWEQIFN I+EKEMILRP+S+VYD

Sbjct 1 MPKKGNSLTKWQHSNRKVIIAIAGFCLGIFGVLCGMFWEQIFNSIVEKEMILRPDSQVYD 60

Query 61 KWKSPPMTLSLDIYLYNWTNPEDFKNLSTKPNFEQLGPYRFTEKQDKVNIQWNPANASVT 120

KWK+PPM LSLDIYLYNWTNPEDFKNLS KP+ EQLGPYRFTEKQDKV+I WNP NASVT

Sbjct 61 KWKTPPMALSLDIYLYNWTNPEDFKNLSAKPSVEQLGPYRFTEKQDKVDIHWNPENASVT 120

Query 121 YRKKSDFYFDAKGSNGSLDDVIVTLNAVALSAAGKAKRWNPVRRNLVDVGLKLYGQEISI 180

YRKKSDFYFDA+GSNGSLDDVIVTLNAVALSAAGKAKRWN VRRNLVDVGLKLYGQE+SI

Sbjct 121 YRKKSDFYFDAEGSNGSLDDVIVTLNAVALSAAGKAKRWNSVRRNLVDVGLKLYGQEMSI 180

Query 181 TRTIDEMLFTGYSDDMLDMARAMPLFGKDVEVPFDKFGWFYTRNGSTDLTGVFNVYTGAD 240

RT+DEMLFTGYSDDML+MARAMPLFGKDVEVPFDKFGWFYTRNGSTDLTGVFNVYTGAD

Sbjct 181 ARTVDEMLFTGYSDDMLEMARAMPLFGKDVEVPFDKFGWFYTRNGSTDLTGVFNVYTGAD 240

Query 241 DISKIGQMHTWNYKHHTGYFQSTCGLVNGSAGEFHPPYLKQYGSVSFFTPDLCRTVPLDY 300

DISKIGQMHTWNYK HTG+FQSTCG +NGSAGEF+PPYLK+ G V+ +TPD+CRTVPLDY

Sbjct 241 DISKIGQMHTWNYKQHTGFFQSTCGHINGSAGEFYPPYLKENGGVALYTPDMCRTVPLDY 300

Query 301 LETAEIEGLLGYKYHGGVRSVDNGTLYPENTCFCGGQCVPSGVMNISSCRFGSPVFMSFP 360

+ T E+EGL G+KY GG RSVDNGTLYPEN+CFCGG+CVPSGVMNISSCRFGSPVFMS+P

Sbjct 301 VGTTEVEGLRGFKYSGGARSVDNGTLYPENSCFCGGECVPSGVMNISSCRFGSPVFMSYP 360

Query 361 HFYNADPYYVQQVDGMQPEQDKHEFYMILEPRTGVALEVAARFQVNMLVEPIKGITLYEN 420

HFYNADPYYVQQVDGMQPEQDKHEFYMILEPRTGVALEVAARFQVNMLVEPI+G++LYE+

Sbjct 361 HFYNADPYYVQQVDGMQPEQDKHEFYMILEPRTGVALEVAARFQVNMLVEPIQGVSLYED 420

Query 421 VPRVFFPLIWFEQKVRITPDLAADLQLLPKVLMGGQIFACICFGVGLILLCWYPIELLWT 480

PRVFFPLIWFEQKVRITPDLAADLQ+LP +LM GQIFAC+CF VGLILLCWYPIE+LW

Sbjct 421 APRVFFPLIWFEQKVRITPDLAADLQMLPIILMAGQIFACLCFAVGLILLCWYPIEMLWA 480

Query 481 RQRSVDLKCTP-TTVENGKQLAKFSSVEELKNR-PPPVVTTNNNKTLDSSPLLERGNGKS 538

RQRSVDLK P TTVENGKQ +KFSS EELK R PPP V NNNK LDSSPLLERGNGK+

Sbjct 481 RQRSVDLKTMPTTTVENGKQTSKFSSGEELKTRPPPPAVMANNNKNLDSSPLLERGNGKT 540

Query 539 ATIVSKSETNESVATDKTAVSNVA 562

I+ KSETNESVATD T VS+ A

Sbjct 541 VIIMPKSETNESVATDNTVVSDDA 564

**Eater**

**>lcl|ORF24**

MKIHRSCQFYGSLLLHVVIVSACVVKVPQIKTRVSLVTMRKFPVDANCTS

NCAPNYNPLVLRTVKESYTDTDEICCDGYVRDNKSGDCQPKCADCKTGKC

VRPDVCLCDAGYNNQKNSTICEPECSEPCINGQCIAPDTCTCNEGFSFIN

SSITECETKCLIDCTNGRCDSPEKCTCNFGYERNETLGLCIPVCEEPCEN

GICKAPNECDCHSGFELRLGTLGKCEPVCQGGCSNGYCSAPNVCKCQLGY

YNLLGLACVPSCKEKCVNAHCTAPNQCTCLEGHIYRNNSRTECEPTCARG

CQNGFCNEPGRCECHQGYKQVEAHVCQPICKDECINGHCSAPDTCTCNEG

YIFKNGSRAECEPHCPRGCKNGNCVSPGVCSCLPGYQSLLFYLCIPVCTH

SCVHGTCTAPDTCRCFSGYRPNTKRPHECEPVCNFDCGHGQCIAPGVCQC

EEGYAKKWLTGRCEPHCAQKCVNSICVAGGICRCYEGFRLRKGSNAICDP

ICLPACINSNCVEPDMCECWSGYEETRHRSLCIAHCRPSCENGMCVAPNK

CQCSDGYRVMNSSEPHRCRAICRDTCINAECMRPDECVCLEGYDFLNGSR

TECAPICDQNCGHGRCIGPNACSCDLGYRLTLSNDTDLPVCVAYCNEWNC

LGGKCDVNGICQCVQGKIYNERRGACVSELGSIEDHLVLSGVSVSRWTIG

AMSLLLIALCALALVLVYREYARRRFREKHGVRLIENPTFGVVMPGAGAE

DGLNMQEEDES

**Graphical representation – ORF**


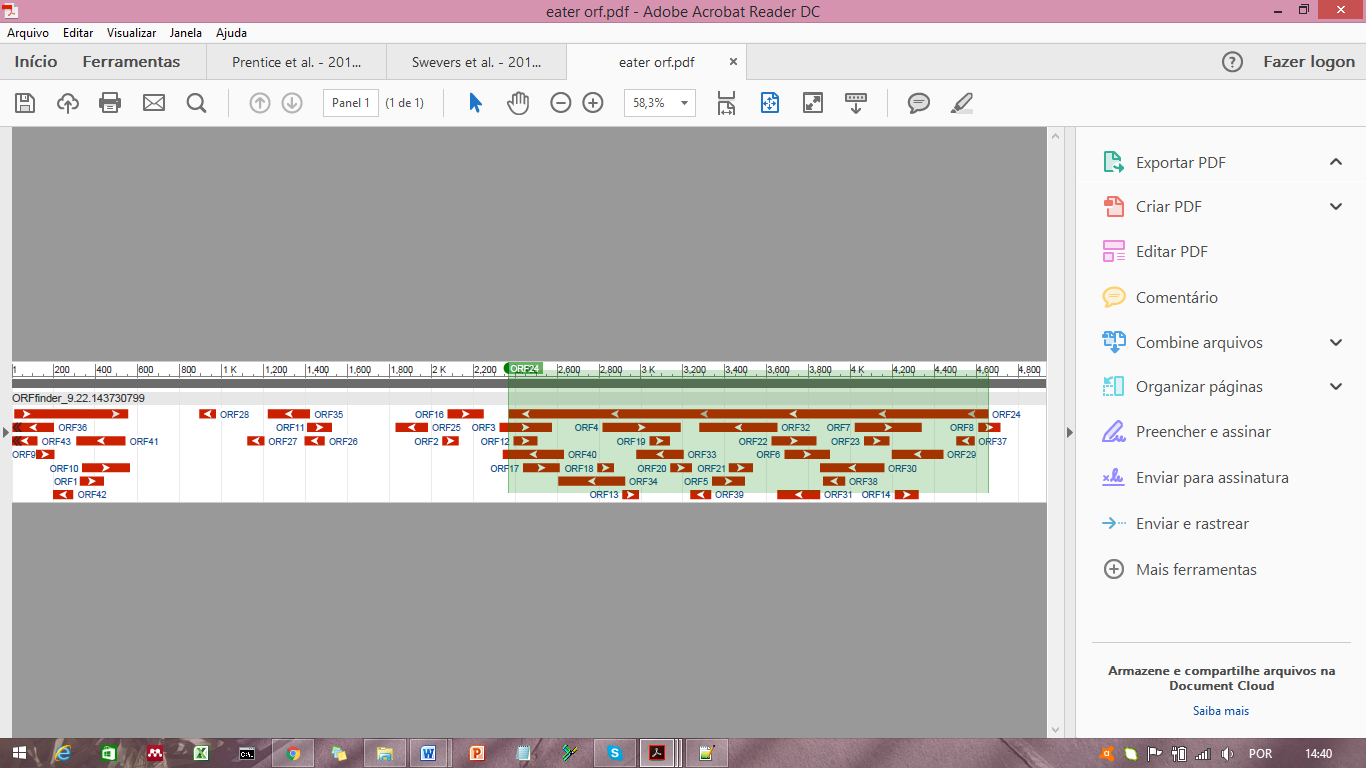


**BlastP (Non-redundant NCBI sequences)**

PREDICTED: multiple epidermal growth factor-like domains protein 10 [Rhagoletis zephyria]

Sequence ID: [XP_017465105.1](https://www.ncbi.nlm.nih.gov/protein/1048008683?report=genbank&log$=protalign&blast_rank=1&RID=WAJN104J014) Length: 761 Number of Matches: 1

Range 1: 1 to 761

Score: 1327 bits(3435)

E-value: 0.0

Identities: 651/761(86%)

Query 1 MKIHRSCQFYGSLLLHVVIVSACVVKVPQIKTRVSLVTMRKFPVDANCTSNCAPNYNPLV 60

MK+ + F L+LH + S+CVVKVP +KTRV+LVTMRKF VD NCT NC P++NPLV

Sbjct 1 MKLQKYSLFGVILMLHAALASSCVVKVPTMKTRVALVTMRKFQVDENCTKNCGPSHNPLV 60

Query 61 LRTVKESYTDTDEICCDGYVRDNKSGDCQPKCADCKTGKCVRPDVCLCDAGYNNQKNSTI 120

LRTVKESYTDT+E+CCDGYVRD K+GDCQP+CADCKTGKCVRPDVCLCD G+NN KNSTI

Sbjct 61 LRTVKESYTDTEEVCCDGYVRDTKNGDCQPRCADCKTGKCVRPDVCLCDEGFNNMKNSTI 120

Query 121 CEPECSEPCINGQCIAPDTCTCNEGFSFINSSITECETKCLIDCTNGRCDSPEKCTCNFG 180

CEPECSEPCINGQCIAPD C C++GFSF+N S+T+CETKCL+DCTNGRCDSP+KCTCNFG

Sbjct 121 CEPECSEPCINGQCIAPDLCACHDGFSFVNGSVTDCETKCLVDCTNGRCDSPQKCTCNFG 180

Query 181 YERNETLGLCIPVCEEPCENGICKAPNECDCHSGFELRLGTLGKCEPVCQGGCSNGYCSA 240

YERNETLGLC+P+CEEPCENGICKAPNEC CH+GFELRLGTLGKCEPVCQGGC NGYC+A

Sbjct 181 YERNETLGLCVPICEEPCENGICKAPNECSCHNGFELRLGTLGKCEPVCQGGCPNGYCNA 240

Query 241 PNVCKCQLGYYNLLGLACVPSCKEKCVNAHCTAPNQCTCLEGHIYRNNSRTECEPTCARG 300

PNVCKCQ GYYNLLGLACVPSCKEKCVNAHCTAPNQCTCLEGHIYRNNSR+ECEPTC RG

Sbjct 241 PNVCKCQEGYYNLLGLACVPSCKEKCVNAHCTAPNQCTCLEGHIYRNNSRSECEPTCVRG 300

Query 301 CQNGFCNEPGRCECHQGYKQVEAHVCQPICKDECINGHCSAPDTCTCNEGYIFKNGSRAE 360

CQNGFCNEPGRCECHQGY+QVEAH+CQPICKDEC+NGHCSAPDTC+CNEGYIFKNGS E

Sbjct 301 CQNGFCNEPGRCECHQGYRQVEAHLCQPICKDECVNGHCSAPDTCSCNEGYIFKNGSSTE 360

Query 361 CEPHCPRGCKNGNCVSPGVCSCLPGYQSLLFYLCIPVCTHSCVHGTCTAPDTCRCFSGYR 420

CEPHCPRGCKNGNCVSPGVCSCLPG+QSLLFYLCIPVCT SCVHGTCTAPDTCRCFSGYR

Sbjct 361 CEPHCPRGCKNGNCVSPGVCSCLPGFQSLLFYLCIPVCTQSCVHGTCTAPDTCRCFSGYR 420

Query 421 PNTKRPHECEPVCNFDCGHGQCIAPGVCQCEEGYAKKWLTGRCEPHCAQKCVNSICVAGG 480

PN R +EC+PVCNFDCGHG CIAPG+CQC EGY+KKWLTGRCEPHC QKC+NS+C AGG

Sbjct 421 PNALRSNECDPVCNFDCGHGHCIAPGICQCAEGYSKKWLTGRCEPHCTQKCINSVCAAGG 480

Query 481 ICRCYEGFRLRKGSNAICDPICLPACINSNCVEPDMCECWSGYEETRHRSLCIAHCRPSC 540

ICRCYEGFRLRKGSNAICDPICLP+CINSNCVEPDMCECWSGYEETRHR+LCIAHCRPSC

Sbjct 481 ICRCYEGFRLRKGSNAICDPICLPSCINSNCVEPDMCECWSGYEETRHRNLCIAHCRPSC 540

Query 541 ENGMCVAPNKCQCSDGYRVMNSSEPHRCRAICRDTCINAECMRPDECVCLEGYDFLNGSR 600

ENG CVAPNKCQC+DGYRV NSSEPHRC+AIC+DTC+NAEC+RPDECVCLEGY FLNGSR

Sbjct 541 ENGKCVAPNKCQCADGYRVTNSSEPHRCQAICKDTCVNAECLRPDECVCLEGYTFLNGSR 600

Query 601 TECAPICDQNCGHGRCIGPNACSCDLGYRLTLSNDTDLPVCVAYCNEWNCLGGKCDVNGI 660

TECAP C+Q+CGHGRCI PNACSCDLGYRL LS D DLPVCVAYCNEWNCLGGKCDVNGI

Sbjct 601 TECAPRCEQDCGHGRCIAPNACSCDLGYRLKLSADNDLPVCVAYCNEWNCLGGKCDVNGI 660

Query 661 CQCVQGKIYNERRGACVSELGSIEDHLVLSGVSVSRWTIGAMSLLLIALCALALVLVYRE 720

CQC +G +YNERRGACVS+L ++E L SGVSVSRWTIGA+SLLLIALCALA +LVYRE

Sbjct 661 CQCGEGTVYNERRGACVSKLDAMEGRLKFSGVSVSRWTIGAVSLLLIALCALAAILVYRE 720

Query 721 YARRRFREKHGVRLIENPTFGVVMPGAGAEDGLNMQEEDES 761

YARRRFREKHGVRLIENPTFG+V+PGAG EDGLN+QEE+E+

Sbjct 721 YARRRFREKHGVRLIENPTFGIVIPGAGEEDGLNVQEEEET 761

Clathrin Heavy chain (chc)

**>lcl|ORF1**

MTQVLPIRFQEHLQLTNVGINLNSISFSTLTMESDKFICVREKVNDTAQV

VIIDMNDVSNPTRRPISADSAIMNPASKVIALKAQKTLQIFNIEMKSKMK

AHTMTEDVVFWKWISLNTLALVTETSVYHWSMEGDSTPQKMFDRHSSLNG

CQIINYRCNPSQQWLLLVGISALPNRVAGAMQLYSVERKVSQAIEGHAAS

FASFKMEGNKEPSTLFCFAVRTASGGKLHIIEVGAPPAGNQPFPKKSVDV

FFPPEAQSDFPVAMQVSAKYDTIYLITKYGYIHLYDMETATCIYMNRISA

DTIFVTAPHEASGGIIGVNRKGQVLSVTVDEEQIIPYINTVLQNPDLALR

MAVRNNLSGAEDLFVRKFNKLFTAGQYAEAAKVAALAPKGILRTPQTIQR

FQQVQSPAGSTTPPLLQYFGILLDQGKLNKYESLELCRPVLVQGKKQLCE

KWLKEEKLECSEELGDLVKTSDLTLALSIYLRANVPNKVIQCFAETGQFQ

KIVLYAKKVNYTPDYIFLLRSVMRTNPEQGAGFASMLVAEEEEPLADINQ

IVDIFMEHSMVQQCTAFLLDALKHNRPNEGALQTRLLEMNLISAPQVADA

ILGNAMFTHYDRAHIAQLCEKAGLLQRALEHYTDLYDIKRAVVHTHLLNA

DWLVSYFGTLSVEDSLECLKAMLTANIRQNLQICVQIATKYHEQLTTKAL

IDLFESFKSYEGLFYFLGSIVNYSQDPEVHFKYIQAACKTNQIKEVERIC

RESNCYNAERVKNFLKEAKLTDQLPLIIVCDRFDFVHDLVLYLYRNNLQK

YIEIYVQKVNPSRLPVVVGGLLDVDCSEDIIKNLILVVKGQFSTDELVAE

VEKRNRLKLLLPWLESRVHDGCVEPATHNALAKIYIDSNNNPERFLKENQ

YYDSRVVGRYCEKRDPHLACVAYERGQCDRELIAVCNENSLFKSEARYLV

RRRDPALWVEVLSESNPYKRQLIDQVVQTALSETQDPDDISVTVKAFMTA

DLPNELIELLEKIILDSSVFSDHRNLQNLLILTAIKADRTRVMDYINRLD

NYDAPDIANIAISNQLYEEAFAIFKKFDVNTSAIQVLIEQVNNLERANEF

AERCNEPAVWSQLAKAQLQQGLVKEAIDSYIKADDPSAYMDVVDVASKAD

SWDDLVRYLQMARKKARESYIESELIYAYARTGRLADLEEFISGPNHADI

QKIGDRCFNDGMYDAAKLLYNNVSNFARLAITLVYLKEFQGAVDSARKAN

STRTWKEVCFACVDAEEFRLAQMCGLHIVVHADELEDLINYYQDRGYFEE

LIALLESALGLERAHMGMFTELAILYSKFKPSKMREHLELFWSRVNIPKV

LRAAESAHLWSELVFLYDKYEEYDNAVLAMMAHPTEAWREGHFKDIITKV

ANIELYYKAIQFYLDYKPLLLNDMLLVLAPRMDHTRAVSFFSKTGHLQLV

KPYLRSVQSLNNKAINEALNGLLIEEEDYQGLRNSIDGFDNFDTIALAQK

LEKHELTEFRRIAAYLYKGNNRWKQSVELCKKDKLYKDAMEYAAESGKQE

IAEELLGWFLERNAHDCFAACLFQCYDLLRPDVILELAWKHNIMDFAMPY

LIQVIREYTSKVDKLEQTEAQREKEDETVEHKNIITMEPQLMITAGPAMG

IPPQYAQNYAAAPGYAPNMAYPGYGM

**Graphical representation – ORF**


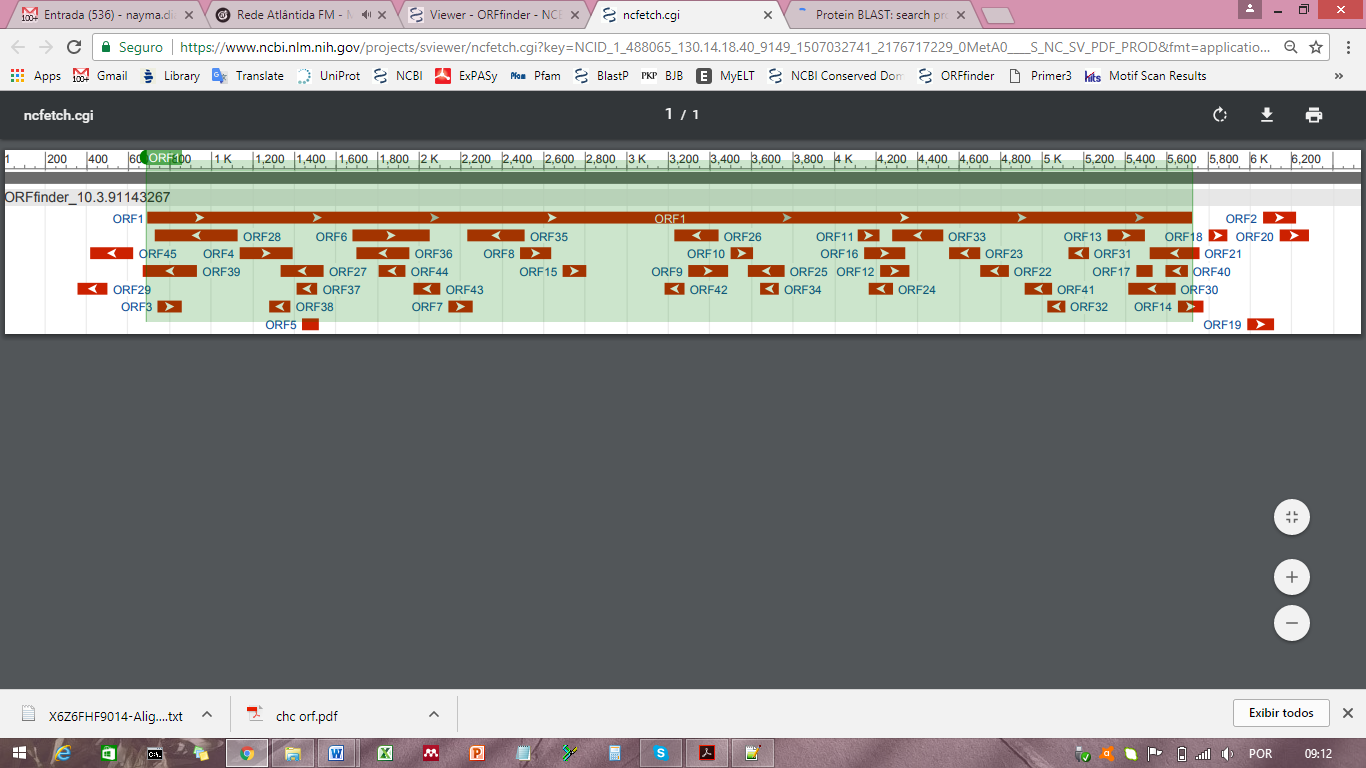


**BlastP (Non-redundant NCBI sequences)**

PREDICTED: clathrin heavy chain isoform X2 [Rhagoletis zephyria]

Sequence ID: [XP_017470846.1](https://www.ncbi.nlm.nih.gov/protein/1048020721?report=genbank&log$=protalign&blast_rank=1&RID=X6ZRD4EY015) Length: 1676 Number of Matches: 1

Range 1: 1 to 1676

Score: 3481 bits(9026)

E-value: 0.0

Identities: 1671/1676(99%)

Query 1 MTQVLPIRFQEHLQLTNVGINLNSISFSTLTMESDKFICVREKVNDTAQVVIIDMNDVSN 60

MTQVLPIRFQEHLQLTNVGINLNSISFSTLTMESDKFICVREKVNDTAQVVIIDMNDVSN

Sbjct 1 MTQVLPIRFQEHLQLTNVGINLNSISFSTLTMESDKFICVREKVNDTAQVVIIDMNDVSN 60

Query 61 PTRRPISADSAIMNPASKVIALKAQKTLQIFNIEMKSKMKAHTMTEDVVFWKWISLNTLA 120

PTRRPISADSAIMNPASKVIALKAQKTLQIFNIEMKSKMKAHTMTEDVVFWKWISLNTLA

Sbjct 61 PTRRPISADSAIMNPASKVIALKAQKTLQIFNIEMKSKMKAHTMTEDVVFWKWISLNTLA 120

Query 121 LVTETSVYHWSMEGDSTPQKMFDRHSSLNGCQIINYRCNPSQQWLLLVGISALPNRVAGA 180

LVTETSVYHWSMEGDSTPQKMFDRHSSLNGCQIINYRCNPSQQWLLLVGISALPNRVAGA

Sbjct 121 LVTETSVYHWSMEGDSTPQKMFDRHSSLNGCQIINYRCNPSQQWLLLVGISALPNRVAGA 180

Query 181 MQLYSVERKVSQAIEGHAASFASFKMEGNKEPSTLFCFAVRTASGGKLHIIEVGAPPAGN 240

MQLYSVERKVSQAIEGHAASFASFKMEGNKEPSTLFCFAVRTASGGKLHIIEVGAPPAGN

Sbjct 181 MQLYSVERKVSQAIEGHAASFASFKMEGNKEPSTLFCFAVRTASGGKLHIIEVGAPPAGN 240

Query 241 QPFPKKSVDVFFPPEAQSDFPVAMQVSAKYDTIYLITKYGYIHLYDMETATCIYMNRISA 300

QPFPKKSVDVFFPPEAQSDFPVAMQVSAKYD IYLITKYGYIHLYDMETATCIYMNRISA

Sbjct 241 QPFPKKSVDVFFPPEAQSDFPVAMQVSAKYDAIYLITKYGYIHLYDMETATCIYMNRISA 300

Query 301 DTIFVTAPHEASGGIIGVNRKGQVLSVTVDEEQIIPYINTVLQNPDLALRMAVRNNLSGA 360

DTIFVTAPHEASGGIIGVNRKGQVLSVTVDEEQIIPYINTVLQNPDLALRMAVRNNLSGA

Sbjct 301 DTIFVTAPHEASGGIIGVNRKGQVLSVTVDEEQIIPYINTVLQNPDLALRMAVRNNLSGA 360

Query 361 EDLFVRKFNKLFTAGQYAEAAKVAALAPKGILRTPQTIQRFQQVQSPAGSTTPPLLQYFG 420

EDLFVRKFNKLFTAGQYAEAAKVAALAPKGILRTPQTIQRFQQVQSPAGSTTPPLLQYFG

Sbjct 361 EDLFVRKFNKLFTAGQYAEAAKVAALAPKGILRTPQTIQRFQQVQSPAGSTTPPLLQYFG 420

Query 421 ILLDQGKLNKYESLELCRPVLVQGKKQLCEKWLKEEKLECSEELGDLVKTSDLTLALSIY 480

ILLDQGKLNKYESLELCRPVLVQGKKQLCEKWLKEEKLECSEELGDLVKTSDLTLALSIY

Sbjct 421 ILLDQGKLNKYESLELCRPVLVQGKKQLCEKWLKEEKLECSEELGDLVKTSDLTLALSIY 480

Query 481 LRANVPNKVIQCFAETGQFQKIVLYAKKVNYTPDYIFLLRSVMRTNPEQGAGFASMLVAE 540

LRANVPNKVIQCFAETGQFQKIVLYAKKVNYTPDYIFLLRSVMRTNPEQGAGFASMLVAE

Sbjct 481 LRANVPNKVIQCFAETGQFQKIVLYAKKVNYTPDYIFLLRSVMRTNPEQGAGFASMLVAE 540

Query 541 EEEPLADINQIVDIFMEHSMVQQCTAFLLDALKHNRPNEGALQTRLLEMNLISAPQVADA 600

EEEPLADINQIVDIFMEHSMVQQCTAFLLDALKHNR NEGALQTRLLEMNLISAPQVADA

Sbjct 541 EEEPLADINQIVDIFMEHSMVQQCTAFLLDALKHNRANEGALQTRLLEMNLISAPQVADA 600

Query 601 ILGNAMFTHYDRAHIAQLCEKAGLLQRALEHYTDLYDIKRAVVHTHLLNADWLVSYFGTL 660

ILGNAMFTHYDRAHIAQLCEKAGLLQRALEHYTDLYDIKRAVVHTHLLNADWLVSYFGTL

Sbjct 601 ILGNAMFTHYDRAHIAQLCEKAGLLQRALEHYTDLYDIKRAVVHTHLLNADWLVSYFGTL 660

Query 661 SVEDSLECLKAMLTANIRQNLQICVQIATKYHEQLTTKALIDLFESFKSYEGLFYFLGSI 720

SVEDSLECLKAMLTANIRQNLQICVQIATKYHEQLTTKALIDLFESFKSYEGLFYFLGSI

Sbjct 661 SVEDSLECLKAMLTANIRQNLQICVQIATKYHEQLTTKALIDLFESFKSYEGLFYFLGSI 720

Query 721 VNYSQDPEVHFKYIQAACKTNQIKEVERICRESNCYNAERVKNFLKEAKLTDQLPLIIVC 780

VNYSQDPEVHFKYIQAACKTNQIKEVERICRESNCYNAERVKNFLKEAKLTDQLPLIIVC

Sbjct 721 VNYSQDPEVHFKYIQAACKTNQIKEVERICRESNCYNAERVKNFLKEAKLTDQLPLIIVC 780

Query 781 DRFDFVHDLVLYLYRNNLQKYIEIYVQKVNPSRLPVVVGGLLDVDCSEDIIKNLILVVKG 840

DRFDFVHDLVLYLYRNNLQKYIEIYVQKVNPSRLPVVVGGLLDVDCSEDIIKNLILVVKG

Sbjct 781 DRFDFVHDLVLYLYRNNLQKYIEIYVQKVNPSRLPVVVGGLLDVDCSEDIIKNLILVVKG 840

Query 841 QFSTDELVAEVEKRNRLKLLLPWLESRVHDGCVEPATHNALAKIYIDSNNNPERFLKENQ 900

QFSTDELVAEVEKRNRLKLLLPWLESRVHDGCVEPATHNALAKIYIDSNNNPERFLKENQ

Sbjct 841 QFSTDELVAEVEKRNRLKLLLPWLESRVHDGCVEPATHNALAKIYIDSNNNPERFLKENQ 900

Query 901 YYDSRVVGRYCEKRDPHLACVAYERGQCDRELIAVCNENSLFKSEARYLVRRRDPALWVE 960

YYDSRVVGRYCEKRDPHLACVAYERGQCDRELIAVCNENSLFKSEARYLVRRRD ALWVE

Sbjct 901 YYDSRVVGRYCEKRDPHLACVAYERGQCDRELIAVCNENSLFKSEARYLVRRRDAALWVE 960

Query 961 VLSESNPYKRQLIDQVVQTALSETQDPDDISVTVKAFMTADLPNELIELLEKIILDSSVF 1020

VLSESNPYKRQLIDQVVQTALSETQDPDDISVTVKAFMTADLPNELIELLEKIILDSSVF

Sbjct 961 VLSESNPYKRQLIDQVVQTALSETQDPDDISVTVKAFMTADLPNELIELLEKIILDSSVF 1020

Query 1021 SDHRNLQNLLILTAIKADRTRVMDYINRLDNYDAPDIANIAISNQLYEEAFAIFKKFDVN 1080

SDHRNLQNLLILTAIKAD TRVMDYINRLDNYDAPDIANIAISNQLYEEAFAIFKKFDVN

Sbjct 1021 SDHRNLQNLLILTAIKADHTRVMDYINRLDNYDAPDIANIAISNQLYEEAFAIFKKFDVN 1080

Query 1081 TSAIQVLIEQVNNLERANEFAERCNEPAVWSQLAKAQLQQGLVKEAIDSYIKADDPSAYM 1140

TSAIQVLIEQVNNLERANEFAERCNEPAVWSQLAKAQLQQGLVKEAIDSYIKADDPSAYM

Sbjct 1081 TSAIQVLIEQVNNLERANEFAERCNEPAVWSQLAKAQLQQGLVKEAIDSYIKADDPSAYM 1140

Query 1141 DVVDVASKADSWDDLVRYLQMARKKARESYIESELIYAYARTGRLADLEEFISGPNHADI 1200

DVVDVASKADSWDDLVRYLQMARKKARESYIESELIYAYARTGRLADLEEFISGPNHADI

Sbjct 1141 DVVDVASKADSWDDLVRYLQMARKKARESYIESELIYAYARTGRLADLEEFISGPNHADI 1200

Query 1201 QKIGDRCFNDGMYDAAKLLYNNVSNFARLAITLVYLKEFQGAVDSARKANSTRTWKEVCF 1260

QKIGDRCFNDGMYDAAKLLYNNVSNFARLAITLVYLKEFQGAVDSARKANSTRTWKEVCF

Sbjct 1201 QKIGDRCFNDGMYDAAKLLYNNVSNFARLAITLVYLKEFQGAVDSARKANSTRTWKEVCF 1260

Query 1261 ACVDAEEFRLAQMCGLHIVVHADELEDLINYYQDRGYFEELIALLESALGLERAHMGMFT 1320

ACVDAEEFRLAQMCGLHIVVHADELEDLINYYQDRGYFEELIALLESALGLERAHMGMFT

Sbjct 1261 ACVDAEEFRLAQMCGLHIVVHADELEDLINYYQDRGYFEELIALLESALGLERAHMGMFT 1320

Query 1321 ELAILYSKFKPSKMREHLELFWSRVNIPKVLRAAESAHLWSELVFLYDKYEEYDNAVLAM 1380

ELAILYSKFKPSKMREHLELFWSRVNIPKVLRAAESAHLWSELVFLYDKYEEYDNAVLAM

Sbjct 1321 ELAILYSKFKPSKMREHLELFWSRVNIPKVLRAAESAHLWSELVFLYDKYEEYDNAVLAM 1380

Query 1381 MAHPTEAWREGHFKDIITKVANIELYYKAIQFYLDYKPLLLNDMLLVLAPRMDHTRAVSF 1440

MAHPTEAWREGHFKDIITKVANIELYYKAIQFYLDYKPLLLNDMLLVLAPRMDHTRAVSF

Sbjct 1381 MAHPTEAWREGHFKDIITKVANIELYYKAIQFYLDYKPLLLNDMLLVLAPRMDHTRAVSF 1440

Query 1441 FSKTGHLQLVKPYLRSVQSLNNKAINEALNGLLIEEEDYQGLRNSIDGFDNFDTIALAQK 1500

FSKTGHLQLVKPYLRSVQSLNNKAINEALNGLLIEEEDYQGLRNSIDGFDNFDTIALAQK

Sbjct 1441 FSKTGHLQLVKPYLRSVQSLNNKAINEALNGLLIEEEDYQGLRNSIDGFDNFDTIALAQK 1500

Query 1501 LEKHELTEFRRIAAYLYKGNNRWKQSVELCKKDKLYKDAMEYAAESGKQEIAEELLGWFL 1560

LEKHELTEFRRIAAYLYKGNNRWKQSVELCKKDKLYKDAMEYAAESGKQEIAEELLGWFL

Sbjct 1501 LEKHELTEFRRIAAYLYKGNNRWKQSVELCKKDKLYKDAMEYAAESGKQEIAEELLGWFL 1560

Query 1561 ERNAHDCFAACLFQCYDLLRPDVILELAWKHNIMDFAMPYLIQVIREYTSKVDKLEQTEA 1620

ERNAHDCFAACLFQCYDLLRPDVILELAWKHNIMDFAMPYLIQVIREYT+KVDKLEQTEA

Sbjct 1561 ERNAHDCFAACLFQCYDLLRPDVILELAWKHNIMDFAMPYLIQVIREYTTKVDKLEQTEA 1620

Query 1621 QREKEDETVEHKNIITMEPQLMITAGPAMGIPPQYAQNYAAAPGYAPNMAYPGYGM 1676

QREKEDETVEHKNIITMEPQLMITAGPAMGIPPQYAQNYAAAPGYAPNMAYPGYGM

Sbjct 1621 QREKEDETVEHKNIITMEPQLMITAGPAMGIPPQYAQNYAAAPGYAPNMAYPGYGM 1676

**Adaptor protein 50 (AP50)**

**>lcl|ORF10**

MIGGLFVYNHKGEVLISRVYRDDIGRNAVDAFRVNVIHARQQVRSPVTNI

ARTSFFHIKRANIWLAAVTKQNVNAAMVFEFLLKIIEVMQSYFGKISEEN

IKNNFVLIYELLDEILDFGYPQNTDSGTLKTFITQQGIKSATKEEQMQIT

SQVTGQIGWRREGIKYRRNELFLDVLEYVNLLMSPQGQVLSAHVAGKVVM

KSYLSGMPECKFGINDKIVMESKGRGIGGNSEAETSRSGKPVVVIDDCQF

HQCVKLSKFETEHSISFIPPDGEFELMRYRTTKDISLPFRVIPLVREVGR

TKMEVKIVLKSNFKPSLLGQKIEVKIPTPLNTSGVQLICLKGKAKYKASD

NAIVWKIKRMAGMKETQLSAEIELLETDTKKKWTRPPISMNFEVPFAPSG

FKVRYLKVFEPKLNYSDHDVVKWVRYIGRSGLYETRC

**Graphical representation – ORF**


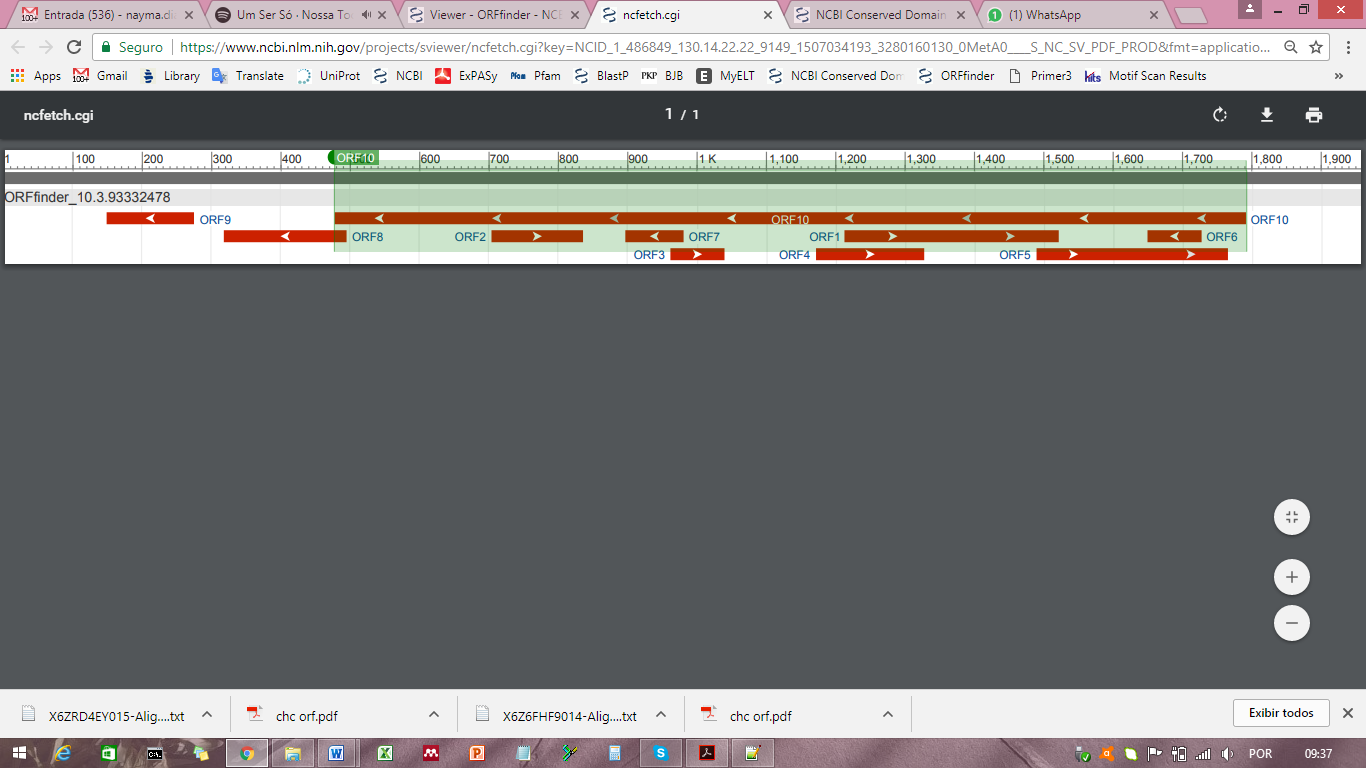


**BlastP (Non-redundant NCBI sequences)**

AP-2 complex subunit mu [Ceratitis capitata]

Sequence ID: [XP_004530702.1](https://www.ncbi.nlm.nih.gov/protein/498985821?report=genbank&log$=protalign&blast_rank=1&RID=X71AE5K6015) Length: 437 Number of Matches: 1

Range 1: 1 to 437

Score: 904 bits(2336)

E-value: 0.0

Identities: 436/437(99%)

Query 1 MIGGLFVYNHKGEVLISRVYRDDIGRNAVDAFRVNVIHARQQVRSPVTNIARTSFFHIKR 60

MIGGLFVYNHKGEVLISRVYRDDIGRNAVDAFRVNVIHARQQVRSPVTNIARTSFFHIKR

Sbjct 1 MIGGLFVYNHKGEVLISRVYRDDIGRNAVDAFRVNVIHARQQVRSPVTNIARTSFFHIKR 60

Query 61 ANIWLAAVTKQNVNAAMVFEFLLKIIEVMQSYFGKISEENIKNNFVLIYELLDEILDFGY 120

ANIWLAAVTKQNVNAAMVFEFLLKIIEVMQSYFGKISEENIKNNFVLIYELLDEILDFGY

Sbjct 61 ANIWLAAVTKQNVNAAMVFEFLLKIIEVMQSYFGKISEENIKNNFVLIYELLDEILDFGY 120

Query 121 PQNTDSGTLKTFITQQGIKSATKEEQMQITSQVTGQIGWRREGIKYRRNELFLDVLEYVN 180

PQNTDSGTLKTFITQQGIKSATKEEQMQITSQVTGQIGWRREGIKYRRNELFLDVLEYVN

Sbjct 121 PQNTDSGTLKTFITQQGIKSATKEEQMQITSQVTGQIGWRREGIKYRRNELFLDVLEYVN 180

Query 181 LLMSPQGQVLSAHVAGKVVMKSYLSGMPECKFGINDKIVMESKGRGIGGNSEAETSRSGK 240

LLMSPQGQVLSAHVAGKVVMKSYLSGMPECKFGINDKIVMESKGRGIGGNSEAETSRSGK

Sbjct 181 LLMSPQGQVLSAHVAGKVVMKSYLSGMPECKFGINDKIVMESKGRGIGGNSEAETSRSGK 240

Query 241 PVVVIDDCQFHQCVKLSKFETEHSISFIPPDGEFELMRYRTTKDISLPFRVIPLVREVGR 300

PVVVIDDCQFHQCVKLSKFETEHSISFIPPDGEFELMRYRTTKDISLPFRVIPLVREVGR

Sbjct 241 PVVVIDDCQFHQCVKLSKFETEHSISFIPPDGEFELMRYRTTKDISLPFRVIPLVREVGR 300

Query 301 TKMEVKIVLKSNFKPSLLGQKIEVKIPTPLNTSGVQLICLKGKAKYKASDNAIVWKIKRM 360

TKMEVK+VLKSNFKPSLLGQKIEVKIPTPLNTSGVQLICLKGKAKYKASDNAIVWKIKRM

Sbjct 301 TKMEVKVVLKSNFKPSLLGQKIEVKIPTPLNTSGVQLICLKGKAKYKASDNAIVWKIKRM 360

Query 361 AGMKETQLSAEIELLETDTKKKWTRPPISMNFEVPFAPSGFKVRYLKVFEPKLNYSDHDV 420

AGMKETQLSAEIELLETDTKKKWTRPPISMNFEVPFAPSGFKVRYLKVFEPKLNYSDHDV

Sbjct 361 AGMKETQLSAEIELLETDTKKKWTRPPISMNFEVPFAPSGFKVRYLKVFEPKLNYSDHDV 420

Query 421 VKWVRYIGRSGLYETRC 437

VKWVRYIGRSGLYETRC

Sbjct 421 VKWVRYIGRSGLYETRC 437

**TRF3**

**>lcl|ORF2**

MSSAKMGYTTIVYLTTALLGVVSLASVQADEQIYRMCVPQLYYKDCLDLL

KDPSEAGIQMECVAGRDRIDCLDKINQRKADVLASEPEDMYVAYHTKNQD

YRVVSEIRSKADKDAEFRYEGIILVKKNSNINSLKELRGKKSCHTGFGRN

VGYKIPITKLKNTHILKISLDPEITATERELKALSEFFTQSCLVGTYSPY

PETDRLLSKSLEVEP

**Graphical representation – ORF**


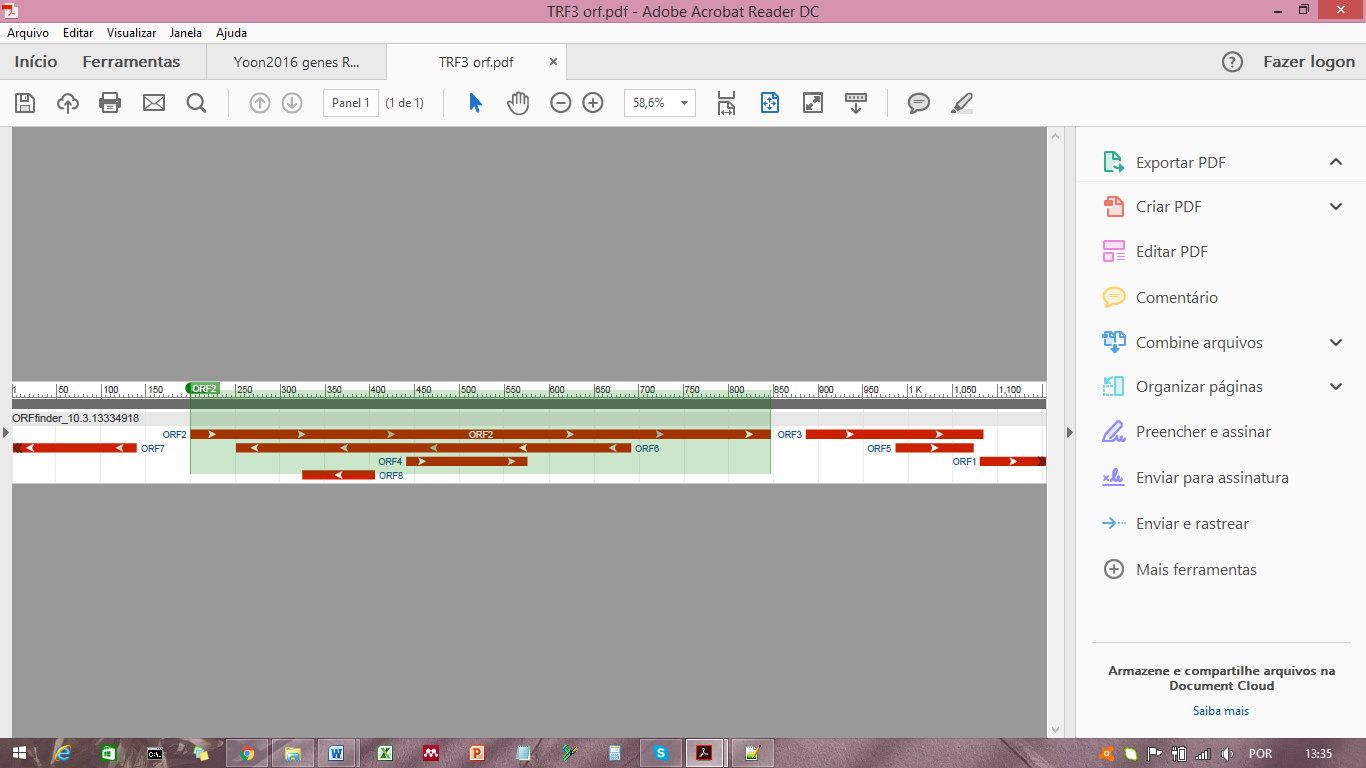


**BlastP (Non-redundant NCBI sequences)**

PREDICTED: transferrin-like, partial [Rhagoletis zephyria]

Sequence ID: [XP_017488222.1](https://www.ncbi.nlm.nih.gov/protein/1048020101?report=genbank&log$=protalign&blast_rank=1&RID=X7F0XZBZ014) Length: 506 Number of Matches: 1

Range 1: 1 to 204

Score: 395 bits(1016)

E-value: 2e-134

Identities: 187/204(92%)

Query 6 MGYTTIVYLTTALLGVVSLASVQADEQIYRMCVPQLYYKDCLDLLKDPSEAGIQMECVAG 65

MG+T VYL TALLGV SLA VQADEQIYRMCVPQ YYKDCLDLLKDPSEAGI+MECVAG

Sbjct 1 MGFTRTVYLATALLGVASLALVQADEQIYRMCVPQKYYKDCLDLLKDPSEAGIKMECVAG 60

Query 66 RDRIDCLDKINQRKADVLASEPEDMYVAYHTKNQDYRVVSEIRSKADKDAEFRYEGIILV 125

RDRIDCLDKINQRKADVLASEPEDMYVAYHTKNQDYRV+SEIR+K DKDAEFRYEGIILV

Sbjct 61 RDRIDCLDKINQRKADVLASEPEDMYVAYHTKNQDYRVISEIRTKEDKDAEFRYEGIILV 120

Query 126 KKNSNINSLKELRGKKSCHTGFGRNVGYKIPITKLKNTHILKISLDPEITATERELKALS 185

KKNSNINS+KELRGKKSCHTGFGRNVGYKIPITKLKNTHILK+SLDP++TATERELKALS

Sbjct 121 KKNSNINSMKELRGKKSCHTGFGRNVGYKIPITKLKNTHILKVSLDPDVTATERELKALS 180

Query 186 EFFTQSCLVGTYSPYPETDRLLSK 209

EFFTQSCLVG+YSPYPETDRLL K

Sbjct 181 EFFTQSCLVGSYSPYPETDRLLKK 204

**Sortilin Like Receptor (SLR)**

**>lcl|ORF102**

MRELCDNIYDCVDYSDEKDCAIEDIERNIFDEDEIIREYAPHQRKPAPTE

GPSLDGLDAQEYYLVQQNLYQKASARNPCNSQQFRCGNNVCIPLQLRCDG

FYHCNDMTDEYGCDQYNAVRRTTARPSNLMRTTEAGQMIPWWRTTAATTT

PRPTAGRWNTTFPTTTRIATTVAPSYNNTCLENIEFTCQNGDCIPIESVC

DGTADCARREDEDYNLCTCSSDKWKCLHGGGCIPKTQVCDGKRQCKDGSD

ESICRCAANQFRCRNGDCVSGSAYCNGYRDCRDGSDEEDCSEPSYDAGRG

CSVSQFRCSNGQCINAAARCDGYTDCADSSDELSCRVPDASPSNPQLNLR

TYPDSQIIKESREVIFRCRDEGPVRARVKWTRPGGRPLPIGSRDNGDGRL

EIPNIRSEDSGQYICEAAGYPRHVPGQQVTVHLTVEKSNPTNDRPPTACR

ANQATCRNNECIDKSQICDGIPHCSDGSDEESCSHGLKCQPNQFMCRNSR

CIDRIWRCDGEDDCFDNSDEDSCDPEPSGAPCRYDEFQCRSGHCIPKNFQ

CDDTNDCIDGTDEIGCMAPAPIRPPPPYIRLQQGDPLNLTCVGTGVPVPL

IVWRLNWGHVPEKCVAKSHAGTGTLYCPDMDTQDQGAYSCEILNSKGRNF

VTPDTQVTVDAREHVDVCPAGFFNMLARRQDECINCFCFGISKSCKSANL

YNFAIQPTIISHKVKNVELNPYSDIIINDAQSPDILSHHHGVQFRVSDVG

YNSREQPYLALPNEYMGNQLKSYGGYLRYEVNYMGNGRLNQAPDVIITGN

GFTLTHRVRSQPEPNVNNKISVQFNSGNWYKTDGRRATREEIMMVLANVD

NILIRLSYIDATEREVELTNIVMDSAGLDDQGLGSASLVEKCACPPGYVG

DSCESCAPGYVREKYGPWLGRCVPFVPEPCQPGTYGDPGRGIPCRQCPCP

QTGANNFASGCQLSPDGDVTCNCYEGYTGRRCEICAPGYQGNPLIPGGSC

HRIPESTCNAEGTYYPHPNGTCECKPLVVGPRCDTCAPESFHLNAFTYTG

CIECFCSGLTKQCSSSSWYRDQVSSNFGRSRAPHGFNLIRDYETSQPSQV

SFQQANSALQFSQQSTSDPLYWSLPAPFLGDRITAYGGKLSYNLSYNPLP

GGLMSRNNAPDVVIKSGEDLTIIHYRKAGVNPNQPSSYSVPIIESAWQRS

DGQVVNREHLLMALSKIDAIYIKATYTTSTKDGQLTHVSLDIATQNKIGT

QRAFEVEECRCPVGYIGLSCERCAPGYKRDTEAGLYLGVCEPCDCNGHSN

QCDAETGVCMNCDHNTAGEFCERCSPGYTGNASGRTPYDCSPDSGPSPYP

PPAPGNQTQCSYCNRDGTVSCDGGYCACKPNVVGPYCDQCRPGTYGLSER

NPDGCEECYCSGKSSRCNSASLYRQLIPVDFISSQPLITDGEGNIVDTEN

LSFDLPTNMYTYSFTSYTEKYWSLRGSVLGNQLYSYGGHLSYALDVDSYG

HYEPGTDVILIGNGMKLLWSRSSDEQEDTEYRVRLHEDENWLRNERGHSV

RASRLDIMSVLANLEHILIRATPKIPTTRTSIRDVILESAVEHRTPDAQL

AVEVEVCSCPAGYSGNSCEKCEPLHYKDSYGSCVACPCREDTTVGCSLQN

NGEVLCQCRSGYSGQQCQIDVKANKNESSEKSTLIDKPDSLVCVCCNDYH

VDLVPNVTIAYNETLQLFRGNRKIGNITKLRLDCNRRDDYERPTQAPPTP

APQPDYKTQITVSIAPPEITIIPIGGSLTLSCTGRMAWSGVPVFVSWSKL

NGRLPYNAEEDGGVLRLYDLQIYDSGVYICRAVNNQTQRVFEDQISITIT

EDSQRTPSQIINLPQVISFEEYQPNEIYCEVNGHPTPTVTWTRVDGQMSL

EAHTDGSRLIFDAPRKSDEGRYRCQATNEIGIDEKYTQVYVHIAPPQPPP

PPRELVYIDPPSYTGETGDIVRLTCQPTTSILLVYEWNKDGYPVYRQRNV

IISGNTIEIRESTPRDSGLYTCIGIDQRGRRNYTNDAQVVIEDSSYSVPG

PQPGPLPGTIAPVVKRLPDENHIVQGQDFSITCEATGSPYPNIKWTKVHE

ALADNVQQTGNVLRIINARPENRGMYLCIAENVAGSHESNTIIDVEPRES

PSVDIHPPEPQTVNVGTEAMLYCSATGIPDPSVQWHRVDGKPLSPRSKEI

SAGYIIIGNIQVEDSGDYECVAKNDVGRATGIQTVRVLVPPMITLEPSDE

VLSLTEGDELKVVCTATGVPSPTVQWVDESTEFTRASLPTSPQYNEAFLE

KYRIDRNDAKSYKCIASNEAGTAESYISVDVRPRRGDASNDSDVIRNPQY

PYPYQSQPPPQYPPTRQPGYGPPQNIYRANEGDAVTLTCDLNNVPNLSTR

WERVDGRPLPDNSYFERNSLIIKRVEEQNSGKYRCNALDNRGAVVTFVIA

ELVLVPIPHITFHPKMPIVVAPNDNIDIYCEVTGEQPIDVAWHTDNNRPL

TDSVRIDGQYLRFVSITPADAGRYYCSASNRYGNTTNMAEVVVNRNGAYQ

PAPRAQVYDIPEGEDVTLQCDVQNAREPIRGEIRYEWRRQDGRPLPRSAL

IRDQRLYLRGVLKQDEGRYVCESYSTGGGRSQPSFVDLNIKPAAAPQFRK

PIIPQPLPERPTPPPQQQTRPRDLSLKLDQQSSQLRIGESTEVECYSSDS

SYTDVIWERADGSPLPPHIQQIGNRLIISHVSASDAGNYVCKCKTDEGDL

YTTSYELGIEEWPHEWKRPKLVHASVGSTAQLNCDAEIPGSYRWSRQYGQ

MQADRVLFNERLELQDVQANDAGTYICTSNTPDGLSIDYPTILVVTGAIP

QFHQEPISYMSFPTLPESYIKFNFDITFRPEQGNGLLLFNGQKRGSGDYI

SLSLKDRYPEFRFDFDGKPLVIRAERPVSLKEWHTIRVNRFRRDGYMQVD

DQHPVAFPTQAQISPLDLIEDLFIGGVPNWDMLPLDAIDQQVGFVGCISR

LTLQGTIVELMKEAKLKEGVTACRPCQQNPCKNGGICLESQTETAYTCIC

QQGWTGNNCGVEGTQCTTGICGSGRCENTEFGMECLCPLNKTGDRCQYIE

HLNENSLAFKLNSYAAYPTPRASKLNVKFKVRPNSLQDAVLLYAAESKLP

SGDFIAVLLHDKHVELIINTGARLNPILVRSQNPLPINKWTEIEVSRRFG

EGILRVGNEPEQRAKATKLARILYIKTPLYLGGYDHENIKLNRDVNITQG

FDGCISSLFEGQRQINLIADIHDAANIQNCGEINEIDQNESFDNYEHEAT

NIQQASGKPIESPSEKEDQQVDACASDPCENGGNCSVHNGKAVCACTIGF

TGRHCEEHITIEYDANFHGNGYLEVDRNQFNSDVDQKYSFAAMVFSTTDP

NGLLLWWGQKRDEEYTGQDFMALAIVDGTVEFSFRLNGEETVIRNPDKRI

DDGRRHIVLIKRTDNTAILELDHLLDAGETRPTGKDKMSLPGHVFIGGTP

DVAKFTGGRYTKNFNGCVRVVEGDAHGLIQLGTAAISGENVDICPQADDE

SLGTEPPVV

**Graphical representation – ORF**


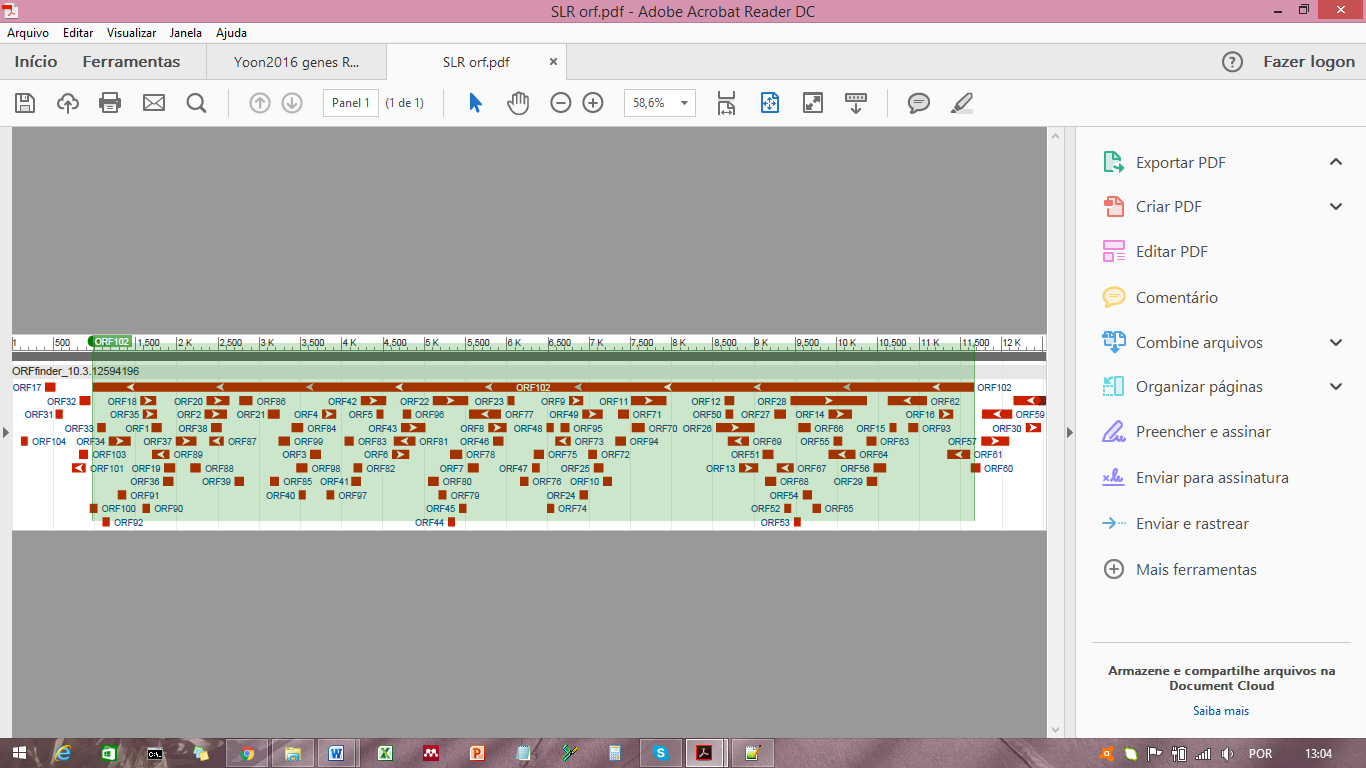


**BlastP (Non-redundant NCBI sequences)**

PREDICTED: basement membrane-specific heparan sulfate proteoglycan core protein isoform X2 [Drosophila miranda]

Sequence ID: [XP_017136706.1](https://www.ncbi.nlm.nih.gov/protein/1036803937?report=genbank&log$=protalign&blast_rank=1&RID=X7D2TX9S014)Length: 4638Number of Matches: 7

Range 1: 1011 to 4638

Score: 4167 bits(10808)

E-value: 0.0

Identities: 2139/3682(58%)

Query 5 CDNIYDCVDYSDEKDCAI--EDIE------RNIFDEDEIIREYAPHQRKPAPTE------ 50

C+ I DC D +DE +C ED++ N +E +I+ E+ + P E

Sbjct 1011 CNGITDCADGADEYNCLFNYEDVDYDTDPDNNPLNECDIL-EFECDLGQCLPLEKKCDGY 1069

Query 51 ---GPSLDGLDAQEYYLVQQNLYQKASARNPCNSQQFRCGNNVCIPLQLRCDGFYHCNDM 107

G D LD + V + +S+ C +F C + C+P C+G +CND

Sbjct 1070 TDCGDETDELDCPAF-TVSVIYHIPSSSTEHCLENEFEC-DEYCMPRDQLCNGIANCNDA 1127

Query 108 TDEYGC-----DQY----------NAVRRTTARPSNLMRTTEAGQMIPWWRTTAATTT-- 150

+DE C D Y N A ++ ++ W T A T

Sbjct 1128 SDERNCTFCRDDAYLCNTGECIADNQRCNGHADCTDASDERHCARVACPWHTMACNGTCV 1187

Query 151 ---------PRPTAGRWNTTFPTTTRIATTVAPSYNNTCLENIEFTCQNGDCIPIESVCD 201

+ GR PT + A + S +CL +++ C N +CI CD

Sbjct 1188 NRSIRCDGKRDCSDGRDERDCPTDVKSAAGLPIS--KSCLP-LQWQCANFECIDRRQYCD 1244

Query 202 GTADCARREDEDYNLCT-----------CSSDKWKC----------------------LH 228

DC DE C CSSD++ C H

Sbjct 1245 NRKDCRDGSDEFPVNCIGNATTRLTAADCSSDQFFCDDSCFNHSIRCNGVSDCSDGRDEH 1304

Query 229 G----------------------GGCIPKTQVCDGKRQCKDGSDESICRCAANQFRCRNG 266

G G C +++ CD +R C+DG DE+ C CA+NQFRC NG

Sbjct 1305 GCHHSIPPPSSHFLPCPQHTCPSGRCYSESERCDRRRHCEDGFDEANC-CASNQFRCHNG 1363

Query 267 DCVSGSAYCNGYRDCRDGSDEEDCSEPSYDAGRGCSVSQFRCSNGQCINAAARCDGYTDC 326

DCV S+ C+G CRDGSDE +C C SQFRC+NGQC++A RC+G TDC

Sbjct 1364 DCVPYSSTCDGIPQCRDGSDELECG-----GSLECLPSQFRCNNGQCVSATVRCNGKTDC 1418

Query 327 ADSSDELSCRVPDASPSNPQLNLRTYPDSQIIKESREVIFRCRDEGPVRARVKWTRPGGR 386

DSSDE +C + + QLNL+TYPDSQIIKE REVIFRCRDEGP RA+V+WTRPGG+

Sbjct 1419 QDSSDEQNC-ANEPGRTTSQLNLKTYPDSQIIKERREVIFRCRDEGPFRAKVRWTRPGGQ 1477

Query 387 PLPIGSRDNGDGRLEIPNIRSEDSGQYICEAAGYPRHVPGQQVTVHLTVEKSNPTNDRPP 446

PLP G D GRLEIPNIR EDSG Y+CEA GYP + GQQVTVHLTVE+ N RP

Sbjct 1478 PLPPGFTDRS-GRLEIPNIRLEDSGTYVCEAVGYPSYQQGQQVTVHLTVERYNDLGARPS 1536

Query 447 TACRANQATCRNNECIDKSQICDGIPHCSDGSDEESCSHGLKCQPNQFMCRNSRCIDRIW 506

TAC QATC N +CIDKS ICDGIPHCSDGSDE SCSHG KCQPNQF+C NS+C+DR W

Sbjct 1537 TACSQYQATCMNGDCIDKSNICDGIPHCSDGSDEHSCSHGRKCQPNQFLCNNSKCVDRTW 1596

Query 507 RCDGEDDCFDNSDEDSCDPEPSGAPCRYDEFQCRSGHCIPKNFQCDDTNDCIDGTDEIGC 566

RCDGE+DC DNSDE SCDPEPSGAPCR+ EFQCRSGHCIPK+FQCD+ +DC DG+DE+GC

Sbjct 1597 RCDGENDCGDNSDEASCDPEPSGAPCRFSEFQCRSGHCIPKSFQCDEVSDCSDGSDEVGC 1656

Query 567 MAPAPIRPPPPYIRLQQGDPLNLTCVGTGVPVPLIVWRLNWGHVPEKCVAKSHAGTGTLY 626

M P PIRPPPP L +G+ L+LTCV TG P+P IVWRLNWGHVPEKC +KS AGTGTL+

Sbjct 1657 MEPVPIRPPPPVKGLLEGESLDLTCVATGTPIPTIVWRLNWGHVPEKCESKSFAGTGTLH 1716

Query 627 CPDMDTQDQGAYSCEILNSKGRNFVTPDTQVTVDAREHVDVCPAGFFNMLARRQDECINC 686

CPDM QD GAYSCEI+NS+G +FVTPDT VTV + C AGFFNMLAR ++ECI C

Sbjct 1717 CPDMQVQDSGAYSCEIINSRGTHFVTPDTIVTVTPNRN-QFCEAGFFNMLARSEEECIKC 1775

Query 687 FCFGISKSCKSANLYNFAIQPTIISHKVKNVELNPYSDIIINDAQS-PDILSHHHGVQFR 745

FCFG++ +C+SANL+++AIQP I+SH+V +VEL+PY I+IN+A S D+L+ HHGVQFR

Sbjct 1776 FCFGVASTCESANLFSYAIQPPILSHRVVSVELSPYRQIVINEATSGQDLLTLHHGVQFR 1835

Query 746 VSDVGYNSREQPYLALPNEYMGNQLKSYGGYLRYEVNYMGNGRLNQAPDVIITGNGFTLT 805

S+V Y RE PYLALPN+YMG+QLKSYGG LRYEV+YMG+GR PDVIITGN FTLT

Sbjct 1836 ASNVHYGGRETPYLALPNDYMGSQLKSYGGNLRYEVSYMGSGRPVSGPDVIITGNRFTLT 1895

Query 806 HRVRSQPEPNVNNKISVQFNSGNWYKTDGRRATREEIMMVLANVDNILIRLSYIDATERE 865

HRVR+ P NNK+SV G W K DGR+ATREEIMM+LANVDNILIRL Y+D+ RE

Sbjct 1896 HRVRTLP--GQNNKVSVPLLPGGWQKPDGRKATREEIMMILANVDNILIRLGYLDSVARE 1953

Query 866 VELTNIVMDSAGLDDQGLGSASLVEKCACPPGYVGDSCESCAPGYVREKYGPWLGRCVPF 925

V+L NI +DSAG DQGLGSASLVEKC CPPGYVGDSCESCAPGYVR+ G WLG CVPF

Sbjct 1954 VDLINISLDSAGTVDQGLGSASLVEKCTCPPGYVGDSCESCAPGYVRQPGGAWLGHCVPF 2013

Query 926 VPEPCQPGTYGDPGRGIPCRQCPCPQTGANNFASGCQLSPDGDVTCNCYEGYTGRRCEIC 985

PEPC GTYGDP RGIPCR CPCPQ+G+NNFASGCQL PDG+V CNC+EGY G+RCE C

Sbjct 2014 TPEPCPAGTYGDPRRGIPCRDCPCPQSGSNNFASGCQLRPDGEVLCNCFEGYAGKRCESC 2073

Query 986 APGYQGNPLIPGGSCHRIPESTCNAEGTYYPHPNGTCECKPLVVGPRCDTCAPESFHLNA 1045

A GYQGNPL PGGSCH+ PES+CN +GTY + +G+C CK V+G CD+CAP SFHLN+

Sbjct 2074 AAGYQGNPLAPGGSCHKTPESSCNVDGTYSIYADGSCHCKDRVIGEHCDSCAPNSFHLNS 2133

Query 1046 FTYTGCIECFCSGLTKQCSSSSWYRDQVSSNFGRSRAPHGFNLIRDYETSQPSQVSFQQA 1105

FTYTGCIECFCSG C S++W RDQV+S+FGR+RAPHGF L+RDY +

Sbjct 2134 FTYTGCIECFCSGHQVGCGSTTWNRDQVTSSFGRTRAPHGFELVRDYTRPTAQSLEIATY 2193

Query 1106 NSALQFSQQST--SDPLYWSLPAPFLGDRITAYGGKLSYNLSYNPLPGGLMSRNNAPDVV 1163

S L F+ + +D LYWSLPA FLG+++ +YGGKLSY LSY+PLPGG MSRN+APDVV

Sbjct 2194 GSELSFNAEGEHGADTLYWSLPAVFLGNKLVSYGGKLSYTLSYSPLPGGQMSRNSAPDVV 2253

Query 1164 IKSGEDLTIIHYRKAGVNPNQPSSYSVPIIESAWQRSDGQVVNREHLLMALSKIDAIYIK 1223

IKSGEDL +IHYRK+ V+P SSYSV I ESAWQR DGQ NREH LMALS I AIYIK

Sbjct 2254 IKSGEDLLLIHYRKSPVSPTTASSYSVEIKESAWQRGDGQFANREHTLMALSNITAIYIK 2313

Query 1224 ATYTTSTKDGQLTHVSLDIATQNKIGTQRAFEVEECRCPVGYIGLSCERCAPGYKRDTEA 1283

ATYTTSTK+G L VSLD AT +GTQRA+EVEECRCP GYIGLSCE CAPGYKRD +

Sbjct 2314 ATYTTSTKEGALKQVSLDTATATPLGTQRAYEVEECRCPTGYIGLSCETCAPGYKRDDVS 2373

Query 1284 GLYLGVCEPCDCNGHSNQCDAETGVCMNCDHNTAGEFCERCSPGYTGNASGRTPYDCSPD 1343

GLYLG+CEPC+CNGHS QCDAE+G C+NC NT G C+RC+ GY G+A+G TPYDC PD

Sbjct 2374 GLYLGLCEPCECNGHSTQCDAESGDCINCSDNTEGPNCDRCAAGYVGDATGGTPYDCQPD 2433

Query 1344 SGPS--PYPPPAPGNQTQ-CS-YCNRDGTVSCDGGYCACKPNVVGPYCDQCRPGTYGLSE 1399

G PY P PGNQT CS +C +GTV+C G YC CKPNV G CDQCRPGTYGLS

Sbjct 2434 GGEPQPPYRPLEPGNQTSDCSRWCQAEGTVNCQGNYCYCKPNVNGDRCDQCRPGTYGLSA 2493

Query 1400 RNPDGCEECYCSGKSSRCNSASLYRQLIPVDFISSQPLITDGEGNIVDTENLSFDLPTNM 1459

NPDGC+ECYCSG+S++C SA LYRQLIPVDF + PL+TD G I D NL FD+ TNM

Sbjct 2494 ANPDGCKECYCSGQSTQCRSAPLYRQLIPVDFFKNPPLLTDETGEIQDENNLDFDVATNM 2553

Query 1460 YTYSFTSYTEKYWSLRGSVLGNQLYSYGGHLSYALDVDSYGHYEPGTDVILIGNGMKLLW 1519

YTYS SY KYWSLRGSVLGNQL+SYGG L Y L V+SYG YEPG DVILIGNG+ L+W

Sbjct 2554 YTYSHPSYLPKYWSLRGSVLGNQLHSYGGELQYKLRVESYGRYEPGQDVILIGNGLTLIW 2613

Query 1520 SRSSDEQEDTEYRVRLHEDENWLRNERGHSVRASRLDIMSVLANLEHILIRATPKIPTTR 1579

S + E D RVRL+ED W R +RG +V A+R D M+VL++LEHILIRATPK+PTTR

Sbjct 2614 S-PNQESPDGLNRVRLNEDGQWQRKDRGRTVPATRSDFMTVLSDLEHILIRATPKVPTTR 2672

Query 1580 TSIRDVILESAVEHRTP-DAQLAVEVEVCSCPAGYSGNSCEKCEPLHYKDSYGSCVACPC 1638

TSI DVILESAV + +P Q A ++E+C CPAGY+GNSCE C PLHY+D+ G+C CPC

Sbjct 2673 TSIGDVILESAVTYASPGHGQAATDIELCQCPAGYTGNSCESCAPLHYRDADGACRLCPC 2732

Query 1639 REDTTVGCSLQNNGEVLCQCRSGYSGQQC-QIDVKANKNESSEKSTLIDKPDSLVCV--- 1694

D + GC L +NG CQC+ + G+ C +ID S +I++P + +

Sbjct 2733 DADNSDGCRLGSNGYPECQCKPRFKGELCREIDT----------SPIIEEPPKICDISRG 2782

Query 1695 -CCNDYHVDLVPNVTIAYNETLQLFRGNRKIGNITKLRLDCNRRDDYERPTQAPPTPAPQ 1753

CC+ + ++ PN TI++N+TLQ+++GNR IGNITKLR C+ RD P Q P+

Sbjct 2783 FCCSGFRFNIAPNETISFNDTLQIYKGNRIIGNITKLRYGCHLRDTGNEPEQE--PEEPE 2840

Query 1754 PDYKTQITVSIAPPEITIIPIGGSLTLSCTGRMAWSGVPVFVSWSKLNGRLPYNAEEDGG 1813

+ +TQI VSIA P+ITI+P+GGSLTLSC+G M W+ PV VSW K N RLP ++E +GG

Sbjct 2841 DNGRTQIIVSIARPQITIVPVGGSLTLSCSGHMRWNNDPVVVSWYKQNSRLPEDSEVEGG 2900

Query 1814 VLRLYDLQIYDSGVYICRAVNNQTQRVFEDQISITITEDSQRTPSQIINLPQVISFEEYQ 1873

VL LY+LQI DSG+Y+C A NN+T V++D +S+TI+++ QR+P++I++LP ++FEEYQ

Sbjct 2901 VLHLYNLQITDSGIYVCHAENNETMHVYQDTVSVTISQEGQRSPARIVDLPNHVTFEEYQ 2960

Query 1874 PNEIYCEVNGHPTPTVTWTRVDGQMSLEAHTDGSRLIFDAPRKSDEGRYRCQATNEIGID 1933

PNEI CEV G+PTP+VTWTR+DGQ + TDG+ LIFD+PRKSDEGRYRCQA N + D

Sbjct 2961 PNEINCEVEGNPTPSVTWTRIDGQADAQTRTDGTHLIFDSPRKSDEGRYRCQAENSLNRD 3020

Query 1934 EKYTQVYVHIAPPQPPPPPRELVYIDPPSYTGETGDIVRLTCQPTTSILLVYEWNKDGYP 1993

EKY VYV + P PPP RE VYI P Y G GD RLTCQ T+ L YEW+ +GYP

Sbjct 3021 EKYVHVYVQSSAPPSPPP-RERVYIQPEEYNGVEGDTFRLTCQSTSGANLHYEWSLNGYP 3079

Query 1994 VYRQRNVIISGNTIEIRESTPRDSGLYTCIGIDQRGRRNYTNDAQVVIEDSSYSVPGPQP 2053

+ QRN+++SGN +E+R+S+ RDSG YTC D R RNYT DA+V IE + +P

Sbjct 3080 LSGQRNIMVSGNVLEVRDSSVRDSGTYTCGAYDLRTHRNYTEDARVYIE--RHDLP---- 3133

Query 2054 GPLPGTIAPVVKRLPDENHIVQGQDFSITCEATGSPYPNIKWTKVHEALADNVQQTGNVL 2113

P + +PV+ RL + I QG+D+SITCEA+G+PYP+IKWTKVH+ LA NV +GNVL

Sbjct 3134 -PTDDSSSPVIVRLQELITIEQGRDYSITCEASGTPYPSIKWTKVHDHLATNVHVSGNVL 3192

Query 2114 RIINARPENRGMYLCIAENVAGSHESNTIIDVEPRESPSVDIHPPEPQTVNVGTEAMLYC 2173

I +ARPENRG Y CIAEN+ GS +S+T ID+EPRE PSV I P QT +VG++A LYC

Sbjct 3193 SIYDARPENRGPYSCIAENIHGSDQSSTNIDIEPRERPSVKIESPSLQTHSVGSQASLYC 3252

Query 2174 SATGIPDPSVQWHRVDGKPLSPRSKEISAGYIIIGNIQVEDSGDYECVAKNDVGRATGIQ 2233

A GIP+P VQW RVDG PLSPR KE+ GY++I +IQ+ D+G YEC A N VG+A+G

Sbjct 3253 RANGIPEPQVQWVRVDGTPLSPRHKEMGRGYVVIDDIQIADAGAYECRADNQVGKASGTA 3312

Query 2234 TVRVLVPPMITLEPSDEVLSLTEGDELKVVCTATGVPSPTVQWVDESTE-FTRASLPTSP 2292

++RV+ P++ ++P +++ LT+GDEL + C A+G P+P+VQW + E T SL +

Sbjct 3313 SLRVVEAPLVVIKPDQQIIRLTDGDELNLECIASGYPNPSVQWSPKGQEPETETSLGVNR 3372

Query 2293 QY--NEAFLEKYRIDRNDAKSYKCIASNEAGTAESYISVDVRPRRGDAS-NDSDVIRNPQ 2349

N A+L+ YR+ +DA Y C NEAG E + VD++P+RGD S ND DV P

Sbjct 3373 DLVSNTAYLKIYRVSLSDAGIYTCSGVNEAGNDERSVRVDIQPKRGDISDNDGDVDHGP- 3431

Query 2350 YPYPYQSQPPPQYPPTRQPGYGPPQNIYRANEGDAVTLTCDLNNVPNLSTRWERVDGRPL 2409

YQ+ PP PP +PG PQ + R N G+ VTLTCDL TRW RVDGRPL

Sbjct 3432 ----YQTDYPP--PPASRPGQ--PQRL-RTNIGENVTLTCDLGP---YVTRWVRVDGRPL 3479

Query 2410 PDNSYFERNSLIIKRVEEQNSGKYRCNALDNRGAVVTFVIAELVLVPIPHITFHPKMPIV 2469

P N+Y ERNSL+I V EQN G+YRCNA DN VVT+V+ ELVL+P+P ITF PK+P+

Sbjct 3480 PSNAYTERNSLVIIYVHEQNLGQYRCNAYDNND-VVTYVVRELVLLPLPQITFQPKIPLR 3538

Query 2470 VAPNDNIDIYCEVTGEQPIDVAWHTDNNRPLTDSVRIDGQYLRFVSITPADAGRYYCSAS 2529

V +N++IYCEVT QP DV W TDNNRPL+ SVRIDGQ LRF+SI PADAG Y C+A+

Sbjct 3539 VDAGENVEIYCEVTNAQPEDVHWATDNNRPLSGSVRIDGQMLRFISIAPADAGGYRCTAT 3598

Query 2530 NRYGNTTNMAEVVVNRNGAYQPAPRAQVYDIPEGEDVTLQCDVQNAREPIRGEIRYEWRR 2589

N YGNTT A+VVVN Y+ P+++V+ EGE++ L+C E RG +++EW R

Sbjct 3599 NYYGNTTKTAQVVVNPPTEYEQVPQSEVHQRREGENIQLRCSATVHGEE-RGNLQFEWYR 3657

Query 2590 QDGRPLPRSALIRDQRLYLRGVLKQDEGRYVCESYSTGGGRSQP-SFVDLNIKPAAAPQF 2648

QDGRPLP A Q L L + QD GRY+C+ Y G+ P + VDL + P

Sbjct 3658 QDGRPLPNGARRDSQVLVLTTLRPQDAGRYICDVYDHASGQRLPATAVDLQVHPGLPVVP 3717

Query 2649 RKPIIPQPLPERPTPPPQQQTRPRDLSLKLDQQSSQLRIGESTEVECYSSDSSYTDVIWE 2708

P P PP + R RD SLKLDQQSS LR GESTEVECYSSD+SYTDV+WE

Sbjct 3718 VTPPPASYRPAY-LPPAKTPGRSRDYSLKLDQQSSNLRAGESTEVECYSSDNSYTDVVWE 3776

Query 2709 RADGSPLPPHIQQIGNRLIISHVSASDAGNYVCKCKTDEGDLYTTSYELGIEEWPHEWKR 2768

RAD PL P++QQ+GNRL+++ V+ +DAG YVCKC+TDEGDLYTTSYEL IE+ PHE KR

Sbjct 3777 RADSQPLSPNVQQVGNRLVMTDVTVADAGQYVCKCRTDEGDLYTTSYELTIEQQPHELKR 3836

Query 2769 PKLVHASVGSTAQLNCDAEIPG--SYRWSRQYGQMQADRVLFNERLELQDVQANDAGTYI 2826

K+VH+ VG+ A+L C A+ SYRWSRQYGQ+Q R L E+L L+D+QANDAGTYI

Sbjct 3837 SKIVHSKVGADAKLQCGADTSRQPSYRWSRQYGQLQPGRSLLGEKLSLEDLQANDAGTYI 3896

Query 2827 CTSNTPDGLSIDYPTILVVTGAIPQFHQEPISYMSFPTLPESYIKFNFDITFRPEQGNGL 2886

CT+ DG S+DYP+ILVVTGAIP FHQ P SYMSFPTLP+S K NF+ITFRPE +GL

Sbjct 3897 CTALYGDGESVDYPSILVVTGAIPHFHQAPRSYMSFPTLPDSSFKLNFEITFRPEGDDGL 3956

Query 2887 LLFNGQKRGSGDYISLSLKDRYPEFRFDFDGKPLVIRAERPVSLKEWHTIRVNRFRRDGY 2946

LLFNGQ RG+GDYI+LSLKDRY EFRFDF GKP++IRAE P+ L EWHT+RV+R RRDGY

Sbjct 3957 LLFNGQTRGTGDYIALSLKDRYAEFRFDFGGKPMLIRAEEPLQLNEWHTVRVHRSRRDGY 4016

Query 2947 MQVDDQHPVAFPTQAQISPLDLIEDLFIGGVPNWDMLPLDAIDQQVGFVGCISRLTLQGT 3006

MQVD+QHPVAFPT +Q+ PLDLIEDL+IGGVP+W++LP DA+ QQ GFVGCISRLTLQ

Sbjct 4017 MQVDEQHPVAFPTLSQVPPLDLIEDLYIGGVPSWELLPADAVTQQTGFVGCISRLTLQSR 4076

Query 3007 IVELMKEAKLKEGVTACRPCQQNPCKNGGICLESQTETAYTCICQQGWTGNNCGVEGTQC 3066

VELM+EAK KEG+T+C+PC Q+PC NGGICLESQTE AY+C+CQQGWTG NC V GTQC

Sbjct 4077 TVELMREAKFKEGITSCQPCAQSPCSNGGICLESQTEVAYSCVCQQGWTGRNCAVSGTQC 4136

Query 3067 TTGICGSGRCENTEFGMECLCPLNKTGDRCQYIEHLNENSLAFKLNSYAAYPTPRASKLN 3126

T G+CG+GRCENT+ MECLCPLN+TGDRCQYIEHLNE SL FK NSYAAY TPR +++N

Sbjct 4137 TPGVCGAGRCENTDLDMECLCPLNRTGDRCQYIEHLNEQSLNFKRNSYAAYGTPRVTRVN 4196

Query 3127 VKFKVRPNSLQDAVLLYAAESKLPSGDFIAVLLHDKHVELIINTGARLNPILVRSQNPLP 3186

+ VRP+SL+D+VLLYAAESKLPSGD++A++L + HVEL+INT ARL P++VRS PLP

Sbjct 4197 ITLSVRPSSLRDSVLLYAAESKLPSGDYLALVLREGHVELLINTAARLRPVVVRSAEPLP 4256

Query 3187 INKWTEIEVSRRFGEGILRVGNEPEQRAKATKLARILYIKTPLYLGGYDHENIKLNRDVN 3246

+++WT IEV RR GE ILRVG EQRAKA+ AR L +KTPLY+GGYD ++K+NRDVN

Sbjct 4257 LHRWTRIEVLRRQGESILRVGEGQEQRAKASGTARTLSLKTPLYVGGYDRASVKINRDVN 4316

Query 3247 ITQGFDGCISSLFEGQRQINLIADIHDAANIQNCGEINEIDQNESFDNYEHEATNIQQAS 3306

IT GFDGCIS L++ QR I L+ADI DAAN+QNCGE+NEI + D + + A

Sbjct 4317 ITDGFDGCISKLYDSQRSIQLLADIKDAANVQNCGELNEIGGGDGGDGEAEGDSEVPVAP 4376

Query 3307 G----KPIESPSEKEDQQVD-----ACASDPCENGGNCSVHNGKAVCACTIGFTGRHCEE 3357

P+ + +++Q+ CASDPCENGG+C +A+C+C +G++G+HC+E

Sbjct 4377 AVVPTAPLPDATSNQEEQLQPYNMAPCASDPCENGGSCIEAENQALCSCPLGYSGKHCQE 4436

Query 3358 HITIEYDANFHGNGYLEVDRNQFNSDVDQKYSFAAMVFSTTDPNGLLLWWGQKRDEEYTG 3417

HI + ++A+F G+GYLE++RNQF++ V+Q ++ AAMVFST+ PNGLLLWWGQ EEY G

Sbjct 4437 HIQVGFNASFRGHGYLEINRNQFDAAVEQVFTSAAMVFSTSKPNGLLLWWGQVAGEEYVG 4496

Query 3418 QDFMALAIVDGTVEFSFRLNGEETVIRNPDKRIDDGRRHIVLIKRTDNTAILELDHLLDA 3477

QDF+ALA+VDG VE+S RLNGEETVI+N D R+DDG+RHIVL+KR +NTAILE+D + +

Sbjct 4497 QDFIALAVVDGYVEYSLRLNGEETVIKNSDTRVDDGQRHIVLVKRVENTAILEVDRISHS 4556

Query 3478 GETRPTGKDKMSLPGHVFIGGTPDVAKFTGGRYTKNFNGCVRVVEGDAHGLIQLGTAAIS 3537

GETRPTGK +M LPG+VFIGG PD+++FTGGR+T NF GC+ VVEGDA G I LG A ++

Sbjct 4557 GETRPTGKKEMKLPGNVFIGGIPDISQFTGGRHTHNFVGCIVVVEGDAVGQINLGQAGVN 4616

Query 3538 GENVDICPQADDESLGTEPPVV 3559

NVD CP D+ GTEPPVV

Sbjct 4617 AVNVDTCPVNDESLGGTEPPVV 4638

**Innexin**

**>lcl|ORF10**

MFDVFGSVKGLLKIDQVCIDNNVFRMHYKATVIILIAFSLLVTSRQYIGD

PIDCIVDEIPLGVMDTYCWIYSTFTVPERLTGITGRDVVQPGVGSHIEGE

DEVKYHKYYQWVCFVLFFQAILFYVPRYLWKSWEGGRLKMLVMDLNCPIV

NDDCKNDRKKILVEYFMGNLNRHNFYAFRFFVCEALNFANVIGQIFFVDF

FLDGEFSTYGSDVLKFTEMEPDERIDPMARVFPKVTKCTFHKYGPSGSVQ

KFDGLCVLPLNIVNEKIYVFLWFWFIILSILSGISLVYRMAVIIGPKLRH

LLLRARSRLAESEEVEAVANKCNIGDWFLLYQLGKNIDPLIYKEVIADLA

REMGEHSSSKQPFEA

**Graphical representation – ORF**


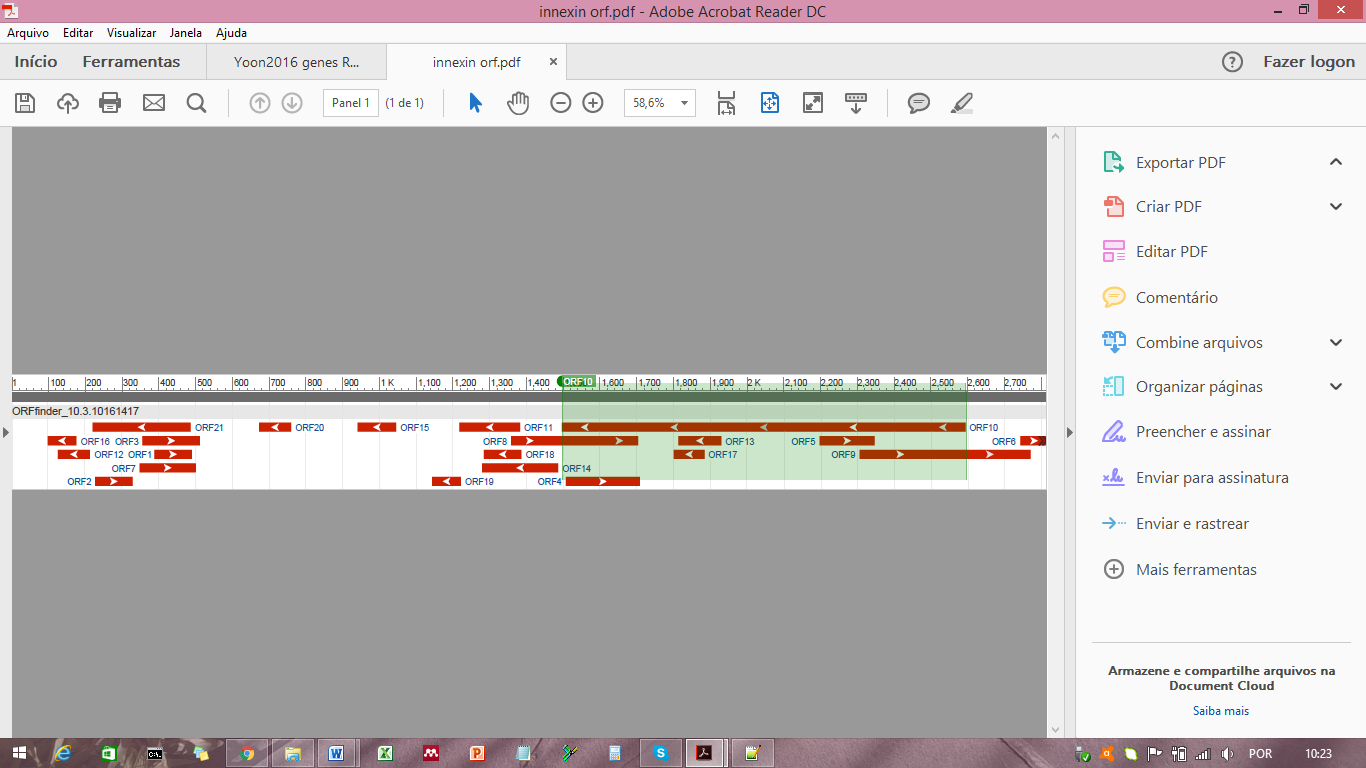


**BlastP (Non-redundant NCBI sequences)**

PREDICTED: innexin inx2 [Rhagoletis zephyria]

Sequence ID: [XP_017488278.1](https://www.ncbi.nlm.nih.gov/protein/1048020204?report=genbank&log$=protalign&blast_rank=1&RID=X73H20MJ014)Length: 365Number of Matches: 1

Range 1: 1 to 365

Score: 739 bits(1907)

E-value: 0.0

Identities: 358/365(98%)

Query 1 MFDVFGSVKGLLKIDQVCIDNNVFRMHYKATVIILIAFSLLVTSRQYIGDPIDCIVDEIP 60

MFDVFGSVKGLLKIDQVCIDNNVFRMHYKATVIILIAFSLLVTSRQYIGDPIDCIVDEIP

Sbjct 1 MFDVFGSVKGLLKIDQVCIDNNVFRMHYKATVIILIAFSLLVTSRQYIGDPIDCIVDEIP 60

Query 61 LGVMDTYCWIYSTFTVPERLTGITGRDVVQPGVGSHIEGEDEVKYHKYYQWVCFVLFFQA 120

LGVMDTYCWIYSTFTVPERLTGITGRDVVQPGVGSHI+G DEVKYHKYYQWVCFVLFFQA

Sbjct 61 LGVMDTYCWIYSTFTVPERLTGITGRDVVQPGVGSHIDGVDEVKYHKYYQWVCFVLFFQA 120

Query 121 ILFYVPRYLWKSWEGGRLKMLVMDLNCPIVNDDCKNDRKKILVEYFMGNLNRHNFYAFRF 180

ILFYVPRYLWKSWEGGRLKMLVMDLN PIVND+CKNDRKKILVEYFMGNLNRHNFYAFRF

Sbjct 121 ILFYVPRYLWKSWEGGRLKMLVMDLNSPIVNDECKNDRKKILVEYFMGNLNRHNFYAFRF 180

Query 181 FVCEALNFANVIGQIFFVDFFLDGEFSTYGSDVLKFTEMEPDERIDPMARVFPKVTKCTF 240

FVCEALNF NVIGQIFFVDFFLDGEFSTYGSDVLKFTEMEPDERIDPMARVFPKVTKCTF

Sbjct 181 FVCEALNFVNVIGQIFFVDFFLDGEFSTYGSDVLKFTEMEPDERIDPMARVFPKVTKCTF 240

Query 241 HKYGPSGSVQKFDGLCVLPLNIVNEKIYVFLWFWFIILSILSGISLVYRMAVIIGPKLRH 300

HKYGPSGSVQKFDGLCVLPLNIVNEKIYVFLWFWFIILSILSGISLVYRMAVIIGPKLRH

Sbjct 241 HKYGPSGSVQKFDGLCVLPLNIVNEKIYVFLWFWFIILSILSGISLVYRMAVIIGPKLRH 300

Query 301 LLLRARSRLAESEEVEAVANKCNIGDWFLLYQLGKNIDPLIYKEVIADLAREMGEHSSSK 360

LLLRARSRLAESEEVEAVANKCNIGDWFLLYQLGKNIDPLIYKEVIADLARE+GEH SSK

Sbjct 301 LLLRARSRLAESEEVEAVANKCNIGDWFLLYQLGKNIDPLIYKEVIADLARELGEHPSSK 360

Query 361 QPFEA 365

QPFEA

Sbjct 361 QPFEA 365

**Low density lipoprotein receptor (LDL)**

**>lcl|ORF15**

MGLKVTRLRDVKGSNSCAVRNGGCTQLCLNRPSDYVCRCSIEYELANDKK

TCVIPEAFLLFSRQEHIGRISIDNNEGNHNDEKIPFKDVRDAHALDVDVA

DKRIYWTDQKSKCIYRAFLNGSFVQRIIDAGLICPEGIAVDWLAHNIYWT

DSEARRIEVARLDGTSRRVLLWKGVEEPRSLVLEPKRGYMYWIESPSDSI

RRAGMDGSELQTIISGANHATGLTLDPETRRLYWATQSRPTKIESADWDG

KKRQVLINTDVDEPYAMSLYQDFVYWSDWNTGDIERVHKMTGQNRSLVHS

GMTYIRSLLIFYQNRQTGSNPCKINNGGCSHLCLAQPTRRGMICACPTHY

TLSKDNMSCLPPKNYIIYSQRNSFGRLLPNTSDCPNVPLPLSGKNIRAVE

YDPISHNIYWVEGRSHSIKHSPANSTFVKVLVGSGSQPFDIALDVIGRLL

FWTCSHSNSINVTRFDGDSIGVIDTGDSEKPRNIAVHAMKRLLFWTDVGS

QQAIIRSRIDGAERVVLAFKLEGVTALAVDQQSDMVYYAHGKRIDSMDIN

GKNKKILVSTHISQVISLAAIQGFVYWLDDKTGVERITVNGDGRRPEMQR

LPQITDIVAVWTPESKLFRNHTCLHSRTICSHICIASSEGRVRDICSCPK

NLMLLEDRQNCGALPACGPDHFTCAAPVKGDGSSSDINKDCIPASWRCDG

QSDCPDKSDEVGCPSCRPDQFSCQSGECIDKALVCDGTTNCANGHDEADC

CKRPGEFQCPINKLCISATLLCDGWDHCADGADES

**Graphical representation – ORF**


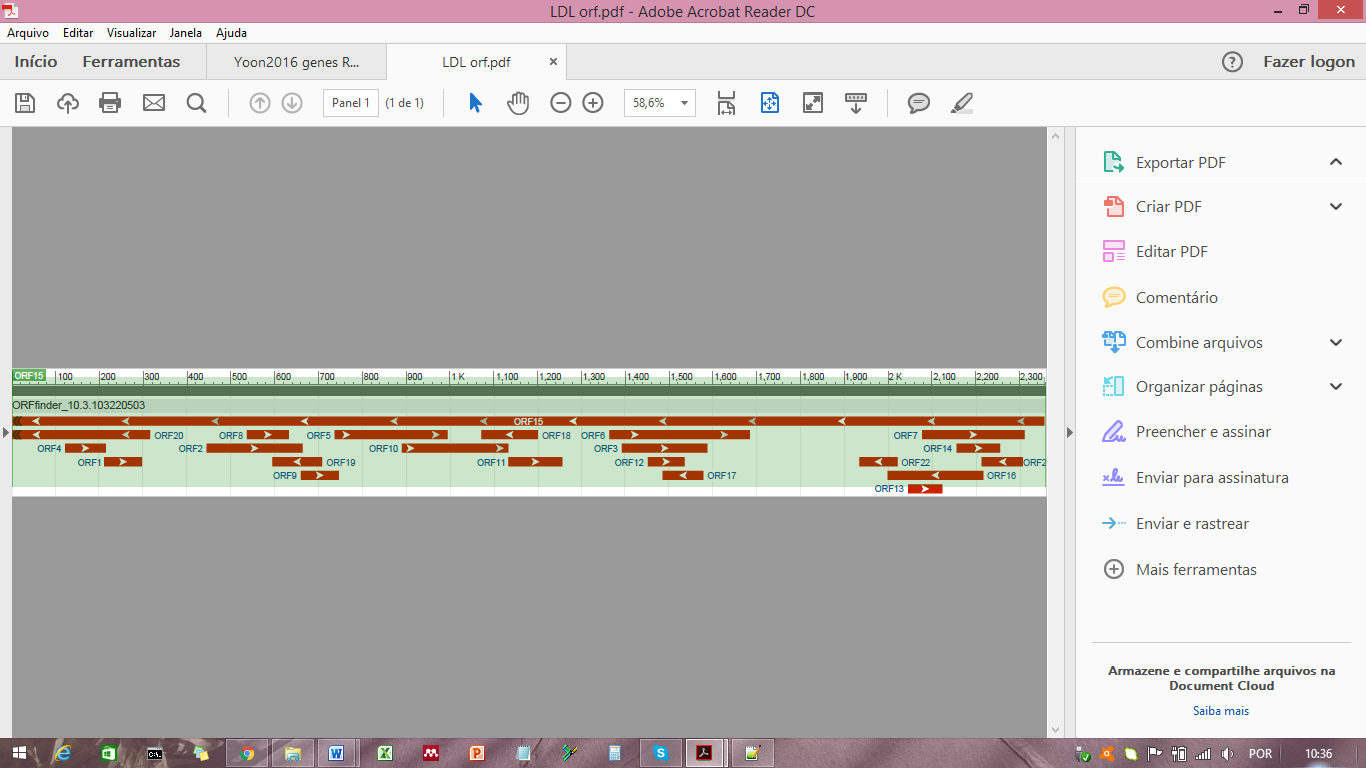


**BlastP (Non-redundant NCBI sequences)**

low-density lipoprotein receptor-related protein 6 [Ceratitis capitata]

Range 1: 751 to 1535

Score: 1584 bits(4101)

E-value: 0.0

Identities: 746/785(95%)

Query 1 MGLKVTRLRDVKGSNSCAVRNGGCTQLCLNRPSDYVCRCSIEYELANDKKTCVIPEAFLL 60

MGLKVTRLRDVKGSN+CAVRNGGC+QLCLNRPSD+VCRCSIEYELANDKKTCVIPEAFLL

Sbjct 751 MGLKVTRLRDVKGSNACAVRNGGCSQLCLNRPSDFVCRCSIEYELANDKKTCVIPEAFLL 810

Query 61 FSRQEHIGRISIDNNEGNHNDEKIPFKDVRDAHALDVDVADKRIYWTDQKSKCIYRAFLN 120

FSRQEHIGRISIDNNEGNHNDEKIPFKDVRDAHALDVDVAD+RIYWTDQKSKCIYRAFLN

Sbjct 811 FSRQEHIGRISIDNNEGNHNDEKIPFKDVRDAHALDVDVADRRIYWTDQKSKCIYRAFLN 870

Query 121 GSFVQRIIDAGLICPEGIAVDWLAHNIYWTDSEARRIEVARLDGTSRRVLLWKGVEEPRS 180

GSFVQRI+DAGLI P+GIAVDWLAHNIYWTDSEARRIEVARLDGTSRRVL+WKGVEEPRS

Sbjct 871 GSFVQRIVDAGLIFPDGIAVDWLAHNIYWTDSEARRIEVARLDGTSRRVLVWKGVEEPRS 930

Query 181 LVLEPKRGYMYWIESPSDSIRRAGMDGSELQTIISGANHATGLTLDPETRRLYWATQSRP 240

LVLEPKRGYMYWIESPSDSIRRAGMDGSELQTIISGANHATGLTLDP+TRRLYWATQSRP

Sbjct 931 LVLEPKRGYMYWIESPSDSIRRAGMDGSELQTIISGANHATGLTLDPDTRRLYWATQSRP 990

Query 241 TKIESADWDGKKRQVLINTDVDEPYAMSLYQDFVYWSDWNTGDIERVHKMTGQNRSLVHS 300

TKIESADWDGKKRQVLINTDVDEPYAMSLYQDFVYWSDWNTGDIERVHK+TG+NR++VH+

Sbjct 991 TKIESADWDGKKRQVLINTDVDEPYAMSLYQDFVYWSDWNTGDIERVHKITGENRTIVHT 1050

Query 301 GMTYIRSLLIFYQNRQTGSNPCKINNGGCSHLCLAQPTRRGMICACPTHYTLSKDNMSCL 360

GMTYIRSLLIFY+NRQ G NPCK+NNGGCSHLCLAQPTRRGMICACPTHYTL+KDNMSCL

Sbjct 1051 GMTYIRSLLIFYKNRQAGHNPCKVNNGGCSHLCLAQPTRRGMICACPTHYTLAKDNMSCL 1110

Query 361 PPKNYIIYSQRNSFGRLLPNTSDCPNVPLPLSGKNIRAVEYDPISHNIYWVEGRSHSIKH 420

PPKNY+IYSQRNSFGRLLPNTSDCPNVPLP+SGKNIRAVEYDPISHNI+WVEGRSHSIK

Sbjct 1111 PPKNYVIYSQRNSFGRLLPNTSDCPNVPLPVSGKNIRAVEYDPISHNIFWVEGRSHSIKR 1170

Query 421 SPANSTFVKVLVGSGSQPFDIALDVIGRLLFWTCSHSNSINVTRFDGDSIGVIDTGDSEK 480

S ANST+VKVLVGSGSQPFDIALDVIGRLLFWTCSHSNSINVTRFDGDSIGVIDTGDSEK

Sbjct 1171 SLANSTYVKVLVGSGSQPFDIALDVIGRLLFWTCSHSNSINVTRFDGDSIGVIDTGDSEK 1230

Query 481 PRNIAVHAMKRLLFWTDVGSQQAIIRSRIDGAERVVLAFKLEGVTALAVDQQSDMVYYAH 540

PRNIAVH M+RLLFWTDVGSQQAIIRSRIDGAERVVLAFKLEGVTALAVDQQSDMVYYAH

Sbjct 1231 PRNIAVHTMRRLLFWTDVGSQQAIIRSRIDGAERVVLAFKLEGVTALAVDQQSDMVYYAH 1290

Query 541 GKRIDSMDINGKNKKILVSTHISQVISLAAIQGFVYWLDDKTGVERITVNGDGRRPEMQR 600

GKRIDSMDINGKNKKILVSTHISQVISLAA+QGFVYWLDDKTGVERITVNGDGRRPEMQR

Sbjct 1291 GKRIDSMDINGKNKKILVSTHISQVISLAALQGFVYWLDDKTGVERITVNGDGRRPEMQR 1350

Query 601 LPQITDIVAVWTPESKLFRNHTCLHSRTICSHICIASSEGRVRDICSCPKNLMLLEDRQN 660

LPQITDIVAVWTPESKL+RNHTCLHSRT CSHICIASSEGR RDICSCPKNLMLLEDRQN

Sbjct 1351 LPQITDIVAVWTPESKLYRNHTCLHSRTKCSHICIASSEGRARDICSCPKNLMLLEDRQN 1410

Query 661 CGALPACGPDHFTCAAPVKGDGSSSDINKDCIPASWRCDGQSDCPDKSDEVGCPSCRPDQ 720

CGALPACGPDHFTCAAPV+GDGSSSD NKDCIPASWRCDGQSDCPDKSDEVGCP+CRPD

Sbjct 1411 CGALPACGPDHFTCAAPVRGDGSSSDTNKDCIPASWRCDGQSDCPDKSDEVGCPTCRPDL 1470

Query 721 FSCQSGECIDKALVCDGTTNCANGHDEADCCKRPGEFQCPINKLCISATLLCDGWDHCAD 780

FSCQSGECIDKALVCDGTTNCANGHDEADCCKRPGEFQCPINK+CISATL+CDGW++CAD

Sbjct 1471 FSCQSGECIDKALVCDGTTNCANGHDEADCCKRPGEFQCPINKVCISATLVCDGWENCAD 1530

Query 781 GADES 785

GADES

Sbjct 1531 GADES 1535

**TRF2**

**>lcl|ORF13**

MFKICLLTIFLCLLQEKCIDSQHHYENEKQVKNLIWCTKSLEEQYKCQNL

TVAIERDRAIFDDAFLNLTCFLGYSADECIHHIDRDKAHVTTLDAGDVFS

AGRYKSLVPIMQEKFVGNLTNYHAVAVIKKDTLQDVYSLRDLRNKRACFP

WVGSMAGWIVPIYTLQRDGGMEIVDCNNQVKTAANYFNNSCAVYSLINKY

NPIGDNSDKLCRLCTGKIPGGRCSARDPYYGYDGAFRCLLEAGEVAFLRD

LTVTEMLQTDEFKSLSPDRFELLCRDGRRMPVTEYRHCNWGIIPSDAIVT

SSARNSLERKKFQQFLRRIIELYSDALRDEAEQQNLRGNTEYGNNNFNYN

NNNNKDQYGNPYGVNINNPYDTSNTYNRNSYDGGNSGFRNDRLDSSFTTE

RNYGEGTNESILYEKFRIFESRRYGKANLMFQDSARSLSPIAEDDQSFSK

YLQNTMQYIYGIRECPVPSMTLCVTSDPELEKCIKMKTALKAQLLKPELI

CKKMHSHINCMQWIQSGKADIAVFDAGDVYTGGLNYELVPFMSEVYNLDE

PEYYVVAVAKEEDPDTELTYLKGKYTCHTGINTAAGWTYPMAFLISNGWI

RPYGCDSVRAAAEYFTKSCVPGAISNEYNTGVPYDSMCDLCHGTSYRYCR

RDASEDYYGHTGAFRCLVEGGGHVAFMKHTTVMESTGGKRKEWWARNALN

DDFELLCTDGTRAELQDYRKCNLGKVKANAIVTRGGVNYNDTQVNAYINL

LTYALQLYGRKDVDSFTFSMFSSPMGFYDLIFQDATRQLRVIPPEQRRYD

IYLGSNFMRARRITDCYAGAGQLVASLPLFLAISAVLLRV

**Graphical representation – ORF**


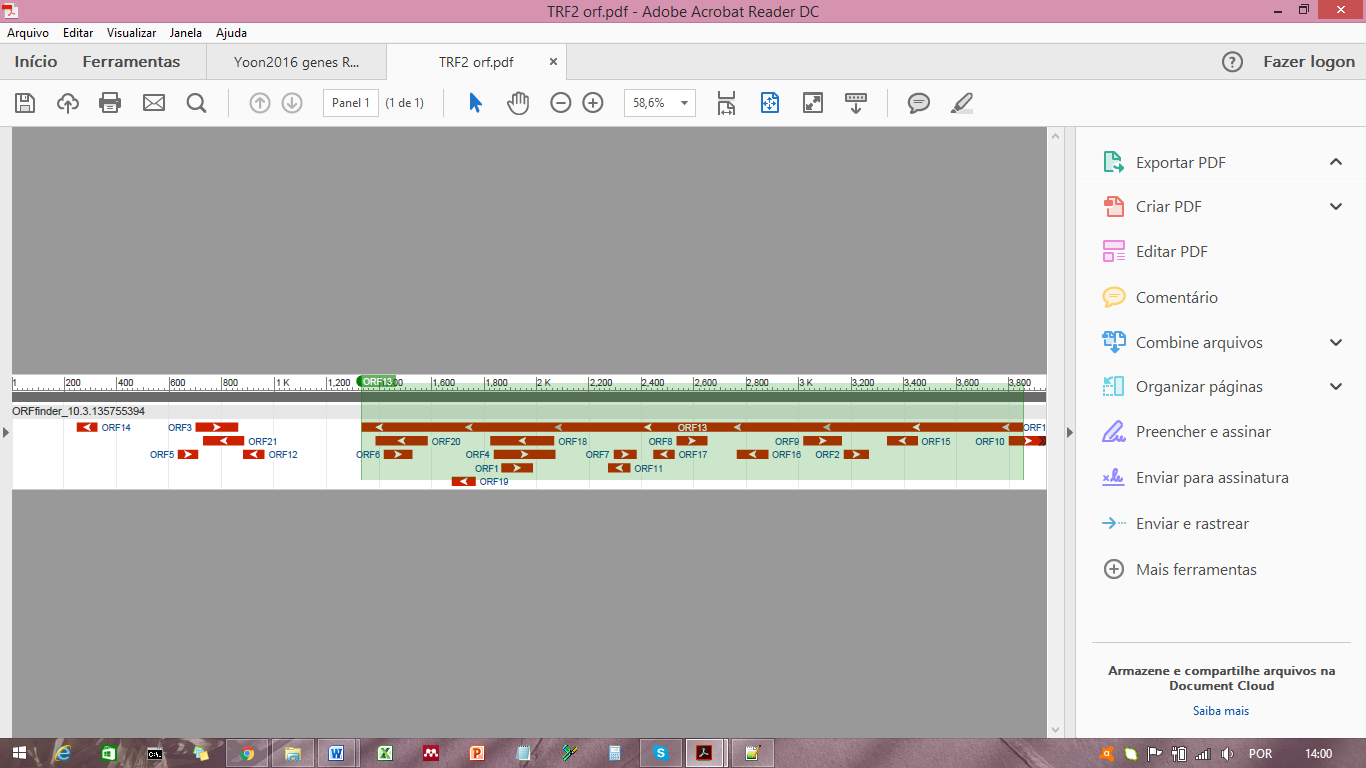


**BlastP (Non-redundant NCBI sequences)**

transferrin 2 [Bactrocera dorsalis]

Sequence ID: [AGL91667.1](https://www.ncbi.nlm.nih.gov/protein/500866223?report=genbank&log$=protalign&blast_rank=1&RID=X7FJF5DW014) Length: 841 Number of Matches: 1

Range 1: 1 to 839

Score: 1564 bits(4050)

E-value: 0.0

Identities: 746/841(89%)

Query 1 MFKICLLTIFLCLLQEKCIDSQHHYENEKQVKNLIWCTKSLEEQYKCQNLTVAIERDRAI 60

M +ICLL+IF+ LLQ K ID+QHHYENE+QVKN+IWCTKSLEEQYKCQNLTVAIERDRA+

Sbjct 1 MLRICLLSIFIFLLQGKDIDAQHHYENERQVKNIIWCTKSLEEQYKCQNLTVAIERDRAL 60

Query 61 FDDAFLNLTCFLGYSADECIHHIDRDKAHVTTLDAGDVFSAGRYKSLVPIMQEKFVGNLT 120

FDDA+LNLTCFLGYSADECIHHID +KAHVTTLDAGDVF+AGRY SLVPIMQEKF G L

Sbjct 61 FDDAYLNLTCFLGYSADECIHHIDHEKAHVTTLDAGDVFTAGRYNSLVPIMQEKFAGGLA 120

Query 121 NYHAVAVIKKDTLQDVYSLRDLRNKRACFPWVGSMAGWIVPIYTLQRDGGMEIVDCNNQV 180

NYHAVAV+KKDTLQDVYSLRDLRNKRACFPWVGSMAGWIVPIYTLQR+GGME+VDCNNQV

Sbjct 121 NYHAVAVVKKDTLQDVYSLRDLRNKRACFPWVGSMAGWIVPIYTLQREGGMEVVDCNNQV 180

Query 181 KTAANYFNNSCAVYSLINKYNPIGDNSDKLCRLCTGKIPGGRCSARDPYYGYDGAFRCLL 240

KTAANYFNNSCAVYSLINKYNPIGDNSDKLC LCTGKIPGGRCSA+DPY+GYDGAFRCLL

Sbjct 181 KTAANYFNNSCAVYSLINKYNPIGDNSDKLCTLCTGKIPGGRCSAKDPYFGYDGAFRCLL 240

Query 241 EAGEVAFLRDLTVTEMLQTDEFKSLSPDRFELLCRDGRRMPVTEYRHCNWGIIPSDAIVT 300

EAGEVAFLRD TVTEMLQT+EF SLSPDRFELLCRDGRR+PV++YR CNWG+IPSDA+VT

Sbjct 241 EAGEVAFLRDSTVTEMLQTNEFSSLSPDRFELLCRDGRRVPVSKYRQCNWGVIPSDAVVT 300

Query 301 SSARNSLERKKFQQFLRRIIELYSDALRDEAEQQNLRGNTEYGNNNFNYNNNNNKDQYGN 360

SSAR S ERKKFQQFLRRII LYSDALR+EA +QN R N E GNN N NNKDQYGN

Sbjct 301 SSARTSFERKKFQQFLRRIIGLYSDALREEANEQNKRLNGEAGNNF--NYNVNNKDQYGN 358

Query 361 PYGVNINNPYDTSNTYNRN---SYDGGNSGFRNDRLDSSFTTERNYGEGTNESILYEKFR 417

YGVN NNPYDTSNTYNRN +YDGGNSGFRNDRLDSSFTT+RNY EGTNE+ILYEKFR

Sbjct 359 TYGVNSNNPYDTSNTYNRNPYDNYDGGNSGFRNDRLDSSFTTDRNYPEGTNETILYEKFR 418

Query 418 IFESRRYGKANLMFQDSARSLSPIAEDDQSFSKYLQNTMQYIYGIRECPVPSMTLCVTSD 477

IFESRR+GKANLMFQDSARSL I EDDQSFSK+LQ+T YIYGIR+CPVPSMTLCVTSD

Sbjct 419 IFESRRHGKANLMFQDSARSLILIPEDDQSFSKHLQDTTTYIYGIRDCPVPSMTLCVTSD 478

Query 478 PELEKCIKMKTALKAQLLKPELICKKMHSHINCMQWIQSGKADIAVFDAGDVYTGGLNYE 537

PELEKCIKMK ALKAQ+LKPELICKK HSHINCMQWIQSGKADIAVFDAGDVYTGGLNY+

Sbjct 479 PELEKCIKMKIALKAQILKPELICKKEHSHINCMQWIQSGKADIAVFDAGDVYTGGLNYD 538

Query 538 LVPFMSEVYNLDEPEYYVVAVAKEEDPDTELTYLKGKYTCHTGINTAAGWTYPMAFLISN 597

L+PFMSEVYNL EPEYYVVAVAKEEDPDTELTYLKGKYTCHTGINTAAGWTYPMAFLISN

Sbjct 539 LIPFMSEVYNLGEPEYYVVAVAKEEDPDTELTYLKGKYTCHTGINTAAGWTYPMAFLISN 598

Query 598 GWIRPYGCDSVRAAAEYFTKSCVPGAISNEYNTGVPYDSMCDLCHGTSYRYCRRDASEDY 657

GWIRPYGCDS+RAAAEYFTKSC+PGAISNEYNTGVPYDSMCDLCHGTSYRYCRRDASEDY

Sbjct 599 GWIRPYGCDSIRAAAEYFTKSCIPGAISNEYNTGVPYDSMCDLCHGTSYRYCRRDASEDY 658

Query 658 YGHTGAFRCLVEGGGHVAFMKHTTVMESTGGKRKEWWARNALNDDFELLCTDGTRAELQD 717

YGHTGAFRCLVEGGGHVAFMKHTTVMESTGGKRKEWWARN LNDDFELLCTDGTRAEL D

Sbjct 659 YGHTGAFRCLVEGGGHVAFMKHTTVMESTGGKRKEWWARNTLNDDFELLCTDGTRAELHD 718

Query 718 YRKCNLGKVKANAIVTRGGVNYNDTQVNAYINLLTYALQLYGRKDVDSFTFSMFSSPMGF 777

Y+KCNLGKVKANAIVTRGGVNYNDTQ+NAYINLLTYA QLYGRK+ D+F+FSMFSSPMGF

Sbjct 719 YKKCNLGKVKANAIVTRGGVNYNDTQINAYINLLTYAQQLYGRKNTDTFSFSMFSSPMGF 778

Query 778 YDLIFQDATRQLRVIPPEQRRYDIYLGSNFMRARRITDCYAGAGQLVASLPLFLAISAVL 837

YDLIFQDATRQLRVIPP QRRYDIYLGSNFMRARRITDCYAGA QL+ S+PLF + A L

Sbjct 779 YDLIFQDATRQLRVIPPNQRRYDIYLGSNFMRARRITDCYAGAAQLMVSVPLFFMVFAFL 838

Query 838 L 838

L

Sbjct 839 L 839

**Vha16**

**>lcl|ORF18**

MSDSQSDNPIYGPFFGVMGAASAIIFSALGAAYGTAKSGTGIAAMSVMRP

ELIMKSIIPVVMAGIIAIYGLVVAVLIAGALEQPSDYSLFKGFIHLGAGL

SVGFSGLAAGFAIGIVGDAGVRGTAQQPRLFVGMILILIFAEVLGLYGLI

VAIYLYTK

**Graphical representation – ORF**


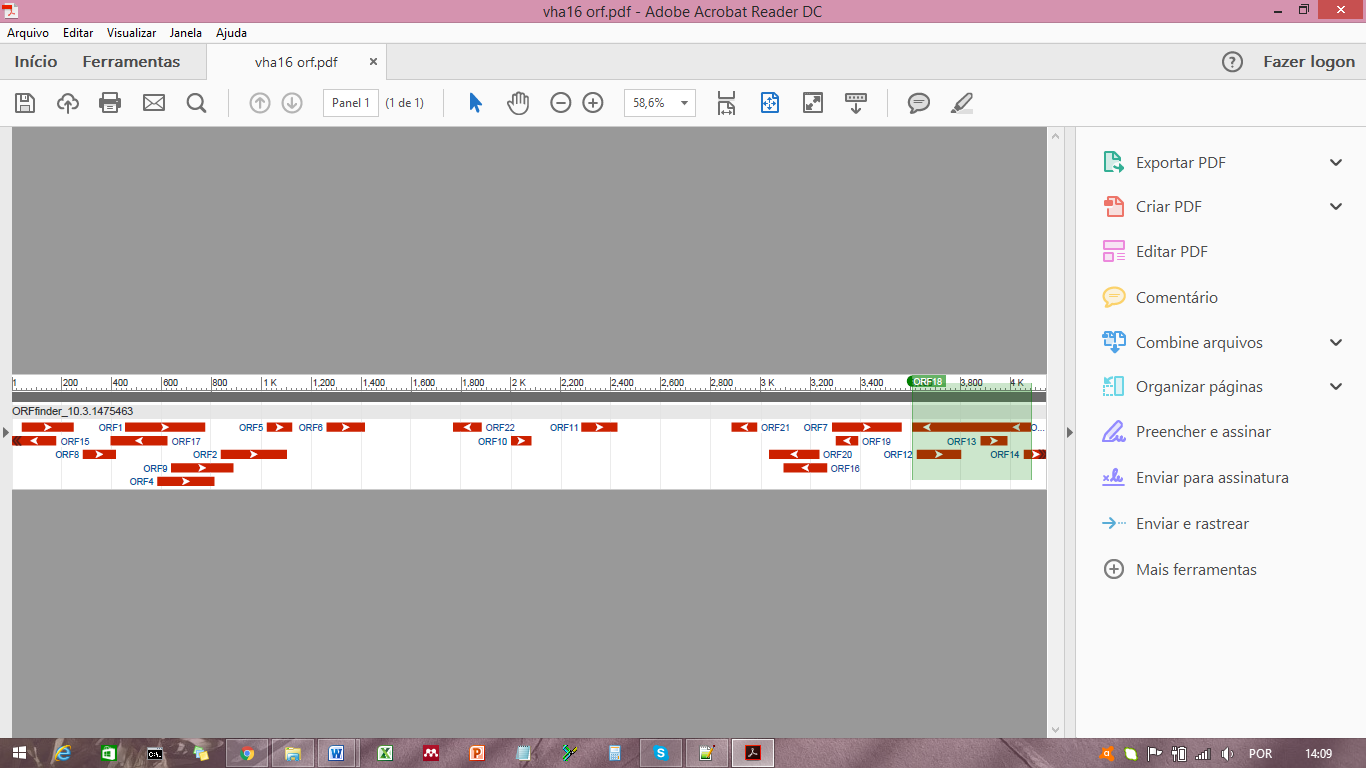


**BlastP (Non-redundant NCBI sequences)**

PREDICTED: V-type proton ATPase 16 kDa proteolipid subunit [Bactrocera dorsalis]

Sequence ID: [XP_011209070.1](https://www.ncbi.nlm.nih.gov/protein/751798321?report=genbank&log$=protalign&blast_rank=1&RID=X7H15MHE014)Length: 158Number of Matches: 1

Range 1: 1 to 158

Score: 293 bits(749)

E-value: 7e-100

Identities: 153/158(97%)

Query 1 MSDSQSDNPIYGPFFGVMGAASAIIFSALGAAYGTAKSGTGIAAMSVMRPELIMKSIIPV 60

MSD+QSDNPIYGPFFGVMGAASAIIFSALGAAYGTAKSGTGIAAMSVMRPELIMKSIIPV

Sbjct 1 MSDAQSDNPIYGPFFGVMGAASAIIFSALGAAYGTAKSGTGIAAMSVMRPELIMKSIIPV 60

Query 61 VMAGIIAIYGLVVAVLIAGALEQPSDYSLFKGFIHLGAGLSVGFSGLAAGFAIGIVGDAG 120

VMAGIIAIYGLVVAVLIAGALE+PS Y+L+KGFIHLGAGLSVGFSGLAAGFAIGIVGDAG

Sbjct 61 VMAGIIAIYGLVVAVLIAGALEEPSSYTLYKGFIHLGAGLSVGFSGLAAGFAIGIVGDAG 120

Query 121 VRGTAQQPRLFVGMILILIFAEVLGLYGLIVAIYLYTK 158

VRGTAQQPRLFVGMILILIFAEVLGLYGLIVAIYLYTK

Sbjct 121 VRGTAQQPRLFVGMILILIFAEVLGLYGLIVAIYLYTK 158

**VhaSFD**

**>lcl|ORF3**

MSSRILAKLACWGHELMPKSDLNFYLQFLKDQLTVNANEYIQSVARCLQM

MLRIDEYRFAFVSVDGISTLISILSSRVNFQVQYQLVFCLWVLTFNPLLA

TKMNKFSVIPILADILNDCAKEKVTRIILAVFRNLIEKPTDPQVAKEHCI

AMVQCKVLKQLSILEQRRFDDEDISADVEFLTEKLQNSVQDLSSFDEYAT

ELRSARLEWSPVHKSAKFWRENAHRLNEKNYELLRILVHLLETSKDHIIL

SVACFDIGEYVRHYPRGKHVLEQLGGKQIVMQLLAHDDPNVRYEALLAVQ

KLMVHNWEYLGKHLEKESENQPQKGGSAPISGKA

**Graphical representation – ORF**


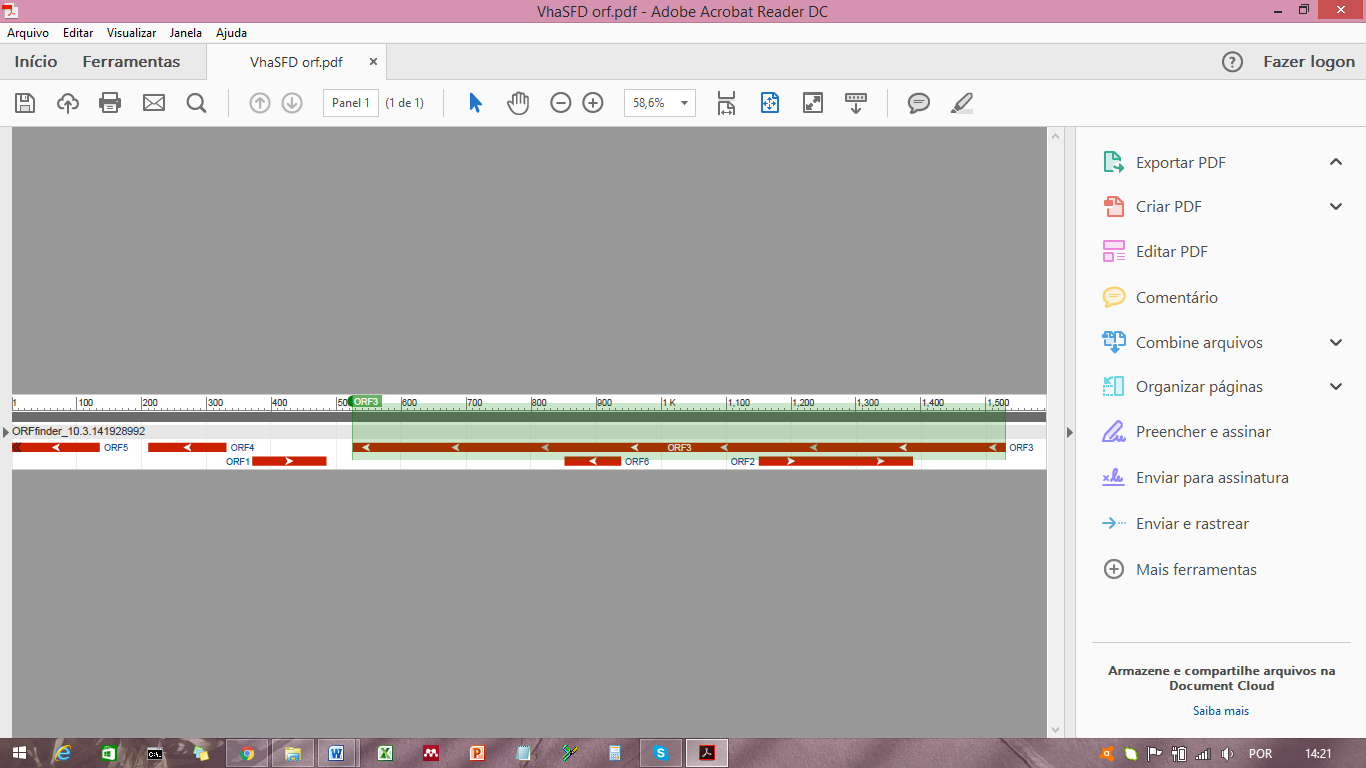


**BlastP (Non-redundant NCBI sequences)**

PREDICTED: V-type proton ATPase subunit H isoform X2 [Zeugodacus cucurbitae]

Sequence ID: [XP_011189552.1](https://www.ncbi.nlm.nih.gov/protein/751468806?report=genbank&log$=protalign&blast_rank=1&RID=X7HRF1CG01R) Length: 473 Number of Matches: 1

Range 1: 140 to 473

687 bits(1774)

0.0

330/334(99%)

Query 1 MSSRILAKLACWGHELMPKSDLNFYLQFLKDQLTVNANEYIQSVARCLQMMLRIDEYRFA 60

MSSRILAKLACWG ELMPKSDLNFYLQFLKDQLTVNANEYIQSVARCLQMMLRIDEYRFA

Sbjct 140 MSSRILAKLACWGRELMPKSDLNFYLQFLKDQLTVNANEYIQSVARCLQMMLRIDEYRFA 199

Query 61 FVSVDGISTLISILSSRVNFQVQYQLVFCLWVLTFNPLLATKMNKFSVIPILADILNDCA 120

FVSVDGISTLISILSSRVNFQVQYQLVFCLWVLTFNPLLATKMNKFSVIPILADIL+DCA

Sbjct 200 FVSVDGISTLISILSSRVNFQVQYQLVFCLWVLTFNPLLATKMNKFSVIPILADILSDCA 259

Query 121 KEKVTRIILAVFRNLIEKPTDPQVAKEHCIAMVQCKVLKQLSILEQRRFDDEDISADVEF 180

KEKVTRIILAVFRNLIEKPTDPQVAKEHCIAMVQCKVLKQLSILEQRRFDDEDISADVEF

Sbjct 260 KEKVTRIILAVFRNLIEKPTDPQVAKEHCIAMVQCKVLKQLSILEQRRFDDEDISADVEF 319

Query 181 LTEKLQNSVQDLSSFDEYATELRSARLEWSPVHKSAKFWRENAHRLNEKNYELLRILVHL 240

LTEKLQNSVQDLSSFDEYATELRSARLEWSPVHKSAKFWRENAHRLNEKNYELLRILVHL

Sbjct 320 LTEKLQNSVQDLSSFDEYATELRSARLEWSPVHKSAKFWRENAHRLNEKNYELLRILVHL 379

Query 241 LETSKDHIILSVACFDIGEYVRHYPRGKHVLEQLGGKQIVMQLLAHDDPNVRYEALLAVQ 300

LETSKDHIILSVACFDIGEYVRHYPRGKHVLEQLGGKQIVMQLLAHDDPNVRYEALLAVQ

Sbjct 380 LETSKDHIILSVACFDIGEYVRHYPRGKHVLEQLGGKQIVMQLLAHDDPNVRYEALLAVQ 439

Query 301 KLMVHNWEYLGKHLEKESENQPQKGGSAPISGKA 334

KLMVHNWEYLGK LEKE+ENQPQKGGSAPISGKA

Sbjct 440 KLMVHNWEYLGKQLEKENENQPQKGGSAPISGKA 473

**Small Rab GTPases (Rab7)**

**>lcl|ORF11**

MASRKKSLLKVIILGDSSVGKTSLMNQYINKRFSNQYKATIGADFCTKEI

IADDRVVTMQIWDTAGQERFQSLGVAFYRGADCCVLVYDVTSPNSFKNLD

SWRDEFLIQASPRDPDHFPFVVLGNKVDMDNRQVSTRRAQQWCQSKNDLP

YFETSAKDGTNVELAFQTIAKNALAQEAEVELYNEFPDQIRLNTDRNNRN

GNADNCQC

**Graphical representation – ORF**


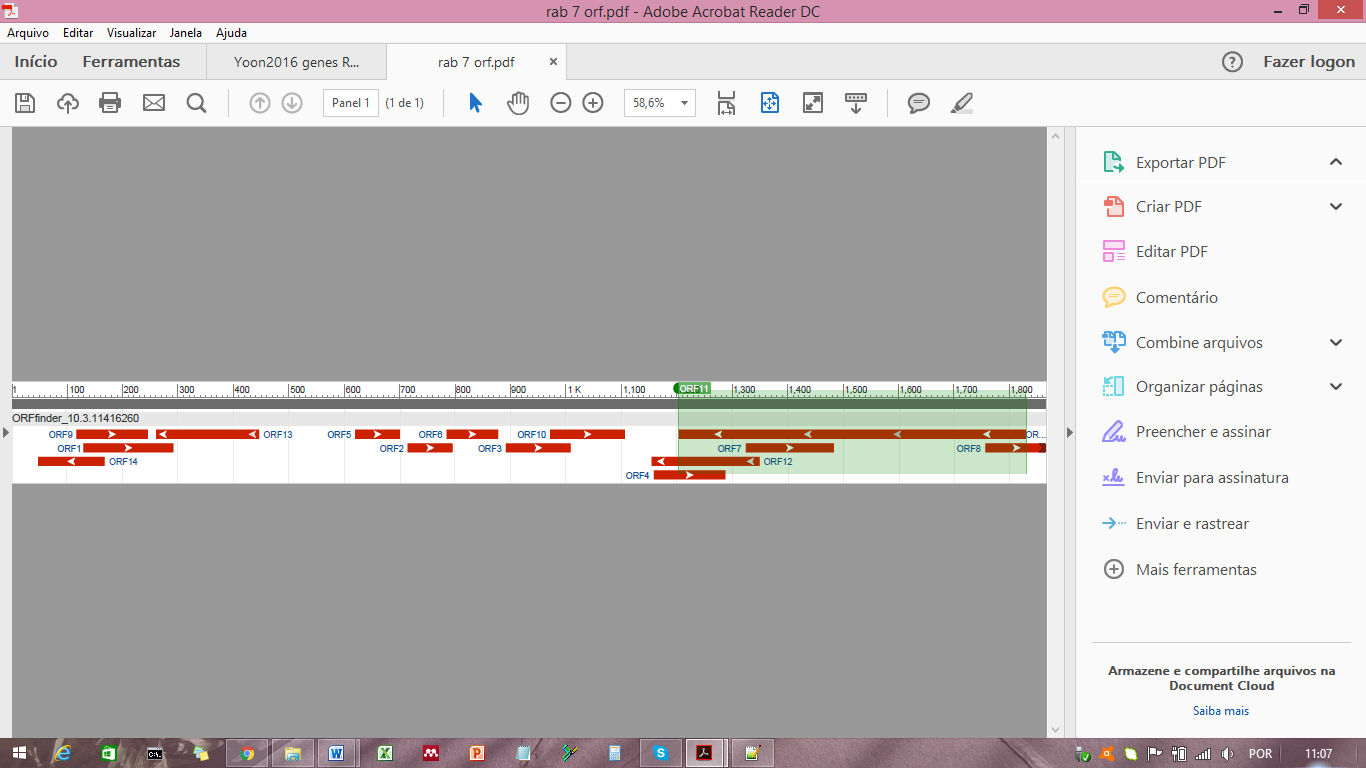


**BlastP (Non-redundant NCBI sequences)**

PREDICTED: ras-related protein Rab-7a [Rhagoletis zephyria]

Sequence ID: [XP_017472236.1](https://www.ncbi.nlm.nih.gov/protein/1048023318?report=genbank&log$=protalign&blast_rank=1&RID=X76AVKUU014) Length: 208 Number of Matches: 1

Range 1: 1 to 208

Score: 433 bits(1113)

E-value: 8e-154

Identities: 207/208(99%)

Query 1 MASRKKSLLKVIILGDSSVGKTSLMNQYINKRFSNQYKATIGADFCTKEIIADDRVVTMQ 60

MASRKKSLLKVIILGDSSVGKTSLMNQYINKRFSNQYKATIGADFCTKEIIADDRVVTMQ

Sbjct 1 MASRKKSLLKVIILGDSSVGKTSLMNQYINKRFSNQYKATIGADFCTKEIIADDRVVTMQ 60

Query 61 IWDTAGQERFQSLGVAFYRGADCCVLVYDVTSPNSFKNLDSWRDEFLIQASPRDPDHFPF 120

IWDTAGQERFQSLGVAFYRGADCCVLVYDVTSPNSFKNLDSWRDEFLIQASPRDPDHFPF

Sbjct 61 IWDTAGQERFQSLGVAFYRGADCCVLVYDVTSPNSFKNLDSWRDEFLIQASPRDPDHFPF 120

Query 121 VVLGNKVDMDNRQVSTRRAQQWCQSKNDLPYFETSAKDGTNVELAFQTIAKNALAQEAEV 180

VVLGNKVDMDNRQVSTRRAQQWCQSKNDLPYFETSAKDGTNVELAFQTIAKNALAQEAEV

Sbjct 121 VVLGNKVDMDNRQVSTRRAQQWCQSKNDLPYFETSAKDGTNVELAFQTIAKNALAQEAEV 180

Query 181 ELYNEFPDQIRLNTDRNNRNGNADNCQC 208

ELYNEFPDQIRLNTDRN+RNGNADNCQC

Sbjct 181 ELYNEFPDQIRLNTDRNSRNGNADNCQC 208

**Ligth**

**>lcl|ORF2**

MAEQEHVNNSDTETESTEDEIEPKFKYQRIANDLRKILNSDVVTCSAVHP

KFLMFGTFLGRVYIFDHQGNSVTSHLSDGPNDFSHTVAVNHIDVDSKAEY

VATCSDDGKVNITGLFSDENNQNLNLGKSIKAVALDPDPKTSAGKRFVVG

DDKLTLYEKNFLKKLKTTVLSSAEGYVLSVCWNGPFVAWASYLGVRVYDL

NEKCSLGLMKWEEPVNARLENFRCNFRWSNATTLLIGWVDTIRICVIRRR

NSIEVASRELPGYIVDPISTFQTTFYVSGLAPLTSNQLVVLGCPKEKDAE

RKSLRPVLCVMEYKLNTSEEICTDSLSLRGYQEYTVNDYSLGCIIEENRY

FIVAPKDIVVASLYETDDRVKWLVDHRKFEEAMEVISTHGGSWSLLSVAK

LYINHLLAMKQYDDAAKLCLRVLGNKKSLWEEEVFKFVKCQQLRSVSAYL

PTSDDCKLDPHVYEIVLYEYLKFDAKGFLNLIKEWPSHLYNCKAVINAIH

DNFRKQNANELLEALAILYLHQRDYESALRMYLKLQNSDVFEMIRRYNLY

DAIHKMIIPLIQLDRERAFKILFEKNKIPPEIVVQQLEQNQEYLYWYLDA

LDKVNKSGKYHWKLVNLYAKYEPEKLLPFLKRSNHYPMQEALDICKRELF

YPEMVYLLGQMGNTIEALNIIIEKIKDIEMAIEFCKERNDSDLWNILIDE

SVKEPAIVLKLLDGIVDYVDPVAVVEKIKLGQKIPGLRDAVVKLLWDYRL

QVEIRKSAQKLQLEFYYDQHARVVNTQNRGRYISSSERCLKCNRSVLSMN

ENIAPLNDIVVFLCGHVYHTNCVPGGIGNEHCEFCNPSDFNQEDDFALLL

QYSRK

**Graphical representation – ORF**


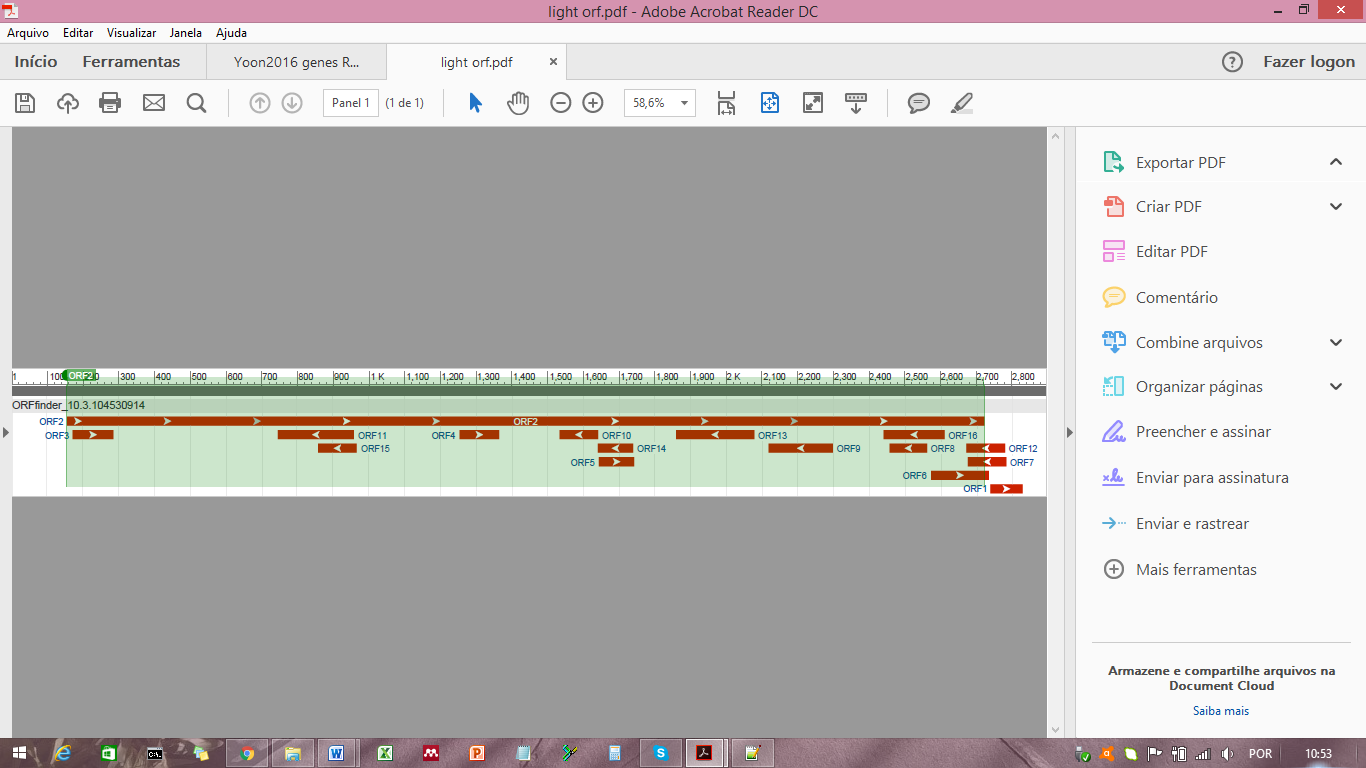


**BlastP (Non-redundant NCBI sequences)**

PREDICTED: vacuolar protein sorting-associated protein 41 homolog [Rhagoletis zephyria]

Sequence ID: [XP_017474853.1](https://www.ncbi.nlm.nih.gov/protein/1048028252?report=genbank&log$=protalign&blast_rank=1&RID=X75AFTYF014) Length: 851 Number of Matches: 1

Score: 1656 bits(4289)

E-value: 0.0

Identities: 799/851(94%)

Query 1 MAEQEHVNNSDTETESTEDEIEPKFKYQRIANDLRKILNSDVVTCSAVHPKFLMFGTFLG 60

MAEQE VNNSDTETES EDEIEPKFKYQRIANDLRKILNSDVVTCSAVHPKFLMFGTFLG

Sbjct 1 MAEQEPVNNSDTETESAEDEIEPKFKYQRIANDLRKILNSDVVTCSAVHPKFLMFGTFLG 60

Query 61 RVYIFDHQGNSVTSHLSDGPNDFSHTVAVNHIDVDSKAEYVATCSDDGKVNITGLFSDEN 120

R+YIFDHQGNSVTSHLSD P DFSHTVAVNH+DVD+KAEYVATCSDDGKVNITGLFSDEN

Sbjct 61 RIYIFDHQGNSVTSHLSDAPTDFSHTVAVNHMDVDAKAEYVATCSDDGKVNITGLFSDEN 120

Query 121 NQNLNLGKSIKAVALDPDPKTSAGKRFVVGDDKLTLYEKNFLKKLKTTVLSSAEGYVLSV 180

NQNLN KSIKA+ALDPDPKT AGKRFVVGDDKLTLYEKNFLKKLKTTVLSSAEGYVLSV

Sbjct 121 NQNLNFAKSIKAIALDPDPKTPAGKRFVVGDDKLTLYEKNFLKKLKTTVLSSAEGYVLSV 180

Query 181 CWNGPFVAWASYLGVRVYDLNEKCSLGLMKWEEPVNARLENFRCNFRWSNATTLLIGWVD 240

CWNG F+AWASYLGVRVYDLNEKCSLGLMKWEEP N+RLENFRCNFRWSNATTLLIGWVD

Sbjct 181 CWNGTFIAWASYLGVRVYDLNEKCSLGLMKWEEPANSRLENFRCNFRWSNATTLLIGWVD 240

Query 241 TIRICVIRRRNSIEVASRELPGYIVDPISTFQTTFYVSGLAPLTSNQLVVLGCPKEKDAE 300

TIRICVIRRRNSIEVASR+LPG+IVDPISTFQTTF++SGLAPLTSNQLVVLGCPKEKDAE

Sbjct 241 TIRICVIRRRNSIEVASRDLPGFIVDPISTFQTTFHISGLAPLTSNQLVVLGCPKEKDAE 300

Query 301 RKSLRPVLCVMEYKLNTSEEICTDSLSLRGYQEYTVNDYSLGCIIEENRYFIVAPKDIVV 360

RKSLRPVLCVMEYKLN+SEEICTDSLSLRGYQEYTVNDYSLGCIIEENRYFIVAPKDIVV

Sbjct 301 RKSLRPVLCVMEYKLNSSEEICTDSLSLRGYQEYTVNDYSLGCIIEENRYFIVAPKDIVV 360

Query 361 ASLYETDDRVKWLVDHRKFEEAMEVISTHGGSWSLLSVAKLYINHLLAMKQYDDAAKLCL 420

ASLYETDDRVKWLVDHRKFEEAMEVISTHGGSWSLLSVAKLYINHLLAM ++DDAAKLCL

Sbjct 361 ASLYETDDRVKWLVDHRKFEEAMEVISTHGGSWSLLSVAKLYINHLLAMNKFDDAAKLCL 420

Query 421 RVLGNKKSLWEEEVFKFVKCQQLRSVSAYLPTSDDCKLDPHVYEIVLYEYLKFDAKGFLN 480

RVLGNKKSLWEEEVFKFV+CQQLRSVSAYLPTSDDCKLDPHVYE+VLYEYLKFD KGFLN

Sbjct 421 RVLGNKKSLWEEEVFKFVRCQQLRSVSAYLPTSDDCKLDPHVYEMVLYEYLKFDVKGFLN 480

Query 481 LIKEWPSHLYNCKAVINAIHDNFRKQNANELLEALAILYLHQRDYESALRMYLKLQNSDV 540

LIKEWPSHLYNC AVINAIHDNFRKQNANELLEALAILYLHQRDYESALRMYLKLQNSDV

Sbjct 481 LIKEWPSHLYNCTAVINAIHDNFRKQNANELLEALAILYLHQRDYESALRMYLKLQNSDV 540

Query 541 FEMIRRYNLYDAIHKMIIPLIQLDRERAFKILFEKNKIPPEIVVQQLEQNQEYLYWYLDA 600

FEMIRRYNLYDAIHKMIIPL+QLDRERAFK+LFE+NKI PEIVVQQLEQNQEYLYWYLDA

Sbjct 541 FEMIRRYNLYDAIHKMIIPLLQLDRERAFKVLFERNKISPEIVVQQLEQNQEYLYWYLDA 600

Query 601 LDKVNKSGKYHWKLVNLYAKYEPEKLLPFLKRSNHYPMQEALDICKRELFYPEMVYLLGQ 660

LDKV+KSGKYHWKLV LYAKYEPEKLLPFLKRSNHYPMQEALD+CKRELFYPEMVYLLGQ

Sbjct 601 LDKVDKSGKYHWKLVKLYAKYEPEKLLPFLKRSNHYPMQEALDVCKRELFYPEMVYLLGQ 660

Query 661 MGNTIEALNIIIEKIKDIEMAIEFCKERNDSDLWNILIDESVKEPAIVLKLLDGIVDYVD 720

MGNT EALNIIIEKIKDIEMAIEFCKE NDSDLWNILIDESVKEPAIVLKLLDGIVDYVD

Sbjct 661 MGNTTEALNIIIEKIKDIEMAIEFCKEHNDSDLWNILIDESVKEPAIVLKLLDGIVDYVD 720

Query 721 PVAVVEKIKLGQKIPGLRDAVVKLLWDYRLQVEIRKSAQKLQLEFYYDQHARVVNTQNRG 780

PVAVVEKIKLGQKIPGLRDAVVKLLWDYRLQVEIRK+AQ++QL++YYDQHARVVNTQNRG

Sbjct 721 PVAVVEKIKLGQKIPGLRDAVVKLLWDYRLQVEIRKTAQEIQLQYYYDQHARVVNTQNRG 780

Query 781 RYISSSERCLKCNRSVLSMNENIAPLNDIVVFLCGHVYHTNCVPGGIGNEHCEFCNPSDF 840

R +SSSE CLKCNRSVLSMN NIAPLND+VVFLCGHVYH+ CVPGGI +EHCEFCNPSDF

Sbjct 781 RCVSSSEHCLKCNRSVLSMNANIAPLNDVVVFLCGHVYHSKCVPGGIEDEHCEFCNPSDF 840

Query 841 NQEDDFALLLQ 851

N++DDFALLLQ

Sbjct 841 NKDDDFALLLQ 851

**Idlcp (Exocytocis)**

**>lcl|ORF4**

MDEVDVATVDQYQTLVRYNNPVLVVKHPDKKGVPTEIELKRPLTAGALLD

TKRETEEILNSILPPRCWEEDGQLWQQTVSSTPATRQDVINLQEMLDTRL

QQTQARETGICPIRRELYTQCFDEIIRQVTINCSERGLLLLRIRDEIAMS

MEAYETLYCSSVAFGMRKALQAHEEKEMLRDRVKTLETEKESLEEIINDM

KIKQEQAERRNAELRASEEKKYAEEVAFLKKTNTQLKAQLEGITAPKK

**Graphical representation – ORF**


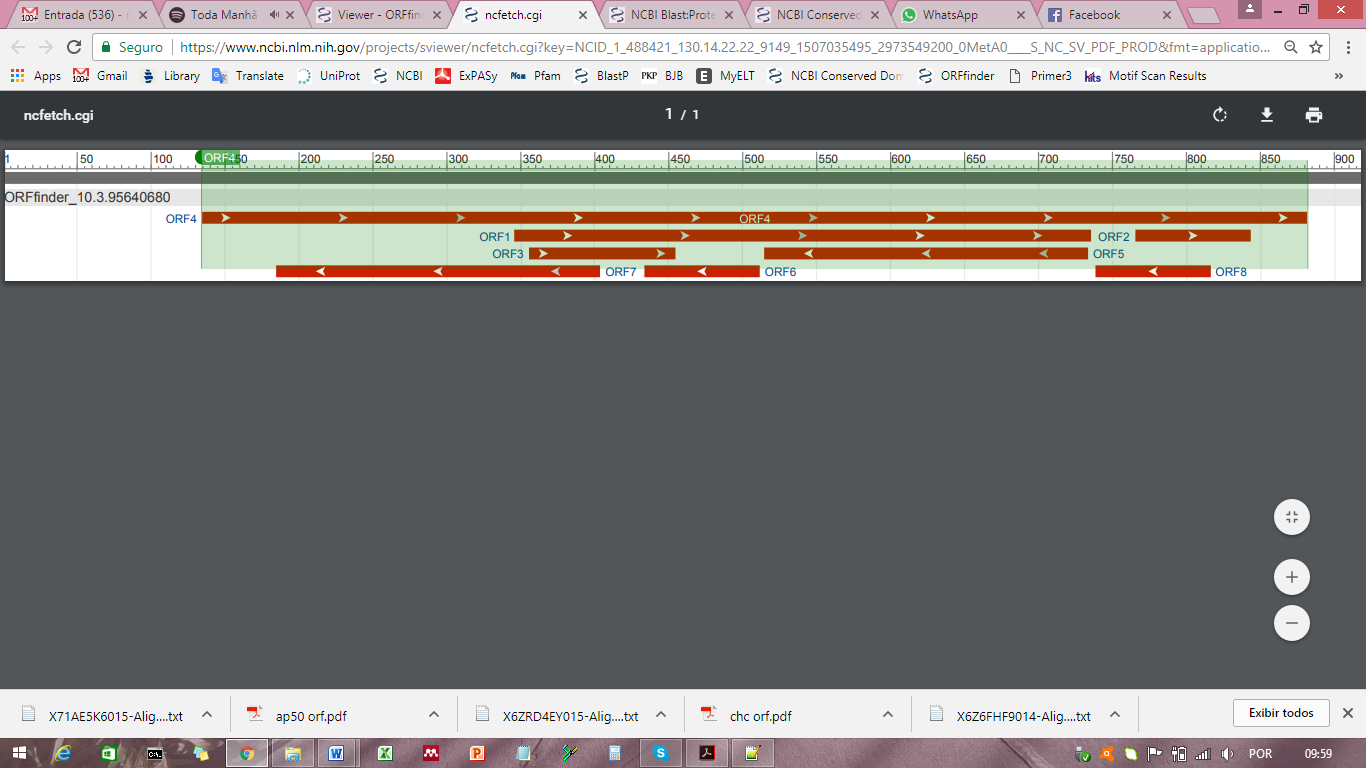


**BlastP (Non-redundant NCBI sequences)**

PREDICTED: putative inner dynein arm light chain, axonemal [Rhagoletis zephyria]

Sequence ID: [XP_017468600.1](https://www.ncbi.nlm.nih.gov/protein/1048015233?report=genbank&log$=protalign&blast_rank=1&RID=X72AYT1Z014) Length: 248 Number of Matches: 1

Range 1: 1 to 248

Score: 501 bits(1289)

E-value: 3e-179

Identities: 245/248(99%)

Query 1 MDEVDVATVDQYQTLVRYNNPVLVVKHPDKKGVPTEIELKRPLTAGALLDTKRETEEILN 60

M+EVDVATVDQYQTLVRYNNPVLVVKHPDKKGVPTEIELKRPLTAGALLDTKRETEEILN

Sbjct 1 MEEVDVATVDQYQTLVRYNNPVLVVKHPDKKGVPTEIELKRPLTAGALLDTKRETEEILN 60

Query 61 SILPPRCWEEDGQLWQQTVSSTPATRQDVINLQEMLDTRLQQTQARETGICPIRRELYTQ 120

SILPPRCWEEDGQLWQQTVSSTPATRQDVINLQEMLDTRLQQTQARETGICPIRRELYTQ

Sbjct 61 SILPPRCWEEDGQLWQQTVSSTPATRQDVINLQEMLDTRLQQTQARETGICPIRRELYTQ 120

Query 121 CFDEIIRQVTINCSERGLLLLRIRDEIAMSMEAYETLYCSSVAFGMRKALQAHEEKEMLR 180

CFDEIIRQVTINCSERGLLLLRIRDEIAMSMEAYETLYCSSVAFGMRKALQAHEEKEMLR

Sbjct 121 CFDEIIRQVTINCSERGLLLLRIRDEIAMSMEAYETLYCSSVAFGMRKALQAHEEKEMLR 180

Query 181 DRVKTLETEKESLEEIINDMKIKQEQAERRNAELRASEEKKYAEEVAFLKKTNTQLKAQL 240

DRVKTLETEKESLEEII DMKIKQEQAERRNAELR SEEKKYAEEVAFLKKTNTQLKAQL

Sbjct 181 DRVKTLETEKESLEEIIGDMKIKQEQAERRNAELRTSEEKKYAEEVAFLKKTNTQLKAQL 240

Query 241 EGITAPKK 248

EGITAPKK

Sbjct 241 EGITAPKK 248

**FBX011**

**>lcl|ORF2**

MPSASFTSSRSYVRRSRRKGANRIALPNRPSGNIMDPMIQNIPGSSASSN

ASAGANSNAIAPPLQNNTNPPNSSVISTTMNVSSIPSGSLSTPQLSSCSS

GDTSSQSAAIVSASSAAMTASAAAGAVSSETFANVSGASCSQSSTTSGPS

TSRSVAGSCNLGVAGGQKMLGATCSSNLISSSVFNTGGGFVGAHLMDTET

SSNCAGSFSSSVNITADTSSSKNNTGSSNSMYSSSVPCNSSTMSNVSAST

STSVASCSHQSPYDLRRKMPANGHEQWFPSASNTLANSSTSSASANTSNS

SPQSVSCGPPHMGGSCNISSISAMPSSSSSTSSSAIVHAPSTSATFPVNS

TATGAPLGSSPTVHSSIPQQHCSALPIGGIEDNNLMSPARKRSRRSYAQT

ADMPCMAACTGSTTPPGPTAAQYLQYELPDEVLLAIYSYLLEQDLCRLAI

VCKRFNTIANDNELWKRLYQSVFEYDLPLFSTEQSKFVFEKPEESEYSNP

WKESFRQLYSGVHVRAGFQDKKYPGRNILFFNTIQAALDYPEQAAAAAIG

GYTNVPASDPTTTNDLISIYQDSITPEDHPGPLIFVHAGHYKGEYLYIES

DVTLIGAASGNVAESVVLEREAGSTMMFVEGAKYAYVGYLTLKFSPDVTS

TVSHHKHYCLDIGENCSPTVDNCIIRSSSVVGAAVCVSGVNANPVIRNCD

ISDCENVGLYVTDYAQGTYEHNEISRNALAGIWVKNFASPIMRENHIHHG

RDVGIFTFENGMGYFEKNDIHNNRIAGFEVKAGANPTVVKCEIHHGQTGG

IYVHENGLGQFIENRIHSNNFAGVWITSNSNPTIRKNEIYNGHQGGVYIF

GEGRGLIEHNNIYGNALAGIQIRTNSDPIVRHNKIHHGQHGGIYVHEKGQ

GLIEENEVYSNTLAGVWITTGSTPVLRRNRIHSGKQVGVYFYDNGHGKLE

DNDIFNHLYSGVQIRTGSNPVIRGNKIWGGQNGGVLVYNGGLGLLEQNEI

FDNAMAGVWIKTDSNPTLKRNKIYDGRDGGICIFNGGKGILEENDIFRNT

QAGVLISTQSHPILRRNRIYDGQAAGVEITNNASATLEHNQIFKNKFGGL

CLASGVHPVVRGNNIFNNEDEVEKAVSGGQCLYKISSYTSFPMHDFYRCQ

TCNTTDRNAICVNCIKHCHAGHDVEFIRHDRFFCDCGAGTLTNQCQLQGE

PTQDTDTLYDSAAPMESHTLMVN

**Graphical representation – ORF**


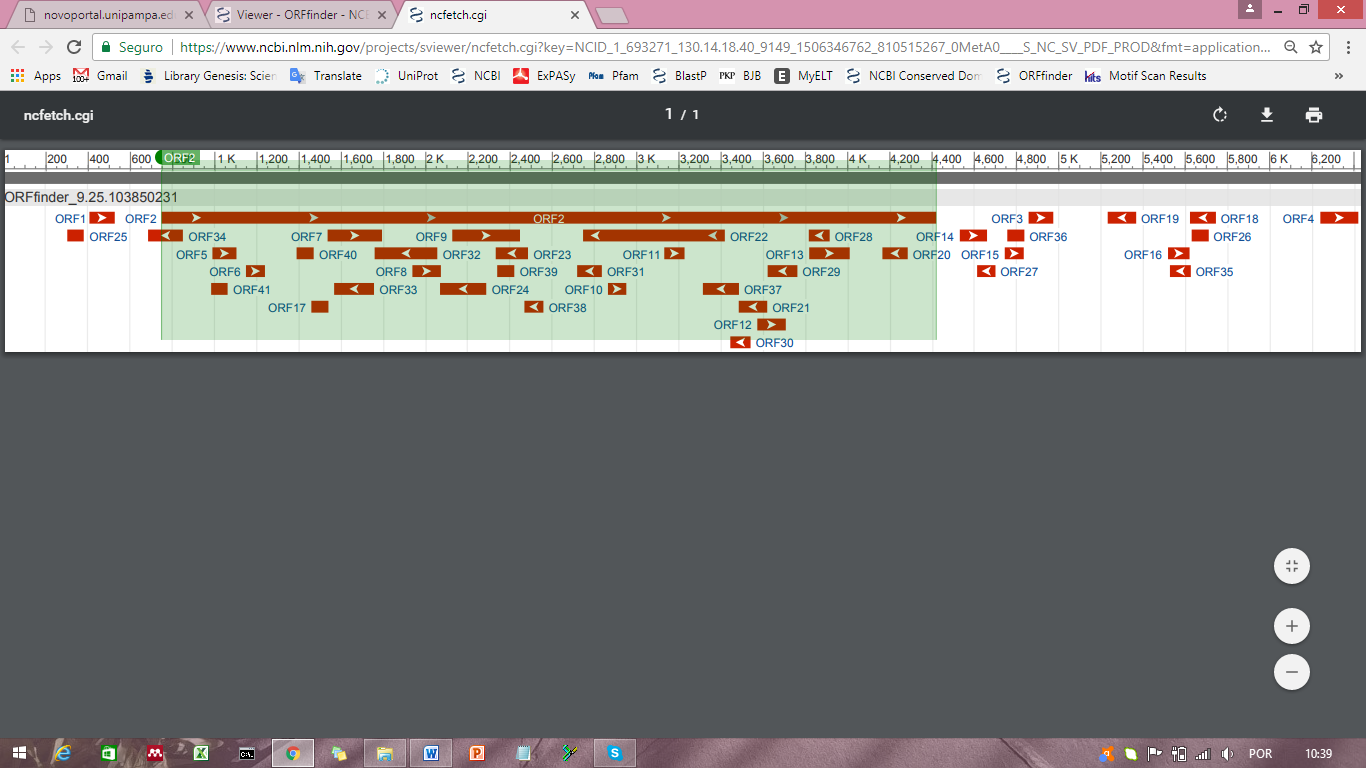


**BlastP (Non-redundant NCBI sequences)**

PREDICTED: F-box only protein 11 isoform X2 [Bactrocera oleae]

Sequence ID: [XP_014091629.1](https://www.ncbi.nlm.nih.gov/protein/929364089?report=genbank&log$=protalign&blast_rank=1&RID=WJ1U7VA4014) Length: 1238 Number of Matches: 1

Range 1: 1 to 1238

Score: 2014 bits(5217)

E-value: 0.0

Identities: 1029/1244(83%)

Query 1 MPSASFTSSRSYVRRSRRKGANRIALPNRPSGNIMDPMIQNIPGSSASSNASAGANSNAI 60

MPSASFTSSRSYVRRSRRKG NRIALPNRPSGNIMDPM QNIPG S++S++ A ++SNA

Sbjct 1 MPSASFTSSRSYVRRSRRKGVNRIALPNRPSGNIMDPMQQNIPGPSSASHSGANSSSNAG 60

Query 61 APPLQNNTNPPNSSVISTTMNVSSIPSGSLSTPQLSSCSSGDTSSQSAAIV-SASSAAMT 119

AP LQNN N NS++ ++ S+I S S ST ++SCSS DTSSQSAAIV S SSA

Sbjct 61 APSLQNNDNITNSNISPAPLDSSAIQSHSHSTSPVASCSSADTSSQSAAIVVSTSSATAA 120

Query 120 ASAAAGAVSSETFANVSGASCSQSSTTSGPSTSRSVAGSCNLGVAGGQKMLGATCSSNLI 179

S AAGAVSSE F +VSGASCSQSS + STS ++GSC+ GV + MLG++CS+N+I

Sbjct 121 TSTAAGAVSSEMFVDVSGASCSQSSNSGP-STSSGISGSCSFGVVNSKNMLGSSCSNNII 179

Query 180 SSSVFNTGGGFVGAHLMDTETSSNCAGSFSSSVNITADTSSSKNNTGSSNSMYSSSVPCN 239

SS N GG FVG HLMD+ +S+ C G SSS + + S+ N+T + NS YS+SV CN

Sbjct 180 GSSGLNGGGSFVGGHLMDSGSSNTCPGPSSSSTHSSTGNSNIVNHTSNCNSHYSNSVLCN 239

Query 240 SSTMSNVSASTSTSVASCSHQSPYDLRRKMPANGHEQWF-PSASNTLANSSTSSASANTS 298

S +SNV+ASTS S + +HQSPYDLRRKMPANGH+ W S S L++ T+ ++A++S

Sbjct 240 S--LSNVTASTSASATNSTHQSPYDLRRKMPANGHDHWCNTSTSAILSSPPTTVSTASSS 297

Query 299 N--------SSPQSVSCG-PPHMGGSCNISSISAMPSSSSSTSSSAIVHAPSTSATFPVN 349

N S+ QSVSCG PPH+GGSCN +SIS MPSSSSS+S AIVHAP TSATFPVN

Sbjct 298 NIATSNTNNSNSQSVSCGSPPHLGGSCNTASISVMPSSSSSSS--AIVHAPLTSATFPVN 355

Query 350 STATGAPLGSSPTVHSSIPQQHCSALPIGGIEDNNLMSPARKRSRRSYAQTADMPCMAAC 409

STATGAPLG SPTVHSSIPQQHCSALPIGGIEDNN M PARKRSRR YAQTA++PC+AAC

Sbjct 356 STATGAPLGPSPTVHSSIPQQHCSALPIGGIEDNNFMLPARKRSRRLYAQTAEVPCIAAC 415

Query 410 TGSTTPPGPTAAQYLQYELPDEVLLAIYSYLLEQDLCRLAIVCKRFNTIANDNELWKRLY 469

TGS GPTAAQYLQYE+PDEVLLAI+SYLLEQDLCRL++VCKRFNTIAND ELWKRLY

Sbjct 416 TGSIMLAGPTAAQYLQYEMPDEVLLAIFSYLLEQDLCRLSLVCKRFNTIANDTELWKRLY 475

Query 470 QSVFEYDLPLFSTEQSKFVFEKPEESEYSNPWKESFRQLYSGVHVRAGFQDKKYPGRNIL 529

QSVFEYD+PLF++E KFVFEKPEESE++NPWKESFRQLY GVHVRAGFQDKKY GR+IL

Sbjct 476 QSVFEYDMPLFNSELCKFVFEKPEESEFTNPWKESFRQLYRGVHVRAGFQDKKYSGRSIL 535

Query 530 FFNTIQAALDYPEQ----------AAAAAIGGYTNVPASDPTTTNDLISIYQDSITPEDH 579

FFNTIQ ALDYPE+ + + A GG N ++ + + + +IY+++I P DH

Sbjct 536 FFNTIQGALDYPEERAAAAAIASSSNSNASGGIANA-SNFGSNISAVANIYEENIQPADH 594

Query 580 PGPLIFVHAGHYKGEYLYIESDVTLIGAASGNVAESVVLEREAGSTMMFVEGAKYAYVGY 639

PGPLIF+HAGHYKGEYLYIESDV LIGAASGNVAESV+LEREAGSTMMFVEGAKYAYVGY

Sbjct 595 PGPLIFLHAGHYKGEYLYIESDVALIGAASGNVAESVILEREAGSTMMFVEGAKYAYVGY 654

Query 640 LTLKFSPDVTSTVSHHKHYCLDIGENCSPTVDNCIIRSSSVVGAAVCVSGVNANPVIRNC 699

LTLKFSPDVTSTVSHHKHYCLDIGENCSPTVDNCIIRSSSVVGAAVCVSGVNANPVIRNC

Sbjct 655 LTLKFSPDVTSTVSHHKHYCLDIGENCSPTVDNCIIRSSSVVGAAVCVSGVNANPVIRNC 714

Query 700 DISDCENVGLYVTDYAQGTYEHNEISRNALAGIWVKNFASPIMRENHIHHGRDVGIFTFE 759

DISDCENVGLYVTDYAQGTYEHNEISRNALAGIWVKNFASPIMRENHIHHGRDVGIFTFE

Sbjct 715 DISDCENVGLYVTDYAQGTYEHNEISRNALAGIWVKNFASPIMRENHIHHGRDVGIFTFE 774

Query 760 NGMGYFEKNDIHNNRIAGFEVKAGANPTVVKCEIHHGQTGGIYVHENGLGQFIENRIHSN 819

NGMGYFEKNDIHNNRIAGFEVKAGANPTVVKCEIHHGQTGGIYVHENGLGQFIENRIHSN

Sbjct 775 NGMGYFEKNDIHNNRIAGFEVKAGANPTVVKCEIHHGQTGGIYVHENGLGQFIENRIHSN 834

Query 820 NFAGVWITSNSNPTIRKNEIYNGHQGGVYIFGEGRGLIEHNNIYGNALAGIQIRTNSDPI 879

NFAGVWITSNSNPTIRKNEIYNGHQGGVYIFGEGRGLIEHNNIYGNALAGIQIRTNSDPI

Sbjct 835 NFAGVWITSNSNPTIRKNEIYNGHQGGVYIFGEGRGLIEHNNIYGNALAGIQIRTNSDPI 894

Query 880 VRHNKIHHGQHGGIYVHEKGQGLIEENEVYSNTLAGVWITTGSTPVLRRNRIHSGKQVGV 939

VRHNKIHHGQHGGIYVHEKGQGLIEENEVYSNTLAGVWITTGSTPVLRRNRIHSGKQVGV

Sbjct 895 VRHNKIHHGQHGGIYVHEKGQGLIEENEVYSNTLAGVWITTGSTPVLRRNRIHSGKQVGV 954

Query 940 YFYDNGHGKLEDNDIFNHLYSGVQIRTGSNPVIRGNKIWGGQNGGVLVYNGGLGLLEQNE 999

YFYDNGHGKLEDNDIFNHLYSGVQIRTGSNPVIRGNKIWGGQNGGVLVYNGGLGLLEQNE

Sbjct 955 YFYDNGHGKLEDNDIFNHLYSGVQIRTGSNPVIRGNKIWGGQNGGVLVYNGGLGLLEQNE 1014

Query 1000 IFDNAMAGVWIKTDSNPTLKRNKIYDGRDGGICIFNGGKGILEENDIFRNTQAGVLISTQ 1059

IFDNAMAGVWIKTDSNPTLKRNKIYDGRDGGICIFNGGKGILEENDIFRNTQAGVLISTQ

Sbjct 1015 IFDNAMAGVWIKTDSNPTLKRNKIYDGRDGGICIFNGGKGILEENDIFRNTQAGVLISTQ 1074

Query 1060 SHPILRRNRIYDGQAAGVEITNNASATLEHNQIFKNKFGGLCLASGVHPVVRGNNIFNNE 1119

SHPILRRNRIYDGQAAGVEITNNA+ATLEHNQIFKNKFGGLCLASGV P++RGNNIFNNE

Sbjct 1075 SHPILRRNRIYDGQAAGVEITNNATATLEHNQIFKNKFGGLCLASGVQPIIRGNNIFNNE 1134

Query 1120 DEVEKAVSGGQCLYKISSYTSFPMHDFYRCQTCNTTDRNAICVNCIKHCHAGHDVEFIRH 1179

DEVEKAVSGGQCLYKISSYTSFPMHDFYRCQTCNTTDRNAICVNCIK+CHAGHDVEFIRH

Sbjct 1135 DEVEKAVSGGQCLYKISSYTSFPMHDFYRCQTCNTTDRNAICVNCIKNCHAGHDVEFIRH 1194

Query 1180 DRFFCDCGAGTLTNQCQLQGEPTQDTDTLYDSAAPMESHTLMVN 1223

DRFFCDCGAGTL+NQCQLQGEPTQDTDTLYDSAAPMESHTLMVN

Sbjct 1195 DRFFCDCGAGTLSNQCQLQGEPTQDTDTLYDSAAPMESHTLMVN 1238

**HPS4 = CG4966**

**>lcl|ORF1**

MGSSKELPESGIASINFDETDSYPEFIGRTSVCSTPMTENKVLQVGNIMS

ICANPEDESNSNKQKTQTANRRNSLRLDFEKFFQNFIANPNKQLERRKSF

TDLQDSLKKISKKLSLKQFSHSFKTDVNRNGSISGIEAIESPDFIENDDD

AEQNSDNSDENNRISRTITDPTYPVFNENGQPISRSLFQEFIEKYYRLWA

ESGSGNGSAKKDIEIAQLIEEFKEFDKELKKLDGSIRQEIGDNTTNTATT

TLKADRNLNTEAPAVVATQKAKTPLDKKSLSLPLKPLADTASNEQFAAAS

RKQPGGVPLTPLMAKLSVLALNEEHTSNTWDASSVEIQTPLNTSKVFSRR

SSLKCEDAVDALATLPTNVQSTSNGLKRLELYMCGQQNMTLLLLMEEGTA

RQQPIVHNMFDICVAKFPHMESHLNQTLNVNVEGDKREGGYSFMCIDAKW

DTLERNGPWNPLELNTLECIHQDLQSNVELTDVVLRSHDAVFYGYKSGRT

EVFYKGAAHPTSGIPPPSDPMGNVAMRAKTQLERDHSYILF

**Graphical representation – ORF**


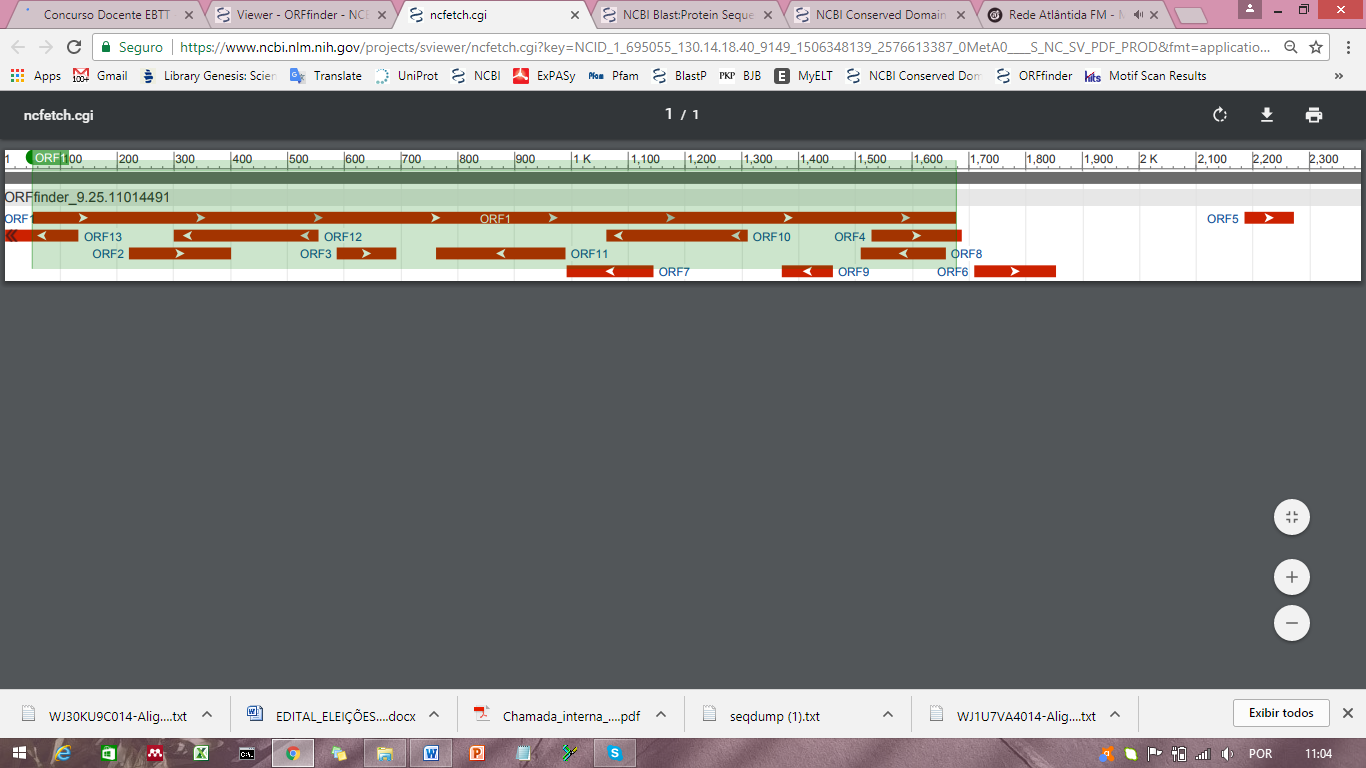


**BlastP (Non-redundant NCBI sequences)**

PREDICTED: uncharacterized protein LOC108377491 isoform X1 [Rhagoletis zephyria]

Sequence ID: [XP_017489249.1](https://www.ncbi.nlm.nih.gov/protein/1048005360?report=genbank&log$=protalign&blast_rank=1&RID=WJ30KU9C014) Length: 937 Number of Matches: 1

Range 1: 392 to 937

Score: 955 bits(2468)

E-value: 0.0

Identities: 481/546(88%)

Query 1 MGSSKELPESGIASINFDETDSYPEFIGRTSVCSTPMTENKVLQVGNIMSICANPEDESN 60

MGSSKELPESGIASINFDETDSYPEFIGRTSVCSTPMTENKVLQVGNIMSICAN EDESN

Sbjct 392 MGSSKELPESGIASINFDETDSYPEFIGRTSVCSTPMTENKVLQVGNIMSICANTEDESN 451

Query 61 SNKQ-KTQTANRRNSLRLDFEKFFQNFIANPNKQLERRKSFTDLQDSLKKISKKLSLKQF 119

SNKQ KT NRRNSL+ DFEKFFQNFIANPNKQ+ERR SFTDLQDSLKKISKKLSLKQF

Sbjct 452 SNKQQKTHNTNRRNSLKFDFEKFFQNFIANPNKQMERRNSFTDLQDSLKKISKKLSLKQF 511

Query 120 SHSFKTDVNRNGSISGIEAIESPDFIENDDD--AEQNSDNSDENNRISRTITDPTYPVFN 177

S+SFKTDVNRNGS SGIEAIESPDFIENDDD AEQNSDNSDENNR SRTITDPTYPVFN

Sbjct 512 SNSFKTDVNRNGSSSGIEAIESPDFIENDDDVDAEQNSDNSDENNRTSRTITDPTYPVFN 571

Query 178 ENGQPISRSLFQEFIEKYYRLWAESGSGNGSAKKDIEIAQLIEEFKEFDKELKKLDGSIR 237

ENGQPISRSLFQEFIEKYYRLWAE+GSGNGSAKKDIEIAQLIEEFKEFD ELK+LD S+R

Sbjct 572 ENGQPISRSLFQEFIEKYYRLWAENGSGNGSAKKDIEIAQLIEEFKEFDNELKQLDESLR 631

Query 238 QEIGDNTTNTATTT--LKADRNLNTEAPAVVATQKAKTPLDKKSLSLPLKPLADTASNEQ 295

+ G N T TTT LK D NLNTEAPAV+ QKAKTPLDKKSLSLPLKPL D +SN+Q

Sbjct 632 LDGGGNNTTITTTTTNLKTDSNLNTEAPAVMTMQKAKTPLDKKSLSLPLKPLTDASSNDQ 691

Query 296 FAAASRKQPGGVPLTPLMAKLSVLALNEEHTSNTWDASSVEIQTPLNTSKVFSRRSSLKC 355

F AASRKQPGGVPLTPLMAKLSVLALNEEHTS+TWD ++VEIQTPLNTSKVFSRRSSLKC

Sbjct 692 FVAASRKQPGGVPLTPLMAKLSVLALNEEHTSSTWDTTAVEIQTPLNTSKVFSRRSSLKC 751

Query 356 EDAVDALATLPTNVQSTSNGLKRLELYMCGQQNMTLLLLMEEGTARQQPIVHNMFDICVA 415

+DAVDALATLP N QS+SNG KRLELYMCGQQNMTLLLLMEEGTARQQP+V MFD+CVA

Sbjct 752 DDAVDALATLPANAQSSSNGTKRLELYMCGQQNMTLLLLMEEGTARQQPVVQKMFDVCVA 811

Query 416 KFPHMESHLNQTLNVNVEGDKREGGYSFMCIDAKWDTLERNGPWNPLELNTLECIHQDLQ 475

KFPHMESHLNQTLNVNVEGDKREGGYSFMCIDAKWDTLERNGPWNPLELNTLEC+HQDLQ

Sbjct 812 KFPHMESHLNQTLNVNVEGDKREGGYSFMCIDAKWDTLERNGPWNPLELNTLECMHQDLQ 871

Query 476 SNVELTDVVLRSHDAVFYGYKSGRTEVFYKGAAHPTSGIPPPSDPMGNVAMRAKTQLERD 535

+ E+ D+VLRSHDAVFYGYKSGR+E FYK AA TSGIPPPSDPMGNVAMRAKT LERD

Sbjct 872 PSDEIMDLVLRSHDAVFYGYKSGRSEFFYKEAAQQTSGIPPPSDPMGNVAMRAKTLLERD 931

Query 536 HSYILF 541

HSYILF

Sbjct 932 HSYILF 937

**SRRT = Ars2**

**>lcl|ORF16**

MSDDEDKSEDDEKAKDKKCLKRKRASSESSSSSSSDSDSSSSSSSDEDDD

DKVKDKYDLDSVKEEKGEEADKDEGVEFKDKNDTDKAVEEESEAPPVESK

LNGEDEKEPLVTQNGFVEEKRTEDNEGNNNEEEHAEKIDTDKVVEKILDD

ESKKDTSLNSDDAKDQEEVEDKDDSVVDEAKKEIETEEKENPDNVQPETI

DLDKVKDGPQPRALHRTSSIFLRNLAPSITKAEIETLCQRFDGYLRVAIA

DPLVDRRWYRRGWVTFKRDVNIKEICWNLNNTRLRDCEMGAIVNRDLSRR

VRPVNGMTAHKSIVRADIKLCAKIALNLDEKFKLWDKGSDSDIKQNAENS

KDNGDISASYGFKSNNPVLQNITDYLIEEASAEEEELLGISGENKESEGE

IIERDAQLISVLDSLILYLRIVHSVDFYNHCEYPYEDEMPNRCGIIHARG

PPPSKVSQNDIQDYIKNFENKMQTFLAKTTTVEEEELKNLGCKDAEVEVE

KFVQANTQELAKDKWLCPLSGKKFKGPEFIRKHIFNKHTEKVDEVRKEVE

FFNNYLRDPKRPQLPEHASSTKRTVSESGGLGYRPPVYPPAFMPPYAAYA

PPMMMPGRGGRGFGPGRREPMEQQRRIIGYHDLDAPANFDMFE

**Graphical representation – ORF**


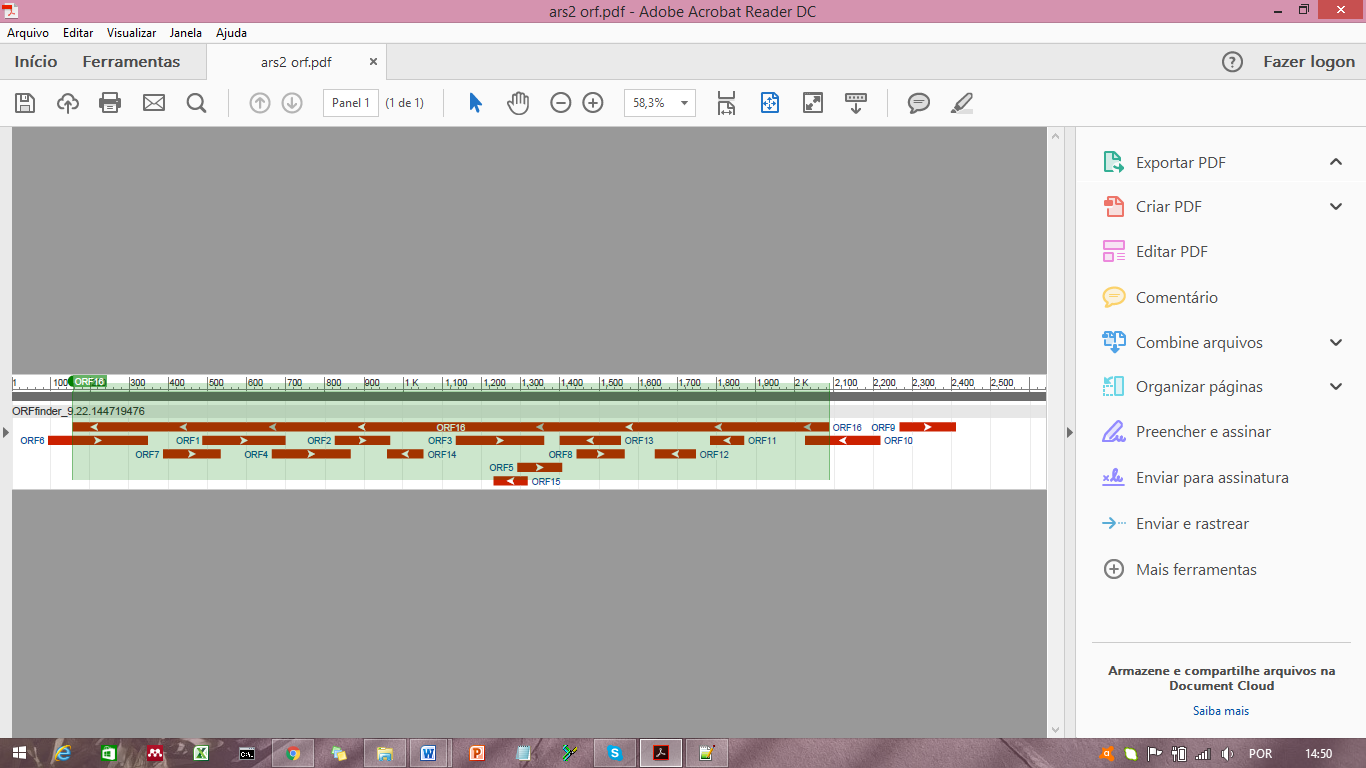


**BlastP (Non-redundant NCBI sequences)**

PREDICTED: serrate RNA effector molecule homolog isoform X2 [Rhagoletis zephyria]

Sequence ID: [XP_017476230.1](https://www.ncbi.nlm.nih.gov/protein/1048038791?report=genbank&log$=protalign&blast_rank=1&RID=WAK6U9SP014) Length: 999 Number of Matches: 1

Range 1: 367 to 999

Score: 972 bits(2512)

E-value: 0.0

Identities: 529/635(83%)

Query 13 KAKDKKCLKRKRASSESSSSSSSDSDSSSSSSSDEDDDDKVKDKYDLDSVKEEKGEEADK 72

K+KDKK LKRKR SSESSSSSSSDSD+SSSSSSDEDDDDK+KDKYDL+ VKEEK E +K

Sbjct 367 KSKDKKSLKRKRTSSESSSSSSSDSDTSSSSSSDEDDDDKIKDKYDLNIVKEEK-VETEK 425

Query 73 DEGVEFKDKNDTDKAVE--EESEAPPVESKLNGEDEK--EPLVTQNGFVEEKRTEDNEGN 128

DE E K+KN+ DK +E +E EA PVESK+NGE+EK EP +++NG EK T+D

Sbjct 426 DETDEIKEKNEVDKVIEAEKEPEAAPVESKVNGEEEKTPEPELSRNGLEAEKTTDDTTNK 485

Query 129 NNEEEHAEKIDTDKVVEKILDDESKKDTSLNSDDAKDQEEVEDKDDSVVDEAKKEIETEE 188

N+ EE+A+K+DTDKV E ++D+E+ K S N+DDAK +E DK +SVVDE KE + E+

Sbjct 486 NDGEENAQKVDTDKVDETMIDNENNKAVSANNDDAKSEETNADKANSVVDEDNKESDAEK 545

Query 189 KENPDNVQPETIDLDKVKDGPQPRALHRTSSIFLRNLAPSITKAEIETLCQRFDGYLRVA 248

+ D+ QPETIDLDKVK+ PQPRALHRTSSIFLRNLAPSITKAEIETLCQRFDGYLRVA

Sbjct 546 TKTNDD-QPETIDLDKVKEEPQPRALHRTSSIFLRNLAPSITKAEIETLCQRFDGYLRVA 604

Query 249 IADPLVDRRWYRRGWVTFKRDVNIKEICWNLNNTRLRDCEMGAIVNRDLSRRVRPVNGMT 308

IADPLVDRRWYRRGWVTFKRDVNIKEICWNLNNTRLRDCEMGAIVNRDLSRRVRPVNGMT

Sbjct 605 IADPLVDRRWYRRGWVTFKRDVNIKEICWNLNNTRLRDCEMGAIVNRDLSRRVRPVNGMT 664

Query 309 AHKSIVRADIKLCAKIALNLDEKFKLWDKGSDSDIKQNAENSKDNGDISASYGFKSNNPV 368

AHKSIVRADIKLCAKI +NLD+KFKLWDK SD D KQ +NSK NGD SYGFKSNNPV

Sbjct 665 AHKSIVRADIKLCAKITMNLDDKFKLWDKESDGDAKQACDNSKGNGDSGGSYGFKSNNPV 724

Query 369 LQNITDYLIEEASAEEEELLGISGENKESEGEIIERDAQLISVLDSLILYLRIVHSVDFY 428

LQNITDYLIEEASAEEEELLGISGENKES+GEIIERDA LISVLD LILYLR+VHSVDFY

Sbjct 725 LQNITDYLIEEASAEEEELLGISGENKESDGEIIERDAPLISVLDCLILYLRVVHSVDFY 784

Query 429 NHCEYPYEDEMPNRCGIIHARGPPPSKVSQNDIQDYIKNFENKMQTFLAKTTTVEEEELK 488

NHCEYPYEDEMPNRCGIIHARGPPPSKV+QNDIQDYIKNFENKMQTFLAK TTV++EELK

Sbjct 785 NHCEYPYEDEMPNRCGIIHARGPPPSKVTQNDIQDYIKNFENKMQTFLAKATTVDDEELK 844

Query 489 NLGCKDAEVEVEKFVQANTQELAKDKWLCPLSGKKFKGPEFIRKHIFNKHTEKVDEVRKE 548

NLG KDAE EVEKFVQANTQELAKDKWLCPLSGKKFKGPEFIRKHIFNKHTEKVDEVRKE

Sbjct 845 NLGSKDAEAEVEKFVQANTQELAKDKWLCPLSGKKFKGPEFIRKHIFNKHTEKVDEVRKE 904

Query 549 VEFFNNYLRDPKRPQLPEHASSTKRTVSESGGLGYRPPVYPPAFMPPYAAYAPPMMMPGR 608

VEFFNNYLRDPKRPQLPEHAS+TKRT SESGGLGYRPP+YPPAFMPPYAAYAPPMMMPGR

Sbjct 905 VEFFNNYLRDPKRPQLPEHASTTKRTTSESGGLGYRPPIYPPAFMPPYAAYAPPMMMPGR 964

Query 609 GGRGFGPGRREPMEQQRRIIGYHDLDAPANFDMFE 643

GGRGFG GRREPMEQQRRIIGYHDLDAPANFDMF+

Sbjct 965 GGRGFGAGRREPMEQQRRIIGYHDLDAPANFDMFD 999

**CG4572**

**RF9**

MLKKMFGRRLVMSRSWTSKSILFLRWNSVICFGLCVLIASAAVTATTVTP

GATKIKTVENAHNISKENGNNSTSEDNTATEQVRVYRLREACTDLARDEE

TLMVFSTLGGGLTAIDPITTEIRWTIADDPPIRAEQEQNVQVPQYFPDPR

DGSIYQLSDLGNLKKLPYTIPQLVASAPCRSSDGILYSGKKSDTWFMVDP

KSGRREKVMGFGSGGSQAAQNPEENENRAKHTNSHSIYLGRTQYTVMMYD

SLAKGKNAKPWNITFYDYSAHTMTPDISKEYEYLHLTTTSNGNVVTLNRK

NGKFLWKHDLTSPVVAAFLLGSEGLLSVPFTTVSDEAFEAILEESKTGNV

NTIKLFQSLYVGEHSHGLYALPSLVDKDTPRISASSPIKLLDGPNSVGDE

SDSKMVYLDDVIRKNVGIVLGHYNMPNEGSLEISPSPAKDENSHDLATIN

HYPDVTTMGSNGYSILTESNSEKNSAEIGVQTDPVIEIKTDSTNAFNKTK

RIILANSNRIQKFFNEWFMDHPSGQVHQILIVLVLVMVAMFWYMCSTMRE

LKYQSENGSKTYSGSNKSSIASSSVNAKDLIDLGDGNIRVGKISFNSNEV

LGKGCEGTFVFRGSFEERSVAVKRLLPECFTFADREVALLRESDAHENVV

RYFCTEQDRQFRYIAVELCAATLQDYTEGERSSELRSQINVWEILRQAAA

GVSHLHSLNIVHRDIKPQNVLLSLPDVSGTVRIMISDFGLCKKLNYGKAS

FSRRSGVTGTDGWIAPEMMRGQRTTTAVDIFSLGCVYYYVLTAGHHAFGD

ALKRQHNILSHEYNLSKLKVDTDNETSMPEEASKFILAEQLIADMIHNDA

QCRPLARCISAHPVFWNNQKILAFLQDVSDRVEKLQFHVEPLKSLEKNGR

CIVRNDWNAHVDPLITDDLRKYRGYMGASVRDLLRALRNKKHHYHELTPE

VQQLLGCIPNDFTNYWINKFPELISHAYHAFTICAEEPIFRHYYNTNYRF

SRPWYFDADENLFPSLKDDPKPLMKQTATGTKDESASPKRLPRTLDKQQQ

QVKQRKGVYKFRKKSDPEAVGVGVGLQRNLELMPTSASVAAEEGDDAGNK

RDAYANFKFRRNYGKSANRNFSNLNANANDNTNNEGNKEKIVTWTLPSKP

ANDE

**Graphical representation – ORF**


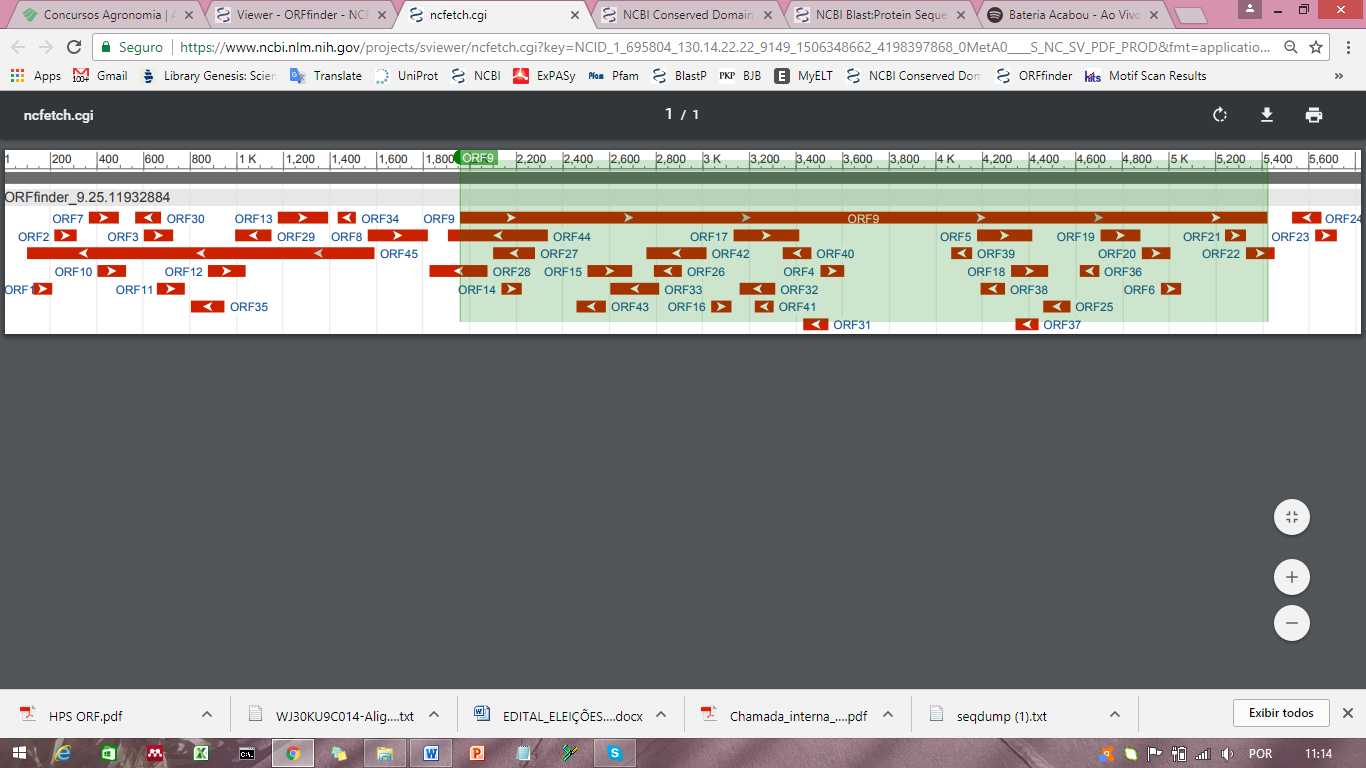


**BlastP (Non-redundant NCBI sequences)**

PREDICTED: serine/threonine-protein kinase/endoribonuclease IRE1 [Rhagoletis zephyria]

Sequence ID: [XP_017474875.1](https://www.ncbi.nlm.nih.gov/protein/1048028288?report=genbank&log$=protalign&blast_rank=1&RID=WJ3SAC9F015) Length: 1152 Number of Matches: 1

Range 1: 2 to 1152

Score: 1995 bits(5169)

E-value: 0.0

Identities: 973/1157(84%)

Query 1 MLKKMFGRRLVMSRSWTSKSILFLRWNSVICFGLCVLIASAAVTATTVTPGATKIKT-VE 59

+ +KMF R V+SRSWTS SIL L+WN ICFGLC+LIASA +T TVTPG+ KIKT VE

Sbjct 2 LRRKMFFRWSVVSRSWTSMSILLLQWNVAICFGLCILIASATLTTATVTPGSAKIKTTVE 61

Query 60 NAHNISKENGNNSTSEDNTATEQVRVYRLREACTDLARDEETLMVFSTLGGGLTAIDPIT 119

NA N+SKENGNN+ + + ATEQ+RV+RLREACTDLARDEETLMVFSTLGGGLTAIDPIT

Sbjct 62 NAQNLSKENGNNTDAREGVATEQMRVFRLREACTDLARDEETLMVFSTLGGGLTAIDPIT 121

Query 120 TEIRWTIADDPPIRAEQEQNVQVPQYFPDPRDGSIYQLSDLGNLKKLPYTIPQLVASAPC 179

+EIRWTIADDPPIRAEQE+NVQ PQY PDPRDGSIYQLSD+GNLKKLPYTIPQLVASAPC

Sbjct 122 SEIRWTIADDPPIRAEQEENVQAPQYLPDPRDGSIYQLSDMGNLKKLPYTIPQLVASAPC 181

Query 180 RSSDGILYSGKKSDTWFMVDPKSGRREKVMGFGSGGSQA--AQNPEENENRAKHTNSHSI 237

RSSDGILYSGKKSDTWFMVDPKSG R+KVMGFG G ++A Q P ENEN+AK S SI

Sbjct 182 RSSDGILYSGKKSDTWFMVDPKSGTRKKVMGFGPGATEADATQKPTENENKAKRGTSQSI 241

Query 238 YLGRTQYTVMMYDSLAKGKNAKPWNITFYDYSAHTMTPDISKEYEYLHLTTTSNGNVVTL 297

YLGRTQYTVMMYDSLAKGKNAKPWNITFYDYSAHTMTPDISKEYEYLHLTTTSNGN+VTL

Sbjct 242 YLGRTQYTVMMYDSLAKGKNAKPWNITFYDYSAHTMTPDISKEYEYLHLTTTSNGNIVTL 301

Query 298 NRKNGKFLWKHDLTSPVVAAFLLGSEGLLSVPFTTVSDEAFEAILEESKTGNVNTIKLFQ 357

+RKNGKFLW+ DLTSPVVA FLLG+EGLLSVPFTTVSDEAFE+ILEESKTGNVNT+KLFQ

Sbjct 302 DRKNGKFLWRRDLTSPVVAVFLLGAEGLLSVPFTTVSDEAFESILEESKTGNVNTVKLFQ 361

Query 358 SLYVGEHSHGLYALPSLVDKDTPRISASSPIKLLDGPNSVGDESDSKMVYLDDVIRKNVG 417

SLYVGEH HGLYALPSLVD+DTPRISAS PIKLLDGPN +ES+ KM+Y++DVIRKN G

Sbjct 362 SLYVGEHRHGLYALPSLVDEDTPRISASLPIKLLDGPNYANEESNPKMIYINDVIRKNAG 421

Query 418 IVLGHYNMPNEGSLEISPSPAKDENSHDLATINHYPDVTTMGSNGYSILTESNSEKNSAE 477

IVLGHYNMP +G+L+ISPSP+K+E S+ LATINH+PDV T+ SNGYSI+T S++EKNSAE

Sbjct 422 IVLGHYNMPTDGNLQISPSPSKEETSNGLATINHFPDVGTVDSNGYSIITGSSNEKNSAE 481

Query 478 IGVQTDPVIEIKTDSTNAFNKTKRIILANSNRIQKFFNEWFMDHPSGQVHQILIVLVLVM 537

IGVQT+PVIE+K +STNAFNKTK++I+AN+N+IQKFFN+WFM+HPSG+VHQILIVLVL M

Sbjct 482 IGVQTEPVIELKIESTNAFNKTKKVIIANTNKIQKFFNDWFMEHPSGKVHQILIVLVLAM 541

Query 538 VAMFWYMCSTMRELKYQSENGSKTYSGSNKSSIASSSVNAKDLIDLGDGNIRVGKISFNS 597

VAMFWYMCSTMRELK QSENGSKTYS SNKSS SS++NA DLIDLGDGNIRVGKISFNS

Sbjct 542 VAMFWYMCSTMRELKNQSENGSKTYSSSNKSSGGSSNINALDLIDLGDGNIRVGKISFNS 601

Query 598 NEVLGKGCEGTFVFRGSFEERSVAVKRLLPECFTFADREVALLRESDAHENVVRYFCTEQ 657

+EVLGKGCEGTFVFRG+FEERSVAVKRLLPECFTFADREVALLRESDAHENVVRYFCTEQ

Sbjct 602 SEVLGKGCEGTFVFRGTFEERSVAVKRLLPECFTFADREVALLRESDAHENVVRYFCTEQ 661

Query 658 DRQFRYIAVELCAATLQDYTEGERSSELRSQINVWEILRQAAAGVSHLHSLNIVHRDIKP 717

DRQFRYIAVELCAATLQDYTEGERS ELR+QINVWE+LRQAAAG+SHLHSL+IVHRDIKP

Sbjct 662 DRQFRYIAVELCAATLQDYTEGERSKELRTQINVWEVLRQAAAGLSHLHSLHIVHRDIKP 721

Query 718 QNVLLSLPDVSGTVRIMISDFGLCKKLNYGKASFSRRSGVTGTDGWIAPEMMRGQRTTTA 777

QNVLLSLPD+SGTVR+MISDFGLCKKLN+GK SFSRRSGVTGTDGWIAPEMMRG RTTTA

Sbjct 722 QNVLLSLPDISGTVRVMISDFGLCKKLNFGKTSFSRRSGVTGTDGWIAPEMMRGLRTTTA 781

Query 778 VDIFSLGCVYYYVLTAGHHAFGDALKRQHNILSHEYNLSKLKVDTDNETSMPEEASKFIL 837

VDIFSLGCVYYYVLT GHHAFGDALKRQHNIL+H+YNLSKLKVDTD++ +MPEEASKFIL

Sbjct 782 VDIFSLGCVYYYVLTGGHHAFGDALKRQHNILAHDYNLSKLKVDTDDDENMPEEASKFIL 841

Query 838 AEQLIADMIHNDAQCRPLARCISAHPVFWNNQKILAFLQDVSDRVEKLQFHVEPLKSLEK 897

AEQLIADMIH DAQCRPLARCISAHPVFWNNQKILAFLQDVSDRVEKLQFHVEPLKSLEK

Sbjct 842 AEQLIADMIHKDAQCRPLARCISAHPVFWNNQKILAFLQDVSDRVEKLQFHVEPLKSLEK 901

Query 898 NGRCIVRNDWNAHVDPLITDDLRKYRGYMGASVRDLLRALRNKKHHYHELTPEVQQLLGC 957

NGRCIVR+DWNAHVDPLIT+DLRKYRGYMGASVRDLLRALRNKKHHYHELTPEVQQ LGC

Sbjct 902 NGRCIVRDDWNAHVDPLITEDLRKYRGYMGASVRDLLRALRNKKHHYHELTPEVQQKLGC 961

Query 958 IPNDFTNYWINKFPELISHAYHAFTICAEEPIFRHYYNTNYRFSRPWYFDADENLFPSLK 1017

IP+DFTNYWINKFPELISHAYHAF+ICAEEPIF HYYN +Y FSRPWYFDAD+NLF SL+

Sbjct 962 IPHDFTNYWINKFPELISHAYHAFSICAEEPIFGHYYNADYHFSRPWYFDADDNLFQSLQ 1021

Query 1018 DDPKPLMKQTATGTKDESASPKRLPRTLDKQQQQVKQRKGVYKFRKKSDPEAVGVGVGLQ 1077

DPKPL +Q A G KD SASPKR PRT +K QQQ+KQRKG+Y FRK D A GVGVGLQ

Sbjct 1022 HDPKPLQRQVAAGAKDGSASPKRPPRTPEK-QQQLKQRKGIYNFRKTPD-AAEGVGVGLQ 1079

Query 1078 RNLELMPTSASVAAEEGDDAGNKRDAYANFKFRRNYGKSANRNFSNLNANANDNTNNEGN 1137

RNL+L + AAEE D+AG KRD +ANFKFRRNYGKSANR + N NANAN N NN GN

Sbjct 1080 RNLDL----GTAAAEEEDEAGAKRDVFANFKFRRNYGKSANRIYGNFNANANANANNGGN 1135

Query 1138 KEKIVTWTLPSKPANDE 1154

KEK VTWTLP K ANDE

Sbjct 1136 KEKNVTWTLPGKQANDE 1152

**Egghead**

**>lcl|ORF9**

MNSSTKHLLHCTLLIIVIITFEVFSGGIKINENSFTLVDPWTEYGQVASV

LLYLLRFLTILTLPQVLFNFCGLVIYNAFPEKVVLKGSPILAPFICIRVV

TRGDFADLVKSNVLRNMNTCLDTGLENFLVEVVTDKPVNLAQHRRIREIV

VPKEYKTRTGALFKSRALQYCLEDDVNVLNDSDWIVHLDEETLLTENSVR

GIINFVLDGKHPFGQGLITYANENVVNWLTTLADSFRVSDDMGKLRLQFK

LFHKPLFSWKGSYVVTQVAAERQVSFDNGIDGSVAEDCFFAMRAFAQGYT

FNFIEGEMYEKSPFTLLDFLQQRKRWLQGILLVVHSKIIPLKHKLLLGIS

VYSWVTMPLSTSNIIFAGLYPIPCPNLVDFVCAFIAAVNIYMYVFGVIKS

FSLYRFGLLKFMACVLGAVCTIPVNVVIENIAVIWGLFGKKHKFYVVQKD

VRAMETV

**Graphical representation – ORF**
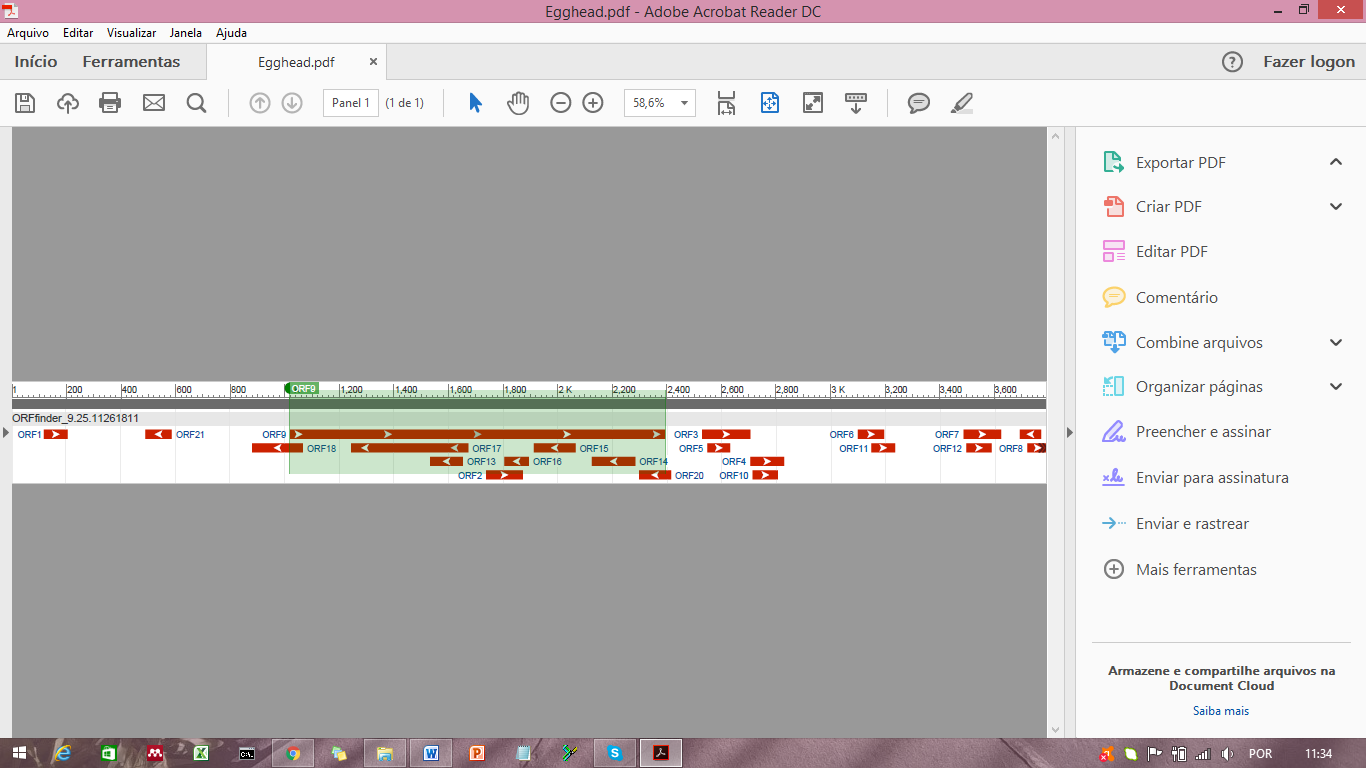


**BlastP (Non-redundant NCBI sequences)**

beta-1,4-mannosyltransferase egh [Ceratitis capitata]

Sequence ID: [XP_012161001.1](https://www.ncbi.nlm.nih.gov/protein/807040176?report=genbank&log$=protalign&blast_rank=1&RID=WJ4TD1VS015) Length: 457 Number of Matches: 1

Range 1: 1 to 457

Score: 924 bits(2388)

E-value: 0.0

Identities: 447/457(98%)

Query 1 MNSSTKHLLHCTLLIIVIITFEVFSGGIKINENSFTLVDPWTEYGQVASVLLYLLRFLTI 60

MNSSTKHLLHCTLLIIVIITFEVFSGGIKI+ENSFTLVDPWTEYGQVA+VLLYLLRFLT

Sbjct 1 MNSSTKHLLHCTLLIIVIITFEVFSGGIKIDENSFTLVDPWTEYGQVAAVLLYLLRFLTF 60

Query 61 LTLPQVLFNFCGLVIYNAFPEKVVLKGSPILAPFICIRVVTRGDFADLVKSNVLRNMNTC 120

LTLPQVLFNFCGLV YNAFPEKVVLKGSPILAPFICIRVVTRGDFADLVK+NVLRNMNTC

Sbjct 61 LTLPQVLFNFCGLVFYNAFPEKVVLKGSPILAPFICIRVVTRGDFADLVKTNVLRNMNTC 120

Query 121 LDTGLENFLVEVVTDKPVNLAQHRRIREIVVPKEYKTRTGALFKSRALQYCLEDDVNVLN 180

LDTGLENFL+EVVTDKPVNLAQHRRIREIVVPKEYKTRTGALFKSRALQYCLEDDVNVLN

Sbjct 121 LDTGLENFLIEVVTDKPVNLAQHRRIREIVVPKEYKTRTGALFKSRALQYCLEDDVNVLN 180

Query 181 DSDWIVHLDEETLLTENSVRGIINFVLDGKHPFGQGLITYANENVVNWLTTLADSFRVSD 240

D+DW+VHLDEETLLTENSVRGIINFVLDGKHPFGQGLITYANENVVNWLTTLADSFRVSD

Sbjct 181 DNDWVVHLDEETLLTENSVRGIINFVLDGKHPFGQGLITYANENVVNWLTTLADSFRVSD 240

Query 241 DMGKLRLQFKLFHKPLFSWKGSYVVTQVAAERQVSFDNGIDGSVAEDCFFAMRAFAQGYT 300

DMGKLRLQFKLFHKPLFSWKGSYVVTQV AERQVSFDNGIDGSVAEDCFFAMRAF+QGYT

Sbjct 241 DMGKLRLQFKLFHKPLFSWKGSYVVTQVGAERQVSFDNGIDGSVAEDCFFAMRAFSQGYT 300

Query 301 FNFIEGEMYEKSPFTLLDFLQQRKRWLQGILLVVHSKIIPLKHKLLLGISVYSWVTMPLS 360

FNFIEGEMYEKSPFTLLDFLQQRKRWLQGILLVVHSKIIPLKHKLLLGISVYSWVTMPLS

Sbjct 301 FNFIEGEMYEKSPFTLLDFLQQRKRWLQGILLVVHSKIIPLKHKLLLGISVYSWVTMPLS 360

Query 361 TSNIIFAGLYPIPCPNLVDFVCAFIAAVNIYMYVFGVIKSFSLYRFGLLKFMACVLGAVC 420

TSNIIFAGLYPIPCPNLVDFVCAFIAAVNIYMYVFGVIKSFSLYRFGLLKFMACVLGAVC

Sbjct 361 TSNIIFAGLYPIPCPNLVDFVCAFIAAVNIYMYVFGVIKSFSLYRFGLLKFMACVLGAVC 420

Query 421 TIPVNVVIENIAVIWGLFGKKHKFYVVQKDVRAMETV 457

TIPVNVVIENIAVIWGLFGKKHKFYVVQKDVRAMETV

Sbjct 421 TIPVNVVIENIAVIWGLFGKKHKFYVVQKDVRAMETV 457

**ninaC**

**>lcl|ORF28**

MYLPYTQLPDPTDKFEIYEEIAQGVNAKVFRAKELENDRMVALKIQHYDE

DHQVSIEEEYRTLRDHCNHPNLPEFYGVYKLVKPNAPDEIWFVMEYCSGG

TAVDMVNKLVKLERRMREEHIAYIIRETCRGAIELNRNHVIHRDIRGDNI

LLTKDGRVKLCDFGLSREVDSTFGKRGTCIGSPCWMAPEVVTAMSAKEPE

VTSRADVWALGITTIELADGQAPFADMHPTRVMFQIVRNPPPTLLRPTNW

SQQINDFVSECLEKNVENRPMMVEMIEHPFLNELFDNDEEMQADIRELLV

FCRDAPAIYKEPEIFVDRGYIKRFDGKPERMYPEDLAAIENATEEMILES

LRNRIELGGSYSFIGDVLLSLNSNDLQKDFDEEFHSKYKFKSRSQNSPHI

FAVADIAYQDMLHHKEPQHIVFSGESFSGKSTNVRLLINHLCYLGSGNRG

ATTRVESSIDAIQMLVNAGTPINNESTRCVLQYFLTFGQTGKLSGAVFNM

YMLEKLRVSTTDMNQHNFHIFYYFYDFMNSGNLLKEYHLKNDRGYRYLRI

PADTQTTKLKYHRDDPIGNVENYKRFESILQDLDFNHKQLETLRKVLAAI

LNIGNIRFRHSGKYAEVENTEMVTRIAELLRVDEKKYMWSLTNFIMVKGG

IAERRQYSTEEARDARDAVASTIYCRLVDWIINKINMNLSFPRAVFGDTN

AVVIHDMYGFECFHRNGLEQLMINTFNEQMQYHYNQRIFVNEMLEMEADD

IPTDNLNFYDNKVALDNLLTKPDGLFYIIDDASRTSQDQDLIMDRVVEKH

SQFVKKHTATEISVAHYTGRIIYDTRAFTDINRDFVPPEMIESFRSSMDE

NIMMMFTNQLTKAGNLTMPFESVQHKPEDSQRKSYPLNTLSAGCISQVNN

LRTLAANFRFTCLNLLKTLTGNINLGVHFVRCIRADLEYKPRAFHADMVQ

QQMKALGVLDTVIGRQRGYSCRITFQEFLRRYQFLAFDFDETVDITKENC

RLLLIRLKMEGWAIGKMKVFLRYYNDEFLARLYEVQVKKVIKVQSMMRAL

LARKRMKGGKDPKKAKGITRNDEAAAKIQKGNKERSEKDFDVEYFDASET

PSEAEELFESARKEEALSAARLAEVEQATQTEDD

**Graphical representation – ORF**


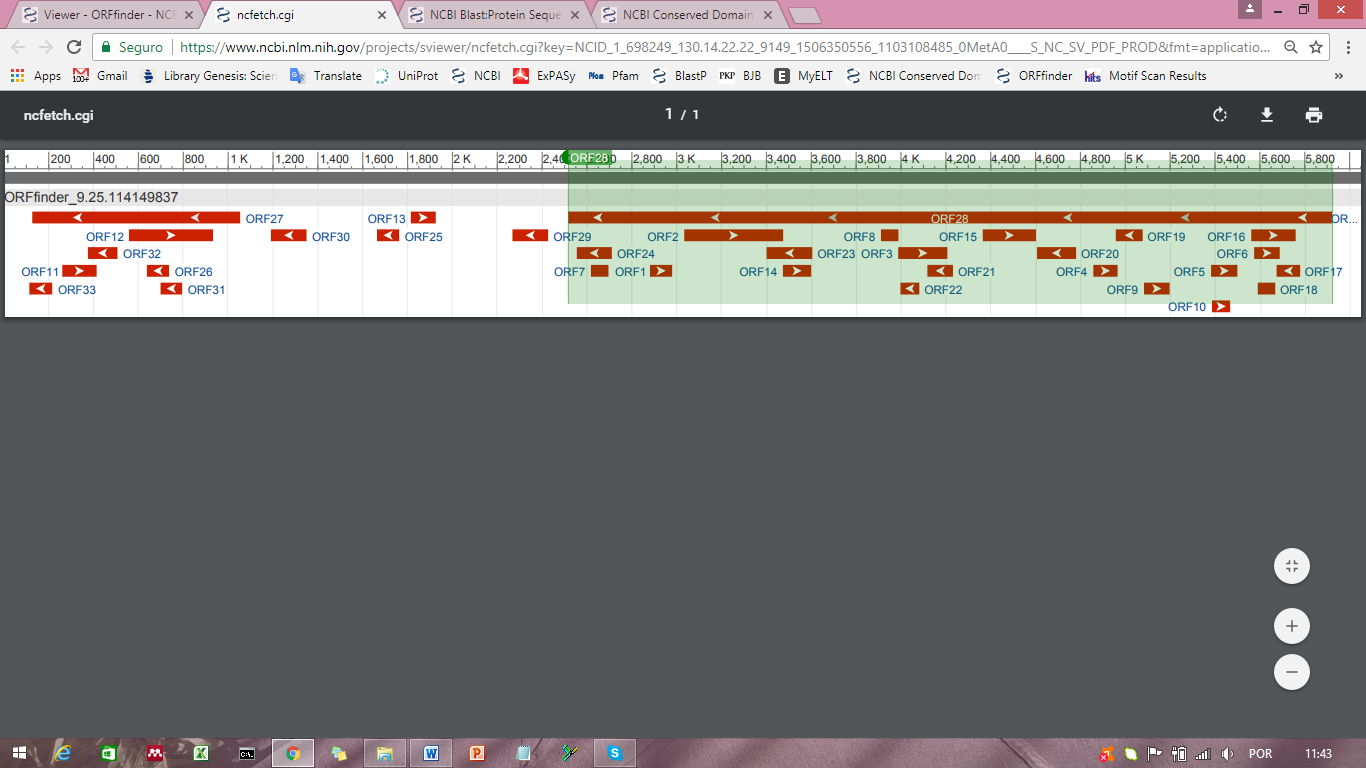


**BlastP (Non-redundant NCBI sequences)**

PREDICTED: neither inactivation nor afterpotential protein C [Zeugodacus cucurbitae]

Sequence ID: [XP_011193874.1](https://www.ncbi.nlm.nih.gov/protein/751476755?report=genbank&log$=protalign&blast_rank=1&RID=WJ5FBJA2014) Length: 1505 Number of Matches: 1

Range 1: 1 to 1081

Score: 2171 bits(5626)

E-value: 0.0

Identities: 1031/1081(95%)

Query 1 MYLPYTQLPDPTDKFEIYEEIAQGVNAKVFRAKELENDRMVALKIQHYDEDHQVSIEEEY 60

MYLPYTQLPDPTDKFEIYEEIAQGVNAKVFRAKEL+N+RMVALKIQHYDE+HQ+SIEEEY

Sbjct 1 MYLPYTQLPDPTDKFEIYEEIAQGVNAKVFRAKELDNERMVALKIQHYDEEHQISIEEEY 60

Query 61 RTLRDHCNHPNLPEFYGVYKLVKPNAPDEIWFVMEYCSGGTAVDMVNKLVKLERRMREEH 120

RTLRDHCNHPNLPEFYGVYKL KPNAPDEIWFVMEYCSGGTAVDMVNKLVKLERRMREEH

Sbjct 61 RTLRDHCNHPNLPEFYGVYKLEKPNAPDEIWFVMEYCSGGTAVDMVNKLVKLERRMREEH 120

Query 121 IAYIIRETCRGAIELNRNHVIHRDIRGDNILLTKDGRVKLCDFGLSREVDSTFGKRGTCI 180

IAYIIRETCRGAIELNRNHVIHRDIRGDNILLTKDGRVKLCDFGLSREVDSTFGKRGTCI

Sbjct 121 IAYIIRETCRGAIELNRNHVIHRDIRGDNILLTKDGRVKLCDFGLSREVDSTFGKRGTCI 180

Query 181 GSPCWMAPEVVTAMSAKEPEVTSRADVWALGITTIELADGQAPFADMHPTRVMFQIVRNP 240

GSPCWMAPEVVTAMSAK+PE+TSRADVWALGITTIELADGQ PFADMHPTRVMFQIVRNP

Sbjct 181 GSPCWMAPEVVTAMSAKDPEITSRADVWALGITTIELADGQPPFADMHPTRVMFQIVRNP 240

Query 241 PPTLLRPTNWSQQINDFVSECLEKNVENRPMMVEMIEHPFLNELFDNDEEMQADIRELLV 300

PPTLLRPTNWSQQINDFVSECLEKNVENRPMMVEMIEHPFLNELFDN+EEMQAD+RELLV

Sbjct 241 PPTLLRPTNWSQQINDFVSECLEKNVENRPMMVEMIEHPFLNELFDNEEEMQADLRELLV 300

Query 301 FCRDAPAIYKEPEIFVDRGYIKRFDGKPERMYPEDLAAIENATEEMILESLRNRIELGGS 360

FCRDAPAIYK+PEIFVDRGYIKRFDGKPERMYPEDLAAIENAT+EMILESLRNR+ELGGS

Sbjct 301 FCRDAPAIYKDPEIFVDRGYIKRFDGKPERMYPEDLAAIENATDEMILESLRNRMELGGS 360

Query 361 YSFIGDVLLSLNSNDLQKDFDEEFHSKYKFKSRSQNSPHIFAVADIAYQDMLHHKEPQHI 420

YSFIGDVLLSLNSNDLQ DFD+EFHSKYKFKSRSQNSPHIFAVADIAYQDMLHHKEPQH+

Sbjct 361 YSFIGDVLLSLNSNDLQTDFDDEFHSKYKFKSRSQNSPHIFAVADIAYQDMLHHKEPQHV 420

Query 421 VFSGESFSGKSTNVRLLINHLCYLGSGNRGATTRVESSIDAIQMLVNAGTPINNESTRCV 480

VFSGESFSGKSTN+RLLINHLCYLGSGNRGAT+RVE+SI+AIQMLVNAGTPINNESTRCV

Sbjct 421 VFSGESFSGKSTNIRLLINHLCYLGSGNRGATSRVENSIEAIQMLVNAGTPINNESTRCV 480

Query 481 LQYFLTFGQTGKLSGAVFNMYMLEKLRVSTTDMNQHNFHIFYYFYDFMNSGNLLKEYHLK 540

LQYFLTFGQTGKLSGA+FNMYMLEKLRVSTTDMNQHNFHIFYYFYDFMNS +LKEY+LK

Sbjct 481 LQYFLTFGQTGKLSGAIFNMYMLEKLRVSTTDMNQHNFHIFYYFYDFMNSEGILKEYNLK 540

Query 541 NDRGYRYLRIPADTQTTKLKYHRDDPIGNVENYKRFESILQDLDFNHKQLETLRKVLAAI 600

++RGYRYLRIPA+ Q TKLKYHRDDPI NVE+YKRFESILQDLDFNHKQLETLRKVLAAI

Sbjct 541 SERGYRYLRIPAEAQPTKLKYHRDDPINNVEHYKRFESILQDLDFNHKQLETLRKVLAAI 600

Query 601 LNIGNIRFRHSGKYAEVENTEMVTRIAELLRVDEKKYMWSLTNFIMVKGGIAERRQYSTE 660

LNIGNIRFR SGKYAEVENTEMVTRIAELLRVDEKKYMWSLTNFIMVKGGIAERRQYSTE

Sbjct 601 LNIGNIRFRTSGKYAEVENTEMVTRIAELLRVDEKKYMWSLTNFIMVKGGIAERRQYSTE 660

Query 661 EARDARDAVASTIYCRLVDWIINKINMNLSFPRAVFGDTNAVVIHDMYGFECFHRNGLEQ 720

EARDARDAVASTIYCRLVDWIINKINMNLSFPRAVFGDTNAVV+HDM+GFECFHRNGL+Q

Sbjct 661 EARDARDAVASTIYCRLVDWIINKINMNLSFPRAVFGDTNAVVVHDMFGFECFHRNGLDQ 720

Query 721 LMINTFNEQMQYHYNQRIFVNEMLEMEADDIPTDNLNFYDNKVALDNLLTKPDGLFYIID 780

LMINT NEQMQYHYNQRIFVNEMLEMEADDIPTDNLNFYDNK ALDNLLTKPDGLFYIID

Sbjct 721 LMINTLNEQMQYHYNQRIFVNEMLEMEADDIPTDNLNFYDNKTALDNLLTKPDGLFYIID 780

Query 781 DASRTSQDQDLIMDRVVEKHSQFVKKHTATEISVAHYTGRIIYDTRAFTDINRDFVPPEM 840

DASRTSQDQDLIMDRVVEKHSQFVKKHTATEISVAHYTGRIIYDTRAFTDINRDFVPPEM

Sbjct 781 DASRTSQDQDLIMDRVVEKHSQFVKKHTATEISVAHYTGRIIYDTRAFTDINRDFVPPEM 840

Query 841 IESFRSSMDENIMMMFTNQLTKAGNLTMPFESVQHKPEDSQRKSYPLNTLSAGCISQVNN 900

IESFRSSMDENIMMMFTNQLTKAGNLTMPFESVQHKPEDSQRKSYPLNTLSAGCISQVNN

Sbjct 841 IESFRSSMDENIMMMFTNQLTKAGNLTMPFESVQHKPEDSQRKSYPLNTLSAGCISQVNN 900

Query 901 LRTLAANFRFTCLNLLKTLTGNINLGVHFVRCIRADLEYKPRAFHADMVQQQMKALGVLD 960

LRTLAANFRFTCL LLKTLT + NLGVHFVRCIRADLEYKPRAFH+DMVQQQMKALGVLD

Sbjct 901 LRTLAANFRFTCLTLLKTLTLSSNLGVHFVRCIRADLEYKPRAFHSDMVQQQMKALGVLD 960

Query 961 TVIGRQRGYSCRITFQEFLRRYQFLAFDFDETVDITKENCRLLLIRLKMEGWAIGKMKVF 1020

TV+GRQRGYSCRITFQEFLRRYQFLAFDFDETVDITKENCRLL+IRLKMEGWAIGK KVF

Sbjct 961 TVVGRQRGYSCRITFQEFLRRYQFLAFDFDETVDITKENCRLLMIRLKMEGWAIGKTKVF 1020

Query 1021 LRYYNDEFLARLYEVQVKKVIKVQSMMRALLARKRMKGGKDPKKAKGITRNDEAAAKIQK 1080

LRYYNDEFLARLYE+QVKKVIKVQSMMRALLARKRMKGGKDPKKAKG TRNDEAA KIQK

Sbjct 1021 LRYYNDEFLARLYEIQVKKVIKVQSMMRALLARKRMKGGKDPKKAKGTTRNDEAATKIQK 1080

Query 1081 G 1081

Sbjct 1081 A 1081

**Snipper**

**>lcl|ORF8**

MALTRLARQLGLLETIYVEGEPGADKTNTSSYLTKLRKPHLTMQPYKYVI

CIDFEATCWENQAPPKWRESEIIEFPAMLVNLQTGKIEAEFHKYVMPIES

PKLSAYCTELTGIQQQTVDNGVPLQTAIMMFQEWLRKELRARNLQLPKMS

KDKLIGNCAFVTWTDWDFGICLAKECKRKLLKKPSYFNQWIDLRAIFRQW

YKYRPINFADALSHVKLVFEGREHSGRDDARNLAALAYKIASDGAQFAIT

KDLAPFQLNSNCIL

**Graphical representation – ORF**


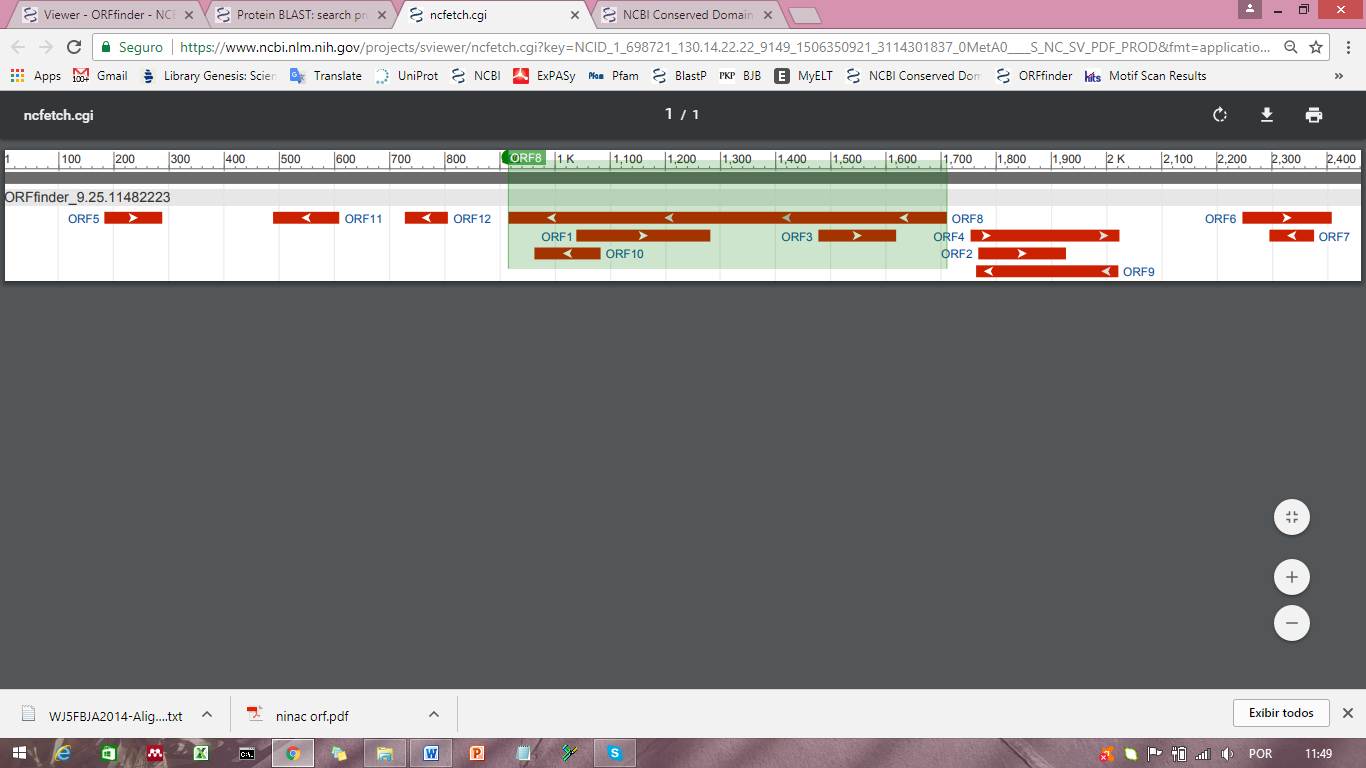


**BlastP (Non-redundant NCBI sequences)**

PREDICTED: ERI1 exoribonuclease 2 isoform X2 [Rhagoletis zephyria]

Sequence ID: [XP_017493825.1](https://www.ncbi.nlm.nih.gov/protein/1048006295?report=genbank&log$=protalign&blast_rank=1&RID=WJ5Y8PSR014) Length: 263 Number of Matches: 1

Range 1: 1 to 263

Score: 497 bits(1280)

E-value: 2e-177

Identities: 234/264(89%)

Query 1 MALTRLARQLGLLETIYVEGEPGADKTNTSSYLTKLRKPHLTMQPYKYVICIDFEATCWE 60

MAL RLARQLGLLETIYVEGE G DK N SSYLTKLRKP L+MQPYKY+IC+DFEATCWE

Sbjct 1 MALIRLARQLGLLETIYVEGESGTDKNN-SSYLTKLRKPQLSMQPYKYLICMDFEATCWE 59

Query 61 NQAPPKWRESEIIEFPAMLVNLQTGKIEAEFHKYVMPIESPKLSAYCTELTGIQQQTVDN 120

NQAPPKWRE+EIIEFPA+++NLQTGKIEAEFHKYVMPIESPKLSA+CTELTGI+QQ+VDN

Sbjct 60 NQAPPKWREAEIIEFPAVMINLQTGKIEAEFHKYVMPIESPKLSAFCTELTGIKQQSVDN 119

Query 121 GVPLQTAIMMFQEWLRKELRARNLQLPKMSKDKLIGNCAFVTWTDWDFGICLAKECKRKL 180

G+PLQTAIMMFQEWLRKELRARNLQLPKMSKDKLIGNCAFVTWTDWDFGICLAKEC+RK

Sbjct 120 GMPLQTAIMMFQEWLRKELRARNLQLPKMSKDKLIGNCAFVTWTDWDFGICLAKECQRKR 179

Query 181 LKKPSYFNQWIDLRAIFRQWYKYRPINFADALSHVKLVFEGREHSGRDDARNLAALAYKI 240

LKKP+YFNQWIDLRAIFR+WYKY+PINFADALSHV L FEGREHSG DDARNLAALAYK+

Sbjct 180 LKKPTYFNQWIDLRAIFREWYKYKPINFADALSHVGLAFEGREHSGIDDARNLAALAYKL 239

Query 241 ASDGAQFAITKDLAPFQLNSNCIL 264

DGA AITKDLAPFQLNSNCIL

Sbjct 240 TCDGAPLAITKDLAPFQLNSNCIL 263

**Nibbler**

**>lcl|ORF1**

MSICRIPAGYEDDEDDLSEVMQKITVVPDITVGAGLNNENFDRNLSSEVA

SWFFFYKDEWNSYKRMPSAQHTLKAMLMTDEDPLLFALKLFANCPGCNNL

KNKSLALFILETVCDLHKTKPEISTNCTDNTRMIAFNFVKTCGIVPLCKA

VISTYELKKIRDLLVPKIKDLIAAGHIKDAAHWTMHLQITHIFGIFDIVF

PLILQDKVSLAEEYLNVAKDLALPTVKFLDSLLDKQKTVIDHCEEVLTKY

EYKDVKCNILTYRPMSKLVTRLAKKYKIDAHDTPNLNFTKACSYLHYLWR

KYRDGGMSHDAWVELAHDAAVTKPIQMDLIKNVVAYGDIKEAGYWISFYK

IPLEECPDAVLDYLKQNRPYGTDKHNTSNKSSKSANSSRGAMNKSKRSNS

FKNNQYLTLNLPIDCIIIVDDRVKFLQMLDHLEGQLLIAFDSEWKPTVCN

ENVISVLQMATMDRVYLLDCLSSNLSNELWQQLGRRVFNNLEILKIGFSL

HQDLRMLHKSLPLELNLNAKTCYLDLRELWRRLKYLQIVKFPFECASNSG

ESLCTLTEICLGKKLDKSNQFSNWANRPLRHDQVIYAALDAHCLLLVYQV

VSEILVRMGLDVDQVVDEVTTGKTGYLFQKKRVITEQNDERSSTRPVKMD

AEGVNSVSAKNENNRSIGTKFICDTMMTGLSKEFRKLGIDCVEIVNNDLG

YYIELARKEHRYILTRDSRYDLFVNELPPEQCLQIPSDSSVDQVLNIIRL

LDIKIYECNLFTRCLSCNSNEFIFALRHEMQTMRFGQALDEIKDMSALNM

NPYGKTYHLLNVSSHIVKTKTTYRGKFIKLNRIRSHILRSKEYFYICDNC

GICTWDGAYSIHSSVKDAVLSDVFEAATAPGI

**Graphical representation – ORF**


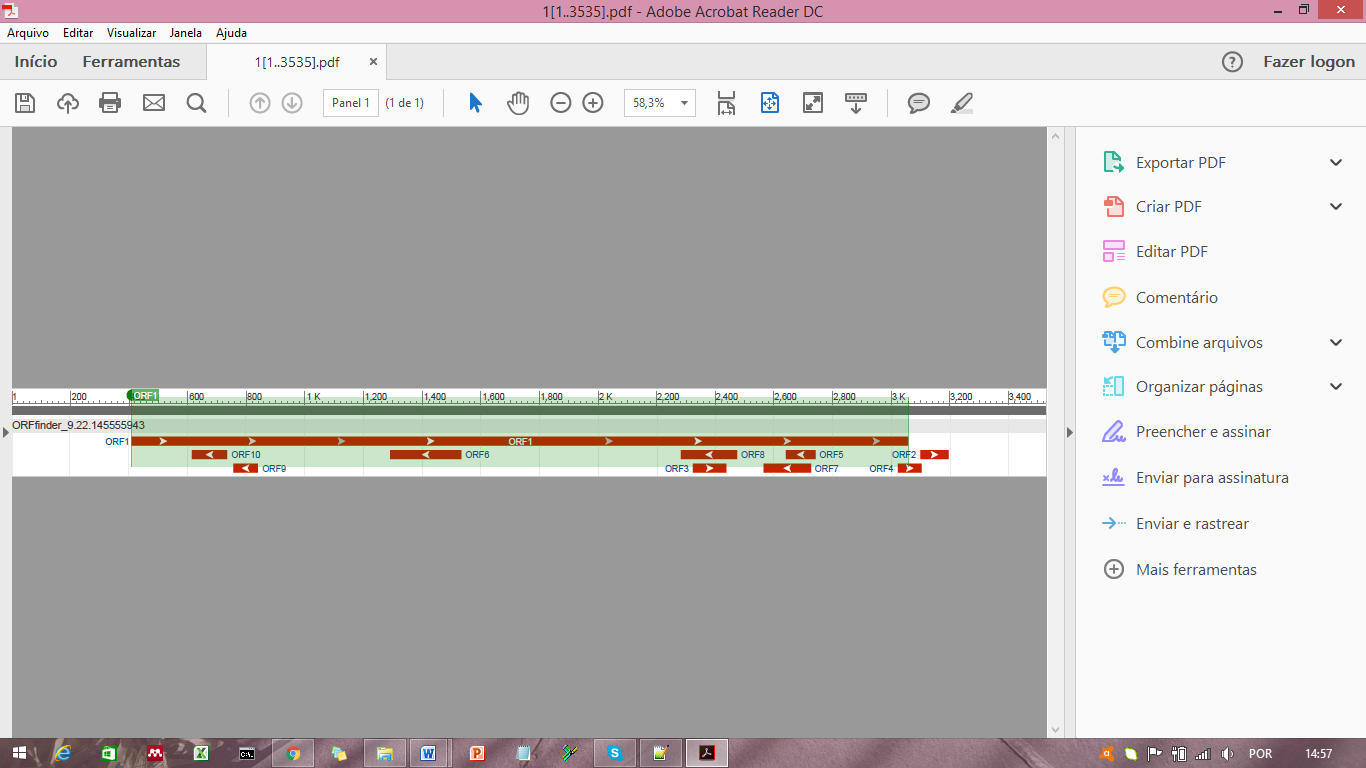


**BlastP (Non-redundant NCBI sequences)**

PREDICTED: exonuclease mut-7 homolog [Rhagoletis zephyria]

Sequence ID: [XP_017471030.1](https://www.ncbi.nlm.nih.gov/protein/1048021069?report=genbank&log$=protalign&blast_rank=1&RID=WAKT17J7014) Length: 881 Number of Matches: 1

Range 1: 1 to 878

Score: 1358 bits(3516)

E-value: 0.0

Identities: 651/881(74%)

Query 1 MSICRIPAGYEDDED--DLSEVMQKITVVPDITVGAGLNNENFDRNLSSEVASWFFFYKD 58

MS IPAGYEDDED LS+ MQKITVVPDIT+GAGLN ENFDR+LS +V +WF YK+

Sbjct 1 MSYRGIPAGYEDDEDWFSLSDGMQKITVVPDITIGAGLNRENFDRSLSPDVGNWFDGYKE 60

Query 59 EWNSYKRMPSAQHTLKAMLMTDEDPLLFALKLFANCPGCNNLKNKSLALFILETVCDLHK 118

WNSYKR PSAQH L MLM D DPLL ALKLFANCPGCNNLKNK+LAL+ILET+ DLHK

Sbjct 61 HWNSYKRTPSAQHNLIVMLMKDADPLLLALKLFANCPGCNNLKNKNLALYILETISDLHK 120

Query 119 TKPEISTNCTDNTRMIAFNFVKTCGIVPLCKAVISTYELKKIRDLLVPKIKDLIAAGHIK 178

P+IS TDNTRM+AFNFVKTCGIVPL KAV++TYEL +IR+LLVPKIK+LI GH K

Sbjct 121 QYPDISNKSTDNTRMVAFNFVKTCGIVPLYKAVMTTYELNRIRELLVPKIKELITHGHFK 180

Query 179 DAAHWTMHLQITHIFGIFDIVFPLILQDKVSLAEEYLNVAKDLALPTVKFLDSLLDKQKT 238

DAA+WTMHLQIT++F IFDI+FPLILQDKVSLAEEYL AKDL LPTV+FLDSLLDKQKT

Sbjct 181 DAAYWTMHLQITNLFDIFDIIFPLILQDKVSLAEEYLTAAKDLTLPTVQFLDSLLDKQKT 240

Query 239 VIDHCEEVLTKYEYKDVKCNILTYRPMSKLVTRLAKKYKIDAHDTPNLNFTKACSYLHYL 298

V++HC+E+LTKYEYKD+K N+L+YRPMSKLVTRLAKKYKID+ TPNLNFTK+CSY+HYL

Sbjct 241 VMEHCDEILTKYEYKDIKHNVLSYRPMSKLVTRLAKKYKIDSQYTPNLNFTKSCSYMHYL 300

Query 299 WRKYRDGGMSHDAWVELAHDAAVTKPIQMDLIKNVVAYGDIKEAGYWISFYKIPLEECPD 358

W KY+DGGMS +AW ELA DAA TKP+Q+DLI NV YGD +EA +W+ ++ + +ECP+

Sbjct 301 WHKYQDGGMSCEAWRELARDAASTKPLQLDLISNVATYGDYEEAIFWVKYFNLQKDECPN 360

Query 359 AVLDYLKQNRPYGTDKHNTSNKSSKSANSSRGAMNKSKRSNSFKNNQYLTLNLPIDCIII 418

+ Y+++NR + + ++ S ++ +S GAMNKSKRSNS N+YLTLNLPI+C++I

Sbjct 361 DIRQYMQKNR--SIEDMHNTSNESSNSANSAGAMNKSKRSNSLTANRYLTLNLPIECVLI 418

Query 419 VDDRVKFLQMLDHLEGQLLIAFDSEWKPTVCNENVISVLQMATMDRVYLLDCLSSNLSNE 478

+DDRVKFL MLD+LEGQLLIAFDSEWKPTVCNENVIS+LQMA MDRVYLLDCLS NLS++

Sbjct 419 IDDRVKFLDMLDYLEGQLLIAFDSEWKPTVCNENVISLLQMAIMDRVYLLDCLSPNLSDD 478

Query 479 LWQQLGRRVFNNLEILKIGFSLHQDLRMLHKSLPLELNLNAKTCYLDLRELWRRLKYLQI 538

LWQ+LGRRVFNNLEILKIGFSLHQDLRMLHKSLPL+LNLN+KTCYLDLRELWRRLKYLQI

Sbjct 479 LWQELGRRVFNNLEILKIGFSLHQDLRMLHKSLPLQLNLNSKTCYLDLRELWRRLKYLQI 538

Query 539 VKFPFECASNSGESLCTLTEICLGKKLDKSNQFSNWANRPLRHDQVIYAALDAHCLLLVY 598

VKFPFE NSGESLC L EICLGKKLDKSNQ SNWANRPLRHDQ++YAALDAHCLL VY

Sbjct 539 VKFPFESGFNSGESLCVLAEICLGKKLDKSNQCSNWANRPLRHDQIVYAALDAHCLLQVY 598

Query 599 QVVSEILVRMGLDVDQVVDEVTTGKTGYLFQKKRVITEQNDERSSTRPVKMDAEGVNSVS 658

QV+SEIL RMGLDVDQVVDEVTTGKTGYLF+ KRVI EQ D+ SS VKM+A+ V +V+

Sbjct 599 QVLSEILTRMGLDVDQVVDEVTTGKTGYLFRMKRVINEQFDDPSSG-VVKMNAQRVKAVN 657

Query 659 AKNENNRSIGTKFICDTMMTGLSKEFRKLGIDCVEIVNNDLGYYIELARKEHRYILTRDS 718

AK ENN I TKFICD+M+ GLSKE RKLGIDCVE++ +DL +YI++A++E+RY+LTRDS

Sbjct 658 AKTENNGGIRTKFICDSMLYGLSKELRKLGIDCVEVIKDDLDHYIDIAKRENRYVLTRDS 717

Query 719 RYDLFVNELPPEQCLQIPSDSSVDQVLNIIRLLDIKIYECNLFTRCLSCNSNEFIFALRH 778

RY+LFV+ELP E CLQIP+DSSV+QVLNI+RLLDIKIYE NLFTRCL+CNSNEFI AL+H

Sbjct 718 RYNLFVSELPQEHCLQIPTDSSVEQVLNILRLLDIKIYESNLFTRCLNCNSNEFILALKH 777

Query 779 EMQTMRFGQALDEIKDMSALNMNPYGKTYHLLNVSSHIVKTKTTYRGKFIKLNRIRSHIL 838

EMQ +R+GQALDE+KD S NM+PY K + LL V++H++K+KTTYRG+ IK+NRI+SHIL

Sbjct 778 EMQIIRYGQALDEMKDESGENMSPYEKNFQLLTVNNHVLKSKTTYRGRSIKVNRIKSHIL 837

Query 839 RSKEYFYICDNCGICTWDGAYSIHSSVKDAVLSDVFEAATA 879

RSKE+FYICDNCG+CTWDGA+SIHSSV + VL E A A

Sbjct 838 RSKEHFYICDNCGVCTWDGAHSIHSSVMENVLYGNCETAAA 878

**Saposin receptor**

**>lcl|ORF15**

MQRSFVLCAAIALLAGTLFVSATPMLGAKQCTWGPSFWCKNLTNAKGCHA

VRHCIQTVWETQNVPVDDDSICKICKDMVTQARDQLRSNETMEELKEVFE

GSCALIPIKIVKKECTKLADDFVPELVEALSSEMNPDQVCSVAGLCNNAR

IDELLQFYYQSALDGTLAEEKSDESKERKEVDAIAPQQQHLLTCSNCNHL

GSLITEKFNKANRDEILENILHLCGEMSSFSDACSNIVLTYFNDIYNHMK

EHLNAAGICHMSGSCAANYHKHAEDPTEPEDAVALASSLGDDIPCKLCEQ

LVQHLRDVLIANTTESEFKDVLHGLCNQTKGFREECNSLVEQYYDAIYNA

LVNNLDANGACFMIGVCPKGNAEAFKGEIRPLLPSVQPAEIKVTLRKLGA

NEPKFTQEEIHAMTLPIDTLMGAANPGLLVDNGELCTFCEYVLHYIQVEL

STSTTEDKIKDVVNNICSRLGRTLRGECHNFLDMYGDAVIALLVQGLNPR

EVCPKMKMCPANHENFDDVEIFAPKISKPSVSEMQHVDESDKPTCPLCLF

AVEQAQEKIKDNKSKANIKNVLDNLCAHLPSKLKSECVDFVETYMNELID

MLITDFKPQEICVAIKLCQNSSDELNELGISLESNSREEGVNEIDSNDSV

ELAFGRIEPPNCLLCEEVIKIAEKRIGKHTTKDEIKKSLDHSCDKLRKNL

RDKCHKYIAKYGDKIADLLVKEMAPKMICREIGLCIWSEQEDLDIDEALK

YDVVVLPGQKYAPHNDRFVGMDDMVNDPPTCVLCEFVMTKLESEIKNAST

QEEIKHTVENICKIMPKTVTKSCNKFIDQYTSTILALIGSVPPKMMCQQM

QLCFAGLDVVSDEVIECGVCHGATSALLPYFKQHLDHESITEYYMLLEGC

QVLDAKYYDICNHMIRTYGQSILNLAQGGETDESSICAKIGKCFSGEKSS

LAFARVSA

**Graphical representation – ORF**


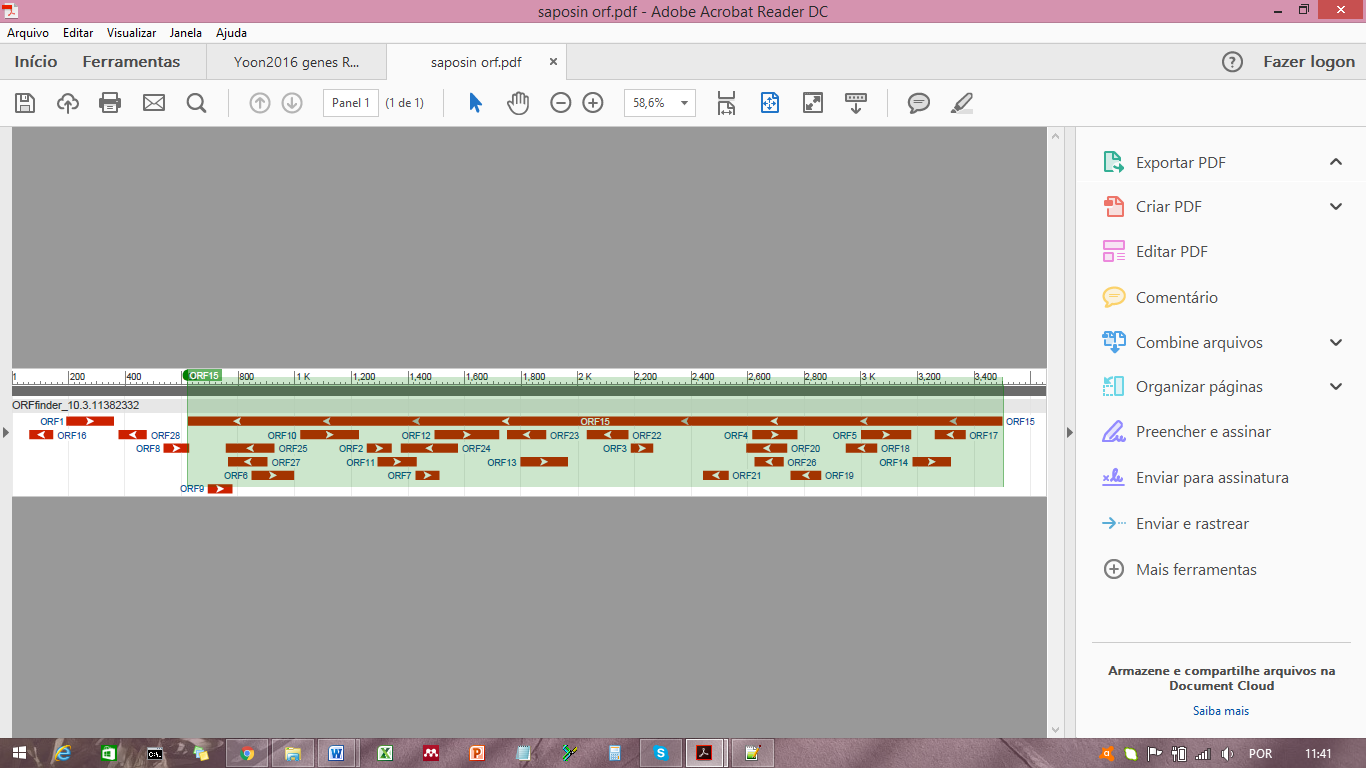


**BlastP (Non-redundant NCBI sequences)**

PREDICTED: prosaposin [Rhagoletis zephyria]

Sequence ID: [XP_017477135.1](https://www.ncbi.nlm.nih.gov/protein/1048040515?report=genbank&log$=protalign&blast_rank=1&RID=X787UKBA015) Length: 958 Number of Matches: 1

Score: 1741 bits(4509)

E-value: 0.0

Identities: 828/959(86%)

Query 1 MQRSFVLCAAIALLAGTLFVSATPMLGAKQCTWGPSFWCKNLTNAKGCHAVRHCIQTVWE 60

MQR FV C IALLAG +ATPMLGA+QCTWGPS+WCKNLTNAKGCHAVRHCIQTVWE

Sbjct 1 MQRGFVCCVVIALLAGAFLANATPMLGARQCTWGPSYWCKNLTNAKGCHAVRHCIQTVWE 60

Query 61 TQNVPVDDDSICKICKDMVTQARDQLRSNETMEELKEVFEGSCALIPIKIVKKECTKLAD 120

TQNVPVDDDSICKICKDMVTQARDQLRSNETMEELKEVFEGSC LIP+KIV+KEC KLAD

Sbjct 61 TQNVPVDDDSICKICKDMVTQARDQLRSNETMEELKEVFEGSCNLIPVKIVRKECDKLAD 120

Query 121 DFVPELVEALSSEMNPDQVCSVAGLCNNARIDELLQFYYQSALDGTLAEEKSDESKERKE 180

DFVPELVEALSS+MNPDQVCSVAGLCNNARIDEL+ +YQ+ALDGTL E+ S ESKE KE

Sbjct 121 DFVPELVEALSSQMNPDQVCSVAGLCNNARIDELMASHYQAALDGTLKED-SAESKESKE 179

Query 181 VDAIAP-QQQHLLTCSNCNHLGSLITEKFNKANRDEILENILHLCGEMSSFSDACSNIVL 239

+++ + QQHLLTC NCNHLGSLITEKFN+A+RDEILENILHLCGEMSSFSDACSNIVL

Sbjct 180 IESKSSVPQQHLLTCGNCNHLGSLITEKFNQADRDEILENILHLCGEMSSFSDACSNIVL 239

Query 240 TYFNDIYNHMKEHLNAAGICHMSGSCAANYHKHAEDPTEPEDAVALASSLGDDIPCKLCE 299

TYFNDIY+HM++HLNAAGICHMSGSCAANYHKHA+DP EPED VALAS+LGDDIPCKLCE

Sbjct 240 TYFNDIYDHMRQHLNAAGICHMSGSCAANYHKHADDPVEPEDPVALASTLGDDIPCKLCE 299

Query 300 QLVQHLRDVLIANTTESEFKDVLHGLCNQTKGFREECNSLVEQYYDAIYNALVNNLDANG 359

QLVQHLRDVLIANTTE+EFK VL+GLCNQTKGFREECNSLVEQYYD IYNAL NNLDANG

Sbjct 300 QLVQHLRDVLIANTTETEFKQVLNGLCNQTKGFREECNSLVEQYYDVIYNALANNLDANG 359

Query 360 ACFMIGVCPKGNAEAFKGEIRPLLPSVQPAEIKVTLRKLGANEPKFTQEEIHAMTLPIDT 419

ACFMIGVCPKGN +AFKGEIRPLLPS++PAEIKVTLRKLGANEPKFTQEEIHAMTLPIDT

Sbjct 360 ACFMIGVCPKGNNDAFKGEIRPLLPSIEPAEIKVTLRKLGANEPKFTQEEIHAMTLPIDT 419

Query 420 LMGAANPGLLVDNGELCTFCEYVLHYIQVELSTSTTEDKIKDVVNNICSRLGRTLRGECH 479

LMGAANPGLLVDNGELCTFCEY++HYIQVELST TTEDKIK+VVNN+CSRLGRTLRGECH

Sbjct 420 LMGAANPGLLVDNGELCTFCEYLIHYIQVELSTPTTEDKIKEVVNNVCSRLGRTLRGECH 479

Query 480 NFLDMYGDAVIALLVQGLNPREVCPKMKMCPANHENFDDVEIFAPKISKPSVSEMQHVDE 539

NF+DMYGDAVIALL+QGLNPRE+CPKM MCP NH+ FDDVEIFAP+ K + + + VDE

Sbjct 480 NFIDMYGDAVIALLIQGLNPREICPKMAMCPPNHDYFDDVEIFAPETPKVAATPARPVDE 539

Query 540 SDKPTCPLCLFAVEQAQEKIKDNKSKANIKNVLDNLCAHLPSKLKSECVDFVETYMNELI 599

SDKPTCPLCLFAV QAQ+KI DNKSKANIKNVLD+LCAHLP KL++ECVDFVETY NEL+

Sbjct 540 SDKPTCPLCLFAVAQAQQKIGDNKSKANIKNVLDHLCAHLPPKLQTECVDFVETYSNELV 599

Query 600 DMLITDFKPQEICVAIKLCQNSSDELNELGISLESNSREEGVNEIDSNDSVELAFGRIEP 659

D LI DFKPQEICV IKLC SSDEL+ELGISLE SRE+G+NEIDSNDSV++AFGRIEP

Sbjct 600 DKLIADFKPQEICVDIKLCATSSDELDELGISLEEKSREQGINEIDSNDSVDIAFGRIEP 659

Query 660 PNCLLCEEVIKIAEKRIGKHTTKDEIKKSLDHSCDKLRKNLRDKCHKYIAKYGDKIADLL 719

PNCLLCEE IKIAEK+IGK T+K EIKK+LDHSCDKLR N+R+KCHKY+AKYGDKIADLL

Sbjct 660 PNCLLCEEFIKIAEKKIGKRTSKQEIKKALDHSCDKLRLNVREKCHKYVAKYGDKIADLL 719

Query 720 VKEMAPKMICREIGLCIWSEQEDLDIDEALKYDVVVLPGQKYAPHNDRFVGMDDMVNDPP 779

VKEMAPKMICREIGLCIWSEQEDLDIDEALKYDVVVLP Q+ + HNDRFVGMDD+V +PP

Sbjct 720 VKEMAPKMICREIGLCIWSEQEDLDIDEALKYDVVVLPDQQISQHNDRFVGMDDIVKEPP 779

Query 780 TCVLCEFVMTKLESEIKNASTQEEIKHTVENICKIMPKTVTKSCNKFIDQYTSTILALIG 839

TCVLCEFVMTKLE+E++NASTQEEIKHTVENICKIMP+TVTKSCNKFID+Y +TIL+LIG

Sbjct 780 TCVLCEFVMTKLEAELRNASTQEEIKHTVENICKIMPRTVTKSCNKFIDEYINTILSLIG 839

Query 840 SVPPKMMCQQMQLCFAGLDVVSDEVIECGVCHGATSALLPYFKQHLDHESITEYYMLLEG 899

+VPPK+MCQQMQLCF GLDVVSDEVIECGVCHGATSALLPYFKQHLDH SITEYYMLLEG

Sbjct 840 TVPPKVMCQQMQLCFGGLDVVSDEVIECGVCHGATSALLPYFKQHLDHGSITEYYMLLEG 899

Query 900 CQVLDAKYYDICNHMIRTYGQSILNLAQGGETDESSICAKIGKCFSGEKSSLAFARVSA 958

CQ LDAKYYDICN MIRTYGQSILNLAQ GETDESSICAKIGKCFSGEKSSLAFA+VSA

Sbjct 900 CQALDAKYYDICNRMIRTYGQSILNLAQSGETDESSICAKIGKCFSGEKSSLAFAKVSA 958
